# Prediction of soil probiotics based on foundation model representation enhancement and stacked aggregation classifier

**DOI:** 10.1093/bib/bbaf567

**Published:** 2025-10-29

**Authors:** Qiang Kang, Haotong Sun, Yayu Wang, Xiaolong Fang, Yuxiang Li, Yong Zhang, Tong Wei, Peng Yin

**Affiliations:** BGI Research, No. 59, Keji 3rd Road, Jiangxia District, Wuhan 430074, China; BGI Research, No. 59, Keji 3rd Road, Jiangxia District, Wuhan 430074, China; School of Artificial Intelligence, University of Chinese Academy of Sciences, No. 19, Yuquan Road, Shijingshan District, Beijing 100049, China; BGI Research, No. 9, Yunhua Road, Yantian District, Shenzhen 518003, China; BGI Research, No. 9, Yunhua Road, Yantian District, Shenzhen 518003, China; BGI Research, No. 59, Keji 3rd Road, Jiangxia District, Wuhan 430074, China; BGI Research, No. 59, Keji 3rd Road, Jiangxia District, Wuhan 430074, China; BGI Research, No. 59, Keji 3rd Road, Jiangxia District, Wuhan 430074, China; BGI Research, No. 59, Keji 3rd Road, Jiangxia District, Wuhan 430074, China

**Keywords:** deep learning, foundation model, representation enhancement, stacked aggregation classifier, probiotics

## Abstract

Soil probiotics are indispensable in agro-ecosystems, enhancing crop yield through nutrient solubilization, pathogen suppression, and soil structure improvement. However, reliable prediction methods for soil probiotics are still lacking. In this study, we use genomic foundation models to generate representations from sample sequences and enhance them by deeply integrating domain-specific engineered features. The enhanced representations enable training a powerful classifier for a target task, rather than relying on conventional parameter fine-tuning. Inspired by the stacking ensemble learning framework, we design a stacked aggregation classifier. It predicts a sample’s label by leveraging only a subset of its sequence segments, effectively addressing the challenges in processing long or incompletely assembled sequences. The proposed method is applied to the prediction of soil probiotics and demonstrates excellent performance on both balanced and imbalanced test sets. Furthermore, potential functional genes are revealed from the predicted probiotics, providing valuable biological insights for related studies.

## Introduction

Soil probiotics, encompassing functionally active microbial consortia such as *Bacillus* and *Pseudomonas*, serve as indispensable regulators of agro-ecosystems [1, 2]. These microorganisms can drive biogeochemical cycles and enhance crop productivity through multifunctional mechanisms. *Azotobacter* has been reported to fix ~20 kg N ha^−1^ yr^−1^, contributing significantly to soil nitrogen content [3], while *P. fluorescens* can produce 2,4-diacetylphloroglucinol to suppress *Ralstonia solanacearum* and has the ability to solubilize phosphate [4]. In field applications, inoculation with cold-tolerant phosphate-solubilizing *Pseudomonas* strains can increase wheat yield by ~22% under natural growing conditions [5]. Targeted discovery and cultivation of soil probiotics are critical for sustainable agriculture, enabling innovations such as pesticides and fertilizers based on microbial consortia that synergistically improve soil resilience and crop health [6–8].

Traditional methods for identifying probiotics rely on culturing bacteria in selective media and analyzing their 16S rRNA gene sequences [9–12]. However, these methods cannot fully reveal the functional capabilities of probiotics, especially since only a small fraction of soil microorganisms can be grown under laboratory conditions [13, 14]. ​In recent years, artificial intelligence (AI) has made significant strides in life sciences, with various models being widely applied to species classification and identification tasks [15–17]. Compared to traditional methods, AI-driven methods can learn deep information from microbial genomic sequences, enabling the rapid and cost-effective identification of candidate strains with desired functional traits.​

For predicting probiotics, methods such as iProbiotics and metaProbiotics have been developed. iProbiotics is a machine learning-based tool that uses *k*-mer features and incremental feature selection to predict potential probiotic strains from genomic data [18]. metaProbiotics employs natural language processing techniques to represent DNA sequences as word vectors and uses random forest classifiers to predict probiotic-associated bins from metagenomic data [19]. However, these methods train the models using data from specific environments, such as the human gut. Given the substantial differences in microbial communities across environments (e.g. soil microbiomes exhibit greater complexity and diversity compared to human gut microbiomes [20]), these methods may not be directly applicable to soil microorganisms. Moreover, retraining these models by directly replacing the training data with soil microorganisms may result in limited effectiveness, as their original designs are inherently tailored to specific sample scales, data preprocessing protocols, and representation strategies for their intended tasks. Currently, there is a lack of AI-driven methods specifically designed for predicting soil probiotics, highlighting the necessity and significance of developing such a method.

Large language models, represented by BERT [21] and the GPT series [22, 23], have rapidly advanced in natural language processing. Their architectures have subsequently been adapted to decipher the genomic “language”, leading to the development of several foundation models. DNABERT is a pioneering model that applies the BERT architecture to DNA sequences using *k*-mer tokenization [24]. DNABERT-2 introduces improvements such as replacing *k*-mer tokenization with byte-pair encoding and incorporating attention with linear biases [25]. Evo comprises 7 billion parameters and is trained on 2.7 million prokaryotic and phage genomes [26]. Its enhanced version, Evo2, expands the parameters to a maximum of 40 billion and is trained on 9.3 trillion DNA base pairs from a highly curated genomic atlas spanning all domains of life [27]. Nucleotide Transformer represents a family of transformer-based models developed on integrated information from 3202 human genomes and 850 genomes from diverse species, with parameter sizes ranging from 50 million to 2.5 billion [28]. Additional models, such as HyenaDNA [29], GenomeBert [30], and FGeneBERT [31], demonstrate diverse applications, collectively underscoring the significant contributions of foundation models to genomics research.

The application of genomic foundation models to target tasks such as the prediction of probiotics typically relies on fine-tuning [21]. However, fine-tuning demands large-scale and high-quality data, and specialized expertise in architecture design and hyperparameter optimization​​, coupled with access to high-performance computing clusters (e.g. multi-GPU systems with NVLink interconnects) [32]. These requirements create barriers for users lacking professional support, such as biologists and clinicians. A critical technical limitation of fine-tuning is catastrophic forgetting [33], in which models rapidly discard previously learned information when adapting to new tasks, thereby reducing reliability in multitask applications. Although low-rank adaptation shows promise in preserving core knowledge [34], its implementation further increases technical complexity. Genome sequences of microorganisms often exceed the context window limitations of most Transformer-based architectures [35]. While Hyena-based architectures mitigate these limitations through implicitly parameterized long convolutions and data-controlled gating [29], they still fail to comprehensively cover the genomic sequences of many microorganisms. In addition, *de novo* genome assembly pipelines are prone to errors due to repetitive regions and sequencing biases, which may introduce inaccuracies prior to model training [36].

To address these challenges, we present a method based on foundation model representation enhancement and stacked aggregation classifier, applying it to the prediction of soil probiotics. We leverage the foundation models for inference only on the target task, followed by enhancement of their output representations by deeply integrating domain-specific engineered features, as an alternative to fine-tuning. It enables the training of a powerful classifier for the target task, significantly reduces computational resource requirements and time consumption, and remains easy to implement and more user-friendly. Furthermore, we split a sequence into segments and stack the enhanced representations of a subset of segments to represent the entire sample. It not only circumvents issues arising from processing long sequences and assembly errors but also enables reliable predictions even for samples with incomplete sequencing coverage. We conduct extensive experiments based on diverse foundation models with different parameter sizes and architectures to systematically evaluate the feasibility and effectiveness of the proposed method. We further explore potential functional genes and their enriched pathways from the predicted probiotics, providing actionable insights for subsequent wet-lab experiments.

## Materials and methods

### Overview of proposed method

**Figure 1:**
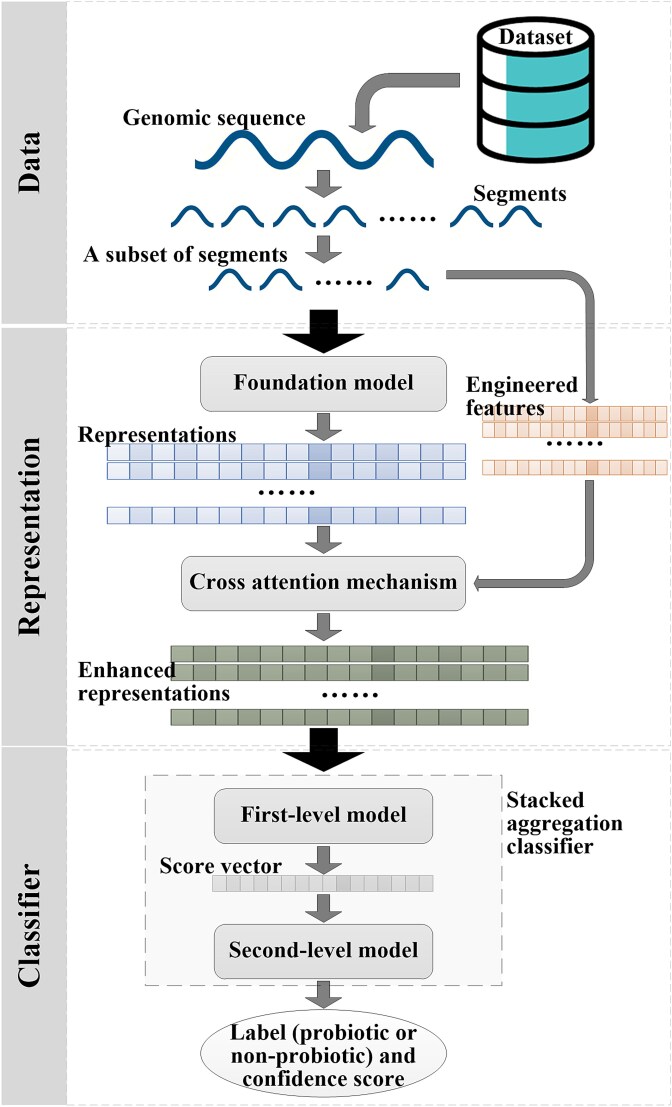
Overview of the proposed method. The genomic sequence of a bacterial sample is split into segments. A subset of segments is fed into a pretrained foundation model to generate representations, while engineered features are extracted from these segments. The foundation model representations and engineered features are aligned, and then the foundation model representations are enhanced by deeply integrating the engineered features via the cross-attention mechanism. The stacked aggregation classifier comprises two submodels. The first-level model processes the enhanced representations of each segment to obtain a score. All scores are aggregated into a vector, which is fed into the second-level model to output the predicted label (probiotic or non-probiotic) and confidence score.

The overview of the proposed method is shown in Fig. 1. For each bacterial sample, its genomic sequence is split into segments, and a subset of the segments is randomly selected. The selected segments are input into a pretrained foundation model to generate representations, while engineered features are extracted from these segments. The foundation model representations and engineered features are aligned, after which the engineered features are deeply integrated into the foundation model representations via the cross-attention mechanism to obtain the enhanced representations. The stacked aggregation classifier comprises two submodels. The first-level model processes each segment’s enhanced representations to output a score, and the scores from all segments are aggregated into a vector. This vector is then input into the second-level model, which outputs the predicted label (probiotic or non-probiotic) and confidence score.

### Datasets and preprocessing

All genomic sequences are downloaded from the National Center for Biotechnology Information (NCBI). Positive samples consist of reported soil probiotics, including diverse species such as *B. subtilis*, *B. amyloliquefaciens*, and *Paenibacillus peoriae*. Negative samples include reported soil pathogens, as well as bacteria belonging to the same species that show a high Average Nucleotide Identity with these pathogens. The details of all samples are shown in Supplementary Table S1. Each genomic sequence is split into 1000 bp segments with a 200 bp overlap between adjacent segments, and the final segment is discarded if its length < 1000 bp.

The completely random splitting scheme, the phylogenetically informed splitting scheme and the hierarchical clustering splitting scheme are employed to divide the samples into diverse datasets. The completely random splitting scheme is widely adopted [18, 19] and preserves the diversity of species in each dataset, facilitating the model’s learning from a broad range of information and its adaptation to various test scenarios. The species-level phylogenetically informed splitting scheme aims to reduce biological similarity between samples across different datasets by leveraging taxonomic information [37]. Concretely, the Genome Taxonomy Database (GTDB) provides a phylogenetically consistent and rank normalized genome-based taxonomy for prokaryotic genomes [38]. For each sample, its phylogenetic placement is retrieved from the GTDB according to its Genome Assembly Accession (GCA). This enables us to assign each sample to a specific taxonomic cluster in the GTDB. Different clusters may exhibit differences in phylogenetic relationships, genomic features, functional roles, etc. Then, the samples are divided into different datasets at the cluster level. This scheme provides a more stringent evaluation of model generalization, particularly for predicting microorganisms from phylogenetically distinct species. The hierarchical clustering splitting scheme aims to reduce sequence similarity between samples across different datasets. We use Mash v2.3 [39], a MinHash-based genomic distance assessment tool, to compute pairwise distances for all sample pairs and construct an all-against-all distance matrix. We then perform hierarchical clustering on this matrix to assign the samples to 20 clusters and divide these samples into different datasets at the cluster level. This scheme offers a stricter and taxonomy-agnostic assessment of model generalization, especially for predicting unseen genomes.

We design Experimental Groups 1, 2, and 3, which use the completely random splitting scheme, the phylogenetically informed splitting scheme, and the hierarchical clustering splitting scheme to divide all samples into the training and test sets, respectively. In each experimental group, the training set is further randomly divided into two subsets, which are used to train the cross-attention mechanism and the stacked aggregation classifier, respectively. The test sets are used to evaluate and compare the performance of different methods, where the imbalanced test set simulates the real soil environment that probiotics constitute a minority. In addition, to conduct the ablation experiment, the training subset 2 in Experimental Group 1 also serves as a cross-validation set. For five-fold cross-validation, the samples in the cross-validation set are divided into five parts using three schemes, respectively. In each fold, four parts are used for training and the remaining one is used for validation. This process is repeated five times to ensure each part is used for validation exactly once. Thus, five sets of results are obtained, which can be used to perform statistical analysis. The details of the datasets are shown in Table 1 and more details on sample division are shown in Supplementary Table S2.

**Table 1:** Details of datasets.

**Experimental Group**	**Dataset**	**#Pos**	**#Seg_Pos**	**#Neg**	**#Seg_Neg**
1	Training subset 1	200	1 284 328	200	1 379 878
	Training subset 2 (Cross-validation set)	120	726 088	120	839 687
	Balanced test set	80	518 285	80	557 708
	Imbalanced test set	18	115 293	180	1 265 553
2	Training subset 1	232	1 491 101	246	1 566 228
	Training subset 2	157	999 404	162	1 077 676
	Imbalanced test set	29	153 489	172	1 398 922
3	Training subset 1	231	1 458 757	243	1 812 195
	Training subset 2	161	1 007 840	162	1 168 579
	Imbalanced test set	26	177 397	175	1 062 052

#Pos: number of positive samples, #Neg: number of negative samples, #Seg_Pos: number of segments from positive samples, #Seg_Neg: number of segments from negative samples.

### Foundation model representation generation

Three cutting-edge genomic foundation models with different parameter sizes and architectures, Nucleotide Transformer-50M [28], DNABERT-2-117M [25], and EVO-7B [26], are used to generate diverse representations. Each sequence segment is tokenized in a manner i.e. specific to each foundation model. Nucleotide Transformer-50M primarily uses 6-mer tokens, with single-nucleotide tokens at sequence-edge positions. DNABERT-2-117M employs a byte-pair encoding tokenizer. EVO-7B treats each nucleotide as an individual token. These tokens are then fed into the model encoder to produce contextual embeddings, forming a feature matrix in which each row corresponds to one token’s feature vector. The embedding of the entire sequence is derived from the first positional token, which ​​integrates​​ global sequence information through the attention mechanism, serving as the representation. Given an input sequence segment *Seq* = *n*^1^*n*^2^…*n*^1000^ (where *n^i^*∈{*A*,*T*,*C*,*G*} denotes the *i*-th nucleotide), Nucleotide Transformer-50M​​ generates the 512-dimensional representation vector as ***R****_nt_* = [*r_nt_*^1^,*r_nt_*^2^,…,*r_nt_*^512^], ​​DNABERT-2-117M​​ produces the 768-dimensional representation vector as ***R****_db_* = [*r_db_*^1^,*r_db_*^2^,…,*r_db_*^768^], and ​​EVO-7B​​ constructs the 4096-dimensional representation vector as ***R****_evo_* = [*r_evo_*^1^,*r_evo_*^2^,…,*r_evo_*^4096^], where *r_nt_^i^*, *r_db_^j^* and *r_evo_^k^* denote the representation values at the *i*-th, *j*-th and *k*-th dimensions of ***R****_nt_*, ***R****_db_*, and ***R****_evo_*, respectively. For each sample, the corresponding three foundation model representations can be expressed as:


(1) \begin{equation*}\boldsymbol{SR}_{nt} =\left[\begin{array}{c} \boldsymbol{R}_{nt}{}^{1} \\ \boldsymbol{R}_{nt}{}^{2} \\ \vdots \\ \boldsymbol{R}_{nt}{}^{m}\end{array}\right]=\left[\begin{array}{cccc} r_{nt}{}^{1,1} & r_{nt}{}^{1,2} & \cdots & r_{nt}{}^{1,512} \\ r_{nt}{}^{2,1} & r_{nt}{}^{2,2} & \cdots & r_{nt}{}^{2,512} \\ \vdots & \vdots & \ddots & \vdots \\ r_{nt}{}^{m,1} & r_{nt}{}^{m,2} & \cdots & r_{nt}{}^{m,512}\end{array}\right]\end{equation*}

(2) \begin{equation*}\boldsymbol{SR}_{db} =\left[\begin{array}{c} \boldsymbol{R}_{db}{}^{1} \\ \boldsymbol{R}_{db}{}^{2} \\ \vdots \\ \boldsymbol{R}_{db}{}^{m}\end{array}\right]=\left[\begin{array}{cccc} r_{db}{}^{1,1} & r_{db}{}^{1,2} & \cdots & r_{db}{}^{1,768} \\ r_{db}{}^{2,1} & r_{db}{}^{2,2} & \cdots & r_{db}{}^{2,768} \\ \vdots & \vdots & \ddots & \vdots \\ r_{db}{}^{m,1} & r_{db}{}^{m,2} & \cdots & r_{db}{}^{m,768}\end{array}\right]\end{equation*}

(3) \begin{equation*}\boldsymbol{SR}_{evo} =\left[\begin{array}{c} \boldsymbol{R}_{evo}{}^{1} \\ \boldsymbol{R}_{evo}{}^{2} \\ \vdots \\ \boldsymbol{R}_{evo}{}^{m}\end{array}\right]=\left[\begin{array}{cccc} r_{evo}{}^{1,1} & r_{evo}{}^{1,2} & \cdots & r_{evo}{}^{1,4096} \\ r_{evo}{}^{2,1} & r_{evo}{}^{2,2} & \cdots & r_{evo}{}^{2,4096} \\ \vdots & \vdots & \ddots & \vdots \\ r_{evo}{}^{m,1} & r_{evo}{}^{m,2} & \cdots & r_{evo}{}^{m,4096}\end{array}\right]\end{equation*}

where ***R****_nt_^l^*, ***R****_db_^l^*, and ***R****_evo_^l^* denote the different representations of the *l*-th segment, and *m* denotes the number of segments.

### Engineered feature extraction

Domain-specific engineered features, *k*-mer and *g*-gap, are extracted from each sequence segment. The *k*-mer features represent the frequency of consecutive *k* nucleotides (e.g. a 3-mer category can be AGC), while the *g*-gap features represent the frequency of discontinuous nucleotides separated by *g* gaps (e.g. a 2-gap category can be A**C, where “*” represents any nucleotide) [40]. Consequently, each *k*-mer category corresponds to 4*^k^* features, and each *g*-gap category corresponds to 4^2^ features. A sliding window is applied to scan each sequence segment, recording the occurrence counts of each *k*-mer category and *g*-gap category. These counts are normalized to generate corresponding feature values as:


(4) \begin{equation*} {f_{k- mer}}^i=\frac{{c_{k- mer}}^i}{\sum_{i=1}^{4^k}{c_{k- mer}}^i}=\frac{{c_{k- mer}}^i}{L-k+1} \end{equation*}

(5) \begin{equation*} {f_{g- gap}}^j=\frac{{c_{g- gap}}^j}{\sum_{j=1}^{4^2}{c_{g- gap}}^j}=\frac{{c_{g- gap}}^j}{L-g-1} \end{equation*}

where *c_k-mer_^i^* denotes the count of the *i*-th *k*-mer category, *c_g-gap_^j^* denotes the count of the *j*-th *g*-gap category, and *L* denotes the sequence segment length. Given a sequence segment, 1-, 2-, and 3-mer features and 1-, 2-, and 3-gap features are extracted to form a 132-dimensional vector ***F*** = [*f*^1^,*f*^2^,…,*f*^132^] = [*f_k-mer_*^1^,*f_k-mer_*^2^,…,*f_k-mer_*^84^,*f_g-gap_*^1^,*f_g-gap_*^2^,…,*f_g-gap_*^48^]. For each sample, the corresponding engineered features can be expressed as:


(6) \begin{eqnarray*} \boldsymbol{SF}=\left[\begin{array}{c}{\boldsymbol{F}}^1\\{}{\boldsymbol{F}}^2\\ \vdots \\{}{\boldsymbol{F}}^m\end{array}\right]=\left[\begin{array}{cccc}{f}^{1,1}& {f}^{1,2}& \cdots & {f}^{\mathrm{1,132}}\\{}{f}^{2,1}& {f}^{2,2}& \cdots & {f}^{\mathrm{2,132}}\\ \vdots & \vdots & \ddots & \vdots \\{}{f}^{m,1}& {f}^{m,2}& \cdots & {f}^{m,132}\end{array}\right]\ =\left[\begin{array}{cccccccc}{f_{k-mer}}^{1,1}& {f_{k-mer}}^{1,2}& \cdots & {f_{k-mer}}^{1,84}& {f_{g-gap}}^{1,1}& {f_{g-gap}}^{1,2}& \cdots & {f_{g-gap}}^{1,48}\\{}{f_{k- mer}}^{2,1}& {f_{k-mer}}^{2,2}& \cdots & {f_{k-mer}}^{2,84}& {f_{g-gap}}^{2,1}& {f_{g-gap}}^{2,2}& \cdots & {f_{g- gap}}^{2,48}\\ \vdots & \vdots & \ddots & \vdots & \vdots & \vdots & \ddots & \vdots \\{}{f_{k- mer}}^{m,1}& {f_{k- mer}}^{m,2}& \cdots & {f_{k- mer}}^{m,84}& {f_{g- gap}}^{m,1}& {f_{g- gap}}^{m,2}& \cdots & {f_{g- gap}}^{m,48}\end{array}\right] \end{eqnarray*}

where ***F****^l^* denotes the engineered feature vector of the *l*-th segment.

### Foundation model representation enhancement

To enhance the foundation model representations by integrating engineered features, their dimensions and scales need to be aligned. For each sample’s ***SF***, global Min-Max Normalization is applied to standardize its scale as:


(7) \begin{equation*} {f}^{\prime i,j}=\frac{f^{i,j}-\mathit{\min}(\boldsymbol{SF})}{\mathit{\max}(\boldsymbol{SF})-\mathit{\min}(\boldsymbol{SF})} \end{equation*}

(8) \begin{equation*} \boldsymbol{S{F}}^{\prime }=\left[\begin{array}{c}{\boldsymbol{F}}^{\prime 1}\\{}{\boldsymbol{F}}^{\prime 2}\\ \vdots \\{}{\boldsymbol{F}}^{\prime m}\end{array}\right]=\left[\begin{array}{cccc}{f}^{\prime 1,1}& {f}^{\prime 1,2}& \cdots & {f}^{\prime \mathrm{1,132}}\\{}{f}^{\prime 2,1}& {f}^{\prime 2,2}& \cdots & {f}^{\prime \mathrm{2,132}}\\ \vdots & \vdots & \ddots & \vdots \\{}{f}^{\prime m,1}& {f}^{\prime m,2}& \cdots & {f}^{\prime m,132}\end{array}\right] \end{equation*}

where *f^i,j^* denotes the feature value at the *i*-th row and *j*-th column in ***SF*** and *f’^i,j^* is its normalized value, min(), and max() denote the minimum and ​​maximum​​ functions, respectively. For each sample’s ***SR****_nt_*, ***SR****_db_*, and ***SR****_evo_*, the following process is performed: (i) their value ranges are unified through global Min-Max Normalization similar to Eqs. (7) and (8); (ii) each representation vector (i.e. each row) is reduced to 132 dimensions via kernel Principal Component Analysis (PCA) [41], ensuring consistency with the dimensionality of each feature vector in ***SF****’*; (iii) global Min-Max Normalization is performed again to ensure scale consistency with ***SF****’*. The processed representations can be obtained as:


(9) \begin{equation*} {\boldsymbol{SR}_{nt}}^{\prime }=\left[\begin{array}{c}{\boldsymbol{R}_{nt}}^{\prime 1}\\{}{\boldsymbol{R}_{nt}}^{\prime 2}\\ \vdots \\{}{\boldsymbol{R}_{nt}}^{\prime m}\end{array}\right]=\left[\begin{array}{cccc}{r_{nt}}^{\prime 1,1}& {r_{nt}}^{\prime 1,2}& \cdots & {r_{nt}}^{\prime \mathrm{1,132}}\\{}{r_{nt}}^{\prime 2,1}& {r_{nt}}^{\prime 2,2}& \cdots & {r_{nt}}^{\prime \mathrm{2,132}}\\ \vdots & \vdots & \ddots & \vdots \\{}{r_{nt}}^{\prime m,1}& {r_{nt}}^{\prime m,2}& \cdots & {r_{nt}}^{\prime m,132}\end{array}\right] \end{equation*}

(10) \begin{equation*} {\boldsymbol{SR}_{db}}^{\prime }=\left[\begin{array}{c}{\boldsymbol{R}_{db}}^{\prime 1}\\{}{\boldsymbol{R}_{db}}^{\prime 2}\\ \vdots \\{}{\boldsymbol{R}_{db}}^{\prime m}\end{array}\right]=\left[\begin{array}{cccc}{r_{db}}^{\prime 1,1}& {r_{db}}^{\prime 1,2}& \cdots & {r_{db}}^{\prime \mathrm{1,132}}\\{}{r_{db}}^{\prime 2,1}& {r_{db}}^{\prime 2,2}& \cdots & {r_{db}}^{\prime \mathrm{2,132}}\\ \vdots & \vdots & \ddots & \vdots \\{}{r_{db}}^{\prime m,1}& {r_{db}}^{\prime m,2}& \cdots & {r_{db}}^{\prime m,132}\end{array}\right] \end{equation*}

(11) \begin{equation*} {\boldsymbol{SR}_{evo}}^{\prime }=\left[\begin{array}{c}{\boldsymbol{R}_{evo}}^{\prime 1}\\{}{\boldsymbol{R}_{evo}}^{\prime 2}\\ \vdots \\{}{\boldsymbol{R}_{evo}}^{\prime m}\end{array}\right]=\left[\begin{array}{cccc}{r_{evo}}^{\prime 1,1}& {r_{evo}}^{\prime 1,2}& \cdots & {r_{evo}}^{\prime \mathrm{1,132}}\\{}{r_{evo}}^{\prime 2,1}& {r_{evo}}^{\prime 2,2}& \cdots & {r_{evo}}^{\prime \mathrm{2,132}}\\ \vdots & \vdots & \ddots & \vdots \\{}{r_{evo}}^{\prime m,1}& {r_{evo}}^{\prime m,2}& \cdots & {r_{evo}}^{\prime m,132}\end{array}\right] \end{equation*}

Since foundation model representations and engineered features exist in ​​mismatched dimensional spaces, nonlinear integration methods are required. The cross-attention mechanism can ​​dynamically bridge heterogeneous representations​​/features through Query, Key, and Value interactions [42]. Although the cross-attention mechanism introduces an additional training step compared to simple integration methods, the number of trainable parameters required is far smaller​​ than that needed for fine-tuning a foundation model. For the *l*-th segment, its feature vector is integrated into the three representation vectors based on the multi-head cross-attention mechanism, respectively. Taking ***SR****_nt_’* as an example (the process is similar for ***SR****_db_’* and ***SR****_evo_’*), for each head, Query is derived from the foundation model representation ***R****_nt_’^l^*, while Key and Value are derived from the engineered feature ***F**’^l^*. The cross-attention is expressed as:


(12) \begin{equation*} {\boldsymbol{Q}}_{nt}={\boldsymbol{R}_{nt}}^{\prime l}{\boldsymbol{W}_{nt}}^Q,\kern1em {\boldsymbol{K}}_{nt}={\boldsymbol{F}}^{\prime l}{\boldsymbol{W}_{nt}}^K,\kern1em {\boldsymbol{V}}_{nt}={\boldsymbol{F}}^{\prime l}{\boldsymbol{W}_{nt}}^V \end{equation*}

(13) \begin{equation*} {\boldsymbol{CA}}_{nt}=\mathrm{softmax}\left(\frac{\boldsymbol{Q}_{nt}{\boldsymbol{K}_{nt}}^\mathrm{T}}{\sqrt{d}}\right){\boldsymbol{V}}_{nt} \end{equation*}

where ***W****_nt_^Q^*, ***W****_nt_^K^*, and ***W****_nt_^V^*∈ℝ^132 × *d*^ are the trainable weight matrices with *d* = 132/*h*, *h* is the number of heads, and the output ***CA****_nt_* is a *d*-dimensional vector. The outputs of all heads are concatenated and linearly projected by a trainable matrix ***W****_nt_^O^*∈ℝ^132 × 132^, and then combined with the original input via a residual connection ​as:


(14) \begin{equation*} {\boldsymbol{R}_{nt}}^{\prime \prime }=\mathrm{Concat}\left({\boldsymbol{CA}_{nt}}^1,{\boldsymbol{CA}_{nt}}^2,\dots, {\boldsymbol{CA}_{nt}}^h\right){\boldsymbol{W}_{nt}}^O+{\boldsymbol{R}_{nt}}^{\prime } \end{equation*}

where ***CA****_nt_^i^* denotes the output vector from the *i*-th head, and ***R****_nt_"* is the enhanced representation vector with 132 dimensions. For each sample, the corresponding enhanced representation can be expressed as:


(15) \begin{equation*} {\boldsymbol{SR}_{nt}}^{\prime \prime }=\left[\begin{array}{c}{\boldsymbol{R}_{nt}}^{\prime \prime 1}\\{}{\boldsymbol{R}_{nt}}^{\prime \prime 2}\\ \vdots \\{}{\boldsymbol{R}_{nt}}^{\prime \prime m}\end{array}\right]=\left[\begin{array}{cccc}{r_{nt}}^{\prime \prime 1,1}& {r_{nt}}^{\prime \prime 1,2}& \cdots & {r_{nt}}^{\prime \prime \mathrm{1,132}}\\{}{r_{nt}}^{\prime \prime 2,1}& {r_{nt}}^{\prime \prime 2,2}& \cdots & {r_{nt}}^{\prime \prime \mathrm{2,132}}\\ \vdots & \vdots & \ddots & \vdots \\{}{r_{nt}}^{\prime \prime m,1}& {r_{nt}}^{\prime \prime m,2}& \cdots & {r_{nt}}^{\prime \prime m,132}\end{array}\right] \end{equation*}

where ***R****_nt_"^l^* denotes the enhanced representation of the *l*-th segment.

### Stacked aggregation classifier

Each sample is represented by an *m* × 132 matrix, which needs to be converted into a vector before being fed into a classifier. The stacking ensemble learning framework integrates the outputs of multiple base learners to generate an ensemble vector, which is then fed into a meta-learner for the final output [43]. Inspired by this, we design a stacked aggregation classifier consisting of two submodels. A fixed number of segments are randomly selected from each sample. The enhanced representation vector of each segment is input into the first-level model (similar to the base learner) to produce a score. All scores are aggregated into a vector, which is then input into the second-level model (similar to the meta-learner) to output the label and confidence score.

The eXtreme Gradient Boosting​​ (XGBoost) model is used as the first-level model. Its gradient-boosted tree architecture recursively learns interactions between representations, effectively modeling nonlinear dependencies across sequence segments. The second-level model is a Logistic Regression (LR) model, which applies L1/L2 regularization to stabilize predictions while maintaining computational efficiency. This stacked aggregation classifier not only represents each sample by a subset of its sequence segments but also has the potential to achieve high accuracy and robustness.

### Cascade training strategy and inference process

The proposed method involves training in two modules, i.e. the cross-attention mechanism and the stacked aggregation classifier. The former aims to establish correlations between foundation model representations and engineered features, while the latter builds decision boundaries on the enhanced representations. Given their distinct training objectives, they are trained separately using different datasets. Compared with joint training, this separate training strategy is simpler to implement, reduces training time, and allows each module to be extended independently.

Because the cross-attention mechanism does not directly produce predictions for loss computation, a Multilayer Perceptron (MLP) is temporarily attached to provide loss signals during training. This MLP is used solely to optimize the cross-attention mechanism and is discarded after training is completed. It cannot replace the role of the stacked aggregation classifier, which requires training two submodels to perform the final prediction. The loss function for the cross-attention mechanism with the MLP is defined as:


(16) \begin{equation*} loss=0.8\times CEL+0.2\times MSE \end{equation*}

where *CEL* denotes the cross-entropy loss between the predicted confidence scores output by the MLP and the labels of the input samples, and *MSE* denotes the mean squared error between the reconstructed features obtained by the MLP from the cross-attention output and the input engineered features. This reconstruction term encourages the model to focus on engineered features, preventing over-reliance on foundation model representations. For the stacked aggregation classifier, the XGBoost and LR models employ their default loss functions.

**Figure 2:**
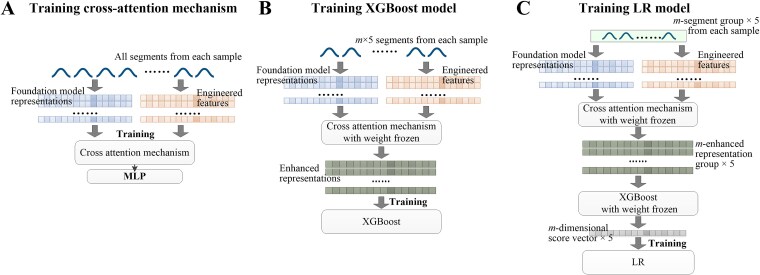
Cascade training strategy. The models are trained sequentially, with earlier-trained models assisting the training of subsequent ones. (A) The cross-attention mechanism with an attached MLP is trained using the aligned foundation model representations and engineered features of all segments from each sample. After this training is completed, the weights of the cross-attention mechanism are frozen. (B) *m* × 5 segments are randomly selected from each sample. Their enhanced representations are obtained based on the cross-attention mechanism. The XGBoost model is trained using these enhanced representations. After this training is completed, the weights of the XGBoost model are frozen. (C) m segments are selected five times with replacement from each sample, and the selected segments are not entirely identical at different times and are completely different from those used to train the XGBoost model. Their corresponding five groups of enhanced representations are obtained based on the cross-attention mechanism. These enhanced representations are fed into the XGBoost model to generate the five *m*-dimensional score vectors. The LR model is trained using these vectors. After this training is completed, the weights of the LR model are frozen.

We adopt a cascade training strategy in which the models are trained sequentially, with earlier-trained models assisting the training of subsequent ones (Fig. 2). Each sequence segment inherits the label from its source sample. The first stage is the training of the cross-attention mechanism. The foundation model representations and engineered features of all segments from each sample are obtained and aligned. They are input into the cross-attention mechanism with an attached MLP for training (Fig. 2A). After this training is completed, the weights of the cross-attention mechanism are frozen. The second stage is the training of the stacked aggregation classifier. We randomly select *m* × 5 segments from each sample (*m* denotes the number of segments selected per sample during inference, which is expanded five-fold here to augment the training data) and obtain their enhanced representations based on the cross-attention mechanism. These enhanced representations are input into the XGBoost model for training (Fig. 2B). After this training is completed, the weights of the XGBoost model are frozen. Subsequently, *m* segments are selected five times with replacement from each sample, ensuring that the selected segments are not entirely identical at different times and are completely different from those used to train the XGBoost model. This procedure can also augment the training data when the number of training samples is limited. Five *m*-enhanced representation groups are obtained based on the cross-attention mechanism and separately fed into the XGBoost model, generating five corresponding *m*-dimensional score vectors. These vectors are input into the LR model for training (Fig. 2C). After this training is completed, the weights of the LR model are frozen. With this, the cascade training is fully completed.

In the inference process, only *m* segments need to be randomly selected from each target sample, through which the proposed method can obtain: (i) foundation model representations, (ii) extracted engineered features, (iii) enhanced representations obtained based on the cross-attention mechanism, and (iv) predicted label and confidence score from the stacked aggregation classifier. For long-sequence samples containing abundant segments, multiple groups of *m* segments can be randomly selected for multiple independent predictions, the results of which can be considered and assessed comprehensively.

### Gene function annotation and enrichment analysis

For a target sample, genes are obtained using Prodigal v2.6.3 [44]. These genes are then annotated with KO identifiers using KofamScan v1.3.0 [45], configured with KEGG release 114. The resulting KO identifiers are filtered according to the criteria: (i) null threshold, (ii) score < threshold, and (iii) *e*-value>1e-5. The filtered KO identifiers are mapped to functions from the KEGG ORTHOLOGY database [46]. These functional annotations are cross-referenced with reported phenotypes of the target sample from public literature to infer which genes may critically influence specific functional traits. Enrichment analysis is performed using ReporterScore v0.1.9 [47], also configured with KEGG release 114, to identify pathways significantly enriched in these potential functional genes.

### Evaluation criteria

The evaluation criteria used for​ experiments​​ on the ​balanced dataset​​ include recall (REC), precision (PRE), specificity (SPE), accuracy (ACC), F1-score (F1_S), Matthews Correlation Coefficient (MCC), and Area Under the Curve (AUC) of the Receiver Operating Characteristic (ROC) curve. For experiments on the ​​imbalanced dataset​​, the evaluation criteria include REC, SPE, F1_S, Geometric mean of Recall and Specificity (GRS), and AUC. The related equations are expressed as:


(17) \begin{equation*} \mathrm{REC}=\frac{\mathrm{TP}}{\mathrm{TP}+\mathrm{FN}} \end{equation*}

(18) \begin{equation*} \mathrm{PRE}=\frac{\mathrm{TP}}{\mathrm{TP}+\mathrm{FP}} \end{equation*}

(19) \begin{equation*} \mathrm{SPE}=\frac{\mathrm{TN}}{\mathrm{TN}+\mathrm{FP}} \end{equation*}

(20) \begin{equation*} \mathrm{ACC}=\frac{\mathrm{TP}+\mathrm{TN}}{\mathrm{TP}+\mathrm{TN}+\mathrm{FP}+\mathrm{FN}} \end{equation*}

(21) \begin{equation*} \mathrm{F}1\_\mathrm{S}=2\times \frac{\mathrm{REC}\times \mathrm{PRE}}{\mathrm{REC}+\mathrm{PRE}}=\frac{2\mathrm{TP}}{2\mathrm{TP}+\mathrm{FP}+\mathrm{FN}} \end{equation*}

(22) \begin{equation*} \mathrm{MCC}=\frac{\mathrm{TP}\times \mathrm{TN}-\mathrm{FP}\times \mathrm{FN}}{\sqrt{\left(\mathrm{TP}+\mathrm{FP}\right)\left(\mathrm{TP}+\mathrm{FN}\right)\left(\mathrm{TN}+\mathrm{FP}\right)\left(\mathrm{TN}+\mathrm{FN}\right)}} \end{equation*}

(23) \begin{equation*} \mathrm{GRS}=\sqrt{\mathrm{REC}\times \mathrm{SPE}}=\sqrt{\frac{\mathrm{TP}}{\mathrm{TP}+\mathrm{FN}}\times \frac{\mathrm{TN}}{\mathrm{TN}+\mathrm{FP}}} \end{equation*}

where True Positive (TP) denotes the number of correctly predicted probiotics, False Negative (FN) denotes the number of incorrectly predicted probiotics, False Positive (FP) denotes the number of incorrectly predicted non-probiotics, and True Negative (TN) denotes the number of correctly predicted non-probiotics. For multiple independent experiments, we calculate the mean and standard deviation values of the results to evaluate the performance and stability of each method, and conduct statistical analysis to assess the significance of differences among the results of different methods.

### Project implementation

The main scripts are developed in a Python v3.8.15 virtual environment on a computing node equipped with four NVIDIA A100 40GB GPUs and 256GB RAM. Mash (implemented in C++) and Prodigal (implemented in C) are compiled with the GNU Compiler Collection v14.2.0. KofamScan is implemented in Ruby v3.2.2. ReporterScore is implemented in R v4.4.0.

The number of heads in the multi-head cross-attention mechanism is set to 11. In the MLP, the first linear layer maps the input from 132 dimensions to 64 dimensions, followed by the ReLU activation function, and the second linear layer reduces the 64-dimensional input to two dimensions. Mash, Prodigal, KofamScan, and ReporterScore are used with default settings. The hyperparameter settings of the three foundation models are provided in Supplementary Table S3, and those of XGBoost and LR are shown in Supplementary Table S4.

## Results

### Investigation on number of segments for representing a sample

**Figure 3:**
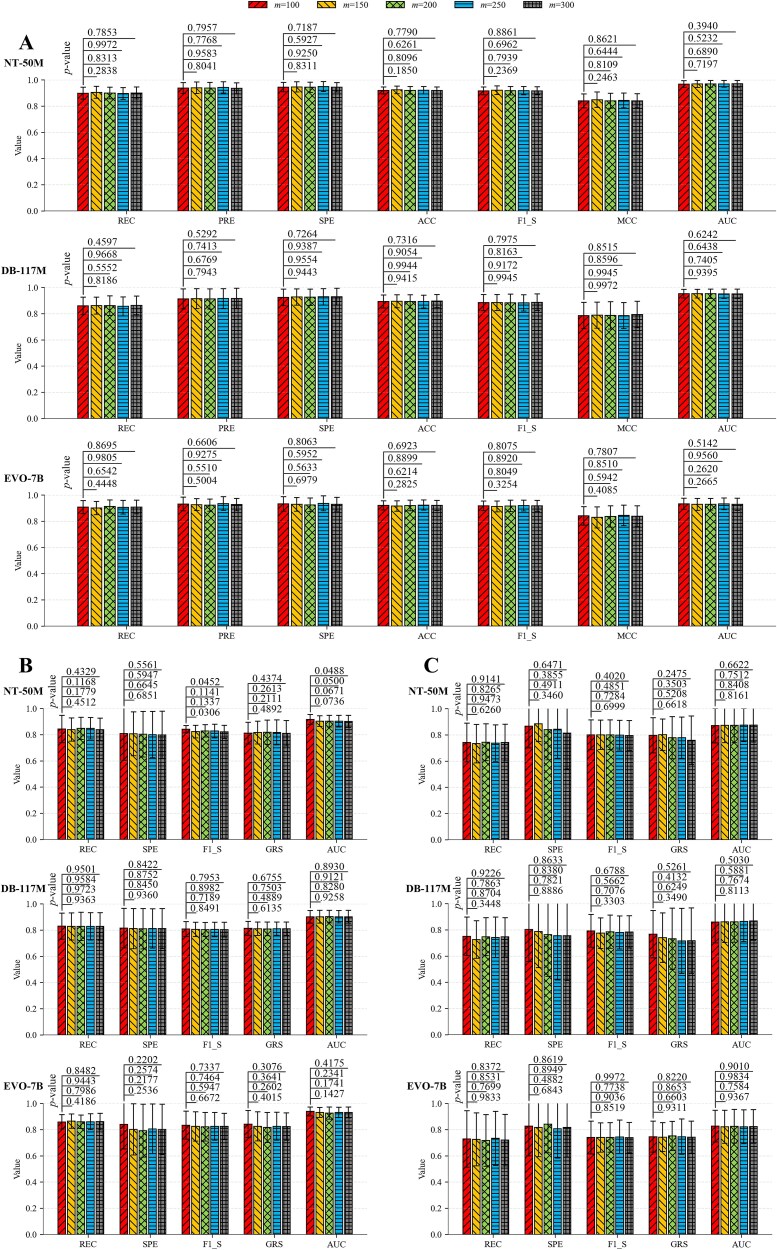
Effect of the number of selected segments (*m*) on the performance of the proposed method. The values above the bars are *p*-values from Mann–Whitney U tests comparing results at *m* = 100 versus other m-values, where *p* ≥ 0.05 indicates there is no significant difference between the results and *p* < 0.05 indicates there is a significant difference between the results. The error bars represent the standard deviation values across multiple independent experiments. NT-50M denotes that the foundation model representations are generated by Nucleotide Transformer-50M. DB-117M denotes that the foundation model representations are generated by DNABERT-2-117M. EVO-7B denotes that the foundation model representations are generated by EVO-7B. (A) The samples are divided using the completely random splitting scheme for cross-validation. (B) The samples are divided using the species-level phylogenetically informed splitting scheme for cross-validation. (C) The samples are divided using the hierarchical clustering splitting scheme for cross-validation. The positive and negative samples for validation in each fold are balanced for (A) and imbalanced for (B) and (C).

For each target sample, *m* segments are selected as input. If *m* is too small, the sample may be inadequately represented, whereas if *m* is too large, resource consumption increases. We examine how varying *m* affects the performance of the proposed method. The cross-attention mechanism is trained on the training subset 1 of Experimental Group 1, and its weights are then frozen to obtain the enhanced representations. We perform ​​five-fold cross-validation​​ on the cross-validation set (i.e. the training subset 2 of Experimental Group 1) using the stacked aggregation classifier. In each fold, 10 independent experiments are conducted, with each experiment selecting *m* segments that are not entirely identical to those in previous experiments, to obtain statistical results (Fig. 3).

When *m* takes different values, the proposed method shows minimal variations overall, regardless of which foundation model is used to generate the representations. Statistical tests reveal that, in most cases, there is no significant difference between the results at *m* = 100 and each of the other values of *m*, except for two cases of significant differences in F1_S and one in AUC. Therefore, we set the number of segments selected from a target sample to 100. This setting achieves a moderate reduction in computational resources usage and time while maintaining applicability. Even for an incompletely assembled sample sequence, prediction can be performed as long as 100 segments can be obtained.

### Effectiveness evaluation of enhanced representation and stacked aggregation classifier

**Figure 4:**
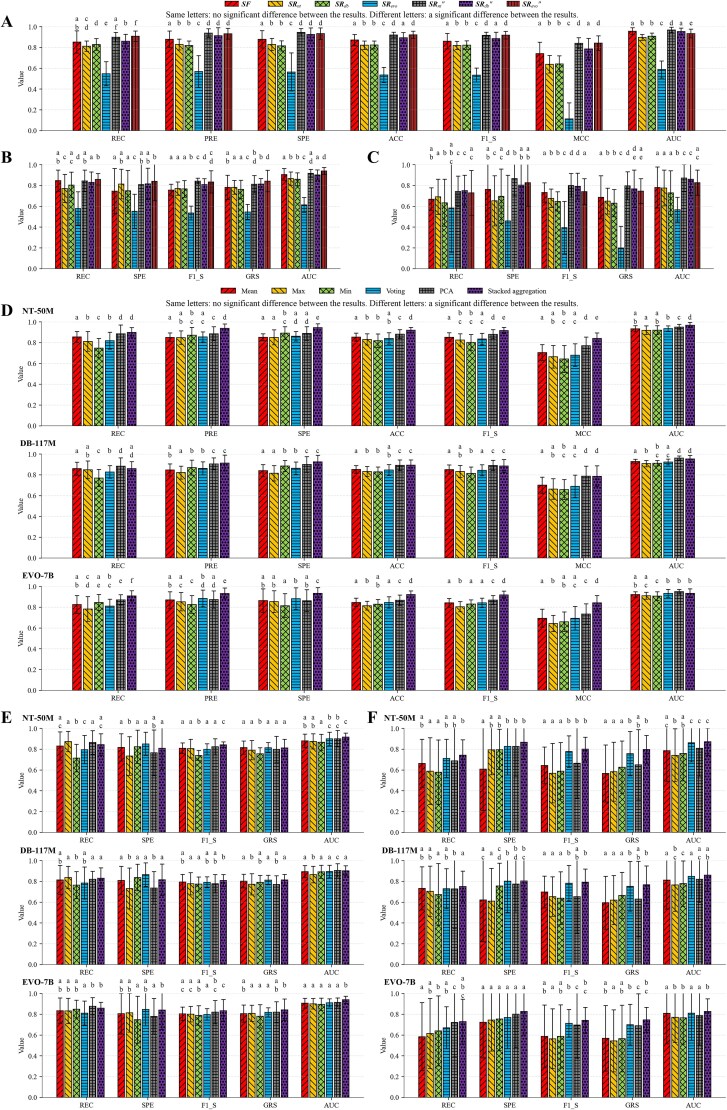
Effectiveness evaluation of enhanced representations and stacked aggregation classifier. The letters above the bars are results of Mann–Whitney U tests, where results labeled with the same letters indicate that there is no significant difference between them (*p* ≥ 0.05), and results labeled with different letters indicate that there is a significant difference between them (*p* < 0.05). The error bars represent the standard deviation values across multiple independent experiments. (A–C) We keep the architecture and training strategy of the stacked aggregation classifier unchanged while varying the features/representations as inputs, where (A) the samples are divided using the completely random splitting scheme for cross-validation, (B) the samples are divided using the species-level phylogenetically informed splitting scheme for cross-validation, and (C) the samples are divided using the hierarchical clustering splitting scheme for cross-validation. The positive and negative samples for validation in each fold are balanced for (A) and imbalanced for (B) and (C). ***SF*** denotes the engineered features. ***SR**_nt_*, ***SR****_db_*, and ***SR****_evo_* denote the foundation model representations generated by Nucleotide Transformer-50M, DNABERT-2- 117M and EVO-7B, respectively. ***SR****_nt_*", ***SR****_db_*", and ***SR****_evo_*" denote the enhanced representations, where the foundation model representations are generated by Nucleotide Transformer-50M, DNABERT-2-117M and EVO-7B, respectively. (D–F) We use the same enhanced representation matrices while employing different approaches for converting matrices to vectors as input, where (D) the samples are divided using the completely random splitting scheme for cross-validation, (E) the samples are divided using the species-level phylogenetically informed splitting scheme for cross-validation, and (F) the samples are divided using the hierarchical clustering splitting scheme for cross-validation. The positive and negative samples for validation in each fold are balanced for (D) and imbalanced for (E) and (F). NT-50M denotes that the foundation model representations are generated by Nucleotide Transformer-50M. DB-117M denotes that the foundation model representations generated by DNABERT-2-117M. EVO-7B denotes that the foundation model representations are generated by EVO-7B. Mean, max, min, and PCA denote the mean-taking approach, maximum-taking approach, minimum-taking approach, and PCA approach, respectively. Voting denotes the segment label voting approach. To ensure fairness, these approaches employ the LR model as the classifier.

The inputs to the stacked aggregation classifier can be enhanced representation vectors, engineered feature vectors, or initial foundation model representation vectors. For the enhanced representation matrix of a sample, in addition to using stacked aggregation to convert it into a vector as the classifier’s input, approaches such as taking the mean, maximum, or minimum along each dimension of the matrix, or applying PCA, can also be employed. Furthermore, a classifier can be trained using the vectors of segments, and then used to predict labels for each segment, with voting determining the label of the target sample.​ We use Experimental Group 1, performing five-fold cross-validation on the cross-validation set, to evaluate the effectiveness of ​​the enhanced representations​​ and the ​​stacked aggregation classifier (Fig. 4). To ensure a fair comparison, we modify only the specific component under evaluation while keeping the remaining components unchanged.

The classifiers trained with enhanced representations generally achieve significantly better results than those trained with the corresponding initial foundation model representations, with particularly substantial performance improvements observed when enhancing the representations from EVO-7B. Classifiers trained with engineered features exhibit strong competitiveness, often outperforming those trained with initial foundation model representations. However, they do not surpass classifiers trained with enhanced representations (in most cases, the latter’s results are either significantly better than or not significantly different from the former’s results). Among the three foundation models, classifiers trained with enhanced representations derived from Nucleotide Transformer-50M and EVO-7B outperform those derived from DNABERT-2-117M overall. The stacked aggregation classifier shows less pronounced improvements when using enhanced representations derived from DNABERT-2-117M, but when using those derived from Nucleotide Transformer-50M and EVO-7B, it generally achieves better results than classifiers based on other approaches. According to these results, we use classifiers trained with enhanced representations derived from Nucleotide Transformer-50M and EVO-7B for comparative experiments with other methods.

### Comparison with other methods on balanced and imbalanced test sets

**Figure 5:**
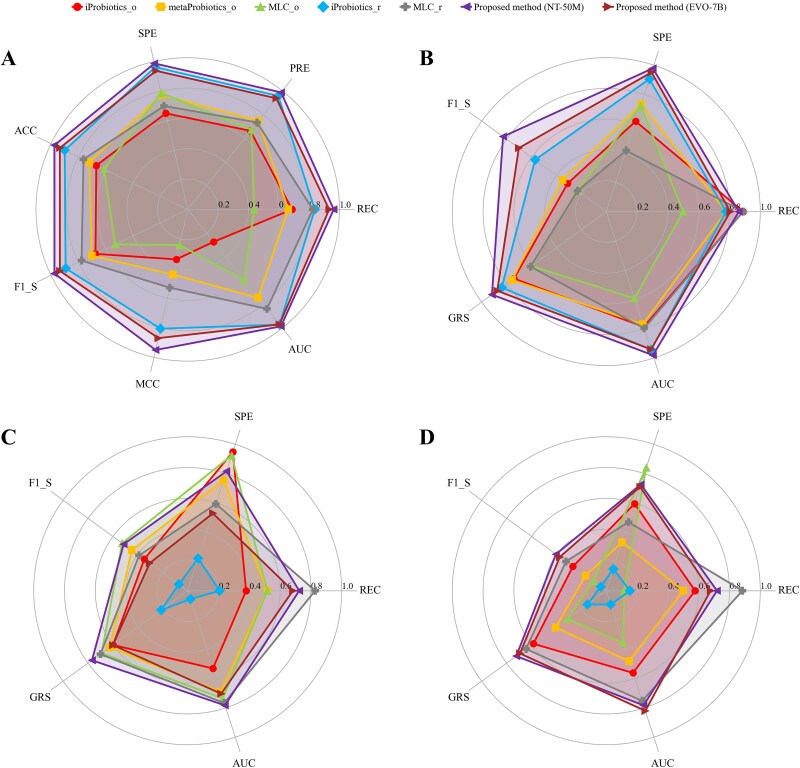
Comparisons of the proposed method with other methods. NT-50M and EVO-7B denote that, in the proposed method, the foundation model representations are generated by Nucleotide Transformer-50M and EVO-7B, respectively. (A) Results on the balanced test set of Experimental Group 1. (B) Results on the imbalanced test set of Experimental Group 1. (C) Results on the imbalanced test set of Experimental Group 2. (D) Results on the imbalanced test set of Experimental Group 3.

We use three experimental groups to compare the proposed method with iProbiotics [18], metaProbiotics [19], and MLC [48]. iProbiotics and metaProbiotics have been introduced previously, while MLC uses the GBRAP tool to extract features from bacterial and archaeal data to train a classifier. iProbiotics_o, metaProbiotics_o, and MLC_o denote the original methods without modification, while iProbiotics_r and MLC_r retain their original frameworks but retrain the classifier on the same training data as the proposed method (i.e. soil microbial data). Since the proposed method only requires randomly selecting 100 segments per test sample for prediction, we conduct 10 independent experiments to obtain the mean for each criterion (Fig. 5). In each independent experiment, a segment group not entirely consistent with previous ones is selected for each test sample.

On the balanced test set of Experimental Group 1, the proposed method (NT-50M) achieves the best results across all criteria, while the proposed method (EVO-7B) produces the second-best results on four criteria. iProbiotics_r shows strong competitiveness, particularly by achieving high PRE and SPE values. On the imbalanced test set of Experimental Group 1, iProbiotics_o and MLC_r perform better on REC, but their results for other criteria are poorer. iProbiotics_r remains the most competitive comparison method, with its AUC being comparable to that of the proposed method (EVO-7B) and second only to the proposed method (NT-50M). Considering all these results, in Experimental Group 1, the proposed method (NT-50M) delivers the best performance, followed by the proposed method (EVO-7B).

On the imbalanced test set of Experimental Group 2, since the training and test samples are divided based on the phylogenetically informed splitting scheme, all methods show a noticeable drop in performance compared with their results in Experimental Group 1 (where the training and test samples are divided completely at random). iProbiotics_r, which is highly competitive on the test sets of Experimental Group 1, produces the poorest results here. This may be because it relies heavily on characteristics specific to the training data, and when the similarity between the training and test data is reduced, the model struggles to accurately identify the characteristics of the test samples. MLC_r achieves the highest REC value, but its SPE value is low, as it predicts a large number of negative samples as positive. iProbiotics_o and MLC_o achieve high SPE values, but their REC values are low, indicating that they predict many positive samples as negative. The proposed method (EVO-7B) also yields unsatisfactory results, with an F1_S slightly above 0.3, a GRS slightly above 0.6, and an AUC slightly above 0.7. Encouragingly, the proposed method (NT-50M) performs well. Its F1_S is second only to that of MLC_o and significantly higher than those of other methods. It also achieves a GRS above 0.76 and an AUC above 0.78, both the highest among all methods.

On the imbalanced test set of Experimental Group 3, where the training and test samples are divided based on the hierarchical clustering splitting scheme, all methods also show reduced performance relative to Experimental Group 1. iProbiotics_r, as in Experimental Group 2, remains the weakest performer. MLC_r obtains higher REC but lower SPE, whereas MLC_o obtains higher SPE but lower REC. The proposed method (EVO-7B) achieves the highest AUC and the second-highest F1_S and GRS. The proposed method (NT-50M) achieves the highest F1_S and GRS and the second-highest AUC.

Overall, the proposed method (NT-50M) achieves the best performance on balanced and imbalanced test sets under the three sample splitting schemes. Moreover, its foundation model is lightweight, with only 50M parameters, thereby reducing the demand for computational resources and runtime, making it the most practical method for soil probiotic prediction.

### Applicability to samples with complete and incomplete genomic sequences

**Figure 6:**
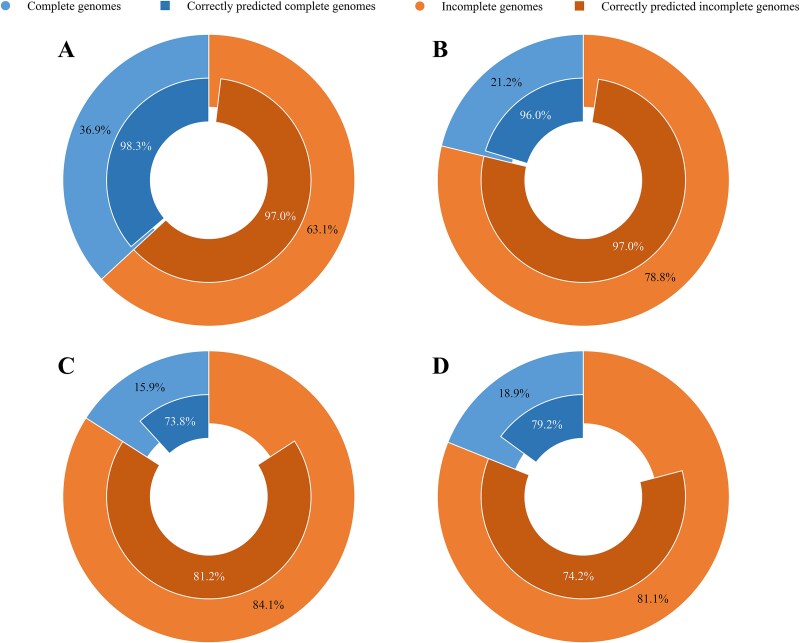
Applicability of the proposed method to samples with complete and incomplete genomic sequences. Proportions of samples with complete and incomplete genomic sequences and corresponding prediction results on (A) the balanced test set of Experimental Group 1, (B) the imbalanced test set of Experimental Group 1, (C) the imbalanced test set of Experimental Group 2, and (D) the imbalanced test set of Experimental Group 3.

Incomplete genomes, such as draft genomes, are commonly encountered due to challenges like low sequencing depth, high microbial diversity, and limitations in assembly tools. Shallow sequencing often results in incomplete genomic coverage, leading to missing or ambiguous data that constrains data-driven models in soil microbiome research. To evaluate the applicability of the proposed method (NT-50M) under these conditions, we compile the proportions of samples with complete and incomplete genome sequences in the test sets from three experimental groups, along with the corresponding average prediction results from 10 independent experiments (Fig. 6).

In the balanced test set of Experimental Group 1, samples with complete genome sequences account for 36.9%, and the proposed method correctly predicts 98.3% of them. Samples with incomplete genome sequences account for 63.1%, among which 97.0% are accurately predicted by the proposed method. In the imbalanced test set of Experimental Group 1, the proportions of samples with complete and incomplete genome sequences are 21.2% and 78.8%, respectively, and the proposed method achieves accuracies of 96.0% and 97.0% for these two categories. For the imbalanced test set of Experimental Group 2, samples with incomplete genome sequences still occupy a larger proportion (84.1%). Although the samples in the test set have relatively low similarity to those in the training set, the proposed method correctly predicts 73.8% of the samples with complete genome sequences and 81.2% of the samples with incomplete genome sequences. For the imbalanced test set of Experimental Group 3, the proportion of samples with incomplete genome sequences is 81.1%, and the proposed method achieves accuracies of 79.2% and 74.2% for samples with complete and incomplete genome sequences, respectively. Since the proposed method only requires randomly selecting a subset of sequence segments for prediction, its performance is independent of the genomic completeness of the target sample. This applicability makes the proposed method reliable in real-world applications where incomplete genomes frequently occur.

### Exploration of potential functional genes

We compile the labels and confidence scores predicted by the proposed method (NT-50M) for all test samples, and select one representative sample (GCA_000242855.2, GCA_015710975.1, and GCA_000385945.1) from each experimental group for gene function annotation and enrichment analysis. These samples are consistently predicted as probiotics across all independent experiments and rank within the top 10 in terms of average confidence scores. We use Prodigal v2.6.3 [44], a fast and accurate open-source tool for prokaryotic gene identification and commonly used to obtain protein-coding genes in both complete and draft microbial genomes, to obtain the gene sequences of these samples.

These bacterial species are reported to possess phosphate-solubilizing activity [49–51]. We obtain 2532, 2914, and 3117 effective annotations for GCA_000242855.2, GCA_015710975.1, and GCA_000385945.1, respectively. The annotations of each sample cover more than 2300 genes and more than 1900 KO identifiers (Supplementary Tables S5–S7). Functional mapping assigns KO identifiers potentially associated with phosphate solubilization, such as K01113, K01077, and K01083, to several genes in each of these samples (Table 2). Enrichment analysis further suggests that these genes may participate in phosphate-solubilization-related pathways, such as map01100 (Metabolic pathways) and map02020 (Two-component system). These findings reveal genes in these bacteria that may play key roles in phosphate-solubilizing capacity.

**Table 2:** Gene function annotations that the KO identifiers are potentially associated with phosphate solubilization.

**Sample**	**Gene ID**	**KO identifier**	**Threshold**	**Score**	***e*-value**
GCA_000242855.2	CP004065.1_3552	K01113	89.83	541.6	6.9e-163
	CP004065.1_2927	K01077	73.07	361.6	1.7e-108
	CP004065.1_1845	K01083	115.73	463.3	2.7e-139
	CP004065.1_1575	K01126	152.53	224.4	4.1e-67
	CP004065.1_1026	K01126	152.53	208.6	2.5e-62
	CP004065.1_2909	K01126	152.53	231.7	2.4e-69
	CP004065.1_3584	K01126	152.53	265.4	1.3e-79
	CP004065.1_55	K22927	277.33	882.3	4.4e-266
GCA_015710975.1	CP049783.1_283	K01077	73.07	338.0	3.4e-101
	CP049783.1_3855	K01077	73.07	327.9	3.8e-98
	CP049783.1_1472	K01083	115.73	479.4	5.3e-144
	CP049783.1_1370	K01126	152.53	249.4	1.4e-74
	CP049783.1_5272	K01126	152.53	217.2	8.7e-65
	CP049783.1_1704	K22927	277.33	844.6	1.7e-254
GCA_000385945.1	CP005080.1_820	K01113	89.83	277.8	8.4e-83
	CP005080.1_1619	K01113	89.83	508.3	1.4e-152
	CP005080.1_1841	K01113	89.83	491.5	1.7e-147
	CP005080.1_1905	K01113	89.83	128.3	1.5e-37
	CP005080.1_216	K01083	115.73	423.9	4.3e-127
	CP005080.1_618	K01126	152.53	171.5	9.1e-51
	CP005080.1_932	K01126	152.53	217.6	8.2e-65
	CP005080.1_1066	K01126	152.53	233.0	1.8e-69
	CP005080.1_1503	K01126	152.53	222.5	2.7e-66
	CP005080.1_2173	K01126	152.53	214.5	7.3e-64
	CP005080.1_3588	K01126	152.53	242.1	3.1e-72
	CP005080.1_5498	K01126	152.53	154.0	1.8e-45

## Discussion

Soil probiotics play indispensable roles in regulating agro-ecosystem functions, and reliable prediction of probiotics is crucial for discovering and cultivating novel functional strains. While current prediction methods predominantly focus on microorganisms in environments such as the human gut, specialized methods for soil probiotics remain conspicuously absent. This study presents a method based on foundation model representation enhancement and a stacked aggregation classifier to address this critical gap.

Classifiers trained with engineered features perform better than those trained solely with foundation model representations, potentially due to​​ their suitability for low-complexity classification tasks. It is encouraging that the classifiers trained using enhanced representations achieve further breakthroughs. The stacked aggregation classifier employs a structure of two submodels to aggregate the enhanced representation vectors of multiple sequence segments into a single vector, improving prediction performance compared to conventional approaches. The first-level model (XGBoost) in the stacked aggregation classifier assigns a score to each input sequence segment. In addition to serving as the input to the second-level model (LR), this score essentially reflects the likelihood that the segment originates from a probiotic strain and may correlate with its frequency of occurrence in probiotics. Since each segment corresponds to a 1000 bp genomic sequence, a high-scoring segment may contain important functional elements, such as complete or partial genes and regulatory regions, that influence probiotic characteristics. This enhances the interpretability of the model and enables tracing of potential functional components based on individual segment scores.

The results of iProbiotics_o, metaProbiotics_o, and MLC_o verify that models trained on nonsoil microbial data are unreliable for predicting soil probiotics. The results of iProbiotics_r and MLC_r further verify that, even when these models are retrained using soil microbial data, their effectiveness is limited for soil probiotic prediction, as their original designs are not tailored for soil probiotic prediction but instead optimized for other specific tasks. ​​The proposed method uses randomly selected sequence segments as input and does not impose strict requirements on the target samples’ genome completeness. As a result, it demonstrates strong performance on both complete and incomplete genomes (e.g. draft genomes), thereby supporting its robustness and applicability in real-world scenarios. Gene function annotation and enrichment analysis for the predicted probiotics further support the utility of the proposed method for biological discovery. The proposed method delivers robust performance for soil probiotic prediction. Its trained cross-attention module enables the integration of heterogeneous representations in related studies, and its stacked aggregation approach provides valuable references for long-sequence processing.

The proposed method consists of foundation model representation generation, engineered feature extraction, representation enhancement, and a stacked aggregation classifier. Within this framework, any module can be flexibly replaced or improved, providing potential for scalability. The modular design allows the application of model compression techniques (e.g. knowledge distillation) to reduce the model size or replace the classifier with simpler alternatives. In addition, since the representation generation and enhancement modules are separated from the classification module, representations that have been generated or enhanced can be reused without repeated generation or enhancement, thereby further reducing computational costs during inference. These designs facilitate the lightweight deployment of the proposed method.

While the proposed method demonstrates strong performance, the use of fixed-length sequence segments may introduce biological limitations by disrupting functional genomic elements such as operons, coding DNA sequences, and regulatory regions. In future work, we plan to replace fixed-length sequence segments with open reading frame sequences, which will directly associate the prediction results with specific genes to enable gene-level interpretation, thereby further improving the interpretability of the model. We will extend the key modules of the proposed method to studies of other environmental microbiomes, including applying the cross-attention mechanism to deeply integrate heterogeneous or multimodal information and adopting the stacked aggregation approach to process long or incompletely assembled genomic sequences. Leveraging the framework of the proposed method, we will also conduct high-accuracy predictions for specific probiotic species, deeply investigate functional genes, and aim to derive novel biological insights through wet-lab experiments.

**Key Points**

- Soil probiotics are predicted based on foundation model representation enhancement and stacked aggregation classifier.
- The foundation model representations are enhanced by deeply integrating domain-specific engineered features, enabling the training of a powerful classifier for a target task.
- A stacked aggregation classifier is designed that leverages a subset of sequence segments from a sample to predict its label, effectively processing long or incompletely assembled sequences.
- The proposed method exhibits better predictive performance than other methods and provides strong support for biological discoveries.

## Supplementary Material

Supplementary_Table_S1_R2_bbaf567

Supplementary_Table_S2_R2_bbaf567

Supplementary_Table_S3_R2_bbaf567

Supplementary_Table_S4_R2_bbaf567

Supplementary_Table_S5_R2_bbaf567

Supplementary_Table_S6_R2_bbaf567

Supplementary_Table_S7_R2_bbaf567

## Data Availability

All data used in this study is publicly available and can be downloaded from NCBI (https://www.ncbi.nlm.nih.gov/). The packaged dataset can also be downloaded from Zenodo (https://zenodo.org/records/17067025). The source codes of the proposed method are available at https://github.com/sunhaotong0605/SPP_FMRESAC.
