# Supplementary material for: Prediction of soil probiotics based on foundation model representation enhancement and stacked aggregation classifier
Source: Brief Bioinform. 2025 Oct 29;26(5):bbaf567. doi: 10.1093/bib/bbaf567 (PMC12570017; doi:10.1093/bib/bbaf567)
Supplement: Supplementary_Table_S1_R2_bbaf567 [file supplementary_table_s1_r2_bbaf567.pdf]

Supplementary Table S1. Details of samples.

GCA	Phylum	Class	Order	Family	Genus	Species	P(positive) N(negative)	Link
GCA_000242855.2	Bacillota	Bacilli	Bacillales	Bacillaceae	Bacillus	Bacillus amyloliquefaciens	P	https://www.ncbi.nlm.nih.gov/datasets/genome/GCA_000242855.2/
GCA_015880875.1	Bacillota	Bacilli	Bacillales	Bacillaceae	Bacillus	Bacillus amyloliquefaciens	P	https://www.ncbi.nlm.nih.gov/datasets/genome/GCA_015880875.1/
GCA_026790565.1	Bacillota	Bacilli	Bacillales	Bacillaceae	Bacillus	Bacillus subtilis	P	https://www.ncbi.nlm.nih.gov/datasets/genome/GCA_026790565.1/
GCA_026792875.1	Bacillota	Bacilli	Bacillales	Bacillaceae	Bacillus	Bacillus subtilis	P	https://www.ncbi.nlm.nih.gov/datasets/genome/GCA_026792875.1/
GCA_000699525.1	Bacillota	Bacilli	Bacillales	Bacillaceae	Bacillus	Bacillus subtilis	P	https://www.ncbi.nlm.nih.gov/datasets/genome/GCA_000699525.1/
GCA_019931715.1	Bacillota	Bacilli	Bacillales	Bacillaceae	Bacillus	Bacillus subtilis	P	https://www.ncbi.nlm.nih.gov/datasets/genome/GCA_019931715.1/
GCA_921008455.1	Bacillota	Bacilli	Bacillales	Bacillaceae	Bacillus	Bacillus sp. (in: firmicutes)	P	https://www.ncbi.nlm.nih.gov/datasets/genome/GCA_921008455.1/
GCA_001375535.1	Bacillota	Bacilli	Bacillales	Bacillaceae	Bacillus	Bacillus niameyensis	P	https://www.ncbi.nlm.nih.gov/datasets/genome/GCA_001375535.1/
GCA_002024555.1	Bacillota	Bacilli	Bacillales	Bacillaceae	Bacillus	Bacillus amyloliquefaciens	P	https://www.ncbi.nlm.nih.gov/datasets/genome/GCA_002024555.1/
GCA_009665145.2	Bacillota	Bacilli	Bacillales	Bacillaceae	Bacillus	Bacillus subtilis	P	https://www.ncbi.nlm.nih.gov/datasets/genome/GCA_009665145.2/
GCA_026123105.1	Bacillota	Bacilli	Bacillales	Bacillaceae	Bacillus	Bacillus amyloliquefaciens	P	https://www.ncbi.nlm.nih.gov/datasets/genome/GCA_026123105.1/
GCA_019599105.1	Bacillota	Bacilli	Bacillales	Bacillaceae	Bacillus	Bacillus subtilis	P	https://www.ncbi.nlm.nih.gov/datasets/genome/GCA_019599105.1/
GCA_001565875.1	Bacillota	Bacilli	Bacillales	Bacillaceae	Bacillus	Bacillus subtilis	P	https://www.ncbi.nlm.nih.gov/datasets/genome/GCA_001565875.1/
GCA_021655335.1	Bacillota	Bacilli	Bacillales	Bacillaceae	Bacillus	Bacillus cereus	P	https://www.ncbi.nlm.nih.gov/datasets/genome/GCA_021655335.1/
GCA_001286965.1	Bacillota	Bacilli	Bacillales	Bacillaceae	Bacillus	Bacillus amyloliquefaciens	P	https://www.ncbi.nlm.nih.gov/datasets/genome/GCA_001286965.1/
GCA_030063805.1	Bacillota	Bacilli	Bacillales	Bacillaceae	Bacillus	Bacillus amyloliquefaciens	P	https://www.ncbi.nlm.nih.gov/datasets/genome/GCA_030063805.1/
GCA_022453545.1	Bacillota	Bacilli	Bacillales	Bacillaceae	Bacillus	Bacillus subtilis	P	https://www.ncbi.nlm.nih.gov/datasets/genome/GCA_022453545.1/
GCA_023521615.1	Bacillota	Bacilli	Bacillales	Bacillaceae	Bacillus	Bacillus subtilis	P	https://www.ncbi.nlm.nih.gov/datasets/genome/GCA_023521615.1/
GCA_023521595.1	Bacillota	Bacilli	Bacillales	Bacillaceae	Bacillus	Bacillus subtilis	P	https://www.ncbi.nlm.nih.gov/datasets/genome/GCA_023521595.1/
GCA_004119535.1	Bacillota	Bacilli	Bacillales	Bacillaceae	Bacillus	Bacillus subtilis	P	https://www.ncbi.nlm.nih.gov/datasets/genome/GCA_004119535.1/
GCA_905315685.2	Bacillota	Bacilli	Bacillales	Bacillaceae	Bacillus	Bacillus subtilis	P	https://www.ncbi.nlm.nih.gov/datasets/genome/GCA_905315685.2/
GCA_001889285.1	Bacillota	Bacilli	Bacillales	Bacillaceae	Bacillus	Bacillus amyloliquefaciens	P	https://www.ncbi.nlm.nih.gov/datasets/genome/GCA_001889285.1/
GCA_000747705.1	Bacillota	Bacilli	Bacillales	Bacillaceae	Bacillus	Bacillus amyloliquefaciens	P	https://www.ncbi.nlm.nih.gov/datasets/genome/GCA_000747705.1/
GCA_021172185.1	Bacillota	Bacilli	Bacillales	Bacillaceae	Bacillus	Bacillus subtilis	P	https://www.ncbi.nlm.nih.gov/datasets/genome/GCA_021172185.1/
GCA_001617995.1	Bacillota	Bacilli	Bacillales	Bacillaceae	Bacillus	Bacillus amyloliquefaciens	P	https://www.ncbi.nlm.nih.gov/datasets/genome/GCA_001617995.1/
GCA_001593785.1	Bacillota	Bacilli	Bacillales	Bacillaceae	Bacillus	Bacillus amyloliquefaciens	P	https://www.ncbi.nlm.nih.gov/datasets/genome/GCA_001593785.1/
GCA_024220155.1	Bacillota	Bacilli	Bacillales	Bacillaceae	Bacillus	Bacillus subtilis	P	https://www.ncbi.nlm.nih.gov/datasets/genome/GCA_024220155.1/
GCA_900156245.1	Bacillota	Bacilli	Bacillales	Bacillaceae	Bacillus	Bacillus amyloliquefaciens	P	https://www.ncbi.nlm.nih.gov/datasets/genome/GCA_900156245.1/
GCA_029371985.1	Bacillota	Bacilli	Bacillales	Bacillaceae	Bacillus	Bacillus amyloliquefaciens	P	https://www.ncbi.nlm.nih.gov/datasets/genome/GCA_029371985.1/
GCA_013346225.1	Bacillota	Bacilli	Bacillales	Bacillaceae	Bacillus	Bacillus amyloliquefaciens	P	https://www.ncbi.nlm.nih.gov/datasets/genome/GCA_013346225.1/
GCA_000196735.1	Bacillota	Bacilli	Bacillales	Bacillaceae	Bacillus	Bacillus amyloliquefaciens	P	https://www.ncbi.nlm.nih.gov/datasets/genome/GCA_000196735.1/
GCA_000982455.1	Bacillota	Bacilli	Bacillales	Bacillaceae	Bacillus	Bacillus subtilis	P	https://www.ncbi.nlm.nih.gov/datasets/genome/GCA_000982455.1/
GCA_001596755.1	Bacillota	Bacilli	Bacillales	Bacillaceae	Bacillus	Bacillus amyloliquefaciens	P	https://www.ncbi.nlm.nih.gov/datasets/genome/GCA_001596755.1/
GCA_008808015.1	Bacillota	Bacilli	Bacillales	Bacillaceae	Bacillus	Bacillus amyloliquefaciens	P	https://www.ncbi.nlm.nih.gov/datasets/genome/GCA_008808015.1/
GCA_009497795.1	Bacillota	Bacilli	Bacillales	Bacillaceae	Bacillus	Bacillus subtilis	P	https://www.ncbi.nlm.nih.gov/datasets/genome/GCA_009497795.1/
GCA_905319555.1	Bacillota	Bacilli	Bacillales	Bacillaceae	Bacillus	Bacillus subtilis	P	https://www.ncbi.nlm.nih.gov/datasets/genome/GCA_905319555.1/
GCA_014792065.1	Bacillota	Bacilli	Bacillales	Bacillaceae	Bacillus	Bacillus amyloliquefaciens	P	https://www.ncbi.nlm.nih.gov/datasets/genome/GCA_014792065.1/
GCA_027497435.1	Bacillota	Bacilli	Bacillales	Bacillaceae	Bacillus	Bacillus subtilis	P	https://www.ncbi.nlm.nih.gov/datasets/genome/GCA_027497435.1/
GCA_004119675.1	Bacillota	Bacilli	Bacillales	Bacillaceae	Bacillus	Bacillus subtilis	P	https://www.ncbi.nlm.nih.gov/datasets/genome/GCA_004119675.1/
GCA_017948535.1	Bacillota	Bacilli	Bacillales	Bacillaceae	Bacillus	Bacillus amyloliquefaciens	P	https://www.ncbi.nlm.nih.gov/datasets/genome/GCA_017948535.1/
GCA_026788685.1	Bacillota	Bacilli	Bacillales	Bacillaceae	Bacillus	Bacillus subtilis	P	https://www.ncbi.nlm.nih.gov/datasets/genome/GCA_026788685.1/
GCA_016065415.1	Bacillota	Bacilli	Bacillales	Bacillaceae	Bacillus	Bacillus subtilis	P	https://www.ncbi.nlm.nih.gov/datasets/genome/GCA_016065415.1/
GCA_003426125.1	Bacillota	Bacilli	Bacillales	Bacillaceae	Bacillus	Bacillus subtilis	P	https://www.ncbi.nlm.nih.gov/datasets/genome/GCA_003426125.1/
GCA_003665255.1	Bacillota	Bacilli	Bacillales	Bacillaceae	Bacillus	Bacillus subtilis	P	https://www.ncbi.nlm.nih.gov/datasets/genome/GCA_003665255.1/
GCA_032930055.1	Bacillota	Bacilli	Bacillales	Bacillaceae	Bacillus	Bacillus amyloliquefaciens	P	https://www.ncbi.nlm.nih.gov/datasets/genome/GCA_032930055.1/
GCA_008534375.1	Bacillota	Bacilli	Bacillales	Bacillaceae	Bacillus	Bacillus amyloliquefaciens	P	https://www.ncbi.nlm.nih.gov/datasets/genome/GCA_008534375.1/
GCA_900104735.1	Pseudomonadota	Gammaproteobacteria	Pseudomonadales	Pseudomonadaceae	Pseudomonas	Pseudomonas lini	P	https://www.ncbi.nlm.nih.gov/datasets/genome/GCA_900104735.1/
GCA_900104365.1	Pseudomonadota	Gammaproteobacteria	Pseudomonadales	Pseudomonadaceae	Pseudomonas	Pseudomonas extremorientalis	P	https://www.ncbi.nlm.nih.gov/datasets/genome/GCA_900104365.1/
GCA_900101415.1	Pseudomonadota	Gammaproteobacteria	Pseudomonadales	Pseudomonadaceae	Pseudomonas	Pseudomonas koreensis	P	https://www.ncbi.nlm.nih.gov/datasets/genome/GCA_900101415.1/
GCA_900101185.1	Pseudomonadota	Gammaproteobacteria	Pseudomonadales	Pseudomonadaceae	Pseudomonas	Pseudomonas gessardii	P	https://www.ncbi.nlm.nih.gov/datasets/genome/GCA_900101185.1/
GCA_033842905.1	Pseudomonadota	Gammaproteobacteria	Pseudomonadales	Pseudomonadaceae	Ectopseudomonas	Ectopseudomonas alcaliphila	P	https://www.ncbi.nlm.nih.gov/datasets/genome/GCA_033842905.1/
GCA_031985445.1	Pseudomonadota	Betaproteobacteria	Burkholderiales	Alcaligenaceae	Alcaligenes	Alcaligenes sp.	P	https://www.ncbi.nlm.nih.gov/datasets/genome/GCA_031985445.1/
GCA_031455735.1	Pseudomonadota	Gammaproteobacteria	Pseudomonadales	Pseudomonadaceae	Pseudomonas	Pseudomonas hunanensis	P	https://www.ncbi.nlm.nih.gov/datasets/genome/GCA_031455735.1/

GCA_031180825.1	Bacillota	Bacilli	Bacillales	Paenibacillaceae	Paenibacillus	Paenibacillus sp.	P	https://www.ncbi.nlm.nih.gov/datasets/genome/GCA_031180825.1/
GCA_029866525.1	Pseudomonadota	Gammaproteobacteria	Pseudomonadales	Pseudomonadaceae	Pseudomonas	Pseudomonas migulae	P	https://www.ncbi.nlm.nih.gov/datasets/genome/GCA_029866525.1/
GCA_029457395.1	Pseudomonadota	Gammaproteobacteria	Pseudomonadales	Pseudomonadaceae	Pseudomonas	Pseudomonas nitroreducens	P	https://www.ncbi.nlm.nih.gov/datasets/genome/GCA_029457395.1/
GCA_024723775.1	Bacillota	Bacilli	Bacillales	Paenibacillaceae	Paenibacillus	Paenibacillus peoriae	P	https://www.ncbi.nlm.nih.gov/datasets/genome/GCA_024723775.1/
GCA_022647425.1	Bacillota	Bacilli	Bacillales	Paenibacillaceae	Aneurinibacillus	Aneurinibacillus aneurinilyticus	P	https://www.ncbi.nlm.nih.gov/datasets/genome/GCA_022647425.1/
GCA_016463995.1	Pseudomonadota	Gammaproteobacteria	Pseudomonadales	Pseudomonadaceae	Pseudomonas	Pseudomonas sp.	P	https://www.ncbi.nlm.nih.gov/datasets/genome/GCA_016463995.1/
GCA_016027415.1	Actinomycetota	Actinomycetes	Micrococcales	Brevibacteriaceae	Brevibacterium	Brevibacterium casei	P	https://www.ncbi.nlm.nih.gov/datasets/genome/GCA_016027415.1/
GCA_014524625.1	Pseudomonadota	Gammaproteobacteria	Pseudomonadales	Pseudomonadaceae	Pseudomonas	Pseudomonas chlororaphis	P	https://www.ncbi.nlm.nih.gov/datasets/genome/GCA_014524625.1/
GCA_014490035.1	Pseudomonadota	Betaproteobacteria	Burkholderiales	Alcaligenaceae	Achromobacter	Achromobacter xylosoxidans	P	https://www.ncbi.nlm.nih.gov/datasets/genome/GCA_014490035.1/
GCA_013752735.1	Pseudomonadota	Alphaproteobacteria	Hyphomicrobiales	Nitrobacteraceae	Bradyrhizobium	Bradyrhizobium japonicum	P	https://www.ncbi.nlm.nih.gov/datasets/genome/GCA_013752735.1/
GCA_013387025.1	Pseudomonadota	Gammaproteobacteria	Pseudomonadales	Pseudomonadaceae	Pseudomonas	Pseudomonas reactans	P	https://www.ncbi.nlm.nih.gov/datasets/genome/GCA_013387025.1/
GCA_013285305.1	Pseudomonadota	Gammaproteobacteria	Pseudomonadales	Pseudomonadaceae	Pseudomonas	Pseudomonas rhodesiae	P	https://www.ncbi.nlm.nih.gov/datasets/genome/GCA_013285305.1/
GCA_009664975.1	Bacillota	Bacilli	Bacillales	Paenibacillaceae	Paenibacillus	Paenibacillus monticola	P	https://www.ncbi.nlm.nih.gov/datasets/genome/GCA_009664975.1/
GCA_008831505.1	Bacteroidota	Flavobacteriia	Flavobacteriales	Weeksellaceae	Chryseobacterium	Chryseobacterium sp.	P	https://www.ncbi.nlm.nih.gov/datasets/genome/GCA_008831505.1/
GCA_008274965.1	Pseudomonadota	Alphaproteobacteria	Rhodospirillales	Azospirillaceae	Azospirillum	Azospirillum brasilense	P	https://www.ncbi.nlm.nih.gov/datasets/genome/GCA_008274965.1/
GCA_007827425.1	Pseudomonadota	Alphaproteobacteria	Rhodospirillales	Azospirillaceae	Azospirillum	Azospirillum brasilense	P	https://www.ncbi.nlm.nih.gov/datasets/genome/GCA_007827425.1/
GCA_004723625.1	Pseudomonadota	Betaproteobacteria	Burkholderiales	Burkholderiaceae	Burkholderia	Burkholderia contaminans	P	https://www.ncbi.nlm.nih.gov/datasets/genome/GCA_004723625.1/
GCA_004306555.1	Pseudomonadota	Alphaproteobacteria	Hyphomicrobiales	Rhizobiaceae	Rhizobium	Rhizobium leguminosarum	P	https://www.ncbi.nlm.nih.gov/datasets/genome/GCA_004306555.1/
GCA_004000925.1	Bacillota	Bacilli	Bacillales	Paenibacillaceae	Paenibacillus	Paenibacillus illinoisensis	P	https://www.ncbi.nlm.nih.gov/datasets/genome/GCA_004000925.1/
GCA_003729985.1	Bacteroidota	Flavobacteriia	Flavobacteriales	Weeksellaceae	Chryseobacterium	Chryseobacterium cucumeris	P	https://www.ncbi.nlm.nih.gov/datasets/genome/GCA_003729985.1/
GCA_002813455.1	Pseudomonadota	Gammaproteobacteria	Pseudomonadales	Pseudomonadaceae	Pseudomonas	Pseudomonas baetica	P	https://www.ncbi.nlm.nih.gov/datasets/genome/GCA_002813455.1/
GCA_002736065.1	Pseudomonadota	Gammaproteobacteria	Pseudomonadales	Pseudomonadaceae	Pseudomonas	Pseudomonas mosselii	P	https://www.ncbi.nlm.nih.gov/datasets/genome/GCA_002736065.1/
GCA_002220155.1	Pseudomonadota	Gammaproteobacteria	Pseudomonadales	Pseudomonadaceae	Azotobacter	Azotobacter chroococcum	P	https://www.ncbi.nlm.nih.gov/datasets/genome/GCA_002220155.1/
GCA_002072065.1	Bacillota	Bacilli	Bacillales	Bacillaceae	Geobacillus	Geobacillus thermodenitrificans	P	https://www.ncbi.nlm.nih.gov/datasets/genome/GCA_002072065.1/
GCA_001633025.1	Bacillota	Bacilli	Bacillales	Paenibacillaceae	Paenibacillus	Paenibacillus glucanolyticus	P	https://www.ncbi.nlm.nih.gov/datasets/genome/GCA_001633025.1/
GCA_001586155.1	Pseudomonadota	Gammaproteobacteria	Pseudomonadales	Pseudomonadaceae	Pseudomonas	Pseudomonas citronellolis	P	https://www.ncbi.nlm.nih.gov/datasets/genome/GCA_001586155.1/
GCA_001439685.1	Pseudomonadota	Gammaproteobacteria	Pseudomonadales	Pseudomonadaceae	Pseudomonas	Pseudomonas libanensis	P	https://www.ncbi.nlm.nih.gov/datasets/genome/GCA_001439685.1/
GCA_000733715.2	Pseudomonadota	Gammaproteobacteria	Pseudomonadales	Pseudomonadaceae	Ectopseudomonas	Ectopseudomonas mendocina	P	https://www.ncbi.nlm.nih.gov/datasets/genome/GCA_000733715.2/
GCA_000262695.1	Pseudomonadota	Betaproteobacteria	Burkholderiales	Burkholderiaceae	Burkholderia	Burkholderia sp.	P	https://www.ncbi.nlm.nih.gov/datasets/genome/GCA_000262695.1/
GCA_000021045.1	Pseudomonadota	Gammaproteobacteria	Pseudomonadales	Pseudomonadaceae	Azotobacter	Azotobacter vinelandii	P	https://www.ncbi.nlm.nih.gov/datasets/genome/GCA_000021045.1/
GCA_000020125.1	Pseudomonadota	Betaproteobacteria	Burkholderiales	Burkholderiaceae	Paraburkholderia	Paraburkholderia phytofirmans	P	https://www.ncbi.nlm.nih.gov/datasets/genome/GCA_000020125.1/
GCA_000013785.1	Pseudomonadota	Gammaproteobacteria	Pseudomonadales	Pseudomonadaceae	Stutzerimonas	Stutzerimonas stutzeri	P	https://www.ncbi.nlm.nih.gov/datasets/genome/GCA_000013785.1/
GCA_000010725.1	Pseudomonadota	Alphaproteobacteria	Rhodospirillales	Azospirillaceae	Azospirillum	Azospirillum sp.	P	https://www.ncbi.nlm.nih.gov/datasets/genome/GCA_000010725.1/
GCA_000010525.1	Pseudomonadota	Alphaproteobacteria	Hyphomicrobiales	Xanthobacteraceae	Azorhizobium	Azorhizobium caulinodans	P	https://www.ncbi.nlm.nih.gov/datasets/genome/GCA_000010525.1/
GCA_036600915.1	Pseudomonadota	Alphaproteobacteria	Hyphomicrobiales	Xanthobacteraceae	Azorhizobium	Azorhizobium caulinodans	P	https://www.ncbi.nlm.nih.gov/datasets/genome/GCA_036600915.1/
GCA_036600855.1	Pseudomonadota	Alphaproteobacteria	Hyphomicrobiales	Xanthobacteraceae	Azorhizobium	Azorhizobium caulinodans	P	https://www.ncbi.nlm.nih.gov/datasets/genome/GCA_036600855.1/
GCA_036600875.1	Pseudomonadota	Alphaproteobacteria	Hyphomicrobiales	Xanthobacteraceae	Azorhizobium	Azorhizobium caulinodans	P	https://www.ncbi.nlm.nih.gov/datasets/genome/GCA_036600875.1/
GCA_036600895.1	Pseudomonadota	Alphaproteobacteria	Hyphomicrobiales	Xanthobacteraceae	Azorhizobium	Azorhizobium caulinodans	P	https://www.ncbi.nlm.nih.gov/datasets/genome/GCA_036600895.1/
GCA_000473085.1	Pseudomonadota	Alphaproteobacteria	Hyphomicrobiales	Xanthobacteraceae	Azorhizobium	Azorhizobium doebereinerae	P	https://www.ncbi.nlm.nih.gov/datasets/genome/GCA_000473085.1/
GCA_008932115.1	Pseudomonadota	Alphaproteobacteria	Hyphomicrobiales	Nitrobacteraceae	Bradyrhizobium	Bradyrhizobium betae	P	https://www.ncbi.nlm.nih.gov/datasets/genome/GCA_008932115.1/
GCA_024585145.1	Pseudomonadota	Alphaproteobacteria	Hyphomicrobiales	Nitrobacteraceae	Bradyrhizobium	Bradyrhizobium betae	P	https://www.ncbi.nlm.nih.gov/datasets/genome/GCA_024585145.1/
GCA_024806875.1	Pseudomonadota	Alphaproteobacteria	Hyphomicrobiales	Nitrobacteraceae	Bradyrhizobium	Bradyrhizobium betae	P	https://www.ncbi.nlm.nih.gov/datasets/genome/GCA_024806875.1/
GCA_004123905.1	Pseudomonadota	Alphaproteobacteria	Hyphomicrobiales	Nitrobacteraceae	Bradyrhizobium	Bradyrhizobium betae	P	https://www.ncbi.nlm.nih.gov/datasets/genome/GCA_004123905.1/
GCA_023278185.1	Pseudomonadota	Alphaproteobacteria	Hyphomicrobiales	Nitrobacteraceae	Bradyrhizobium	Bradyrhizobium elkanii	P	https://www.ncbi.nlm.nih.gov/datasets/genome/GCA_023278185.1/
GCA_030584765.1	Pseudomonadota	Alphaproteobacteria	Hyphomicrobiales	Nitrobacteraceae	Bradyrhizobium	Bradyrhizobium elkanii	P	https://www.ncbi.nlm.nih.gov/datasets/genome/GCA_030584765.1/
GCA_000284375.1	Pseudomonadota	Alphaproteobacteria	Hyphomicrobiales	Nitrobacteraceae	Bradyrhizobium	Bradyrhizobium japonicum	P	https://www.ncbi.nlm.nih.gov/datasets/genome/GCA_000284375.1/
GCA_000261645.1	Pseudomonadota	Alphaproteobacteria	Hyphomicrobiales	Nitrobacteraceae	Bradyrhizobium	Bradyrhizobium liaoningense	P	https://www.ncbi.nlm.nih.gov/datasets/genome/GCA_000261645.1/
GCA_030160735.1	Pseudomonadota	Alphaproteobacteria	Hyphomicrobiales	Nitrobacteraceae	Bradyrhizobium	Bradyrhizobium liaoningense	P	https://www.ncbi.nlm.nih.gov/datasets/genome/GCA_030160735.1/
GCA_018130685.1	Pseudomonadota	Alphaproteobacteria	Hyphomicrobiales	Nitrobacteraceae	Bradyrhizobium	Bradyrhizobium liaoningense	P	https://www.ncbi.nlm.nih.gov/datasets/genome/GCA_018130685.1/
GCA_018130825.1	Pseudomonadota	Alphaproteobacteria	Hyphomicrobiales	Nitrobacteraceae	Bradyrhizobium	Bradyrhizobium liaoningense	P	https://www.ncbi.nlm.nih.gov/datasets/genome/GCA_018130825.1/
GCA_018130725.1	Pseudomonadota	Alphaproteobacteria	Hyphomicrobiales	Nitrobacteraceae	Bradyrhizobium	Bradyrhizobium liaoningense	P	https://www.ncbi.nlm.nih.gov/datasets/genome/GCA_018130725.1/
GCA_900011245.1	Pseudomonadota	Alphaproteobacteria	Hyphomicrobiales	Nitrobacteraceae	Bradyrhizobium	Bradyrhizobium sp.	P	https://www.ncbi.nlm.nih.gov/datasets/genome/GCA_900011245.1/
GCA_900011265.1	Pseudomonadota	Alphaproteobacteria	Hyphomicrobiales	Nitrobacteraceae	Bradyrhizobium	Bradyrhizobium sp.	P	https://www.ncbi.nlm.nih.gov/datasets/genome/GCA_900011265.1/
GCA_020350345.1	Pseudomonadota	Alphaproteobacteria	Hyphomicrobiales	Nitrobacteraceae	Bradyrhizobium	Bradyrhizobium sp.	P	https://www.ncbi.nlm.nih.gov/datasets/genome/GCA_020350345.1/
GCA_037201505.1	Pseudomonadota	Alphaproteobacteria	Hyphomicrobiales	Nitrobacteraceae	Bradyrhizobium	Bradyrhizobium sp.	P	https://www.ncbi.nlm.nih.gov/datasets/genome/GCA_037201505.1/
GCA_035575945.1	Pseudomonadota	Alphaproteobacteria	Hyphomicrobiales	Nitrobacteraceae	Bradyrhizobium	Bradyrhizobium sp.	P	https://www.ncbi.nlm.nih.gov/datasets/genome/GCA_035575945.1/

GCA_029167075.1	Pseudomonadota	Alphaproteobacteria	Hyphomicrobiales	Nitrobacteraceae	Bradyrhizobium	Bradyrhizobium yuanmingense	P	https://www.ncbi.nlm.nih.gov/datasets/genome/GCA_029167075.1/
GCA_025200905.1	Pseudomonadota	Alphaproteobacteria	Hyphomicrobiales	Nitrobacteraceae	Bradyrhizobium	Bradyrhizobium yuanmingense	P	https://www.ncbi.nlm.nih.gov/datasets/genome/GCA_025200905.1/
GCA_005157565.1	Pseudomonadota	Alphaproteobacteria	Hyphomicrobiales	Nitrobacteraceae	Bradyrhizobium	Bradyrhizobium yuanmingense	P	https://www.ncbi.nlm.nih.gov/datasets/genome/GCA_005157565.1/
GCA_900094575.1	Pseudomonadota	Alphaproteobacteria	Hyphomicrobiales	Nitrobacteraceae	Bradyrhizobium	Bradyrhizobium yuanmingense	P	https://www.ncbi.nlm.nih.gov/datasets/genome/GCA_900094575.1/
GCA_029167055.1	Pseudomonadota	Alphaproteobacteria	Hyphomicrobiales	Nitrobacteraceae	Bradyrhizobium	Bradyrhizobium yuanmingense	P	https://www.ncbi.nlm.nih.gov/datasets/genome/GCA_029167055.1/
GCA_034421895.1	Pseudomonadota	Alphaproteobacteria	Hyphomicrobiales	Phyllobacteriaceae	Mesorhizobium	Mesorhizobium huakuii	P	https://www.ncbi.nlm.nih.gov/datasets/genome/GCA_034421895.1/
GCA_014189455.1	Pseudomonadota	Alphaproteobacteria	Hyphomicrobiales	Phyllobacteriaceae	Mesorhizobium	Mesorhizobium huakuii	P	https://www.ncbi.nlm.nih.gov/datasets/genome/GCA_014189455.1/
GCA_030160235.1	Pseudomonadota	Alphaproteobacteria	Hyphomicrobiales	Phyllobacteriaceae	Mesorhizobium	Mesorhizobium huakuii	P	https://www.ncbi.nlm.nih.gov/datasets/genome/GCA_030160235.1/
GCA_000709395.2	Pseudomonadota	Alphaproteobacteria	Hyphomicrobiales	Phyllobacteriaceae	Mesorhizobium	Mesorhizobium huakuii	P	https://www.ncbi.nlm.nih.gov/datasets/genome/GCA_000709395.2/
GCA_003148495.1	Pseudomonadota	Alphaproteobacteria	Hyphomicrobiales	Phyllobacteriaceae	Mesorhizobium	Mesorhizobium loti	P	https://www.ncbi.nlm.nih.gov/datasets/genome/GCA_003148495.1/
GCA_013170705.1	Pseudomonadota	Alphaproteobacteria	Hyphomicrobiales	Phyllobacteriaceae	Mesorhizobium	Mesorhizobium loti	P	https://www.ncbi.nlm.nih.gov/datasets/genome/GCA_013170705.1/
GCA_013170845.1	Pseudomonadota	Alphaproteobacteria	Hyphomicrobiales	Phyllobacteriaceae	Mesorhizobium	Mesorhizobium loti	P	https://www.ncbi.nlm.nih.gov/datasets/genome/GCA_013170845.1/
GCA_001672355.1	Pseudomonadota	Alphaproteobacteria	Hyphomicrobiales	Phyllobacteriaceae	Mesorhizobium	Mesorhizobium loti	P	https://www.ncbi.nlm.nih.gov/datasets/genome/GCA_001672355.1/
GCA_001671485.1	Pseudomonadota	Alphaproteobacteria	Hyphomicrobiales	Phyllobacteriaceae	Mesorhizobium	Mesorhizobium loti	P	https://www.ncbi.nlm.nih.gov/datasets/genome/GCA_001671485.1/
GCA_002119845.1	Pseudomonadota	Alphaproteobacteria	Hyphomicrobiales	Rhizobiaceae	Rhizobium	Rhizobium etli	P	https://www.ncbi.nlm.nih.gov/datasets/genome/GCA_002119845.1/
GCA_000092045.1	Pseudomonadota	Alphaproteobacteria	Hyphomicrobiales	Rhizobiaceae	Rhizobium	Rhizobium etli	P	https://www.ncbi.nlm.nih.gov/datasets/genome/GCA_000092045.1/
GCA_000698845.1	Pseudomonadota	Alphaproteobacteria	Hyphomicrobiales	Rhizobiaceae	Rhizobium	Rhizobium etli	P	https://www.ncbi.nlm.nih.gov/datasets/genome/GCA_000698845.1/
GCA_001908375.1	Pseudomonadota	Alphaproteobacteria	Hyphomicrobiales	Rhizobiaceae	Rhizobium	Rhizobium etli	P	https://www.ncbi.nlm.nih.gov/datasets/genome/GCA_001908375.1/
GCA_000442435.1	Pseudomonadota	Alphaproteobacteria	Hyphomicrobiales	Rhizobiaceae	Rhizobium	Rhizobium etli	P	https://www.ncbi.nlm.nih.gov/datasets/genome/GCA_000442435.1/
GCA_003985135.1	Pseudomonadota	Alphaproteobacteria	Hyphomicrobiales	Rhizobiaceae	Rhizobium	Rhizobium fabae	P	https://www.ncbi.nlm.nih.gov/datasets/genome/GCA_003985135.1/
GCA_014196235.1	Pseudomonadota	Alphaproteobacteria	Hyphomicrobiales	Rhizobiaceae	Rhizobium	Rhizobium fabae	P	https://www.ncbi.nlm.nih.gov/datasets/genome/GCA_014196235.1/
GCA_000731315.1	Pseudomonadota	Alphaproteobacteria	Hyphomicrobiales	Rhizobiaceae	Neorhizobium	Neorhizobium galegae	P	https://www.ncbi.nlm.nih.gov/datasets/genome/GCA_000731315.1/
GCA_021391675.1	Pseudomonadota	Alphaproteobacteria	Hyphomicrobiales	Rhizobiaceae	Neorhizobium	Neorhizobium galegae	P	https://www.ncbi.nlm.nih.gov/datasets/genome/GCA_021391675.1/
GCA_000731295.1	Pseudomonadota	Alphaproteobacteria	Hyphomicrobiales	Rhizobiaceae	Neorhizobium	Neorhizobium galegae	P	https://www.ncbi.nlm.nih.gov/datasets/genome/GCA_000731295.1/
GCA_008806425.1	Pseudomonadota	Alphaproteobacteria	Hyphomicrobiales	Rhizobiaceae	Neorhizobium	Neorhizobium galegae	P	https://www.ncbi.nlm.nih.gov/datasets/genome/GCA_008806425.1/
GCA_024384545.1	Pseudomonadota	Alphaproteobacteria	Hyphomicrobiales	Rhizobiaceae	Neorhizobium	Neorhizobium galegae	P	https://www.ncbi.nlm.nih.gov/datasets/genome/GCA_024384545.1/
GCA_017357305.1	Pseudomonadota	Alphaproteobacteria	Hyphomicrobiales	Rhizobiaceae	Rhizobium	Rhizobium leguminosarum	P	https://www.ncbi.nlm.nih.gov/datasets/genome/GCA_017357305.1/
GCA_001890425.1	Pseudomonadota	Alphaproteobacteria	Hyphomicrobiales	Rhizobiaceae	Rhizobium	Rhizobium leguminosarum	P	https://www.ncbi.nlm.nih.gov/datasets/genome/GCA_001890425.1/
GCA_029714245.1	Pseudomonadota	Alphaproteobacteria	Hyphomicrobiales	Rhizobiaceae	Rhizobium	Rhizobium leguminosarum	P	https://www.ncbi.nlm.nih.gov/datasets/genome/GCA_029714245.1/
GCA_001679785.1	Pseudomonadota	Alphaproteobacteria	Hyphomicrobiales	Rhizobiaceae	Rhizobium	Rhizobium leguminosarum	P	https://www.ncbi.nlm.nih.gov/datasets/genome/GCA_001679785.1/
GCA_003177055.1	Pseudomonadota	Alphaproteobacteria	Hyphomicrobiales	Rhizobiaceae	Sinorhizobium	Sinorhizobium fredii	P	https://www.ncbi.nlm.nih.gov/datasets/genome/GCA_003177055.1/
GCA_000018545.1	Pseudomonadota	Alphaproteobacteria	Hyphomicrobiales	Rhizobiaceae	Sinorhizobium	Sinorhizobium fredii	P	https://www.ncbi.nlm.nih.gov/datasets/genome/GCA_000018545.1/
GCA_024400375.1	Pseudomonadota	Alphaproteobacteria	Hyphomicrobiales	Rhizobiaceae	Sinorhizobium	Sinorhizobium fredii	P	https://www.ncbi.nlm.nih.gov/datasets/genome/GCA_024400375.1/
GCA_002944405.1	Pseudomonadota	Alphaproteobacteria	Hyphomicrobiales	Rhizobiaceae	Sinorhizobium	Sinorhizobium fredii	P	https://www.ncbi.nlm.nih.gov/datasets/genome/GCA_002944405.1/
GCA_003100575.1	Pseudomonadota	Alphaproteobacteria	Hyphomicrobiales	Rhizobiaceae	Sinorhizobium	Sinorhizobium fredii	P	https://www.ncbi.nlm.nih.gov/datasets/genome/GCA_003100575.1/
GCA_037023865.1	Pseudomonadota	Alphaproteobacteria	Hyphomicrobiales	Rhizobiaceae	Sinorhizobium	Sinorhizobium meliloti	P	https://www.ncbi.nlm.nih.gov/datasets/genome/GCA_037023865.1/
GCA_037482275.1	Pseudomonadota	Alphaproteobacteria	Hyphomicrobiales	Rhizobiaceae	Sinorhizobium	Sinorhizobium meliloti	P	https://www.ncbi.nlm.nih.gov/datasets/genome/GCA_037482275.1/
GCA_002197125.1	Pseudomonadota	Alphaproteobacteria	Hyphomicrobiales	Rhizobiaceae	Sinorhizobium	Sinorhizobium meliloti	P	https://www.ncbi.nlm.nih.gov/datasets/genome/GCA_002197125.1/
GCA_002197145.1	Pseudomonadota	Alphaproteobacteria	Hyphomicrobiales	Rhizobiaceae	Sinorhizobium	Sinorhizobium meliloti	P	https://www.ncbi.nlm.nih.gov/datasets/genome/GCA_002197145.1/
GCA_002197025.1	Pseudomonadota	Alphaproteobacteria	Hyphomicrobiales	Rhizobiaceae	Sinorhizobium	Sinorhizobium meliloti	P	https://www.ncbi.nlm.nih.gov/datasets/genome/GCA_002197025.1/
GCA_001315015.1	Pseudomonadota	Alphaproteobacteria	Rhodospirillales	Azospirillaceae	Azospirillum	Azospirillum brasilense	P	https://www.ncbi.nlm.nih.gov/datasets/genome/GCA_001315015.1/
GCA_008274945.1	Pseudomonadota	Alphaproteobacteria	Rhodospirillales	Azospirillaceae	Azospirillum	Azospirillum brasilense	P	https://www.ncbi.nlm.nih.gov/datasets/genome/GCA_008274945.1/
GCA_022023855.1	Pseudomonadota	Alphaproteobacteria	Rhodospirillales	Azospirillaceae	Azospirillum	Azospirillum brasilense	P	https://www.ncbi.nlm.nih.gov/datasets/genome/GCA_022023855.1/
GCA_005222205.1	Pseudomonadota	Alphaproteobacteria	Rhodospirillales	Azospirillaceae	Azospirillum	Azospirillum brasilense	P	https://www.ncbi.nlm.nih.gov/datasets/genome/GCA_005222205.1/
GCA_008364955.1	Pseudomonadota	Alphaproteobacteria	Rhodospirillales	Azospirillaceae	Azospirillum	Azospirillum lipoferum	P	https://www.ncbi.nlm.nih.gov/datasets/genome/GCA_008364955.1/
GCA_000283655.1	Pseudomonadota	Alphaproteobacteria	Rhodospirillales	Azospirillaceae	Azospirillum	Azospirillum lipoferum	P	https://www.ncbi.nlm.nih.gov/datasets/genome/GCA_000283655.1/
GCA_024170005.1	Pseudomonadota	Alphaproteobacteria	Rhodospirillales	Azospirillaceae	Azospirillum	Azospirillum lipoferum	P	https://www.ncbi.nlm.nih.gov/datasets/genome/GCA_024170005.1/
GCA_900177475.1	Pseudomonadota	Alphaproteobacteria	Rhodospirillales	Azospirillaceae	Azospirillum	Azospirillum lipoferum	P	https://www.ncbi.nlm.nih.gov/datasets/genome/GCA_900177475.1/
GCA_900110885.1	Pseudomonadota	Gammaproteobacteria	Pseudomonadales	Pseudomonadaceae	Azotobacter	Azotobacter beijerinckii	P	https://www.ncbi.nlm.nih.gov/datasets/genome/GCA_900110885.1/
GCA_900108965.1	Pseudomonadota	Gammaproteobacteria	Pseudomonadales	Pseudomonadaceae	Azotobacter	Azotobacter beijerinckii	P	https://www.ncbi.nlm.nih.gov/datasets/genome/GCA_900108965.1/
GCA_900112015.1	Pseudomonadota	Gammaproteobacteria	Pseudomonadales	Pseudomonadaceae	Azotobacter	Azotobacter beijerinckii	P	https://www.ncbi.nlm.nih.gov/datasets/genome/GCA_900112015.1/
GCA_900108885.1	Pseudomonadota	Gammaproteobacteria	Pseudomonadales	Pseudomonadaceae	Azotobacter	Azotobacter beijerinckii	P	https://www.ncbi.nlm.nih.gov/datasets/genome/GCA_900108885.1/
GCA_900114395.1	Pseudomonadota	Gammaproteobacteria	Pseudomonadales	Pseudomonadaceae	Azotobacter	Azotobacter beijerinckii	P	https://www.ncbi.nlm.nih.gov/datasets/genome/GCA_900114395.1/
GCA_016406165.1	Pseudomonadota	Gammaproteobacteria	Pseudomonadales	Pseudomonadaceae	Azotobacter	Azotobacter chroococcum	P	https://www.ncbi.nlm.nih.gov/datasets/genome/GCA_016406165.1/
GCA_000817975.1	Pseudomonadota	Gammaproteobacteria	Pseudomonadales	Pseudomonadaceae	Azotobacter	Azotobacter chroococcum	P	https://www.ncbi.nlm.nih.gov/datasets/genome/GCA_000817975.1/
GCA_004327905.1	Pseudomonadota	Gammaproteobacteria	Pseudomonadales	Pseudomonadaceae	Azotobacter	Azotobacter chroococcum	P	https://www.ncbi.nlm.nih.gov/datasets/genome/GCA_004327905.1/

GCA_004339665.1	Pseudomonadota	Gammaproteobacteria	Pseudomonadales	Pseudomonadaceae	Azotobacter	Azotobacter chroococcum	P	https://www.ncbi.nlm.nih.gov/datasets/genome/GCA_004339665.1/
GCA_030506185.1	Pseudomonadota	Gammaproteobacteria	Pseudomonadales	Pseudomonadaceae	Azotobacter	Azotobacter vinelandii	P	https://www.ncbi.nlm.nih.gov/datasets/genome/GCA_030506185.1/
GCA_036687375.1	Pseudomonadota	Gammaproteobacteria	Pseudomonadales	Pseudomonadaceae	Azotobacter	Azotobacter vinelandii	P	https://www.ncbi.nlm.nih.gov/datasets/genome/GCA_036687375.1/
GCA_036687365.1	Pseudomonadota	Gammaproteobacteria	Pseudomonadales	Pseudomonadaceae	Azotobacter	Azotobacter vinelandii	P	https://www.ncbi.nlm.nih.gov/datasets/genome/GCA_036687365.1/
GCA_000380335.1	Pseudomonadota	Gammaproteobacteria	Pseudomonadales	Pseudomonadaceae	Azotobacter	Azotobacter vinelandii	P	https://www.ncbi.nlm.nih.gov/datasets/genome/GCA_000380335.1/
GCA_000019845.1	Pseudomonadota	Alphaproteobacteria	Hyphomicrobiales	Beijerinckiaceae	Beijerinckia	Beijerinckia indica	P	https://www.ncbi.nlm.nih.gov/datasets/genome/GCA_000019845.1/
GCA_001402875.1	Pseudomonadota	Alphaproteobacteria	Hyphomicrobiales	Blastochloridaceae	Blastochloris	Blastochloris viridis	P	https://www.ncbi.nlm.nih.gov/datasets/genome/GCA_001402875.1/
GCA_001548155.2	Pseudomonadota	Alphaproteobacteria	Hyphomicrobiales	Blastochloridaceae	Blastochloris	Blastochloris viridis	P	https://www.ncbi.nlm.nih.gov/datasets/genome/GCA_001548155.2/
GCA_001459775.1	Pseudomonadota	Alphaproteobacteria	Hyphomicrobiales	Blastochloridaceae	Blastochloris	Blastochloris viridis	P	https://www.ncbi.nlm.nih.gov/datasets/genome/GCA_001459775.1/
GCA_005768725.1	Pseudomonadota	Alphaproteobacteria	Hyphomicrobiales	Blastochloridaceae	Blastochloris	Blastochloris viridis	P	https://www.ncbi.nlm.nih.gov/datasets/genome/GCA_005768725.1/
GCA_900108475.1	Pseudomonadota	Alphaproteobacteria	Rhodospirillales	Rhodospirillaceae	Magnetospirillum	Magnetospirillum fulvum	P	https://www.ncbi.nlm.nih.gov/datasets/genome/GCA_900108475.1/
GCA_000442515.1	Pseudomonadota	Alphaproteobacteria	Rhodospirillales	Rhodospirillaceae	Magnetospirillum	Magnetospirillum fulvum	P	https://www.ncbi.nlm.nih.gov/datasets/genome/GCA_000442515.1/
GCA_021391315.1	Pseudomonadota	Alphaproteobacteria	Rhodobacterales	Paracoccaceae	Cereibacter	Cereibacter azotoformans	P	https://www.ncbi.nlm.nih.gov/datasets/genome/GCA_021391315.1/
GCA_022227035.1	Pseudomonadota	Alphaproteobacteria	Rhodobacterales	Paracoccaceae	Cereibacter	Cereibacter azotoformans	P	https://www.ncbi.nlm.nih.gov/datasets/genome/GCA_022227035.1/
GCA_003050905.1	Pseudomonadota	Alphaproteobacteria	Rhodobacterales	Paracoccaceae	Cereibacter	Cereibacter azotoformans	P	https://www.ncbi.nlm.nih.gov/datasets/genome/GCA_003050905.1/
GCA_002844445.1	Pseudomonadota	Alphaproteobacteria	Rhodobacterales	Paracoccaceae	Cereibacter	Cereibacter azotoformans	P	https://www.ncbi.nlm.nih.gov/datasets/genome/GCA_002844445.1/
GCA_017599285.1	Pseudomonadota	Alphaproteobacteria	Rhodobacterales	Paracoccaceae	Cereibacter	Cereibacter azotoformans	P	https://www.ncbi.nlm.nih.gov/datasets/genome/GCA_017599285.1/
GCA_000021865.1	Pseudomonadota	Alphaproteobacteria	Rhodobacterales	Rhodobacteraceae	Rhodobacter	Rhodobacter capsulatus	P	https://www.ncbi.nlm.nih.gov/datasets/genome/GCA_000021865.1/
GCA_029277405.1	Pseudomonadota	Alphaproteobacteria	Rhodobacterales	Rhodobacteraceae	Rhodobacter	Rhodobacter capsulatus	P	https://www.ncbi.nlm.nih.gov/datasets/genome/GCA_029277405.1/
GCA_014622665.1	Pseudomonadota	Alphaproteobacteria	Rhodobacterales	Rhodobacteraceae	Rhodobacter	Rhodobacter capsulatus	P	https://www.ncbi.nlm.nih.gov/datasets/genome/GCA_014622665.1/
GCA_003254295.1	Pseudomonadota	Alphaproteobacteria	Rhodobacterales	Rhodobacteraceae	Rhodobacter	Rhodobacter capsulatus	P	https://www.ncbi.nlm.nih.gov/datasets/genome/GCA_003254295.1/
GCA_006546695.1	Pseudomonadota	Alphaproteobacteria	Rhodobacterales	Rhodobacteraceae	Rhodobacter	Rhodobacter capsulatus	P	https://www.ncbi.nlm.nih.gov/datasets/genome/GCA_006546695.1/
GCA_000021005.1	Pseudomonadota	Alphaproteobacteria	Rhodobacterales	Paracoccaceae	Cereibacter	Cereibacter sphaeroides	P	https://www.ncbi.nlm.nih.gov/datasets/genome/GCA_000021005.1/
GCA_003324715.1	Pseudomonadota	Alphaproteobacteria	Rhodobacterales	Paracoccaceae	Cereibacter	Cereibacter sphaeroides	P	https://www.ncbi.nlm.nih.gov/datasets/genome/GCA_003324715.1/
GCA_000273405.1	Pseudomonadota	Alphaproteobacteria	Rhodobacterales	Paracoccaceae	Cereibacter	Cereibacter sphaeroides	P	https://www.ncbi.nlm.nih.gov/datasets/genome/GCA_000273405.1/
GCA_012647365.1	Pseudomonadota	Alphaproteobacteria	Rhodobacterales	Paracoccaceae	Cereibacter	Cereibacter sphaeroides	P	https://www.ncbi.nlm.nih.gov/datasets/genome/GCA_012647365.1/
GCA_001685625.1	Pseudomonadota	Alphaproteobacteria	Rhodobacterales	Paracoccaceae	Cereibacter	Cereibacter sphaeroides	P	https://www.ncbi.nlm.nih.gov/datasets/genome/GCA_001685625.1/
GCA_900187365.1	Pseudomonadota	Alphaproteobacteria	Hyphomicrobiales	Rhodoblastaceae	Rhodoblastus	Rhodoblastus acidophilus	P	https://www.ncbi.nlm.nih.gov/datasets/genome/GCA_900187365.1/
GCA_002937135.1	Pseudomonadota	Alphaproteobacteria	Hyphomicrobiales	Rhodoblastaceae	Rhodoblastus	Rhodoblastus acidophilus	P	https://www.ncbi.nlm.nih.gov/datasets/genome/GCA_002937135.1/
GCA_003258765.1	Pseudomonadota	Alphaproteobacteria	Hyphomicrobiales	Rhodoblastaceae	Rhodoblastus	Rhodoblastus acidophilus	P	https://www.ncbi.nlm.nih.gov/datasets/genome/GCA_003258765.1/
GCA_025961595.1	Pseudomonadota	Alphaproteobacteria	Hyphomicrobiales	Rhodoblastaceae	Rhodoblastus	Rhodoblastus acidophilus	P	https://www.ncbi.nlm.nih.gov/datasets/genome/GCA_025961595.1/
GCA_025961435.1	Pseudomonadota	Alphaproteobacteria	Hyphomicrobiales	Rhodoblastaceae	Rhodoblastus	Rhodoblastus acidophilus	P	https://www.ncbi.nlm.nih.gov/datasets/genome/GCA_025961435.1/
GCA_002937115.1	Pseudomonadota	Alphaproteobacteria	Rhodospirillales	Acetobacteraceae	Rhodopila	Rhodopila globiformis	P	https://www.ncbi.nlm.nih.gov/datasets/genome/GCA_002937115.1/
GCA_016584445.1	Pseudomonadota	Alphaproteobacteria	Hyphomicrobiales	Nitrobacteraceae	Rhodopseudomonas	Rhodopseudomonas palustris	P	https://www.ncbi.nlm.nih.gov/datasets/genome/GCA_016584445.1/
GCA_025811435.1	Pseudomonadota	Alphaproteobacteria	Hyphomicrobiales	Nitrobacteraceae	Rhodopseudomonas	Rhodopseudomonas palustris	P	https://www.ncbi.nlm.nih.gov/datasets/genome/GCA_025811435.1/
GCA_026625025.1	Pseudomonadota	Alphaproteobacteria	Hyphomicrobiales	Nitrobacteraceae	Rhodopseudomonas	Rhodopseudomonas palustris	P	https://www.ncbi.nlm.nih.gov/datasets/genome/GCA_026625025.1/
GCA_031600295.1	Pseudomonadota	Alphaproteobacteria	Hyphomicrobiales	Nitrobacteraceae	Rhodopseudomonas	Rhodopseudomonas palustris	P	https://www.ncbi.nlm.nih.gov/datasets/genome/GCA_031600295.1/
GCA_027912195.1	Pseudomonadota	Alphaproteobacteria	Hyphomicrobiales	Nitrobacteraceae	Rhodopseudomonas	Rhodopseudomonas palustris	P	https://www.ncbi.nlm.nih.gov/datasets/genome/GCA_027912195.1/
GCA_000225955.1	Pseudomonadota	Alphaproteobacteria	Rhodospirillales	Rhodospirillaceae	Rhodospirillum	Rhodospirillum rubrum	P	https://www.ncbi.nlm.nih.gov/datasets/genome/GCA_000225955.1/
GCA_019134555.1	Pseudomonadota	Alphaproteobacteria	Rhodospirillales	Rhodospirillaceae	Rhodospirillum	Rhodospirillum rubrum	P	https://www.ncbi.nlm.nih.gov/datasets/genome/GCA_019134555.1/
GCA_000013085.1	Pseudomonadota	Alphaproteobacteria	Rhodospirillales	Rhodospirillaceae	Rhodospirillum	Rhodospirillum rubrum	P	https://www.ncbi.nlm.nih.gov/datasets/genome/GCA_000013085.1/
GCA_016583945.1	Pseudomonadota	Alphaproteobacteria	Rhodospirillales	Rhodospirillaceae	Rhodospirillum	Rhodospirillum rubrum	P	https://www.ncbi.nlm.nih.gov/datasets/genome/GCA_016583945.1/
GCA_016583925.1	Pseudomonadota	Alphaproteobacteria	Rhodospirillales	Rhodospirillaceae	Rhodospirillum	Rhodospirillum rubrum	P	https://www.ncbi.nlm.nih.gov/datasets/genome/GCA_016583925.1/
GCA_000515255.1	Pseudomonadota	Alphaproteobacteria	Rhodospirillales	Rhodovibrionaceae	Rhodovibrio	Rhodovibrio salinarum	P	https://www.ncbi.nlm.nih.gov/datasets/genome/GCA_000515255.1/
GCA_016583505.1	Pseudomonadota	Alphaproteobacteria	Rhodospirillales	Rhodovibrionaceae	Rhodovibrio	Rhodovibrio salinarum	P	https://www.ncbi.nlm.nih.gov/datasets/genome/GCA_016583505.1/
GCA_001633145.1	Pseudomonadota	Alphaproteobacteria	Rhodobacterales	Paracoccaceae	Rhodovulum	Rhodovulum sulfidophilum	P	https://www.ncbi.nlm.nih.gov/datasets/genome/GCA_001633145.1/
GCA_001633165.1	Pseudomonadota	Alphaproteobacteria	Rhodobacterales	Paracoccaceae	Rhodovulum	Rhodovulum sulfidophilum	P	https://www.ncbi.nlm.nih.gov/datasets/genome/GCA_001633165.1/
GCA_025961485.1	Pseudomonadota	Alphaproteobacteria	Rhodobacterales	Paracoccaceae	Rhodovulum	Rhodovulum sulfidophilum	P	https://www.ncbi.nlm.nih.gov/datasets/genome/GCA_025961485.1/
GCA_001941695.1	Pseudomonadota	Alphaproteobacteria	Rhodobacterales	Paracoccaceae	Rhodovulum	Rhodovulum sulfidophilum	P	https://www.ncbi.nlm.nih.gov/datasets/genome/GCA_001941695.1/
GCA_010119435.1	Pseudomonadota	Alphaproteobacteria	Rhodobacterales	Paracoccaceae	Rhodovulum	Rhodovulum sulfidophilum	P	https://www.ncbi.nlm.nih.gov/datasets/genome/GCA_010119435.1/
GCA_004340905.1	Pseudomonadota	Betaproteobacteria	Burkholderiales	Sphaerotilaceae	Rubrivivax	Rubrivivax gelatinosus	P	https://www.ncbi.nlm.nih.gov/datasets/genome/GCA_004340905.1/
GCA_000284255.1	Pseudomonadota	Betaproteobacteria	Burkholderiales	Sphaerotilaceae	Rubrivivax	Rubrivivax gelatinosus	P	https://www.ncbi.nlm.nih.gov/datasets/genome/GCA_000284255.1/
GCA_016583525.1	Pseudomonadota	Betaproteobacteria	Burkholderiales	Sphaerotilaceae	Rubrivivax	Rubrivivax gelatinosus	P	https://www.ncbi.nlm.nih.gov/datasets/genome/GCA_016583525.1/
GCA_015751865.1	Pseudomonadota	Betaproteobacteria	Burkholderiales	Sphaerotilaceae	Rubrivivax	Rubrivivax gelatinosus	P	https://www.ncbi.nlm.nih.gov/datasets/genome/GCA_015751865.1/
GCA_016583725.1	Pseudomonadota	Betaproteobacteria	Burkholderiales	Sphaerotilaceae	Rubrivivax	Rubrivivax gelatinosus	P	https://www.ncbi.nlm.nih.gov/datasets/genome/GCA_016583725.1/
GCA_009662475.1	Pseudomonadota	Acidithiobacillia	Acidithiobacillales	Acidithiobacillaceae	Acidithiobacillus	Acidithiobacillus thiooxidans	P	https://www.ncbi.nlm.nih.gov/datasets/genome/GCA_009662475.1/

GCA_006718285.1	Pseudomonadota	Acidithiobacillia	Acidithiobacillales	Acidithiobacillaceae	Acidithiobacillus	Acidithiobacillus thiooxidans	P	https://www.ncbi.nlm.nih.gov/datasets/genome/GCA_006718285.1/
GCA_000227215.2	Pseudomonadota	Acidithiobacillia	Acidithiobacillales	Acidithiobacillaceae	Acidithiobacillus	Acidithiobacillus thiooxidans	P	https://www.ncbi.nlm.nih.gov/datasets/genome/GCA_000227215.2/
GCA_033842445.1	Pseudomonadota	Acidithiobacillia	Acidithiobacillales	Acidithiobacillaceae	Acidithiobacillus	Acidithiobacillus thiooxidans	P	https://www.ncbi.nlm.nih.gov/datasets/genome/GCA_033842445.1/
GCA_031460335.1	Pseudomonadota	Acidithiobacillia	Acidithiobacillales	Acidithiobacillaceae	Acidithiobacillus	Acidithiobacillus thiooxidans	P	https://www.ncbi.nlm.nih.gov/datasets/genome/GCA_031460335.1/
GCA_000832905.1	Bacillota	Bacilli	Bacillales	Bacillaceae	Heyndrickxia	Heyndrickxia coagulans	P	https://www.ncbi.nlm.nih.gov/datasets/genome/GCA_000832905.1/
GCA_001039495.1	Bacillota	Bacilli	Bacillales	Bacillaceae	Heyndrickxia	Heyndrickxia coagulans	P	https://www.ncbi.nlm.nih.gov/datasets/genome/GCA_001039495.1/
GCA_019443305.1	Bacillota	Bacilli	Bacillales	Bacillaceae	Heyndrickxia	Heyndrickxia coagulans	P	https://www.ncbi.nlm.nih.gov/datasets/genome/GCA_019443305.1/
GCA_004114715.1	Bacillota	Bacilli	Bacillales	Bacillaceae	Heyndrickxia	Heyndrickxia coagulans	P	https://www.ncbi.nlm.nih.gov/datasets/genome/GCA_004114715.1/
GCA_003184245.1	Bacillota	Bacilli	Bacillales	Bacillaceae	Heyndrickxia	Heyndrickxia coagulans	P	https://www.ncbi.nlm.nih.gov/datasets/genome/GCA_003184245.1/
GCA_030123345.1	Bacillota	Bacilli	Bacillales	Bacillaceae	Cytobacillus	Cytobacillus firmus	P	https://www.ncbi.nlm.nih.gov/datasets/genome/GCA_030123345.1/
GCA_030123225.1	Bacillota	Bacilli	Bacillales	Bacillaceae	Cytobacillus	Cytobacillus firmus	P	https://www.ncbi.nlm.nih.gov/datasets/genome/GCA_030123225.1/
GCA_023657595.1	Bacillota	Bacilli	Bacillales	Bacillaceae	Cytobacillus	Cytobacillus firmus	P	https://www.ncbi.nlm.nih.gov/datasets/genome/GCA_023657595.1/
GCA_025732135.1	Bacillota	Bacilli	Bacillales	Bacillaceae	Cytobacillus	Cytobacillus firmus	P	https://www.ncbi.nlm.nih.gov/datasets/genome/GCA_025732135.1/
GCA_002243645.1	Bacillota	Bacilli	Bacillales	Bacillaceae	Peribacillus	Peribacillus simplex	P	https://www.ncbi.nlm.nih.gov/datasets/genome/GCA_002243645.1/
GCA_030123325.1	Bacillota	Bacilli	Bacillales	Bacillaceae	Peribacillus	Peribacillus simplex	P	https://www.ncbi.nlm.nih.gov/datasets/genome/GCA_030123325.1/
GCA_039905915.1	Bacillota	Bacilli	Bacillales	Bacillaceae	Peribacillus	Peribacillus simplex	P	https://www.ncbi.nlm.nih.gov/datasets/genome/GCA_039905915.1/
GCA_030123045.1	Bacillota	Bacilli	Bacillales	Bacillaceae	Peribacillus	Peribacillus simplex	P	https://www.ncbi.nlm.nih.gov/datasets/genome/GCA_030123045.1/
GCA_002706795.1	Bacillota	Bacilli	Bacillales	Paenibacillaceae	Brevibacillus	Brevibacillus laterosporus	P	https://www.ncbi.nlm.nih.gov/datasets/genome/GCA_002706795.1/
GCA_003590075.1	Bacillota	Bacilli	Bacillales	Paenibacillaceae	Brevibacillus	Brevibacillus laterosporus	P	https://www.ncbi.nlm.nih.gov/datasets/genome/GCA_003590075.1/
GCA_033192675.1	Bacillota	Bacilli	Bacillales	Paenibacillaceae	Brevibacillus	Brevibacillus laterosporus	P	https://www.ncbi.nlm.nih.gov/datasets/genome/GCA_033192675.1/
GCA_002865525.1	Bacillota	Bacilli	Bacillales	Paenibacillaceae	Brevibacillus	Brevibacillus laterosporus	P	https://www.ncbi.nlm.nih.gov/datasets/genome/GCA_002865525.1/
GCA_003663745.1	Bacillota	Bacilli	Bacillales	Paenibacillaceae	Brevibacillus	Brevibacillus laterosporus	P	https://www.ncbi.nlm.nih.gov/datasets/genome/GCA_003663745.1/
GCA_001187725.1	Bacillota	Bacilli	Bacillales	Paenibacillaceae	Brevibacillus	Brevibacillus reuszeri	P	https://www.ncbi.nlm.nih.gov/datasets/genome/GCA_001187725.1/
GCA_006540225.1	Bacillota	Bacilli	Bacillales	Paenibacillaceae	Brevibacillus	Brevibacillus reuszeri	P	https://www.ncbi.nlm.nih.gov/datasets/genome/GCA_006540225.1/
GCA_018333155.1	Bacillota	Bacilli	Bacillales	Paenibacillaceae	Brevibacillus	Brevibacillus reuszeri	P	https://www.ncbi.nlm.nih.gov/datasets/genome/GCA_018333155.1/
GCA_036213185.1	Bacillota	Bacilli	Bacillales	Paenibacillaceae	Brevibacillus	Brevibacillus reuszeri	P	https://www.ncbi.nlm.nih.gov/datasets/genome/GCA_036213185.1/
GCA_002897295.1	Bacillota	Bacilli	Bacillales	Paenibacillaceae	Brevibacillus	Brevibacillus reuszeri	P	https://www.ncbi.nlm.nih.gov/datasets/genome/GCA_002897295.1/
GCA_000756615.1	Bacillota	Bacilli	Bacillales	Paenibacillaceae	Paenibacillus	Paenibacillus durus	P	https://www.ncbi.nlm.nih.gov/datasets/genome/GCA_000756615.1/
GCA_000993825.1	Bacillota	Bacilli	Bacillales	Paenibacillaceae	Paenibacillus	Paenibacillus durus	P	https://www.ncbi.nlm.nih.gov/datasets/genome/GCA_000993825.1/
GCA_000520635.1	Bacillota	Bacilli	Bacillales	Paenibacillaceae	Paenibacillus	Paenibacillus durus	P	https://www.ncbi.nlm.nih.gov/datasets/genome/GCA_000520635.1/
GCA_029542585.1	Bacillota	Bacilli	Bacillales	Paenibacillaceae	Paenibacillus	Paenibacillus mucilaginosus	P	https://www.ncbi.nlm.nih.gov/datasets/genome/GCA_029542585.1/
GCA_000250655.1	Bacillota	Bacilli	Bacillales	Paenibacillaceae	Paenibacillus	Paenibacillus mucilaginosus	P	https://www.ncbi.nlm.nih.gov/datasets/genome/GCA_000250655.1/
GCA_000218915.1	Bacillota	Bacilli	Bacillales	Paenibacillaceae	Paenibacillus	Paenibacillus mucilaginosus	P	https://www.ncbi.nlm.nih.gov/datasets/genome/GCA_000218915.1/
GCA_028751605.1	Bacillota	Bacilli	Bacillales	Paenibacillaceae	Paenibacillus	Paenibacillus mucilaginosus	P	https://www.ncbi.nlm.nih.gov/datasets/genome/GCA_028751605.1/
GCA_000236805.2	Bacillota	Bacilli	Bacillales	Paenibacillaceae	Paenibacillus	Paenibacillus peoriae	P	https://www.ncbi.nlm.nih.gov/datasets/genome/GCA_000236805.2/
GCA_014692735.1	Bacillota	Bacilli	Bacillales	Paenibacillaceae	Paenibacillus	Paenibacillus peoriae	P	https://www.ncbi.nlm.nih.gov/datasets/genome/GCA_014692735.1/
GCA_036630045.1	Bacillota	Bacilli	Bacillales	Paenibacillaceae	Paenibacillus	Paenibacillus peoriae	P	https://www.ncbi.nlm.nih.gov/datasets/genome/GCA_036630045.1/
GCA_040024215.1	Bacillota	Bacilli	Bacillales	Paenibacillaceae	Paenibacillus	Paenibacillus peoriae	P	https://www.ncbi.nlm.nih.gov/datasets/genome/GCA_040024215.1/
GCA_001272655.2	Bacillota	Bacilli	Bacillales	Paenibacillaceae	Paenibacillus	Paenibacillus peoriae	P	https://www.ncbi.nlm.nih.gov/datasets/genome/GCA_001272655.2/
GCA_015710975.1	Bacillota	Bacilli	Bacillales	Paenibacillaceae	Paenibacillus	Paenibacillus polymyxa	P	https://www.ncbi.nlm.nih.gov/datasets/genome/GCA_015710975.1/
GCA_022811565.1	Bacillota	Bacilli	Bacillales	Paenibacillaceae	Paenibacillus	Paenibacillus polymyxa	P	https://www.ncbi.nlm.nih.gov/datasets/genome/GCA_022811565.1/
GCA_001874425.3	Bacillota	Bacilli	Bacillales	Paenibacillaceae	Paenibacillus	Paenibacillus polymyxa	P	https://www.ncbi.nlm.nih.gov/datasets/genome/GCA_001874425.3/
GCA_001922145.1	Bacillota	Bacilli	Bacillales	Paenibacillaceae	Paenibacillus	Paenibacillus polymyxa	P	https://www.ncbi.nlm.nih.gov/datasets/genome/GCA_001922145.1/
GCA_000389675.2	Bacillota	Bacilli	Lactobacillales	Lactobacillaceae	Lactobacillus	Lactobacillus acidophilus	P	https://www.ncbi.nlm.nih.gov/datasets/genome/GCA_000389675.2/
GCA_003047065.1	Bacillota	Bacilli	Lactobacillales	Lactobacillaceae	Lactobacillus	Lactobacillus acidophilus	P	https://www.ncbi.nlm.nih.gov/datasets/genome/GCA_003047065.1/
GCA_034298135.1	Bacillota	Bacilli	Lactobacillales	Lactobacillaceae	Lactobacillus	Lactobacillus acidophilus	P	https://www.ncbi.nlm.nih.gov/datasets/genome/GCA_034298135.1/
GCA_003952845.1	Bacillota	Bacilli	Lactobacillales	Lactobacillaceae	Lactobacillus	Lactobacillus acidophilus	P	https://www.ncbi.nlm.nih.gov/datasets/genome/GCA_003952845.1/
GCA_024397395.1	Bacillota	Bacilli	Lactobacillales	Lactobacillaceae	Lactobacillus	Lactobacillus acidophilus	P	https://www.ncbi.nlm.nih.gov/datasets/genome/GCA_024397395.1/
GCA_003813165.1	Bacillota	Bacilli	Lactobacillales	Lactobacillaceae	Levilactobacillus	Levilactobacillus brevis	P	https://www.ncbi.nlm.nih.gov/datasets/genome/GCA_003813165.1/
GCA_900475625.1	Bacillota	Bacilli	Lactobacillales	Lactobacillaceae	Levilactobacillus	Levilactobacillus brevis	P	https://www.ncbi.nlm.nih.gov/datasets/genome/GCA_900475625.1/
GCA_002762175.1	Bacillota	Bacilli	Lactobacillales	Lactobacillaceae	Levilactobacillus	Levilactobacillus brevis	P	https://www.ncbi.nlm.nih.gov/datasets/genome/GCA_002762175.1/
GCA_006228205.1	Bacillota	Bacilli	Lactobacillales	Lactobacillaceae	Levilactobacillus	Levilactobacillus brevis	P	https://www.ncbi.nlm.nih.gov/datasets/genome/GCA_006228205.1/
GCA_006228285.1	Bacillota	Bacilli	Lactobacillales	Lactobacillaceae	Levilactobacillus	Levilactobacillus brevis	P	https://www.ncbi.nlm.nih.gov/datasets/genome/GCA_006228285.1/
GCA_900015005.1	Bacillota	Bacilli	Lactobacillales	Lactobacillaceae	Paucilactobacillus	Paucilactobacillus oligofermentans	P	https://www.ncbi.nlm.nih.gov/datasets/genome/GCA_900015005.1/
GCA_943193025.1	Bacillota	Bacilli	Lactobacillales	Lactobacillaceae	Lactobacillus	Lactobacillus agrestimuris	P	https://www.ncbi.nlm.nih.gov/datasets/genome/GCA_943193025.1/
GCA_000504525.1	Bacillota	Bacilli	Lactobacillales	Lactobacillaceae	Ligilactobacillus	Ligilactobacillus equi	P	https://www.ncbi.nlm.nih.gov/datasets/genome/GCA_000504525.1/

GCA_900070175.1	Bacillota	Bacilli	Lactobacillales	Lactobacillaceae	Lacticaseibacillus	Lacticaseibacillus rhamnosus	P	https://www.ncbi.nlm.nih.gov/datasets/genome/GCA_900070175.1/
GCA_900196735.1	Bacillota	Bacilli	Lactobacillales	Lactobacillaceae	Lactobacillus	Lactobacillus delbrueckii	P	https://www.ncbi.nlm.nih.gov/datasets/genome/GCA_900196735.1/
GCA_001888925.1	Bacillota	Bacilli	Lactobacillales	Lactobacillaceae	Lactobacillus	Lactobacillus delbrueckii	P	https://www.ncbi.nlm.nih.gov/datasets/genome/GCA_001888925.1/
GCA_006740305.1	Bacillota	Bacilli	Lactobacillales	Lactobacillaceae	Lactobacillus	Lactobacillus delbrueckii	P	https://www.ncbi.nlm.nih.gov/datasets/genome/GCA_006740305.1/
GCA_000056065.1	Bacillota	Bacilli	Lactobacillales	Lactobacillaceae	Lactobacillus	Lactobacillus delbrueckii	P	https://www.ncbi.nlm.nih.gov/datasets/genome/GCA_000056065.1/
GCA_001908415.1	Bacillota	Bacilli	Lactobacillales	Lactobacillaceae	Lactobacillus	Lactobacillus delbrueckii	P	https://www.ncbi.nlm.nih.gov/datasets/genome/GCA_001908415.1/
GCA_002278095.1	Bacillota	Bacilli	Lactobacillales	Lactobacillaceae	Lactobacillus	Lactobacillus delbrueckii	P	https://www.ncbi.nlm.nih.gov/datasets/genome/GCA_002278095.1/
GCA_003053085.1	Bacillota	Bacilli	Lactobacillales	Lactobacillaceae	Lactobacillus	Lactobacillus helveticus	P	https://www.ncbi.nlm.nih.gov/datasets/genome/GCA_003053085.1/
GCA_018408455.1	Bacillota	Bacilli	Lactobacillales	Lactobacillaceae	Lactobacillus	Lactobacillus helveticus	P	https://www.ncbi.nlm.nih.gov/datasets/genome/GCA_018408455.1/
GCA_002849935.1	Bacillota	Bacilli	Lactobacillales	Lactobacillaceae	Lactobacillus	Lactobacillus helveticus	P	https://www.ncbi.nlm.nih.gov/datasets/genome/GCA_002849935.1/
GCA_002849955.1	Bacillota	Bacilli	Lactobacillales	Lactobacillaceae	Lactobacillus	Lactobacillus helveticus	P	https://www.ncbi.nlm.nih.gov/datasets/genome/GCA_002849955.1/
GCA_039623715.1	Bacillota	Bacilli	Lactobacillales	Lactobacillaceae	Lactobacillus	Lactobacillus helveticus	P	https://www.ncbi.nlm.nih.gov/datasets/genome/GCA_039623715.1/
GCA_000155515.2	Bacillota	Bacilli	Lactobacillales	Lactobacillaceae	Lacticaseibacillus	Lacticaseibacillus paracasei	P	https://www.ncbi.nlm.nih.gov/datasets/genome/GCA_000155515.2/
GCA_000829035.1	Bacillota	Bacilli	Lactobacillales	Lactobacillaceae	Lacticaseibacillus	Lacticaseibacillus paracasei	P	https://www.ncbi.nlm.nih.gov/datasets/genome/GCA_000829035.1/
GCA_028609725.1	Bacillota	Bacilli	Lactobacillales	Lactobacillaceae	Lacticaseibacillus	Lacticaseibacillus paracasei	P	https://www.ncbi.nlm.nih.gov/datasets/genome/GCA_028609725.1/
GCA_030061895.1	Bacillota	Bacilli	Lactobacillales	Lactobacillaceae	Lacticaseibacillus	Lacticaseibacillus paracasei	P	https://www.ncbi.nlm.nih.gov/datasets/genome/GCA_030061895.1/
GCA_026013725.1	Bacillota	Bacilli	Lactobacillales	Lactobacillaceae	Lacticaseibacillus	Lacticaseibacillus paracasei	P	https://www.ncbi.nlm.nih.gov/datasets/genome/GCA_026013725.1/
GCA_005864225.1	Bacillota	Bacilli	Lactobacillales	Lactobacillaceae	Lentilactobacillus	Lentilactobacillus parafarraginis	P	https://www.ncbi.nlm.nih.gov/datasets/genome/GCA_005864225.1/
GCA_001435895.1	Bacillota	Bacilli	Lactobacillales	Lactobacillaceae	Lentilactobacillus	Lentilactobacillus parafarraginis	P	https://www.ncbi.nlm.nih.gov/datasets/genome/GCA_001435895.1/
GCA_001311355.1	Bacillota	Bacilli	Lactobacillales	Lactobacillaceae	Lentilactobacillus	Lentilactobacillus parafarraginis	P	https://www.ncbi.nlm.nih.gov/datasets/genome/GCA_001311355.1/
GCA_000238835.1	Bacillota	Bacilli	Lactobacillales	Lactobacillaceae	Lentilactobacillus	Lentilactobacillus parafarraginis	P	https://www.ncbi.nlm.nih.gov/datasets/genome/GCA_000238835.1/
GCA_902388245.1	Bacillota	Bacilli	Lactobacillales	Lactobacillaceae	Lentilactobacillus	Lentilactobacillus parafarraginis	P	https://www.ncbi.nlm.nih.gov/datasets/genome/GCA_902388245.1/
GCA_006151905.1	Bacillota	Bacilli	Lactobacillales	Lactobacillaceae	Lacticaseibacillus	Lacticaseibacillus rhamnosus	P	https://www.ncbi.nlm.nih.gov/datasets/genome/GCA_006151905.1/
GCA_900636965.1	Bacillota	Bacilli	Lactobacillales	Lactobacillaceae	Lacticaseibacillus	Lacticaseibacillus rhamnosus	P	https://www.ncbi.nlm.nih.gov/datasets/genome/GCA_900636965.1/
GCA_016653515.1	Bacillota	Bacilli	Lactobacillales	Lactobacillaceae	Lacticaseibacillus	Lacticaseibacillus rhamnosus	P	https://www.ncbi.nlm.nih.gov/datasets/genome/GCA_016653515.1/
GCA_032465975.1	Bacillota	Bacilli	Lactobacillales	Lactobacillaceae	Lacticaseibacillus	Lacticaseibacillus rhamnosus	P	https://www.ncbi.nlm.nih.gov/datasets/genome/GCA_032465975.1/
GCA_031593775.1	Bacillota	Bacilli	Lactobacillales	Lactobacillaceae	Lacticaseibacillus	Lacticaseibacillus rhamnosus	P	https://www.ncbi.nlm.nih.gov/datasets/genome/GCA_031593775.1/
GCA_003176835.1	Bacillota	Bacilli	Lactobacillales	Streptococcaceae	Lactococcus	Lactococcus lactis	P	https://www.ncbi.nlm.nih.gov/datasets/genome/GCA_003176835.1/
GCA_029023865.1	Bacillota	Bacilli	Lactobacillales	Streptococcaceae	Lactococcus	Lactococcus lactis	P	https://www.ncbi.nlm.nih.gov/datasets/genome/GCA_029023865.1/
GCA_016028835.1	Bacillota	Bacilli	Lactobacillales	Streptococcaceae	Lactococcus	Lactococcus lactis	P	https://www.ncbi.nlm.nih.gov/datasets/genome/GCA_016028835.1/
GCA_016649195.2	Bacillota	Bacilli	Lactobacillales	Streptococcaceae	Lactococcus	Lactococcus lactis	P	https://www.ncbi.nlm.nih.gov/datasets/genome/GCA_016649195.2/
GCA_028994235.1	Bacillota	Bacilli	Lactobacillales	Streptococcaceae	Lactococcus	Lactococcus lactis	P	https://www.ncbi.nlm.nih.gov/datasets/genome/GCA_028994235.1/
GCA_000014505.1	Bacillota	Bacilli	Lactobacillales	Lactobacillaceae	Pediococcus	Pediococcus pentosaceus	P	https://www.ncbi.nlm.nih.gov/datasets/genome/GCA_000014505.1/
GCA_016127775.1	Bacillota	Bacilli	Lactobacillales	Lactobacillaceae	Pediococcus	Pediococcus pentosaceus	P	https://www.ncbi.nlm.nih.gov/datasets/genome/GCA_016127775.1/
GCA_030480445.1	Bacillota	Bacilli	Lactobacillales	Lactobacillaceae	Pediococcus	Pediococcus pentosaceus	P	https://www.ncbi.nlm.nih.gov/datasets/genome/GCA_030480445.1/
GCA_033882205.1	Bacillota	Bacilli	Lactobacillales	Lactobacillaceae	Pediococcus	Pediococcus pentosaceus	P	https://www.ncbi.nlm.nih.gov/datasets/genome/GCA_033882205.1/
GCA_007923185.1	Bacillota	Bacilli	Lactobacillales	Lactobacillaceae	Pediococcus	Pediococcus pentosaceus	P	https://www.ncbi.nlm.nih.gov/datasets/genome/GCA_007923185.1/
GCA_903886475.1	Bacillota	Bacilli	Lactobacillales	Streptococcaceae	Streptococcus	Streptococcus thermophilus	P	https://www.ncbi.nlm.nih.gov/datasets/genome/GCA_903886475.1/
GCA_010120595.1	Bacillota	Bacilli	Lactobacillales	Streptococcaceae	Streptococcus	Streptococcus thermophilus	P	https://www.ncbi.nlm.nih.gov/datasets/genome/GCA_010120595.1/
GCA_900474985.1	Bacillota	Bacilli	Lactobacillales	Streptococcaceae	Streptococcus	Streptococcus thermophilus	P	https://www.ncbi.nlm.nih.gov/datasets/genome/GCA_900474985.1/
GCA_903886645.1	Bacillota	Bacilli	Lactobacillales	Streptococcaceae	Streptococcus	Streptococcus thermophilus	P	https://www.ncbi.nlm.nih.gov/datasets/genome/GCA_903886645.1/
GCA_903886745.1	Bacillota	Bacilli	Lactobacillales	Streptococcaceae	Streptococcus	Streptococcus thermophilus	P	https://www.ncbi.nlm.nih.gov/datasets/genome/GCA_903886745.1/
GCA_000197735.1	Actinomycetota	Actinomycetes	Micrococcales	Micrococcaceae	Glutamicibacter	Glutamicibacter arilaitensis	P	https://www.ncbi.nlm.nih.gov/datasets/genome/GCA_000197735.1/
GCA_004525745.1	Actinomycetota	Actinomycetes	Micrococcales	Micrococcaceae	Glutamicibacter	Glutamicibacter arilaitensis	P	https://www.ncbi.nlm.nih.gov/datasets/genome/GCA_004525745.1/
GCA_002878675.1	Actinomycetota	Actinomycetes	Micrococcales	Micrococcaceae	Glutamicibacter	Glutamicibacter arilaitensis	P	https://www.ncbi.nlm.nih.gov/datasets/genome/GCA_002878675.1/
GCA_014897395.1	Actinomycetota	Actinomycetes	Micrococcales	Micrococcaceae	Glutamicibacter	Glutamicibacter arilaitensis	P	https://www.ncbi.nlm.nih.gov/datasets/genome/GCA_014897395.1/
GCA_006538985.1	Actinomycetota	Actinomycetes	Micrococcales	Micrococcaceae	Paenarthrobacter	Paenarthrobacter aurescens	P	https://www.ncbi.nlm.nih.gov/datasets/genome/GCA_006538985.1/
GCA_039535035.1	Actinomycetota	Actinomycetes	Micrococcales	Micrococcaceae	Paenarthrobacter	Paenarthrobacter aurescens	P	https://www.ncbi.nlm.nih.gov/datasets/genome/GCA_039535035.1/
GCA_025421775.1	Actinomycetota	Actinomycetes	Micrococcales	Micrococcaceae	Paenarthrobacter	Paenarthrobacter aurescens	P	https://www.ncbi.nlm.nih.gov/datasets/genome/GCA_025421775.1/
GCA_030546435.1	Actinomycetota	Actinomycetes	Micrococcales	Micrococcaceae	Paenarthrobacter	Paenarthrobacter aurescens	P	https://www.ncbi.nlm.nih.gov/datasets/genome/GCA_030546435.1/
GCA_030546425.1	Actinomycetota	Actinomycetes	Micrococcales	Micrococcaceae	Paenarthrobacter	Paenarthrobacter aurescens	P	https://www.ncbi.nlm.nih.gov/datasets/genome/GCA_030546425.1/
GCA_040208375.1	Bacillota	Bacilli	Bacillales	Bacillaceae	Shouchella	Shouchella clausii	P	https://www.ncbi.nlm.nih.gov/datasets/genome/GCA_040208375.1/
GCA_038098565.1	Bacillota	Bacilli	Bacillales	Bacillaceae	Shouchella	Shouchella clausii	P	https://www.ncbi.nlm.nih.gov/datasets/genome/GCA_038098565.1/
GCA_040208385.1	Bacillota	Bacilli	Bacillales	Bacillaceae	Shouchella	Shouchella clausii	P	https://www.ncbi.nlm.nih.gov/datasets/genome/GCA_040208385.1/
GCA_002208825.2	Pseudomonadota	Alphaproteobacteria	Caulobacterales	Caulobacteraceae	Brevundimonas	Brevundimonas vesicularis	P	https://www.ncbi.nlm.nih.gov/datasets/genome/GCA_002208825.2/
GCA_027105095.1	Pseudomonadota	Alphaproteobacteria	Caulobacterales	Caulobacteraceae	Brevundimonas	Brevundimonas vesicularis	P	https://www.ncbi.nlm.nih.gov/datasets/genome/GCA_027105095.1/

GCA_027886425.1	Pseudomonadota	Alphaproteobacteria	Caulobacterales	Caulobacteraceae	Brevundimonas	Brevundimonas vesicularis	P	https://www.ncbi.nlm.nih.gov/datasets/genome/GCA_027886425.1/
GCA_001592205.1	Pseudomonadota	Alphaproteobacteria	Caulobacterales	Caulobacteraceae	Brevundimonas	Brevundimonas vesicularis	P	https://www.ncbi.nlm.nih.gov/datasets/genome/GCA_001592205.1/
GCA_030818665.1	Pseudomonadota	Alphaproteobacteria	Caulobacterales	Caulobacteraceae	Brevundimonas	Brevundimonas vesicularis	P	https://www.ncbi.nlm.nih.gov/datasets/genome/GCA_030818665.1/
GCA_001571145.1	Pseudomonadota	Betaproteobacteria	Burkholderiales	Comamonadaceae	Hydrogenophaga	Hydrogenophaga flava	P	https://www.ncbi.nlm.nih.gov/datasets/genome/GCA_001571145.1/
GCA_024927925.1	Bacillota	Bacilli	Bacillales	Thermoactinomycetaceae	Laceyella	Laceyella sacchari	P	https://www.ncbi.nlm.nih.gov/datasets/genome/GCA_024927925.1/
GCA_004343255.1	Bacillota	Bacilli	Bacillales	Thermoactinomycetaceae	Laceyella	Laceyella sacchari	P	https://www.ncbi.nlm.nih.gov/datasets/genome/GCA_004343255.1/
GCA_020546525.1	Bacillota	Bacilli	Bacillales	Bacillaceae	Lysinibacillus	Lysinibacillus sphaericus	P	https://www.ncbi.nlm.nih.gov/datasets/genome/GCA_020546525.1/
GCA_002982115.1	Bacillota	Bacilli	Bacillales	Bacillaceae	Lysinibacillus	Lysinibacillus sphaericus	P	https://www.ncbi.nlm.nih.gov/datasets/genome/GCA_002982115.1/
GCA_001629735.1	Bacillota	Bacilli	Bacillales	Bacillaceae	Lysinibacillus	Lysinibacillus sphaericus	P	https://www.ncbi.nlm.nih.gov/datasets/genome/GCA_001629735.1/
GCA_001581875.1	Bacillota	Bacilli	Bacillales	Bacillaceae	Lysinibacillus	Lysinibacillus sphaericus	P	https://www.ncbi.nlm.nih.gov/datasets/genome/GCA_001581875.1/
GCA_001598075.1	Bacillota	Bacilli	Bacillales	Bacillaceae	Lysinibacillus	Lysinibacillus sphaericus	P	https://www.ncbi.nlm.nih.gov/datasets/genome/GCA_001598075.1/
GCA_014647735.1	Actinomycetota	Actinomycetes	Micrococcales	Promicromonosporaceae	Promicromonospora	Promicromonospora citrea	P	https://www.ncbi.nlm.nih.gov/datasets/genome/GCA_014647735.1/
GCA_013004695.1	Actinomycetota	Actinomycetes	Micrococcales	Promicromonosporaceae	Promicromonospora	Promicromonospora citrea	P	https://www.ncbi.nlm.nih.gov/datasets/genome/GCA_013004695.1/
GCA_900215245.1	Pseudomonadota	Gammaproteobacteria	Pseudomonadales	Pseudomonadaceae	Pseudomonas	Pseudomonas fluorescens	P	https://www.ncbi.nlm.nih.gov/datasets/genome/GCA_900215245.1/
GCA_900475215.1	Pseudomonadota	Gammaproteobacteria	Pseudomonadales	Pseudomonadaceae	Pseudomonas	Pseudomonas fluorescens	P	https://www.ncbi.nlm.nih.gov/datasets/genome/GCA_900475215.1/
GCA_001307275.1	Pseudomonadota	Gammaproteobacteria	Pseudomonadales	Pseudomonadaceae	Pseudomonas	Pseudomonas fluorescens	P	https://www.ncbi.nlm.nih.gov/datasets/genome/GCA_001307275.1/
GCA_015074865.1	Pseudomonadota	Gammaproteobacteria	Pseudomonadales	Pseudomonadaceae	Pseudomonas	Pseudomonas fluorescens	P	https://www.ncbi.nlm.nih.gov/datasets/genome/GCA_015074865.1/
GCA_038447645.1	Pseudomonadota	Gammaproteobacteria	Pseudomonadales	Pseudomonadaceae	Pseudomonas	Pseudomonas fluorescens	P	https://www.ncbi.nlm.nih.gov/datasets/genome/GCA_038447645.1/
GCA_000412675.1	Pseudomonadota	Gammaproteobacteria	Pseudomonadales	Pseudomonadaceae	Pseudomonas	Pseudomonas putida	P	https://www.ncbi.nlm.nih.gov/datasets/genome/GCA_000412675.1/
GCA_024508115.1	Pseudomonadota	Gammaproteobacteria	Pseudomonadales	Pseudomonadaceae	Pseudomonas	Pseudomonas putida	P	https://www.ncbi.nlm.nih.gov/datasets/genome/GCA_024508115.1/
GCA_003228315.1	Pseudomonadota	Gammaproteobacteria	Pseudomonadales	Pseudomonadaceae	Pseudomonas	Pseudomonas putida	P	https://www.ncbi.nlm.nih.gov/datasets/genome/GCA_003228315.1/
GCA_001630725.2	Pseudomonadota	Gammaproteobacteria	Pseudomonadales	Pseudomonadaceae	Pseudomonas	Pseudomonas putida	P	https://www.ncbi.nlm.nih.gov/datasets/genome/GCA_001630725.2/
GCA_002356095.1	Pseudomonadota	Gammaproteobacteria	Pseudomonadales	Pseudomonadaceae	Pseudomonas	Pseudomonas putida	P	https://www.ncbi.nlm.nih.gov/datasets/genome/GCA_002356095.1/
GCA_019704535.1	Pseudomonadota	Gammaproteobacteria	Pseudomonadales	Pseudomonadaceae	Stutzerimonas	Stutzerimonas stutzeri	P	https://www.ncbi.nlm.nih.gov/datasets/genome/GCA_019704535.1/
GCA_015291885.1	Pseudomonadota	Gammaproteobacteria	Pseudomonadales	Pseudomonadaceae	Stutzerimonas	Stutzerimonas stutzeri	P	https://www.ncbi.nlm.nih.gov/datasets/genome/GCA_015291885.1/
GCA_000219605.1	Pseudomonadota	Gammaproteobacteria	Pseudomonadales	Pseudomonadaceae	Stutzerimonas	Stutzerimonas stutzeri	P	https://www.ncbi.nlm.nih.gov/datasets/genome/GCA_000219605.1/
GCA_016028655.1	Pseudomonadota	Gammaproteobacteria	Pseudomonadales	Pseudomonadaceae	Stutzerimonas	Stutzerimonas stutzeri	P	https://www.ncbi.nlm.nih.gov/datasets/genome/GCA_016028655.1/
GCA_001648195.1	Pseudomonadota	Gammaproteobacteria	Pseudomonadales	Pseudomonadaceae	Stutzerimonas	Stutzerimonas stutzeri	P	https://www.ncbi.nlm.nih.gov/datasets/genome/GCA_001648195.1/
GCA_016725865.1	Bacteroidota	Sphingobacteriia	Sphingobacteriales	Sphingobacteriaceae	Sphingobacterium	Sphingobacterium multivorum	P	https://www.ncbi.nlm.nih.gov/datasets/genome/GCA_016725865.1/
GCA_016725465.1	Bacteroidota	Sphingobacteriia	Sphingobacteriales	Sphingobacteriaceae	Sphingobacterium	Sphingobacterium multivorum	P	https://www.ncbi.nlm.nih.gov/datasets/genome/GCA_016725465.1/
GCA_016725925.1	Bacteroidota	Sphingobacteriia	Sphingobacteriales	Sphingobacteriaceae	Sphingobacterium	Sphingobacterium multivorum	P	https://www.ncbi.nlm.nih.gov/datasets/genome/GCA_016725925.1/
GCA_039511225.1	Bacteroidota	Sphingobacteriia	Sphingobacteriales	Sphingobacteriaceae	Sphingobacterium	Sphingobacterium multivorum	P	https://www.ncbi.nlm.nih.gov/datasets/genome/GCA_039511225.1/
GCA_016726045.1	Bacteroidota	Sphingobacteriia	Sphingobacteriales	Sphingobacteriaceae	Sphingobacterium	Sphingobacterium multivorum	P	https://www.ncbi.nlm.nih.gov/datasets/genome/GCA_016726045.1/
GCA_013267375.1	Pseudomonadota	Betaproteobacteria	Burkholderiales	Alcaligenaceae	Achromobacter	Achromobacter denitrificans	P	https://www.ncbi.nlm.nih.gov/datasets/genome/GCA_013267375.1/
GCA_013267395.1	Pseudomonadota	Betaproteobacteria	Burkholderiales	Alcaligenaceae	Achromobacter	Achromobacter denitrificans	P	https://www.ncbi.nlm.nih.gov/datasets/genome/GCA_013267395.1/
GCA_039565655.1	Pseudomonadota	Betaproteobacteria	Burkholderiales	Alcaligenaceae	Achromobacter	Achromobacter denitrificans	P	https://www.ncbi.nlm.nih.gov/datasets/genome/GCA_039565655.1/
GCA_002205315.1	Pseudomonadota	Betaproteobacteria	Burkholderiales	Alcaligenaceae	Achromobacter	Achromobacter denitrificans	P	https://www.ncbi.nlm.nih.gov/datasets/genome/GCA_002205315.1/
GCA_013343095.1	Pseudomonadota	Betaproteobacteria	Burkholderiales	Alcaligenaceae	Achromobacter	Achromobacter denitrificans	P	https://www.ncbi.nlm.nih.gov/datasets/genome/GCA_013343095.1/
GCA_016728825.1	Pseudomonadota	Betaproteobacteria	Burkholderiales	Alcaligenaceae	Achromobacter	Achromobacter xylosoxidans	P	https://www.ncbi.nlm.nih.gov/datasets/genome/GCA_016728825.1/
GCA_022870085.1	Pseudomonadota	Betaproteobacteria	Burkholderiales	Alcaligenaceae	Achromobacter	Achromobacter xylosoxidans	P	https://www.ncbi.nlm.nih.gov/datasets/genome/GCA_022870085.1/
GCA_001457475.1	Pseudomonadota	Betaproteobacteria	Burkholderiales	Alcaligenaceae	Achromobacter	Achromobacter xylosoxidans	P	https://www.ncbi.nlm.nih.gov/datasets/genome/GCA_001457475.1/
GCA_013343135.1	Pseudomonadota	Betaproteobacteria	Burkholderiales	Alcaligenaceae	Achromobacter	Achromobacter xylosoxidans	P	https://www.ncbi.nlm.nih.gov/datasets/genome/GCA_013343135.1/
GCA_033096405.1	Pseudomonadota	Betaproteobacteria	Burkholderiales	Alcaligenaceae	Achromobacter	Achromobacter xylosoxidans	P	https://www.ncbi.nlm.nih.gov/datasets/genome/GCA_033096405.1/
GCA_008632635.1	Pseudomonadota	Gammaproteobacteria	Moraxellales	Moraxellaceae	Acinetobacter	Acinetobacter baumannii	P	https://www.ncbi.nlm.nih.gov/datasets/genome/GCA_008632635.1/
GCA_009035845.1	Pseudomonadota	Gammaproteobacteria	Moraxellales	Moraxellaceae	Acinetobacter	Acinetobacter baumannii	P	https://www.ncbi.nlm.nih.gov/datasets/genome/GCA_009035845.1/
GCA_020911985.1	Pseudomonadota	Gammaproteobacteria	Moraxellales	Moraxellaceae	Acinetobacter	Acinetobacter baumannii	P	https://www.ncbi.nlm.nih.gov/datasets/genome/GCA_020911985.1/
GCA_022870045.1	Pseudomonadota	Gammaproteobacteria	Moraxellales	Moraxellaceae	Acinetobacter	Acinetobacter baumannii	P	https://www.ncbi.nlm.nih.gov/datasets/genome/GCA_022870045.1/
GCA_014672755.1	Pseudomonadota	Gammaproteobacteria	Moraxellales	Moraxellaceae	Acinetobacter	Acinetobacter baumannii	P	https://www.ncbi.nlm.nih.gov/datasets/genome/GCA_014672755.1/
GCA_002055515.1	Pseudomonadota	Gammaproteobacteria	Moraxellales	Moraxellaceae	Acinetobacter	Acinetobacter calcoaceticus	P	https://www.ncbi.nlm.nih.gov/datasets/genome/GCA_002055515.1/
GCA_021165835.1	Pseudomonadota	Gammaproteobacteria	Moraxellales	Moraxellaceae	Acinetobacter	Acinetobacter calcoaceticus	P	https://www.ncbi.nlm.nih.gov/datasets/genome/GCA_021165835.1/
GCA_021165855.1	Pseudomonadota	Gammaproteobacteria	Moraxellales	Moraxellaceae	Acinetobacter	Acinetobacter calcoaceticus	P	https://www.ncbi.nlm.nih.gov/datasets/genome/GCA_021165855.1/
GCA_900520355.1	Pseudomonadota	Gammaproteobacteria	Moraxellales	Moraxellaceae	Acinetobacter	Acinetobacter calcoaceticus	P	https://www.ncbi.nlm.nih.gov/datasets/genome/GCA_900520355.1/
GCA_016939475.1	Pseudomonadota	Gammaproteobacteria	Moraxellales	Moraxellaceae	Acinetobacter	Acinetobacter calcoaceticus	P	https://www.ncbi.nlm.nih.gov/datasets/genome/GCA_016939475.1/
GCA_000967305.2	Pseudomonadota	Betaproteobacteria	Burkholderiales	Alcaligenaceae	Alcaligenes	Alcaligenes faecalis	P	https://www.ncbi.nlm.nih.gov/datasets/genome/GCA_000967305.2/
GCA_002443155.1	Pseudomonadota	Betaproteobacteria	Burkholderiales	Alcaligenaceae	Alcaligenes	Alcaligenes faecalis	P	https://www.ncbi.nlm.nih.gov/datasets/genome/GCA_002443155.1/
GCA_958448115.1	Pseudomonadota	Betaproteobacteria	Burkholderiales	Alcaligenaceae	Alcaligenes	Alcaligenes faecalis	P	https://www.ncbi.nlm.nih.gov/datasets/genome/GCA_958448115.1/

GCA_027595045.1	Pseudomonadota	Betaproteobacteria	Burkholderiales	Alcaligenaceae	Alcaligenes	Alcaligenes faecalis	P	https://www.ncbi.nlm.nih.gov/datasets/genome/GCA_027595045.1/
GCA_010092625.1	Pseudomonadota	Betaproteobacteria	Burkholderiales	Alcaligenaceae	Alcaligenes	Alcaligenes faecalis	P	https://www.ncbi.nlm.nih.gov/datasets/genome/GCA_010092625.1/
GCA_900445995.1	Pseudomonadota	Alphaproteobacteria	Caulobacterales	Caulobacteraceae	Brevundimonas	Brevundimonas diminuta	P	https://www.ncbi.nlm.nih.gov/datasets/genome/GCA_900445995.1/
GCA_038020885.1	Pseudomonadota	Alphaproteobacteria	Caulobacterales	Caulobacteraceae	Brevundimonas	Brevundimonas diminuta	P	https://www.ncbi.nlm.nih.gov/datasets/genome/GCA_038020885.1/
GCA_004102925.1	Pseudomonadota	Alphaproteobacteria	Caulobacterales	Caulobacteraceae	Brevundimonas	Brevundimonas diminuta	P	https://www.ncbi.nlm.nih.gov/datasets/genome/GCA_004102925.1/
GCA_016127655.1	Pseudomonadota	Alphaproteobacteria	Caulobacterales	Caulobacteraceae	Brevundimonas	Brevundimonas diminuta	P	https://www.ncbi.nlm.nih.gov/datasets/genome/GCA_016127655.1/
GCA_034424705.1	Pseudomonadota	Alphaproteobacteria	Caulobacterales	Caulobacteraceae	Brevundimonas	Brevundimonas diminuta	P	https://www.ncbi.nlm.nih.gov/datasets/genome/GCA_034424705.1/
GCA_019047105.1	Pseudomonadota	Gammaproteobacteria	Enterobacterales	Enterobacteriaceae	Enterobacter	Enterobacter cloacae	P	https://www.ncbi.nlm.nih.gov/datasets/genome/GCA_019047105.1/
GCA_000025565.1	Pseudomonadota	Gammaproteobacteria	Enterobacterales	Enterobacteriaceae	Enterobacter	Enterobacter cloacae	P	https://www.ncbi.nlm.nih.gov/datasets/genome/GCA_000025565.1/
GCA_018140965.1	Pseudomonadota	Gammaproteobacteria	Enterobacterales	Enterobacteriaceae	Enterobacter	Enterobacter cloacae	P	https://www.ncbi.nlm.nih.gov/datasets/genome/GCA_018140965.1/
GCA_003019925.1	Pseudomonadota	Gammaproteobacteria	Enterobacterales	Enterobacteriaceae	Pluralibacter	Pluralibacter gergoviae	P	https://www.ncbi.nlm.nih.gov/datasets/genome/GCA_003019925.1/
GCA_000757785.1	Pseudomonadota	Gammaproteobacteria	Enterobacterales	Enterobacteriaceae	Pluralibacter	Pluralibacter gergoviae	P	https://www.ncbi.nlm.nih.gov/datasets/genome/GCA_000757785.1/
GCA_902387975.1	Pseudomonadota	Gammaproteobacteria	Enterobacterales	Enterobacteriaceae	Pluralibacter	Pluralibacter gergoviae	P	https://www.ncbi.nlm.nih.gov/datasets/genome/GCA_902387975.1/
GCA_001598855.1	Pseudomonadota	Gammaproteobacteria	Enterobacterales	Enterobacteriaceae	Pluralibacter	Pluralibacter gergoviae	P	https://www.ncbi.nlm.nih.gov/datasets/genome/GCA_001598855.1/
GCA_902729455.1	Pseudomonadota	Gammaproteobacteria	Enterobacterales	Enterobacteriaceae	Pluralibacter	Pluralibacter gergoviae	P	https://www.ncbi.nlm.nih.gov/datasets/genome/GCA_902729455.1/
GCA_009914515.1	Actinomycetota	Actinomycetes	Mycobacteriales	Gordoniaceae	Gordonia	Gordonia amarae	P	https://www.ncbi.nlm.nih.gov/datasets/genome/GCA_009914515.1/
GCA_009914495.1	Actinomycetota	Actinomycetes	Mycobacteriales	Gordoniaceae	Gordonia	Gordonia amarae	P	https://www.ncbi.nlm.nih.gov/datasets/genome/GCA_009914495.1/
GCA_025268675.1	Actinomycetota	Actinomycetes	Mycobacteriales	Gordoniaceae	Gordonia	Gordonia amarae	P	https://www.ncbi.nlm.nih.gov/datasets/genome/GCA_025268675.1/
GCA_000241345.2	Actinomycetota	Actinomycetes	Mycobacteriales	Gordoniaceae	Gordonia	Gordonia amarae	P	https://www.ncbi.nlm.nih.gov/datasets/genome/GCA_000241345.2/
GCA_024809495.1	Actinomycetota	Actinomycetes	Mycobacteriales	Gordoniaceae	Gordonia	Gordonia amarae	P	https://www.ncbi.nlm.nih.gov/datasets/genome/GCA_024809495.1/
GCA_030536535.1	Pseudomonadota	Gammaproteobacteria	Enterobacterales	Morganellaceae	Proteus	Proteus sp. (in: enterobacteria)	P	https://www.ncbi.nlm.nih.gov/datasets/genome/GCA_030536535.1/
GCA_030146765.1	Pseudomonadota	Gammaproteobacteria	Enterobacterales	Morganellaceae	Proteus	Proteus sp. (in: enterobacteria)	P	https://www.ncbi.nlm.nih.gov/datasets/genome/GCA_030146765.1/
GCA_030147105.1	Pseudomonadota	Gammaproteobacteria	Enterobacterales	Morganellaceae	Proteus	Proteus sp. (in: enterobacteria)	P	https://www.ncbi.nlm.nih.gov/datasets/genome/GCA_030147105.1/
GCA_003523365.1	Pseudomonadota	Gammaproteobacteria	Enterobacterales	Morganellaceae	Proteus	Proteus sp. (in: enterobacteria)	P	https://www.ncbi.nlm.nih.gov/datasets/genome/GCA_003523365.1/
GCA_036010585.1	Actinomycetota	Actinomycetes	Kitasatosporales	Streptomycetaceae	Streptomyces	Streptomyces microflavus	P	https://www.ncbi.nlm.nih.gov/datasets/genome/GCA_036010585.1/
GCA_036237355.1	Actinomycetota	Actinomycetes	Kitasatosporales	Streptomycetaceae	Streptomyces	Streptomyces microflavus	P	https://www.ncbi.nlm.nih.gov/datasets/genome/GCA_036237355.1/
GCA_000385945.1	Actinomycetota	Actinomycetes	Kitasatosporales	Streptomycetaceae	Streptomyces	Streptomyces microflavus	P	https://www.ncbi.nlm.nih.gov/datasets/genome/GCA_000385945.1/
GCA_036227025.1	Actinomycetota	Actinomycetes	Kitasatosporales	Streptomycetaceae	Streptomyces	Streptomyces microflavus	P	https://www.ncbi.nlm.nih.gov/datasets/genome/GCA_036227025.1/
GCA_013364315.1	Actinomycetota	Actinomycetes	Kitasatosporales	Streptomycetaceae	Streptomyces	Streptomyces microflavus	P	https://www.ncbi.nlm.nih.gov/datasets/genome/GCA_013364315.1/
GCA_003702755.1	Pseudomonadota	Gammaproteobacteria	Pseudomonadales	Pseudomonadaceae	Pseudomonas	Pseudomonas syringae group genomosp. 3	N	https://www.ncbi.nlm.nih.gov/datasets/genome/GCA_003702755.1/
GCA_013321415.1	Pseudomonadota	Alphaproteobacteria	Hyphomicrobiales	Rhizobiaceae	Rhizobium	Rhizobium rhizogenes	N	https://www.ncbi.nlm.nih.gov/datasets/genome/GCA_013321415.1/
GCA_028555185.1	Pseudomonadota	Gammaproteobacteria	Enterobacterales	Erwiniaceae	Pantoea	Pantoea ananatis	N	https://www.ncbi.nlm.nih.gov/datasets/genome/GCA_028555185.1/
GCA_003699785.1	Pseudomonadota	Gammaproteobacteria	Pseudomonadales	Pseudomonadaceae	Pseudomonas	Pseudomonas coronafaciens	N	https://www.ncbi.nlm.nih.gov/datasets/genome/GCA_003699785.1/
GCA_017656535.1	Pseudomonadota	Gammaproteobacteria	Enterobacterales	Pectobacteriaceae	Dickeya	Dickeya fangzhongdai	N	https://www.ncbi.nlm.nih.gov/datasets/genome/GCA_017656535.1/
GCA_000807875.2	Pseudomonadota	Gammaproteobacteria	Lysobacterales	Lysobacteraceae	Xanthomonas	Xanthomonas phaseoli	N	https://www.ncbi.nlm.nih.gov/datasets/genome/GCA_000807875.2/
GCA_029086895.1	Pseudomonadota	Gammaproteobacteria	Enterobacterales	Erwiniaceae	Pantoea	Pantoea ananatis	N	https://www.ncbi.nlm.nih.gov/datasets/genome/GCA_029086895.1/
GCA_013608115.1	Pseudomonadota	Betaproteobacteria	Burkholderiales	Burkholderiaceae	Burkholderia	Burkholderia gladioli	N	https://www.ncbi.nlm.nih.gov/datasets/genome/GCA_013608115.1/
GCA_032190835.1	Pseudomonadota	Gammaproteobacteria	Enterobacterales	Enterobacteriaceae	Enterobacter	Enterobacter cancerogenus	N	https://www.ncbi.nlm.nih.gov/datasets/genome/GCA_032190835.1/
GCA_003416255.1	Pseudomonadota	Gammaproteobacteria	Pseudomonadales	Pseudomonadaceae	Pseudomonas	Pseudomonas coronafaciens	N	https://www.ncbi.nlm.nih.gov/datasets/genome/GCA_003416255.1/
GCA_003700905.1	Pseudomonadota	Gammaproteobacteria	Pseudomonadales	Pseudomonadaceae	Pseudomonas	Pseudomonas coronafaciens	N	https://www.ncbi.nlm.nih.gov/datasets/genome/GCA_003700905.1/
GCA_032198945.1	Pseudomonadota	Gammaproteobacteria	Enterobacterales	Enterobacteriaceae	Enterobacter	Enterobacter cancerogenus	N	https://www.ncbi.nlm.nih.gov/datasets/genome/GCA_032198945.1/
GCA_022385255.1	Pseudomonadota	Gammaproteobacteria	Enterobacterales	Pectobacteriaceae	Dickeya	Dickeya fangzhongdai	N	https://www.ncbi.nlm.nih.gov/datasets/genome/GCA_022385255.1/
GCA_016803175.1	Pseudomonadota	Gammaproteobacteria	Pseudomonadales	Pseudomonadaceae	Pseudomonas	Pseudomonas syringae group genomosp. 3	N	https://www.ncbi.nlm.nih.gov/datasets/genome/GCA_016803175.1/
GCA_029081345.1	Pseudomonadota	Gammaproteobacteria	Enterobacterales	Erwiniaceae	Pantoea	Pantoea ananatis	N	https://www.ncbi.nlm.nih.gov/datasets/genome/GCA_029081345.1/
GCA_902829525.1	Pseudomonadota	Betaproteobacteria	Burkholderiales	Burkholderiaceae	Burkholderia	Burkholderia gladioli	N	https://www.ncbi.nlm.nih.gov/datasets/genome/GCA_902829525.1/
GCA_900581015.1	Pseudomonadota	Gammaproteobacteria	Pseudomonadales	Pseudomonadaceae	Pseudomonas	Pseudomonas viridiflava	N	https://www.ncbi.nlm.nih.gov/datasets/genome/GCA_900581015.1/
GCA_000981885.1	Pseudomonadota	Betaproteobacteria	Burkholderiales	Burkholderiaceae	Burkholderia	Burkholderia gladioli	N	https://www.ncbi.nlm.nih.gov/datasets/genome/GCA_000981885.1/
GCA_003699955.1	Pseudomonadota	Gammaproteobacteria	Pseudomonadales	Pseudomonadaceae	Pseudomonas	Pseudomonas coronafaciens	N	https://www.ncbi.nlm.nih.gov/datasets/genome/GCA_003699955.1/
GCA_900580895.1	Pseudomonadota	Gammaproteobacteria	Pseudomonadales	Pseudomonadaceae	Pseudomonas	Pseudomonas viridiflava	N	https://www.ncbi.nlm.nih.gov/datasets/genome/GCA_900580895.1/
GCA_034809025.1	Pseudomonadota	Gammaproteobacteria	Enterobacterales	Erwiniaceae	Pantoea	Pantoea ananatis	N	https://www.ncbi.nlm.nih.gov/datasets/genome/GCA_034809025.1/
GCA_019042515.1	Pseudomonadota	Betaproteobacteria	Burkholderiales	Burkholderiaceae	Burkholderia	Burkholderia gladioli	N	https://www.ncbi.nlm.nih.gov/datasets/genome/GCA_019042515.1/
GCA_032916665.1	Actinomycetota	Actinomycetes	Micrococcales	Microbacteriaceae	Clavibacter	Clavibacter michiganensis	N	https://www.ncbi.nlm.nih.gov/datasets/genome/GCA_032916665.1/
GCA_029081695.1	Pseudomonadota	Gammaproteobacteria	Enterobacterales	Erwiniaceae	Pantoea	Pantoea ananatis	N	https://www.ncbi.nlm.nih.gov/datasets/genome/GCA_029081695.1/
GCA_021605965.1	Pseudomonadota	Gammaproteobacteria	Pseudomonadales	Pseudomonadaceae	Pseudomonas	Pseudomonas syringae	N	https://www.ncbi.nlm.nih.gov/datasets/genome/GCA_021605965.1/
GCA_003590625.2	Pseudomonadota	Betaproteobacteria	Burkholderiales	Burkholderiaceae	Ralstonia	Ralstonia solanacearum	N	https://www.ncbi.nlm.nih.gov/datasets/genome/GCA_003590625.2/
GCA_032655185.1	Actinomycetota	Actinomycetes	Micrococcales	Microbacteriaceae	Clavibacter	Clavibacter michiganensis	N	https://www.ncbi.nlm.nih.gov/datasets/genome/GCA_032655185.1/

GCA_020813055.1	Pseudomonadota	Gammaproteobacteria	Lysobacterales	Lysobacteraceae	Xanthomonas	Xanthomonas campestris	N	https://www.ncbi.nlm.nih.gov/datasets/genome/GCA_020813055.1/
GCA_000155995.1	Pseudomonadota	Gammaproteobacteria	Enterobacterales	Enterobacteriaceae	Enterobacter	Enterobacter cancerogenus	N	https://www.ncbi.nlm.nih.gov/datasets/genome/GCA_000155995.1/
GCA_029086265.1	Pseudomonadota	Gammaproteobacteria	Enterobacterales	Erwiniaceae	Pantoea	Pantoea ananatis	N	https://www.ncbi.nlm.nih.gov/datasets/genome/GCA_029086265.1/
GCA_010677205.1	Pseudomonadota	Gammaproteobacteria	Enterobacterales	Erwiniaceae	Pantoea	Pantoea ananatis	N	https://www.ncbi.nlm.nih.gov/datasets/genome/GCA_010677205.1/
GCA_000172895.1	Pseudomonadota	Gammaproteobacteria	Pseudomonadales	Pseudomonadaceae	Pseudomonas	Pseudomonas syringae group genomosp. 3	N	https://www.ncbi.nlm.nih.gov/datasets/genome/GCA_000172895.1/
GCA_002959435.1	Pseudomonadota	Gammaproteobacteria	Enterobacterales	Erwiniaceae	Pantoea	Pantoea ananatis	N	https://www.ncbi.nlm.nih.gov/datasets/genome/GCA_002959435.1/
GCA_001475565.1	Pseudomonadota	Gammaproteobacteria	Enterobacterales	Enterobacteriaceae	Enterobacter	Enterobacter cancerogenus	N	https://www.ncbi.nlm.nih.gov/datasets/genome/GCA_001475565.1/
GCA_001475535.1	Pseudomonadota	Gammaproteobacteria	Enterobacterales	Enterobacteriaceae	Enterobacter	Enterobacter cancerogenus	N	https://www.ncbi.nlm.nih.gov/datasets/genome/GCA_001475535.1/
GCA_017921235.1	Pseudomonadota	Gammaproteobacteria	Enterobacterales	Erwiniaceae	Pantoea	Pantoea ananatis	N	https://www.ncbi.nlm.nih.gov/datasets/genome/GCA_017921235.1/
GCA_029684855.1	Pseudomonadota	Gammaproteobacteria	Enterobacterales	Enterobacteriaceae	Enterobacter	Enterobacter cancerogenus	N	https://www.ncbi.nlm.nih.gov/datasets/genome/GCA_029684855.1/
GCA_017920195.1	Pseudomonadota	Gammaproteobacteria	Enterobacterales	Erwiniaceae	Pantoea	Pantoea ananatis	N	https://www.ncbi.nlm.nih.gov/datasets/genome/GCA_017920195.1/
GCA_001644805.1	Pseudomonadota	Betaproteobacteria	Burkholderiales	Burkholderiaceae	Ralstonia	Ralstonia solanacearum	N	https://www.ncbi.nlm.nih.gov/datasets/genome/GCA_001644805.1/
GCA_019880465.1	Pseudomonadota	Gammaproteobacteria	Enterobacterales	Enterobacteriaceae	Enterobacter	Enterobacter cancerogenus	N	https://www.ncbi.nlm.nih.gov/datasets/genome/GCA_019880465.1/
GCA_001400345.1	Pseudomonadota	Gammaproteobacteria	Pseudomonadales	Pseudomonadaceae	Pseudomonas	Pseudomonas coronafaciens	N	https://www.ncbi.nlm.nih.gov/datasets/genome/GCA_001400345.1/
GCA_001293975.1	Pseudomonadota	Gammaproteobacteria	Pseudomonadales	Pseudomonadaceae	Pseudomonas	Pseudomonas amygdali	N	https://www.ncbi.nlm.nih.gov/datasets/genome/GCA_001293975.1/
GCA_000710015.2	Pseudomonadota	Gammaproteobacteria	Enterobacterales	Erwiniaceae	Pantoea	Pantoea ananatis	N	https://www.ncbi.nlm.nih.gov/datasets/genome/GCA_000710015.2/
GCA_003416545.1	Pseudomonadota	Gammaproteobacteria	Pseudomonadales	Pseudomonadaceae	Pseudomonas	Pseudomonas coronafaciens	N	https://www.ncbi.nlm.nih.gov/datasets/genome/GCA_003416545.1/
GCA_003416565.1	Pseudomonadota	Gammaproteobacteria	Pseudomonadales	Pseudomonadaceae	Pseudomonas	Pseudomonas coronafaciens	N	https://www.ncbi.nlm.nih.gov/datasets/genome/GCA_003416565.1/
GCA_015865025.1	Pseudomonadota	Gammaproteobacteria	Pseudomonadales	Pseudomonadaceae	Pseudomonas	Pseudomonas syringae group genomosp. 3	N	https://www.ncbi.nlm.nih.gov/datasets/genome/GCA_015865025.1/
GCA_003415895.1	Pseudomonadota	Gammaproteobacteria	Pseudomonadales	Pseudomonadaceae	Pseudomonas	Pseudomonas coronafaciens	N	https://www.ncbi.nlm.nih.gov/datasets/genome/GCA_003415895.1/
GCA_003416575.1	Pseudomonadota	Gammaproteobacteria	Pseudomonadales	Pseudomonadaceae	Pseudomonas	Pseudomonas coronafaciens	N	https://www.ncbi.nlm.nih.gov/datasets/genome/GCA_003416575.1/
GCA_003416425.1	Pseudomonadota	Gammaproteobacteria	Pseudomonadales	Pseudomonadaceae	Pseudomonas	Pseudomonas coronafaciens	N	https://www.ncbi.nlm.nih.gov/datasets/genome/GCA_003416425.1/
GCA_003701555.1	Pseudomonadota	Gammaproteobacteria	Pseudomonadales	Pseudomonadaceae	Pseudomonas	Pseudomonas coronafaciens	N	https://www.ncbi.nlm.nih.gov/datasets/genome/GCA_003701555.1/
GCA_003416245.1	Pseudomonadota	Gammaproteobacteria	Pseudomonadales	Pseudomonadaceae	Pseudomonas	Pseudomonas coronafaciens	N	https://www.ncbi.nlm.nih.gov/datasets/genome/GCA_003416245.1/
GCA_013321905.1	Pseudomonadota	Alphaproteobacteria	Hyphomicrobiales	Rhizobiaceae	Rhizobium	Rhizobium rhizogenes	N	https://www.ncbi.nlm.nih.gov/datasets/genome/GCA_013321905.1/
GCA_024919385.1	Actinomycetota	Actinomycetes	Micrococcales	Microbacteriaceae	Curtobacterium	Curtobacterium flaccumfaciens	N	https://www.ncbi.nlm.nih.gov/datasets/genome/GCA_024919385.1/
GCA_003416035.1	Pseudomonadota	Gammaproteobacteria	Pseudomonadales	Pseudomonadaceae	Pseudomonas	Pseudomonas coronafaciens	N	https://www.ncbi.nlm.nih.gov/datasets/genome/GCA_003416035.1/
GCA_003416455.1	Pseudomonadota	Gammaproteobacteria	Pseudomonadales	Pseudomonadaceae	Pseudomonas	Pseudomonas coronafaciens	N	https://www.ncbi.nlm.nih.gov/datasets/genome/GCA_003416455.1/
GCA_003701785.1	Pseudomonadota	Gammaproteobacteria	Pseudomonadales	Pseudomonadaceae	Pseudomonas	Pseudomonas coronafaciens	N	https://www.ncbi.nlm.nih.gov/datasets/genome/GCA_003701785.1/
GCA_003415685.1	Pseudomonadota	Gammaproteobacteria	Pseudomonadales	Pseudomonadaceae	Pseudomonas	Pseudomonas coronafaciens	N	https://www.ncbi.nlm.nih.gov/datasets/genome/GCA_003415685.1/
GCA_003416355.1	Pseudomonadota	Gammaproteobacteria	Pseudomonadales	Pseudomonadaceae	Pseudomonas	Pseudomonas coronafaciens	N	https://www.ncbi.nlm.nih.gov/datasets/genome/GCA_003416355.1/
GCA_021606145.1	Pseudomonadota	Gammaproteobacteria	Pseudomonadales	Pseudomonadaceae	Pseudomonas	Pseudomonas syringae	N	https://www.ncbi.nlm.nih.gov/datasets/genome/GCA_021606145.1/
GCA_003416495.1	Pseudomonadota	Gammaproteobacteria	Pseudomonadales	Pseudomonadaceae	Pseudomonas	Pseudomonas coronafaciens	N	https://www.ncbi.nlm.nih.gov/datasets/genome/GCA_003416495.1/
GCA_003415975.1	Pseudomonadota	Gammaproteobacteria	Pseudomonadales	Pseudomonadaceae	Pseudomonas	Pseudomonas coronafaciens	N	https://www.ncbi.nlm.nih.gov/datasets/genome/GCA_003415975.1/
GCA_021609365.1	Pseudomonadota	Gammaproteobacteria	Pseudomonadales	Pseudomonadaceae	Pseudomonas	Pseudomonas marginalis	N	https://www.ncbi.nlm.nih.gov/datasets/genome/GCA_021609365.1/
GCA_041379965.2	Pseudomonadota	Gammaproteobacteria	Pseudomonadales	Pseudomonadaceae	Pseudomonas	Pseudomonas syringae group genomosp. 3	N	https://www.ncbi.nlm.nih.gov/datasets/genome/GCA_041379965.2/
GCA_003700475.1	Pseudomonadota	Gammaproteobacteria	Pseudomonadales	Pseudomonadaceae	Pseudomonas	Pseudomonas coronafaciens	N	https://www.ncbi.nlm.nih.gov/datasets/genome/GCA_003700475.1/
GCA_021777455.1	Pseudomonadota	Gammaproteobacteria	Lysobacterales	Lysobacteraceae	Xanthomonas	Xanthomonas campestris	N	https://www.ncbi.nlm.nih.gov/datasets/genome/GCA_021777455.1/
GCA_003416405.1	Pseudomonadota	Gammaproteobacteria	Pseudomonadales	Pseudomonadaceae	Pseudomonas	Pseudomonas coronafaciens	N	https://www.ncbi.nlm.nih.gov/datasets/genome/GCA_003416405.1/
GCA_003416505.1	Pseudomonadota	Gammaproteobacteria	Pseudomonadales	Pseudomonadaceae	Pseudomonas	Pseudomonas coronafaciens	N	https://www.ncbi.nlm.nih.gov/datasets/genome/GCA_003416505.1/
GCA_021864555.1	Pseudomonadota	Gammaproteobacteria	Lysobacterales	Lysobacteraceae	Xanthomonas	Xanthomonas campestris	N	https://www.ncbi.nlm.nih.gov/datasets/genome/GCA_021864555.1/
GCA_019689265.1	Pseudomonadota	Gammaproteobacteria	Pseudomonadales	Pseudomonadaceae	Pseudomonas	Pseudomonas syringae group genomosp. 3	N	https://www.ncbi.nlm.nih.gov/datasets/genome/GCA_019689265.1/
GCA_001400895.1	Pseudomonadota	Gammaproteobacteria	Pseudomonadales	Pseudomonadaceae	Pseudomonas	Pseudomonas coronafaciens	N	https://www.ncbi.nlm.nih.gov/datasets/genome/GCA_001400895.1/
GCA_023701545.1	Pseudomonadota	Gammaproteobacteria	Pseudomonadales	Pseudomonadaceae	Pseudomonas	Pseudomonas marginalis	N	https://www.ncbi.nlm.nih.gov/datasets/genome/GCA_023701545.1/
GCA_003416385.1	Pseudomonadota	Gammaproteobacteria	Pseudomonadales	Pseudomonadaceae	Pseudomonas	Pseudomonas coronafaciens	N	https://www.ncbi.nlm.nih.gov/datasets/genome/GCA_003416385.1/
GCA_019688775.1	Pseudomonadota	Gammaproteobacteria	Pseudomonadales	Pseudomonadaceae	Pseudomonas	Pseudomonas syringae group genomosp. 3	N	https://www.ncbi.nlm.nih.gov/datasets/genome/GCA_019688775.1/
GCA_003416665.1	Pseudomonadota	Gammaproteobacteria	Pseudomonadales	Pseudomonadaceae	Pseudomonas	Pseudomonas coronafaciens	N	https://www.ncbi.nlm.nih.gov/datasets/genome/GCA_003416665.1/
GCA_016307745.1	Pseudomonadota	Gammaproteobacteria	Pseudomonadales	Pseudomonadaceae	Pseudomonas	Pseudomonas syringae	N	https://www.ncbi.nlm.nih.gov/datasets/genome/GCA_016307745.1/
GCA_021459985.1	Pseudomonadota	Gammaproteobacteria	Lysobacterales	Lysobacteraceae	Xanthomonas	Xanthomonas campestris	N	https://www.ncbi.nlm.nih.gov/datasets/genome/GCA_021459985.1/
GCA_002850575.1	Pseudomonadota	Gammaproteobacteria	Enterobacterales	Enterobacteriaceae	Enterobacter	Enterobacter cancerogenus	N	https://www.ncbi.nlm.nih.gov/datasets/genome/GCA_002850575.1/
GCA_003416655.1	Pseudomonadota	Gammaproteobacteria	Pseudomonadales	Pseudomonadaceae	Pseudomonas	Pseudomonas coronafaciens	N	https://www.ncbi.nlm.nih.gov/datasets/genome/GCA_003416655.1/
GCA_001401235.1	Pseudomonadota	Gammaproteobacteria	Pseudomonadales	Pseudomonadaceae	Pseudomonas	Pseudomonas coronafaciens	N	https://www.ncbi.nlm.nih.gov/datasets/genome/GCA_001401235.1/
GCA_001400435.1	Pseudomonadota	Gammaproteobacteria	Pseudomonadales	Pseudomonadaceae	Pseudomonas	Pseudomonas amygdali	N	https://www.ncbi.nlm.nih.gov/datasets/genome/GCA_001400435.1/
GCA_021864855.1	Pseudomonadota	Gammaproteobacteria	Lysobacterales	Lysobacteraceae	Xanthomonas	Xanthomonas campestris	N	https://www.ncbi.nlm.nih.gov/datasets/genome/GCA_021864855.1/
GCA_023278345.1	Pseudomonadota	Gammaproteobacteria	Lysobacterales	Lysobacteraceae	Xanthomonas	Xanthomonas campestris	N	https://www.ncbi.nlm.nih.gov/datasets/genome/GCA_023278345.1/
GCA_019689235.1	Pseudomonadota	Gammaproteobacteria	Pseudomonadales	Pseudomonadaceae	Pseudomonas	Pseudomonas syringae group genomosp. 3	N	https://www.ncbi.nlm.nih.gov/datasets/genome/GCA_019689235.1/

GCA_028749765.1	Pseudomonadota	Gammaproteobacteria	Lysobacterales	Lysobacteraceae	Xanthomonas	Xanthomonas campestris	N	https://www.ncbi.nlm.nih.gov/datasets/genome/GCA_028749765.1/
GCA_021649995.1	Pseudomonadota	Gammaproteobacteria	Enterobacterales	Enterobacteriaceae	Enterobacter	Enterobacter cancerogenus	N	https://www.ncbi.nlm.nih.gov/datasets/genome/GCA_021649995.1/
GCA_001535815.1	Pseudomonadota	Gammaproteobacteria	Pseudomonadales	Pseudomonadaceae	Pseudomonas	Pseudomonas savastanoi	N	https://www.ncbi.nlm.nih.gov/datasets/genome/GCA_001535815.1/
GCA_019689375.1	Pseudomonadota	Gammaproteobacteria	Pseudomonadales	Pseudomonadaceae	Pseudomonas	Pseudomonas syringae group genomosp. 3	N	https://www.ncbi.nlm.nih.gov/datasets/genome/GCA_019689375.1/
GCA_002981875.1	Pseudomonadota	Betaproteobacteria	Burkholderiales	Burkholderiaceae	Burkholderia	Burkholderia gladioli	N	https://www.ncbi.nlm.nih.gov/datasets/genome/GCA_002981875.1/
GCA_019688895.1	Pseudomonadota	Gammaproteobacteria	Pseudomonadales	Pseudomonadaceae	Pseudomonas	Pseudomonas syringae group genomosp. 3	N	https://www.ncbi.nlm.nih.gov/datasets/genome/GCA_019688895.1/
GCA_013322025.1	Pseudomonadota	Alphaproteobacteria	Hyphomicrobiales	Rhizobiaceae	Rhizobium	Rhizobium rhizogenes	N	https://www.ncbi.nlm.nih.gov/datasets/genome/GCA_013322025.1/
GCA_013321135.1	Pseudomonadota	Alphaproteobacteria	Hyphomicrobiales	Rhizobiaceae	Rhizobium	Rhizobium rhizogenes	N	https://www.ncbi.nlm.nih.gov/datasets/genome/GCA_013321135.1/
GCA_000177455.1	Pseudomonadota	Gammaproteobacteria	Pseudomonadales	Pseudomonadaceae	Pseudomonas	Pseudomonas syringae group genomosp. 3	N	https://www.ncbi.nlm.nih.gov/datasets/genome/GCA_000177455.1/
GCA_019688965.1	Pseudomonadota	Gammaproteobacteria	Pseudomonadales	Pseudomonadaceae	Pseudomonas	Pseudomonas syringae group genomosp. 3	N	https://www.ncbi.nlm.nih.gov/datasets/genome/GCA_019688965.1/
GCA_019689355.1	Pseudomonadota	Gammaproteobacteria	Pseudomonadales	Pseudomonadaceae	Pseudomonas	Pseudomonas syringae group genomosp. 3	N	https://www.ncbi.nlm.nih.gov/datasets/genome/GCA_019689355.1/
GCA_964208085.1	Pseudomonadota	Gammaproteobacteria	Enterobacterales	Enterobacteriaceae	Enterobacter	Enterobacter cancerogenus	N	https://www.ncbi.nlm.nih.gov/datasets/genome/GCA_964208085.1/
GCA_949769235.1	Pseudomonadota	Gammaproteobacteria	Pseudomonadales	Pseudomonadaceae	Pseudomonas	Pseudomonas syringae group genomosp. 3	N	https://www.ncbi.nlm.nih.gov/datasets/genome/GCA_949769235.1/
GCA_003800545.1	Pseudomonadota	Gammaproteobacteria	Enterobacterales	Pectobacteriaceae	Pectobacterium	Pectobacterium atrosepticum	N	https://www.ncbi.nlm.nih.gov/datasets/genome/GCA_003800545.1/
GCA_021459805.1	Pseudomonadota	Gammaproteobacteria	Lysobacterales	Lysobacteraceae	Xylella	Xylella fastidiosa	N	https://www.ncbi.nlm.nih.gov/datasets/genome/GCA_021459805.1/
GCA_013321855.1	Pseudomonadota	Alphaproteobacteria	Hyphomicrobiales	Rhizobiaceae	Rhizobium	Rhizobium rhizogenes	N	https://www.ncbi.nlm.nih.gov/datasets/genome/GCA_013321855.1/
GCA_003415955.1	Pseudomonadota	Gammaproteobacteria	Pseudomonadales	Pseudomonadaceae	Pseudomonas	Pseudomonas coronafaciens	N	https://www.ncbi.nlm.nih.gov/datasets/genome/GCA_003415955.1/
GCA_013321045.1	Pseudomonadota	Alphaproteobacteria	Hyphomicrobiales	Rhizobiaceae	Rhizobium	Rhizobium rhizogenes	N	https://www.ncbi.nlm.nih.gov/datasets/genome/GCA_013321045.1/
GCA_000567985.1	Pseudomonadota	Gammaproteobacteria	Lysobacterales	Lysobacteraceae	Xylella	Xylella fastidiosa	N	https://www.ncbi.nlm.nih.gov/datasets/genome/GCA_000567985.1/
GCA_021864635.1	Pseudomonadota	Gammaproteobacteria	Lysobacterales	Lysobacteraceae	Xanthomonas	Xanthomonas campestris	N	https://www.ncbi.nlm.nih.gov/datasets/genome/GCA_021864635.1/
GCA_016307765.1	Pseudomonadota	Gammaproteobacteria	Pseudomonadales	Pseudomonadaceae	Pseudomonas	Pseudomonas syringae	N	https://www.ncbi.nlm.nih.gov/datasets/genome/GCA_016307765.1/
GCA_007002795.1	Pseudomonadota	Alphaproteobacteria	Hyphomicrobiales	Rhizobiaceae	Rhizobium	Rhizobium rhizogenes	N	https://www.ncbi.nlm.nih.gov/datasets/genome/GCA_007002795.1/
GCA_019689015.1	Pseudomonadota	Gammaproteobacteria	Pseudomonadales	Pseudomonadaceae	Pseudomonas	Pseudomonas syringae group genomosp. 3	N	https://www.ncbi.nlm.nih.gov/datasets/genome/GCA_019689015.1/
GCA_905220785.1	Pseudomonadota	Gammaproteobacteria	Lysobacterales	Lysobacteraceae	Xanthomonas	Xanthomonas arboricola	N	https://www.ncbi.nlm.nih.gov/datasets/genome/GCA_905220785.1/
GCA_009648915.1	Pseudomonadota	Gammaproteobacteria	Enterobacterales	Enterobacteriaceae	Enterobacter	Enterobacter cancerogenus	N	https://www.ncbi.nlm.nih.gov/datasets/genome/GCA_009648915.1/
GCA_021864915.1	Pseudomonadota	Gammaproteobacteria	Lysobacterales	Lysobacteraceae	Xanthomonas	Xanthomonas campestris	N	https://www.ncbi.nlm.nih.gov/datasets/genome/GCA_021864915.1/
GCA_002019075.1	Pseudomonadota	Gammaproteobacteria	Lysobacterales	Lysobacteraceae	Xanthomonas	Xanthomonas campestris	N	https://www.ncbi.nlm.nih.gov/datasets/genome/GCA_002019075.1/
GCA_902830195.1	Pseudomonadota	Betaproteobacteria	Burkholderiales	Burkholderiaceae	Burkholderia	Burkholderia gladioli	N	https://www.ncbi.nlm.nih.gov/datasets/genome/GCA_902830195.1/
GCA_013322065.1	Pseudomonadota	Alphaproteobacteria	Hyphomicrobiales	Rhizobiaceae	Rhizobium	Rhizobium rhizogenes	N	https://www.ncbi.nlm.nih.gov/datasets/genome/GCA_013322065.1/
GCA_000355635.2	Pseudomonadota	Gammaproteobacteria	Lysobacterales	Lysobacteraceae	Xanthomonas	Xanthomonas arboricola	N	https://www.ncbi.nlm.nih.gov/datasets/genome/GCA_000355635.2/
GCA_005233655.1	Pseudomonadota	Gammaproteobacteria	Enterobacterales	Enterobacteriaceae	Enterobacter	Enterobacter cancerogenus	N	https://www.ncbi.nlm.nih.gov/datasets/genome/GCA_005233655.1/
GCA_002981405.1	Pseudomonadota	Betaproteobacteria	Burkholderiales	Burkholderiaceae	Burkholderia	Burkholderia gladioli	N	https://www.ncbi.nlm.nih.gov/datasets/genome/GCA_002981405.1/
GCA_003700445.1	Pseudomonadota	Gammaproteobacteria	Pseudomonadales	Pseudomonadaceae	Pseudomonas	Pseudomonas savastanoi	N	https://www.ncbi.nlm.nih.gov/datasets/genome/GCA_003700445.1/
GCA_013321175.1	Pseudomonadota	Alphaproteobacteria	Hyphomicrobiales	Rhizobiaceae	Rhizobium	Rhizobium rhizogenes	N	https://www.ncbi.nlm.nih.gov/datasets/genome/GCA_013321175.1/
GCA_019041335.1	Pseudomonadota	Betaproteobacteria	Burkholderiales	Burkholderiaceae	Burkholderia	Burkholderia gladioli	N	https://www.ncbi.nlm.nih.gov/datasets/genome/GCA_019041335.1/
GCA_000698825.1	Pseudomonadota	Gammaproteobacteria	Lysobacterales	Lysobacteraceae	Xylella	Xylella fastidiosa	N	https://www.ncbi.nlm.nih.gov/datasets/genome/GCA_000698825.1/
GCA_002939845.1	Pseudomonadota	Gammaproteobacteria	Lysobacterales	Lysobacteraceae	Xanthomonas	Xanthomonas arboricola	N	https://www.ncbi.nlm.nih.gov/datasets/genome/GCA_002939845.1/
GCA_013321295.1	Pseudomonadota	Alphaproteobacteria	Hyphomicrobiales	Rhizobiaceae	Rhizobium	Rhizobium rhizogenes	N	https://www.ncbi.nlm.nih.gov/datasets/genome/GCA_013321295.1/
GCA_001400395.1	Pseudomonadota	Gammaproteobacteria	Pseudomonadales	Pseudomonadaceae	Pseudomonas	Pseudomonas amygdali	N	https://www.ncbi.nlm.nih.gov/datasets/genome/GCA_001400395.1/
GCA_026891515.1	Pseudomonadota	Gammaproteobacteria	Enterobacterales	Pectobacteriaceae	Dickeya	Dickeya solani	N	https://www.ncbi.nlm.nih.gov/datasets/genome/GCA_026891515.1/
GCA_021606345.1	Pseudomonadota	Gammaproteobacteria	Pseudomonadales	Pseudomonadaceae	Pseudomonas	Pseudomonas syringae	N	https://www.ncbi.nlm.nih.gov/datasets/genome/GCA_021606345.1/
GCA_904425455.1	Pseudomonadota	Gammaproteobacteria	Enterobacterales	Enterobacteriaceae	Enterobacter	Enterobacter cancerogenus	N	https://www.ncbi.nlm.nih.gov/datasets/genome/GCA_904425455.1/
GCA_014841015.1	Pseudomonadota	Gammaproteobacteria	Lysobacterales	Lysobacteraceae	Xanthomonas	Xanthomonas campestris	N	https://www.ncbi.nlm.nih.gov/datasets/genome/GCA_014841015.1/
GCA_024413095.1	Pseudomonadota	Gammaproteobacteria	Pseudomonadales	Pseudomonadaceae	Pseudomonas	Pseudomonas savastanoi	N	https://www.ncbi.nlm.nih.gov/datasets/genome/GCA_024413095.1/
GCA_002019215.1	Pseudomonadota	Gammaproteobacteria	Lysobacterales	Lysobacteraceae	Xanthomonas	Xanthomonas axonopodis	N	https://www.ncbi.nlm.nih.gov/datasets/genome/GCA_002019215.1/
GCA_003701105.1	Pseudomonadota	Gammaproteobacteria	Pseudomonadales	Pseudomonadaceae	Pseudomonas	Pseudomonas syringae group genomosp. 3	N	https://www.ncbi.nlm.nih.gov/datasets/genome/GCA_003701105.1/
GCA_013321255.1	Pseudomonadota	Alphaproteobacteria	Hyphomicrobiales	Rhizobiaceae	Rhizobium	Rhizobium rhizogenes	N	https://www.ncbi.nlm.nih.gov/datasets/genome/GCA_013321255.1/
GCA_013321215.1	Pseudomonadota	Alphaproteobacteria	Hyphomicrobiales	Rhizobiaceae	Rhizobium	Rhizobium rhizogenes	N	https://www.ncbi.nlm.nih.gov/datasets/genome/GCA_013321215.1/
GCA_002891235.1	Pseudomonadota	Gammaproteobacteria	Enterobacterales	Enterobacteriaceae	Enterobacter	Enterobacter cancerogenus	N	https://www.ncbi.nlm.nih.gov/datasets/genome/GCA_002891235.1/
GCA_943912745.1	Pseudomonadota	Gammaproteobacteria	Lysobacterales	Lysobacteraceae	Xanthomonas	Xanthomonas campestris	N	https://www.ncbi.nlm.nih.gov/datasets/genome/GCA_943912745.1/
GCA_013321835.1	Pseudomonadota	Alphaproteobacteria	Hyphomicrobiales	Rhizobiaceae	Rhizobium	Rhizobium rhizogenes	N	https://www.ncbi.nlm.nih.gov/datasets/genome/GCA_013321835.1/
GCA_002019375.1	Pseudomonadota	Gammaproteobacteria	Lysobacterales	Lysobacteraceae	Xanthomonas	Xanthomonas axonopodis	N	https://www.ncbi.nlm.nih.gov/datasets/genome/GCA_002019375.1/
GCA_002270015.1	Pseudomonadota	Gammaproteobacteria	Pseudomonadales	Pseudomonadaceae	Pseudomonas	Pseudomonas savastanoi	N	https://www.ncbi.nlm.nih.gov/datasets/genome/GCA_002270015.1/
GCA_013321715.1	Pseudomonadota	Alphaproteobacteria	Hyphomicrobiales	Rhizobiaceae	Rhizobium	Rhizobium rhizogenes	N	https://www.ncbi.nlm.nih.gov/datasets/genome/GCA_013321715.1/
GCA_037136815.1	Pseudomonadota	Gammaproteobacteria	Enterobacterales	Pectobacteriaceae	Dickeya	Dickeya chrysanthemi	N	https://www.ncbi.nlm.nih.gov/datasets/genome/GCA_037136815.1/
GCA_019775675.1	Pseudomonadota	Alphaproteobacteria	Hyphomicrobiales	Rhizobiaceae	Agrobacterium	Agrobacterium tumefaciens	N	https://www.ncbi.nlm.nih.gov/datasets/genome/GCA_019775675.1/

GCA_021606025.1	Pseudomonadota	Gammaproteobacteria	Pseudomonadales	Pseudomonadaceae	Pseudomonas	Pseudomonas syringae	N	https://www.ncbi.nlm.nih.gov/datasets/genome/GCA_021606025.1/
GCA_003699855.1	Pseudomonadota	Gammaproteobacteria	Pseudomonadales	Pseudomonadaceae	Pseudomonas	Pseudomonas amygdali	N	https://www.ncbi.nlm.nih.gov/datasets/genome/GCA_003699855.1/
GCA_021606305.1	Pseudomonadota	Gammaproteobacteria	Pseudomonadales	Pseudomonadaceae	Pseudomonas	Pseudomonas syringae	N	https://www.ncbi.nlm.nih.gov/datasets/genome/GCA_021606305.1/
GCA_902830915.1	Pseudomonadota	Betaproteobacteria	Burkholderiales	Burkholderiaceae	Burkholderia	Burkholderia gladioli	N	https://www.ncbi.nlm.nih.gov/datasets/genome/GCA_902830915.1/
GCA_003703225.1	Pseudomonadota	Gammaproteobacteria	Pseudomonadales	Pseudomonadaceae	Pseudomonas	Pseudomonas coronafaciens	N	https://www.ncbi.nlm.nih.gov/datasets/genome/GCA_003703225.1/
GCA_003699255.1	Pseudomonadota	Gammaproteobacteria	Pseudomonadales	Pseudomonadaceae	Pseudomonas	Pseudomonas savastanoi	N	https://www.ncbi.nlm.nih.gov/datasets/genome/GCA_003699255.1/
GCA_030370035.1	Pseudomonadota	Gammaproteobacteria	Enterobacterales	Pectobacteriaceae	Dickeya	Dickeya chrysanthemi	N	https://www.ncbi.nlm.nih.gov/datasets/genome/GCA_030370035.1/
GCA_003702985.1	Pseudomonadota	Gammaproteobacteria	Pseudomonadales	Pseudomonadaceae	Pseudomonas	Pseudomonas savastanoi	N	https://www.ncbi.nlm.nih.gov/datasets/genome/GCA_003702985.1/
GCA_013320995.1	Pseudomonadota	Alphaproteobacteria	Hyphomicrobiales	Rhizobiaceae	Rhizobium	Rhizobium rhizogenes	N	https://www.ncbi.nlm.nih.gov/datasets/genome/GCA_013320995.1/
GCA_021606045.1	Pseudomonadota	Gammaproteobacteria	Pseudomonadales	Pseudomonadaceae	Pseudomonas	Pseudomonas syringae	N	https://www.ncbi.nlm.nih.gov/datasets/genome/GCA_021606045.1/
GCA_002019095.1	Pseudomonadota	Gammaproteobacteria	Lysobacterales	Lysobacteraceae	Xanthomonas	Xanthomonas axonopodis	N	https://www.ncbi.nlm.nih.gov/datasets/genome/GCA_002019095.1/
GCA_021606265.1	Pseudomonadota	Gammaproteobacteria	Pseudomonadales	Pseudomonadaceae	Pseudomonas	Pseudomonas syringae	N	https://www.ncbi.nlm.nih.gov/datasets/genome/GCA_021606265.1/
GCA_000959725.1	Pseudomonadota	Betaproteobacteria	Burkholderiales	Burkholderiaceae	Burkholderia	Burkholderia gladioli	N	https://www.ncbi.nlm.nih.gov/datasets/genome/GCA_000959725.1/
GCA_002981475.1	Pseudomonadota	Betaproteobacteria	Burkholderiales	Burkholderiaceae	Burkholderia	Burkholderia gladioli	N	https://www.ncbi.nlm.nih.gov/datasets/genome/GCA_002981475.1/
GCA_013321035.1	Pseudomonadota	Alphaproteobacteria	Hyphomicrobiales	Rhizobiaceae	Rhizobium	Rhizobium rhizogenes	N	https://www.ncbi.nlm.nih.gov/datasets/genome/GCA_013321035.1/
GCA_032594455.1	Pseudomonadota	Gammaproteobacteria	Lysobacterales	Lysobacteraceae	Xylella	Xylella fastidiosa	N	https://www.ncbi.nlm.nih.gov/datasets/genome/GCA_032594455.1/
GCA_001400935.1	Pseudomonadota	Gammaproteobacteria	Pseudomonadales	Pseudomonadaceae	Pseudomonas	Pseudomonas savastanoi	N	https://www.ncbi.nlm.nih.gov/datasets/genome/GCA_001400935.1/
GCA_013321635.1	Pseudomonadota	Alphaproteobacteria	Hyphomicrobiales	Rhizobiaceae	Rhizobium	Rhizobium rhizogenes	N	https://www.ncbi.nlm.nih.gov/datasets/genome/GCA_013321635.1/
GCA_022026035.1	Pseudomonadota	Gammaproteobacteria	Pseudomonadales	Pseudomonadaceae	Pseudomonas	Pseudomonas savastanoi	N	https://www.ncbi.nlm.nih.gov/datasets/genome/GCA_022026035.1/
GCA_013320955.1	Pseudomonadota	Alphaproteobacteria	Hyphomicrobiales	Rhizobiaceae	Rhizobium	Rhizobium rhizogenes	N	https://www.ncbi.nlm.nih.gov/datasets/genome/GCA_013320955.1/
GCA_015682315.1	Pseudomonadota	Gammaproteobacteria	Enterobacterales	Enterobacteriaceae	Enterobacter	Enterobacter cloacae	N	https://www.ncbi.nlm.nih.gov/datasets/genome/GCA_015682315.1/
GCA_013321375.1	Pseudomonadota	Alphaproteobacteria	Hyphomicrobiales	Rhizobiaceae	Rhizobium	Rhizobium rhizogenes	N	https://www.ncbi.nlm.nih.gov/datasets/genome/GCA_013321375.1/
GCA_003699805.1	Pseudomonadota	Gammaproteobacteria	Pseudomonadales	Pseudomonadaceae	Pseudomonas	Pseudomonas syringae group genomosp. 3	N	https://www.ncbi.nlm.nih.gov/datasets/genome/GCA_003699805.1/
GCA_013322155.1	Pseudomonadota	Alphaproteobacteria	Hyphomicrobiales	Rhizobiaceae	Rhizobium	Rhizobium rhizogenes	N	https://www.ncbi.nlm.nih.gov/datasets/genome/GCA_013322155.1/
GCA_900706785.1	Pseudomonadota	Gammaproteobacteria	Enterobacterales	Enterobacteriaceae	Enterobacter	Enterobacter cancerogenus	N	https://www.ncbi.nlm.nih.gov/datasets/genome/GCA_900706785.1/
GCA_021864665.1	Pseudomonadota	Gammaproteobacteria	Lysobacterales	Lysobacteraceae	Xanthomonas	Xanthomonas campestris	N	https://www.ncbi.nlm.nih.gov/datasets/genome/GCA_021864665.1/
GCA_013322015.1	Pseudomonadota	Alphaproteobacteria	Hyphomicrobiales	Rhizobiaceae	Rhizobium	Rhizobium rhizogenes	N	https://www.ncbi.nlm.nih.gov/datasets/genome/GCA_013322015.1/
GCA_022828345.1	Pseudomonadota	Gammaproteobacteria	Pseudomonadales	Pseudomonadaceae	Pseudomonas	Pseudomonas amygdali	N	https://www.ncbi.nlm.nih.gov/datasets/genome/GCA_022828345.1/
GCA_003700715.1	Pseudomonadota	Gammaproteobacteria	Pseudomonadales	Pseudomonadaceae	Pseudomonas	Pseudomonas amygdali	N	https://www.ncbi.nlm.nih.gov/datasets/genome/GCA_003700715.1/
GCA_001275735.1	Pseudomonadota	Gammaproteobacteria	Pseudomonadales	Pseudomonadaceae	Pseudomonas	Pseudomonas coronafaciens	N	https://www.ncbi.nlm.nih.gov/datasets/genome/GCA_001275735.1/
GCA_001417925.1	Pseudomonadota	Gammaproteobacteria	Lysobacterales	Lysobacteraceae	Xylella	Xylella fastidiosa	N	https://www.ncbi.nlm.nih.gov/datasets/genome/GCA_001417925.1/
GCA_000770155.1	Pseudomonadota	Gammaproteobacteria	Enterobacterales	Enterobacteriaceae	Enterobacter	Enterobacter cloacae	N	https://www.ncbi.nlm.nih.gov/datasets/genome/GCA_000770155.1/
GCA_008180055.1	Pseudomonadota	Gammaproteobacteria	Enterobacterales	Enterobacteriaceae	Enterobacter	Enterobacter cloacae	N	https://www.ncbi.nlm.nih.gov/datasets/genome/GCA_008180055.1/
GCA_016308215.1	Pseudomonadota	Gammaproteobacteria	Pseudomonadales	Pseudomonadaceae	Pseudomonas	Pseudomonas syringae	N	https://www.ncbi.nlm.nih.gov/datasets/genome/GCA_016308215.1/
GCA_003700515.1	Pseudomonadota	Gammaproteobacteria	Pseudomonadales	Pseudomonadaceae	Pseudomonas	Pseudomonas coronafaciens	N	https://www.ncbi.nlm.nih.gov/datasets/genome/GCA_003700515.1/
GCA_013364135.1	Pseudomonadota	Gammaproteobacteria	Enterobacterales	Erwiniaceae	Pantoea	Pantoea ananatis	N	https://www.ncbi.nlm.nih.gov/datasets/genome/GCA_013364135.1/
GCA_013912115.1	Pseudomonadota	Gammaproteobacteria	Enterobacterales	Erwiniaceae	Pantoea	Pantoea ananatis	N	https://www.ncbi.nlm.nih.gov/datasets/genome/GCA_013912115.1/
GCA_004127335.1	Pseudomonadota	Gammaproteobacteria	Enterobacterales	Enterobacteriaceae	Enterobacter	Enterobacter cloacae	N	https://www.ncbi.nlm.nih.gov/datasets/genome/GCA_004127335.1/
GCA_002931295.1	Actinomycetota	Actinomycetes	Micrococcales	Microbacteriaceae	Clavibacter	Clavibacter michiganensis	N	https://www.ncbi.nlm.nih.gov/datasets/genome/GCA_002931295.1/
GCA_015682715.1	Pseudomonadota	Gammaproteobacteria	Enterobacterales	Enterobacteriaceae	Enterobacter	Enterobacter cloacae	N	https://www.ncbi.nlm.nih.gov/datasets/genome/GCA_015682715.1/
GCA_001373275.1	Pseudomonadota	Betaproteobacteria	Burkholderiales	Burkholderiaceae	Ralstonia	Ralstonia solanacearum	N	https://www.ncbi.nlm.nih.gov/datasets/genome/GCA_001373275.1/
GCA_002152055.1	Pseudomonadota	Gammaproteobacteria	Enterobacterales	Enterobacteriaceae	Enterobacter	Enterobacter cloacae	N	https://www.ncbi.nlm.nih.gov/datasets/genome/GCA_002152055.1/
GCA_003701915.1	Pseudomonadota	Gammaproteobacteria	Pseudomonadales	Pseudomonadaceae	Pseudomonas	Pseudomonas savastanoi	N	https://www.ncbi.nlm.nih.gov/datasets/genome/GCA_003701915.1/
GCA_015682655.1	Pseudomonadota	Gammaproteobacteria	Enterobacterales	Enterobacteriaceae	Enterobacter	Enterobacter cloacae	N	https://www.ncbi.nlm.nih.gov/datasets/genome/GCA_015682655.1/
GCA_024925425.1	Actinomycetota	Actinomycetes	Micrococcales	Microbacteriaceae	Curtobacterium	Curtobacterium flaccumfaciens	N	https://www.ncbi.nlm.nih.gov/datasets/genome/GCA_024925425.1/
GCA_002278295.1	Pseudomonadota	Gammaproteobacteria	Enterobacterales	Enterobacteriaceae	Enterobacter	Enterobacter cloacae	N	https://www.ncbi.nlm.nih.gov/datasets/genome/GCA_002278295.1/
GCA_015682745.1	Pseudomonadota	Gammaproteobacteria	Enterobacterales	Enterobacteriaceae	Enterobacter	Enterobacter cloacae	N	https://www.ncbi.nlm.nih.gov/datasets/genome/GCA_015682745.1/
GCA_009907135.1	Pseudomonadota	Gammaproteobacteria	Enterobacterales	Enterobacteriaceae	Enterobacter	Enterobacter cloacae	N	https://www.ncbi.nlm.nih.gov/datasets/genome/GCA_009907135.1/
GCA_003986865.1	Pseudomonadota	Gammaproteobacteria	Enterobacterales	Enterobacteriaceae	Enterobacter	Enterobacter cloacae	N	https://www.ncbi.nlm.nih.gov/datasets/genome/GCA_003986865.1/
GCA_020990965.1	Pseudomonadota	Gammaproteobacteria	Lysobacterales	Lysobacteraceae	Xanthomonas	Xanthomonas campestris	N	https://www.ncbi.nlm.nih.gov/datasets/genome/GCA_020990965.1/
GCA_016307755.1	Pseudomonadota	Gammaproteobacteria	Pseudomonadales	Pseudomonadaceae	Pseudomonas	Pseudomonas syringae	N	https://www.ncbi.nlm.nih.gov/datasets/genome/GCA_016307755.1/
GCA_004127365.1	Pseudomonadota	Gammaproteobacteria	Enterobacterales	Enterobacteriaceae	Enterobacter	Enterobacter cloacae	N	https://www.ncbi.nlm.nih.gov/datasets/genome/GCA_004127365.1/
GCA_026622875.1	Pseudomonadota	Alphaproteobacteria	Hyphomicrobiales	Rhizobiaceae	Rhizobium	Rhizobium rhizogenes	N	https://www.ncbi.nlm.nih.gov/datasets/genome/GCA_026622875.1/
GCA_001537985.1	Pseudomonadota	Gammaproteobacteria	Pseudomonadales	Pseudomonadaceae	Pseudomonas	Pseudomonas amygdali	N	https://www.ncbi.nlm.nih.gov/datasets/genome/GCA_001537985.1/
GCA_003985225.1	Pseudomonadota	Gammaproteobacteria	Enterobacterales	Enterobacteriaceae	Enterobacter	Enterobacter cloacae	N	https://www.ncbi.nlm.nih.gov/datasets/genome/GCA_003985225.1/
GCA_013522775.1	Pseudomonadota	Gammaproteobacteria	Pseudomonadales	Pseudomonadaceae	Pseudomonas	Pseudomonas oryzihabitans	N	https://www.ncbi.nlm.nih.gov/datasets/genome/GCA_013522775.1/

GCA_021271025.1	Actinomycetota	Actinomycetes	Micrococcales	Microbacteriaceae	Curtobacterium	Curtobacterium flaccumfaciens	N	https://www.ncbi.nlm.nih.gov/datasets/genome/GCA_021271025.1/
GCA_002528975.1	Pseudomonadota	Gammaproteobacteria	Enterobacterales	Enterobacteriaceae	Enterobacter	Enterobacter cloacae	N	https://www.ncbi.nlm.nih.gov/datasets/genome/GCA_002528975.1/
GCA_009727145.1	Pseudomonadota	Gammaproteobacteria	Enterobacterales	Enterobacteriaceae	Enterobacter	Enterobacter cloacae	N	https://www.ncbi.nlm.nih.gov/datasets/genome/GCA_009727145.1/
GCA_000163275.1	Pseudomonadota	Gammaproteobacteria	Pseudomonadales	Pseudomonadaceae	Pseudomonas	Pseudomonas amygdali	N	https://www.ncbi.nlm.nih.gov/datasets/genome/GCA_000163275.1/
GCA_002278165.1	Pseudomonadota	Gammaproteobacteria	Enterobacterales	Enterobacteriaceae	Enterobacter	Enterobacter cloacae	N	https://www.ncbi.nlm.nih.gov/datasets/genome/GCA_002278165.1/
GCA_029854985.1	Pseudomonadota	Gammaproteobacteria	Pseudomonadales	Pseudomonadaceae	Pseudomonas	Pseudomonas amygdali	N	https://www.ncbi.nlm.nih.gov/datasets/genome/GCA_029854985.1/
GCA_900581155.1	Pseudomonadota	Gammaproteobacteria	Pseudomonadales	Pseudomonadaceae	Pseudomonas	Pseudomonas viridiflava	N	https://www.ncbi.nlm.nih.gov/datasets/genome/GCA_900581155.1/
GCA_002376205.1	Pseudomonadota	Gammaproteobacteria	Pseudomonadales	Pseudomonadaceae	Pseudomonas	Pseudomonas oryzihabitans	N	https://www.ncbi.nlm.nih.gov/datasets/genome/GCA_002376205.1/
GCA_032104565.1	Pseudomonadota	Gammaproteobacteria	Pseudomonadales	Pseudomonadaceae	Pseudomonas	Pseudomonas oryzihabitans	N	https://www.ncbi.nlm.nih.gov/datasets/genome/GCA_032104565.1/
GCA_025985525.1	Pseudomonadota	Gammaproteobacteria	Pseudomonadales	Pseudomonadaceae	Pseudomonas	Pseudomonas viridiflava	N	https://www.ncbi.nlm.nih.gov/datasets/genome/GCA_025985525.1/
GCA_000935305.1	Pseudomonadota	Gammaproteobacteria	Pseudomonadales	Pseudomonadaceae	Pseudomonas	Pseudomonas oryzihabitans	N	https://www.ncbi.nlm.nih.gov/datasets/genome/GCA_000935305.1/
GCA_001400675.1	Pseudomonadota	Gammaproteobacteria	Pseudomonadales	Pseudomonadaceae	Pseudomonas	Pseudomonas amygdali	N	https://www.ncbi.nlm.nih.gov/datasets/genome/GCA_001400675.1/
GCA_010592545.1	Pseudomonadota	Gammaproteobacteria	Enterobacterales	Enterobacteriaceae	Enterobacter	Enterobacter cloacae	N	https://www.ncbi.nlm.nih.gov/datasets/genome/GCA_010592545.1/
GCA_007678515.1	Pseudomonadota	Gammaproteobacteria	Pseudomonadales	Pseudomonadaceae	Pseudomonas	Pseudomonas oryzihabitans	N	https://www.ncbi.nlm.nih.gov/datasets/genome/GCA_007678515.1/
GCA_001294215.1	Pseudomonadota	Gammaproteobacteria	Pseudomonadales	Pseudomonadaceae	Pseudomonas	Pseudomonas amygdali	N	https://www.ncbi.nlm.nih.gov/datasets/genome/GCA_001294215.1/
GCA_900102665.1	Pseudomonadota	Gammaproteobacteria	Pseudomonadales	Pseudomonadaceae	Pseudomonas	Pseudomonas oryzihabitans	N	https://www.ncbi.nlm.nih.gov/datasets/genome/GCA_900102665.1/
GCA_012986165.1	Pseudomonadota	Gammaproteobacteria	Pseudomonadales	Pseudomonadaceae	Pseudomonas	Pseudomonas oryzihabitans	N	https://www.ncbi.nlm.nih.gov/datasets/genome/GCA_012986165.1/
GCA_900581375.1	Pseudomonadota	Gammaproteobacteria	Pseudomonadales	Pseudomonadaceae	Pseudomonas	Pseudomonas viridiflava	N	https://www.ncbi.nlm.nih.gov/datasets/genome/GCA_900581375.1/
GCA_040195035.1	Pseudomonadota	Gammaproteobacteria	Lysobacterales	Lysobacteraceae	Xanthomonas	Xanthomonas campestris	N	https://www.ncbi.nlm.nih.gov/datasets/genome/GCA_040195035.1/
GCA_900580555.1	Pseudomonadota	Gammaproteobacteria	Pseudomonadales	Pseudomonadaceae	Pseudomonas	Pseudomonas viridiflava	N	https://www.ncbi.nlm.nih.gov/datasets/genome/GCA_900580555.1/
GCA_024925305.1	Actinomycetota	Actinomycetes	Micrococcales	Microbacteriaceae	Curtobacterium	Curtobacterium flaccumfaciens	N	https://www.ncbi.nlm.nih.gov/datasets/genome/GCA_024925305.1/
GCA_024919475.1	Actinomycetota	Actinomycetes	Micrococcales	Microbacteriaceae	Curtobacterium	Curtobacterium flaccumfaciens	N	https://www.ncbi.nlm.nih.gov/datasets/genome/GCA_024919475.1/
GCA_026241805.1	Actinomycetota	Actinomycetes	Micrococcales	Microbacteriaceae	Curtobacterium	Curtobacterium flaccumfaciens	N	https://www.ncbi.nlm.nih.gov/datasets/genome/GCA_026241805.1/
GCA_013358225.1	Pseudomonadota	Alphaproteobacteria	Hyphomicrobiales	Rhizobiaceae	Agrobacterium	Agrobacterium tumefaciens	N	https://www.ncbi.nlm.nih.gov/datasets/genome/GCA_013358225.1/
GCA_000755525.1	Pseudomonadota	Gammaproteobacteria	Enterobacterales	Enterobacteriaceae	Enterobacter	Enterobacter cloacae	N	https://www.ncbi.nlm.nih.gov/datasets/genome/GCA_000755525.1/
GCA_004024145.1	Pseudomonadota	Gammaproteobacteria	Enterobacterales	Enterobacteriaceae	Enterobacter	Enterobacter cloacae	N	https://www.ncbi.nlm.nih.gov/datasets/genome/GCA_004024145.1/
GCA_900581245.1	Pseudomonadota	Gammaproteobacteria	Pseudomonadales	Pseudomonadaceae	Pseudomonas	Pseudomonas viridiflava	N	https://www.ncbi.nlm.nih.gov/datasets/genome/GCA_900581245.1/
GCA_013376815.1	Pseudomonadota	Gammaproteobacteria	Enterobacterales	Enterobacteriaceae	Enterobacter	Enterobacter cloacae	N	https://www.ncbi.nlm.nih.gov/datasets/genome/GCA_013376815.1/
GCA_000967935.1	Pseudomonadota	Gammaproteobacteria	Pseudomonadales	Pseudomonadaceae	Pseudomonas	Pseudomonas marginalis	N	https://www.ncbi.nlm.nih.gov/datasets/genome/GCA_000967935.1/
GCA_018383465.1	Pseudomonadota	Gammaproteobacteria	Pseudomonadales	Pseudomonadaceae	Pseudomonas	Pseudomonas syringae	N	https://www.ncbi.nlm.nih.gov/datasets/genome/GCA_018383465.1/
GCA_014489975.1	Pseudomonadota	Alphaproteobacteria	Hyphomicrobiales	Rhizobiaceae	Agrobacterium	Agrobacterium tumefaciens	N	https://www.ncbi.nlm.nih.gov/datasets/genome/GCA_014489975.1/
GCA_003700015.1	Pseudomonadota	Gammaproteobacteria	Pseudomonadales	Pseudomonadaceae	Pseudomonas	Pseudomonas syringae	N	https://www.ncbi.nlm.nih.gov/datasets/genome/GCA_003700015.1/
GCA_016307855.1	Pseudomonadota	Gammaproteobacteria	Pseudomonadales	Pseudomonadaceae	Pseudomonas	Pseudomonas syringae	N	https://www.ncbi.nlm.nih.gov/datasets/genome/GCA_016307855.1/
GCA_034424355.1	Pseudomonadota	Gammaproteobacteria	Pseudomonadales	Pseudomonadaceae	Pseudomonas	Pseudomonas marginalis	N	https://www.ncbi.nlm.nih.gov/datasets/genome/GCA_034424355.1/
GCA_030544665.1	Pseudomonadota	Gammaproteobacteria	Pseudomonadales	Pseudomonadaceae	Pseudomonas	Pseudomonas viridiflava	N	https://www.ncbi.nlm.nih.gov/datasets/genome/GCA_030544665.1/
GCA_012367015.1	Pseudomonadota	Gammaproteobacteria	Enterobacterales	Erwiniaceae	Erwinia	Erwinia amylovora	N	https://www.ncbi.nlm.nih.gov/datasets/genome/GCA_012367015.1/
GCA_012367325.1	Pseudomonadota	Gammaproteobacteria	Enterobacterales	Erwiniaceae	Erwinia	Erwinia amylovora	N	https://www.ncbi.nlm.nih.gov/datasets/genome/GCA_012367325.1/
GCA_003415785.1	Pseudomonadota	Gammaproteobacteria	Pseudomonadales	Pseudomonadaceae	Pseudomonas	Pseudomonas amygdali	N	https://www.ncbi.nlm.nih.gov/datasets/genome/GCA_003415785.1/
GCA_018388505.1	Pseudomonadota	Gammaproteobacteria	Pseudomonadales	Pseudomonadaceae	Pseudomonas	Pseudomonas syringae	N	https://www.ncbi.nlm.nih.gov/datasets/genome/GCA_018388505.1/
GCA_900581105.1	Pseudomonadota	Gammaproteobacteria	Pseudomonadales	Pseudomonadaceae	Pseudomonas	Pseudomonas viridiflava	N	https://www.ncbi.nlm.nih.gov/datasets/genome/GCA_900581105.1/
GCA_019334485.1	Pseudomonadota	Gammaproteobacteria	Pseudomonadales	Pseudomonadaceae	Pseudomonas	Pseudomonas amygdali	N	https://www.ncbi.nlm.nih.gov/datasets/genome/GCA_019334485.1/
GCA_012367595.1	Pseudomonadota	Gammaproteobacteria	Enterobacterales	Erwiniaceae	Erwinia	Erwinia amylovora	N	https://www.ncbi.nlm.nih.gov/datasets/genome/GCA_012367595.1/
GCA_002318825.1	Pseudomonadota	Gammaproteobacteria	Pseudomonadales	Pseudomonadaceae	Pseudomonas	Pseudomonas syringae	N	https://www.ncbi.nlm.nih.gov/datasets/genome/GCA_002318825.1/
GCA_006369965.1	Pseudomonadota	Gammaproteobacteria	Lysobacterales	Lysobacteraceae	Xylella	Xylella fastidiosa	N	https://www.ncbi.nlm.nih.gov/datasets/genome/GCA_006369965.1/
GCA_003700425.1	Pseudomonadota	Gammaproteobacteria	Pseudomonadales	Pseudomonadaceae	Pseudomonas	Pseudomonas amygdali	N	https://www.ncbi.nlm.nih.gov/datasets/genome/GCA_003700425.1/
GCA_037199295.1	Pseudomonadota	Gammaproteobacteria	Enterobacterales	Enterobacteriaceae	Enterobacter	Enterobacter cancerogenus	N	https://www.ncbi.nlm.nih.gov/datasets/genome/GCA_037199295.1/
GCA_946478005.1	Pseudomonadota	Gammaproteobacteria	Enterobacterales	Enterobacteriaceae	Enterobacter	Enterobacter cancerogenus	N	https://www.ncbi.nlm.nih.gov/datasets/genome/GCA_946478005.1/
GCA_002179795.1	Pseudomonadota	Alphaproteobacteria	Hyphomicrobiales	Rhizobiaceae	Agrobacterium	Agrobacterium tumefaciens	N	https://www.ncbi.nlm.nih.gov/datasets/genome/GCA_002179795.1/
GCA_024919455.1	Actinomycetota	Actinomycetes	Micrococcales	Microbacteriaceae	Curtobacterium	Curtobacterium flaccumfaciens	N	https://www.ncbi.nlm.nih.gov/datasets/genome/GCA_024919455.1/
GCA_004319505.1	Pseudomonadota	Gammaproteobacteria	Lysobacterales	Lysobacteraceae	Xanthomonas	Xanthomonas oryzae	N	https://www.ncbi.nlm.nih.gov/datasets/genome/GCA_004319505.1/
GCA_026241835.1	Actinomycetota	Actinomycetes	Micrococcales	Microbacteriaceae	Curtobacterium	Curtobacterium flaccumfaciens	N	https://www.ncbi.nlm.nih.gov/datasets/genome/GCA_026241835.1/
GCA_002318875.1	Pseudomonadota	Gammaproteobacteria	Pseudomonadales	Pseudomonadaceae	Pseudomonas	Pseudomonas syringae	N	https://www.ncbi.nlm.nih.gov/datasets/genome/GCA_002318875.1/
GCA_001538055.1	Pseudomonadota	Gammaproteobacteria	Pseudomonadales	Pseudomonadaceae	Pseudomonas	Pseudomonas amygdali	N	https://www.ncbi.nlm.nih.gov/datasets/genome/GCA_001538055.1/
GCA_016308045.1	Pseudomonadota	Gammaproteobacteria	Pseudomonadales	Pseudomonadaceae	Pseudomonas	Pseudomonas syringae	N	https://www.ncbi.nlm.nih.gov/datasets/genome/GCA_016308045.1/
GCA_031556935.1	Pseudomonadota	Gammaproteobacteria	Enterobacterales	Erwiniaceae	Pantoea	Pantoea cypripedii	N	https://www.ncbi.nlm.nih.gov/datasets/genome/GCA_031556935.1/
GCA_020406575.2	Pseudomonadota	Gammaproteobacteria	Enterobacterales	Pectobacteriaceae	Dickeya	Dickeya zeae	N	https://www.ncbi.nlm.nih.gov/datasets/genome/GCA_020406575.2/

GCA_018598385.1	Actinomycetota	Actinomycetes	Micrococcales	Microbacteriaceae	Curtobacterium	Curtobacterium flaccumfaciens	N	https://www.ncbi.nlm.nih.gov/datasets/genome/GCA_018598385.1/
GCA_017745315.1	Pseudomonadota	Gammaproteobacteria	Lysobacterales	Lysobacteraceae	Xanthomonas	Xanthomonas phaseoli	N	https://www.ncbi.nlm.nih.gov/datasets/genome/GCA_017745315.1/
GCA_018831445.1	Pseudomonadota	Gammaproteobacteria	Lysobacterales	Lysobacteraceae	Xanthomonas	Xanthomonas phaseoli	N	https://www.ncbi.nlm.nih.gov/datasets/genome/GCA_018831445.1/
GCA_001886315.1	Pseudomonadota	Gammaproteobacteria	Lysobacterales	Lysobacteraceae	Xylella	Xylella fastidiosa	N	https://www.ncbi.nlm.nih.gov/datasets/genome/GCA_001886315.1/
GCA_017742795.1	Pseudomonadota	Gammaproteobacteria	Lysobacterales	Lysobacteraceae	Xanthomonas	Xanthomonas phaseoli	N	https://www.ncbi.nlm.nih.gov/datasets/genome/GCA_017742795.1/
GCA_025987725.1	Pseudomonadota	Gammaproteobacteria	Lysobacterales	Lysobacteraceae	Xanthomonas	Xanthomonas phaseoli	N	https://www.ncbi.nlm.nih.gov/datasets/genome/GCA_025987725.1/
GCA_017745345.1	Pseudomonadota	Gammaproteobacteria	Lysobacterales	Lysobacteraceae	Xanthomonas	Xanthomonas phaseoli	N	https://www.ncbi.nlm.nih.gov/datasets/genome/GCA_017745345.1/
GCA_020917325.1	Pseudomonadota	Gammaproteobacteria	Pseudomonadales	Pseudomonadaceae	Pseudomonas	Pseudomonas savastanoi	N	https://www.ncbi.nlm.nih.gov/datasets/genome/GCA_020917325.1/
GCA_006369915.1	Pseudomonadota	Gammaproteobacteria	Lysobacterales	Lysobacteraceae	Xylella	Xylella fastidiosa	N	https://www.ncbi.nlm.nih.gov/datasets/genome/GCA_006369915.1/
GCA_003701175.1	Pseudomonadota	Gammaproteobacteria	Pseudomonadales	Pseudomonadaceae	Pseudomonas	Pseudomonas syringae group genomosp. 3	N	https://www.ncbi.nlm.nih.gov/datasets/genome/GCA_003701175.1/
GCA_900234425.1	Pseudomonadota	Gammaproteobacteria	Lysobacterales	Lysobacteraceae	Xanthomonas	Xanthomonas phaseoli	N	https://www.ncbi.nlm.nih.gov/datasets/genome/GCA_900234425.1/
GCA_003999445.1	Pseudomonadota	Gammaproteobacteria	Lysobacterales	Lysobacteraceae	Xanthomonas	Xanthomonas phaseoli	N	https://www.ncbi.nlm.nih.gov/datasets/genome/GCA_003999445.1/
GCA_000791705.1	Pseudomonadota	Gammaproteobacteria	Pseudomonadales	Pseudomonadaceae	Pseudomonas	Pseudomonas aeruginosa	N	https://www.ncbi.nlm.nih.gov/datasets/genome/GCA_000791705.1/
GCA_016308015.1	Pseudomonadota	Gammaproteobacteria	Pseudomonadales	Pseudomonadaceae	Pseudomonas	Pseudomonas syringae	N	https://www.ncbi.nlm.nih.gov/datasets/genome/GCA_016308015.1/
GCA_000808735.2	Pseudomonadota	Gammaproteobacteria	Lysobacterales	Lysobacteraceae	Xanthomonas	Xanthomonas phaseoli	N	https://www.ncbi.nlm.nih.gov/datasets/genome/GCA_000808735.2/
GCA_002759155.2	Pseudomonadota	Gammaproteobacteria	Lysobacterales	Lysobacteraceae	Xanthomonas	Xanthomonas phaseoli	N	https://www.ncbi.nlm.nih.gov/datasets/genome/GCA_002759155.2/
GCA_025560205.1	Pseudomonadota	Alphaproteobacteria	Hyphomicrobiales	Rhizobiaceae	Agrobacterium	Agrobacterium tumefaciens	N	https://www.ncbi.nlm.nih.gov/datasets/genome/GCA_025560205.1/
GCA_025560005.1	Pseudomonadota	Alphaproteobacteria	Hyphomicrobiales	Rhizobiaceae	Agrobacterium	Agrobacterium tumefaciens	N	https://www.ncbi.nlm.nih.gov/datasets/genome/GCA_025560005.1/
GCA_001010435.1	Pseudomonadota	Gammaproteobacteria	Lysobacterales	Lysobacteraceae	Xanthomonas	Xanthomonas hyacinthi	N	https://www.ncbi.nlm.nih.gov/datasets/genome/GCA_001010435.1/
GCA_000808715.2	Pseudomonadota	Gammaproteobacteria	Lysobacterales	Lysobacteraceae	Xanthomonas	Xanthomonas phaseoli	N	https://www.ncbi.nlm.nih.gov/datasets/genome/GCA_000808715.2/
GCA_003111925.1	Pseudomonadota	Gammaproteobacteria	Lysobacterales	Lysobacteraceae	Xanthomonas	Xanthomonas axonopodis	N	https://www.ncbi.nlm.nih.gov/datasets/genome/GCA_003111925.1/
GCA_000145765.1	Pseudomonadota	Gammaproteobacteria	Pseudomonadales	Pseudomonadaceae	Pseudomonas	Pseudomonas amygdali	N	https://www.ncbi.nlm.nih.gov/datasets/genome/GCA_000145765.1/
GCA_000382585.2	Pseudomonadota	Gammaproteobacteria	Enterobacterales	Pectobacteriaceae	Dickeya	Dickeya zeae	N	https://www.ncbi.nlm.nih.gov/datasets/genome/GCA_000382585.2/
GCA_038396405.1	Pseudomonadota	Gammaproteobacteria	Enterobacterales	Pectobacteriaceae	Dickeya	Dickeya zeae	N	https://www.ncbi.nlm.nih.gov/datasets/genome/GCA_038396405.1/
GCA_000732705.1	Pseudomonadota	Gammaproteobacteria	Lysobacterales	Lysobacteraceae	Xylella	Xylella fastidiosa	N	https://www.ncbi.nlm.nih.gov/datasets/genome/GCA_000732705.1/
GCA_004376015.1	Pseudomonadota	Gammaproteobacteria	Pseudomonadales	Pseudomonadaceae	Pseudomonas	Pseudomonas syringae group genomosp. 3	N	https://www.ncbi.nlm.nih.gov/datasets/genome/GCA_004376015.1/
GCA_008693825.1	Pseudomonadota	Gammaproteobacteria	Pseudomonadales	Pseudomonadaceae	Pseudomonas	Pseudomonas oryzihabitans	N	https://www.ncbi.nlm.nih.gov/datasets/genome/GCA_008693825.1/
GCA_004376045.1	Pseudomonadota	Gammaproteobacteria	Pseudomonadales	Pseudomonadaceae	Pseudomonas	Pseudomonas syringae group genomosp. 3	N	https://www.ncbi.nlm.nih.gov/datasets/genome/GCA_004376045.1/
GCA_036861355.1	Pseudomonadota	Betaproteobacteria	Burkholderiales	Burkholderiaceae	Burkholderia	Burkholderia gladioli	N	https://www.ncbi.nlm.nih.gov/datasets/genome/GCA_036861355.1/
GCA_902829495.1	Pseudomonadota	Betaproteobacteria	Burkholderiales	Burkholderiaceae	Burkholderia	Burkholderia gladioli	N	https://www.ncbi.nlm.nih.gov/datasets/genome/GCA_902829495.1/
GCA_036861395.1	Pseudomonadota	Betaproteobacteria	Burkholderiales	Burkholderiaceae	Burkholderia	Burkholderia gladioli	N	https://www.ncbi.nlm.nih.gov/datasets/genome/GCA_036861395.1/
GCA_001526345.1	Pseudomonadota	Betaproteobacteria	Burkholderiales	Burkholderiaceae	Burkholderia	Burkholderia cepacia	N	https://www.ncbi.nlm.nih.gov/datasets/genome/GCA_001526345.1/
GCA_032002685.1	Pseudomonadota	Gammaproteobacteria	Pseudomonadales	Pseudomonadaceae	Pseudomonas	Pseudomonas oryzihabitans	N	https://www.ncbi.nlm.nih.gov/datasets/genome/GCA_032002685.1/
GCA_902829635.1	Pseudomonadota	Betaproteobacteria	Burkholderiales	Burkholderiaceae	Burkholderia	Burkholderia gladioli	N	https://www.ncbi.nlm.nih.gov/datasets/genome/GCA_902829635.1/
GCA_001530045.1	Pseudomonadota	Betaproteobacteria	Burkholderiales	Burkholderiaceae	Burkholderia	Burkholderia cepacia	N	https://www.ncbi.nlm.nih.gov/datasets/genome/GCA_001530045.1/
GCA_001529825.1	Pseudomonadota	Betaproteobacteria	Burkholderiales	Burkholderiaceae	Burkholderia	Burkholderia cepacia	N	https://www.ncbi.nlm.nih.gov/datasets/genome/GCA_001529825.1/
GCA_003854435.1	Pseudomonadota	Betaproteobacteria	Burkholderiales	Burkholderiaceae	Burkholderia	Burkholderia cepacia	N	https://www.ncbi.nlm.nih.gov/datasets/genome/GCA_003854435.1/
GCA_001522525.1	Pseudomonadota	Betaproteobacteria	Burkholderiales	Burkholderiaceae	Burkholderia	Burkholderia cepacia	N	https://www.ncbi.nlm.nih.gov/datasets/genome/GCA_001522525.1/
GCA_001531525.1	Pseudomonadota	Betaproteobacteria	Burkholderiales	Burkholderiaceae	Burkholderia	Burkholderia cepacia	N	https://www.ncbi.nlm.nih.gov/datasets/genome/GCA_001531525.1/
GCA_902830905.1	Pseudomonadota	Betaproteobacteria	Burkholderiales	Burkholderiaceae	Burkholderia	Burkholderia gladioli	N	https://www.ncbi.nlm.nih.gov/datasets/genome/GCA_902830905.1/
GCA_030482245.1	Pseudomonadota	Betaproteobacteria	Burkholderiales	Burkholderiaceae	Burkholderia	Burkholderia gladioli	N	https://www.ncbi.nlm.nih.gov/datasets/genome/GCA_030482245.1/
GCA_036862195.1	Pseudomonadota	Betaproteobacteria	Burkholderiales	Burkholderiaceae	Burkholderia	Burkholderia gladioli	N	https://www.ncbi.nlm.nih.gov/datasets/genome/GCA_036862195.1/
GCA_001528685.1	Pseudomonadota	Betaproteobacteria	Burkholderiales	Burkholderiaceae	Burkholderia	Burkholderia cepacia	N	https://www.ncbi.nlm.nih.gov/datasets/genome/GCA_001528685.1/
GCA_003858175.1	Pseudomonadota	Betaproteobacteria	Burkholderiales	Burkholderiaceae	Burkholderia	Burkholderia cepacia	N	https://www.ncbi.nlm.nih.gov/datasets/genome/GCA_003858175.1/
GCA_003858185.1	Pseudomonadota	Betaproteobacteria	Burkholderiales	Burkholderiaceae	Burkholderia	Burkholderia cepacia	N	https://www.ncbi.nlm.nih.gov/datasets/genome/GCA_003858185.1/
GCA_016008885.1	Pseudomonadota	Gammaproteobacteria	Pseudomonadales	Pseudomonadaceae	Pseudomonas	Pseudomonas oryzihabitans	N	https://www.ncbi.nlm.nih.gov/datasets/genome/GCA_016008885.1/
GCA_001533445.1	Pseudomonadota	Betaproteobacteria	Burkholderiales	Burkholderiaceae	Burkholderia	Burkholderia cepacia	N	https://www.ncbi.nlm.nih.gov/datasets/genome/GCA_001533445.1/
GCA_001521975.1	Pseudomonadota	Betaproteobacteria	Burkholderiales	Burkholderiaceae	Burkholderia	Burkholderia cepacia	N	https://www.ncbi.nlm.nih.gov/datasets/genome/GCA_001521975.1/
GCA_042604585.1	Pseudomonadota	Betaproteobacteria	Burkholderiales	Burkholderiaceae	Burkholderia	Burkholderia gladioli	N	https://www.ncbi.nlm.nih.gov/datasets/genome/GCA_042604585.1/
GCA_001971465.2	Pseudomonadota	Gammaproteobacteria	Lysobacterales	Lysobacteraceae	Xylella	Xylella fastidiosa	N	https://www.ncbi.nlm.nih.gov/datasets/genome/GCA_001971465.2/
GCA_001526905.1	Pseudomonadota	Betaproteobacteria	Burkholderiales	Burkholderiaceae	Burkholderia	Burkholderia cepacia	N	https://www.ncbi.nlm.nih.gov/datasets/genome/GCA_001526905.1/
GCA_022549195.1	Pseudomonadota	Betaproteobacteria	Burkholderiales	Burkholderiaceae	Burkholderia	Burkholderia gladioli	N	https://www.ncbi.nlm.nih.gov/datasets/genome/GCA_022549195.1/
GCA_004377645.1	Pseudomonadota	Betaproteobacteria	Burkholderiales	Burkholderiaceae	Burkholderia	Burkholderia cepacia	N	https://www.ncbi.nlm.nih.gov/datasets/genome/GCA_004377645.1/
GCA_036862355.1	Pseudomonadota	Betaproteobacteria	Burkholderiales	Burkholderiaceae	Burkholderia	Burkholderia gladioli	N	https://www.ncbi.nlm.nih.gov/datasets/genome/GCA_036862355.1/
GCA_025244785.1	Pseudomonadota	Betaproteobacteria	Burkholderiales	Burkholderiaceae	Burkholderia	Burkholderia gladioli	N	https://www.ncbi.nlm.nih.gov/datasets/genome/GCA_025244785.1/
GCA_001530415.1	Pseudomonadota	Betaproteobacteria	Burkholderiales	Burkholderiaceae	Burkholderia	Burkholderia cepacia	N	https://www.ncbi.nlm.nih.gov/datasets/genome/GCA_001530415.1/

GCA_001522985.1	Pseudomonadota	Betaproteobacteria	Burkholderiales	Burkholderiaceae	Burkholderia	Burkholderia cepacia	N	https://www.ncbi.nlm.nih.gov/datasets/genome/GCA_001522985.1/
GCA_001522805.1	Pseudomonadota	Betaproteobacteria	Burkholderiales	Burkholderiaceae	Burkholderia	Burkholderia cepacia	N	https://www.ncbi.nlm.nih.gov/datasets/genome/GCA_001522805.1/
GCA_016505965.1	Pseudomonadota	Betaproteobacteria	Burkholderiales	Burkholderiaceae	Burkholderia	Burkholderia gladioli	N	https://www.ncbi.nlm.nih.gov/datasets/genome/GCA_016505965.1/
GCA_019969865.1	Pseudomonadota	Alphaproteobacteria	Hyphomicrobiales	Rhizobiaceae	Agrobacterium	Agrobacterium tumefaciens	N	https://www.ncbi.nlm.nih.gov/datasets/genome/GCA_019969865.1/
GCA_041019735.1	Pseudomonadota	Betaproteobacteria	Burkholderiales	Burkholderiaceae	Burkholderia	Burkholderia gladioli	N	https://www.ncbi.nlm.nih.gov/datasets/genome/GCA_041019735.1/
GCA_001522925.1	Pseudomonadota	Betaproteobacteria	Burkholderiales	Burkholderiaceae	Burkholderia	Burkholderia cepacia	N	https://www.ncbi.nlm.nih.gov/datasets/genome/GCA_001522925.1/
GCA_001523985.1	Pseudomonadota	Betaproteobacteria	Burkholderiales	Burkholderiaceae	Burkholderia	Burkholderia cepacia	N	https://www.ncbi.nlm.nih.gov/datasets/genome/GCA_001523985.1/
GCA_001533295.1	Pseudomonadota	Betaproteobacteria	Burkholderiales	Burkholderiaceae	Burkholderia	Burkholderia cepacia	N	https://www.ncbi.nlm.nih.gov/datasets/genome/GCA_001533295.1/
GCA_002980955.1	Pseudomonadota	Betaproteobacteria	Burkholderiales	Burkholderiaceae	Burkholderia	Burkholderia gladioli	N	https://www.ncbi.nlm.nih.gov/datasets/genome/GCA_002980955.1/
GCA_001522875.1	Pseudomonadota	Betaproteobacteria	Burkholderiales	Burkholderiaceae	Burkholderia	Burkholderia cepacia	N	https://www.ncbi.nlm.nih.gov/datasets/genome/GCA_001522875.1/
GCA_030482765.1	Pseudomonadota	Betaproteobacteria	Burkholderiales	Burkholderiaceae	Burkholderia	Burkholderia gladioli	N	https://www.ncbi.nlm.nih.gov/datasets/genome/GCA_030482765.1/
GCA_001529525.1	Pseudomonadota	Betaproteobacteria	Burkholderiales	Burkholderiaceae	Burkholderia	Burkholderia cepacia	N	https://www.ncbi.nlm.nih.gov/datasets/genome/GCA_001529525.1/
GCA_001521915.1	Pseudomonadota	Betaproteobacteria	Burkholderiales	Burkholderiaceae	Burkholderia	Burkholderia cepacia	N	https://www.ncbi.nlm.nih.gov/datasets/genome/GCA_001521915.1/
GCA_001525925.1	Pseudomonadota	Betaproteobacteria	Burkholderiales	Burkholderiaceae	Burkholderia	Burkholderia cepacia	N	https://www.ncbi.nlm.nih.gov/datasets/genome/GCA_001525925.1/
GCA_001522945.1	Pseudomonadota	Betaproteobacteria	Burkholderiales	Burkholderiaceae	Burkholderia	Burkholderia cepacia	N	https://www.ncbi.nlm.nih.gov/datasets/genome/GCA_001522945.1/
GCA_030811185.1	Pseudomonadota	Alphaproteobacteria	Hyphomicrobiales	Rhizobiaceae	Agrobacterium	Agrobacterium tumefaciens	N	https://www.ncbi.nlm.nih.gov/datasets/genome/GCA_030811185.1/
GCA_013401495.1	Pseudomonadota	Alphaproteobacteria	Hyphomicrobiales	Rhizobiaceae	Agrobacterium	Agrobacterium tumefaciens	N	https://www.ncbi.nlm.nih.gov/datasets/genome/GCA_013401495.1/
GCA_001530205.1	Pseudomonadota	Betaproteobacteria	Burkholderiales	Burkholderiaceae	Burkholderia	Burkholderia cepacia	N	https://www.ncbi.nlm.nih.gov/datasets/genome/GCA_001530205.1/
GCA_036861555.1	Pseudomonadota	Betaproteobacteria	Burkholderiales	Burkholderiaceae	Burkholderia	Burkholderia gladioli	N	https://www.ncbi.nlm.nih.gov/datasets/genome/GCA_036861555.1/
GCA_000406065.1	Pseudomonadota	Gammaproteobacteria	Enterobacterales	Pectobacteriaceae	Dickeya	Dickeya chrysanthemi	N	https://www.ncbi.nlm.nih.gov/datasets/genome/GCA_000406065.1/
GCA_016026015.1	Pseudomonadota	Betaproteobacteria	Burkholderiales	Burkholderiaceae	Burkholderia	Burkholderia gladioli	N	https://www.ncbi.nlm.nih.gov/datasets/genome/GCA_016026015.1/
GCA_038040145.1	Pseudomonadota	Betaproteobacteria	Burkholderiales	Burkholderiaceae	Burkholderia	Burkholderia gladioli	N	https://www.ncbi.nlm.nih.gov/datasets/genome/GCA_038040145.1/
GCA_038040075.1	Pseudomonadota	Betaproteobacteria	Burkholderiales	Burkholderiaceae	Burkholderia	Burkholderia gladioli	N	https://www.ncbi.nlm.nih.gov/datasets/genome/GCA_038040075.1/
GCA_017700795.1	Actinomycetota	Actinomycetes	Micrococcales	Microbacteriaceae	Curtobacterium	Curtobacterium flaccumfaciens	N	https://www.ncbi.nlm.nih.gov/datasets/genome/GCA_017700795.1/
GCA_031500285.1	Pseudomonadota	Betaproteobacteria	Burkholderiales	Burkholderiaceae	Burkholderia	Burkholderia gladioli	N	https://www.ncbi.nlm.nih.gov/datasets/genome/GCA_031500285.1/
GCA_001266515.1	Pseudomonadota	Gammaproteobacteria	Lysobacterales	Lysobacteraceae	Xanthomonas	Xanthomonas arboricola	N	https://www.ncbi.nlm.nih.gov/datasets/genome/GCA_001266515.1/
GCA_014141625.1	Pseudomonadota	Betaproteobacteria	Burkholderiales	Burkholderiaceae	Burkholderia	Burkholderia cepacia	N	https://www.ncbi.nlm.nih.gov/datasets/genome/GCA_014141625.1/
GCA_019781985.1	Actinomycetota	Actinomycetes	Mycobacteriales	Nocardiaceae	Rhodococcoides	Rhodococcoides fascians	N	https://www.ncbi.nlm.nih.gov/datasets/genome/GCA_019781985.1/
GCA_001264385.1	Pseudomonadota	Gammaproteobacteria	Lysobacterales	Lysobacteraceae	Xanthomonas	Xanthomonas arboricola	N	https://www.ncbi.nlm.nih.gov/datasets/genome/GCA_001264385.1/
GCA_902829355.1	Pseudomonadota	Betaproteobacteria	Burkholderiales	Burkholderiaceae	Burkholderia	Burkholderia gladioli	N	https://www.ncbi.nlm.nih.gov/datasets/genome/GCA_902829355.1/
GCA_038040095.1	Pseudomonadota	Betaproteobacteria	Burkholderiales	Burkholderiaceae	Burkholderia	Burkholderia gladioli	N	https://www.ncbi.nlm.nih.gov/datasets/genome/GCA_038040095.1/
GCA_002269785.1	Pseudomonadota	Gammaproteobacteria	Pseudomonadales	Pseudomonadaceae	Pseudomonas	Pseudomonas savastanoi	N	https://www.ncbi.nlm.nih.gov/datasets/genome/GCA_002269785.1/
GCA_004377105.1	Pseudomonadota	Betaproteobacteria	Burkholderiales	Burkholderiaceae	Burkholderia	Burkholderia cepacia	N	https://www.ncbi.nlm.nih.gov/datasets/genome/GCA_004377105.1/
GCA_014142075.1	Pseudomonadota	Betaproteobacteria	Burkholderiales	Burkholderiaceae	Burkholderia	Burkholderia cepacia	N	https://www.ncbi.nlm.nih.gov/datasets/genome/GCA_014142075.1/
GCA_001266535.1	Pseudomonadota	Gammaproteobacteria	Lysobacterales	Lysobacteraceae	Xanthomonas	Xanthomonas arboricola	N	https://www.ncbi.nlm.nih.gov/datasets/genome/GCA_001266535.1/
GCA_025000365.1	Actinomycetota	Actinomycetes	Micrococcales	Microbacteriaceae	Curtobacterium	Curtobacterium flaccumfaciens	N	https://www.ncbi.nlm.nih.gov/datasets/genome/GCA_025000365.1/
GCA_001264295.1	Pseudomonadota	Gammaproteobacteria	Lysobacterales	Lysobacteraceae	Xanthomonas	Xanthomonas arboricola	N	https://www.ncbi.nlm.nih.gov/datasets/genome/GCA_001264295.1/
GCA_001264275.1	Pseudomonadota	Gammaproteobacteria	Lysobacterales	Lysobacteraceae	Xanthomonas	Xanthomonas arboricola	N	https://www.ncbi.nlm.nih.gov/datasets/genome/GCA_001264275.1/
GCA_019782455.1	Actinomycetota	Actinomycetes	Mycobacteriales	Nocardiaceae	Rhodococcoides	Rhodococcoides fascians	N	https://www.ncbi.nlm.nih.gov/datasets/genome/GCA_019782455.1/
GCA_019104085.1	Pseudomonadota	Gammaproteobacteria	Pseudomonadales	Pseudomonadaceae	Pseudomonas	Pseudomonas viridiflava	N	https://www.ncbi.nlm.nih.gov/datasets/genome/GCA_019104085.1/
GCA_019781485.1	Actinomycetota	Actinomycetes	Mycobacteriales	Nocardiaceae	Rhodococcoides	Rhodococcoides fascians	N	https://www.ncbi.nlm.nih.gov/datasets/genome/GCA_019781485.1/
GCA_019782485.1	Actinomycetota	Actinomycetes	Mycobacteriales	Nocardiaceae	Rhodococcoides	Rhodococcoides fascians	N	https://www.ncbi.nlm.nih.gov/datasets/genome/GCA_019782485.1/
GCA_001264355.1	Pseudomonadota	Gammaproteobacteria	Lysobacterales	Lysobacteraceae	Xanthomonas	Xanthomonas arboricola	N	https://www.ncbi.nlm.nih.gov/datasets/genome/GCA_001264355.1/
GCA_001264425.1	Pseudomonadota	Gammaproteobacteria	Lysobacterales	Lysobacteraceae	Xanthomonas	Xanthomonas arboricola	N	https://www.ncbi.nlm.nih.gov/datasets/genome/GCA_001264425.1/
GCA_003031365.1	Pseudomonadota	Gammaproteobacteria	Lysobacterales	Lysobacteraceae	Xanthomonas	Xanthomonas oryzae	N	https://www.ncbi.nlm.nih.gov/datasets/genome/GCA_003031365.1/
GCA_035011625.1	Pseudomonadota	Gammaproteobacteria	Lysobacterales	Lysobacteraceae	Xanthomonas	Xanthomonas campestris	N	https://www.ncbi.nlm.nih.gov/datasets/genome/GCA_035011625.1/
GCA_001643295.1	Pseudomonadota	Gammaproteobacteria	Lysobacterales	Lysobacteraceae	Xanthomonas	Xanthomonas arboricola	N	https://www.ncbi.nlm.nih.gov/datasets/genome/GCA_001643295.1/
GCA_018598265.1	Actinomycetota	Actinomycetes	Micrococcales	Microbacteriaceae	Curtobacterium	Curtobacterium flaccumfaciens	N	https://www.ncbi.nlm.nih.gov/datasets/genome/GCA_018598265.1/
GCA_026241795.1	Actinomycetota	Actinomycetes	Micrococcales	Microbacteriaceae	Curtobacterium	Curtobacterium flaccumfaciens	N	https://www.ncbi.nlm.nih.gov/datasets/genome/GCA_026241795.1/
GCA_003410095.1	Pseudomonadota	Gammaproteobacteria	Lysobacterales	Lysobacteraceae	Xanthomonas	Xanthomonas campestris	N	https://www.ncbi.nlm.nih.gov/datasets/genome/GCA_003410095.1/
GCA_030011855.1	Actinomycetota	Actinomycetes	Micrococcales	Microbacteriaceae	Curtobacterium	Curtobacterium flaccumfaciens	N	https://www.ncbi.nlm.nih.gov/datasets/genome/GCA_030011855.1/
GCA_014141995.1	Pseudomonadota	Betaproteobacteria	Burkholderiales	Burkholderiaceae	Burkholderia	Burkholderia cepacia	N	https://www.ncbi.nlm.nih.gov/datasets/genome/GCA_014141995.1/
GCA_002940235.1	Pseudomonadota	Gammaproteobacteria	Lysobacterales	Lysobacteraceae	Xanthomonas	Xanthomonas arboricola	N	https://www.ncbi.nlm.nih.gov/datasets/genome/GCA_002940235.1/
GCA_040195245.1	Pseudomonadota	Gammaproteobacteria	Lysobacterales	Lysobacteraceae	Xanthomonas	Xanthomonas campestris	N	https://www.ncbi.nlm.nih.gov/datasets/genome/GCA_040195245.1/
GCA_001052915.1	Pseudomonadota	Betaproteobacteria	Burkholderiales	Burkholderiaceae	Burkholderia	Burkholderia cepacia	N	https://www.ncbi.nlm.nih.gov/datasets/genome/GCA_001052915.1/
GCA_035012925.1	Pseudomonadota	Gammaproteobacteria	Lysobacterales	Lysobacteraceae	Xanthomonas	Xanthomonas campestris	N	https://www.ncbi.nlm.nih.gov/datasets/genome/GCA_035012925.1/

GCA_040194775.1	Pseudomonadota	Gammaproteobacteria	Lysobacterales	Lysobacteraceae	Xanthomonas	Xanthomonas campestris	N	https://www.ncbi.nlm.nih.gov/datasets/genome/GCA_040194775.1/
GCA_905123105.1	Pseudomonadota	Gammaproteobacteria	Lysobacterales	Lysobacteraceae	Xanthomonas	Xanthomonas arboricola	N	https://www.ncbi.nlm.nih.gov/datasets/genome/GCA_905123105.1/
GCA_019782525.1	Actinomycetota	Actinomycetes	Mycobacteriales	Nocardiaceae	Rhodococcoides	Rhodococcoides fascians	N	https://www.ncbi.nlm.nih.gov/datasets/genome/GCA_019782525.1/
GCA_905220695.1	Pseudomonadota	Gammaproteobacteria	Lysobacterales	Lysobacteraceae	Xanthomonas	Xanthomonas arboricola	N	https://www.ncbi.nlm.nih.gov/datasets/genome/GCA_905220695.1/
GCA_001264345.1	Pseudomonadota	Gammaproteobacteria	Lysobacterales	Lysobacteraceae	Xanthomonas	Xanthomonas arboricola	N	https://www.ncbi.nlm.nih.gov/datasets/genome/GCA_001264345.1/
GCA_018598425.1	Actinomycetota	Actinomycetes	Micrococcales	Microbacteriaceae	Curtobacterium	Curtobacterium flaccumfaciens	N	https://www.ncbi.nlm.nih.gov/datasets/genome/GCA_018598425.1/
GCA_001530005.1	Pseudomonadota	Betaproteobacteria	Burkholderiales	Burkholderiaceae	Burkholderia	Burkholderia cepacia	N	https://www.ncbi.nlm.nih.gov/datasets/genome/GCA_001530005.1/
GCA_019781245.1	Actinomycetota	Actinomycetes	Mycobacteriales	Nocardiaceae	Rhodococcoides	Rhodococcoides fascians	N	https://www.ncbi.nlm.nih.gov/datasets/genome/GCA_019781245.1/
GCA_019782705.1	Actinomycetota	Actinomycetes	Mycobacteriales	Nocardiaceae	Rhodococcoides	Rhodococcoides fascians	N	https://www.ncbi.nlm.nih.gov/datasets/genome/GCA_019782705.1/
GCA_019782685.1	Actinomycetota	Actinomycetes	Mycobacteriales	Nocardiaceae	Rhodococcoides	Rhodococcoides fascians	N	https://www.ncbi.nlm.nih.gov/datasets/genome/GCA_019782685.1/
GCA_019782605.1	Actinomycetota	Actinomycetes	Mycobacteriales	Nocardiaceae	Rhodococcoides	Rhodococcoides fascians	N	https://www.ncbi.nlm.nih.gov/datasets/genome/GCA_019782605.1/
GCA_019781405.1	Actinomycetota	Actinomycetes	Mycobacteriales	Nocardiaceae	Rhodococcoides	Rhodococcoides fascians	N	https://www.ncbi.nlm.nih.gov/datasets/genome/GCA_019781405.1/
GCA_902829615.1	Pseudomonadota	Betaproteobacteria	Burkholderiales	Burkholderiaceae	Burkholderia	Burkholderia gladioli	N	https://www.ncbi.nlm.nih.gov/datasets/genome/GCA_902829615.1/
GCA_019781695.1	Actinomycetota	Actinomycetes	Mycobacteriales	Nocardiaceae	Rhodococcoides	Rhodococcoides fascians	N	https://www.ncbi.nlm.nih.gov/datasets/genome/GCA_019781695.1/
GCA_019783045.1	Actinomycetota	Actinomycetes	Mycobacteriales	Nocardiaceae	Rhodococcoides	Rhodococcoides fascians	N	https://www.ncbi.nlm.nih.gov/datasets/genome/GCA_019783045.1/
GCA_028749625.1	Pseudomonadota	Gammaproteobacteria	Lysobacterales	Lysobacteraceae	Xanthomonas	Xanthomonas campestris	N	https://www.ncbi.nlm.nih.gov/datasets/genome/GCA_028749625.1/
GCA_019781515.1	Actinomycetota	Actinomycetes	Mycobacteriales	Nocardiaceae	Rhodococcoides	Rhodococcoides fascians	N	https://www.ncbi.nlm.nih.gov/datasets/genome/GCA_019781515.1/
GCA_019783075.1	Actinomycetota	Actinomycetes	Mycobacteriales	Nocardiaceae	Rhodococcoides	Rhodococcoides fascians	N	https://www.ncbi.nlm.nih.gov/datasets/genome/GCA_019783075.1/
GCA_019400155.1	Pseudomonadota	Betaproteobacteria	Burkholderiales	Burkholderiaceae	Burkholderia	Burkholderia gladioli	N	https://www.ncbi.nlm.nih.gov/datasets/genome/GCA_019400155.1/
GCA_001530805.1	Pseudomonadota	Betaproteobacteria	Burkholderiales	Burkholderiaceae	Burkholderia	Burkholderia cepacia	N	https://www.ncbi.nlm.nih.gov/datasets/genome/GCA_001530805.1/
GCA_019781885.1	Actinomycetota	Actinomycetes	Mycobacteriales	Nocardiaceae	Rhodococcoides	Rhodococcoides fascians	N	https://www.ncbi.nlm.nih.gov/datasets/genome/GCA_019781885.1/
GCA_011761675.1	Pseudomonadota	Gammaproteobacteria	Lysobacterales	Lysobacteraceae	Xanthomonas	Xanthomonas arboricola	N	https://www.ncbi.nlm.nih.gov/datasets/genome/GCA_011761675.1/
GCA_002259465.1	Actinomycetota	Actinomycetes	Mycobacteriales	Nocardiaceae	Rhodococcoides	Rhodococcoides fascians	N	https://www.ncbi.nlm.nih.gov/datasets/genome/GCA_002259465.1/
GCA_002259505.1	Actinomycetota	Actinomycetes	Mycobacteriales	Nocardiaceae	Rhodococcoides	Rhodococcoides fascians	N	https://www.ncbi.nlm.nih.gov/datasets/genome/GCA_002259505.1/
GCA_019781925.1	Actinomycetota	Actinomycetes	Mycobacteriales	Nocardiaceae	Rhodococcoides	Rhodococcoides fascians	N	https://www.ncbi.nlm.nih.gov/datasets/genome/GCA_019781925.1/
GCA_019781325.1	Actinomycetota	Actinomycetes	Mycobacteriales	Nocardiaceae	Rhodococcoides	Rhodococcoides fascians	N	https://www.ncbi.nlm.nih.gov/datasets/genome/GCA_019781325.1/
GCA_019781765.1	Actinomycetota	Actinomycetes	Mycobacteriales	Nocardiaceae	Rhodococcoides	Rhodococcoides fascians	N	https://www.ncbi.nlm.nih.gov/datasets/genome/GCA_019781765.1/
GCA_001529685.1	Pseudomonadota	Betaproteobacteria	Burkholderiales	Burkholderiaceae	Burkholderia	Burkholderia cepacia	N	https://www.ncbi.nlm.nih.gov/datasets/genome/GCA_001529685.1/
GCA_019781645.1	Actinomycetota	Actinomycetes	Mycobacteriales	Nocardiaceae	Rhodococcoides	Rhodococcoides fascians	N	https://www.ncbi.nlm.nih.gov/datasets/genome/GCA_019781645.1/
GCA_019781545.1	Actinomycetota	Actinomycetes	Mycobacteriales	Nocardiaceae	Rhodococcoides	Rhodococcoides fascians	N	https://www.ncbi.nlm.nih.gov/datasets/genome/GCA_019781545.1/
GCA_011927495.1	Pseudomonadota	Gammaproteobacteria	Lysobacterales	Lysobacteraceae	Xanthomonas	Xanthomonas arboricola	N	https://www.ncbi.nlm.nih.gov/datasets/genome/GCA_011927495.1/
GCA_019782445.1	Actinomycetota	Actinomycetes	Mycobacteriales	Nocardiaceae	Rhodococcoides	Rhodococcoides fascians	N	https://www.ncbi.nlm.nih.gov/datasets/genome/GCA_019782445.1/
GCA_003702965.1	Pseudomonadota	Gammaproteobacteria	Pseudomonadales	Pseudomonadaceae	Pseudomonas	Pseudomonas savastanoi	N	https://www.ncbi.nlm.nih.gov/datasets/genome/GCA_003702965.1/
GCA_019782985.1	Actinomycetota	Actinomycetes	Mycobacteriales	Nocardiaceae	Rhodococcoides	Rhodococcoides fascians	N	https://www.ncbi.nlm.nih.gov/datasets/genome/GCA_019782985.1/
GCA_002940365.1	Pseudomonadota	Gammaproteobacteria	Lysobacterales	Lysobacteraceae	Xanthomonas	Xanthomonas arboricola	N	https://www.ncbi.nlm.nih.gov/datasets/genome/GCA_002940365.1/
GCA_030519585.1	Actinomycetota	Actinomycetes	Micrococcales	Microbacteriaceae	Curtobacterium	Curtobacterium flaccumfaciens	N	https://www.ncbi.nlm.nih.gov/datasets/genome/GCA_030519585.1/
GCA_000306055.1	Pseudomonadota	Gammaproteobacteria	Lysobacterales	Lysobacteraceae	Xanthomonas	Xanthomonas arboricola	N	https://www.ncbi.nlm.nih.gov/datasets/genome/GCA_000306055.1/
GCA_900580415.1	Pseudomonadota	Gammaproteobacteria	Pseudomonadales	Pseudomonadaceae	Pseudomonas	Pseudomonas viridiflava	N	https://www.ncbi.nlm.nih.gov/datasets/genome/GCA_900580415.1/
GCA_018598295.1	Actinomycetota	Actinomycetes	Micrococcales	Microbacteriaceae	Curtobacterium	Curtobacterium flaccumfaciens	N	https://www.ncbi.nlm.nih.gov/datasets/genome/GCA_018598295.1/
GCA_001620005.1	Actinomycetota	Actinomycetes	Mycobacteriales	Nocardiaceae	Rhodococcoides	Rhodococcoides fascians	N	https://www.ncbi.nlm.nih.gov/datasets/genome/GCA_001620005.1/
GCA_015351035.1	Actinomycetota	Actinomycetes	Micrococcales	Microbacteriaceae	Curtobacterium	Curtobacterium flaccumfaciens	N	https://www.ncbi.nlm.nih.gov/datasets/genome/GCA_015351035.1/
GCA_019782265.1	Actinomycetota	Actinomycetes	Mycobacteriales	Nocardiaceae	Rhodococcoides	Rhodococcoides fascians	N	https://www.ncbi.nlm.nih.gov/datasets/genome/GCA_019782265.1/
GCA_900581435.1	Pseudomonadota	Gammaproteobacteria	Pseudomonadales	Pseudomonadaceae	Pseudomonas	Pseudomonas viridiflava	N	https://www.ncbi.nlm.nih.gov/datasets/genome/GCA_900581435.1/
GCA_040201855.2	Pseudomonadota	Gammaproteobacteria	Lysobacterales	Lysobacteraceae	Xanthomonas	Xanthomonas arboricola	N	https://www.ncbi.nlm.nih.gov/datasets/genome/GCA_040201855.2/
GCA_003701235.1	Pseudomonadota	Gammaproteobacteria	Pseudomonadales	Pseudomonadaceae	Pseudomonas	Pseudomonas syringae group genomosp. 3	N	https://www.ncbi.nlm.nih.gov/datasets/genome/GCA_003701235.1/
GCA_900580825.1	Pseudomonadota	Gammaproteobacteria	Pseudomonadales	Pseudomonadaceae	Pseudomonas	Pseudomonas viridiflava	N	https://www.ncbi.nlm.nih.gov/datasets/genome/GCA_900580825.1/
GCA_036349535.1	Pseudomonadota	Gammaproteobacteria	Pseudomonadales	Pseudomonadaceae	Pseudomonas	Pseudomonas viridiflava	N	https://www.ncbi.nlm.nih.gov/datasets/genome/GCA_036349535.1/
GCA_034808185.1	Pseudomonadota	Gammaproteobacteria	Pseudomonadales	Pseudomonadaceae	Pseudomonas	Pseudomonas viridiflava	N	https://www.ncbi.nlm.nih.gov/datasets/genome/GCA_034808185.1/
GCA_900580875.1	Pseudomonadota	Gammaproteobacteria	Pseudomonadales	Pseudomonadaceae	Pseudomonas	Pseudomonas viridiflava	N	https://www.ncbi.nlm.nih.gov/datasets/genome/GCA_900580875.1/
GCA_004124655.1	Pseudomonadota	Gammaproteobacteria	Pseudomonadales	Pseudomonadaceae	Pseudomonas	Pseudomonas syringae	N	https://www.ncbi.nlm.nih.gov/datasets/genome/GCA_004124655.1/
GCA_905220745.1	Pseudomonadota	Gammaproteobacteria	Lysobacterales	Lysobacteraceae	Xanthomonas	Xanthomonas arboricola	N	https://www.ncbi.nlm.nih.gov/datasets/genome/GCA_905220745.1/
GCA_900581305.1	Pseudomonadota	Gammaproteobacteria	Pseudomonadales	Pseudomonadaceae	Pseudomonas	Pseudomonas viridiflava	N	https://www.ncbi.nlm.nih.gov/datasets/genome/GCA_900581305.1/
GCA_019782945.1	Actinomycetota	Actinomycetes	Mycobacteriales	Nocardiaceae	Rhodococcoides	Rhodococcoides fascians	N	https://www.ncbi.nlm.nih.gov/datasets/genome/GCA_019782945.1/
GCA_900580705.1	Pseudomonadota	Gammaproteobacteria	Pseudomonadales	Pseudomonadaceae	Pseudomonas	Pseudomonas viridiflava	N	https://www.ncbi.nlm.nih.gov/datasets/genome/GCA_900580705.1/
GCA_004124735.1	Pseudomonadota	Gammaproteobacteria	Pseudomonadales	Pseudomonadaceae	Pseudomonas	Pseudomonas syringae	N	https://www.ncbi.nlm.nih.gov/datasets/genome/GCA_004124735.1/
GCA_900581085.1	Pseudomonadota	Gammaproteobacteria	Pseudomonadales	Pseudomonadaceae	Pseudomonas	Pseudomonas viridiflava	N	https://www.ncbi.nlm.nih.gov/datasets/genome/GCA_900581085.1/

GCA_003993355.1	Pseudomonadota	Gammaproteobacteria	Lysobacterales	Lysobacteraceae	Xanthomonas	Xanthomonas arboricola	N	https://www.ncbi.nlm.nih.gov/datasets/genome/GCA_003993355.1/
GCA_019782855.1	Actinomycetota	Actinomycetes	Mycobacteriales	Nocardiaceae	Rhodococcoides	Rhodococcoides fascians	N	https://www.ncbi.nlm.nih.gov/datasets/genome/GCA_019782855.1/
GCA_900581325.1	Pseudomonadota	Gammaproteobacteria	Pseudomonadales	Pseudomonadaceae	Pseudomonas	Pseudomonas viridiflava	N	https://www.ncbi.nlm.nih.gov/datasets/genome/GCA_900581325.1/
GCA_037136035.1	Pseudomonadota	Gammaproteobacteria	Enterobacterales	Pectobacteriaceae	Pectobacterium	Pectobacterium carotovorum	N	https://www.ncbi.nlm.nih.gov/datasets/genome/GCA_037136035.1/
GCA_009898315.1	Pseudomonadota	Gammaproteobacteria	Pseudomonadales	Pseudomonadaceae	Pseudomonas	Pseudomonas syringae	N	https://www.ncbi.nlm.nih.gov/datasets/genome/GCA_009898315.1/
GCA_900580665.1	Pseudomonadota	Gammaproteobacteria	Pseudomonadales	Pseudomonadaceae	Pseudomonas	Pseudomonas viridiflava	N	https://www.ncbi.nlm.nih.gov/datasets/genome/GCA_900580665.1/
GCA_900580465.1	Pseudomonadota	Gammaproteobacteria	Pseudomonadales	Pseudomonadaceae	Pseudomonas	Pseudomonas viridiflava	N	https://www.ncbi.nlm.nih.gov/datasets/genome/GCA_900580465.1/
GCA_009898365.1	Pseudomonadota	Gammaproteobacteria	Pseudomonadales	Pseudomonadaceae	Pseudomonas	Pseudomonas syringae	N	https://www.ncbi.nlm.nih.gov/datasets/genome/GCA_009898365.1/
GCA_004124865.1	Pseudomonadota	Gammaproteobacteria	Pseudomonadales	Pseudomonadaceae	Pseudomonas	Pseudomonas syringae	N	https://www.ncbi.nlm.nih.gov/datasets/genome/GCA_004124865.1/
GCA_019782565.1	Actinomycetota	Actinomycetes	Mycobacteriales	Nocardiaceae	Rhodococcoides	Rhodococcoides fascians	N	https://www.ncbi.nlm.nih.gov/datasets/genome/GCA_019782565.1/
GCA_013449385.1	Pseudomonadota	Gammaproteobacteria	Enterobacterales	Pectobacteriaceae	Pectobacterium	Pectobacterium carotovorum	N	https://www.ncbi.nlm.nih.gov/datasets/genome/GCA_013449385.1/
GCA_036349475.1	Pseudomonadota	Gammaproteobacteria	Pseudomonadales	Pseudomonadaceae	Pseudomonas	Pseudomonas viridiflava	N	https://www.ncbi.nlm.nih.gov/datasets/genome/GCA_036349475.1/
GCA_037136335.1	Pseudomonadota	Gammaproteobacteria	Enterobacterales	Pectobacteriaceae	Pectobacterium	Pectobacterium carotovorum	N	https://www.ncbi.nlm.nih.gov/datasets/genome/GCA_037136335.1/
GCA_019685595.1	Actinomycetota	Actinomycetes	Mycobacteriales	Nocardiaceae	Rhodococcoides	Rhodococcoides fascians	N	https://www.ncbi.nlm.nih.gov/datasets/genome/GCA_019685595.1/
GCA_900580435.1	Pseudomonadota	Gammaproteobacteria	Pseudomonadales	Pseudomonadaceae	Pseudomonas	Pseudomonas viridiflava	N	https://www.ncbi.nlm.nih.gov/datasets/genome/GCA_900580435.1/
GCA_040203135.1	Pseudomonadota	Gammaproteobacteria	Lysobacterales	Lysobacteraceae	Xanthomonas	Xanthomonas arboricola	N	https://www.ncbi.nlm.nih.gov/datasets/genome/GCA_040203135.1/
GCA_000808435.1	Pseudomonadota	Gammaproteobacteria	Enterobacterales	Pectobacteriaceae	Pectobacterium	Pectobacterium carotovorum	N	https://www.ncbi.nlm.nih.gov/datasets/genome/GCA_000808435.1/
GCA_020406855.1	Pseudomonadota	Gammaproteobacteria	Enterobacterales	Pectobacteriaceae	Pectobacterium	Pectobacterium carotovorum	N	https://www.ncbi.nlm.nih.gov/datasets/genome/GCA_020406855.1/
GCA_022507295.1	Pseudomonadota	Gammaproteobacteria	Enterobacterales	Pectobacteriaceae	Pectobacterium	Pectobacterium carotovorum	N	https://www.ncbi.nlm.nih.gov/datasets/genome/GCA_022507295.1/
GCA_000749855.1	Pseudomonadota	Gammaproteobacteria	Enterobacterales	Pectobacteriaceae	Pectobacterium	Pectobacterium carotovorum	N	https://www.ncbi.nlm.nih.gov/datasets/genome/GCA_000749855.1/
GCA_037136655.1	Pseudomonadota	Gammaproteobacteria	Enterobacterales	Pectobacteriaceae	Pectobacterium	Pectobacterium carotovorum	N	https://www.ncbi.nlm.nih.gov/datasets/genome/GCA_037136655.1/
GCA_030388995.2	Pseudomonadota	Gammaproteobacteria	Lysobacterales	Lysobacteraceae	Xanthomonas	Xanthomonas arboricola	N	https://www.ncbi.nlm.nih.gov/datasets/genome/GCA_030388995.2/
GCA_001039055.1	Pseudomonadota	Gammaproteobacteria	Enterobacterales	Pectobacteriaceae	Pectobacterium	Pectobacterium carotovorum	N	https://www.ncbi.nlm.nih.gov/datasets/genome/GCA_001039055.1/
GCA_035066895.1	Pseudomonadota	Gammaproteobacteria	Enterobacterales	Pectobacteriaceae	Pectobacterium	Pectobacterium carotovorum	N	https://www.ncbi.nlm.nih.gov/datasets/genome/GCA_035066895.1/
GCA_030388865.2	Pseudomonadota	Gammaproteobacteria	Lysobacterales	Lysobacteraceae	Xanthomonas	Xanthomonas arboricola	N	https://www.ncbi.nlm.nih.gov/datasets/genome/GCA_030388865.2/
GCA_036961575.1	Pseudomonadota	Gammaproteobacteria	Enterobacterales	Pectobacteriaceae	Pectobacterium	Pectobacterium carotovorum	N	https://www.ncbi.nlm.nih.gov/datasets/genome/GCA_036961575.1/
GCA_037136885.1	Pseudomonadota	Gammaproteobacteria	Enterobacterales	Pectobacteriaceae	Pectobacterium	Pectobacterium carotovorum	N	https://www.ncbi.nlm.nih.gov/datasets/genome/GCA_037136885.1/
GCA_035066985.1	Pseudomonadota	Gammaproteobacteria	Enterobacterales	Pectobacteriaceae	Pectobacterium	Pectobacterium carotovorum	N	https://www.ncbi.nlm.nih.gov/datasets/genome/GCA_035066985.1/
GCA_020406635.1	Pseudomonadota	Gammaproteobacteria	Enterobacterales	Pectobacteriaceae	Pectobacterium	Pectobacterium carotovorum	N	https://www.ncbi.nlm.nih.gov/datasets/genome/GCA_020406635.1/
GCA_035066845.1	Pseudomonadota	Gammaproteobacteria	Enterobacterales	Pectobacteriaceae	Pectobacterium	Pectobacterium carotovorum	N	https://www.ncbi.nlm.nih.gov/datasets/genome/GCA_035066845.1/
GCA_037135995.1	Pseudomonadota	Gammaproteobacteria	Enterobacterales	Pectobacteriaceae	Pectobacterium	Pectobacterium carotovorum	N	https://www.ncbi.nlm.nih.gov/datasets/genome/GCA_037135995.1/
GCA_018598625.1	Actinomycetota	Actinomycetes	Micrococcales	Microbacteriaceae	Curtobacterium	Curtobacterium flaccumfaciens	N	https://www.ncbi.nlm.nih.gov/datasets/genome/GCA_018598625.1/
GCA_037136515.1	Pseudomonadota	Gammaproteobacteria	Enterobacterales	Pectobacteriaceae	Pectobacterium	Pectobacterium carotovorum	N	https://www.ncbi.nlm.nih.gov/datasets/genome/GCA_037136515.1/
GCA_021049225.1	Pseudomonadota	Gammaproteobacteria	Enterobacterales	Pectobacteriaceae	Pectobacterium	Pectobacterium carotovorum	N	https://www.ncbi.nlm.nih.gov/datasets/genome/GCA_021049225.1/
GCA_040225055.1	Pseudomonadota	Gammaproteobacteria	Enterobacterales	Pectobacteriaceae	Pectobacterium	Pectobacterium carotovorum	N	https://www.ncbi.nlm.nih.gov/datasets/genome/GCA_040225055.1/
GCA_005771575.1	Pseudomonadota	Gammaproteobacteria	Enterobacterales	Pectobacteriaceae	Pectobacterium	Pectobacterium carotovorum	N	https://www.ncbi.nlm.nih.gov/datasets/genome/GCA_005771575.1/
GCA_001718395.1	Pseudomonadota	Betaproteobacteria	Burkholderiales	Burkholderiaceae	Burkholderia	Burkholderia cepacia	N	https://www.ncbi.nlm.nih.gov/datasets/genome/GCA_001718395.1/
GCA_000758345.1	Pseudomonadota	Gammaproteobacteria	Enterobacterales	Pectobacteriaceae	Dickeya	Dickeya fangzhongdai	N	https://www.ncbi.nlm.nih.gov/datasets/genome/GCA_000758345.1/
GCA_037136475.1	Pseudomonadota	Gammaproteobacteria	Enterobacterales	Pectobacteriaceae	Pectobacterium	Pectobacterium carotovorum	N	https://www.ncbi.nlm.nih.gov/datasets/genome/GCA_037136475.1/
GCA_013321275.1	Pseudomonadota	Alphaproteobacteria	Hyphomicrobiales	Rhizobiaceae	Rhizobium	Rhizobium rhizogenes	N	https://www.ncbi.nlm.nih.gov/datasets/genome/GCA_013321275.1/
GCA_020520265.1	Pseudomonadota	Gammaproteobacteria	Enterobacterales	Pectobacteriaceae	Pectobacterium	Pectobacterium carotovorum	N	https://www.ncbi.nlm.nih.gov/datasets/genome/GCA_020520265.1/
GCA_009765495.1	Pseudomonadota	Gammaproteobacteria	Pseudomonadales	Pseudomonadaceae	Pseudomonas	Pseudomonas viridiflava	N	https://www.ncbi.nlm.nih.gov/datasets/genome/GCA_009765495.1/
GCA_002251605.3	Pseudomonadota	Betaproteobacteria	Burkholderiales	Burkholderiaceae	Ralstonia	Ralstonia solanacearum	N	https://www.ncbi.nlm.nih.gov/datasets/genome/GCA_002251605.3/
GCA_900581265.1	Pseudomonadota	Gammaproteobacteria	Pseudomonadales	Pseudomonadaceae	Pseudomonas	Pseudomonas viridiflava	N	https://www.ncbi.nlm.nih.gov/datasets/genome/GCA_900581265.1/
GCA_900580635.1	Pseudomonadota	Gammaproteobacteria	Pseudomonadales	Pseudomonadaceae	Pseudomonas	Pseudomonas viridiflava	N	https://www.ncbi.nlm.nih.gov/datasets/genome/GCA_900580635.1/
GCA_036350005.1	Pseudomonadota	Gammaproteobacteria	Pseudomonadales	Pseudomonadaceae	Pseudomonas	Pseudomonas viridiflava	N	https://www.ncbi.nlm.nih.gov/datasets/genome/GCA_036350005.1/
GCA_016307675.1	Pseudomonadota	Gammaproteobacteria	Pseudomonadales	Pseudomonadaceae	Pseudomonas	Pseudomonas viridiflava	N	https://www.ncbi.nlm.nih.gov/datasets/genome/GCA_016307675.1/
GCA_000808495.1	Pseudomonadota	Gammaproteobacteria	Enterobacterales	Pectobacteriaceae	Pectobacterium	Pectobacterium carotovorum	N	https://www.ncbi.nlm.nih.gov/datasets/genome/GCA_000808495.1/
GCA_900581355.1	Pseudomonadota	Gammaproteobacteria	Pseudomonadales	Pseudomonadaceae	Pseudomonas	Pseudomonas viridiflava	N	https://www.ncbi.nlm.nih.gov/datasets/genome/GCA_900581355.1/
GCA_001642795.1	Pseudomonadota	Gammaproteobacteria	Pseudomonadales	Pseudomonadaceae	Pseudomonas	Pseudomonas viridiflava	N	https://www.ncbi.nlm.nih.gov/datasets/genome/GCA_001642795.1/
GCA_900581395.1	Pseudomonadota	Gammaproteobacteria	Pseudomonadales	Pseudomonadaceae	Pseudomonas	Pseudomonas viridiflava	N	https://www.ncbi.nlm.nih.gov/datasets/genome/GCA_900581395.1/
GCA_036349785.1	Pseudomonadota	Gammaproteobacteria	Pseudomonadales	Pseudomonadaceae	Pseudomonas	Pseudomonas viridiflava	N	https://www.ncbi.nlm.nih.gov/datasets/genome/GCA_036349785.1/
GCA_036349765.1	Pseudomonadota	Gammaproteobacteria	Pseudomonadales	Pseudomonadaceae	Pseudomonas	Pseudomonas viridiflava	N	https://www.ncbi.nlm.nih.gov/datasets/genome/GCA_036349765.1/
GCA_036349835.1	Pseudomonadota	Gammaproteobacteria	Pseudomonadales	Pseudomonadaceae	Pseudomonas	Pseudomonas viridiflava	N	https://www.ncbi.nlm.nih.gov/datasets/genome/GCA_036349835.1/
GCA_014156405.1	Actinomycetota	Actinomycetes	Micrococcales	Microbacteriaceae	Curtobacterium	Curtobacterium flaccumfaciens	N	https://www.ncbi.nlm.nih.gov/datasets/genome/GCA_014156405.1/
GCA_036349845.1	Pseudomonadota	Gammaproteobacteria	Pseudomonadales	Pseudomonadaceae	Pseudomonas	Pseudomonas viridiflava	N	https://www.ncbi.nlm.nih.gov/datasets/genome/GCA_036349845.1/

GCA_036349715.1	Pseudomonadota	Gammaproteobacteria	Pseudomonadales	Pseudomonadaceae	Pseudomonas	Pseudomonas viridiflava	N	https://www.ncbi.nlm.nih.gov/datasets/genome/GCA_036349715.1/
GCA_036663515.1	Pseudomonadota	Gammaproteobacteria	Enterobacterales	Erwiniaceae	Pantoea	Pantoea cypripedii	N	https://www.ncbi.nlm.nih.gov/datasets/genome/GCA_036663515.1/
GCA_003363805.1	Pseudomonadota	Gammaproteobacteria	Lysobacterales	Lysobacteraceae	Xanthomonas	Xanthomonas campestris	N	https://www.ncbi.nlm.nih.gov/datasets/genome/GCA_003363805.1/
GCA_036349255.1	Pseudomonadota	Gammaproteobacteria	Pseudomonadales	Pseudomonadaceae	Pseudomonas	Pseudomonas viridiflava	N	https://www.ncbi.nlm.nih.gov/datasets/genome/GCA_036349255.1/
GCA_001013485.1	Pseudomonadota	Gammaproteobacteria	Lysobacterales	Lysobacteraceae	Xanthomonas	Xanthomonas arboricola	N	https://www.ncbi.nlm.nih.gov/datasets/genome/GCA_001013485.1/
GCA_007680075.1	Pseudomonadota	Gammaproteobacteria	Pseudomonadales	Pseudomonadaceae	Pseudomonas	Pseudomonas oryzihabitans	N	https://www.ncbi.nlm.nih.gov/datasets/genome/GCA_007680075.1/
GCA_036349955.1	Pseudomonadota	Gammaproteobacteria	Pseudomonadales	Pseudomonadaceae	Pseudomonas	Pseudomonas viridiflava	N	https://www.ncbi.nlm.nih.gov/datasets/genome/GCA_036349955.1/
GCA_003602375.1	Pseudomonadota	Gammaproteobacteria	Lysobacterales	Lysobacteraceae	Xanthomonas	Xanthomonas campestris	N	https://www.ncbi.nlm.nih.gov/datasets/genome/GCA_003602375.1/
GCA_905123915.1	Pseudomonadota	Gammaproteobacteria	Lysobacterales	Lysobacteraceae	Xanthomonas	Xanthomonas arboricola	N	https://www.ncbi.nlm.nih.gov/datasets/genome/GCA_905123915.1/
GCA_003363965.1	Pseudomonadota	Gammaproteobacteria	Lysobacterales	Lysobacteraceae	Xanthomonas	Xanthomonas campestris	N	https://www.ncbi.nlm.nih.gov/datasets/genome/GCA_003363965.1/
GCA_036349345.1	Pseudomonadota	Gammaproteobacteria	Pseudomonadales	Pseudomonadaceae	Pseudomonas	Pseudomonas viridiflava	N	https://www.ncbi.nlm.nih.gov/datasets/genome/GCA_036349345.1/
GCA_019782065.1	Actinomycetota	Actinomycetes	Mycobacteriales	Nocardiaceae	Rhodococcoides	Rhodococcoides fascians	N	https://www.ncbi.nlm.nih.gov/datasets/genome/GCA_019782065.1/
GCA_025962135.1	Pseudomonadota	Gammaproteobacteria	Lysobacterales	Lysobacteraceae	Xanthomonas	Xanthomonas campestris	N	https://www.ncbi.nlm.nih.gov/datasets/genome/GCA_025962135.1/
GCA_020879215.1	Pseudomonadota	Gammaproteobacteria	Lysobacterales	Lysobacteraceae	Xanthomonas	Xanthomonas arboricola	N	https://www.ncbi.nlm.nih.gov/datasets/genome/GCA_020879215.1/
GCA_019781905.1	Actinomycetota	Actinomycetes	Mycobacteriales	Nocardiaceae	Rhodococcoides	Rhodococcoides fascians	N	https://www.ncbi.nlm.nih.gov/datasets/genome/GCA_019781905.1/
GCA_019782135.1	Actinomycetota	Actinomycetes	Mycobacteriales	Nocardiaceae	Rhodococcoides	Rhodococcoides fascians	N	https://www.ncbi.nlm.nih.gov/datasets/genome/GCA_019782135.1/
GCA_019782185.1	Actinomycetota	Actinomycetes	Mycobacteriales	Nocardiaceae	Rhodococcoides	Rhodococcoides fascians	N	https://www.ncbi.nlm.nih.gov/datasets/genome/GCA_019782185.1/
GCA_019781945.1	Actinomycetota	Actinomycetes	Mycobacteriales	Nocardiaceae	Rhodococcoides	Rhodococcoides fascians	N	https://www.ncbi.nlm.nih.gov/datasets/genome/GCA_019781945.1/
GCA_019782045.1	Actinomycetota	Actinomycetes	Mycobacteriales	Nocardiaceae	Rhodococcoides	Rhodococcoides fascians	N	https://www.ncbi.nlm.nih.gov/datasets/genome/GCA_019782045.1/
GCA_019782365.1	Actinomycetota	Actinomycetes	Mycobacteriales	Nocardiaceae	Rhodococcoides	Rhodococcoides fascians	N	https://www.ncbi.nlm.nih.gov/datasets/genome/GCA_019782365.1/
GCA_040631315.1	Actinomycetota	Actinomycetes	Kitasatosporales	Streptomycetaceae	Streptomyces	Streptomyces scabiei	N	https://www.ncbi.nlm.nih.gov/datasets/genome/GCA_040631315.1/
GCA_019782005.1	Actinomycetota	Actinomycetes	Mycobacteriales	Nocardiaceae	Rhodococcoides	Rhodococcoides fascians	N	https://www.ncbi.nlm.nih.gov/datasets/genome/GCA_019782005.1/
GCA_019782325.1	Actinomycetota	Actinomycetes	Mycobacteriales	Nocardiaceae	Rhodococcoides	Rhodococcoides fascians	N	https://www.ncbi.nlm.nih.gov/datasets/genome/GCA_019782325.1/
GCA_013408025.1	Pseudomonadota	Gammaproteobacteria	Lysobacterales	Lysobacteraceae	Xanthomonas	Xanthomonas arboricola	N	https://www.ncbi.nlm.nih.gov/datasets/genome/GCA_013408025.1/
GCA_014199105.1	Pseudomonadota	Gammaproteobacteria	Lysobacterales	Lysobacteraceae	Xanthomonas	Xanthomonas arboricola	N	https://www.ncbi.nlm.nih.gov/datasets/genome/GCA_014199105.1/
GCA_014195715.1	Pseudomonadota	Gammaproteobacteria	Lysobacterales	Lysobacteraceae	Xanthomonas	Xanthomonas arboricola	N	https://www.ncbi.nlm.nih.gov/datasets/genome/GCA_014195715.1/
GCA_020812955.1	Pseudomonadota	Gammaproteobacteria	Lysobacterales	Lysobacteraceae	Xanthomonas	Xanthomonas campestris	N	https://www.ncbi.nlm.nih.gov/datasets/genome/GCA_020812955.1/
GCA_013523015.1	Pseudomonadota	Gammaproteobacteria	Pseudomonadales	Pseudomonadaceae	Pseudomonas	Pseudomonas oryzihabitans	N	https://www.ncbi.nlm.nih.gov/datasets/genome/GCA_013523015.1/
GCA_014199755.1	Pseudomonadota	Gammaproteobacteria	Lysobacterales	Lysobacteraceae	Xanthomonas	Xanthomonas arboricola	N	https://www.ncbi.nlm.nih.gov/datasets/genome/GCA_014199755.1/
GCA_019104065.1	Pseudomonadota	Gammaproteobacteria	Pseudomonadales	Pseudomonadaceae	Pseudomonas	Pseudomonas viridiflava	N	https://www.ncbi.nlm.nih.gov/datasets/genome/GCA_019104065.1/
GCA_025881995.1	Pseudomonadota	Gammaproteobacteria	Enterobacterales	Erwiniaceae	Pantoea	Pantoea ananatis	N	https://www.ncbi.nlm.nih.gov/datasets/genome/GCA_025881995.1/
GCA_003700105.1	Pseudomonadota	Gammaproteobacteria	Pseudomonadales	Pseudomonadaceae	Pseudomonas	Pseudomonas syringae group genomosp. 3	N	https://www.ncbi.nlm.nih.gov/datasets/genome/GCA_003700105.1/
GCA_003700855.1	Pseudomonadota	Gammaproteobacteria	Pseudomonadales	Pseudomonadaceae	Pseudomonas	Pseudomonas syringae group genomosp. 3	N	https://www.ncbi.nlm.nih.gov/datasets/genome/GCA_003700855.1/
GCA_025882585.1	Pseudomonadota	Gammaproteobacteria	Enterobacterales	Erwiniaceae	Pantoea	Pantoea ananatis	N	https://www.ncbi.nlm.nih.gov/datasets/genome/GCA_025882585.1/
GCA_000808215.1	Pseudomonadota	Gammaproteobacteria	Enterobacterales	Pectobacteriaceae	Pectobacterium	Pectobacterium carotovorum	N	https://www.ncbi.nlm.nih.gov/datasets/genome/GCA_000808215.1/
GCA_005233555.1	Pseudomonadota	Gammaproteobacteria	Pseudomonadales	Pseudomonadaceae	Pseudomonas	Pseudomonas viridiflava	N	https://www.ncbi.nlm.nih.gov/datasets/genome/GCA_005233555.1/
GCA_014838925.1	Pseudomonadota	Gammaproteobacteria	Pseudomonadales	Pseudomonadaceae	Pseudomonas	Pseudomonas viridiflava	N	https://www.ncbi.nlm.nih.gov/datasets/genome/GCA_014838925.1/
GCA_013523075.1	Pseudomonadota	Gammaproteobacteria	Pseudomonadales	Pseudomonadaceae	Pseudomonas	Pseudomonas oryzihabitans	N	https://www.ncbi.nlm.nih.gov/datasets/genome/GCA_013523075.1/
GCA_025881925.1	Pseudomonadota	Gammaproteobacteria	Enterobacterales	Erwiniaceae	Pantoea	Pantoea ananatis	N	https://www.ncbi.nlm.nih.gov/datasets/genome/GCA_025881925.1/
GCA_023497985.1	Pseudomonadota	Gammaproteobacteria	Pseudomonadales	Pseudomonadaceae	Pseudomonas	Pseudomonas viridiflava	N	https://www.ncbi.nlm.nih.gov/datasets/genome/GCA_023497985.1/
GCA_001549825.1	Pseudomonadota	Gammaproteobacteria	Lysobacterales	Lysobacteraceae	Xylella	Xylella fastidiosa	N	https://www.ncbi.nlm.nih.gov/datasets/genome/GCA_001549825.1/
GCA_001476115.1	Pseudomonadota	Gammaproteobacteria	Enterobacterales	Erwiniaceae	Pantoea	Pantoea ananatis	N	https://www.ncbi.nlm.nih.gov/datasets/genome/GCA_001476115.1/
GCA_002361025.1	Pseudomonadota	Gammaproteobacteria	Pseudomonadales	Pseudomonadaceae	Pseudomonas	Pseudomonas oryzihabitans	N	https://www.ncbi.nlm.nih.gov/datasets/genome/GCA_002361025.1/
GCA_000811965.1	Pseudomonadota	Gammaproteobacteria	Lysobacterales	Lysobacteraceae	Xylella	Xylella fastidiosa	N	https://www.ncbi.nlm.nih.gov/datasets/genome/GCA_000811965.1/
GCA_025882025.1	Pseudomonadota	Gammaproteobacteria	Enterobacterales	Erwiniaceae	Pantoea	Pantoea ananatis	N	https://www.ncbi.nlm.nih.gov/datasets/genome/GCA_025882025.1/
GCA_001475885.1	Pseudomonadota	Gammaproteobacteria	Enterobacterales	Erwiniaceae	Pantoea	Pantoea ananatis	N	https://www.ncbi.nlm.nih.gov/datasets/genome/GCA_001475885.1/
GCA_001549735.1	Pseudomonadota	Gammaproteobacteria	Lysobacterales	Lysobacteraceae	Xylella	Xylella fastidiosa	N	https://www.ncbi.nlm.nih.gov/datasets/genome/GCA_001549735.1/
GCA_037136455.1	Pseudomonadota	Gammaproteobacteria	Enterobacterales	Pectobacteriaceae	Dickeya	Dickeya chrysanthemi	N	https://www.ncbi.nlm.nih.gov/datasets/genome/GCA_037136455.1/
GCA_002916335.1	Pseudomonadota	Gammaproteobacteria	Pseudomonadales	Pseudomonadaceae	Pseudomonas	Pseudomonas syringae	N	https://www.ncbi.nlm.nih.gov/datasets/genome/GCA_002916335.1/
GCA_963669685.1	Pseudomonadota	Gammaproteobacteria	Lysobacterales	Lysobacteraceae	Xanthomonas	Xanthomonas citri	N	https://www.ncbi.nlm.nih.gov/datasets/genome/GCA_963669685.1/
GCA_001901665.1	Pseudomonadota	Betaproteobacteria	Burkholderiales	Burkholderiaceae	Ralstonia	Ralstonia solanacearum	N	https://www.ncbi.nlm.nih.gov/datasets/genome/GCA_001901665.1/
GCA_002954185.1	Pseudomonadota	Gammaproteobacteria	Lysobacterales	Lysobacteraceae	Xylella	Xylella fastidiosa	N	https://www.ncbi.nlm.nih.gov/datasets/genome/GCA_002954185.1/
GCA_025385085.1	Pseudomonadota	Gammaproteobacteria	Lysobacterales	Lysobacteraceae	Xanthomonas	Xanthomonas citri	N	https://www.ncbi.nlm.nih.gov/datasets/genome/GCA_025385085.1/
GCA_025385235.1	Pseudomonadota	Gammaproteobacteria	Lysobacterales	Lysobacteraceae	Xanthomonas	Xanthomonas citri	N	https://www.ncbi.nlm.nih.gov/datasets/genome/GCA_025385235.1/
GCA_000175135.1	Pseudomonadota	Gammaproteobacteria	Lysobacterales	Lysobacteraceae	Xanthomonas	Xanthomonas citri	N	https://www.ncbi.nlm.nih.gov/datasets/genome/GCA_000175135.1/
GCA_019464635.1	Pseudomonadota	Gammaproteobacteria	Enterobacterales	Pectobacteriaceae	Dickeya	Dickeya zeae	N	https://www.ncbi.nlm.nih.gov/datasets/genome/GCA_019464635.1/

GCA_019444095.1	Pseudomonadota	Gammaproteobacteria	Enterobacterales	Pectobacteriaceae	Dickeya	Dickeya zeae	N	https://www.ncbi.nlm.nih.gov/datasets/genome/GCA_019444095.1/
GCA_001610915.1	Pseudomonadota	Gammaproteobacteria	Lysobacterales	Lysobacteraceae	Xanthomonas	Xanthomonas citri	N	https://www.ncbi.nlm.nih.gov/datasets/genome/GCA_001610915.1/
GCA_000786915.2	Pseudomonadota	Gammaproteobacteria	Lysobacterales	Lysobacteraceae	Xanthomonas	Xanthomonas phaseoli	N	https://www.ncbi.nlm.nih.gov/datasets/genome/GCA_000786915.2/
GCA_037135635.1	Pseudomonadota	Gammaproteobacteria	Enterobacterales	Pectobacteriaceae	Dickeya	Dickeya chrysanthemi	N	https://www.ncbi.nlm.nih.gov/datasets/genome/GCA_037135635.1/
GCA_032088135.1	Pseudomonadota	Gammaproteobacteria	Pseudomonadales	Pseudomonadaceae	Pseudomonas	Pseudomonas oryzihabitans	N	https://www.ncbi.nlm.nih.gov/datasets/genome/GCA_032088135.1/
GCA_000006725.1	Pseudomonadota	Gammaproteobacteria	Lysobacterales	Lysobacteraceae	Xylella	Xylella fastidiosa	N	https://www.ncbi.nlm.nih.gov/datasets/genome/GCA_000006725.1/
GCA_019739115.1	Pseudomonadota	Gammaproteobacteria	Enterobacterales	Pectobacteriaceae	Dickeya	Dickeya chrysanthemi	N	https://www.ncbi.nlm.nih.gov/datasets/genome/GCA_019739115.1/
GCA_002898475.1	Pseudomonadota	Gammaproteobacteria	Lysobacterales	Lysobacteraceae	Xanthomonas	Xanthomonas citri	N	https://www.ncbi.nlm.nih.gov/datasets/genome/GCA_002898475.1/
GCA_041475655.1	Pseudomonadota	Gammaproteobacteria	Lysobacterales	Lysobacteraceae	Xanthomonas	Xanthomonas axonopodis	N	https://www.ncbi.nlm.nih.gov/datasets/genome/GCA_041475655.1/
GCA_000506905.2	Pseudomonadota	Gammaproteobacteria	Lysobacterales	Lysobacteraceae	Xylella	Xylella fastidiosa	N	https://www.ncbi.nlm.nih.gov/datasets/genome/GCA_000506905.2/
GCA_009832785.1	Pseudomonadota	Betaproteobacteria	Burkholderiales	Burkholderiaceae	Ralstonia	Ralstonia solanacearum	N	https://www.ncbi.nlm.nih.gov/datasets/genome/GCA_009832785.1/
GCA_001526205.1	Pseudomonadota	Betaproteobacteria	Burkholderiales	Burkholderiaceae	Burkholderia	Burkholderia cepacia	N	https://www.ncbi.nlm.nih.gov/datasets/genome/GCA_001526205.1/
GCA_002894765.1	Pseudomonadota	Betaproteobacteria	Burkholderiales	Burkholderiaceae	Ralstonia	Ralstonia solanacearum	N	https://www.ncbi.nlm.nih.gov/datasets/genome/GCA_002894765.1/
GCA_000710695.1	Pseudomonadota	Betaproteobacteria	Burkholderiales	Burkholderiaceae	Ralstonia	Ralstonia solanacearum	N	https://www.ncbi.nlm.nih.gov/datasets/genome/GCA_000710695.1/
GCA_001299555.1	Pseudomonadota	Betaproteobacteria	Burkholderiales	Burkholderiaceae	Ralstonia	Ralstonia solanacearum	N	https://www.ncbi.nlm.nih.gov/datasets/genome/GCA_001299555.1/
GCA_002906055.1	Pseudomonadota	Gammaproteobacteria	Pseudomonadales	Pseudomonadaceae	Pseudomonas	Pseudomonas syringae group genomosp. 3	N	https://www.ncbi.nlm.nih.gov/datasets/genome/GCA_002906055.1/
GCA_001696855.1	Pseudomonadota	Betaproteobacteria	Burkholderiales	Burkholderiaceae	Ralstonia	Ralstonia solanacearum	N	https://www.ncbi.nlm.nih.gov/datasets/genome/GCA_001696855.1/
GCA_000749995.1	Pseudomonadota	Betaproteobacteria	Burkholderiales	Burkholderiaceae	Ralstonia	Ralstonia solanacearum	N	https://www.ncbi.nlm.nih.gov/datasets/genome/GCA_000749995.1/
GCA_001696845.1	Pseudomonadota	Betaproteobacteria	Burkholderiales	Burkholderiaceae	Ralstonia	Ralstonia solanacearum	N	https://www.ncbi.nlm.nih.gov/datasets/genome/GCA_001696845.1/
GCA_002501565.1	Pseudomonadota	Betaproteobacteria	Burkholderiales	Burkholderiaceae	Ralstonia	Ralstonia solanacearum	N	https://www.ncbi.nlm.nih.gov/datasets/genome/GCA_002501565.1/
GCA_002894775.1	Pseudomonadota	Betaproteobacteria	Burkholderiales	Burkholderiaceae	Ralstonia	Ralstonia solanacearum	N	https://www.ncbi.nlm.nih.gov/datasets/genome/GCA_002894775.1/
GCA_003860765.1	Pseudomonadota	Betaproteobacteria	Burkholderiales	Burkholderiaceae	Ralstonia	Ralstonia solanacearum	N	https://www.ncbi.nlm.nih.gov/datasets/genome/GCA_003860765.1/
GCA_000710135.3	Pseudomonadota	Betaproteobacteria	Burkholderiales	Burkholderiaceae	Ralstonia	Ralstonia solanacearum	N	https://www.ncbi.nlm.nih.gov/datasets/genome/GCA_000710135.3/
GCA_008271875.1	Pseudomonadota	Betaproteobacteria	Burkholderiales	Burkholderiaceae	Ralstonia	Ralstonia solanacearum	N	https://www.ncbi.nlm.nih.gov/datasets/genome/GCA_008271875.1/
GCA_020520245.1	Pseudomonadota	Gammaproteobacteria	Enterobacterales	Pectobacteriaceae	Dickeya	Dickeya zeae	N	https://www.ncbi.nlm.nih.gov/datasets/genome/GCA_020520245.1/
GCA_001587155.1	Pseudomonadota	Betaproteobacteria	Burkholderiales	Burkholderiaceae	Ralstonia	Ralstonia solanacearum	N	https://www.ncbi.nlm.nih.gov/datasets/genome/GCA_001587155.1/
GCA_003860705.1	Pseudomonadota	Betaproteobacteria	Burkholderiales	Burkholderiaceae	Ralstonia	Ralstonia solanacearum	N	https://www.ncbi.nlm.nih.gov/datasets/genome/GCA_003860705.1/
GCA_000825825.2	Pseudomonadota	Betaproteobacteria	Burkholderiales	Burkholderiaceae	Ralstonia	Ralstonia solanacearum	N	https://www.ncbi.nlm.nih.gov/datasets/genome/GCA_000825825.2/
GCA_013320895.1	Pseudomonadota	Alphaproteobacteria	Hyphomicrobiales	Rhizobiaceae	Agrobacterium	Agrobacterium rubi	N	https://www.ncbi.nlm.nih.gov/datasets/genome/GCA_013320895.1/
GCA_001644815.1	Pseudomonadota	Betaproteobacteria	Burkholderiales	Burkholderiaceae	Ralstonia	Ralstonia solanacearum	N	https://www.ncbi.nlm.nih.gov/datasets/genome/GCA_001644815.1/
GCA_000825885.2	Pseudomonadota	Betaproteobacteria	Burkholderiales	Burkholderiaceae	Ralstonia	Ralstonia solanacearum	N	https://www.ncbi.nlm.nih.gov/datasets/genome/GCA_000825885.2/
GCA_013320905.1	Pseudomonadota	Alphaproteobacteria	Hyphomicrobiales	Rhizobiaceae	Agrobacterium	Agrobacterium rubi	N	https://www.ncbi.nlm.nih.gov/datasets/genome/GCA_013320905.1/
GCA_013320875.1	Pseudomonadota	Alphaproteobacteria	Hyphomicrobiales	Rhizobiaceae	Agrobacterium	Agrobacterium rubi	N	https://www.ncbi.nlm.nih.gov/datasets/genome/GCA_013320875.1/
GCA_017700745.1	Actinomycetota	Actinomycetes	Micrococcales	Microbacteriaceae	Curtobacterium	Curtobacterium flaccumfaciens	N	https://www.ncbi.nlm.nih.gov/datasets/genome/GCA_017700745.1/
GCA_013320925.1	Pseudomonadota	Alphaproteobacteria	Hyphomicrobiales	Rhizobiaceae	Agrobacterium	Agrobacterium rubi	N	https://www.ncbi.nlm.nih.gov/datasets/genome/GCA_013320925.1/
GCA_023212925.1	Pseudomonadota	Gammaproteobacteria	Enterobacterales	Pectobacteriaceae	Dickeya	Dickeya zeae	N	https://www.ncbi.nlm.nih.gov/datasets/genome/GCA_023212925.1/
GCA_016642095.1	Pseudomonadota	Gammaproteobacteria	Enterobacterales	Pectobacteriaceae	Pectobacterium	Pectobacterium carotovorum	N	https://www.ncbi.nlm.nih.gov/datasets/genome/GCA_016642095.1/
GCA_023507825.1	Pseudomonadota	Gammaproteobacteria	Enterobacterales	Pectobacteriaceae	Pectobacterium	Pectobacterium carotovorum	N	https://www.ncbi.nlm.nih.gov/datasets/genome/GCA_023507825.1/
GCA_030269105.1	Pseudomonadota	Gammaproteobacteria	Enterobacterales	Pectobacteriaceae	Pectobacterium	Pectobacterium carotovorum	N	https://www.ncbi.nlm.nih.gov/datasets/genome/GCA_030269105.1/
GCA_024343685.1	Pseudomonadota	Gammaproteobacteria	Enterobacterales	Pectobacteriaceae	Pectobacterium	Pectobacterium carotovorum	N	https://www.ncbi.nlm.nih.gov/datasets/genome/GCA_024343685.1/
GCA_030269145.1	Pseudomonadota	Gammaproteobacteria	Enterobacterales	Pectobacteriaceae	Pectobacterium	Pectobacterium carotovorum	N	https://www.ncbi.nlm.nih.gov/datasets/genome/GCA_030269145.1/
GCA_019718835.1	Pseudomonadota	Gammaproteobacteria	Pseudomonadales	Pseudomonadaceae	Pseudomonas	Pseudomonas cichorii	N	https://www.ncbi.nlm.nih.gov/datasets/genome/GCA_019718835.1/
GCA_014861545.1	Pseudomonadota	Gammaproteobacteria	Enterobacterales	Enterobacteriaceae	Enterobacter	Enterobacter cloacae	N	https://www.ncbi.nlm.nih.gov/datasets/genome/GCA_014861545.1/
GCA_019718875.1	Pseudomonadota	Gammaproteobacteria	Pseudomonadales	Pseudomonadaceae	Pseudomonas	Pseudomonas cichorii	N	https://www.ncbi.nlm.nih.gov/datasets/genome/GCA_019718875.1/
GCA_002411725.1	Pseudomonadota	Gammaproteobacteria	Enterobacterales	Enterobacteriaceae	Enterobacter	Enterobacter cloacae	N	https://www.ncbi.nlm.nih.gov/datasets/genome/GCA_002411725.1/
GCA_019718895.1	Pseudomonadota	Gammaproteobacteria	Pseudomonadales	Pseudomonadaceae	Pseudomonas	Pseudomonas cichorii	N	https://www.ncbi.nlm.nih.gov/datasets/genome/GCA_019718895.1/
GCA_000406225.1	Pseudomonadota	Gammaproteobacteria	Enterobacterales	Pectobacteriaceae	Dickeya	Dickeya zeae	N	https://www.ncbi.nlm.nih.gov/datasets/genome/GCA_000406225.1/
GCA_012985915.1	Pseudomonadota	Gammaproteobacteria	Pseudomonadales	Pseudomonadaceae	Pseudomonas	Pseudomonas oryzihabitans	N	https://www.ncbi.nlm.nih.gov/datasets/genome/GCA_012985915.1/
GCA_026967555.1	Pseudomonadota	Gammaproteobacteria	Lysobacterales	Lysobacteraceae	Xanthomonas	Xanthomonas fragariae	N	https://www.ncbi.nlm.nih.gov/datasets/genome/GCA_026967555.1/
