# Supplementary material for: Prediction of soil probiotics based on foundation model representation enhancement and stacked aggregation classifier
Source: Brief Bioinform. 2025 Oct 29;26(5):bbaf567. doi: 10.1093/bib/bbaf567 (PMC12570017; doi:10.1093/bib/bbaf567)
Supplement: Supplementary_Table_S2_R2_bbaf567 [file supplementary_table_s2_r2_bbaf567.pdf]

Supplementary Table S2. Details of samples division.
(TR-SS1: Training subset 1, TR-SS2: Training subset 2, BAL-TS: Balanced test set, IMBAL-TS: Imbalanced test set)

GCA	Experiment Group 1	Experiment Group 2	Experiment Group 3
GCA_000242855.2	BAL-TS	TR-SS1	TR-SS2
GCA_015880875.1	TR-SS1	TR-SS2	TR-SS2
GCA_026790565.1	TR-SS1	TR-SS2	TR-SS1
GCA_026792875.1	BAL-TS	TR-SS1	TR-SS1
GCA_000699525.1	TR-SS1	TR-SS1	TR-SS1
GCA_019931715.1	TR-SS1	TR-SS1	TR-SS2
GCA_921008455.1	TR-SS1	TR-SS2	TR-SS2
GCA_001375535.1	TR-SS1	TR-SS1	TR-SS2
GCA_002024555.1	TR-SS1	TR-SS1	TR-SS2
GCA_009665145.2	TR-SS1	TR-SS1	TR-SS2
GCA_026123105.1	TR-SS1	TR-SS1	TR-SS1
GCA_019599105.1	BAL-TS	TR-SS2	TR-SS2
GCA_001565875.1	BAL-TS	TR-SS2	TR-SS1
GCA_021655335.1	BAL-TS	TR-SS1	TR-SS2
GCA_001286965.1	BAL-TS	TR-SS1	TR-SS1
GCA_030063805.1	BAL-TS	TR-SS1	TR-SS2
GCA_022453545.1	TR-SS2	TR-SS2	TR-SS1
GCA_023521615.1	TR-SS1	TR-SS1	TR-SS1
GCA_023521595.1	TR-SS1	TR-SS1	TR-SS1
GCA_004119535.1	TR-SS1	TR-SS1	TR-SS1
GCA_905315685.2	TR-SS1	TR-SS1	TR-SS2
GCA_001889285.1	TR-SS1	TR-SS1	TR-SS1
GCA_000747705.1	TR-SS1	IMBAL-TS	TR-SS1
GCA_021172185.1	IMBAL-TS	TR-SS1	TR-SS2
GCA_001617995.1	TR-SS1	TR-SS2	TR-SS1
GCA_001593785.1	TR-SS2	TR-SS1	TR-SS2
GCA_024220155.1	TR-SS1	TR-SS2	TR-SS1
GCA_900156245.1	TR-SS2	TR-SS1	TR-SS1
GCA_029371985.1	BAL-TS	TR-SS2	TR-SS1
GCA_013346225.1	TR-SS1	TR-SS2	TR-SS1
GCA_000196735.1	TR-SS2	IMBAL-TS	TR-SS1
GCA_000982455.1	TR-SS1	TR-SS2	TR-SS1
GCA_001596755.1	TR-SS1	TR-SS2	TR-SS2
GCA_008808015.1	TR-SS2	TR-SS2	TR-SS2
GCA_009497795.1	BAL-TS	TR-SS1	TR-SS1
GCA_905319555.1	TR-SS2	TR-SS1	TR-SS1
GCA_014792065.1	TR-SS2	TR-SS1	TR-SS2
GCA_027497435.1	TR-SS2	TR-SS1	TR-SS1
GCA_004119675.1	BAL-TS	TR-SS2	TR-SS2
GCA_017948535.1	TR-SS1	TR-SS1	TR-SS2
GCA_026788685.1	TR-SS1	TR-SS2	TR-SS2
GCA_016065415.1	TR-SS1	TR-SS1	TR-SS2
GCA_003426125.1	TR-SS2	TR-SS1	TR-SS1
GCA_003665255.1	TR-SS2	TR-SS1	TR-SS2
GCA_032930055.1	TR-SS2	TR-SS2	TR-SS2
GCA_008534375.1	TR-SS2	TR-SS1	TR-SS1
GCA_900104735.1	TR-SS1	TR-SS1	TR-SS1
GCA_900104365.1	BAL-TS	TR-SS1	TR-SS2
GCA_900101415.1	TR-SS2	TR-SS2	TR-SS1
GCA_900101185.1	BAL-TS	TR-SS2	TR-SS1
GCA_033842905.1	TR-SS1	TR-SS1	TR-SS1
GCA_031985445.1	TR-SS2	TR-SS2	TR-SS2
GCA_031455735.1	TR-SS2	TR-SS1	TR-SS1
GCA_031180825.1	TR-SS1	TR-SS1	TR-SS1
GCA_029866525.1	TR-SS1	TR-SS1	TR-SS1
GCA_029457395.1	TR-SS2	TR-SS1	TR-SS2
GCA_024723775.1	TR-SS1	TR-SS1	TR-SS1
GCA_022647425.1	TR-SS1	TR-SS1	TR-SS1
GCA_016463995.1	IMBAL-TS	IMBAL-TS	TR-SS1
GCA_016027415.1	TR-SS2	TR-SS2	IMBAL-TS
GCA_014524625.1	TR-SS2	TR-SS1	TR-SS1
GCA_014490035.1	IMBAL-TS	IMBAL-TS	TR-SS1
GCA_013752735.1	TR-SS1	TR-SS1	TR-SS1
GCA_013387025.1	TR-SS2	TR-SS2	TR-SS1
GCA_013285305.1	TR-SS1	TR-SS2	TR-SS1
GCA_009664975.1	BAL-TS	TR-SS1	TR-SS2

GCA_008831505.1	BAL-TS	TR-SS1	TR-SS1
GCA_008274965.1	BAL-TS	TR-SS1	TR-SS1
GCA_007827425.1	TR-SS1	TR-SS2	TR-SS1
GCA_004723625.1	BAL-TS	TR-SS1	TR-SS1
GCA_004306555.1	TR-SS2	TR-SS2	TR-SS2
GCA_004000925.1	BAL-TS	TR-SS2	TR-SS2
GCA_003729985.1	TR-SS2	TR-SS1	TR-SS2
GCA_002813455.1	TR-SS2	TR-SS1	TR-SS2
GCA_002736065.1	BAL-TS	TR-SS1	TR-SS1
GCA_002220155.1	BAL-TS	IMBAL-TS	TR-SS2
GCA_002072065.1	TR-SS2	TR-SS1	TR-SS1
GCA_001633025.1	TR-SS1	TR-SS2	TR-SS1
GCA_001586155.1	TR-SS2	TR-SS1	TR-SS1
GCA_001439685.1	TR-SS1	TR-SS2	TR-SS1
GCA_000733715.2	TR-SS1	IMBAL-TS	TR-SS2
GCA_000262695.1	TR-SS2	TR-SS2	TR-SS1
GCA_000021045.1	TR-SS2	TR-SS1	TR-SS1
GCA_000020125.1	TR-SS2	TR-SS2	TR-SS1
GCA_000013785.1	BAL-TS	TR-SS2	TR-SS2
GCA_000010725.1	BAL-TS	TR-SS2	TR-SS2
GCA_000010525.1	TR-SS2	TR-SS2	TR-SS2
GCA_036600915.1	TR-SS1	TR-SS2	TR-SS1
GCA_036600855.1	TR-SS1	TR-SS1	TR-SS1
GCA_036600875.1	BAL-TS	TR-SS1	TR-SS1
GCA_036600895.1	BAL-TS	TR-SS1	TR-SS1
GCA_000473085.1	BAL-TS	TR-SS1	TR-SS2
GCA_008932115.1	TR-SS2	TR-SS1	TR-SS2
GCA_024585145.1	BAL-TS	IMBAL-TS	TR-SS2
GCA_024806875.1	TR-SS1	TR-SS1	TR-SS2
GCA_004123905.1	TR-SS1	TR-SS2	TR-SS2
GCA_023278185.1	TR-SS1	TR-SS1	TR-SS2
GCA_030584765.1	TR-SS1	TR-SS2	TR-SS2
GCA_000284375.1	TR-SS1	TR-SS2	TR-SS1
GCA_000261645.1	IMBAL-TS	TR-SS1	TR-SS1
GCA_030160735.1	BAL-TS	TR-SS2	TR-SS2
GCA_018130685.1	IMBAL-TS	TR-SS1	TR-SS1
GCA_018130825.1	TR-SS1	TR-SS2	TR-SS2
GCA_018130725.1	TR-SS1	TR-SS1	TR-SS2
GCA_900011245.1	TR-SS2	TR-SS1	TR-SS2
GCA_900011265.1	TR-SS1	TR-SS1	TR-SS1
GCA_020350345.1	TR-SS2	TR-SS2	TR-SS1
GCA_037201505.1	TR-SS1	TR-SS1	TR-SS1
GCA_035575945.1	BAL-TS	TR-SS1	TR-SS1
GCA_029167075.1	BAL-TS	TR-SS1	TR-SS1
GCA_025200905.1	BAL-TS	TR-SS2	TR-SS1
GCA_005157565.1	TR-SS1	TR-SS1	TR-SS2
GCA_900094575.1	BAL-TS	TR-SS2	TR-SS2
GCA_029167055.1	TR-SS2	IMBAL-TS	TR-SS1
GCA_034421895.1	TR-SS2	TR-SS2	TR-SS1
GCA_014189455.1	TR-SS1	TR-SS2	TR-SS1
GCA_030160235.1	TR-SS2	TR-SS1	TR-SS2
GCA_000709395.2	TR-SS2	TR-SS2	TR-SS1
GCA_003148495.1	TR-SS1	TR-SS1	TR-SS1
GCA_013170705.1	BAL-TS	TR-SS2	TR-SS1
GCA_013170845.1	TR-SS1	TR-SS2	TR-SS1
GCA_001672355.1	TR-SS1	TR-SS2	TR-SS2
GCA_001671485.1	TR-SS1	TR-SS1	TR-SS2
GCA_002119845.1	TR-SS2	TR-SS2	TR-SS1
GCA_000092045.1	TR-SS2	TR-SS1	TR-SS1
GCA_000698845.1	TR-SS1	TR-SS1	TR-SS2
GCA_001908375.1	IMBAL-TS	TR-SS1	TR-SS2
GCA_000442435.1	BAL-TS	TR-SS1	TR-SS1
GCA_003985135.1	TR-SS1	TR-SS1	TR-SS1
GCA_014196235.1	TR-SS1	TR-SS1	TR-SS2
GCA_000731315.1	TR-SS1	TR-SS1	TR-SS1
GCA_021391675.1	TR-SS1	TR-SS2	TR-SS1
GCA_000731295.1	IMBAL-TS	TR-SS2	TR-SS1
GCA_008806425.1	TR-SS1	TR-SS1	TR-SS1
GCA_024384545.1	TR-SS2	TR-SS1	TR-SS2

GCA_017357305.1	TR-SS2	TR-SS2	TR-SS1
GCA_001890425.1	TR-SS1	TR-SS2	TR-SS1
GCA_029714245.1	BAL-TS	TR-SS2	TR-SS1
GCA_001679785.1	TR-SS1	TR-SS2	TR-SS2
GCA_003177055.1	BAL-TS	TR-SS1	TR-SS1
GCA_000018545.1	TR-SS1	TR-SS1	TR-SS2
GCA_024400375.1	TR-SS1	TR-SS1	TR-SS1
GCA_002944405.1	TR-SS1	TR-SS1	TR-SS1
GCA_003100575.1	TR-SS2	TR-SS1	TR-SS2
GCA_037023865.1	TR-SS2	TR-SS1	TR-SS1
GCA_037482275.1	TR-SS1	TR-SS2	TR-SS2
GCA_002197125.1	TR-SS2	TR-SS2	TR-SS1
GCA_002197145.1	TR-SS1	TR-SS1	TR-SS1
GCA_002197025.1	TR-SS1	TR-SS1	TR-SS1
GCA_001315015.1	TR-SS2	TR-SS2	TR-SS1
GCA_008274945.1	TR-SS1	TR-SS1	TR-SS1
GCA_022023855.1	TR-SS1	TR-SS1	TR-SS2
GCA_005222205.1	BAL-TS	TR-SS2	TR-SS2
GCA_008364955.1	TR-SS2	TR-SS1	TR-SS1
GCA_000283655.1	TR-SS2	TR-SS1	TR-SS2
GCA_024170005.1	BAL-TS	TR-SS2	TR-SS1
GCA_900177475.1	TR-SS1	TR-SS1	TR-SS1
GCA_900110885.1	TR-SS1	TR-SS1	TR-SS2
GCA_900108965.1	TR-SS1	TR-SS2	TR-SS1
GCA_900112015.1	TR-SS2	TR-SS1	TR-SS1
GCA_900108885.1	TR-SS1	TR-SS2	TR-SS2
GCA_900114395.1	BAL-TS	TR-SS1	TR-SS1
GCA_016406165.1	BAL-TS	IMBAL-TS	TR-SS1
GCA_000817975.1	TR-SS1	IMBAL-TS	TR-SS1
GCA_004327905.1	TR-SS1	IMBAL-TS	TR-SS1
GCA_004339665.1	BAL-TS	IMBAL-TS	TR-SS2
GCA_030506185.1	TR-SS1	TR-SS1	TR-SS1
GCA_036687375.1	BAL-TS	TR-SS2	TR-SS1
GCA_036687365.1	TR-SS1	TR-SS2	TR-SS1
GCA_000380335.1	BAL-TS	TR-SS1	TR-SS1
GCA_000019845.1	TR-SS2	TR-SS2	TR-SS2
GCA_001402875.1	TR-SS1	TR-SS1	TR-SS1
GCA_001548155.2	TR-SS2	TR-SS1	TR-SS2
GCA_001459775.1	TR-SS1	TR-SS1	TR-SS1
GCA_005768725.1	TR-SS1	TR-SS1	IMBAL-TS
GCA_900108475.1	BAL-TS	TR-SS1	TR-SS1
GCA_000442515.1	TR-SS2	TR-SS1	TR-SS2
GCA_021391315.1	TR-SS1	TR-SS2	TR-SS1
GCA_022227035.1	TR-SS1	TR-SS1	TR-SS2
GCA_003050905.1	TR-SS1	TR-SS1	TR-SS1
GCA_002844445.1	TR-SS1	TR-SS1	TR-SS2
GCA_017599285.1	TR-SS2	TR-SS1	TR-SS2
GCA_000021865.1	TR-SS2	TR-SS1	TR-SS2
GCA_029277405.1	TR-SS1	TR-SS2	TR-SS1
GCA_014622665.1	TR-SS1	TR-SS2	TR-SS1
GCA_003254295.1	TR-SS1	TR-SS1	TR-SS2
GCA_006546695.1	TR-SS1	TR-SS1	TR-SS1
GCA_000021005.1	TR-SS1	TR-SS1	TR-SS2
GCA_003324715.1	TR-SS2	TR-SS1	TR-SS2
GCA_000273405.1	TR-SS1	TR-SS1	TR-SS2
GCA_012647365.1	TR-SS1	TR-SS2	TR-SS1
GCA_001685625.1	TR-SS1	TR-SS2	TR-SS1
GCA_900187365.1	TR-SS1	TR-SS1	TR-SS2
GCA_002937135.1	TR-SS2	TR-SS1	TR-SS1
GCA_003258765.1	TR-SS1	TR-SS1	TR-SS2
GCA_025961595.1	IMBAL-TS	TR-SS2	TR-SS1
GCA_025961435.1	TR-SS1	TR-SS1	TR-SS1
GCA_002937115.1	TR-SS1	TR-SS1	TR-SS1
GCA_016584445.1	TR-SS2	TR-SS1	TR-SS2
GCA_025811435.1	TR-SS1	TR-SS1	TR-SS2
GCA_026625025.1	TR-SS1	TR-SS2	TR-SS1
GCA_031600295.1	IMBAL-TS	TR-SS2	TR-SS1
GCA_027912195.1	TR-SS2	TR-SS1	TR-SS2
GCA_000225955.1	IMBAL-TS	TR-SS1	TR-SS1

GCA_019134555.1	TR-SS1	TR-SS2	TR-SS1
GCA_000013085.1	TR-SS1	TR-SS1	TR-SS2
GCA_016583945.1	TR-SS2	TR-SS2	TR-SS1
GCA_016583925.1	TR-SS1	TR-SS2	TR-SS1
GCA_000515255.1	TR-SS2	TR-SS2	TR-SS2
GCA_016583505.1	BAL-TS	TR-SS2	TR-SS1
GCA_001633145.1	TR-SS1	TR-SS2	TR-SS1
GCA_001633165.1	BAL-TS	TR-SS1	TR-SS1
GCA_025961485.1	TR-SS1	TR-SS2	TR-SS1
GCA_001941695.1	TR-SS2	TR-SS2	TR-SS1
GCA_010119435.1	TR-SS1	TR-SS2	TR-SS1
GCA_004340905.1	TR-SS1	TR-SS2	TR-SS1
GCA_000284255.1	TR-SS2	TR-SS1	TR-SS2
GCA_016583525.1	TR-SS2	TR-SS1	TR-SS2
GCA_015751865.1	TR-SS1	TR-SS1	TR-SS1
GCA_016583725.1	TR-SS2	TR-SS1	TR-SS1
GCA_009662475.1	TR-SS2	TR-SS2	TR-SS2
GCA_006718285.1	TR-SS2	TR-SS2	TR-SS1
GCA_000227215.2	TR-SS2	TR-SS1	TR-SS2
GCA_033842445.1	TR-SS1	TR-SS1	TR-SS1
GCA_031460335.1	TR-SS2	TR-SS1	TR-SS2
GCA_000832905.1	TR-SS1	TR-SS1	TR-SS1
GCA_001039495.1	TR-SS1	TR-SS1	TR-SS1
GCA_019443305.1	TR-SS2	TR-SS2	TR-SS2
GCA_004114715.1	TR-SS1	TR-SS1	TR-SS1
GCA_003184245.1	TR-SS2	TR-SS1	TR-SS1
GCA_030123345.1	TR-SS2	TR-SS2	TR-SS2
GCA_030123225.1	TR-SS2	TR-SS2	TR-SS2
GCA_023657595.1	BAL-TS	TR-SS2	TR-SS2
GCA_025732135.1	TR-SS1	TR-SS1	TR-SS1
GCA_002243645.1	TR-SS2	TR-SS2	TR-SS1
GCA_030123325.1	TR-SS2	TR-SS1	TR-SS2
GCA_039905915.1	TR-SS1	TR-SS1	TR-SS2
GCA_030123045.1	TR-SS1	TR-SS1	TR-SS1
GCA_002706795.1	TR-SS1	TR-SS1	TR-SS2
GCA_003590075.1	TR-SS1	TR-SS1	TR-SS1
GCA_033192675.1	TR-SS1	TR-SS2	TR-SS2
GCA_002865525.1	TR-SS2	TR-SS2	TR-SS1
GCA_003663745.1	TR-SS1	TR-SS1	TR-SS1
GCA_001187725.1	BAL-TS	TR-SS1	TR-SS1
GCA_006540225.1	BAL-TS	TR-SS2	TR-SS1
GCA_018333155.1	TR-SS2	TR-SS1	TR-SS2
GCA_036213185.1	TR-SS1	TR-SS2	TR-SS1
GCA_002897295.1	TR-SS2	TR-SS2	TR-SS1
GCA_000756615.1	TR-SS1	TR-SS1	TR-SS2
GCA_000993825.1	TR-SS1	TR-SS1	TR-SS1
GCA_000520635.1	TR-SS1	TR-SS1	TR-SS1
GCA_029542585.1	BAL-TS	TR-SS1	TR-SS1
GCA_000250655.1	TR-SS2	TR-SS1	TR-SS1
GCA_000218915.1	TR-SS1	TR-SS1	TR-SS1
GCA_028751605.1	TR-SS1	TR-SS2	TR-SS1
GCA_000236805.2	BAL-TS	TR-SS2	TR-SS1
GCA_014692735.1	TR-SS1	TR-SS2	TR-SS1
GCA_036630045.1	TR-SS2	TR-SS1	TR-SS1
GCA_040024215.1	TR-SS1	TR-SS2	TR-SS1
GCA_001272655.2	TR-SS1	TR-SS2	TR-SS1
GCA_015710975.1	TR-SS1	IMBAL-TS	TR-SS1
GCA_022811565.1	TR-SS1	IMBAL-TS	TR-SS2
GCA_001874425.3	TR-SS1	TR-SS2	TR-SS2
GCA_001922145.1	TR-SS1	TR-SS1	TR-SS2
GCA_000389675.2	TR-SS2	IMBAL-TS	TR-SS2
GCA_003047065.1	TR-SS1	IMBAL-TS	TR-SS2
GCA_034298135.1	TR-SS1	IMBAL-TS	TR-SS2
GCA_003952845.1	TR-SS2	IMBAL-TS	TR-SS1
GCA_024397395.1	TR-SS1	IMBAL-TS	TR-SS1
GCA_003813165.1	TR-SS2	TR-SS2	TR-SS1
GCA_900475625.1	TR-SS2	TR-SS1	TR-SS1
GCA_002762175.1	TR-SS1	TR-SS1	TR-SS2
GCA_006228205.1	IMBAL-TS	TR-SS1	TR-SS2

GCA_006228285.1	TR-SS2	TR-SS1	TR-SS1
GCA_900015005.1	IMBAL-TS	TR-SS1	TR-SS2
GCA_943193025.1	TR-SS1	TR-SS1	TR-SS2
GCA_000504525.1	TR-SS2	TR-SS2	TR-SS2
GCA_900070175.1	TR-SS1	TR-SS1	TR-SS2
GCA_900196735.1	BAL-TS	TR-SS2	TR-SS1
GCA_001888925.1	TR-SS1	TR-SS1	TR-SS1
GCA_006740305.1	BAL-TS	TR-SS1	TR-SS1
GCA_000056065.1	BAL-TS	TR-SS1	TR-SS1
GCA_001908415.1	BAL-TS	TR-SS2	TR-SS2
GCA_002278095.1	TR-SS1	TR-SS2	TR-SS2
GCA_003053085.1	TR-SS2	TR-SS2	TR-SS2
GCA_018408455.1	TR-SS1	TR-SS1	TR-SS1
GCA_002849935.1	TR-SS1	TR-SS1	TR-SS2
GCA_002849955.1	TR-SS1	TR-SS2	TR-SS1
GCA_039623715.1	BAL-TS	TR-SS2	TR-SS1
GCA_000155515.2	BAL-TS	TR-SS1	TR-SS1
GCA_000829035.1	BAL-TS	TR-SS2	TR-SS1
GCA_028609725.1	TR-SS1	TR-SS2	TR-SS2
GCA_030061895.1	TR-SS2	TR-SS1	TR-SS1
GCA_026013725.1	TR-SS1	TR-SS2	TR-SS2
GCA_005864225.1	TR-SS1	TR-SS2	TR-SS1
GCA_001435895.1	TR-SS2	TR-SS1	TR-SS2
GCA_001311355.1	BAL-TS	TR-SS1	TR-SS2
GCA_000238835.1	BAL-TS	TR-SS1	TR-SS2
GCA_902388245.1	IMBAL-TS	TR-SS1	TR-SS2
GCA_006151905.1	BAL-TS	TR-SS2	TR-SS1
GCA_900636965.1	TR-SS1	TR-SS1	TR-SS1
GCA_016653515.1	TR-SS2	TR-SS1	TR-SS2
GCA_032465975.1	TR-SS2	TR-SS1	TR-SS1
GCA_031593775.1	IMBAL-TS	TR-SS1	TR-SS1
GCA_003176835.1	TR-SS1	TR-SS1	TR-SS1
GCA_029023865.1	TR-SS1	TR-SS2	TR-SS1
GCA_016028835.1	TR-SS2	TR-SS1	TR-SS1
GCA_016649195.2	TR-SS1	TR-SS2	TR-SS1
GCA_028994235.1	TR-SS1	TR-SS1	TR-SS1
GCA_000014505.1	TR-SS1	TR-SS2	TR-SS1
GCA_016127775.1	TR-SS1	TR-SS1	TR-SS2
GCA_030480445.1	TR-SS2	TR-SS2	TR-SS1
GCA_033882205.1	BAL-TS	TR-SS1	TR-SS1
GCA_007923185.1	BAL-TS	TR-SS1	TR-SS2
GCA_903886475.1	TR-SS2	TR-SS2	TR-SS1
GCA_010120595.1	TR-SS2	TR-SS1	TR-SS2
GCA_900474985.1	BAL-TS	TR-SS2	TR-SS1
GCA_903886645.1	TR-SS1	TR-SS1	TR-SS1
GCA_903886745.1	TR-SS2	TR-SS2	TR-SS1
GCA_000197735.1	TR-SS1	IMBAL-TS	TR-SS1
GCA_004525745.1	BAL-TS	IMBAL-TS	TR-SS2
GCA_002878675.1	TR-SS2	IMBAL-TS	TR-SS1
GCA_014897395.1	TR-SS2	IMBAL-TS	TR-SS1
GCA_006538985.1	TR-SS1	TR-SS1	TR-SS2
GCA_039535035.1	BAL-TS	TR-SS2	TR-SS1
GCA_025421775.1	TR-SS2	TR-SS2	TR-SS2
GCA_030546435.1	TR-SS2	TR-SS1	TR-SS2
GCA_030546425.1	TR-SS2	TR-SS1	TR-SS2
GCA_040208375.1	TR-SS1	TR-SS1	TR-SS2
GCA_038098565.1	TR-SS1	TR-SS1	TR-SS2
GCA_040208385.1	IMBAL-TS	TR-SS2	TR-SS2
GCA_002208825.2	TR-SS2	TR-SS2	TR-SS2
GCA_027105095.1	TR-SS2	IMBAL-TS	TR-SS1
GCA_027886425.1	TR-SS1	TR-SS2	TR-SS1
GCA_001592205.1	TR-SS1	TR-SS1	TR-SS1
GCA_030818665.1	TR-SS1	TR-SS1	TR-SS1
GCA_001571145.1	TR-SS2	TR-SS2	TR-SS2
GCA_024927925.1	TR-SS2	TR-SS1	TR-SS1
GCA_004343255.1	TR-SS1	TR-SS2	TR-SS1
GCA_020546525.1	TR-SS2	TR-SS2	TR-SS2
GCA_002982115.1	TR-SS1	TR-SS1	TR-SS1
GCA_001629735.1	TR-SS1	TR-SS2	TR-SS2

GCA_001581875.1	BAL-TS	TR-SS1	TR-SS2
GCA_001598075.1	TR-SS2	TR-SS1	TR-SS1
GCA_014647735.1	TR-SS1	TR-SS1	IMBAL-TS
GCA_013004695.1	TR-SS2	TR-SS1	IMBAL-TS
GCA_900215245.1	TR-SS2	TR-SS1	TR-SS2
GCA_900475215.1	TR-SS1	TR-SS2	TR-SS2
GCA_001307275.1	TR-SS1	TR-SS1	TR-SS2
GCA_015074865.1	IMBAL-TS	TR-SS1	TR-SS2
GCA_038447645.1	TR-SS1	TR-SS1	TR-SS2
GCA_000412675.1	BAL-TS	TR-SS1	TR-SS1
GCA_024508115.1	TR-SS1	TR-SS2	TR-SS2
GCA_003228315.1	TR-SS1	TR-SS1	TR-SS2
GCA_001630725.2	TR-SS1	TR-SS2	TR-SS1
GCA_002356095.1	BAL-TS	TR-SS1	TR-SS1
GCA_019704535.1	TR-SS1	TR-SS2	TR-SS2
GCA_015291885.1	TR-SS1	TR-SS2	TR-SS2
GCA_000219605.1	BAL-TS	TR-SS2	TR-SS2
GCA_016028655.1	TR-SS1	TR-SS1	TR-SS1
GCA_001648195.1	TR-SS2	TR-SS1	TR-SS2
GCA_016725865.1	TR-SS1	TR-SS1	TR-SS2
GCA_016725465.1	TR-SS1	TR-SS2	TR-SS1
GCA_016725925.1	TR-SS1	TR-SS2	TR-SS1
GCA_039511225.1	TR-SS1	TR-SS1	TR-SS1
GCA_016726045.1	TR-SS2	TR-SS1	TR-SS1
GCA_013267375.1	TR-SS1	TR-SS1	TR-SS2
GCA_013267395.1	TR-SS2	TR-SS1	TR-SS1
GCA_039565655.1	BAL-TS	TR-SS2	TR-SS1
GCA_002205315.1	TR-SS1	TR-SS2	TR-SS2
GCA_013343095.1	TR-SS1	TR-SS1	TR-SS1
GCA_016728825.1	BAL-TS	TR-SS1	TR-SS2
GCA_022870085.1	TR-SS2	TR-SS1	TR-SS2
GCA_001457475.1	TR-SS1	TR-SS2	TR-SS1
GCA_013343135.1	BAL-TS	TR-SS1	TR-SS2
GCA_033096405.1	TR-SS1	TR-SS2	TR-SS2
GCA_008632635.1	TR-SS1	IMBAL-TS	TR-SS1
GCA_009035845.1	TR-SS1	IMBAL-TS	TR-SS2
GCA_020911985.1	TR-SS2	IMBAL-TS	TR-SS1
GCA_022870045.1	BAL-TS	IMBAL-TS	TR-SS1
GCA_014672755.1	TR-SS2	IMBAL-TS	TR-SS1
GCA_002055515.1	BAL-TS	TR-SS2	TR-SS1
GCA_021165835.1	TR-SS1	TR-SS1	TR-SS1
GCA_021165855.1	TR-SS1	TR-SS1	TR-SS1
GCA_900520355.1	TR-SS1	TR-SS2	TR-SS1
GCA_016939475.1	TR-SS2	TR-SS2	TR-SS2
GCA_000967305.2	TR-SS2	TR-SS2	TR-SS1
GCA_002443155.1	IMBAL-TS	TR-SS2	TR-SS2
GCA_958448115.1	TR-SS2	TR-SS1	TR-SS1
GCA_027595045.1	TR-SS1	TR-SS2	TR-SS1
GCA_010092625.1	TR-SS1	TR-SS1	TR-SS1
GCA_900445995.1	TR-SS1	TR-SS1	TR-SS2
GCA_038020885.1	TR-SS1	TR-SS2	TR-SS2
GCA_004102925.1	TR-SS1	TR-SS1	TR-SS1
GCA_016127655.1	BAL-TS	TR-SS2	TR-SS2
GCA_034424705.1	TR-SS1	TR-SS2	TR-SS1
GCA_019047105.1	BAL-TS	TR-SS2	IMBAL-TS
GCA_000025565.1	TR-SS1	TR-SS1	IMBAL-TS
GCA_018140965.1	TR-SS1	TR-SS1	IMBAL-TS
GCA_003019925.1	TR-SS1	TR-SS2	IMBAL-TS
GCA_000757785.1	TR-SS2	TR-SS1	IMBAL-TS
GCA_902387975.1	TR-SS1	TR-SS1	IMBAL-TS
GCA_001598855.1	BAL-TS	TR-SS1	IMBAL-TS
GCA_902729455.1	TR-SS2	TR-SS1	IMBAL-TS
GCA_009914515.1	BAL-TS	TR-SS1	IMBAL-TS
GCA_009914495.1	BAL-TS	TR-SS2	IMBAL-TS
GCA_025268675.1	TR-SS2	TR-SS2	IMBAL-TS
GCA_000241345.2	TR-SS1	TR-SS1	IMBAL-TS
GCA_024809495.1	TR-SS1	TR-SS1	IMBAL-TS
GCA_030536535.1	TR-SS2	TR-SS2	IMBAL-TS
GCA_030146765.1	TR-SS1	TR-SS1	IMBAL-TS

GCA_030147105.1	TR-SS2	TR-SS2	IMBAL-TS
GCA_003523365.1	BAL-TS	TR-SS2	IMBAL-TS
GCA_036010585.1	IMBAL-TS	TR-SS1	IMBAL-TS
GCA_036237355.1	BAL-TS	TR-SS1	IMBAL-TS
GCA_000385945.1	TR-SS1	TR-SS1	IMBAL-TS
GCA_036227025.1	TR-SS2	TR-SS1	IMBAL-TS
GCA_013364315.1	TR-SS1	TR-SS1	IMBAL-TS
GCA_003702755.1	BAL-TS	TR-SS1	TR-SS1
GCA_013321415.1	TR-SS2	TR-SS2	TR-SS2
GCA_028555185.1	TR-SS2	TR-SS2	IMBAL-TS
GCA_003699785.1	IMBAL-TS	IMBAL-TS	TR-SS2
GCA_017656535.1	TR-SS2	TR-SS1	IMBAL-TS
GCA_000807875.2	TR-SS1	TR-SS1	TR-SS1
GCA_029086895.1	TR-SS1	TR-SS2	IMBAL-TS
GCA_013608115.1	BAL-TS	IMBAL-TS	TR-SS1
GCA_032190835.1	TR-SS2	TR-SS1	IMBAL-TS
GCA_003416255.1	BAL-TS	IMBAL-TS	TR-SS1
GCA_003700905.1	TR-SS2	IMBAL-TS	TR-SS1
GCA_032198945.1	IMBAL-TS	TR-SS1	IMBAL-TS
GCA_022385255.1	TR-SS2	TR-SS2	IMBAL-TS
GCA_016803175.1	IMBAL-TS	TR-SS1	TR-SS1
GCA_029081345.1	TR-SS2	TR-SS2	IMBAL-TS
GCA_902829525.1	IMBAL-TS	IMBAL-TS	TR-SS2
GCA_900581015.1	TR-SS1	TR-SS1	TR-SS1
GCA_000981885.1	IMBAL-TS	IMBAL-TS	TR-SS2
GCA_003699955.1	BAL-TS	IMBAL-TS	TR-SS1
GCA_900580895.1	TR-SS2	TR-SS1	TR-SS1
GCA_034809025.1	IMBAL-TS	TR-SS1	IMBAL-TS
GCA_019042515.1	TR-SS1	IMBAL-TS	TR-SS1
GCA_032916665.1	TR-SS1	IMBAL-TS	IMBAL-TS
GCA_029081695.1	TR-SS1	TR-SS1	IMBAL-TS
GCA_021605965.1	IMBAL-TS	TR-SS2	TR-SS1
GCA_003590625.2	TR-SS2	TR-SS1	TR-SS1
GCA_032655185.1	TR-SS2	IMBAL-TS	IMBAL-TS
GCA_020813055.1	TR-SS1	TR-SS2	TR-SS1
GCA_000155995.1	TR-SS1	TR-SS1	IMBAL-TS
GCA_029086265.1	BAL-TS	TR-SS2	IMBAL-TS
GCA_010677205.1	BAL-TS	TR-SS1	IMBAL-TS
GCA_000172895.1	IMBAL-TS	TR-SS2	TR-SS2
GCA_002959435.1	BAL-TS	TR-SS2	IMBAL-TS
GCA_001475565.1	IMBAL-TS	TR-SS1	IMBAL-TS
GCA_001475535.1	IMBAL-TS	TR-SS2	IMBAL-TS
GCA_017921235.1	TR-SS2	TR-SS2	IMBAL-TS
GCA_029684855.1	TR-SS2	TR-SS1	IMBAL-TS
GCA_017920195.1	TR-SS1	TR-SS1	IMBAL-TS
GCA_001644805.1	TR-SS2	TR-SS1	TR-SS2
GCA_019880465.1	TR-SS1	TR-SS2	IMBAL-TS
GCA_001400345.1	TR-SS1	IMBAL-TS	TR-SS1
GCA_001293975.1	TR-SS1	IMBAL-TS	TR-SS1
GCA_000710015.2	TR-SS1	TR-SS1	IMBAL-TS
GCA_003416545.1	TR-SS2	IMBAL-TS	TR-SS1
GCA_003416565.1	TR-SS1	IMBAL-TS	TR-SS1
GCA_015865025.1	TR-SS1	TR-SS2	TR-SS1
GCA_003415895.1	TR-SS2	IMBAL-TS	TR-SS1
GCA_003416575.1	IMBAL-TS	IMBAL-TS	TR-SS1
GCA_003416425.1	IMBAL-TS	IMBAL-TS	TR-SS2
GCA_003701555.1	IMBAL-TS	IMBAL-TS	TR-SS1
GCA_003416245.1	IMBAL-TS	IMBAL-TS	TR-SS1
GCA_013321905.1	TR-SS1	TR-SS2	TR-SS1
GCA_024919385.1	IMBAL-TS	TR-SS1	IMBAL-TS
GCA_003416035.1	TR-SS1	IMBAL-TS	TR-SS1
GCA_003416455.1	IMBAL-TS	IMBAL-TS	TR-SS1
GCA_003701785.1	TR-SS2	IMBAL-TS	TR-SS1
GCA_003415685.1	TR-SS1	IMBAL-TS	TR-SS2
GCA_003416355.1	IMBAL-TS	IMBAL-TS	TR-SS2
GCA_021606145.1	TR-SS1	TR-SS1	TR-SS1
GCA_003416495.1	TR-SS1	IMBAL-TS	TR-SS1
GCA_003415975.1	IMBAL-TS	IMBAL-TS	TR-SS1
GCA_021609365.1	TR-SS1	TR-SS1	TR-SS1

GCA_041379965.2	IMBAL-TS	TR-SS1	TR-SS1
GCA_003700475.1	IMBAL-TS	IMBAL-TS	TR-SS1
GCA_021777455.1	IMBAL-TS	TR-SS1	TR-SS1
GCA_003416405.1	TR-SS1	IMBAL-TS	TR-SS1
GCA_003416505.1	TR-SS2	IMBAL-TS	TR-SS2
GCA_021864555.1	IMBAL-TS	TR-SS2	TR-SS1
GCA_019689265.1	TR-SS1	TR-SS2	TR-SS2
GCA_001400895.1	BAL-TS	IMBAL-TS	TR-SS1
GCA_023701545.1	BAL-TS	TR-SS1	TR-SS1
GCA_003416385.1	TR-SS1	IMBAL-TS	TR-SS2
GCA_019688775.1	IMBAL-TS	TR-SS2	TR-SS1
GCA_003416665.1	TR-SS1	IMBAL-TS	TR-SS2
GCA_016307745.1	IMBAL-TS	TR-SS2	TR-SS1
GCA_021459985.1	TR-SS2	TR-SS1	TR-SS1
GCA_002850575.1	IMBAL-TS	TR-SS2	IMBAL-TS
GCA_003416655.1	TR-SS2	IMBAL-TS	TR-SS1
GCA_001401235.1	TR-SS2	IMBAL-TS	TR-SS2
GCA_001400435.1	IMBAL-TS	IMBAL-TS	TR-SS1
GCA_021864855.1	IMBAL-TS	TR-SS1	TR-SS1
GCA_023278345.1	TR-SS1	TR-SS1	TR-SS2
GCA_019689235.1	TR-SS2	TR-SS1	TR-SS1
GCA_028749765.1	IMBAL-TS	TR-SS1	TR-SS2
GCA_021649995.1	IMBAL-TS	TR-SS2	IMBAL-TS
GCA_001535815.1	BAL-TS	IMBAL-TS	TR-SS2
GCA_019689375.1	TR-SS2	TR-SS1	TR-SS2
GCA_002981875.1	BAL-TS	IMBAL-TS	TR-SS2
GCA_019688895.1	TR-SS1	TR-SS2	TR-SS1
GCA_013322025.1	TR-SS1	TR-SS1	TR-SS1
GCA_013321135.1	TR-SS2	TR-SS1	TR-SS2
GCA_000177455.1	BAL-TS	TR-SS2	TR-SS2
GCA_019688965.1	TR-SS1	TR-SS2	TR-SS1
GCA_019689355.1	IMBAL-TS	TR-SS2	TR-SS2
GCA_964208085.1	TR-SS1	TR-SS2	IMBAL-TS
GCA_949769235.1	IMBAL-TS	TR-SS1	TR-SS1
GCA_003800545.1	IMBAL-TS	TR-SS1	IMBAL-TS
GCA_021459805.1	IMBAL-TS	TR-SS1	TR-SS2
GCA_013321855.1	TR-SS1	TR-SS2	TR-SS1
GCA_003415955.1	BAL-TS	IMBAL-TS	TR-SS2
GCA_013321045.1	TR-SS2	TR-SS1	TR-SS2
GCA_000567985.1	IMBAL-TS	TR-SS2	TR-SS1
GCA_021864635.1	BAL-TS	TR-SS1	TR-SS2
GCA_016307765.1	TR-SS1	TR-SS1	TR-SS2
GCA_007002795.1	TR-SS2	TR-SS2	TR-SS1
GCA_019689015.1	IMBAL-TS	TR-SS2	TR-SS1
GCA_905220785.1	BAL-TS	TR-SS1	TR-SS1
GCA_009648915.1	TR-SS1	TR-SS2	IMBAL-TS
GCA_021864915.1	TR-SS2	TR-SS1	TR-SS2
GCA_002019075.1	TR-SS2	TR-SS1	TR-SS1
GCA_902830195.1	BAL-TS	IMBAL-TS	TR-SS2
GCA_013322065.1	IMBAL-TS	TR-SS1	TR-SS2
GCA_000355635.2	TR-SS2	TR-SS1	TR-SS1
GCA_005233655.1	TR-SS1	TR-SS2	IMBAL-TS
GCA_002981405.1	BAL-TS	IMBAL-TS	TR-SS1
GCA_003700445.1	TR-SS2	IMBAL-TS	TR-SS2
GCA_013321175.1	TR-SS2	TR-SS1	TR-SS2
GCA_019041335.1	IMBAL-TS	IMBAL-TS	TR-SS2
GCA_000698825.1	BAL-TS	TR-SS2	TR-SS2
GCA_002939845.1	TR-SS1	TR-SS2	TR-SS1
GCA_013321295.1	TR-SS2	TR-SS1	TR-SS1
GCA_001400395.1	TR-SS1	IMBAL-TS	TR-SS1
GCA_026891515.1	TR-SS1	TR-SS2	IMBAL-TS
GCA_021606345.1	TR-SS1	TR-SS1	TR-SS1
GCA_904425455.1	IMBAL-TS	TR-SS1	IMBAL-TS
GCA_014841015.1	TR-SS1	TR-SS1	TR-SS2
GCA_024413095.1	TR-SS1	IMBAL-TS	TR-SS1
GCA_002019215.1	BAL-TS	TR-SS2	TR-SS2
GCA_003701105.1	BAL-TS	TR-SS2	TR-SS1
GCA_013321255.1	TR-SS2	TR-SS1	TR-SS2
GCA_013321215.1	IMBAL-TS	TR-SS2	TR-SS1

GCA_002891235.1	IMBAL-TS	TR-SS2	IMBAL-TS
GCA_943912745.1	BAL-TS	TR-SS1	TR-SS2
GCA_013321835.1	IMBAL-TS	TR-SS2	TR-SS1
GCA_002019375.1	BAL-TS	TR-SS1	TR-SS1
GCA_002270015.1	TR-SS1	IMBAL-TS	TR-SS1
GCA_013321715.1	TR-SS2	TR-SS2	TR-SS2
GCA_037136815.1	IMBAL-TS	TR-SS1	IMBAL-TS
GCA_019775675.1	IMBAL-TS	TR-SS1	TR-SS1
GCA_021606025.1	TR-SS1	TR-SS1	TR-SS2
GCA_003699855.1	IMBAL-TS	IMBAL-TS	TR-SS1
GCA_021606305.1	TR-SS2	TR-SS1	TR-SS2
GCA_902830915.1	TR-SS1	IMBAL-TS	TR-SS1
GCA_003703225.1	TR-SS1	IMBAL-TS	TR-SS1
GCA_003699255.1	IMBAL-TS	IMBAL-TS	TR-SS2
GCA_030370035.1	TR-SS2	TR-SS2	IMBAL-TS
GCA_003702985.1	TR-SS2	IMBAL-TS	TR-SS1
GCA_013320995.1	IMBAL-TS	TR-SS2	TR-SS1
GCA_021606045.1	TR-SS2	TR-SS1	TR-SS2
GCA_002019095.1	TR-SS1	TR-SS1	TR-SS1
GCA_021606265.1	BAL-TS	TR-SS2	TR-SS1
GCA_000959725.1	TR-SS1	IMBAL-TS	TR-SS2
GCA_002981475.1	TR-SS1	IMBAL-TS	TR-SS1
GCA_013321035.1	TR-SS2	TR-SS1	TR-SS1
GCA_032594455.1	TR-SS2	TR-SS1	TR-SS1
GCA_001400935.1	TR-SS2	IMBAL-TS	TR-SS1
GCA_013321635.1	TR-SS1	TR-SS1	TR-SS1
GCA_022026035.1	TR-SS1	IMBAL-TS	TR-SS2
GCA_013320955.1	TR-SS1	TR-SS2	TR-SS2
GCA_015682315.1	BAL-TS	TR-SS1	IMBAL-TS
GCA_013321375.1	BAL-TS	TR-SS2	TR-SS2
GCA_003699805.1	IMBAL-TS	TR-SS1	TR-SS2
GCA_013322155.1	TR-SS1	TR-SS2	TR-SS1
GCA_900706785.1	TR-SS2	TR-SS2	IMBAL-TS
GCA_021864665.1	TR-SS1	TR-SS1	TR-SS1
GCA_013322015.1	IMBAL-TS	TR-SS1	TR-SS1
GCA_022828345.1	TR-SS1	IMBAL-TS	TR-SS2
GCA_003700715.1	TR-SS2	IMBAL-TS	TR-SS1
GCA_001275735.1	IMBAL-TS	IMBAL-TS	TR-SS2
GCA_001417925.1	BAL-TS	TR-SS2	TR-SS1
GCA_000770155.1	IMBAL-TS	TR-SS1	IMBAL-TS
GCA_008180055.1	TR-SS2	TR-SS2	IMBAL-TS
GCA_016308215.1	IMBAL-TS	TR-SS1	TR-SS1
GCA_003700515.1	TR-SS1	TR-SS2	TR-SS1
GCA_013364135.1	TR-SS1	TR-SS1	IMBAL-TS
GCA_013912115.1	IMBAL-TS	TR-SS1	IMBAL-TS
GCA_004127335.1	IMBAL-TS	TR-SS2	IMBAL-TS
GCA_002931295.1	TR-SS1	IMBAL-TS	IMBAL-TS
GCA_015682715.1	BAL-TS	TR-SS2	IMBAL-TS
GCA_001373275.1	TR-SS1	TR-SS1	TR-SS1
GCA_002152055.1	IMBAL-TS	TR-SS1	IMBAL-TS
GCA_003701915.1	TR-SS2	IMBAL-TS	TR-SS2
GCA_015682655.1	BAL-TS	TR-SS1	IMBAL-TS
GCA_024925425.1	IMBAL-TS	TR-SS1	IMBAL-TS
GCA_002278295.1	IMBAL-TS	TR-SS1	IMBAL-TS
GCA_015682745.1	TR-SS2	TR-SS1	IMBAL-TS
GCA_009907135.1	IMBAL-TS	TR-SS1	IMBAL-TS
GCA_003986865.1	TR-SS2	TR-SS1	IMBAL-TS
GCA_020990965.1	IMBAL-TS	TR-SS1	TR-SS2
GCA_016307755.1	TR-SS1	TR-SS2	TR-SS2
GCA_004127365.1	IMBAL-TS	TR-SS1	IMBAL-TS
GCA_026622875.1	IMBAL-TS	TR-SS1	TR-SS1
GCA_001537985.1	IMBAL-TS	IMBAL-TS	TR-SS1
GCA_003985225.1	TR-SS1	TR-SS2	IMBAL-TS
GCA_013522775.1	TR-SS1	TR-SS2	TR-SS1
GCA_021271025.1	TR-SS1	TR-SS1	IMBAL-TS
GCA_002528975.1	TR-SS1	TR-SS2	IMBAL-TS
GCA_009727145.1	BAL-TS	TR-SS1	IMBAL-TS
GCA_000163275.1	TR-SS1	IMBAL-TS	TR-SS1
GCA_002278165.1	IMBAL-TS	TR-SS2	IMBAL-TS

GCA_029854985.1	BAL-TS	IMBAL-TS	TR-SS1
GCA_900581155.1	BAL-TS	TR-SS2	TR-SS2
GCA_002376205.1	TR-SS2	TR-SS1	TR-SS2
GCA_032104565.1	TR-SS2	TR-SS1	TR-SS2
GCA_025985525.1	TR-SS1	TR-SS1	TR-SS1
GCA_000935305.1	BAL-TS	TR-SS1	TR-SS1
GCA_001400675.1	IMBAL-TS	IMBAL-TS	TR-SS2
GCA_010592545.1	TR-SS1	TR-SS1	IMBAL-TS
GCA_007678515.1	TR-SS1	TR-SS1	TR-SS1
GCA_001294215.1	IMBAL-TS	IMBAL-TS	TR-SS2
GCA_900102665.1	TR-SS1	TR-SS2	TR-SS2
GCA_012986165.1	TR-SS2	TR-SS2	TR-SS1
GCA_900581375.1	IMBAL-TS	TR-SS1	TR-SS1
GCA_040195035.1	TR-SS1	TR-SS1	TR-SS1
GCA_900580555.1	IMBAL-TS	TR-SS2	TR-SS1
GCA_024925305.1	TR-SS1	TR-SS2	IMBAL-TS
GCA_024919475.1	BAL-TS	TR-SS1	IMBAL-TS
GCA_026241805.1	TR-SS1	TR-SS1	IMBAL-TS
GCA_013358225.1	IMBAL-TS	TR-SS2	TR-SS1
GCA_000755525.1	BAL-TS	TR-SS2	IMBAL-TS
GCA_004024145.1	TR-SS2	TR-SS1	IMBAL-TS
GCA_900581245.1	TR-SS2	TR-SS2	TR-SS1
GCA_013376815.1	TR-SS2	TR-SS1	IMBAL-TS
GCA_000967935.1	IMBAL-TS	TR-SS1	TR-SS1
GCA_018383465.1	TR-SS2	TR-SS1	TR-SS2
GCA_014489975.1	IMBAL-TS	TR-SS2	TR-SS1
GCA_003700015.1	TR-SS2	TR-SS1	TR-SS1
GCA_016307855.1	IMBAL-TS	TR-SS1	TR-SS2
GCA_034424355.1	TR-SS2	TR-SS2	TR-SS1
GCA_030544665.1	TR-SS1	TR-SS2	TR-SS1
GCA_012367015.1	IMBAL-TS	TR-SS1	IMBAL-TS
GCA_012367325.1	TR-SS2	TR-SS1	IMBAL-TS
GCA_003415785.1	TR-SS2	IMBAL-TS	TR-SS2
GCA_018388505.1	TR-SS2	TR-SS1	TR-SS2
GCA_900581105.1	BAL-TS	TR-SS2	TR-SS1
GCA_019334485.1	TR-SS1	IMBAL-TS	TR-SS1
GCA_012367595.1	IMBAL-TS	TR-SS1	IMBAL-TS
GCA_002318825.1	TR-SS1	TR-SS2	TR-SS2
GCA_006369965.1	TR-SS2	TR-SS1	TR-SS1
GCA_003700425.1	IMBAL-TS	IMBAL-TS	TR-SS1
GCA_037199295.1	IMBAL-TS	TR-SS2	IMBAL-TS
GCA_946478005.1	TR-SS1	TR-SS2	IMBAL-TS
GCA_002179795.1	IMBAL-TS	TR-SS1	TR-SS2
GCA_024919455.1	TR-SS2	TR-SS1	IMBAL-TS
GCA_004319505.1	TR-SS1	TR-SS2	TR-SS1
GCA_026241835.1	IMBAL-TS	TR-SS1	IMBAL-TS
GCA_002318875.1	BAL-TS	TR-SS2	TR-SS2
GCA_001538055.1	TR-SS1	IMBAL-TS	TR-SS1
GCA_016308045.1	TR-SS1	TR-SS2	TR-SS1
GCA_031556935.1	TR-SS1	TR-SS2	IMBAL-TS
GCA_020406575.2	TR-SS1	TR-SS1	IMBAL-TS
GCA_018598385.1	BAL-TS	TR-SS2	IMBAL-TS
GCA_017745315.1	TR-SS1	TR-SS1	TR-SS1
GCA_018831445.1	TR-SS2	TR-SS2	TR-SS2
GCA_001886315.1	TR-SS2	TR-SS2	TR-SS2
GCA_017742795.1	IMBAL-TS	TR-SS2	TR-SS1
GCA_025987725.1	TR-SS1	TR-SS2	TR-SS1
GCA_017745345.1	TR-SS2	TR-SS2	TR-SS2
GCA_020917325.1	IMBAL-TS	IMBAL-TS	TR-SS1
GCA_006369915.1	TR-SS1	TR-SS1	TR-SS1
GCA_003701175.1	BAL-TS	TR-SS1	TR-SS1
GCA_900234425.1	BAL-TS	TR-SS1	TR-SS2
GCA_003999445.1	TR-SS1	TR-SS2	TR-SS1
GCA_000791705.1	TR-SS2	TR-SS2	TR-SS1
GCA_016308015.1	TR-SS1	TR-SS1	TR-SS1
GCA_000808735.2	IMBAL-TS	TR-SS2	TR-SS2
GCA_002759155.2	TR-SS2	TR-SS2	TR-SS1
GCA_025560205.1	IMBAL-TS	TR-SS1	TR-SS1
GCA_025560005.1	IMBAL-TS	TR-SS1	TR-SS1

GCA_001010435.1	BAL-TS	TR-SS1	TR-SS1
GCA_000808715.2	TR-SS1	TR-SS1	TR-SS2
GCA_003111925.1	TR-SS1	TR-SS1	TR-SS1
GCA_000145765.1	IMBAL-TS	TR-SS1	TR-SS1
GCA_000382585.2	TR-SS2	TR-SS2	IMBAL-TS
GCA_038396405.1	TR-SS2	TR-SS1	IMBAL-TS
GCA_000732705.1	TR-SS2	TR-SS2	TR-SS1
GCA_004376015.1	IMBAL-TS	TR-SS1	TR-SS2
GCA_008693825.1	TR-SS1	TR-SS1	TR-SS2
GCA_004376045.1	TR-SS2	TR-SS1	TR-SS1
GCA_036861355.1	IMBAL-TS	IMBAL-TS	TR-SS2
GCA_902829495.1	IMBAL-TS	IMBAL-TS	TR-SS1
GCA_036861395.1	TR-SS1	IMBAL-TS	TR-SS1
GCA_001526345.1	TR-SS1	IMBAL-TS	TR-SS2
GCA_032002685.1	IMBAL-TS	TR-SS2	TR-SS1
GCA_902829635.1	IMBAL-TS	IMBAL-TS	TR-SS1
GCA_001530045.1	IMBAL-TS	IMBAL-TS	TR-SS1
GCA_001529825.1	IMBAL-TS	IMBAL-TS	TR-SS1
GCA_003854435.1	TR-SS1	IMBAL-TS	TR-SS2
GCA_001522525.1	BAL-TS	IMBAL-TS	TR-SS1
GCA_001531525.1	BAL-TS	IMBAL-TS	TR-SS1
GCA_902830905.1	TR-SS1	IMBAL-TS	TR-SS2
GCA_030482245.1	IMBAL-TS	IMBAL-TS	TR-SS1
GCA_036862195.1	TR-SS1	IMBAL-TS	TR-SS2
GCA_001528685.1	IMBAL-TS	IMBAL-TS	TR-SS1
GCA_003858175.1	TR-SS1	IMBAL-TS	TR-SS1
GCA_003858185.1	TR-SS1	IMBAL-TS	TR-SS1
GCA_016008885.1	TR-SS1	TR-SS1	TR-SS2
GCA_001533445.1	TR-SS1	IMBAL-TS	TR-SS2
GCA_001521975.1	TR-SS1	IMBAL-TS	TR-SS1
GCA_042604585.1	TR-SS2	IMBAL-TS	TR-SS2
GCA_001971465.2	BAL-TS	TR-SS1	TR-SS2
GCA_001526905.1	IMBAL-TS	IMBAL-TS	TR-SS1
GCA_022549195.1	TR-SS1	IMBAL-TS	TR-SS1
GCA_004377645.1	BAL-TS	IMBAL-TS	TR-SS1
GCA_036862355.1	TR-SS1	IMBAL-TS	TR-SS1
GCA_025244785.1	TR-SS1	IMBAL-TS	TR-SS2
GCA_001530415.1	TR-SS2	IMBAL-TS	TR-SS1
GCA_001522985.1	BAL-TS	IMBAL-TS	TR-SS2
GCA_001522805.1	TR-SS2	IMBAL-TS	TR-SS2
GCA_016505965.1	IMBAL-TS	IMBAL-TS	TR-SS1
GCA_019969865.1	TR-SS1	TR-SS1	TR-SS2
GCA_041019735.1	TR-SS1	IMBAL-TS	TR-SS1
GCA_001522925.1	IMBAL-TS	IMBAL-TS	TR-SS1
GCA_001523985.1	TR-SS1	IMBAL-TS	TR-SS1
GCA_001533295.1	IMBAL-TS	IMBAL-TS	TR-SS1
GCA_002980955.1	TR-SS2	IMBAL-TS	TR-SS1
GCA_001522875.1	IMBAL-TS	IMBAL-TS	TR-SS1
GCA_030482765.1	TR-SS1	IMBAL-TS	TR-SS1
GCA_001529525.1	IMBAL-TS	IMBAL-TS	TR-SS1
GCA_001521915.1	TR-SS2	IMBAL-TS	TR-SS1
GCA_001525925.1	IMBAL-TS	IMBAL-TS	TR-SS1
GCA_001522945.1	TR-SS1	IMBAL-TS	TR-SS1
GCA_030811185.1	TR-SS2	TR-SS2	TR-SS2
GCA_013401495.1	TR-SS1	TR-SS1	TR-SS2
GCA_001530205.1	TR-SS2	IMBAL-TS	TR-SS2
GCA_036861555.1	IMBAL-TS	IMBAL-TS	TR-SS2
GCA_000406065.1	BAL-TS	TR-SS1	IMBAL-TS
GCA_016026015.1	IMBAL-TS	IMBAL-TS	TR-SS2
GCA_038040145.1	TR-SS2	IMBAL-TS	TR-SS1
GCA_038040075.1	TR-SS2	IMBAL-TS	TR-SS2
GCA_017700795.1	TR-SS1	TR-SS1	IMBAL-TS
GCA_031500285.1	BAL-TS	IMBAL-TS	TR-SS2
GCA_001266515.1	BAL-TS	TR-SS1	TR-SS2
GCA_014141625.1	TR-SS2	IMBAL-TS	TR-SS2
GCA_019781985.1	TR-SS1	IMBAL-TS	IMBAL-TS
GCA_001264385.1	TR-SS1	TR-SS1	TR-SS1
GCA_902829355.1	IMBAL-TS	IMBAL-TS	TR-SS1
GCA_038040095.1	IMBAL-TS	IMBAL-TS	TR-SS1

GCA_002269785.1	IMBAL-TS	IMBAL-TS	TR-SS1
GCA_004377105.1	IMBAL-TS	IMBAL-TS	TR-SS1
GCA_014142075.1	TR-SS2	IMBAL-TS	TR-SS1
GCA_001266535.1	TR-SS1	TR-SS2	TR-SS2
GCA_025000365.1	IMBAL-TS	TR-SS2	IMBAL-TS
GCA_001264295.1	TR-SS1	TR-SS1	TR-SS1
GCA_001264275.1	TR-SS1	TR-SS1	TR-SS1
GCA_019782455.1	TR-SS1	IMBAL-TS	IMBAL-TS
GCA_019104085.1	TR-SS1	TR-SS2	TR-SS2
GCA_019781485.1	TR-SS1	IMBAL-TS	IMBAL-TS
GCA_019782485.1	TR-SS1	IMBAL-TS	IMBAL-TS
GCA_001264355.1	IMBAL-TS	TR-SS1	TR-SS1
GCA_001264425.1	BAL-TS	TR-SS1	TR-SS2
GCA_003031365.1	IMBAL-TS	TR-SS1	TR-SS1
GCA_035011625.1	IMBAL-TS	TR-SS2	TR-SS2
GCA_001643295.1	TR-SS2	TR-SS1	TR-SS2
GCA_018598265.1	BAL-TS	TR-SS1	IMBAL-TS
GCA_026241795.1	IMBAL-TS	TR-SS2	IMBAL-TS
GCA_003410095.1	TR-SS1	TR-SS2	TR-SS2
GCA_030011855.1	TR-SS1	TR-SS1	IMBAL-TS
GCA_014141995.1	TR-SS2	IMBAL-TS	TR-SS2
GCA_002940235.1	IMBAL-TS	TR-SS2	TR-SS1
GCA_040195245.1	TR-SS2	TR-SS2	TR-SS1
GCA_001052915.1	BAL-TS	IMBAL-TS	TR-SS1
GCA_035012925.1	TR-SS2	TR-SS1	TR-SS2
GCA_040194775.1	BAL-TS	TR-SS1	TR-SS1
GCA_905123105.1	TR-SS2	TR-SS2	TR-SS1
GCA_019782525.1	TR-SS1	IMBAL-TS	IMBAL-TS
GCA_905220695.1	TR-SS1	TR-SS1	TR-SS1
GCA_001264345.1	TR-SS1	TR-SS2	TR-SS1
GCA_018598425.1	IMBAL-TS	TR-SS2	IMBAL-TS
GCA_001530005.1	TR-SS2	IMBAL-TS	TR-SS2
GCA_019781245.1	BAL-TS	IMBAL-TS	IMBAL-TS
GCA_019782705.1	TR-SS2	IMBAL-TS	IMBAL-TS
GCA_019782685.1	TR-SS1	IMBAL-TS	IMBAL-TS
GCA_019782605.1	IMBAL-TS	IMBAL-TS	IMBAL-TS
GCA_019781405.1	TR-SS2	IMBAL-TS	IMBAL-TS
GCA_902829615.1	BAL-TS	IMBAL-TS	TR-SS1
GCA_019781695.1	TR-SS2	IMBAL-TS	IMBAL-TS
GCA_019783045.1	TR-SS1	IMBAL-TS	IMBAL-TS
GCA_028749625.1	TR-SS1	TR-SS1	TR-SS1
GCA_019781515.1	TR-SS1	IMBAL-TS	IMBAL-TS
GCA_019783075.1	TR-SS1	IMBAL-TS	IMBAL-TS
GCA_019400155.1	IMBAL-TS	IMBAL-TS	TR-SS1
GCA_001530805.1	TR-SS1	IMBAL-TS	TR-SS2
GCA_019781885.1	TR-SS2	IMBAL-TS	IMBAL-TS
GCA_011761675.1	BAL-TS	TR-SS2	TR-SS1
GCA_002259465.1	TR-SS1	IMBAL-TS	IMBAL-TS
GCA_002259505.1	BAL-TS	IMBAL-TS	IMBAL-TS
GCA_019781925.1	BAL-TS	IMBAL-TS	IMBAL-TS
GCA_019781325.1	TR-SS1	IMBAL-TS	IMBAL-TS
GCA_019781765.1	TR-SS1	IMBAL-TS	IMBAL-TS
GCA_001529685.1	TR-SS2	IMBAL-TS	TR-SS1
GCA_019781645.1	TR-SS2	IMBAL-TS	IMBAL-TS
GCA_019781545.1	IMBAL-TS	IMBAL-TS	IMBAL-TS
GCA_011927495.1	IMBAL-TS	TR-SS1	TR-SS1
GCA_019782445.1	TR-SS1	IMBAL-TS	IMBAL-TS
GCA_003702965.1	TR-SS2	IMBAL-TS	TR-SS2
GCA_019782985.1	TR-SS1	IMBAL-TS	IMBAL-TS
GCA_002940365.1	IMBAL-TS	TR-SS1	TR-SS2
GCA_030519585.1	IMBAL-TS	TR-SS1	IMBAL-TS
GCA_000306055.1	IMBAL-TS	TR-SS1	TR-SS1
GCA_900580415.1	IMBAL-TS	TR-SS2	TR-SS2
GCA_018598295.1	BAL-TS	TR-SS1	IMBAL-TS
GCA_001620005.1	IMBAL-TS	IMBAL-TS	IMBAL-TS
GCA_015351035.1	IMBAL-TS	TR-SS2	IMBAL-TS
GCA_019782265.1	IMBAL-TS	IMBAL-TS	IMBAL-TS
GCA_900581435.1	TR-SS1	TR-SS1	TR-SS1
GCA_040201855.2	TR-SS1	TR-SS2	TR-SS1

GCA_003701235.1	IMBAL-TS	TR-SS1	TR-SS1
GCA_900580825.1	TR-SS2	TR-SS2	TR-SS1
GCA_036349535.1	IMBAL-TS	TR-SS1	TR-SS1
GCA_034808185.1	IMBAL-TS	TR-SS2	TR-SS1
GCA_900580875.1	TR-SS1	TR-SS1	TR-SS2
GCA_004124655.1	TR-SS1	TR-SS2	TR-SS2
GCA_905220745.1	TR-SS1	TR-SS1	TR-SS2
GCA_900581305.1	BAL-TS	TR-SS1	TR-SS2
GCA_019782945.1	TR-SS1	IMBAL-TS	IMBAL-TS
GCA_900580705.1	TR-SS2	TR-SS1	TR-SS2
GCA_004124735.1	BAL-TS	TR-SS1	TR-SS2
GCA_900581085.1	TR-SS2	TR-SS2	TR-SS2
GCA_003993355.1	IMBAL-TS	TR-SS2	TR-SS1
GCA_019782855.1	TR-SS1	IMBAL-TS	IMBAL-TS
GCA_900581325.1	IMBAL-TS	TR-SS1	TR-SS2
GCA_037136035.1	IMBAL-TS	TR-SS1	IMBAL-TS
GCA_009898315.1	TR-SS2	TR-SS1	TR-SS1
GCA_900580665.1	TR-SS2	TR-SS1	TR-SS2
GCA_900580465.1	IMBAL-TS	TR-SS2	TR-SS1
GCA_009898365.1	TR-SS1	TR-SS1	TR-SS2
GCA_004124865.1	TR-SS1	TR-SS1	TR-SS1
GCA_019782565.1	TR-SS2	IMBAL-TS	IMBAL-TS
GCA_013449385.1	IMBAL-TS	TR-SS2	IMBAL-TS
GCA_036349475.1	TR-SS2	TR-SS2	TR-SS2
GCA_037136335.1	TR-SS2	TR-SS2	IMBAL-TS
GCA_019685595.1	IMBAL-TS	IMBAL-TS	IMBAL-TS
GCA_900580435.1	BAL-TS	TR-SS2	TR-SS2
GCA_040203135.1	BAL-TS	TR-SS1	TR-SS1
GCA_000808435.1	TR-SS1	TR-SS1	IMBAL-TS
GCA_020406855.1	TR-SS1	TR-SS2	IMBAL-TS
GCA_022507295.1	BAL-TS	TR-SS1	IMBAL-TS
GCA_000749855.1	IMBAL-TS	TR-SS1	IMBAL-TS
GCA_037136655.1	IMBAL-TS	TR-SS2	IMBAL-TS
GCA_030388995.2	BAL-TS	TR-SS1	TR-SS2
GCA_001039055.1	TR-SS1	TR-SS1	IMBAL-TS
GCA_035066895.1	TR-SS1	TR-SS1	IMBAL-TS
GCA_030388865.2	IMBAL-TS	TR-SS1	TR-SS2
GCA_036961575.1	BAL-TS	TR-SS2	IMBAL-TS
GCA_037136885.1	TR-SS1	TR-SS1	IMBAL-TS
GCA_035066985.1	IMBAL-TS	TR-SS1	IMBAL-TS
GCA_020406635.1	IMBAL-TS	TR-SS1	IMBAL-TS
GCA_035066845.1	TR-SS2	TR-SS1	IMBAL-TS
GCA_037135995.1	IMBAL-TS	TR-SS1	IMBAL-TS
GCA_018598625.1	BAL-TS	TR-SS1	IMBAL-TS
GCA_037136515.1	IMBAL-TS	TR-SS1	IMBAL-TS
GCA_021049225.1	TR-SS1	TR-SS2	IMBAL-TS
GCA_040225055.1	TR-SS1	TR-SS1	IMBAL-TS
GCA_005771575.1	TR-SS1	TR-SS2	IMBAL-TS
GCA_001718395.1	TR-SS2	IMBAL-TS	TR-SS1
GCA_000758345.1	IMBAL-TS	TR-SS2	IMBAL-TS
GCA_037136475.1	TR-SS2	TR-SS1	IMBAL-TS
GCA_013321275.1	TR-SS1	TR-SS2	TR-SS1
GCA_020520265.1	BAL-TS	TR-SS2	IMBAL-TS
GCA_009765495.1	TR-SS1	TR-SS1	TR-SS2
GCA_002251605.3	TR-SS2	TR-SS1	TR-SS1
GCA_900581265.1	TR-SS1	TR-SS2	TR-SS2
GCA_900580635.1	TR-SS1	TR-SS2	TR-SS1
GCA_036350005.1	TR-SS1	TR-SS2	TR-SS2
GCA_016307675.1	IMBAL-TS	TR-SS2	TR-SS1
GCA_000808495.1	TR-SS1	TR-SS1	IMBAL-TS
GCA_900581355.1	IMBAL-TS	TR-SS1	TR-SS1
GCA_001642795.1	IMBAL-TS	TR-SS1	TR-SS1
GCA_900581395.1	TR-SS2	TR-SS1	TR-SS1
GCA_036349785.1	IMBAL-TS	TR-SS1	TR-SS2
GCA_036349765.1	TR-SS1	TR-SS1	TR-SS2
GCA_036349835.1	IMBAL-TS	TR-SS1	TR-SS1
GCA_014156405.1	IMBAL-TS	TR-SS1	IMBAL-TS
GCA_036349845.1	IMBAL-TS	TR-SS2	TR-SS1
GCA_036349715.1	BAL-TS	TR-SS1	TR-SS2

GCA_036663515.1	TR-SS2	TR-SS2	IMBAL-TS
GCA_003363805.1	IMBAL-TS	TR-SS1	TR-SS2
GCA_036349255.1	TR-SS1	TR-SS1	TR-SS1
GCA_001013485.1	TR-SS2	TR-SS1	TR-SS2
GCA_007680075.1	TR-SS1	TR-SS1	TR-SS2
GCA_036349955.1	TR-SS2	TR-SS1	TR-SS1
GCA_003602375.1	TR-SS1	TR-SS2	TR-SS1
GCA_905123915.1	IMBAL-TS	TR-SS1	TR-SS2
GCA_003363965.1	IMBAL-TS	TR-SS1	TR-SS2
GCA_036349345.1	TR-SS1	TR-SS1	TR-SS1
GCA_019782065.1	TR-SS2	IMBAL-TS	IMBAL-TS
GCA_025962135.1	TR-SS1	TR-SS1	TR-SS1
GCA_020879215.1	TR-SS1	TR-SS1	TR-SS1
GCA_019781905.1	BAL-TS	IMBAL-TS	IMBAL-TS
GCA_019782135.1	BAL-TS	IMBAL-TS	IMBAL-TS
GCA_019782185.1	TR-SS1	IMBAL-TS	IMBAL-TS
GCA_019781945.1	TR-SS1	IMBAL-TS	IMBAL-TS
GCA_019782045.1	TR-SS1	IMBAL-TS	IMBAL-TS
GCA_019782365.1	TR-SS1	IMBAL-TS	IMBAL-TS
GCA_040631315.1	TR-SS1	TR-SS1	IMBAL-TS
GCA_019782005.1	TR-SS1	IMBAL-TS	IMBAL-TS
GCA_019782325.1	IMBAL-TS	IMBAL-TS	IMBAL-TS
GCA_013408025.1	TR-SS1	TR-SS2	TR-SS2
GCA_014199105.1	BAL-TS	TR-SS2	TR-SS1
GCA_014195715.1	TR-SS1	TR-SS1	TR-SS2
GCA_020812955.1	IMBAL-TS	TR-SS2	TR-SS2
GCA_013523015.1	IMBAL-TS	TR-SS2	TR-SS2
GCA_014199755.1	TR-SS2	TR-SS1	TR-SS2
GCA_019104065.1	TR-SS1	TR-SS2	TR-SS1
GCA_025881995.1	TR-SS1	TR-SS2	IMBAL-TS
GCA_003700105.1	TR-SS1	TR-SS1	TR-SS1
GCA_003700855.1	IMBAL-TS	TR-SS1	TR-SS2
GCA_025882585.1	TR-SS1	TR-SS2	IMBAL-TS
GCA_000808215.1	TR-SS1	TR-SS2	IMBAL-TS
GCA_005233555.1	TR-SS1	TR-SS1	TR-SS1
GCA_014838925.1	TR-SS1	TR-SS1	TR-SS1
GCA_013523075.1	TR-SS1	TR-SS2	TR-SS1
GCA_025881925.1	IMBAL-TS	TR-SS2	IMBAL-TS
GCA_023497985.1	TR-SS1	TR-SS2	TR-SS1
GCA_001549825.1	TR-SS1	TR-SS1	TR-SS1
GCA_001476115.1	TR-SS1	TR-SS2	IMBAL-TS
GCA_002361025.1	BAL-TS	TR-SS2	TR-SS1
GCA_000811965.1	TR-SS1	TR-SS1	TR-SS1
GCA_025882025.1	IMBAL-TS	TR-SS1	IMBAL-TS
GCA_001475885.1	TR-SS1	TR-SS1	IMBAL-TS
GCA_001549735.1	TR-SS2	TR-SS1	TR-SS2
GCA_037136455.1	TR-SS1	TR-SS1	IMBAL-TS
GCA_002916335.1	IMBAL-TS	TR-SS2	TR-SS2
GCA_963669685.1	IMBAL-TS	TR-SS1	TR-SS1
GCA_001901665.1	TR-SS1	TR-SS2	TR-SS2
GCA_002954185.1	TR-SS1	TR-SS1	TR-SS2
GCA_025385085.1	IMBAL-TS	TR-SS1	TR-SS1
GCA_025385235.1	TR-SS2	TR-SS2	TR-SS1
GCA_000175135.1	IMBAL-TS	TR-SS2	TR-SS2
GCA_019464635.1	IMBAL-TS	TR-SS1	IMBAL-TS
GCA_019444095.1	IMBAL-TS	TR-SS2	IMBAL-TS
GCA_001610915.1	TR-SS1	TR-SS1	TR-SS2
GCA_000786915.2	TR-SS1	TR-SS1	TR-SS2
GCA_037135635.1	IMBAL-TS	TR-SS1	IMBAL-TS
GCA_032088135.1	IMBAL-TS	TR-SS1	TR-SS1
GCA_000006725.1	IMBAL-TS	TR-SS1	TR-SS1
GCA_019739115.1	IMBAL-TS	TR-SS1	IMBAL-TS
GCA_002898475.1	TR-SS2	TR-SS2	TR-SS2
GCA_041475655.1	IMBAL-TS	TR-SS2	TR-SS1
GCA_000506905.2	TR-SS1	TR-SS1	TR-SS2
GCA_009832785.1	IMBAL-TS	TR-SS2	TR-SS2
GCA_001526205.1	IMBAL-TS	IMBAL-TS	TR-SS1
GCA_002894765.1	BAL-TS	TR-SS1	TR-SS1
GCA_000710695.1	TR-SS2	TR-SS1	TR-SS1

GCA_001299555.1	TR-SS1	TR-SS2	TR-SS2
GCA_002906055.1	TR-SS1	TR-SS1	TR-SS2
GCA_001696855.1	IMBAL-TS	TR-SS1	TR-SS1
GCA_000749995.1	TR-SS1	TR-SS2	TR-SS1
GCA_001696845.1	BAL-TS	TR-SS1	TR-SS1
GCA_002501565.1	BAL-TS	TR-SS1	TR-SS1
GCA_002894775.1	TR-SS1	TR-SS1	TR-SS2
GCA_003860765.1	IMBAL-TS	TR-SS1	TR-SS2
GCA_000710135.3	IMBAL-TS	TR-SS1	TR-SS2
GCA_008271875.1	TR-SS1	TR-SS2	TR-SS2
GCA_020520245.1	BAL-TS	TR-SS1	IMBAL-TS
GCA_001587155.1	IMBAL-TS	TR-SS1	TR-SS1
GCA_003860705.1	IMBAL-TS	TR-SS2	TR-SS2
GCA_000825825.2	IMBAL-TS	TR-SS1	TR-SS1
GCA_013320895.1	TR-SS1	TR-SS1	TR-SS1
GCA_001644815.1	TR-SS2	TR-SS1	TR-SS2
GCA_000825885.2	IMBAL-TS	TR-SS2	TR-SS1
GCA_013320905.1	TR-SS1	TR-SS1	TR-SS1
GCA_013320875.1	IMBAL-TS	TR-SS1	TR-SS2
GCA_017700745.1	IMBAL-TS	TR-SS1	IMBAL-TS
GCA_013320925.1	IMBAL-TS	TR-SS2	TR-SS1
GCA_023212925.1	TR-SS1	TR-SS1	IMBAL-TS
GCA_016642095.1	TR-SS1	TR-SS1	IMBAL-TS
GCA_023507825.1	IMBAL-TS	TR-SS1	IMBAL-TS
GCA_030269105.1	BAL-TS	TR-SS2	IMBAL-TS
GCA_024343685.1	TR-SS2	TR-SS2	IMBAL-TS
GCA_030269145.1	TR-SS2	TR-SS2	IMBAL-TS
GCA_019718835.1	BAL-TS	TR-SS1	TR-SS2
GCA_014861545.1	IMBAL-TS	IMBAL-TS	IMBAL-TS
GCA_019718875.1	TR-SS2	TR-SS1	TR-SS2
GCA_002411725.1	TR-SS1	IMBAL-TS	IMBAL-TS
GCA_019718895.1	BAL-TS	TR-SS1	TR-SS1
GCA_000406225.1	TR-SS2	TR-SS2	IMBAL-TS
GCA_012985915.1	TR-SS1	TR-SS1	TR-SS1
GCA_026967555.1	BAL-TS	IMBAL-TS	TR-SS2
