# Supplementary material for: Prediction of soil probiotics based on foundation model representation enhancement and stacked aggregation classifier
Source: Brief Bioinform. 2025 Oct 29;26(5):bbaf567. doi: 10.1093/bib/bbaf567 (PMC12570017; doi:10.1093/bib/bbaf567)
Supplement: Supplementary_Table_S3_R2_bbaf567 [file supplementary_table_s3_r2_bbaf567.pdf]

Supplementary Table S3. Hyperparameter settings of three foundation models.

Foundation model	Hyperparameter	Setting
Nucleotide Transformer-50M	hidden_size	512
	num_hidden_layers	12
	num_attention_heads	16
	intermediate_size	2048
	initializer_range	0.02
	hidden_dropout_prob	0
	attention_probs_dropout_prob	0
	position_embedding_type	rotary
	max_position_embeddings	2050
	mask_token_id	2
	pad_token_id	1
	vocab_size	4107
	emb_layer_norm_before	FALSE
	esmfold_config	NULL
	is_folding_model	FALSE
	tie_word_embeddings	FALSE
	torch_dtype	float32
	transformers_version	4.32.0.dev0
	use_cache	FALSE
DNABERT-2-117M	transformers_version	4.28.0
	torch_dtype	float32
	hidden_size	768
	num_hidden_layers	12
	num_attention_heads	12
	intermediate_size	3072
	hidden_act	gelu
	initializer_range	0.02
	layer_norm_eps	1e-12
	attention_probs_dropout_prob	0
	hidden_dropout_prob	0.1
	classifier_dropout	NULL
	position_embedding_type	absolute
	max_position_embeddings	512
	alibi_starting_size	512
	type_vocab_size	2
	vocab_size	4096
	gradient_checkpointing	FALSE
	use_cache	TRUE
	model_type	StripedHyena
	torch_dtype	bfloat16
	vocab_size	512
	max_seqlen	8192
	hidden_size	4096
	num_layers	32
	num_attention_heads	32
	num_filters	4096
	inner_mlp_size	10928
	mlp_activation	gelu
	rotary_emb_base	10000
	rotary_emb_scaling_factor	1
	short_filter_length	3
	state_size	8
	column_split	FALSE
	column_split_hyena	TRUE

	hyena_layer_idxs	[0,1,2,3,4,5,6,7,8,9,10,11,12,13,14,15,16,17,18,19,20,21,22,23,24,25,26,27,28,29,30,31]
	split_k0	TRUE
EVO-7B	smeared_gqa	FALSE
	tie_embeddings	TRUE
	use_cache	TRUE
	use_flash_attn	TRUE
	use_flash_depthwise	FALSE
	use_flash_rmsnorm	FALSE
	use_flashfft	FALSE
	use_interpolated_rotary_pos_emb	FALSE
	eps	1e-6
	final_norm	TRUE
	inference_mode	FALSE
	log_intermediate_values	FALSE
	make_vocab_size_divisible_by	8
	mha_out_proj_bias	TRUE
	qkv_proj_bias	TRUE
	prefill_style	fft
	proj_groups	1
	short_filter_bias	TRUE
	transformers_version	NULL
