# Supplementary material for: Prediction of soil probiotics based on foundation model representation enhancement and stacked aggregation classifier
Source: Brief Bioinform. 2025 Oct 29;26(5):bbaf567. doi: 10.1093/bib/bbaf567 (PMC12570017; doi:10.1093/bib/bbaf567)
Supplement: Supplementary_Table_S4_R2_bbaf567 [file supplementary_table_s4_r2_bbaf567.pdf]

Supplementary Table S4. Hyperparameter settings of XGBoost and LR.

Experimal Group	Sub-model	Hyperparameter	Setting	
			Nucleotide Transformer-50M	EVO-7B
1	XGBoost (first-level model)	max_depth	5	5
		learning_rate	0.2	0.2
		n_estimators	300	300
	LR (second-level model)	C	0.1	0.1
		penalty	"l2"	"l2"
2	XGBoost (first-level model)	max_depth	6	4
		learning_rate	0.05	0.005
		n_estimators	400	700
	LR (second-level model)	C	0.02	0.01
		penalty	"l2"	"l2"
3	XGBoost (first-level model)	max_depth	8	5
		learning_rate	0.05	0.05
		n_estimators	200	200
	LR (second-level model)	C	0.01	0.01
		penalty	"l2"	"l2"
