# Supplementary material for: Prediction of soil probiotics based on foundation model representation enhancement and stacked aggregation classifier
Source: Brief Bioinform. 2025 Oct 29;26(5):bbaf567. doi: 10.1093/bib/bbaf567 (PMC12570017; doi:10.1093/bib/bbaf567)
Supplement: Supplementary_Table_S5_R2_bbaf567 [file supplementary_table_s5_r2_bbaf567.pdf]

Supplementary Table S5. Genes and their KO identifiers of GCA_000242855.2.

Gene_ID	KO	Threshold	Score	*e* _value	KO_definition
CP004065.1_1	K03536	40.13	122.8	4.5e-36	ribonuclease P protein component [EC:3.1.26.5]
CP004065.1_2	K03217	121.07	285.5	1.3e-85	YidC/Oxa1 family membrane protein insertase
CP004065.1_3	K06346	71.00	232.4	1.6e-69	spoIIIJ-associated protein
CP004065.1_4	K03650	165.63	542.6	3.6e-163	tRNA modification GTPase [EC:3.6.-.-]
CP004065.1_5	K03495	422.67	1029.1	4.6e-310	tRNA uridine 5-carboxymethylaminomethyl modification enzyme
CP004065.1_6	K03501	59.80	253.2	8.7e-76	16S rRNA (guanine527-N7)-methyltransferase [EC:2.1.1.170]
CP004065.1_7	K03497	54.23	235.7	2.2e-70	ParB family transcriptional regulator, chromosome partitioning protein
CP004065.1_8	K03496	131.53	354.6	2.1e-106	chromosome partitioning protein
CP004065.1_9	K03497	54.23	274.7	3.3e-82	ParB family transcriptional regulator, chromosome partitioning protein
CP004065.1_15	K06942	430.83	561.4	4.9e-169	ribosome-binding ATPase
CP004065.1_16	K02990	24.13	102.9	3.7e-30	small subunit ribosomal protein S6
CP004065.1_17	K03111	28.83	166.1	3.1e-49	single-strand DNA-binding protein
CP004065.1_18	K02963	75.13	108.1	1.2e-31	small subunit ribosomal protein S18
CP004065.1_19	K13530	199.90	294.1	1.8e-88	AraC family transcriptional regulator, regulatory protein of adaptative response / methylphosphotriester-DNA alkyltransferase methyltransferase [EC:2.1.1.-]
CP004065.1_20	K13531	197.13	256.9	1.7e-77	methylated-DNA-[protein]-cysteine S-methyltransferase [EC:2.1.1.63]
CP004065.1_21	K01142	228.27	383.4	4.1e-115	exodeoxyribonuclease III [EC:3.1.11.2]
CP004065.1_24	K08369	358.47	421.5	8.9e-127	MFS transporter, putative metabolite:H+ symporter
CP004065.1_25	K07052	31.30	62.6	6.6e-18	CAAX protease family protein
CP004065.1_26	K06888	62.67	997.1	3.3e-300	uncharacterized protein
CP004065.1_27	K03297	108.13	140.7	1.4e-41	small multidrug resistance pump
CP004065.1_30	K01118	93.70	184.2	8.2e-55	FMN-dependent NADH-azoreductase [EC:1.7.1.17]
CP004065.1_32	K18907	484.60	859.9	9.3e-260	GntR family transcriptional regulator, regulator for abcA and norABC
CP004065.1_33	K00019	183.03	209.4	2e-62	3-hydroxybutyrate dehydrogenase [EC:1.1.1.30]
CP004065.1_35	K01607	17.10	144.6	1.1e-42	4-carboxymuconolactone decarboxylase [EC:4.1.1.44]
CP004065.1_42	K15976	141.90	151.5	4.1e-45	putative NAD(P)H nitroreductase [EC:1.-.-.-]
CP004065.1_43	K08987	33.40	139.6	2.5e-41	putative membrane protein
CP004065.1_47	K01212	646.57	748.4	3.2e-225	levanase [EC:3.2.1.65]
CP004065.1_47	K01193	187.77	315.5	1.6e-94	beta-fructofuranosidase [EC:3.2.1.26]
CP004065.1_48	K00692	298.30	561.4	5e-169	levansucrase [EC:2.4.1.10]
CP004065.1_50	K15986	162.20	448.0	3.5e-135	manganese-dependent inorganic pyrophosphatase [EC:3.6.1.1]
CP004065.1_52	K06329	113.17	221.5	9.7e-67	spore coat protein F
CP004065.1_55	K22927	277.33	882.3	4.4e-266	cyclic-di-AMP phosphodiesterase [EC:3.1.4.59]
CP004065.1_56	K02939	60.20	136.2	3.6e-40	large subunit ribosomal protein L9
CP004065.1_57	K07176	96.37	109.9	2.8e-32	putative serine/threonine protein kinase
CP004065.1_60	K17810	158.87	300.4	5.8e-90	D-aspartate ligase [EC:6.3.1.12]
CP004065.1_66	K02314	110.70	679.0	1.5e-204	replicative DNA helicase [EC:5.6.2.3]
CP004065.1_68	K01939	131.03	649.2	1.3e-195	adenylosuccinate synthase [EC:6.3.4.4]
CP004065.1_69	K07668	340.87	414.8	2.6e-125	two-component system, OmpR family, response regulator VicR
CP004065.1_69	K07658	338.17	340.4	1.9e-102	two-component system, OmpR family, alkaline phosphatase synthesis response regulator PhoP
CP004065.1_69	K02483	242.00	270.2	8.3e-81	two-component system, OmpR family, response regulator
CP004065.1_70	K07652	474.87	642.6	2.3e-193	two-component system, OmpR family, sensor histidine kinase VicK [EC:2.7.13.3]
CP004065.1_70	K07636	310.93	341.4	1.9e-102	two-component system, OmpR family, phosphate regulon sensor histidine kinase PhoR [EC:2.7.13.3]
CP004065.1_76	K02484	312.80	315.7	1.7e-94	two-component system, OmpR family, sensor kinase [EC:2.7.13.3]
CP004065.1_82	K06714	491.73	712.4	7.4e-215	arginine utilization regulatory protein
CP004065.1_83	K26993	108.47	171.4	3e-51	ArsR family transcriptional regulator, repressor of sdpIR and other operons
CP004065.1_83	K03892	89.73	97.9	1.6e-28	ArsR family transcriptional regulator, arsenate/arsenite/antimonite-responsive transcriptional repressor
CP004065.1_84	K26996	27.07	144.6	8.2e-43	immunity protein, SdpI family
CP004065.1_85	K00819	481.33	680.4	4e-205	ornithine--oxo-acid transaminase [EC:2.6.1.13]
CP004065.1_86	K02205	722.83	784.0	1.1e-236	arginine/ornithine permease
CP004065.1_87	K01476	187.27	375.3	1.3e-112	arginase [EC:3.5.3.1]
CP004065.1_91	K05275	257.70	317.4	3.2e-95	pyridoxine 4-dehydrogenase [EC:1.1.1.65]
CP004065.1_96	K00783	35.63	220.6	6.1e-66	23S rRNA (pseudouridine1915-N3)-methyltransferase [EC:2.1.1.177]
CP004065.1_99	K07497	13.93	128.2	1e-37	putative transposase
CP004065.1_100	K07483	40.77	45.3	2.1e-12	transposase

CP004065.1_102	K09384	127.43	515.5	4.5e-155	uncharacterized protein
CP004065.1_106	K07402	68.07	291.2	3e-87	xanthine dehydrogenase accessory factor
CP004065.1_107	K07141	98.60	161.2	7.8e-48	molybdenum cofactor cytidylyltransferase [EC:2.7.7.76]
CP004065.1_111	K04041	529.37	1007.8	4.1e-304	fructose-1,6-bisphosphatase III [EC:3.1.3.11]
CP004065.1_112	K11737	666.23	729.0	1.1e-219	D-serine/D-alanine/glycine transporter
CP004065.1_113	K03387	352.27	846.4	1.7e-255	NADH-dependent peroxiredoxin subunit F [EC:1.8.1.-]
CP004065.1_114	K24119	236.70	345.8	3.7e-104	NADH-dependent peroxiredoxin subunit C [EC:1.11.1.26]
CP004065.1_123	K10255	236.43	424.0	1.2e-127	acyl-lipid omega-6 desaturase (Delta-12 desaturase) [EC:1.14.19.23 1.14.19.45]
CP004065.1_124	K07778	170.90	292.4	1.4e-87	two-component system, NarL family, sensor histidine kinase DesK [EC:2.7.13.3]
CP004065.1_125	K07693	228.97	287.5	2.8e-86	two-component system, NarL family, response regulator DesR
CP004065.1_126	K18009	303.23	313.1	3.9e-94	meso-butanediol dehydrogenase / (S,S)-butanediol dehydrogenase / diacetyl reductase [EC:1.1.1.- 1.1.1.76 1.1.1.304]
CP004065.1_126	K00059	269.80	283.9	4.5e-85	3-oxoacyl-[acyl-carrier protein] reductase [EC:1.1.1.100]
CP004065.1_128	K04079	91.80	723.8	8.2e-218	molecular chaperone HtpG
CP004065.1_129	K06609	469.27	506.7	1.1e-152	MFS transporter, SP family, major inositol transporter
CP004065.1_130	K06607	318.77	492.0	1.2e-148	myo-inositol catabolism protein IolS [EC:1.1.1.-]
CP004065.1_131	K06608	250.50	358.4	4.7e-108	DeoR family transcriptional regulator, myo-inositol catabolism operon repressor
CP004065.1_132	K00140	503.87	771.2	3.1e-232	malonate-semialdehyde dehydrogenase (acetylating) / methylmalonate-semialdehyde dehydrogenase [EC:1.2.1.18 1.2.1.27]
CP004065.1_133	K03337	142.03	328.4	9.8e-99	5-deoxy-glucuronate isomerase [EC:5.3.1.30]
CP004065.1_134	K03338	276.17	362.8	4.6e-109	5-dehydro-2-deoxygluconokinase [EC:2.7.1.92]
CP004065.1_135	K03336	427.33	903.9	1.1e-272	3D-(3,5/4)-trihydroxycyclohexane-1,2-dione acylhydrolase (decyclizing) [EC:3.7.1.22]
CP004065.1_136	K03335	136.63	377.8	1.4e-113	inosose dehydratase [EC:4.2.1.44]
CP004065.1_137	K06610	271.17	533.7	5.2e-161	MFS transporter, SP family, inositol transporter
CP004065.1_138	K00010	272.37	354.9	1.3e-106	myo-inositol 2-dehydrogenase / D-chiro-inositol 1-dehydrogenase [EC:1.1.1.18 1.1.1.369]
CP004065.1_139	K06605	279.07	453.3	5.4e-137	myo-inositol catabolism protein IolH
CP004065.1_140	K06606	172.93	325.6	4.8e-98	2-keto-myo-inositol isomerase [EC:5.3.99.11]
CP004065.1_141	K03339	466.97	490.2	3.7e-148	6-phospho-5-dehydro-2-deoxy-D-gluconate aldolase [EC:4.1.2.29]
CP004065.1_141	K01624	67.57	334.0	5.4e-100	fructose-bisphosphate aldolase, class II [EC:4.1.2.13]
CP004065.1_142	K11634	298.93	367.0	8.2e-111	two-component system, OmpR family, response regulator YxdJ
CP004065.1_143	K11633	303.53	418.2	5.4e-126	two-component system, OmpR family, sensor histidine kinase YxdK [EC:2.7.13.3]
CP004065.1_144	K11635	402.27	437.6	2.4e-132	putative ABC transport system ATP-binding protein
CP004065.1_144	K02003	292.97	313.0	6.7e-94	putative ABC transport system ATP-binding protein
CP004065.1_145	K11636	601.77	837.4	1.5e-252	putative ABC transport system permease protein
CP004065.1_147	K25308	264.57	366.5	2.7e-110	ferric hydroxamate/heme transport system substrate-binding protein
CP004065.1_153	K21064	166.97	204.6	4.6e-61	5-amino-6-(5-phospho-D-ribitylamino)uracil phosphatase [EC:3.1.3.104]
CP004065.1_154	K05346	305.00	420.8	1.3e-126	deoxyribonucleoside regulator
CP004065.1_155	K01619	39.37	336.9	3.8e-101	deoxyribose-phosphate aldolase [EC:4.1.2.4]
CP004065.1_156	K11535	487.87	618.4	1e-186	nucleoside transport protein
CP004065.1_157	K00756	568.60	718.2	9.6e-217	pyrimidine-nucleoside phosphorylase [EC:2.4.2.2]
CP004065.1_158	K16236	853.50	939.5	7.5e-284	histidine permease
CP004065.1_158	K16235	689.77	715.8	4.5e-216	S-methylmethionine transporter
CP004065.1_159	K01479	205.03	264.4	3.5e-79	formiminoglutamase [EC:3.5.3.8]
CP004065.1_160	K01468	322.67	559.4	3.5e-168	imidazolonepropionase [EC:3.5.2.7]
CP004065.1_161	K01712	290.83	818.2	1.4e-246	urocanate hydratase [EC:4.2.1.49]
CP004065.1_162	K01745	410.10	731.2	1.9e-220	histidine ammonia-lyase [EC:4.3.1.3]
CP004065.1_163	K09683	175.93	228.0	8.2e-69	hut operon positive regulatory protein
CP004065.1_164	K06113	228.80	404.0	2.4e-121	arabinan endo-1,5-alpha-L-arabinosidase [EC:3.2.1.99]
CP004065.1_165	K03574	70.57	80.9	1.9e-23	8-oxo-dGTP diphosphatase [EC:3.6.1.55]
CP004065.1_169	K01732	213.53	236.5	1.5e-70	pectin lyase [EC:4.2.2.10]
CP004065.1_184	K05592	531.43	545.9	2.8e-164	ATP-dependent RNA helicase DeaD [EC:5.6.2.7]
CP004065.1_185	K06902	142.93	458.1	6.8e-138	MFS transporter, UMF1 family
CP004065.1_188	K01216	265.47	420.5	1.8e-126	licheninase [EC:3.2.1.73]
CP004065.1_189	K03300	129.87	685.2	8.7e-207	citrate-Mg2+:H+ or citrate-Ca2+:H+ symporter, CitMHS family
CP004065.1_190	K03781	68.70	804.8	1.7e-242	catalase [EC:1.11.1.6]
CP004065.1_193	K16323	510.63	620.6	2.6e-187	purine nucleoside transport protein
CP004065.1_194	K00563	111.90	264.9	1.8e-79	23S rRNA (guanine745-N1)-methyltransferase [EC:2.1.1.187]
CP004065.1_195	K00549	27.93	129.9	2.4e-38	5-methyltetrahydropteroyltriglutamate--homocysteine methyltransferase [EC:2.1.1.14]

CP004065.1_199	K01258	167.60	524.0	9.1e-158	tripeptide aminopeptidase [EC:3.4.11.4]
CP004065.1_200	K01784	253.97	514.0	1.1e-154	UDP-glucose 4-epimerase [EC:5.1.3.2]
CP004065.1_204	K10112	546.20	603.6	8.6e-182	multiple sugar transport system ATP-binding protein [EC:7.5.2.-]
CP004065.1_204	K05816	538.27	543.1	1.1e-163	sn-glycerol 3-phosphate transport system ATP-binding protein [EC:7.6.2.10]
CP004065.1_205	K22278	73.27	94.0	2.2e-27	peptidoglycan-N-acetylglucosamine deacetylase [EC:3.5.1.104]
CP004065.1_206	K11616	246.63	544.7	2.5e-164	malate:Na+ symporter
CP004065.1_207	K00425	142.17	619.8	1.4e-186	cytochrome bd ubiquinol oxidase subunit I [EC:7.1.1.7]
CP004065.1_208	K00426	28.43	396.2	4.3e-119	cytochrome bd ubiquinol oxidase subunit II [EC:7.1.1.7]
CP004065.1_209	K16013	477.03	679.2	1.7e-204	ATP-binding cassette, subfamily C, bacterial CydD
CP004065.1_210	K16012	468.53	604.7	4.2e-182	ATP-binding cassette, subfamily C, bacterial CydC
CP004065.1_211	K17758	277.03	288.2	2.1e-86	ADP-dependent NAD(P)H-hydrate dehydratase [EC:4.2.1.136]
CP004065.1_212	K03457	180.17	271.4	2.9e-81	nucleobase:cation symporter-1, NCS1 family
CP004065.1_213	K24041	365.37	491.6	2.5e-148	penicillin V amidase [EC:3.5.1.11]
CP004065.1_213	K01442	349.37	392.4	7.2e-118	choloylglycine hydrolase [EC:3.5.1.24]
CP004065.1_214	K03781	68.70	734.0	4.5e-221	catalase [EC:1.11.1.6]
CP004065.1_215	K01218	70.20	225.1	4.6e-67	mannan endo-1,4-beta-mannosidase [EC:3.2.1.78]
CP004065.1_216	K01809	33.17	204.3	9.3e-61	mannose-6-phosphate isomerase [EC:5.3.1.8]
CP004065.1_218	K01223	479.40	540.4	8.9e-163	6-phospho-beta-glucosidase [EC:3.2.1.86]
CP004065.1_219	K02761	189.70	521.3	5.8e-157	cellobiose PTS system EIIC component
CP004065.1_220	K02759	33.93	131.8	4.2e-39	cellobiose PTS system EIIA component [EC:2.7.1.196 2.7.1.205]
CP004065.1_221	K02760	71.37	133.6	1.4e-39	cellobiose PTS system EIIB component [EC:2.7.1.196 2.7.1.205]
CP004065.1_222	K03893	199.80	555.8	2e-167	arsenical pump membrane protein
CP004065.1_223	K03892	89.73	106.3	4.3e-31	ArsR family transcriptional regulator, arsenate/arsenite/antimonite-responsive transcriptional repressor
CP004065.1_225	K25289	386.53	426.1	2e-128	ferric hydroxamate/heme transport system permease protein
CP004065.1_226	K25288	404.60	436.0	1.8e-131	ferric hydroxamate/heme transport system permease protein
CP004065.1_227	K03652	52.00	229.4	1.1e-68	DNA-3-methyladenine glycosylase [EC:3.2.2.21]
CP004065.1_228	K03491	482.23	708.3	2.2e-213	probable licABCH operon transcriptional regulator
CP004065.1_229	K02760	71.37	142.6	2.5e-42	cellobiose PTS system EIIB component [EC:2.7.1.196 2.7.1.205]
CP004065.1_230	K02761	189.70	538.6	3.4e-162	cellobiose PTS system EIIC component
CP004065.1_231	K02759	33.93	149.0	2.3e-44	cellobiose PTS system EIIA component [EC:2.7.1.196 2.7.1.205]
CP004065.1_232	K01222	394.63	610.0	6e-184	6-phospho-beta-glucosidase [EC:3.2.1.86]
CP004065.1_233	K00826	167.23	457.4	1.1e-137	branched-chain amino acid aminotransferase [EC:2.6.1.42]
CP004065.1_234	K03740	166.73	445.2	6.2e-134	D-alanine transfer protein
CP004065.1_235	K14188	60.20	114.3	4.3e-34	D-alanine--poly(phosphoribitol) ligase subunit 2 [EC:6.1.1.13]
CP004065.1_236	K03739	275.03	510.4	7.1e-154	membrane protein involved in D-alanine export
CP004065.1_237	K03367	499.93	665.6	9.7e-201	D-alanine--poly(phosphoribitol) ligase subunit 1 [EC:6.1.1.13]
CP004065.1_239	K02548	210.70	297.9	1.9e-89	1,4-dihydroxy-2-naphthoate polyprenyltransferase [EC:2.5.1.74]
CP004065.1_240	K07816	97.83	310.7	3.1e-93	GTP pyrophosphokinase [EC:2.7.6.5]
CP004065.1_242	K13277	440.37	565.8	2.5e-170	minor extracellular protease Epr [EC:3.4.21.-]
CP004065.1_243	K02761	189.70	524.4	6.9e-158	cellobiose PTS system EIIC component
CP004065.1_244	K01759	77.63	90.4	2.7e-26	lactoylglutathione lyase [EC:4.4.1.5]
CP004065.1_245	K06969	218.10	357.6	3.7e-107	23S rRNA (cytosine1962-C5)-methyltransferase [EC:2.1.1.191]
CP004065.1_246	K21744	131.27	202.0	3.9e-60	MerR family transcriptional regulator, thiopeptide resistance regulator
CP004065.1_249	K06518	37.43	136.3	2.1e-40	holin-like protein
CP004065.1_251	K00878	112.23	345.8	7.3e-104	hydroxyethylthiazole kinase [EC:2.7.1.50]
CP004065.1_252	K00788	164.63	254.4	3.5e-76	thiamine-phosphate pyrophosphorylase [EC:2.5.1.3]
CP004065.1_253	K16301	470.73	520.1	1e-156	deferrochelatase/peroxidase EfeB [EC:1.11.1.-]
CP004065.1_257	K19432	66.80	73.3	1.8e-21	anti-repressor of SlrR
CP004065.1_262	K02826	378.57	457.7	5.2e-138	cytochrome aa3-600 menaquinol oxidase subunit II [EC:7.1.1.5]
CP004065.1_263	K02827	976.50	1169.9	0	cytochrome aa3-600 menaquinol oxidase subunit I [EC:7.1.1.5]
CP004065.1_263	K02274	750.43	768.4	1.9e-231	cytochrome c oxidase subunit I [EC:7.1.1.9]
CP004065.1_264	K02828	277.00	310.1	1.4e-93	cytochrome aa3-600 menaquinol oxidase subunit III [EC:7.1.1.5]
CP004065.1_265	K02829	99.33	156.1	5.5e-47	cytochrome aa3-600 menaquinol oxidase subunit IV [EC:7.1.1.5]
CP004065.1_267	K03829	91.77	282.3	8.5e-85	putative acetyltransferase [EC:2.3.1.-]
CP004065.1_269	K19285	297.90	345.8	6.2e-104	FMN reductase (NADPH) [EC:1.5.1.38]
CP004065.1_271	K14647	588.50	828.6	1.7e-249	minor extracellular serine protease Vpr [EC:3.4.21.-]

CP004065.1_273	K26932	374.90	437.1	5.1e-132	sacPA operon transcriptional antiterminator
CP004065.1_273	K03488	318.67	319.5	5.5e-96	beta-glucoside operon transcriptional antiterminator
CP004065.1_274	K02810	596.80	637.7	6.4e-192	sucrose PTS system EIIBCA or EIIBC component [EC:2.7.1.211]
CP004065.1_275	K01193	187.77	440.1	2.9e-132	beta-fructofuranosidase [EC:3.2.1.26]
CP004065.1_278	K10947	65.70	125.5	7.1e-37	PadR family transcriptional regulator
CP004065.1_282	K03648	63.37	385.3	9e-116	uracil-DNA glycosylase [EC:3.2.2.27]
CP004065.1_286	K06305	74.63	219.7	6.2e-66	spore germination protein Q
CP004065.1_289	K13010	413.10	442.7	4.6e-133	perosamine synthetase [EC:2.6.1.102]
CP004065.1_290	K16704	89.27	172.9	2.2e-51	dTDP-4-amino-4,6-dideoxy-D-galactose acyltransferase [EC:2.3.1.210]
CP004065.1_292	K07257	112.70	261.5	2e-78	spore coat polysaccharide biosynthesis protein SpsF
CP004065.1_293	K15897	137.87	202.7	1.9e-60	UDP-2,4-diacetamido-2,4,6-trideoxy-beta-L-altropyranose hydrolase [EC:3.6.1.57]
CP004065.1_294	K00973	234.80	295.3	2.1e-88	glucose-1-phosphate thymidylyltransferase [EC:2.7.7.24]
CP004065.1_295	K01710	493.40	501.2	7.5e-151	dTDP-glucose 4,6-dehydratase [EC:4.2.1.46]
CP004065.1_296	K00067	299.30	364.5	2.2e-109	dTDP-4-dehydrorhamnose reductase [EC:1.1.1.133]
CP004065.1_299	K00260	633.70	705.0	5.9e-213	glutamate dehydrogenase [EC:1.4.1.2]
CP004065.1_300	K00294	478.63	518.7	4.2e-156	1-pyrroline-5-carboxylate dehydrogenase [EC:1.2.1.88]
CP004065.1_302	K02205	722.83	765.0	6.4e-231	arginine/ornithine permease
CP004065.1_303	K07552	308.70	475.9	3.4e-143	MFS transporter, DHA1 family, multidrug resistance protein
CP004065.1_305	K19546	187.90	312.6	1.6e-94	prephenate decarboxylase [EC:4.1.1.100]
CP004065.1_306	K19547	196.40	353.2	7.9e-107	3-[(4R)-4-hydroxycyclohexa-1,5-dien-1-yl]-2-oxopropanoate isomerase [EC:5.3.3.19]
CP004065.1_307	K19548	333.20	414.8	2.4e-125	dihydroanticapsin dehydrogenase [EC:1.1.1.385]
CP004065.1_307	K00059	269.80	277.4	4.2e-83	3-oxoacyl-[acyl-carrier protein] reductase [EC:1.1.1.100]
CP004065.1_308	K13037	475.30	906.0	9.2e-274	L-alanine-L-anticapsin ligase [EC:6.3.2.49]
CP004065.1_309	K19552	745.53	843.0	7e-255	MFS transporter, DHA3 family, bacilysin exporter BacE
CP004065.1_310	K19549	688.50	828.6	1.6e-250	bacilysin biosynthesis transaminase BacF [EC:2.6.1.-]
CP004065.1_311	K19550	388.33	465.1	9.7e-141	bacilysin biosynthesis oxidoreductase BacG [EC:1.3.1.-]
CP004065.1_312	K00435	268.83	310.5	1.7e-93	hydrogen peroxide-dependent heme synthase [EC:1.3.98.5]
CP004065.1_313	K00625	386.90	440.2	1.7e-132	phosphate acetyltransferase [EC:2.3.1.8]
CP004065.1_315	K21900	290.47	376.0	4.5e-113	LysR family transcriptional regulator, transcriptional activator of the cysJI operon
CP004065.1_316	K16869	116.30	328.0	1.3e-98	octanoyl-[GcvH]:protein N-octanoyltransferase [EC:2.3.1.204]
CP004065.1_319	K10947	65.70	118.1	1.3e-34	PadR family transcriptional regulator
CP004065.1_320	K06314	87.53	300.1	2e-90	prespore-specific regulator
CP004065.1_322	K06885	290.33	464.9	9e-140	uncharacterized protein
CP004065.1_323	K09388	56.27	211.4	1.9e-63	uncharacterized protein
CP004065.1_324	K03294	476.00	566.5	1.4e-170	basic amino acid/polyamine antiporter, APA family
CP004065.1_326	K01821	25.90	73.0	6.2e-21	4-oxalocrotonate tautomerase [EC:5.3.2.6]
CP004065.1_330	K21464	776.57	923.3	2e-278	penicillin-binding protein 2D [EC:2.4.99.28 3.4.16.4]
CP004065.1_330	K05366	639.07	665.7	2.6e-200	penicillin-binding protein 1A [EC:2.4.99.28 3.4.16.4]
CP004065.1_331	K00797	93.83	296.9	5.6e-89	spermidine synthase [EC:2.5.1.16]
CP004065.1_332	K01480	221.23	327.5	3e-98	agmatinase [EC:3.5.3.11]
CP004065.1_335	K06384	34.00	95.9	6.4e-28	stage II sporulation protein M
CP004065.1_338	K07729	60.67	91.5	1e-26	putative transcriptional regulator
CP004065.1_339	K06355	77.43	94.9	3.8e-28	phosphatase RapF regulator
CP004065.1_340	K06364	650.10	682.2	4.1e-206	response regulator aspartate phosphatase F [EC:3.1.-.-]
CP004065.1_342	K01887	107.30	481.8	9.2e-145	arginyl-tRNA synthetase [EC:6.1.1.19]
CP004065.1_343	K02575	149.60	352.1	1.5e-105	MFS transporter, NNP family, nitrate/nitrite transporter
CP004065.1_347	K00370	817.30	2036.8	0	nitrate reductase / nitrite oxidoreductase, alpha subunit [EC:1.7.5.1 1.7.99.-]
CP004065.1_348	K00371	338.43	957.5	2.1e-288	nitrate reductase / nitrite oxidoreductase, beta subunit [EC:1.7.5.1 1.7.99.-]
CP004065.1_349	K00373	70.20	170.9	9.4e-51	nitrate reductase molybdenum cofactor assembly chaperone NarJ/NarW
CP004065.1_350	K00374	179.63	303.9	3.8e-91	nitrate reductase gamma subunit [EC:1.7.5.1 1.7.99.-]
CP004065.1_352	K06131	262.77	475.4	7.5e-143	cardiolipin synthase A/B [EC:2.7.8.-]
CP004065.1_353	K06147	612.93	661.4	4.4e-199	ATP-binding cassette, subfamily B, bacterial
CP004065.1_355	K13281	143.30	360.3	4.6e-108	UV DNA damage endonuclease [EC:3.-.-.-]
CP004065.1_356	K06131	262.77	377.4	3.4e-113	cardiolipin synthase A/B [EC:2.7.8.-]
CP004065.1_357	K08264	318.53	350.0	3.4e-105	heterodisulfide reductase subunit D [EC:1.8.98.1]
CP004065.1_358	K03048	43.67	155.1	4.2e-46	DNA-directed RNA polymerase subunit delta

CP004065.1_359	K01937	133.63	898.8	8.8e-271	CTP synthase [EC:6.3.4.2]
CP004065.1_361	K02490	154.10	192.0	1.6e-57	two-component system, response regulator, stage 0 sporulation protein F
CP004065.1_362	K01624	67.57	381.8	1.8e-114	fructose-bisphosphate aldolase, class II [EC:4.1.2.13]
CP004065.1_363	K00616	134.00	189.8	2.4e-56	transaldolase [EC:2.2.1.2]
CP004065.1_364	K00790	172.73	599.9	7.5e-181	UDP-N-acetylglucosamine 1-carboxyvinyltransferase [EC:2.5.1.7]
CP004065.1_365	K02446	462.67	514.2	5.8e-155	fructose-1,6-bisphosphatase II [EC:3.1.3.11]
CP004065.1_366	K03628	186.43	702.7	5.9e-212	transcription termination factor Rho
CP004065.1_367	K02909	21.83	118.9	4.3e-35	large subunit ribosomal protein L31
CP004065.1_368	K00857	82.33	193.6	1.4e-57	thymidine kinase [EC:2.7.1.21]
CP004065.1_369	K00029	287.77	616.2	1.5e-185	malate dehydrogenase (oxaloacetate-decarboxylating)(NADP+) [EC:1.1.1.40]
CP004065.1_369	K00027	507.30	551.8	6.7e-166	malate dehydrogenase (oxaloacetate-decarboxylating) [EC:1.1.1.38]
CP004065.1_370	K13936	190.93	201.6	4.3e-60	malonate transporter and related proteins
CP004065.1_371	K11686	101.30	184.7	3e-55	chromosome-anchoring protein RacA
CP004065.1_372	K08234	96.17	145.0	7.4e-43	glyoxylase I family protein
CP004065.1_373	K02835	355.53	522.4	3.4e-157	peptide chain release factor 1
CP004065.1_374	K02493	233.73	282.4	1.7e-84	release factor glutamine methyltransferase [EC:2.1.1.297]
CP004065.1_377	K06387	65.47	278.6	8.9e-84	stage II sporulation protein R
CP004065.1_379	K07566	93.50	441.7	8.5e-133	L-threonylcarbamoyladenylate synthase [EC:2.7.7.87]
CP004065.1_380	K23242	61.23	178.4	3.7e-53	manganese efflux pump family protein
CP004065.1_381	K20201	148.33	196.9	5.1e-59	protein arginine phosphatase [EC:3.9.1.2]
CP004065.1_382	K01808	185.87	251.9	2.1e-75	ribose 5-phosphate isomerase B [EC:5.3.1.6]
CP004065.1_384	K00600	75.20	702.4	1.3e-211	glycine hydroxymethyltransferase [EC:2.1.2.1]
CP004065.1_385	K00761	217.77	343.0	4.1e-103	uracil phosphoribosyltransferase [EC:2.4.2.9]
CP004065.1_386	K02116	21.47	25.2	3.1e-06	ATP synthase protein I
CP004065.1_387	K02108	158.57	218.7	3.2e-65	F-type H+-transporting ATPase subunit a
CP004065.1_388	K02110	68.60	86.5	4.3e-25	F-type H+-transporting ATPase subunit c
CP004065.1_389	K02109	43.13	130.8	1.7e-38	F-type H+-transporting ATPase subunit b
CP004065.1_390	K02113	45.90	148.3	7.2e-44	F-type H+-transporting ATPase subunit delta
CP004065.1_391	K02111	432.10	929.5	3.5e-280	F-type H+/Na+-transporting ATPase subunit alpha [EC:7.1.2.2 7.2.2.1]
CP004065.1_392	K02115	246.97	388.6	5.7e-117	F-type H+-transporting ATPase subunit gamma
CP004065.1_393	K02112	465.83	895.5	4.6e-270	F-type H+/Na+-transporting ATPase subunit beta [EC:7.1.2.2 7.2.2.1]
CP004065.1_394	K02114	99.10	158.3	4.4e-47	F-type H+-transporting ATPase subunit epsilon
CP004065.1_398	K00790	172.73	650.8	2.8e-196	UDP-N-acetylglucosamine 1-carboxyvinyltransferase [EC:2.5.1.7]
CP004065.1_399	K06381	107.00	372.5	7.2e-112	stage II sporulation protein D (peptidoglycan lytic transglycosylase) [EC:4.2.2.29]
CP004065.1_403	K02379	83.90	299.2	1.1e-89	FdhD protein
CP004065.1_404	K03639	198.23	429.1	5.9e-129	GTP 3',8-cyclase [EC:4.1.99.22]
CP004065.1_408	K01430	175.63	190.4	3.8e-57	urease subunit gamma [EC:3.5.1.5]
CP004065.1_409	K01429	171.47	173.5	1.2e-51	urease subunit beta [EC:3.5.1.5]
CP004065.1_410	K01428	617.27	1059.7	1.1e-319	urease subunit alpha [EC:3.5.1.5]
CP004065.1_415	K06131	262.77	534.1	1.3e-160	cardiolipin synthase A/B [EC:2.7.8.-]
CP004065.1_419	K06386	162.03	366.7	2.4e-110	stage II sporulation protein Q
CP004065.1_421	K19302	106.60	132.2	5.8e-39	undecaprenyl-diphosphatase [EC:3.6.1.27]
CP004065.1_422	K03588	356.93	446.4	2.3e-134	cell division protein FtsW
CP004065.1_423	K04751	115.67	127.5	1.6e-37	nitrogen regulatory protein P-II 1
CP004065.1_424	K03320	145.33	503.0	3.3e-151	ammonium transporter, Amt family
CP004065.1_427	K03457	180.17	521.3	8.1e-157	nucleobase:cation symporter-1, NCS1 family
CP004065.1_430	K06283	55.80	159.3	4.5e-48	putative DeoR family transcriptional regulator, stage III sporulation protein D
CP004065.1_431	K03569	108.73	518.6	2.2e-156	rod shape-determining protein MreB and related proteins
CP004065.1_432	K02391	233.33	251.6	2.1e-75	flagellar basal-body rod protein FlgF
CP004065.1_434	K06362	447.23	529.9	7.3e-160	response regulator aspartate phosphatase D [EC:3.1.-.-]
CP004065.1_435	K02372	79.93	232.5	2.3e-69	3-hydroxyacyl-[acyl-carrier-protein] dehydratase [EC:4.2.1.59]
CP004065.1_436	K03282	37.57	185.3	3e-55	large conductance mechanosensitive channel
CP004065.1_439	K03111	28.83	136.5	3.4e-40	single-strand DNA-binding protein
CP004065.1_440	K22103	255.30	364.4	5.4e-110	DeoR family transcriptional regulator, carbon catabolite repression regulator
CP004065.1_441	K21064	166.97	221.9	2.4e-66	5-amino-6-(5-phospho-D-ribitylamino)uracil phosphatase [EC:3.1.3.104]
CP004065.1_445	K19420	201.30	306.5	3.6e-92	protein tyrosine kinase modulator

CP004065.1_446	K00903	250.43	283.5	5.9e-85	protein-tyrosine kinase [EC:2.7.10.3]
CP004065.1_448	K00012	358.77	616.7	8.4e-186	UDPglucose 6-dehydrogenase [EC:1.1.1.22]
CP004065.1_456	K21494	85.80	179.2	1.1e-53	antitoxin YxxD
CP004065.1_459	K05982	231.57	231.8	3.6e-69	deoxyribonuclease V [EC:3.1.21.7]
CP004065.1_463	K07240	36.03	129.1	4.5e-38	chromate transporter
CP004065.1_464	K07240	36.03	143.2	2.2e-42	chromate transporter
CP004065.1_466	K00681	540.00	585.9	2.8e-176	gamma-glutamyltranspeptidase / glutathione hydrolase [EC:2.3.2.2 3.4.19.13]
CP004065.1_470	K06330	124.73	368.7	9.9e-111	spore coat protein H
CP004065.1_471	K06325	71.47	329.9	2.6e-99	spore coat protein B
CP004065.1_473	K03893	199.80	390.4	2.6e-117	arsenical pump membrane protein
CP004065.1_475	K21757	301.17	331.6	1.2e-99	LysR family transcriptional regulator, benzoate and cis,cis-muconate-responsive activator of ben and cat genes
CP004065.1_475	K05817	281.53	294.1	2.4e-88	LysR family transcriptional regulator, hca operon transcriptional activator
CP004065.1_477	K01575	93.43	336.9	1.7e-101	acetolactate decarboxylase [EC:4.1.1.5]
CP004065.1_480	K10439	190.90	319.5	1.2e-95	ribose transport system substrate-binding protein
CP004065.1_481	K10440	333.83	432.6	4.2e-130	ribose transport system permease protein
CP004065.1_481	K02057	301.83	322.0	1.3e-96	simple sugar transport system permease protein
CP004065.1_482	K10441	693.10	725.1	1.8e-218	ribose transport system ATP-binding protein [EC:7.5.2.7]
CP004065.1_483	K06726	111.10	201.2	2.2e-60	D-ribose pyranase [EC:5.4.99.62]
CP004065.1_484	K00852	237.13	379.5	7.1e-114	ribokinase [EC:2.7.1.15]
CP004065.1_485	K02529	268.37	316.3	9.4e-95	LacI family transcriptional regulator, galactose operon repressor
CP004065.1_488	K06295	452.20	662.9	1.6e-199	spore germination protein KA
CP004065.1_489	K01932	138.93	509.4	2.7e-153	gamma-polyglutamate synthase [EC:6.3.2.-]
CP004065.1_490	K22116	52.40	142.4	3.7e-42	gamma-polyglutamate biosynthesis protein CapC
CP004065.1_491	K07282	220.47	570.6	3e-172	gamma-polyglutamate biosynthesis protein CapA
CP004065.1_493	K21471	158.57	330.7	5.5e-99	peptidoglycan DL-endopeptidase CwlO [EC:3.4.-.-]
CP004065.1_493	K19303	194.30	325.8	8.9e-98	murein DD-endopeptidase [EC:3.4.-.-]
CP004065.1_495	K21064	166.97	220.8	5.5e-66	5-amino-6-(5-phospho-D-ribitylamino)uracil phosphatase [EC:3.1.3.104]
CP004065.1_496	K01005	193.97	290.3	7.1e-87	polyisoprenyl-teichoic acid--peptidoglycan teichoic acid transferase [EC:2.7.8.-]
CP004065.1_497	K06609	469.27	479.4	2.2e-144	MFS transporter, SP family, major inositol transporter
CP004065.1_498	K16870	147.83	171.9	5.6e-51	N-acetylglucosaminyl-diphospho-decaprenol L-rhamnosyltransferase [EC:2.4.1.289]
CP004065.1_499	K06293	379.83	514.2	4.4e-155	spore germination protein BC
CP004065.1_500	K06292	400.60	557.0	3.9e-168	spore germination protein BB
CP004065.1_501	K06291	806.93	850.4	5.9e-257	spore germination protein BA
CP004065.1_501	K06295	452.20	620.8	8.6e-187	spore germination protein KA
CP004065.1_502	K01809	33.17	177.2	1.5e-52	mannose-6-phosphate isomerase [EC:5.3.1.8]
CP004065.1_503	K23989	349.43	533.4	2.6e-160	mannosyl-glycoprotein endo-beta-N-acetylglucosaminidase [EC:3.2.1.96]
CP004065.1_504	K21285	284.57	394.5	1.5e-118	teichoic acid glycerol-phosphate primase [EC:2.7.8.44]
CP004065.1_505	K05946	235.07	280.3	6.8e-84	N-acetylglucosaminyldiphosphoundecaprenol N-acetyl-beta-D-mannosaminyltransferase [EC:2.4.1.187]
CP004065.1_506	K00980	122.07	214.3	2.3e-64	glycerol-3-phosphate cytidylyltransferase [EC:2.7.7.39]
CP004065.1_508	K09809	276.63	531.7	1.1e-159	CDP-glycerol glycerophosphotransferase [EC:2.7.8.12]
CP004065.1_509	K09692	218.47	301.0	2.1e-90	teichoic acid transport system permease protein
CP004065.1_510	K09693	367.70	432.6	3e-130	teichoic acid transport system ATP-binding protein [EC:7.5.2.4]
CP004065.1_511	K00963	76.77	159.4	3.3e-47	UTP--glucose-1-phosphate uridylyltransferase [EC:2.7.7.9]
CP004065.1_512	K01791	432.27	586.5	1.5e-176	UDP-N-acetylglucosamine 2-epimerase (non-hydrolysing) [EC:5.1.3.14]
CP004065.1_513	K01005	193.97	308.9	1.6e-92	polyisoprenyl-teichoic acid--peptidoglycan teichoic acid transferase [EC:2.7.8.-]
CP004065.1_515	K06381	107.00	268.3	3e-80	stage II sporulation protein D (peptidoglycan lytic transglycosylase) [EC:4.2.2.29]
CP004065.1_516	K01448	29.13	228.6	2.2e-68	N-acetylmuramoyl-L-alanine amidase [EC:3.5.1.28]
CP004065.1_518	K16694	612.33	815.5	3e-246	teichuronic acid exporter
CP004065.1_519	K16697	576.73	691.2	7.5e-209	teichuronic acid biosynthesis glycosyltransferase TuaC [EC:2.4.-.-]
CP004065.1_520	K00012	358.77	635.1	2.2e-191	UDPglucose 6-dehydrogenase [EC:1.1.1.22]
CP004065.1_521	K16705	369.13	748.2	7.7e-226	teichuronic acid biosynthesis protein TuaE
CP004065.1_522	K16706	162.43	341.4	5.6e-103	teichuronic acid biosynthesis protein TuaF
CP004065.1_523	K16698	353.27	453.0	4.9e-137	teichuronic acid biosynthesis glycosyltransferase TuaG [EC:2.4.-.-]
CP004065.1_524	K16699	408.97	661.0	9.4e-200	teichuronic acid biosynthesis glycosyltransferase TuaH [EC:2.4.-.-]
CP004065.1_525	K02851	237.03	403.1	3.3e-121	UDP-GlcNAc:undecaprenyl-phosphate/decaprenyl-phosphate GlcNAc-1-phosphate transferase [EC:2.7.8.33 2.7.8.35]
CP004065.1_526	K01005	193.97	324.7	2.6e-97	polyisoprenyl-teichoic acid--peptidoglycan teichoic acid transferase [EC:2.7.8.-]

CP004065.1_528	K07777	248.37	451.1	1.1e-135	two-component system, NarL family, sensor histidine kinase DegS [EC:2.7.13.3]
CP004065.1_529	K07692	255.17	350.0	1.5e-105	two-component system, NarL family, response regulator DegU
CP004065.1_530	K25232	87.57	328.1	1.7e-98	fatty acid kinase fatty acid binding subunit
CP004065.1_531	K02240	272.23	607.4	7.5e-183	competence protein ComFA
CP004065.1_532	K02241	47.70	72.3	7.3e-21	competence protein ComFB
CP004065.1_533	K02242	65.47	141.6	8.2e-42	competence protein ComFC
CP004065.1_535	K02398	24.90	64.9	2.4e-18	negative regulator of flagellin synthesis FlgM
CP004065.1_536	K02399	40.07	52.5	1.2e-14	flagellar biosynthesis protein FlgN
CP004065.1_537	K02396	100.10	499.0	7.6e-150	flagellar hook-associated protein 1
CP004065.1_538	K02397	144.10	257.4	4.8e-77	flagellar hook-associated protein 3 FlgL
CP004065.1_540	K13626	52.33	171.5	5e-51	flagellar assembly factor FliW
CP004065.1_541	K03563	23.50	98.2	9.4e-29	carbon storage regulator
CP004065.1_542	K02406	132.37	429.9	1.5e-129	flagellin
CP004065.1_543	K02407	69.27	370.9	3.2e-111	flagellar hook-associated protein 2
CP004065.1_544	K02422	28.13	151.7	6.2e-45	flagellar secretion chaperone FliS
CP004065.1_545	K02423	26.07	41.1	4.6e-11	flagellar protein FliT
CP004065.1_547	K05808	97.77	232.8	6.4e-70	ribosome hibernation promoting factor
CP004065.1_548	K03070	231.23	1249.6	0	preprotein translocase subunit SecA [EC:7.4.2.8]
CP004065.1_549	K02836	378.07	562.9	1.9e-169	peptide chain release factor 2
CP004065.1_551	K12263	109.73	130.4	1.2e-38	cytochrome c551
CP004065.1_552	K09812	287.03	373.2	2.3e-112	cell division transport system ATP-binding protein
CP004065.1_553	K09811	79.70	272.1	1.1e-81	cell division transport system permease protein
CP004065.1_555	K03797	127.93	400.8	2.6e-120	carboxyl-terminal processing protease [EC:3.4.21.102]
CP004065.1_561	K07493	56.73	121.1	1.4e-35	putative transposase
CP004065.1_565	K22579	503.63	564.7	1.1e-169	rifampicin phosphotransferase [EC:2.7.9.6]
CP004065.1_567	K03702	343.33	1165.6	0	excinuclease ABC subunit B
CP004065.1_568	K03701	341.57	1487.3	0	excinuclease ABC subunit A
CP004065.1_571	K11621	70.10	99.7	3.4e-29	lia operon protein LiaG
CP004065.1_573	K08972	34.93	116.4	4.4e-34	putative membrane protein
CP004065.1_575	K18939	163.23	267.7	1.2e-80	TetR/AcrR family transcriptional regulator, lmrAB and yxaGH operons repressor
CP004065.1_575	K16137	104.80	106.7	4e-31	TetR/AcrR family transcriptional regulator, transcriptional repressor for nem operon
CP004065.1_576	K24967	246.20	293.0	8.8e-88	GntR family transcriptional regulator, N-acetylglucosamine utilization regulator
CP004065.1_576	K03710	178.10	229.3	2e-68	GntR family transcriptional regulator
CP004065.1_577	K02564	97.07	346.3	4.5e-104	glucosamine-6-phosphate deaminase [EC:3.5.99.6]
CP004065.1_578	K01443	131.87	420.8	2.2e-126	N-acetylglucosamine-6-phosphate deacetylase [EC:3.5.1.25]
CP004065.1_579	K06023	97.50	459.5	3.1e-138	HPr kinase/phosphorylase [EC:2.7.11.- 2.7.4.-]
CP004065.1_580	K13292	50.50	295.6	9.7e-89	phosphatidylglycerol---prolipoprotein diacylglyceryl transferase [EC:2.5.1.145]
CP004065.1_581	K06019	203.97	330.4	1.4e-99	pyrophosphatase PpaX [EC:3.6.1.1]
CP004065.1_581	K01091	112.83	175.5	4.4e-52	phosphoglycolate phosphatase [EC:3.1.3.18]
CP004065.1_582	K24872	135.27	300.0	7.3e-91	heptaprenylglycerol acetyltransferase [EC:2.3.1.-]
CP004065.1_584	K02502	242.30	377.6	1.9e-113	ATP phosphoribosyltransferase regulatory subunit
CP004065.1_585	K00765	43.93	232.9	1.3e-69	ATP phosphoribosyltransferase [EC:2.4.2.17]
CP004065.1_586	K00013	519.37	641.4	4.2e-193	histidinol dehydrogenase [EC:1.1.1.23]
CP004065.1_587	K01693	300.33	310.9	1.4e-93	imidazoleglycerol-phosphate dehydratase [EC:4.2.1.19]
CP004065.1_588	K02501	231.60	274.5	1.6e-82	imidazole glycerol-phosphate synthase subunit HisH [EC:4.3.2.10]
CP004065.1_589	K01814	249.67	290.8	4.8e-87	phosphoribosylformimino-5-aminoimidazole carboxamide ribotide isomerase [EC:5.3.1.16]
CP004065.1_590	K02500	339.30	447.3	1.7e-134	imidazole glycerol-phosphate synthase subunit HisF [EC:4.3.2.10]
CP004065.1_591	K11755	228.33	329.5	7e-99	phosphoribosyl-AMP cyclohydrolase / phosphoribosyl-ATP pyrophosphohydrolase [EC:3.5.4.19 3.6.1.31]
CP004065.1_592	K18104	642.17	953.6	1.4e-287	ATP-binding cassette, subfamily B, bacterial AbcA/BmrA [EC:7.6.2.2]
CP004065.1_594	K02483	242.00	269.0	1.9e-80	two-component system, OmpR family, response regulator
CP004065.1_598	K00384	332.10	426.1	3.6e-128	thioredoxin reductase (NADPH) [EC:1.8.1.9]
CP004065.1_600	K06958	140.17	425.6	3.4e-128	RNase adapter protein RapZ
CP004065.1_602	K09762	93.53	414.3	8.9e-125	cell division protein WhiA
CP004065.1_603	K11184	111.63	136.9	3e-41	catabolite repression HPr-like protein
CP004065.1_603	K02784	81.30	107.4	1.8e-31	phosphocarrier protein HPr
CP004065.1_605	K01167	44.03	50.5	5.8e-14	ribonuclease T1 [EC:4.6.1.24]

CP004065.1_606	K00090	433.87	460.7	4.8e-139	glyoxylate/hydroxypyruvate/2-ketogluconate reductase [EC:1.1.1.79 1.1.1.81 1.1.1.215]
CP004065.1_607	K01673	19.40	80.5	1.9e-23	carbonic anhydrase [EC:4.2.1.1]
CP004065.1_608	K03321	345.37	515.7	4.3e-155	sulfate permease, SulP family
CP004065.1_610	K22522	96.03	231.8	3.4e-69	cytokinin riboside 5'-monophosphate phosphoribohydrolase [EC:3.2.2.-]
CP004065.1_612	K08369	358.47	434.4	1e-130	MFS transporter, putative metabolite:H+ symporter
CP004065.1_615	K01358	76.73	364.0	1.5e-109	ATP-dependent Clp protease, protease subunit [EC:3.4.21.92]
CP004065.1_616	K03484	339.57	402.9	3.1e-121	LacI family transcriptional regulator, sucrose operon repressor
CP004065.1_616	K02529	268.37	291.2	3.6e-87	LacI family transcriptional regulator, galactose operon repressor
CP004065.1_617	K02532	177.13	582.4	8.3e-176	MFS transporter, OHS family, lactose permease
CP004065.1_618	K01193	187.77	533.7	1.3e-160	beta-fructofuranosidase [EC:3.2.1.26]
CP004065.1_625	K13727	152.17	285.2	2.6e-86	phenacrylate decarboxylase [EC:4.1.1.102]
CP004065.1_626	K22441	138.10	247.9	2.9e-74	diamine N-acetyltransferase [EC:2.3.1.57]
CP004065.1_627	K07149	76.20	166.1	1.8e-49	uncharacterized protein
CP004065.1_630	K03929	467.47	566.2	2.1e-170	para-nitrobenzyl esterase [EC:3.1.1.-]
CP004065.1_631	K19417	255.67	275.0	3.7e-83	XRE family transcriptional regulator, biofilm formation regulator
CP004065.1_632	K19420	201.30	244.7	2.3e-73	protein tyrosine kinase modulator
CP004065.1_633	K00903	250.43	313.2	6e-94	protein-tyrosine kinase [EC:2.7.10.3]
CP004065.1_634	K19421	1153.33	1262.0	0	polysaccharide biosynthesis protein EpsC
CP004065.1_635	K19422	366.37	580.9	1.9e-175	glycosyltransferase EpsD [EC:2.4.-.-]
CP004065.1_636	K19423	227.77	406.5	9.3e-123	glycosyltransferase EpsE [EC:2.4.-.-]
CP004065.1_637	K19424	269.13	558.2	1.5e-168	glycosyltransferase EpsF [EC:2.4.-.-]
CP004065.1_638	K19419	144.33	462.3	1.7e-139	transmembrane protein EpsG
CP004065.1_639	K19425	407.90	525.1	1.8e-158	glycosyltransferase EpsH [EC:2.4.-.-]
CP004065.1_640	K19426	252.43	571.3	1.4e-172	pyruvyl transferase EpsI [EC:2.-.-.-]
CP004065.1_641	K19427	316.40	470.2	1e-141	glycosyltransferase EpsJ [EC:2.4.-.-]
CP004065.1_642	K19418	371.70	845.8	2.3e-255	membrane protein EpsK
CP004065.1_643	K19428	366.17	388.2	1.1e-117	sugar transferase EpsL [EC:2.-.-.-]
CP004065.1_643	K15915	274.00	280.2	1.4e-84	undecaprenyl phosphate N,N'-diacetylbacillosamine 1-phosphate transferase [EC:2.7.8.36]
CP004065.1_644	K19429	223.10	295.5	3.6e-89	acetyltransferase EpsM [EC:2.3.1.-]
CP004065.1_645	K19430	573.97	666.7	1.8e-201	pyridoxal phosphate-dependent aminotransferase EpsN [EC:2.6.1.-]
CP004065.1_646	K19431	359.93	529.7	4.8e-160	pyruvyl transferase EpsO [EC:2.-.-.-]
CP004065.1_648	K03092	57.77	466.1	3.2e-140	RNA polymerase sigma-54 factor
CP004065.1_649	K03303	102.10	666.5	7.7e-201	lactate permease
CP004065.1_650	K05799	252.00	292.4	5.7e-88	GntR family transcriptional regulator, transcriptional repressor for pyruvate dehydrogenase complex
CP004065.1_651	K05825	385.80	415.7	7.1e-125	2-aminoadipate transaminase [EC:2.6.1.-]
CP004065.1_652	K18928	254.60	382.3	2.9e-115	L-lactate dehydrogenase complex protein LldE
CP004065.1_653	K18929	437.67	711.7	1.7e-214	L-lactate dehydrogenase complex protein LldF
CP004065.1_654	K00782	116.30	154.1	1.2e-45	L-lactate dehydrogenase complex protein LldG
CP004065.1_655	K03299	88.80	555.2	2.8e-167	gluconate:H+ symporter, GntP family
CP004065.1_656	K25031	463.90	746.5	3.4e-225	gluconokinase [EC:2.7.1.12]
CP004065.1_658	K21064	166.97	189.7	1.5e-56	5-amino-6-(5-phospho-D-ribitylamino)uracil phosphatase [EC:3.1.3.104]
CP004065.1_659	K07149	76.20	183.6	8.5e-55	uncharacterized protein
CP004065.1_663	K15269	141.60	146.8	1.5e-43	probable blue pigment (indigoidine) exporter
CP004065.1_667	K02103	274.17	471.8	4.4e-142	GntR family transcriptional regulator, arabinose operon transcriptional repressor
CP004065.1_668	K02100	553.43	630.3	4.6e-190	MFS transporter, SP family, arabinose:H+ symporter
CP004065.1_670	K05311	215.10	489.8	6.5e-148	central glycolytic genes regulator
CP004065.1_671	K00134	414.10	549.9	1.2e-165	glyceraldehyde 3-phosphate dehydrogenase (phosphorylating) [EC:1.2.1.12]
CP004065.1_671	K00150	349.40	351.9	6.9e-106	glyceraldehyde-3-phosphate dehydrogenase (NAD(P)+) (phosphorylating) [EC:1.2.1.59]
CP004065.1_672	K00927	104.93	649.3	9.3e-196	phosphoglycerate kinase [EC:2.7.2.3]
CP004065.1_673	K01803	24.57	338.8	7.9e-102	triosephosphate isomerase (TIM) [EC:5.3.1.1]
CP004065.1_674	K15633	73.30	798.4	8.1e-241	2,3-bisphosphoglycerate-independent phosphoglycerate mutase [EC:5.4.2.12]
CP004065.1_675	K01689	269.20	776.3	8.9e-234	enolase 1/2/3 [EC:4.2.1.11]
CP004065.1_676	K19701	238.97	347.8	3.2e-104	aminopeptidase YwaD [EC:3.4.11.6 3.4.11.10]
CP004065.1_677	K13015	437.70	605.0	2.7e-182	UDP-N-acetyl-D-glucosamine dehydrogenase [EC:1.1.1.136]
CP004065.1_678	K13010	413.10	431.8	9.8e-130	perosamine synthetase [EC:2.6.1.102]
CP004065.1_683	K01463	161.63	189.8	1.5e-56	N-acetylglucosamine malate deacetylase 1 [EC:3.5.1.-]

CP004065.1_687	K21463	189.60	200.7	7.8e-60	membrane-associated protein TcaA
CP004065.1_689	K05595	81.43	188.9	2.8e-56	multiple antibiotic resistance protein
CP004065.1_690	K22301	217.30	267.3	1.7e-80	HTH-type transcriptional regulator, osmoprotectant uptake regulator
CP004065.1_691	K05847	352.27	490.8	7.7e-148	osmoprotectant transport system ATP-binding protein [EC:7.6.2.9]
CP004065.1_692	K05846	171.73	290.2	7.5e-87	osmoprotectant transport system permease protein
CP004065.1_693	K05845	152.73	310.7	3.3e-93	osmoprotectant transport system substrate-binding protein
CP004065.1_694	K05846	171.73	274.5	4.4e-82	osmoprotectant transport system permease protein
CP004065.1_695	K22301	217.30	277.8	1.1e-83	HTH-type transcriptional regulator, osmoprotectant uptake regulator
CP004065.1_696	K05847	352.27	484.3	7.3e-146	osmoprotectant transport system ATP-binding protein [EC:7.6.2.9]
CP004065.1_697	K05846	171.73	281.7	2.9e-84	osmoprotectant transport system permease protein
CP004065.1_698	K05845	152.73	276.5	7.9e-83	osmoprotectant transport system substrate-binding protein
CP004065.1_699	K05846	171.73	271.1	4.7e-81	osmoprotectant transport system permease protein
CP004065.1_700	K20490	293.47	348.4	3.5e-105	lantibiotic transport system ATP-binding protein
CP004065.1_700	K01990	262.37	281.9	2.1e-84	ABC-2 type transport system ATP-binding protein
CP004065.1_701	K20491	99.07	226.1	1e-67	lantibiotic transport system permease protein
CP004065.1_702	K20492	117.57	266.9	3.1e-80	lantibiotic transport system permease protein
CP004065.1_703	K20488	263.17	349.0	1.9e-105	two-component system, OmpR family, lantibiotic biosynthesis response regulator NisR/SpaR
CP004065.1_704	K20487	242.97	439.7	3e-132	two-component system, OmpR family, lantibiotic biosynthesis sensor histidine kinase NisK/SpaK [EC:2.7.13.3]
CP004065.1_706	K22299	76.73	134.1	1e-39	HTH-type transcriptional regulator, competence development regulator
CP004065.1_707	K22299	76.73	134.6	7.3e-40	HTH-type transcriptional regulator, competence development regulator
CP004065.1_709	K03075	26.13	80.6	3.3e-23	preprotein translocase subunit SecG
CP004065.1_710	K03928	134.07	264.9	2.7e-79	carboxylesterase [EC:3.1.1.1]
CP004065.1_711	K12573	484.07	1059.6	1.9e-319	ribonuclease R [EC:3.1.13.1]
CP004065.1_712	K03664	42.43	253.9	2.5e-76	SsrA-binding protein
CP004065.1_714	K03623	37.70	110.2	8.3e-33	ribonuclease inhibitor
CP004065.1_716	K21960	245.43	401.3	4.8e-121	LysR family transcriptional regulator, regulator of the ytmI operon
CP004065.1_717	K00680	233.53	264.8	5.3e-80	uncharacterized N-acetyltransferase [EC:2.3.1.-]
CP004065.1_718	K16956	410.33	470.1	3.7e-142	L-cystine transport system substrate-binding protein
CP004065.1_718	K16957	256.73	320.4	2.5e-96	L-cystine transport system substrate-binding protein
CP004065.1_718	K02030	76.77	123.9	1.9e-36	polar amino acid transport system substrate-binding protein
CP004065.1_719	K16957	256.73	344.7	1e-103	L-cystine transport system substrate-binding protein
CP004065.1_719	K02030	76.77	113.7	2.5e-33	polar amino acid transport system substrate-binding protein
CP004065.1_720	K16958	278.73	311.9	4.6e-94	L-cystine transport system permease protein
CP004065.1_720	K02029	214.53	233.9	7.1e-70	polar amino acid transport system permease protein
CP004065.1_721	K16959	256.10	308.9	3.9e-93	L-cystine transport system permease protein
CP004065.1_722	K02028	386.70	430.0	2.8e-129	polar amino acid transport system ATP-binding protein [EC:7.4.2.1]
CP004065.1_722	K16960	403.47	412.9	7.2e-125	L-cystine transport system ATP-binding protein [EC:7.4.2.1]
CP004065.1_725	K24116	523.57	549.8	9.3e-166	N-acetyl-S-(2-succino)cysteine monooxygenase [EC:1.14.-.-]
CP004065.1_727	K01607	17.10	142.9	3.5e-42	4-carboxymuconolactone decarboxylase [EC:4.1.1.44]
CP004065.1_728	K08224	171.37	172.0	3.3e-51	MFS transporter, YNFM family, putative membrane transport protein
CP004065.1_730	K01118	93.70	209.7	1.4e-62	FMN-dependent NADH-azoreductase [EC:1.7.1.17]
CP004065.1_731	K16044	544.50	651.8	4.7e-197	scyllo-inositol 2-dehydrogenase (NADP+) [EC:1.1.1.371]
CP004065.1_732	K21600	85.87	136.7	2.1e-40	CsoR family transcriptional regulator, copper-sensing transcriptional repressor
CP004065.1_733	K07213	66.63	73.8	4.7e-21	copper chaperone
CP004065.1_734	K17686	799.73	1112.5	0	P-type Cu+ transporter [EC:7.2.2.8]
CP004065.1_734	K01533	923.57	970.4	3.7e-292	P-type Cu2+ transporter [EC:7.2.2.9]
CP004065.1_735	K01534	713.30	876.0	5e-264	Zn2+/Cd2+-exporting ATPase [EC:7.2.2.12 7.2.2.21]
CP004065.1_741	K00380	519.97	737.4	6.2e-222	sulfite reductase (NADPH) flavoprotein alpha-component [EC:1.8.1.2]
CP004065.1_742	K00381	518.57	1006.9	3.3e-303	sulfite reductase (NADPH) hemoprotein beta-component [EC:1.8.1.2]
CP004065.1_743	K24163	379.43	604.9	5.7e-182	monovalent cation/hydrogen antiporter
CP004065.1_745	K23257	397.27	473.1	4.6e-143	methylglyoxal/glyoxal reductase [EC:1.1.1.283 1.1.1.-]
CP004065.1_745	K06221	349.23	429.9	1.1e-129	2,5-diketo-D-gluconate reductase A [EC:1.1.1.346]
CP004065.1_746	K25142	220.57	307.6	2.8e-92	linearmycin/streptolysin S transport system permease protein
CP004065.1_746	K01992	16.23	24.9	1e-06	ABC-2 type transport system permease protein
CP004065.1_747	K25143	233.20	297.9	2.7e-89	linearmycin/streptolysin S transport system permease protein
CP004065.1_747	K01992	16.23	27.4	1.8e-07	ABC-2 type transport system permease protein

CP004065.1_748	K25144	376.07	433.8	1.8e-130	linearmycin/streptolysin S transport system ATP-binding protein
CP004065.1_748	K01990	262.37	311.3	2.6e-93	ABC-2 type transport system ATP-binding protein
CP004065.1_751	K02018	245.63	275.3	2.4e-82	molybdate transport system permease protein
CP004065.1_752	K02020	109.77	297.7	3.7e-89	molybdate transport system substrate-binding protein
CP004065.1_753	K07219	145.43	324.3	3.4e-97	putative molybdopterin biosynthesis protein
CP004065.1_754	K19005	299.10	702.3	2.3e-211	lipoteichoic acid synthase [EC:2.7.8.20]
CP004065.1_755	K07038	27.13	87.8	1.5e-25	inner membrane protein
CP004065.1_756	K06427	87.13	106.7	1e-31	small acid-soluble spore protein J (minor)
CP004065.1_757	K03758	364.87	494.7	6.8e-149	arginine:ornithine antiporter / lysine permease
CP004065.1_759	K25308	264.57	352.6	4.3e-106	ferric hydroxamate/heme transport system substrate-binding protein
CP004065.1_760	K25288	404.60	493.4	6.3e-149	ferric hydroxamate/heme transport system permease protein
CP004065.1_761	K25289	386.53	452.0	2.7e-136	ferric hydroxamate/heme transport system permease protein
CP004065.1_762	K25290	398.40	438.3	1.8e-132	ferric hydroxamate/heme transport system ATP-binding protein [EC:7.2.2.16]
CP004065.1_762	K02013	238.30	336.6	5.4e-101	iron complex transport system ATP-binding protein [EC:7.2.2.-]
CP004065.1_763	K26991	150.93	207.0	2.1e-62	membrane-bound negative regulator
CP004065.1_764	K01569	93.33	618.7	9.8e-187	oxalate decarboxylase [EC:4.1.1.2]
CP004065.1_766	K26992	75.90	224.1	2.9e-67	RNA polymerase sigma-O factor
CP004065.1_767	K26990	52.93	90.4	1.1e-26	sigma-O factor regulatory protein RsoA
CP004065.1_770	K01053	182.60	191.0	8e-57	gluconolactonase [EC:3.1.1.17]
CP004065.1_772	K25034	226.00	308.2	1.6e-92	cobalamin transport system substrate-binding protein
CP004065.1_772	K02016	154.03	235.6	2.4e-70	iron complex transport system substrate-binding protein
CP004065.1_773	K25027	379.47	387.9	1.1e-116	cobalamin transport system permease protein
CP004065.1_774	K25028	368.20	390.6	3.7e-117	cobalamin transport system ATP-binding protein [EC:7.6.2.8]
CP004065.1_774	K02013	238.30	336.0	8.1e-101	iron complex transport system ATP-binding protein [EC:7.2.2.-]
CP004065.1_775	K00798	35.70	243.1	6.8e-73	cob(I)alamin adenosyltransferase [EC:2.5.1.17]
CP004065.1_776	K08217	225.00	295.6	1.1e-88	MFS transporter, DHA3 family, macrolide efflux protein
CP004065.1_777	K11619	49.83	115.0	3.9e-34	lia operon protein LiaI
CP004065.1_778	K11620	193.03	217.4	3.5e-65	lia operon protein LiaH
CP004065.1_778	K03969	84.50	145.5	6.1e-43	phage shock protein A
CP004065.1_779	K11621	70.10	230.6	4.9e-69	lia operon protein LiaG
CP004065.1_780	K11622	83.40	236.7	6.4e-71	lia operon protein LiaF
CP004065.1_781	K11617	255.57	396.4	2.9e-119	two-component system, NarL family, sensor histidine kinase LiaS [EC:2.7.13.3]
CP004065.1_782	K11618	268.20	278.6	1.3e-83	two-component system, NarL family, response regulator LiaR
CP004065.1_783	K06290	431.80	573.7	3.2e-173	spore germination protein AC
CP004065.1_784	K06289	347.57	477.4	5.5e-144	spore germination protein AB
CP004065.1_785	K06288	612.40	768.9	3.9e-232	spore germination protein AA
CP004065.1_785	K06295	452.20	596.0	2.8e-179	spore germination protein KA
CP004065.1_787	K01679	639.40	860.9	2.5e-259	fumarate hydratase, class II [EC:4.2.1.2]
CP004065.1_790	K07650	247.40	538.4	5.6e-162	two-component system, OmpR family, sensor histidine kinase CssS [EC:2.7.13.3]
CP004065.1_791	K07770	272.90	341.4	4.9e-103	two-component system, OmpR family, response regulator CssR
CP004065.1_793	K04047	77.30	183.7	1.3e-54	starvation-inducible DNA-binding protein
CP004065.1_795	K08602	113.03	454.8	1.3e-136	oligoendopeptidase F [EC:3.4.24.-]
CP004065.1_797	K23188	469.87	482.3	5.8e-145	iron-siderophore transport system ATP-binding protein [EC:7.2.2.17 7.2.2.-]
CP004065.1_797	K25130	419.90	467.1	2.8e-141	iron-siderophore transport system ATP-binding protein [EC:7.2.2.-]
CP004065.1_797	K02013	238.30	327.9	2.2e-98	iron complex transport system ATP-binding protein [EC:7.2.2.-]
CP004065.1_799	K00077	75.90	235.1	3.7e-70	2-dehydropantoate 2-reductase [EC:1.1.1.169]
CP004065.1_800	K01574	109.27	383.6	7.9e-116	acetoacetate decarboxylase [EC:4.1.1.4]
CP004065.1_806	K00318	179.80	210.7	8.5e-63	proline dehydrogenase [EC:1.5.5.2]
CP004065.1_807	K07516	434.53	544.4	9.8e-164	3-hydroxyacyl-CoA dehydrogenase [EC:1.1.1.35]
CP004065.1_808	K00632	515.37	545.4	1.7e-164	acetyl-CoA acyltransferase [EC:2.3.1.16]
CP004065.1_811	K02437	35.70	193.3	8.5e-58	glycine cleavage system H protein
CP004065.1_813	K07476	82.00	165.7	7.9e-50	toprim domain protein
CP004065.1_816	K02071	332.87	517.3	8.4e-156	D-methionine transport system ATP-binding protein
CP004065.1_817	K02072	119.67	308.5	9.1e-93	D-methionine transport system permease protein
CP004065.1_818	K02073	57.57	328.9	1.3e-98	D-methionine transport system substrate-binding protein
CP004065.1_819	K01607	17.10	25.0	2.8e-06	4-carboxymuconolactone decarboxylase [EC:4.1.1.44]

CP004065.1_820	K09013	161.43	421.2	4.3e-127	Fe-S cluster assembly ATP-binding protein
CP004065.1_821	K09015	272.00	330.5	4.6e-99	Fe-S cluster assembly protein SufD
CP004065.1_822	K11717	566.53	713.4	5.5e-215	cysteine desulfurase / selenocysteine lyase [EC:2.8.1.7 4.4.1.16]
CP004065.1_823	K04488	144.00	194.7	3.8e-58	nitrogen fixation protein NifU and related proteins
CP004065.1_824	K09014	377.87	823.0	3.2e-248	Fe-S cluster assembly protein SufB
CP004065.1_825	K01233	36.97	274.9	3.3e-82	chitosanase [EC:3.2.1.132]
CP004065.1_829	K26996	27.07	37.0	6e-10	immunity protein, SdpI family
CP004065.1_831	K10708	341.97	497.4	3.9e-150	fructoselysine 6-phosphate deglycase [EC:3.5.-.-]
CP004065.1_832	K10117	246.87	351.0	4e-105	raffinose/stachyose/melibiose transport system substrate-binding protein
CP004065.1_832	K02027	193.17	235.2	4.2e-70	multiple sugar transport system substrate-binding protein
CP004065.1_833	K10118	333.37	372.1	6.6e-112	raffinose/stachyose/melibiose transport system permease protein
CP004065.1_833	K02025	276.90	325.3	9.8e-98	multiple sugar transport system permease protein
CP004065.1_834	K10119	296.93	354.5	1.2e-106	raffinose/stachyose/melibiose transport system permease protein
CP004065.1_834	K02026	280.30	310.7	2.8e-93	multiple sugar transport system permease protein
CP004065.1_835	K10710	176.40	356.6	1.8e-107	fructoselysine 6-kinase [EC:2.7.1.218]
CP004065.1_836	K10711	243.70	369.7	1.2e-111	GntR family transcriptional regulator, frlABCD operon transcriptional regulator
CP004065.1_836	K03710	178.10	207.8	7e-62	GntR family transcriptional regulator
CP004065.1_837	K10112	546.20	581.2	5.3e-175	multiple sugar transport system ATP-binding protein [EC:7.5.2.-]
CP004065.1_839	K02083	464.77	480.8	1.6e-144	allantoate deiminase [EC:3.5.3.9]
CP004065.1_840	K00839	545.70	692.0	8.7e-209	(S)-ureidoglycine---glyoxylate transaminase [EC:2.6.1.112]
CP004065.1_840	K00830	397.67	432.5	5.3e-130	alanine-glyoxylate transaminase / serine-glyoxylate transaminase / serine-pyruvate transaminase [EC:2.6.1.44 2.6.1.45 2.6.1.51]
CP004065.1_841	K08153	415.17	507.8	3.3e-153	MFS transporter, DHA1 family, multidrug resistance protein
CP004065.1_843	K09684	188.00	422.5	7.7e-127	PucR family transcriptional regulator, purine catabolism regulatory protein
CP004065.1_844	K01466	373.03	542.4	4.3e-163	allantoinase [EC:3.5.2.5]
CP004065.1_847	K07090	37.03	187.3	1.1e-55	uncharacterized protein
CP004065.1_850	K21472	117.40	210.9	5.3e-63	peptidoglycan LD-endopeptidase LytH [EC:3.4.-.-]
CP004065.1_851	K03644	109.93	494.6	7.2e-149	lipoyl synthase [EC:2.8.1.8]
CP004065.1_854	K02566	258.43	276.8	8.4e-83	5'-nucleotidase [EC:3.1.3.5]
CP004065.1_857	K00003	285.10	553.6	1.6e-166	homoserine dehydrogenase [EC:1.1.1.3]
CP004065.1_858	K01733	243.03	439.1	5.5e-132	threonine synthase [EC:4.2.3.1]
CP004065.1_859	K00872	123.40	319.2	8e-96	homoserine kinase [EC:2.7.1.39]
CP004065.1_860	K01303	141.47	226.6	1.4e-67	acylaminoacyl-peptidase [EC:3.4.19.1]
CP004065.1_861	K22074	86.17	92.7	5.3e-27	NFU1 iron-sulfur cluster scaffold homolog, mitochondrial
CP004065.1_863	K03885	203.30	294.9	3.3e-88	NADH:quinone reductase (non-electrogenic) [EC:1.6.5.9]
CP004065.1_865	K03317	582.20	628.3	4.2e-189	concentrative nucleoside transporter, CNT family
CP004065.1_866	K01778	61.00	341.7	1.1e-102	diaminopimelate epimerase [EC:5.1.1.7]
CP004065.1_867	K13628	138.77	155.6	2.7e-46	iron-sulfur cluster assembly protein
CP004065.1_868	K14189	194.93	329.2	4.7e-99	uncharacterized oxidoreductase [EC:1.-.-.-]
CP004065.1_874	K06384	34.00	92.6	6.2e-27	stage II sporulation protein M
CP004065.1_878	K21567	376.93	514.2	3.1e-155	ferredoxin/flavodoxin---NADP+ reductase [EC:1.18.1.2 1.19.1.1]
CP004065.1_879	K03885	203.30	426.7	4.3e-128	NADH:quinone reductase (non-electrogenic) [EC:1.6.5.9]
CP004065.1_882	K09775	76.70	197.8	3.6e-59	uncharacterized protein
CP004065.1_883	K01255	422.07	549.0	4e-165	leucyl aminopeptidase [EC:3.4.11.1]
CP004065.1_884	K07084	342.63	674.4	6.1e-203	putative amino acid transporter
CP004065.1_885	K03523	54.23	146.3	2.2e-43	biotin transport system substrate-specific component
CP004065.1_887	K07017	254.87	376.4	4.5e-113	ferri-bacillibactin esterase [EC:3.1.-.-]
CP004065.1_888	K00216	256.33	416.8	2.1e-125	2,3-dihydro-2,3-dihydroxybenzoate dehydrogenase [EC:1.3.1.28]
CP004065.1_889	K02361	375.43	479.9	1.8e-144	isochorismate synthase [EC:5.4.4.2]
CP004065.1_890	K02363	778.17	941.8	2.9e-284	2,3-dihydroxybenzoate---[aryl-carrier protein] ligase [EC:6.3.2.14 6.2.1.71]
CP004065.1_891	K01252	314.80	448.3	2e-135	bifunctional isochorismate lyase / aryl carrier protein [EC:3.3.2.1 6.3.2.14]
CP004065.1_892	K04780	2024.83	3821.6	0	glyine---[glycyl-carrier protein] ligase [EC:6.2.1.66]
CP004065.1_892	K16118	1359.93	1444.8	0	pristinamycin I synthase 3 and 4
CP004065.1_893	K05375	34.70	108.9	2.8e-32	MbtH protein
CP004065.1_895	K00259	287.10	605.4	1.2e-182	alanine dehydrogenase [EC:1.4.1.1]
CP004065.1_896	K23253	221.73	266.9	6e-80	PucR family transcriptional regulator, proline-responsive transcriptional activator
CP004065.1_897	K14956	27.87	40.7	6.8e-11	ESAT-6 family protein

CP004065.1_900	K03466	191.80	223.3	1.4e-66	DNA segregation ATPase FtsK/SpoIIIE, S-DNA-T family
CP004065.1_903	K16216	195.37	294.5	2.6e-88	benzil reductase ((S)-benzoin forming) [EC:1.1.1.320]
CP004065.1_910	K08281	104.80	114.5	1.4e-33	nicotinamidase/pyrazinamidase [EC:3.5.1.19 3.5.1.-]
CP004065.1_911	K00763	105.10	597.5	9.3e-180	nicotinate phosphoribosyltransferase [EC:6.3.4.21]
CP004065.1_912	K07181	195.00	414.6	9.6e-125	c-di-GMP phosphodiesterase [EC:3.1.4.52]
CP004065.1_916	K02251	188.87	334.1	1.8e-100	competence protein ComQ
CP004065.1_917	K02253	41.17	75.1	5.5e-22	competence protein ComX
CP004065.1_918	K07680	232.70	761.0	3.2e-229	two-component system, NarL family, sensor histidine kinase ComP [EC:2.7.13.3]
CP004065.1_919	K07691	194.87	272.2	5.5e-82	two-component system, NarL family, competent response regulator ComA
CP004065.1_920	K19222	83.07	164.9	4.4e-49	1,4-dihydroxy-2-naphthoyl-CoA hydrolase [EC:3.1.2.28]
CP004065.1_921	K05571	107.83	124.9	5.6e-37	multicomponent Na+:H+ antiporter subunit G
CP004065.1_922	K05570	77.63	99.1	4.5e-29	multicomponent Na+:H+ antiporter subunit F
CP004065.1_923	K05569	112.73	139.6	2.6e-41	multicomponent Na+:H+ antiporter subunit E
CP004065.1_924	K05568	402.87	492.3	4.4e-148	multicomponent Na+:H+ antiporter subunit D
CP004065.1_925	K05567	122.63	155.9	4.5e-46	multicomponent Na+:H+ antiporter subunit C
CP004065.1_926	K05566	120.23	143.7	1.1e-42	multicomponent Na+:H+ antiporter subunit B
CP004065.1_927	K05565	689.50	948.1	1.6e-285	multicomponent Na+:H+ antiporter subunit A
CP004065.1_929	K23536	183.10	395.1	6.4e-119	general nucleoside transport system permease protein
CP004065.1_930	K23535	209.57	422.6	4.9e-127	general nucleoside transport system permease protein
CP004065.1_931	K23537	596.97	828.7	1.2e-249	general nucleoside transport system ATP-binding protein
CP004065.1_932	K07335	118.73	367.2	3.5e-110	basic membrane protein A and related proteins
CP004065.1_933	K11615	291.70	330.8	8.6e-100	two-component system, CitB family, response regulator MalR
CP004065.1_934	K11614	633.27	706.1	5e-213	two-component system, CitB family, sensor histidine kinase MalK [EC:2.7.13.3]
CP004065.1_937	K18770	600.13	910.0	2.5e-274	penicillin-binding protein 4 [EC:2.4.99.28 3.4.16.4]
CP004065.1_938	K08161	244.40	500.9	6.4e-151	MFS transporter, DHA1 family, multidrug resistance protein
CP004065.1_939	K06348	150.63	292.3	4.4e-88	sporulation inhibitor KapD
CP004065.1_940	K06347	108.77	185.2	8.2e-56	kinase-associated protein B
CP004065.1_941	K07697	304.97	433.7	2.1e-130	two-component system, sporulation sensor kinase B [EC:2.7.13.3]
CP004065.1_942	K14155	287.10	559.6	1.9e-168	cysteine-S-conjugate beta-lyase [EC:4.4.1.13]
CP004065.1_946	K10907	500.80	588.2	2.9e-177	aminotransferase [EC:2.6.1.-]
CP004065.1_947	K07570	128.93	170.6	5.4e-51	general stress protein 13
CP004065.1_948	K09779	32.10	130.9	7.7e-39	uncharacterized protein
CP004065.1_949	K19955	440.33	612.1	6.7e-185	alcohol dehydrogenase [EC:1.1.1.-]
CP004065.1_950	K19955	440.33	588.9	7.5e-178	alcohol dehydrogenase [EC:1.1.1.-]
CP004065.1_952	K01810	196.17	308.0	3.7e-92	glucose-6-phosphate isomerase [EC:5.3.1.9]
CP004065.1_958	K06973	70.50	318.3	1.1e-95	uncharacterized protein
CP004065.1_959	K03699	305.47	501.0	8.8e-151	magnesium and cobalt exporter, CNNM family
CP004065.1_960	K01187	286.07	287.6	5.1e-86	alpha-glucosidase [EC:3.2.1.20]
CP004065.1_962	K00459	256.27	395.7	1.1e-118	nitronate monooxygenase [EC:1.13.12.16]
CP004065.1_964	K00686	95.80	291.7	1.5e-87	protein-glutamine gamma-glutamyltransferase [EC:2.3.2.13]
CP004065.1_965	K03406	65.50	308.8	1.9e-92	methyl-accepting chemotaxis protein
CP004065.1_966	K03406	65.50	312.7	1.2e-93	methyl-accepting chemotaxis protein
CP004065.1_967	K03406	65.50	333.7	5.2e-100	methyl-accepting chemotaxis protein
CP004065.1_968	K03406	65.50	312.0	1.9e-93	methyl-accepting chemotaxis protein
CP004065.1_969	K05520	175.93	243.1	9.5e-73	deglycase [EC:3.5.1.124]
CP004065.1_970	K22230	271.20	514.3	6.3e-155	scyllo-inositol 2-dehydrogenase (NADP+) [EC:1.1.1.-]
CP004065.1_972	K06153	46.03	314.4	2.5e-94	undecaprenyl-diphosphatase [EC:3.6.1.27]
CP004065.1_975	K06366	543.43	654.1	1.7e-197	response regulator aspartate phosphatase H [EC:3.1.-.-]
CP004065.1_980	K05916	302.67	554.3	6.4e-167	nitric oxide dioxygenase [EC:1.14.12.17]
CP004065.1_981	K02395	35.20	77.7	2.9e-22	peptidoglycan hydrolase FlgJ
CP004065.1_982	K03499	127.40	178.5	4.1e-53	trk/ktr system potassium uptake protein
CP004065.1_984	K22109	186.30	236.4	4.3e-71	HTH-type transcriptional regulator, glycine betaine synthesis regulator
CP004065.1_985	K00130	687.90	747.8	1.9e-225	betaine-aldehyde dehydrogenase [EC:1.2.1.8]
CP004065.1_986	K11440	584.67	810.0	6e-245	choline dehydrogenase [EC:1.1.1.1]
CP004065.1_990	K07192	64.60	500.5	1.8e-150	flotillin
CP004065.1_991	K16789	53.13	209.2	1.7e-62	thiamine transporter

CP004065.1_994	K05770	25.73	88.8	8.8e-26	translocator protein
CP004065.1_995	K06204	35.73	85.3	1.5e-24	RNA polymerase-binding transcription factor
CP004065.1_996	K02552	371.53	450.7	2.5e-135	menaquinone-specific isochorismate synthase [EC:5.4.4.2]
CP004065.1_997	K02551	363.53	720.4	8.4e-217	2-succinyl-5-enolpyruvyl-6-hydroxy-3-cyclohexene-1-carboxylate synthase [EC:2.2.1.9]
CP004065.1_998	K08680	244.40	307.0	2.6e-92	2-succinyl-6-hydroxy-2,4-cyclohexadiene-1-carboxylate synthase [EC:4.2.99.20]
CP004065.1_999	K01661	396.53	488.1	3.5e-147	naphthoate synthase [EC:4.1.3.36]
CP004065.1_1000	K01911	338.30	427.2	2.1e-128	o-succinylbenzoate---CoA ligase [EC:6.2.1.26]
CP004065.1_1001	K02549	213.50	319.7	8.2e-96	o-succinylbenzoate synthase [EC:4.2.1.113]
CP004065.1_1003	K00426	28.43	118.5	7.3e-35	cytochrome bd ubiquinol oxidase subunit II [EC:7.1.1.7]
CP004065.1_1004	K00425	142.17	425.8	6.7e-128	cytochrome bd ubiquinol oxidase subunit I [EC:7.1.1.7]
CP004065.1_1005	K02909	21.83	90.7	2e-26	large subunit ribosomal protein L31
CP004065.1_1006	K01673	19.40	68.1	1e-19	carbonic anhydrase [EC:4.2.1.1]
CP004065.1_1007	K08998	23.87	147.9	7.9e-44	uncharacterized protein
CP004065.1_1008	K07173	101.70	227.3	5.2e-68	S-ribosylhomocysteine lyase [EC:4.4.1.21]
CP004065.1_1011	K04047	77.30	183.0	2.1e-54	starvation-inducible DNA-binding protein
CP004065.1_1014	K02050	145.27	200.2	1.4e-59	NitT/TauT family transport system permease protein
CP004065.1_1016	K02051	157.20	177.8	1.1e-52	NitT/TauT family transport system substrate-binding protein
CP004065.1_1019	K01610	97.20	852.3	9e-257	phosphoenolpyruvate carboxykinase (ATP) [EC:4.1.1.49]
CP004065.1_1020	K00789	19.03	685.0	1.7e-206	S-adenosylmethionine synthetase [EC:2.5.1.6]
CP004065.1_1021	K01953	106.30	554.2	1.2e-166	asparagine synthase (glutamine-hydrolysing) [EC:6.3.5.4]
CP004065.1_1022	K03293	609.50	667.7	3.2e-201	amino acid transporter, AAT family
CP004065.1_1023	K01726	184.57	193.2	1.7e-57	gamma-carbonic anhydrase [EC:4.2.1.-]
CP004065.1_1024	K01048	195.10	215.2	3.5e-64	lysophospholipase [EC:3.1.1.5]
CP004065.1_1025	K16188	206.80	571.8	1.2e-172	tetraprenyl-beta-curcumene synthase [EC:4.2.3.130]
CP004065.1_1026	K01126	152.53	208.6	2.5e-62	glycerophosphoryl diester phosphodiesterase [EC:3.1.4.46]
CP004065.1_1029	K07139	198.50	491.1	6e-148	uncharacterized protein
CP004065.1_1032	K07979	106.93	178.4	1.7e-53	GntR family transcriptional regulator
CP004065.1_1033	K16921	296.27	362.4	2.9e-109	acetoin utilization transport system ATP-binding protein
CP004065.1_1034	K16919	63.27	159.2	3.9e-47	acetoin utilization transport system permease protein
CP004065.1_1035	K16919	63.27	161.7	7e-48	acetoin utilization transport system permease protein
CP004065.1_1036	K16919	63.27	134.4	1.3e-39	acetoin utilization transport system permease protein
CP004065.1_1037	K16920	348.57	414.7	1.7e-125	acetoin utilization transport system ATP-binding protein
CP004065.1_1037	K02003	292.97	326.8	4.4e-98	putative ABC transport system ATP-binding protein
CP004065.1_1038	K16918	402.73	611.3	2.4e-184	acetoin utilization transport system permease protein
CP004065.1_1038	K02004	52.33	119.6	2.7e-35	putative ABC transport system permease protein
CP004065.1_1039	K11630	356.10	417.8	2e-126	two-component system, OmpR family, bacitracin resistance response regulator BceR
CP004065.1_1040	K11629	365.30	515.3	1.5e-155	two-component system, OmpR family, bacitracin resistance sensor histidine kinase BceS [EC:2.7.13.3]
CP004065.1_1041	K11631	388.27	409.8	7.7e-124	bacitracin transport system ATP-binding protein
CP004065.1_1041	K02003	292.97	306.9	4.5e-92	putative ABC transport system ATP-binding protein
CP004065.1_1042	K11632	471.07	609.4	2.4e-183	bacitracin transport system permease protein
CP004065.1_1045	K17763	101.07	119.6	4.5e-35	rsbT co-antagonist protein RsbR
CP004065.1_1047	K01869	385.87	1291.0	0	leucyl-tRNA synthetase [EC:6.1.1.4]
CP004065.1_1050	K07406	369.43	708.8	1.5e-213	alpha-galactosidase [EC:3.2.1.22]
CP004065.1_1051	K10119	296.93	315.0	1.2e-94	raffinose/stachyose/melibiose transport system permease protein
CP004065.1_1052	K10118	333.37	340.9	1.9e-102	raffinose/stachyose/melibiose transport system permease protein
CP004065.1_1052	K02025	276.90	299.5	6.8e-90	multiple sugar transport system permease protein
CP004065.1_1053	K10117	246.87	255.5	3.4e-76	raffinose/stachyose/melibiose transport system substrate-binding protein
CP004065.1_1056	K05020	674.73	753.6	2.1e-227	glycine betaine transporter
CP004065.1_1059	K06183	293.40	355.7	6e-107	16S rRNA pseudouridine516 synthase [EC:5.4.99.19]
CP004065.1_1063	K06901	93.90	528.1	5.2e-159	adenine/guanine/hypoxanthine permease
CP004065.1_1064	K01439	285.40	332.2	1.6e-99	succinyl-diaminopimelate desuccinylase [EC:3.5.1.18]
CP004065.1_1065	K01738	426.93	443.0	3.1e-133	cysteine synthase [EC:2.5.1.47]
CP004065.1_1066	K01975	64.70	132.9	3.3e-39	RNA 2',3'-cyclic 3'-phosphodiesterase [EC:3.1.4.58]
CP004065.1_1068	K07029	147.20	192.2	2.8e-57	diacylglycerol kinase (ATP) [EC:2.7.1.107]
CP004065.1_1071	K03439	68.23	194.8	6.9e-58	tRNA (guanine-N7-)-methyltransferase [EC:2.1.1.33]
CP004065.1_1073	K00029	287.77	634.6	3.9e-191	malate dehydrogenase (oxaloacetate-decarboxylating)(NADP+) [EC:1.1.1.40]

CP004065.1_1073	K00027	507.30	571.5	7.2e-172	malate dehydrogenase (oxaloacetate-decarboxylating) [EC:1.1.1.38]
CP004065.1_1075	K01261	363.67	522.4	9.1e-158	glutamyl aminopeptidase [EC:3.4.11.7]
CP004065.1_1080	K03466	191.80	782.7	1.7e-235	DNA segregation ATPase FtsK/SpoIIIE, S-DNA-T family
CP004065.1_1081	K01924	374.10	462.6	4.9e-139	UDP-N-acetylmuramate--alanine ligase [EC:6.3.2.8]
CP004065.1_1084	K26959	82.33	140.3	1.4e-41	monothiol bacilliredoxin
CP004065.1_1085	K13853	465.03	644.4	9.5e-195	3-deoxy-7-phosphoheptulonate synthase / chorismate mutase [EC:2.5.1.54 5.4.99.5]
CP004065.1_1086	K02529	268.37	371.1	2.2e-111	LacI family transcriptional regulator, galactose operon repressor
CP004065.1_1087	K02556	81.20	311.4	2e-93	chemotaxis protein MotA
CP004065.1_1088	K02557	144.00	209.3	1.7e-62	chemotaxis protein MotB
CP004065.1_1089	K04768	357.53	574.5	8.5e-173	acetoin utilization protein AcuC
CP004065.1_1090	K04767	140.13	184.1	6.9e-55	acetoin utilization protein AcuB
CP004065.1_1091	K04766	139.97	339.8	7.8e-103	acetoin utilization protein AcuA [EC:2.3.1.-]
CP004065.1_1093	K01866	88.87	424.4	2e-127	tyrosyl-tRNA synthetase [EC:6.1.1.1]
CP004065.1_1094	K26606	27.30	63.8	3.1e-18	branched chain amino acid efflux pump
CP004065.1_1095	K26605	54.60	224.0	4.9e-67	branched chain amino acid efflux pump
CP004065.1_1096	K23356	46.03	170.3	1.3e-50	HTH-type transcriptional regulator, sugar sensing transcriptional regulator
CP004065.1_1097	K13283	187.13	303.4	3.6e-91	ferrous-iron efflux pump FieF
CP004065.1_1100	K05520	175.93	240.5	5.8e-72	deglycase [EC:3.5.1.124]
CP004065.1_1102	K05916	302.67	548.1	4.9e-165	nitric oxide dioxygenase [EC:1.14.12.17]
CP004065.1_1105	K02986	67.73	209.6	2e-62	small subunit ribosomal protein S4
CP004065.1_1107	K08968	91.93	253.1	7.6e-76	L-methionine (R)-S-oxide reductase [EC:1.8.4.14]
CP004065.1_1109	K04486	126.10	247.7	4.5e-74	histidinol-phosphatase (PHP family) [EC:3.1.3.15]
CP004065.1_1110	K06286	148.63	561.4	3.8e-169	septation ring formation regulator
CP004065.1_1111	K03311	209.80	570.3	6.7e-172	branched-chain amino acid:cation transporter, LIVCS family
CP004065.1_1112	K04487	287.40	450.0	2.3e-135	cysteine desulfurase [EC:2.8.1.7]
CP004065.1_1113	K03151	126.60	542.3	3.6e-163	tRNA uracil 4-sulfurtransferase [EC:2.8.1.4]
CP004065.1_1114	K06419	107.57	109.0	2e-32	small acid-soluble spore protein B (major beta-type SASP)
CP004065.1_1114	K06418	101.07	108.2	3.3e-32	small acid-soluble spore protein A (major alpha-type SASP)
CP004065.1_1117	K00858	38.63	104.8	1.1e-30	NAD+ kinase [EC:2.7.1.23]
CP004065.1_1118	K04773	182.47	320.0	6.5e-96	protease IV [EC:3.4.21.-]
CP004065.1_1122	K11065	132.27	263.7	3.3e-79	thioredoxin-dependent peroxiredoxin [EC:1.11.1.24]
CP004065.1_1124	K00925	190.80	652.8	1.9e-196	acetate kinase [EC:2.7.2.1]
CP004065.1_1125	K03638	158.63	252.8	9.5e-76	molybdopterin adenylyltransferase [EC:2.7.7.75]
CP004065.1_1126	K01940	115.50	620.7	8e-187	argininosuccinate synthase [EC:6.3.4.5]
CP004065.1_1127	K01755	169.37	748.6	2.1e-225	argininosuccinate lyase [EC:4.3.2.1]
CP004065.1_1133	K06881	205.10	308.6	1.3e-92	bifunctional oligoribonuclease and PAP phosphatase NrnA [EC:3.1.3.7 3.1.13.3]
CP004065.1_1137	K02337	932.93	1412.5	0	DNA polymerase III subunit alpha [EC:2.7.7.7]
CP004065.1_1138	K00027	507.30	629.5	2e-189	malate dehydrogenase (oxaloacetate-decarboxylating) [EC:1.1.1.38]
CP004065.1_1138	K00029	287.77	311.3	2.6e-93	malate dehydrogenase (oxaloacetate-decarboxylating)(NADP+) [EC:1.1.1.40]
CP004065.1_1139	K01963	380.60	446.6	2.6e-134	acetyl-CoA carboxylase carboxyl transferase subunit beta [EC:6.4.1.2 2.1.3.15]
CP004065.1_1140	K01962	408.77	565.0	7.9e-170	acetyl-CoA carboxylase carboxyl transferase subunit alpha [EC:6.4.1.2 2.1.3.15]
CP004065.1_1141	K00850	359.57	417.9	1.8e-125	6-phosphofructokinase 1 [EC:2.7.1.11]
CP004065.1_1142	K00873	49.90	694.1	4e-209	pyruvate kinase [EC:2.7.1.40]
CP004065.1_1143	K07113	64.97	134.3	7.8e-40	UPF0716 protein FxsA
CP004065.1_1147	K01659	471.93	573.8	5.1e-173	2-methylcitrate synthase [EC:2.3.3.5]
CP004065.1_1148	K00031	117.90	188.8	4.4e-56	isocitrate dehydrogenase [EC:1.1.1.42]
CP004065.1_1149	K00024	377.73	408.5	8e-123	malate dehydrogenase [EC:1.1.1.37]
CP004065.1_1150	K07658	338.17	367.2	1.4e-110	two-component system, OmpR family, alkaline phosphatase synthesis response regulator PhoP
CP004065.1_1150	K02483	242.00	283.6	6.9e-85	two-component system, OmpR family, response regulator
CP004065.1_1151	K07636	310.93	395.7	6.2e-119	two-component system, OmpR family, phosphate regulon sensor histidine kinase PhoR [EC:2.7.13.3]
CP004065.1_1152	K02335	481.67	1084.9	0	DNA polymerase I [EC:2.7.7.7]
CP004065.1_1153	K10563	196.13	354.3	2.2e-106	formamidopyrimidine-DNA glycosylase [EC:3.2.2.23 4.2.99.18]
CP004065.1_1154	K23242	61.23	96.6	3.1e-28	manganese efflux pump family protein
CP004065.1_1155	K00859	53.27	300.8	5.2e-90	dephospho-CoA kinase [EC:2.7.1.24]
CP004065.1_1156	K23257	397.27	460.8	2.6e-139	methylglyoxal/glyoxal reductase [EC:1.1.1.283 1.1.1.-]
CP004065.1_1156	K06221	349.23	420.0	1.1e-126	2,5-diketo-D-gluconate reductase A [EC:1.1.1.346]

CP004065.1_1159	K00134	414.10	505.8	2.8e-152	glyceraldehyde 3-phosphate dehydrogenase (phosphorylating) [EC:1.2.1.12]
CP004065.1_1159	K00150	349.40	384.2	1e-115	glyceraldehyde-3-phosphate dehydrogenase (NAD(P)+) (phosphorylating) [EC:1.2.1.59]
CP004065.1_1160	K01611	16.97	48.4	1.6e-13	S-adenosylmethionine decarboxylase [EC:4.1.1.50]
CP004065.1_1161	K07738	41.80	232.1	1.2e-69	transcriptional repressor NrdR
CP004065.1_1162	K03346	83.40	460.5	1.7e-138	replication initiation and membrane attachment protein
CP004065.1_1163	K11144	162.27	388.7	5.8e-117	primosomal protein DnaI
CP004065.1_1166	K01868	151.50	872.9	6.8e-263	threonyl-tRNA synthetase [EC:6.1.1.3]
CP004065.1_1167	K25528	212.23	418.7	1.5e-126	phosphoserine phosphatase [EC:3.1.3.3]
CP004065.1_1167	K07025	112.47	129.4	3.5e-38	putative hydrolase of the HAD superfamily
CP004065.1_1168	K07704	616.23	790.3	2e-238	two-component system, LytTR family, sensor histidine kinase LytS [EC:2.7.13.3]
CP004065.1_1169	K07705	281.60	315.7	4.4e-95	two-component system, LytTR family, response regulator LytT
CP004065.1_1169	K02477	121.47	243.0	1.1e-72	two-component system, LytTR family, response regulator
CP004065.1_1170	K05338	139.97	186.7	3.7e-56	holin-like protein
CP004065.1_1170	K06518	37.43	104.7	9.7e-31	holin-like protein
CP004065.1_1171	K05339	288.17	348.7	3.1e-105	holin-like protein LrgB
CP004065.1_1172	K02520	34.97	261.3	2.6e-78	translation initiation factor IF-3
CP004065.1_1173	K02916	23.07	54.6	2.6e-15	large subunit ribosomal protein L35
CP004065.1_1174	K02887	45.33	208.2	3.8e-62	large subunit ribosomal protein L20
CP004065.1_1177	K01261	363.67	414.8	4.6e-125	glutamyl aminopeptidase [EC:3.4.11.7]
CP004065.1_1177	K20608	364.13	399.7	1.7e-120	tetrahedral aminopeptidase [EC:3.4.11.-]
CP004065.1_1178	K06113	228.80	351.4	1.9e-105	arabinan endo-1,5-alpha-L-arabinosidase [EC:3.2.1.99]
CP004065.1_1179	K01804	182.77	695.4	6.8e-210	L-arabinose isomerase [EC:5.3.1.4]
CP004065.1_1180	K00853	372.30	762.5	8e-230	L-ribulokinase [EC:2.7.1.16]
CP004065.1_1181	K03077	237.90	332.9	4.8e-100	L-ribulose-5-phosphate 4-epimerase [EC:5.1.3.4]
CP004065.1_1182	K02101	353.40	462.9	4.5e-140	sugar-phosphatase [EC:3.1.3.23]
CP004065.1_1183	K00096	164.17	423.8	2.2e-127	glycerol-1-phosphate dehydrogenase [NAD(P)+] [EC:1.1.1.261]
CP004065.1_1184	K17234	307.53	604.0	2.8e-182	arabinooligosaccharide transport system substrate-binding protein
CP004065.1_1184	K02027	193.17	206.7	1.8e-61	multiple sugar transport system substrate-binding protein
CP004065.1_1185	K17235	341.27	444.6	3.9e-134	arabinooligosaccharide transport system permease protein
CP004065.1_1185	K02025	276.90	307.9	1.9e-92	multiple sugar transport system permease protein
CP004065.1_1186	K17236	335.93	420.2	8.1e-127	arabinooligosaccharide transport system permease protein
CP004065.1_1186	K02026	280.30	302.9	6.6e-91	multiple sugar transport system permease protein
CP004065.1_1187	K01209	85.37	449.0	7.7e-135	alpha-L-arabinofuranosidase [EC:3.2.1.55]
CP004065.1_1188	K06200	289.50	658.3	2.5e-198	carbon starvation protein
CP004065.1_1189	K11473	280.10	525.6	2.5e-158	glycolate dehydrogenase iron-sulfur subunit [EC:1.1.99.14]
CP004065.1_1190	K00104	535.33	694.3	3.4e-209	glycolate dehydrogenase FAD-linked subunit [EC:1.1.99.14]
CP004065.1_1191	K02647	173.57	329.9	5.4e-99	carbohydrate diacid regulator
CP004065.1_1192	K06426	67.10	112.1	2.3e-33	small acid-soluble spore protein I (minor)
CP004065.1_1193	K03437	214.63	299.1	9.3e-90	RNA methyltransferase, TrmH family
CP004065.1_1194	K01889	95.27	386.7	4.4e-116	phenylalanyl-tRNA synthetase alpha chain [EC:6.1.1.20]
CP004065.1_1195	K01890	122.80	635.7	2.7e-191	phenylalanyl-tRNA synthetase beta chain [EC:6.1.1.20]
CP004065.1_1196	K03471	231.93	389.4	2.6e-117	ribonuclease HIII [EC:3.1.26.4]
CP004065.1_1197	K09888	23.30	87.5	2.2e-25	cell division protein ZapA
CP004065.1_1198	K03558	39.73	74.0	2.9e-21	membrane protein required for colicin V production
CP004065.1_1199	K02347	338.50	774.5	2.3e-233	DNA polymerase (family X)
CP004065.1_1200	K07456	353.83	926.2	6.9e-279	DNA mismatch repair protein MutS2
CP004065.1_1201	K08989	42.47	157.5	4.4e-47	putative membrane protein
CP004065.1_1203	K01897	430.97	462.7	4.9e-139	long-chain acyl-CoA synthetase [EC:6.2.1.3]
CP004065.1_1204	K13770	123.73	251.8	1.9e-75	TetR/AcrR family transcriptional regulator, fatty acid metabolism regulator protein
CP004065.1_1205	K13767	317.23	371.3	4.6e-112	enoyl-CoA hydratase [EC:4.2.1.17]
CP004065.1_1206	K03521	77.00	290.5	5.3e-87	electron transfer flavoprotein beta subunit
CP004065.1_1207	K03522	225.07	405.2	1.3e-121	electron transfer flavoprotein alpha subunit
CP004065.1_1208	K01209	85.37	461.4	1.3e-138	alpha-L-arabinofuranosidase [EC:3.2.1.55]
CP004065.1_1209	K03671	112.43	151.1	1.1e-44	thioredoxin
CP004065.1_1210	K03703	236.70	761.5	1.9e-229	excinuclease ABC subunit C
CP004065.1_1211	K00928	394.00	507.3	9.7e-153	aspartate kinase [EC:2.7.2.4]

CP004065.1_1213	K00241	50.80	125.2	7.7e-37	succinate dehydrogenase cytochrome b subunit
CP004065.1_1214	K00239	665.27	716.8	4.6e-216	succinate dehydrogenase flavoprotein subunit [EC:1.3.5.1]
CP004065.1_1215	K00240	157.40	244.3	3.2e-73	succinate dehydrogenase iron-sulfur subunit [EC:1.3.5.1]
CP004065.1_1216	K07107	72.43	100.1	4.9e-29	acyl-CoA thioester hydrolase [EC:3.1.2.-]
CP004065.1_1217	K01994	89.63	137.3	2.6e-41	LuxR family transcriptional regulator, transcriptional regulator of spore coat protein
CP004065.1_1219	K01776	98.97	347.6	2e-104	glutamate racemase [EC:5.1.1.3]
CP004065.1_1220	K06298	94.10	338.3	1.4e-101	germination protein M
CP004065.1_1221	K00989	189.03	443.5	1.6e-133	ribonuclease PH [EC:2.7.7.56]
CP004065.1_1222	K01519	53.90	265.1	2.6e-79	XTP/dITP diphosphohydrolase [EC:3.6.1.66]
CP004065.1_1223	K07095	94.43	131.5	5.8e-39	uncharacterized protein
CP004065.1_1226	K01652	517.70	788.8	9.5e-238	acetolactate synthase I/II/III large subunit [EC:2.2.1.6]
CP004065.1_1227	K01653	49.70	222.3	1e-66	acetolactate synthase I/III small subunit [EC:2.2.1.6]
CP004065.1_1228	K00053	107.87	514.5	8.6e-155	ketol-acid reductoisomerase [EC:1.1.1.86]
CP004065.1_1229	K01649	498.33	640.8	1e-192	2-isopropylmalate synthase [EC:2.3.3.13]
CP004065.1_1230	K00052	432.33	603.6	1.1e-181	3-isopropylmalate dehydrogenase [EC:1.1.1.85]
CP004065.1_1231	K01703	454.33	655.5	1.4e-197	3-isopropylmalate/(R)-2-methylmalate dehydratase large subunit [EC:4.2.1.33 4.2.1.35]
CP004065.1_1232	K01704	131.03	267.7	3e-80	3-isopropylmalate/(R)-2-methylmalate dehydratase small subunit [EC:4.2.1.33 4.2.1.35]
CP004065.1_1234	K03545	69.90	478.2	8.5e-144	trigger factor
CP004065.1_1235	K03544	145.17	649.6	1.1e-195	ATP-dependent Clp protease ATP-binding subunit ClpX
CP004065.1_1236	K04076	407.83	524.3	1.2e-157	ATP-dependent Lon protease [EC:3.4.21.53]
CP004065.1_1237	K01338	863.10	1107.4	0	ATP-dependent Lon protease [EC:3.4.21.53]
CP004065.1_1238	K03978	95.90	269.8	1.1e-80	GTP-binding protein
CP004065.1_1240	K02492	71.10	531.4	6.1e-160	glutamyl-tRNA reductase [EC:1.2.1.70]
CP004065.1_1241	K02497	138.90	379.9	1.2e-114	HemX protein
CP004065.1_1242	K01749	105.33	464.3	8.8e-140	hydroxymethylbilane synthase [EC:2.5.1.61]
CP004065.1_1243	K01719	43.23	143.9	1.7e-42	uroporphyrinogen-III synthase [EC:4.2.1.75]
CP004065.1_1244	K01698	94.97	556.2	1.7e-167	porphobilinogen synthase [EC:4.2.1.24]
CP004065.1_1245	K01845	312.23	683.9	5.7e-206	glutamate-1-semialdehyde 2,1-aminomutase [EC:5.4.3.8]
CP004065.1_1246	K06417	121.47	263.6	5.1e-79	stage VI sporulation protein D
CP004065.1_1249	K01873	582.43	1331.2	0	valyl-tRNA synthetase [EC:6.1.1.9]
CP004065.1_1250	K11754	379.57	504.9	1.2e-151	dihydrofolate synthase / folylpolyglutamate synthase [EC:6.3.2.12 6.3.2.17]
CP004065.1_1251	K02654	116.93	164.1	1.2e-48	leader peptidase (prepilin peptidase) / N-methyltransferase [EC:3.4.23.43 2.1.1.-]
CP004065.1_1252	K06380	120.37	211.3	6e-63	stage II sporulation protein B
CP004065.1_1253	K06287	207.20	275.2	1.7e-82	nucleoside triphosphate pyrophosphatase [EC:3.6.1.-]
CP004065.1_1254	K03630	40.07	311.2	2.9e-93	DNA repair protein RadC
CP004065.1_1255	K03569	108.73	535.3	2e-161	rod shape-determining protein MreB and related proteins
CP004065.1_1256	K03570	52.93	218.6	4.3e-65	rod shape-determining protein MreC
CP004065.1_1257	K03571	31.87	76.5	5.9e-22	rod shape-determining protein MreD
CP004065.1_1258	K03610	34.13	148.8	4.8e-44	septum site-determining protein MinC
CP004065.1_1259	K03609	203.03	316.6	7.9e-95	septum site-determining protein MinD
CP004065.1_1260	K06401	141.23	265.0	1.6e-79	stage IV sporulation protein FA
CP004065.1_1261	K06402	102.07	262.3	1.4e-78	stage IV sporulation protein FB [EC:3.4.24.-]
CP004065.1_1262	K02888	28.73	141.6	5.9e-42	large subunit ribosomal protein L21
CP004065.1_1263	K07584	34.67	105.4	6.6e-31	uncharacterized protein
CP004065.1_1264	K02899	36.30	144.2	1e-42	large subunit ribosomal protein L27
CP004065.1_1265	K06375	74.23	185.6	2e-55	stage 0 sporulation protein B (sporulation initiation phosphotransferase) [EC:2.7.-.-]
CP004065.1_1266	K03979	149.20	547.0	8e-165	GTPase [EC:3.6.5.-]
CP004065.1_1267	K06209	123.30	208.4	8.1e-63	chorismate mutase [EC:5.4.99.5]
CP004065.1_1269	K07105	96.27	221.7	1.7e-66	uncharacterized protein
CP004065.1_1270	K04487	287.40	407.2	2.1e-122	cysteine desulfurase [EC:2.8.1.7]
CP004065.1_1271	K00278	526.70	635.5	2.3e-191	L-aspartate oxidase [EC:1.4.3.16]
CP004065.1_1272	K00767	217.50	378.2	1.1e-113	nicotinate-nucleotide pyrophosphorylase (carboxylating) [EC:2.4.2.19]
CP004065.1_1273	K03517	105.40	380.9	1.8e-114	quinolinate synthase [EC:2.5.1.72]
CP004065.1_1274	K06370	69.50	195.1	6.6e-58	morphogenetic protein associated with SpoVID
CP004065.1_1275	K06345	62.77	159.9	1.7e-47	spore cortex protein
CP004065.1_1277	K06318	82.87	178.9	1.6e-53	forespore regulator of the sigma-K checkpoint

CP004065.1_1278	K03550	46.37	220.6	5.7e-66	holliday junction DNA helicase RuvA
CP004065.1_1279	K03551	186.47	622.6	1e-187	holliday junction DNA helicase RuvB [EC:5.6.2.4]
CP004065.1_1281	K07568	161.50	518.5	2.7e-156	S-adenosylmethionine:tRNA ribosyltransferase-isomerase [EC:2.4.99.17]
CP004065.1_1282	K00773	528.43	652.9	7.5e-197	queuine tRNA-ribosyltransferase [EC:2.4.2.29]
CP004065.1_1283	K03210	32.57	128.3	9.4e-38	preprotein translocase subunit YajC
CP004065.1_1286	K06409	388.70	598.2	3.2e-180	stage V sporulation protein B
CP004065.1_1288	K12257	548.43	747.2	4.7e-225	SecD/SecF fusion protein
CP004065.1_1289	K07228	80.77	181.4	2.2e-54	K+:H+ antiporter subunit KhtT
CP004065.1_1290	K08992	32.97	70.0	5.9e-20	lipopolysaccharide assembly protein A
CP004065.1_1291	K07462	66.97	764.9	2.3e-230	single-stranded-DNA-specific exonuclease [EC:3.1.-.-]
CP004065.1_1292	K00759	92.70	237.5	3.9e-71	adenine phosphoribosyltransferase [EC:2.4.2.7]
CP004065.1_1293	K01139	801.03	1037.7	6.6e-313	GTP diphosphokinase / guanosine-3',5'-bis(diphosphate) 3'-diphosphatase [EC:2.7.6.5 3.1.7.2]
CP004065.1_1293	K00951	685.77	689.9	1.4e-207	GTP pyrophosphokinase [EC:2.7.6.5]
CP004065.1_1294	K07560	40.67	225.0	2.8e-67	D-aminoacyl-tRNA deacylase [EC:3.1.1.96]
CP004065.1_1295	K01448	29.13	205.8	1.9e-61	N-acetylmuramoyl-L-alanine amidase [EC:3.5.1.28]
CP004065.1_1298	K01892	275.30	389.2	6.3e-117	histidyl-tRNA synthetase [EC:6.1.1.21]
CP004065.1_1299	K01876	301.23	928.4	1.4e-279	aspartyl-tRNA synthetase [EC:6.1.1.12]
CP004065.1_1300	K22132	163.63	338.7	1.5e-101	tRNA threonylcarbamoyladenosine dehydratase
CP004065.1_1301	K07478	187.97	574.2	1e-172	putative ATPase
CP004065.1_1302	K17472	166.73	176.2	1e-52	Rrf2 family transcriptional regulator, cysteine metabolism repressor
CP004065.1_1303	K04487	287.40	540.1	1.1e-162	cysteine desulfurase [EC:2.8.1.7]
CP004065.1_1304	K00566	424.50	560.1	7.8e-169	tRNA-uridine 2-sulfurtransferase [EC:2.8.1.13]
CP004065.1_1306	K03581	242.43	483.0	3.5e-145	exodeoxyribonuclease V alpha subunit [EC:3.1.11.5]
CP004065.1_1310	K10040	255.97	279.4	6.6e-84	aspartate/glutamate/glutamine transport system permease protein
CP004065.1_1310	K02029	214.53	243.7	7.8e-73	polar amino acid transport system permease protein
CP004065.1_1311	K10040	255.97	314.9	1e-94	aspartate/glutamate/glutamine transport system permease protein
CP004065.1_1311	K02029	214.53	271.0	4e-81	polar amino acid transport system permease protein
CP004065.1_1312	K10039	279.17	392.6	1.8e-118	aspartate/glutamate/glutamine transport system substrate-binding protein
CP004065.1_1312	K02030	76.77	153.2	2.4e-45	polar amino acid transport system substrate-binding protein
CP004065.1_1313	K02028	386.70	452.3	4.8e-136	polar amino acid transport system ATP-binding protein [EC:7.4.2.1]
CP004065.1_1313	K10041	409.80	439.4	5e-133	aspartate/glutamate/glutamine transport system ATP-binding protein [EC:7.4.2.1]
CP004065.1_1315	K01872	199.53	1139.1	0	alanyl-tRNA synthetase [EC:6.1.1.7]
CP004065.1_1317	K07447	43.93	186.6	1.4e-55	putative pre-16S rRNA nuclease [EC:3.1.-.-]
CP004065.1_1319	K07082	42.70	354.9	1.1e-106	peptidoglycan lytic transglycosylase G [EC:4.2.2.29]
CP004065.1_1322	K08303	406.50	483.9	2e-145	U32 family peptidase [EC:3.4.-.-]
CP004065.1_1323	K00876	144.07	294.2	3.6e-88	uridine kinase [EC:2.7.1.48]
CP004065.1_1324	K14059	167.87	205.3	4.5e-61	integrase
CP004065.1_1342	K00558	166.00	260.9	4.3e-78	DNA (cytosine-5)-methyltransferase 1 [EC:2.1.1.37]
CP004065.1_1343	K06223	67.20	230.2	1.1e-68	DNA adenine methylase [EC:2.1.1.72]
CP004065.1_1350	K24072	90.50	181.4	2.3e-54	positive control factor
CP004065.1_1360	K06904	50.60	125.3	7.7e-37	Escherichia/Staphylococcus phage prohead protease
CP004065.1_1371	K02395	35.20	68.0	2.7e-19	peptidoglycan hydrolase FlgJ
CP004065.1_1377	K01447	118.50	240.5	5.5e-72	N-acetylmuramoyl-L-alanine amidase [EC:3.5.1.28]
CP004065.1_1382	K03624	159.57	214.9	2.7e-64	transcription elongation factor GreA
CP004065.1_1383	K21468	498.27	758.6	9.3e-229	penicillin-binding protein 4B
CP004065.1_1386	K17462	117.33	248.6	2.3e-74	putative AdoMet-dependent methyltransferase [EC:2.1.1.-]
CP004065.1_1387	K01243	103.73	268.1	2.9e-80	adenosylhomocysteine nucleosidase [EC:3.2.2.9]
CP004065.1_1388	K17216	447.97	488.7	1.2e-147	cystathionine beta-synthase (O-acetyl-L-serine) [EC:2.5.1.134]
CP004065.1_1389	K17217	607.37	633.9	1.5e-191	cystathionine gamma-lyase / homocysteine desulfhydrase [EC:4.4.1.1 4.4.1.2]
CP004065.1_1389	K01760	493.17	529.6	1.4e-159	cysteine-S-conjugate beta-lyase [EC:4.4.1.13]
CP004065.1_1392	K00123	847.33	1064.9	0	formate dehydrogenase major subunit [EC:1.17.1.9]
CP004065.1_1394	K21993	293.73	370.1	2.3e-111	formate transporter
CP004065.1_1397	K22106	149.30	204.6	4.5e-61	TetR/AcrR family transcriptional regulator, repressor of fatR-cypB operon
CP004065.1_1398	K14338	601.20	1469.5	0	cytochrome P450 / NADPH-cytochrome P450 reductase [EC:1.14.14.1 1.6.2.4]
CP004065.1_1399	K08224	171.37	525.5	2.8e-158	MFS transporter, YNFM family, putative membrane transport protein
CP004065.1_1400	K09681	263.80	266.9	4.3e-80	LysR family transcriptional regulator, transcription activator of glutamate synthase operon

CP004065.1_1401	K03976	99.40	194.3	7.6e-58	Cys-tRNA(Pro)/Cys-tRNA(Cys) deacylase [EC:3.1.1.-]
CP004065.1_1402	K13985	99.67	330.5	4.5e-99	N-acyl-phosphatidylethanolamine-hydrolysing phospholipase D [EC:3.1.4.54]
CP004065.1_1403	K02538	499.37	924.5	6.8e-279	mannose operon transcriptional activator
CP004065.1_1403	K03491	482.23	482.4	5.1e-145	probable licABCH operon transcriptional regulator
CP004065.1_1403	K03483	467.10	470.5	2.3e-141	mannitol operon transcriptional activator
CP004065.1_1404	K25814	717.47	1025.1	2.4e-309	mannose PTS system EIIBCA component [EC:2.7.1.191]
CP004065.1_1404	K02768	144.33	166.7	1.5e-49	fructose PTS system EIIA component [EC:2.7.1.202]
CP004065.1_1405	K01809	33.17	195.9	3.2e-58	mannose-6-phosphate isomerase [EC:5.3.1.8]
CP004065.1_1411	K19273	177.90	271.0	8.8e-82	streptothricin acetyltransferase [EC:2.3.-.-]
CP004065.1_1412	K08168	286.97	588.9	1.4e-177	MFS transporter, DHA2 family, metal-tetracycline-proton antiporter
CP004065.1_1415	K13653	208.93	322.9	7.9e-97	AraC family transcriptional regulator
CP004065.1_1418	K03091	189.70	335.9	4.9e-101	RNA polymerase sigma-E/F/G factor
CP004065.1_1421	K22278	73.27	258.3	3.1e-77	peptidoglycan-N-acetylglucosamine deacetylase [EC:3.5.1.104]
CP004065.1_1422	K10804	102.87	121.0	1.5e-35	acyl-CoA thioesterase I [EC:3.1.2.- 3.1.2.2 3.1.1.2 3.1.1.5]
CP004065.1_1424	K07015	105.17	267.8	2.6e-80	putative phosphatase [EC:3.1.3.-]
CP004065.1_1425	K06948	254.17	563.1	5.2e-170	30S ribosome assembly GTPase
CP004065.1_1426	K00014	294.53	344.9	1.7e-103	shikimate dehydrogenase [EC:1.1.1.25]
CP004065.1_1427	K07574	51.13	129.6	2.5e-38	RNA-binding protein
CP004065.1_1428	K00969	121.70	272.3	1.8e-81	nicotinate-nucleotide adenylyltransferase [EC:2.7.7.18]
CP004065.1_1430	K09710	113.50	162.0	3.7e-48	ribosome-associated protein
CP004065.1_1432	K02239	266.50	414.6	3.1e-125	competence protein ComER
CP004065.1_1433	K02237	75.73	191.3	6.7e-57	competence protein ComEA
CP004065.1_1434	K01493	80.20	168.4	5.8e-50	dCMP deaminase [EC:3.5.4.12]
CP004065.1_1435	K02238	64.77	519.0	6e-156	competence protein ComEC
CP004065.1_1437	K02340	96.93	286.6	8.9e-86	DNA polymerase III subunit delta [EC:2.7.7.7]
CP004065.1_1438	K02968	24.97	106.9	2.8e-31	small subunit ribosomal protein S20
CP004065.1_1439	K06012	187.50	478.8	1.6e-144	spore protease [EC:3.4.24.78]
CP004065.1_1440	K06385	107.47	333.3	5.6e-100	stage II sporulation protein P
CP004065.1_1442	K03596	927.43	1164.3	0	GTP-binding protein LepA
CP004065.1_1443	K02495	261.80	355.5	9.2e-107	oxygen-independent coproporphyrinogen III oxidase [EC:1.3.98.3]
CP004065.1_1444	K03705	67.57	395.0	1.2e-118	heat-inducible transcriptional repressor
CP004065.1_1445	K03687	31.27	203.6	1.1e-60	molecular chaperone GrpE
CP004065.1_1446	K04043	801.23	941.9	8e-284	molecular chaperone DnaK
CP004065.1_1447	K03686	406.37	529.9	2.2e-159	molecular chaperone DnaJ
CP004065.1_1448	K02687	125.73	331.6	1.6e-99	ribosomal protein L11 methyltransferase [EC:2.1.1.-]
CP004065.1_1449	K09761	57.73	254.2	3.6e-76	16S rRNA (uracil1498-N3)-methyltransferase [EC:2.1.1.193]
CP004065.1_1450	K18707	413.13	603.4	7.8e-182	threonylcarbamoyladenosine tRNA methylthiotransferase MtaB [EC:2.8.4.5]
CP004065.1_1451	K03324	151.43	304.1	4.5e-91	phosphate:Na+ symporter
CP004065.1_1452	K02970	28.27	64.7	2.2e-18	small subunit ribosomal protein S21
CP004065.1_1453	K09117	49.67	163.3	2e-48	uncharacterized protein
CP004065.1_1454	K07403	121.90	357.2	3.7e-107	membrane-bound serine protease (ClpP class)
CP004065.1_1458	K06438	146.87	442.5	3.6e-133	similar to stage IV sporulation protein
CP004065.1_1459	K06217	216.80	522.3	4.7e-157	phosphate starvation-inducible protein PhoH and related proteins
CP004065.1_1460	K07037	135.10	780.8	3.2e-235	cyclic-di-AMP phosphodiesterase PgpH [EC:3.1.4.-]
CP004065.1_1461	K07042	60.10	182.2	2.8e-54	probable rRNA maturation factor
CP004065.1_1462	K00887	148.73	169.2	1.4e-50	undecaprenol kinase [EC:2.7.1.66]
CP004065.1_1463	K01489	31.20	212.4	2.1e-63	cytidine deaminase [EC:3.5.4.5]
CP004065.1_1464	K03595	144.20	429.8	3.6e-129	GTPase
CP004065.1_1466	K03584	28.20	193.1	1.7e-57	DNA repair protein RecO (recombination protein O)
CP004065.1_1467	K01878	507.67	590.0	9.4e-178	glycyl-tRNA synthetase alpha chain [EC:6.1.1.14]
CP004065.1_1468	K01879	573.03	857.8	2.8e-258	glycyl-tRNA synthetase beta chain [EC:6.1.1.14]
CP004065.1_1469	K23774	112.50	332.4	2.7e-100	DeoR family transcriptional regulator, catabolite repression regulator
CP004065.1_1470	K09773	264.67	346.9	2.3e-104	[pyruvate, water dikinase]-phosphate phosphotransferase / [pyruvate, water dikinase] kinase [EC:2.7.4.28 2.7.11.33]
CP004065.1_1471	K09768	74.23	190.8	6.8e-57	uncharacterized protein
CP004065.1_1472	K02316	140.00	540.0	2e-162	DNA primase [EC:2.7.7.101]
CP004065.1_1473	K03086	466.80	578.1	5e-174	RNA polymerase primary sigma factor

CP004065.1_1473	K03093	118.63	128.5	8e-38	RNA polymerase sigma-I factor
CP004065.1_1474	K13300	122.47	149.4	2e-44	cytochrome c550
CP004065.1_1475	K06967	59.70	297.5	3e-89	tRNA (adenine22-N1)-methyltransferase [EC:2.1.1.217]
CP004065.1_1476	K22391	167.17	190.8	7.3e-57	GTP cyclohydrolase I [EC:3.5.4.16]
CP004065.1_1477	K03527	70.27	347.7	2.6e-104	4-hydroxy-3-methylbut-2-en-1-yl diphosphate reductase [EC:1.17.7.4]
CP004065.1_1479	K18692	461.20	641.8	1.1e-193	ATP-dependent RNA helicase CshB [EC:5.6.2.7]
CP004065.1_1480	K01151	120.17	379.0	5.9e-114	deoxyribonuclease IV [EC:3.1.21.2]
CP004065.1_1483	K02076	141.93	219.6	4.5e-66	Fur family transcriptional regulator, zinc uptake regulator
CP004065.1_1484	K05967	108.73	231.4	1.7e-69	uncharacterized protein
CP004065.1_1486	K03526	92.70	567.9	4.9e-171	(E)-4-hydroxy-3-methylbut-2-enyl-diphosphate synthase [EC:1.17.7.1 1.17.7.3]
CP004065.1_1489	K09793	59.33	154.7	5.2e-46	uncharacterized protein
CP004065.1_1490	K04564	41.50	322.9	3.2e-97	superoxide dismutase, Fe-Mn family [EC:1.15.1.1]
CP004065.1_1491	K08222	125.80	539.4	1.1e-162	MFS transporter, YQGE family, putative transporter
CP004065.1_1492	K21465	815.63	964.4	7.5e-291	penicillin-binding protein A
CP004065.1_1493	K02040	56.17	258.9	2.4e-77	phosphate transport system substrate-binding protein
CP004065.1_1494	K02037	256.00	330.1	6e-99	phosphate transport system permease protein
CP004065.1_1495	K02038	250.80	271.2	2.7e-81	phosphate transport system permease protein
CP004065.1_1496	K02036	260.70	441.4	3.8e-133	phosphate transport system ATP-binding protein [EC:7.3.2.1]
CP004065.1_1497	K02036	260.70	441.3	3.9e-133	phosphate transport system ATP-binding protein [EC:7.3.2.1]
CP004065.1_1500	K02913	23.23	52.1	1.3e-14	large subunit ribosomal protein L33
CP004065.1_1501	K01934	93.97	182.3	3.1e-54	5-formyltetrahydrofolate cyclo-ligase [EC:6.3.3.2]
CP004065.1_1503	K03315	171.93	543.2	1.5e-163	Na+:H+ antiporter, NhaC family
CP004065.1_1504	K25026	274.23	343.4	4.4e-103	glucokinase [EC:2.7.1.2]
CP004065.1_1505	K01308	202.97	392.6	8.1e-118	g-D-glutamyl-meso-diaminopimelate peptidase [EC:3.4.19.11]
CP004065.1_1509	K01069	139.83	177.5	9e-53	hydroxyacylglutathione hydrolase [EC:3.1.2.6]
CP004065.1_1511	K16509	84.43	156.9	1.3e-46	regulatory protein spx
CP004065.1_1512	K17763	101.07	201.3	6.9e-60	rsbT co-antagonist protein RsbR
CP004065.1_1513	K03699	305.47	482.6	3.4e-145	magnesium and cobalt exporter, CNNM family
CP004065.1_1515	K02243	327.63	430.5	7.8e-130	competence protein ComGA
CP004065.1_1516	K02244	223.90	351.8	8.2e-106	competence protein ComGB
CP004065.1_1517	K02245	83.00	133.4	1.3e-39	competence protein ComGC
CP004065.1_1518	K02246	65.00	122.8	3.8e-36	competence protein ComGD
CP004065.1_1519	K02247	48.53	80.4	2.8e-23	competence protein ComGE
CP004065.1_1520	K02248	53.53	71.1	2.4e-20	competence protein ComGF
CP004065.1_1521	K02249	43.97	118.6	4.4e-35	competence protein ComGG
CP004065.1_1524	K19433	258.40	359.8	8.3e-109	TasA anchoring/assembly protein
CP004065.1_1525	K13280	61.43	123.0	3.6e-36	signal peptidase I [EC:3.4.21.89]
CP004065.1_1526	K06336	51.03	189.5	2.9e-56	spore coat-associated protein N
CP004065.1_1527	K19449	108.97	158.5	1.7e-47	XRE family transcriptional regulator, master regulator for biofilm formation
CP004065.1_1528	K06372	48.40	74.2	1.1e-21	antagonist of SinR
CP004065.1_1530	K03580	286.07	320.4	6.7e-96	ATP-dependent helicase HepA [EC:5.6.2.-]
CP004065.1_1531	K00605	295.73	525.2	3.8e-158	glycine cleavage system T protein (aminomethyltransferase) [EC:2.1.2.10]
CP004065.1_1532	K00282	413.50	692.7	4.4e-209	glycine cleavage system P protein (glycine dehydrogenase) subunit 1 [EC:1.4.4.2]
CP004065.1_1533	K00283	537.67	883.0	3e-266	glycine cleavage system P protein (glycine dehydrogenase) subunit 2 [EC:1.4.4.2]
CP004065.1_1534	K03972	95.13	105.5	6e-31	phage shock protein E
CP004065.1_1535	K23734	217.60	391.0	1.1e-117	lipoyl(octanoyl) transferase [EC:2.3.1.181]
CP004065.1_1536	K11924	157.13	224.0	4.8e-67	DtxR family transcriptional regulator, manganese transport regulator
CP004065.1_1537	K07001	95.57	161.8	5.1e-48	NTE family protein
CP004065.1_1541	K01262	268.13	293.4	9.4e-88	Xaa-Pro aminopeptidase [EC:3.4.11.9]
CP004065.1_1542	K02356	149.23	296.1	7e-89	elongation factor P
CP004065.1_1544	K06390	237.00	437.4	8.7e-132	stage III sporulation protein AA
CP004065.1_1545	K06391	68.77	213.9	3.4e-64	stage III sporulation protein AB
CP004065.1_1546	K06392	56.47	102.3	2.4e-30	stage III sporulation protein AC
CP004065.1_1547	K06393	50.83	142.9	1e-42	stage III sporulation protein AD
CP004065.1_1548	K06394	111.20	538.2	2.5e-162	stage III sporulation protein AE
CP004065.1_1549	K06395	34.30	187.6	6.8e-56	stage III sporulation protein AF

CP004065.1_1550	K06396	67.37	235.7	9.8e-71	stage III sporulation protein AG
CP004065.1_1551	K06397	56.83	183.1	1e-54	stage III sporulation protein AH
CP004065.1_1552	K02160	93.20	179.0	2.5e-53	acetyl-CoA carboxylase biotin carboxyl carrier protein
CP004065.1_1553	K01961	729.57	813.0	2.6e-245	acetyl-CoA carboxylase, biotin carboxylase subunit [EC:6.4.1.2 6.3.4.14]
CP004065.1_1555	K03625	88.60	175.7	3.2e-52	transcription antitermination protein NusB
CP004065.1_1556	K01491	414.73	485.2	3.7e-146	methylenetetrahydrofolate dehydrogenase (NADP+) / methenyltetrahydrofolate cyclohydrolase [EC:1.5.1.5 3.5.4.9]
CP004065.1_1557	K03601	56.10	542.3	3.7e-163	exodeoxyribonuclease VII large subunit [EC:3.1.11.6]
CP004065.1_1558	K03602	21.00	82.9	4.5e-24	exodeoxyribonuclease VII small subunit [EC:3.1.11.6]
CP004065.1_1559	K13789	367.20	372.7	4.6e-112	geranylgeranyl diphosphate synthase, type II [EC:2.5.1.1 2.5.1.10 2.5.1.29]
CP004065.1_1560	K01662	325.00	877.4	1.6e-264	1-deoxy-D-xylulose-5-phosphate synthase [EC:2.2.1.7]
CP004065.1_1561	K06442	67.40	388.8	6.9e-117	23S rRNA (cytidine1920-2'-O)/16S rRNA (cytidine1409-2'-O)-methyltransferase [EC:2.1.1.226 2.1.1.227]
CP004065.1_1562	K03402	48.50	197.5	5e-59	transcriptional regulator of arginine metabolism
CP004065.1_1563	K03631	130.47	689.1	1.4e-207	DNA repair protein RecN (Recombination protein N)
CP004065.1_1564	K06399	223.57	582.2	2e-175	stage IV sporulation protein B [EC:3.4.21.116]
CP004065.1_1565	K07699	109.57	423.6	5.9e-128	two-component system, response regulator, stage 0 sporulation protein A
CP004065.1_1566	K01775	106.13	420.6	1.5e-126	alanine racemase [EC:5.1.1.1]
CP004065.1_1574	K01448	29.13	235.8	1.5e-70	N-acetylmuramoyl-L-alanine amidase [EC:3.5.1.28]
CP004065.1_1575	K01126	152.53	224.4	4.1e-67	glycerophosphoryl diester phosphodiesterase [EC:3.1.4.46]
CP004065.1_1576	K00626	488.13	495.0	6.7e-149	acetyl-CoA C-acetyltransferase [EC:2.3.1.9]
CP004065.1_1577	K00074	404.50	470.9	1.2e-141	3-hydroxybutyryl-CoA dehydrogenase [EC:1.1.1.157]
CP004065.1_1578	K18244	584.47	589.6	6.7e-178	acyl-CoA dehydrogenase [EC:1.3.99.-]
CP004065.1_1578	K00248	523.17	557.0	8.9e-168	butyryl-CoA dehydrogenase [EC:1.3.8.1]
CP004065.1_1578	K00249	382.07	425.2	9.7e-128	acyl-CoA dehydrogenase [EC:1.3.8.7]
CP004065.1_1580	K01720	231.63	667.9	2.9e-201	2-methylcitrate dehydratase [EC:4.2.1.79]
CP004065.1_1581	K03417	365.37	400.9	5.8e-121	methylisocitrate lyase [EC:4.1.3.30]
CP004065.1_1583	K06714	491.73	545.6	2.4e-164	arginine utilization regulatory protein
CP004065.1_1585	K00263	475.53	575.1	2.6e-173	leucine dehydrogenase [EC:1.4.1.9]
CP004065.1_1586	K00929	187.47	590.6	2.5e-178	butyrate kinase [EC:2.7.2.7]
CP004065.1_1587	K00382	465.60	619.4	1.2e-186	dihydrolipoyl dehydrogenase [EC:1.8.1.4]
CP004065.1_1588	K00166	419.63	434.5	1.8e-130	2-oxoisovalerate dehydrogenase E1 component subunit alpha [EC:1.2.4.4]
CP004065.1_1589	K00167	490.37	506.1	3.5e-152	2-oxoisovalerate dehydrogenase E1 component subunit beta [EC:1.2.4.4]
CP004065.1_1590	K09699	429.73	461.6	5.8e-139	2-oxoisovalerate dehydrogenase E2 component (dihydrolipoyl transacylase) [EC:2.3.1.168]
CP004065.1_1591	K07029	147.20	239.6	1.1e-71	diacylglycerol kinase (ATP) [EC:2.7.1.107]
CP004065.1_1592	K26958	151.57	229.7	2.5e-69	bacilliredoxin
CP004065.1_1593	K02030	76.77	181.5	6.2e-54	polar amino acid transport system substrate-binding protein
CP004065.1_1594	K02029	214.53	261.5	3.2e-78	polar amino acid transport system permease protein
CP004065.1_1595	K02028	386.70	455.3	5.7e-137	polar amino acid transport system ATP-binding protein [EC:7.4.2.1]
CP004065.1_1595	K23060	426.27	440.3	1.1e-132	arginine/lysine/histidine transport system ATP-binding protein [EC:7.4.2.1]
CP004065.1_1599	K05606	80.33	166.0	1.7e-49	methylmalonyl-CoA/ethylmalonyl-CoA epimerase [EC:5.1.99.1]
CP004065.1_1601	K01258	167.60	414.7	1.2e-124	tripeptide aminopeptidase [EC:3.4.11.4]
CP004065.1_1602	K03217	121.07	217.1	7.4e-65	YidC/Oxa1 family membrane protein insertase
CP004065.1_1604	K02346	368.87	454.7	7.5e-137	DNA polymerase IV [EC:2.7.7.7]
CP004065.1_1605	K00033	157.37	828.1	1.3e-249	6-phosphogluconate dehydrogenase [EC:1.1.1.44 1.1.1.343]
CP004065.1_1606	K00036	396.33	642.3	2.3e-193	glucose-6-phosphate 1-dehydrogenase [EC:1.1.1.49 1.1.1.363]
CP004065.1_1607	K00784	70.63	295.8	1.2e-88	ribonuclease Z [EC:3.1.26.11]
CP004065.1_1612	K02601	101.20	104.8	1.1e-30	transcription termination/antitermination protein NusG
CP004065.1_1613	K15329	513.20	1214.1	0	trans-AT polyketide synthase, acyltransferase and oxidoreductase domains
CP004065.1_1613	K00645	247.17	326.0	1.2e-97	[acyl-carrier-protein] S-malonyltransferase [EC:2.3.1.39]
CP004065.1_1615	K02078	39.80	42.2	1.8e-11	acyl carrier protein
CP004065.1_1617	K00059	269.80	276.2	9.6e-83	3-oxoacyl-[acyl-carrier protein] reductase [EC:1.1.1.100]
CP004065.1_1618	K25849	7894.80	8855.8	0	difficidin synthase DfnD
CP004065.1_1619	K25850	5010.60	5037.2	0	difficidin synthase DfnE
CP004065.1_1621	K25852	7367.40	11888.0	0	difficidin synthase DfnG
CP004065.1_1622	K25853	4023.33	5251.7	0	difficidin synthase DfnH
CP004065.1_1623	K25854	2707.23	4915.8	0	difficidin synthase DfnI
CP004065.1_1624	K00344	279.00	291.9	2.1e-87	NADPH:quinone reductase [EC:1.6.5.5]

CP004065.1_1626	K15311	506.87	716.2	2.3e-216	3-carboxymethyl-3-hydroxy-acyl-[acp] synthase [EC:2.3.3.22]
CP004065.1_1628	K00286	164.07	322.1	1.8e-96	pyrroline-5-carboxylate reductase [EC:1.5.1.2]
CP004065.1_1630	K07124	220.10	229.8	1.1e-68	uncharacterized protein
CP004065.1_1631	K01753	290.13	669.0	6.7e-202	D-serine dehydratase [EC:4.3.1.18]
CP004065.1_1632	K00867	171.90	466.5	1.5e-140	type I pantothenate kinase [EC:2.7.1.33]
CP004065.1_1638	K02346	368.87	394.9	1e-118	DNA polymerase IV [EC:2.7.7.7]
CP004065.1_1644	K06889	113.67	144.3	1e-42	uncharacterized protein
CP004065.1_1648	K01515	150.10	210.5	1e-62	ADP-ribose diphosphatase [EC:3.6.1.13 3.6.1.-]
CP004065.1_1651	K01424	50.80	316.2	1.1e-94	L-asparaginase [EC:3.5.1.1]
CP004065.1_1652	K01744	667.80	756.5	4.5e-228	aspartate ammonia-lyase [EC:4.3.1.1]
CP004065.1_1653	K03315	171.93	560.3	9.8e-169	Na+:H+ antiporter, NhaC family
CP004065.1_1658	K06384	34.00	135.0	7.3e-40	stage II sporulation protein M
CP004065.1_1659	K03711	142.10	164.3	1e-48	Fur family transcriptional regulator, ferric uptake regulator
CP004065.1_1661	K04763	301.10	348.1	2.2e-104	integrase/recombinase XerD
CP004065.1_1662	K01839	156.37	660.9	3.4e-199	phosphopentomutase [EC:5.4.2.7]
CP004065.1_1663	K03783	165.87	449.3	3.8e-135	purine-nucleoside phosphorylase [EC:2.4.2.1]
CP004065.1_1664	K07258	223.10	383.8	2.8e-115	serine-type D-Ala-D-Ala carboxypeptidase (penicillin-binding protein 5/6) [EC:3.4.16.4]
CP004065.1_1665	K06378	115.47	141.4	5.7e-42	stage II sporulation protein AA (anti-sigma F factor antagonist)
CP004065.1_1665	K04749	63.93	91.4	1.7e-26	anti-sigma B factor antagonist
CP004065.1_1666	K06379	153.40	229.7	4.5e-69	stage II sporulation protein AB (anti-sigma F factor) [EC:2.7.11.1]
CP004065.1_1666	K04757	53.50	89.7	4.9e-26	serine/threonine-protein kinase RsbW [EC:2.7.11.1]
CP004065.1_1667	K03091	189.70	253.4	6.4e-76	RNA polymerase sigma-E/F/G factor
CP004065.1_1668	K06403	121.77	289.6	2.7e-87	stage V sporulation protein AA
CP004065.1_1669	K06404	101.63	153.2	1.1e-45	stage V sporulation protein AB
CP004065.1_1670	K06405	98.60	228.1	8.3e-69	stage V sporulation protein AC
CP004065.1_1671	K06406	180.47	519.6	5.1e-157	stage V sporulation protein AD
CP004065.1_1672	K06407	58.07	138.8	1.7e-41	stage V sporulation protein AE
CP004065.1_1673	K06407	58.07	142.7	1.1e-42	stage V sporulation protein AE
CP004065.1_1674	K06408	458.97	746.5	3.2e-225	stage V sporulation protein AF
CP004065.1_1675	K01586	276.10	541.4	8.9e-163	diaminopimelate decarboxylase [EC:4.1.1.20]
CP004065.1_1679	K03100	114.77	189.4	1.9e-56	signal peptidase I [EC:3.4.21.89]
CP004065.1_1681	K11752	189.40	503.7	2.4e-151	diaminohydroxyphosphoribosylaminopyrimidine deaminase / 5-amino-6-(5-phosphoribosylamino)uracil reductase [EC:3.5.4.26 1.1.1.193]
CP004065.1_1682	K00793	67.77	288.5	1.1e-86	riboflavin synthase [EC:2.5.1.9]
CP004065.1_1683	K14652	367.03	685.6	1.7e-206	3,4-dihydroxy 2-butanone 4-phosphate synthase / GTP cyclohydrolase II [EC:4.1.99.12 3.5.4.25]
CP004065.1_1683	K01497	257.40	323.9	3.4e-97	GTP cyclohydrolase II [EC:3.5.4.25]
CP004065.1_1684	K00794	39.60	258.7	1.6e-77	6,7-dimethyl-8-ribityllumazine synthase [EC:2.5.1.78]
CP004065.1_1685	K02859	82.13	167.2	3.1e-50	riboflavin biosynthesis RibT protein
CP004065.1_1686	K09763	44.57	110.2	3.1e-32	uncharacterized protein
CP004065.1_1688	K05896	40.37	279.0	1.1e-83	segregation and condensation protein A
CP004065.1_1689	K06024	48.53	210.4	8e-63	segregation and condensation protein B
CP004065.1_1691	K07258	223.10	345.0	1.7e-103	serine-type D-Ala-D-Ala carboxypeptidase (penicillin-binding protein 5/6) [EC:3.4.16.4]
CP004065.1_1692	K06373	144.10	314.6	1.1e-94	spore maturation protein A
CP004065.1_1693	K06374	151.90	264.8	7.1e-80	spore maturation protein B
CP004065.1_1694	K06178	253.10	334.9	1.8e-100	23S rRNA pseudouridine2605 synthase [EC:5.4.99.22]
CP004065.1_1695	K02199	121.53	137.4	1.2e-40	cytochrome c biogenesis protein CcmG, thiol:disulfide interchange protein DsbE
CP004065.1_1696	K07399	102.77	304.4	3.3e-91	cytochrome c biogenesis protein
CP004065.1_1698	K07775	311.73	371.3	4.8e-112	two-component system, OmpR family, response regulator ResD
CP004065.1_1698	K02483	242.00	274.4	4.3e-82	two-component system, OmpR family, response regulator
CP004065.1_1699	K07651	523.70	784.7	1.5e-236	two-component system, OmpR family, sensor histidine kinase ResE [EC:2.7.13.3]
CP004065.1_1699	K07636	310.93	348.0	1.8e-104	two-component system, OmpR family, phosphate regulon sensor histidine kinase PhoR [EC:2.7.13.3]
CP004065.1_1700	K03088	96.50	140.8	1.2e-41	RNA polymerase sigma-70 factor, ECF subfamily
CP004065.1_1702	K00058	329.93	497.6	7.8e-150	D-3-phosphoglycerate dehydrogenase / 2-oxoglutarate reductase [EC:1.1.1.95 1.1.1.399]
CP004065.1_1703	K23675	54.77	209.6	1.8e-62	riboflavin transporter
CP004065.1_1704	K05337	42.27	71.6	1.4e-20	ferredoxin
CP004065.1_1707	K07052	31.30	62.7	6.1e-18	CAAX protease family protein
CP004065.1_1710	K07098	96.03	194.3	7.1e-58	uncharacterized protein

CP004065.1_1711	K16511	72.03	236.8	6.8e-71	adapter protein MecA 1/2
CP004065.1_1712	K00260	633.70	720.6	1.1e-217	glutamate dehydrogenase [EC:1.4.1.2]
CP004065.1_1713	K26960	268.90	491.3	2e-148	bacillithiol disulfide reductase [EC:1.8.1.-]
CP004065.1_1714	K24131	48.00	150.7	1.4e-44	protease PrsW [EC:3.4.-.-]
CP004065.1_1715	K27245	246.57	387.7	1.5e-116	peptidoglycan lytic transglycosylase [EC:4.2.2.29]
CP004065.1_1715	K01449	48.60	185.5	2.6e-55	cell wall hydrolase
CP004065.1_1716	K06313	156.80	563.5	7.9e-170	spore germination protein
CP004065.1_1719	K00945	42.83	312.1	1.3e-93	CMP/dCMP kinase [EC:2.7.4.25]
CP004065.1_1720	K02945	147.87	437.9	1.3e-131	small subunit ribosomal protein S1
CP004065.1_1724	K03977	233.47	636.7	6.4e-192	GTPase
CP004065.1_1725	K00057	279.33	526.6	1.7e-158	glycerol-3-phosphate dehydrogenase (NAD(P)+) [EC:1.1.1.94]
CP004065.1_1728	K06398	484.37	835.9	2.5e-252	stage IV sporulation protein A
CP004065.1_1729	K03530	115.33	159.5	2.9e-47	DNA-binding protein HU-beta
CP004065.1_1730	K01495	58.13	321.1	1.5e-96	GTP cyclohydrolase IA [EC:3.5.4.16]
CP004065.1_1731	K06285	79.87	136.3	4.2e-41	transcription attenuation protein (tryptophan RNA-binding attenuator protein)
CP004065.1_1732	K00805	56.80	249.7	8.1e-75	heptaprenyl diphosphate synthase component 1 [EC:2.5.1.30]
CP004065.1_1733	K03183	266.63	327.2	2.8e-98	demethylmenaquinone methyltransferase / 2-methoxy-6-polyprenyl-1,4-benzoquinol methylase [EC:2.1.1.163 2.1.1.201]
CP004065.1_1734	K24873	367.43	499.4	6.7e-151	heptaprenyl diphosphate synthase component 2 [EC:2.5.1.30]
CP004065.1_1735	K00940	149.93	263.0	8.5e-79	nucleoside-diphosphate kinase [EC:2.7.4.6]
CP004065.1_1736	K00575	241.67	297.1	6.9e-89	chemotaxis protein methyltransferase CheR [EC:2.1.1.80]
CP004065.1_1737	K01736	306.20	494.0	1.9e-148	chorismate synthase [EC:4.2.3.5]
CP004065.1_1738	K01735	305.30	426.5	3.4e-128	3-dehydroquinate synthase [EC:4.2.3.4]
CP004065.1_1739	K06208	80.70	194.3	2.4e-58	chorismate mutase [EC:5.4.99.5]
CP004065.1_1740	K01657	465.93	605.1	3.4e-182	anthranilate synthase component I [EC:4.1.3.27]
CP004065.1_1741	K00766	109.93	419.6	3.2e-126	anthranilate phosphoribosyltransferase [EC:2.4.2.18]
CP004065.1_1744	K01696	625.47	781.6	3.7e-235	tryptophan synthase beta chain [EC:4.2.1.20]
CP004065.1_1745	K01695	85.97	297.6	2.7e-89	tryptophan synthase alpha chain [EC:4.2.1.20]
CP004065.1_1746	K00817	293.33	410.5	2.8e-123	histidinol-phosphate aminotransferase [EC:2.6.1.9]
CP004065.1_1746	K04517	224.97	375.0	1.7e-112	prephenate dehydrogenase [EC:1.3.1.12]
CP004065.1_1747	K04517	224.97	329.4	1e-98	prephenate dehydrogenase [EC:1.3.1.12]
CP004065.1_1748	K00800	150.47	444.0	1.9e-133	3-phosphoshikimate 1-carboxyvinyltransferase [EC:2.5.1.19]
CP004065.1_1752	K03886	129.77	224.1	3.2e-67	quinol---cytochrome c reductase iron-sulfur subunit, bacillus type [EC:7.1.1.8]
CP004065.1_1753	K03887	277.47	339.0	2.5e-102	quinol---cytochrome c reductase cytochrome b subunit, bacillus type [EC:7.1.1.8]
CP004065.1_1754	K03888	183.03	384.5	8.4e-116	quinol---cytochrome c reductase cytochrome c subunit, bacillus type [EC:7.1.1.8]
CP004065.1_1757	K01569	93.33	616.6	4.2e-186	oxalate decarboxylase [EC:4.1.1.2]
CP004065.1_1760	K00215	135.50	265.2	2.2e-79	4-hydroxy-tetrahydrodipicolinate reductase [EC:1.17.1.8]
CP004065.1_1761	K01734	76.13	215.9	6.9e-65	methylglyoxal synthase [EC:4.2.3.3]
CP004065.1_1762	K01463	161.63	249.1	1.4e-74	N-acetylglucosamine malate deacetylase 1 [EC:3.5.1.-]
CP004065.1_1763	K00754	262.90	368.1	2e-110	L-malate glycosyltransferase [EC:2.4.1.-]
CP004065.1_1764	K00974	272.67	311.3	2.4e-93	tRNA nucleotidyltransferase (CCA-adding enzyme) [EC:2.7.7.72 3.1.3.- 3.1.4.-]
CP004065.1_1765	K03524	154.23	247.8	3.9e-74	BirA family transcriptional regulator, biotin operon repressor / biotin---[acetyl-CoA-carboxylase] ligase [EC:6.3.4.15]
CP004065.1_1766	K00606	82.33	410.0	1.9e-123	3-methyl-2-oxobutanoate hydroxymethyltransferase [EC:2.1.2.11]
CP004065.1_1767	K01918	67.70	375.1	8.6e-113	pantoate--beta-alanine ligase [EC:6.3.2.1]
CP004065.1_1768	K01579	52.57	230.2	4.5e-69	aspartate 1-decarboxylase [EC:4.1.1.11]
CP004065.1_1769	K03722	230.90	546.6	2.5e-164	ATP-dependent DNA helicase DinG [EC:5.6.2.3]
CP004065.1_1769	K02342	101.50	197.2	9.3e-59	DNA polymerase III subunit epsilon [EC:2.7.7.7]
CP004065.1_1769	K09951	46.77	52.9	9.6e-15	CRISPR-associated protein Cas2
CP004065.1_1772	K00812	468.17	558.3	2.7e-168	aspartate aminotransferase [EC:2.6.1.1]
CP004065.1_1773	K01893	373.80	540.4	9.7e-163	asparaginyl-tRNA synthetase [EC:6.1.1.22]
CP004065.1_1774	K02086	54.33	301.3	1.4e-90	DNA replication protein
CP004065.1_1775	K10773	133.83	278.9	1.9e-83	endonuclease III [EC:3.2.2.- 4.2.99.18]
CP004065.1_1777	K05366	639.07	731.5	3.3e-220	penicillin-binding protein 1A [EC:2.4.99.28 3.4.16.4]
CP004065.1_1778	K03700	53.70	272.6	1.1e-81	recombination protein U
CP004065.1_1780	K06430	37.37	65.3	1e-18	small acid-soluble spore protein M (minor)
CP004065.1_1786	K02777	194.60	200.6	5.8e-60	sugar PTS system EIIA component [EC:2.7.1.-]
CP004065.1_1787	K06877	273.53	932.2	6.6e-281	DEAD/DEAH box helicase domain-containing protein

CP004065.1_1788	K07502	85.30	378.3	1.3e-113	uncharacterized protein
CP004065.1_1790	K06327	40.20	94.7	1.5e-27	spore coat protein D
CP004065.1_1792	K04074	47.10	65.9	8.5e-19	cell division initiation protein
CP004065.1_1793	K07444	419.40	578.7	2.7e-174	putative N6-adenine-specific DNA methylase [EC:2.1.1.-]
CP004065.1_1797	K03722	230.90	407.9	2.1e-122	ATP-dependent DNA helicase DinG [EC:5.6.2.3]
CP004065.1_1798	K01299	201.97	665.4	1.4e-200	carboxypeptidase Taq [EC:3.4.17.19]
CP004065.1_1799	K03816	140.40	304.8	3.6e-92	xanthine phosphoribosyltransferase [EC:2.4.2.22]
CP004065.1_1800	K16169	599.97	680.2	2e-205	xanthine permease
CP004065.1_1806	K07443	96.07	145.6	3.6e-43	methylated-DNA-protein-cysteine methyltransferase related protein
CP004065.1_1807	K22479	120.87	259.1	1.1e-77	N-acetyltransferase
CP004065.1_1808	K16167	337.77	435.6	2.9e-131	alkylresorcinol/alkylpyrone synthase
CP004065.1_1809	K16168	117.50	244.7	3.1e-73	methyltransferase
CP004065.1_1813	K23786	338.90	430.4	6e-130	5'-3' exonuclease [EC:3.1.11.-]
CP004065.1_1814	K06429	37.57	57.1	3.4e-16	small acid-soluble spore protein L (minor)
CP004065.1_1816	K09125	29.70	201.0	6.6e-60	queuosine precursor transporter
CP004065.1_1821	K03704	80.73	115.1	6.9e-34	cold shock protein
CP004065.1_1822	K03429	296.23	385.7	9.8e-116	processive 1,2-diacylglycerol beta-glucosyltransferase [EC:2.4.1.315]
CP004065.1_1823	K00651	244.77	474.9	3.7e-143	homoserine O-succinyltransferase/O-acetyltransferase [EC:2.3.1.46 2.3.1.31]
CP004065.1_1824	K00432	54.07	252.5	9.3e-76	glutathione peroxidase [EC:1.11.1.9]
CP004065.1_1825	K06950	47.43	164.2	1.1e-48	uncharacterized protein
CP004065.1_1827	K01687	599.53	948.1	1e-285	dihydroxy-acid dehydratase [EC:4.2.1.9]
CP004065.1_1828	K26958	151.57	225.5	4.8e-68	bacilliredoxin
CP004065.1_1829	K15984	88.30	150.6	1.5e-44	16S rRNA (guanine1516-N2)-methyltransferase [EC:2.1.1.242]
CP004065.1_1831	K00560	113.10	488.0	5.7e-147	thymidylate synthase [EC:2.1.1.45]
CP004065.1_1832	K00287	64.20	244.2	6e-73	dihydrofolate reductase [EC:1.5.1.3]
CP004065.1_1834	K11068	170.70	279.2	9.3e-84	hemolysin III
CP004065.1_1836	K01754	266.27	450.6	1.8e-135	threonine dehydratase [EC:4.3.1.19]
CP004065.1_1838	K07152	55.90	158.4	6.8e-47	protein SCO1
CP004065.1_1842	K07304	43.27	258.8	1.8e-77	peptide-methionine (S)-S-oxide reductase [EC:1.8.4.11]
CP004065.1_1843	K07305	92.37	204.4	3.8e-61	peptide-methionine (R)-S-oxide reductase [EC:1.8.4.12]
CP004065.1_1844	K15894	314.93	385.8	3.2e-116	UDP-N-acetylglucosamine 4,6-dehydratase/5-epimerase [EC:4.2.1.115 5.1.3.-]
CP004065.1_1845	K01083	115.73	463.3	2.7e-139	3-phytase [EC:3.1.3.8]
CP004065.1_1846	K06320	85.60	203.5	1.1e-60	spore maturation protein CgeB
CP004065.1_1847	K06319	70.03	183.3	3.6e-55	spore maturation protein CgeA
CP004065.1_1848	K06321	137.03	160.1	2.5e-48	spore maturation protein CgeC
CP004065.1_1849	K06322	347.60	695.7	4.2e-210	spore maturation protein CgeD
CP004065.1_1850	K06323	155.93	387.9	5.1e-117	spore maturation protein CgeE
CP004065.1_1854	K01034	300.93	324.7	5e-98	acetate CoA/acetoacetate CoA-transferase alpha subunit [EC:2.8.3.8 2.8.3.9]
CP004065.1_1856	K01438	295.90	350.5	5.1e-105	acetylornithine deacetylase [EC:3.5.1.16]
CP004065.1_1857	K21935	123.73	332.4	6.2e-100	beta-lysine N6-acetyltransferase [EC:2.3.1.264]
CP004065.1_1858	K01843	367.80	608.8	1.6e-183	lysine 2,3-aminomutase [EC:5.4.3.2]
CP004065.1_1865	K19302	106.60	143.1	2.7e-42	undecaprenyl-diphosphatase [EC:3.6.1.27]
CP004065.1_1868	K03784	226.90	404.0	6.8e-122	purine-nucleoside phosphorylase [EC:2.4.2.1]
CP004065.1_1869	K07260	64.77	274.3	4.3e-82	zinc D-Ala-D-Ala carboxypeptidase [EC:3.4.17.14]
CP004065.1_1872	K03797	127.93	409.7	5.4e-123	carboxyl-terminal processing protease [EC:3.4.21.102]
CP004065.1_1873	K03489	211.60	337.9	4.3e-102	GntR family transcriptional regulator, transcriptional regulator of bglA
CP004065.1_1874	K01223	479.40	763.6	2.2e-230	6-phospho-beta-glucosidase [EC:3.2.1.86]
CP004065.1_1875	K01201	204.70	394.3	2.1e-118	glucosylceramidase [EC:3.2.1.45]
CP004065.1_1878	K15975	357.47	423.4	2.3e-127	glyoxalase family protein
CP004065.1_1879	K06999	93.57	159.7	2.2e-47	phospholipase/carboxylesterase
CP004065.1_1880	K15976	141.90	173.3	8.6e-52	putative NAD(P)H nitroreductase [EC:1.-.-.-]
CP004065.1_1886	K05786	96.17	390.6	1.4e-117	chloramphenicol-sensitive protein RarD
CP004065.1_1889	K22135	192.50	294.6	2e-88	N-acetylglucosamine malate deacetylase 2 [EC:3.5.1.-]
CP004065.1_1890	K17763	101.07	187.3	1.2e-55	rsbT co-antagonist protein RsbR
CP004065.1_1891	K26939	355.77	447.1	1.5e-134	MATE family, multidrug efflux pump
CP004065.1_1893	K18672	81.73	187.4	1e-55	diadenylate cyclase [EC:2.7.7.85]

CP004065.1_1895	K19220	406.67	477.1	2.1e-143	peptidoglycan DL-endopeptidase CwlS [EC:3.4.-.-]
CP004065.1_1895	K19223	333.63	382.3	1.1e-114	peptidoglycan DL-endopeptidase LytF [EC:3.4.-.-]
CP004065.1_1895	K19224	198.37	268.5	2.3e-80	peptidoglycan DL-endopeptidase LytE [EC:3.4.-.-]
CP004065.1_1896	K04565	84.73	113.6	2.3e-33	superoxide dismutase, Cu-Zn family [EC:1.15.1.1]
CP004065.1_1897	K04748	193.03	293.0	8.1e-88	nitric oxide reductase NorQ protein
CP004065.1_1900	K00658	444.90	557.4	5.9e-168	2-oxoglutarate dehydrogenase E2 component (dihydrolipoamide succinyltransferase) [EC:2.3.1.61]
CP004065.1_1902	K03453	223.63	375.7	1e-112	bile acid:Na+ symporter, BASS family
CP004065.1_1903	K03308	387.23	532.8	2.1e-160	neurotransmitter:Na+ symporter, NSS family
CP004065.1_1904	K04564	41.50	291.4	1.3e-87	superoxide dismutase, Fe-Mn family [EC:1.15.1.1]
CP004065.1_1905	K18115	624.37	880.2	1.4e-265	sporulenol synthase [EC:4.2.1.137]
CP004065.1_1906	K00146	695.17	709.9	2.7e-214	phenylacetaldehyde dehydrogenase [EC:1.2.1.39]
CP004065.1_1906	K00130	687.90	692.8	8.6e-209	betaine-aldehyde dehydrogenase [EC:1.2.1.8]
CP004065.1_1906	K00128	593.30	685.6	1.1e-206	aldehyde dehydrogenase (NAD+) [EC:1.2.1.3]
CP004065.1_1915	K06204	35.73	89.5	8.4e-26	RNA polymerase-binding transcription factor
CP004065.1_1917	K01118	93.70	216.3	1.4e-64	FMN-dependent NADH-azoreductase [EC:1.7.1.17]
CP004065.1_1918	K03654	618.33	720.2	8e-217	ATP-dependent DNA helicase RecQ [EC:5.6.2.4]
CP004065.1_1921	K01297	137.13	246.6	8.6e-74	muramoyltetrapeptide carboxypeptidase [EC:3.4.17.13]
CP004065.1_1924	K08976	36.27	233.7	4.7e-70	putative membrane protein
CP004065.1_1926	K22043	126.53	162.0	1.7e-48	ArsR family transcriptional regulator, zinc-responsive transcriptional repressor
CP004065.1_1926	K03892	89.73	93.9	2.7e-27	ArsR family transcriptional regulator, arsenate/arsenite/antimonite-responsive transcriptional repressor
CP004065.1_1931	K06878	128.33	148.0	5.6e-44	tRNA-binding protein
CP004065.1_1945	K00286	164.07	286.2	1.4e-85	pyrroline-5-carboxylate reductase [EC:1.5.1.2]
CP004065.1_1946	K00931	289.63	445.7	7.1e-134	glutamate 5-kinase [EC:2.7.2.11]
CP004065.1_1947	K09681	263.80	483.0	6.5e-146	LysR family transcriptional regulator, transcription activator of glutamate synthase operon
CP004065.1_1948	K00265	1712.27	2287.4	0	glutamate synthase (NADPH) large chain [EC:1.4.1.13]
CP004065.1_1949	K00266	509.03	603.0	1.4e-181	glutamate synthase (NADPH) small chain [EC:1.4.1.13]
CP004065.1_1951	K13955	330.60	480.5	4.3e-145	zinc-binding alcohol dehydrogenase/oxidoreductase
CP004065.1_1952	K21959	245.10	413.0	1.2e-124	LysR family transcriptional regulator, cell division regulator
CP004065.1_1955	K08164	409.17	559.2	8.2e-169	MFS transporter, DHA1 family, putative efflux transporter
CP004065.1_1956	K00681	540.00	689.3	1.4e-207	gamma-glutamyltranspeptidase / glutathione hydrolase [EC:2.3.2.2 3.4.19.13]
CP004065.1_1959	K23598	84.67	182.0	2.5e-54	iseA protein
CP004065.1_1960	K26937	267.43	393.2	3.1e-118	MATE family, multidrug efflux pump
CP004065.1_1961	K01785	42.83	137.9	1.1e-40	aldose 1-epimerase [EC:5.1.3.3]
CP004065.1_1962	K07259	126.90	405.9	6.4e-122	serine-type D-Ala-D-Ala carboxypeptidase/endopeptidase (penicillin-binding protein 4) [EC:3.4.16.4 3.4.21.-]
CP004065.1_1963	K15664	4458.07	5278.6	0	plipastatin/fengycin lipopeptide synthetase A
CP004065.1_1964	K15665	4492.57	5288.2	0	plipastatin/fengycin lipopeptide synthetase B
CP004065.1_1965	K15666	4455.47	4905.3	0	plipastatin/fengycin lipopeptide synthetase C
CP004065.1_1965	K16118	1359.93	1649.7	0	pristinamycin I synthase 3 and 4
CP004065.1_1966	K15667	4063.50	7165.5	0	plipastatin/fengycin lipopeptide synthetase D
CP004065.1_1967	K15668	2076.13	2157.1	0	plipastatin/fengycin lipopeptide synthetase E
CP004065.1_1970	K00248	523.17	544.3	6e-164	butyryl-CoA dehydrogenase [EC:1.3.8.1]
CP004065.1_1970	K11410	517.13	530.1	6.7e-160	short-chain 2-methylacyl-CoA dehydrogenase [EC:1.3.8.5]
CP004065.1_1970	K00249	382.07	428.2	1.2e-128	acyl-CoA dehydrogenase [EC:1.3.8.7]
CP004065.1_1971	K00666	594.33	692.4	1.7e-208	fatty-acyl-CoA synthase [EC:6.2.1.-]
CP004065.1_1971	K01897	430.97	431.7	1.1e-129	long-chain acyl-CoA synthetase [EC:6.2.1.3]
CP004065.1_1974	K01640	135.00	431.3	1e-129	hydroxymethylglutaryl-CoA lyase [EC:4.1.3.4]
CP004065.1_1977	K03975	59.97	104.0	1.7e-30	membrane-associated protein
CP004065.1_1978	K00963	76.77	140.8	1.4e-41	UTP--glucose-1-phosphate uridylyltransferase [EC:2.7.7.9]
CP004065.1_1980	K01906	151.03	302.5	4.4e-91	6-carboxyhexanoate--CoA ligase [EC:6.2.1.14]
CP004065.1_1981	K19563	783.60	943.9	2.3e-285	lysine---8-amino-7-oxononanoate aminotransferase [EC:2.6.1.105]
CP004065.1_1981	K00833	407.37	534.6	5.3e-161	adenosylmethionine---8-amino-7-oxononanoate aminotransferase [EC:2.6.1.62]
CP004065.1_1982	K00652	449.27	515.1	5.5e-155	8-amino-7-oxononanoate synthase [EC:2.3.1.47]
CP004065.1_1983	K01935	123.07	238.2	3.7e-71	dethiobiotin synthetase [EC:6.3.3.3]
CP004065.1_1984	K01012	173.23	405.8	5.8e-122	biotin synthase [EC:2.8.1.6]
CP004065.1_1985	K16593	478.83	673.7	2.1e-203	pimeloyl-[acyl-carrier protein] synthase [EC:1.14.14.46]
CP004065.1_1987	K01028	407.90	424.1	4.9e-128	3-oxoacid CoA-transferase subunit A [EC:2.8.3.5]

CP004065.1_1988	K01029	368.27	388.3	3.1e-117	3-oxoacid CoA-transferase subunit B [EC:2.8.3.5]
CP004065.1_1989	K00019	183.03	201.2	6.4e-60	3-hydroxybutyrate dehydrogenase [EC:1.1.1.30]
CP004065.1_1990	K00645	247.17	346.9	5.8e-104	[acyl-carrier-protein] S-malonyltransferase [EC:2.3.1.39]
CP004065.1_1991	K15661	3755.90	7988.3	0	iturin family lipopeptide synthetase A
CP004065.1_1991	K01845	312.23	414.7	1.6e-124	glutamate-1-semialdehyde 2,1-aminomutase [EC:5.4.3.8]
CP004065.1_1992	K15662	10498.77	11528.6	0	iturin family lipopeptide synthetase B
CP004065.1_1992	K16118	1359.93	1408.0	0	pristinamycin I synthase 3 and 4
CP004065.1_1993	K15663	4322.10	5593.1	0	iturin family lipopeptide synthetase C
CP004065.1_1993	K03367	499.93	507.2	9.3e-153	D-alanine--poly(phosphoribitol) ligase subunit 1 [EC:6.1.1.13]
CP004065.1_1994	K15921	357.33	738.7	1.4e-222	arabinoxylan arabinofuranohydrolase [EC:3.2.1.55]
CP004065.1_1995	K15924	253.57	705.8	1.5e-212	glucuronoarabinoxylan endo-1,4-beta-xylanase [EC:3.2.1.136]
CP004065.1_2006	K02525	324.67	383.9	1.5e-115	LacI family transcriptional regulator, kdg operon repressor
CP004065.1_2006	K02529	268.37	289.6	1.1e-86	LacI family transcriptional regulator, galactose operon repressor
CP004065.1_2007	K01686	137.10	163.9	1.1e-48	mannonate dehydratase [EC:4.2.1.8]
CP004065.1_2008	K01686	137.10	264.2	4.4e-79	mannonate dehydratase [EC:4.2.1.8]
CP004065.1_2009	K01686	137.10	138.2	6.9e-41	mannonate dehydratase [EC:4.2.1.8]
CP004065.1_2010	K01625	193.93	229.3	2e-68	2-dehydro-3-deoxyphosphogluconate aldolase / (4S)-4-hydroxy-2-oxoglutarate aldolase [EC:4.1.2.14 4.1.3.42]
CP004065.1_2012	K00874	245.93	307.3	6.4e-92	2-dehydro-3-deoxygluconokinase [EC:2.7.1.45]
CP004065.1_2013	K03310	262.87	582.5	1.9e-175	alanine or glycine:cation symporter, AGCS family
CP004065.1_2016	K02621	874.50	933.4	3.2e-281	topoisomerase IV subunit A [EC:5.6.2.2]
CP004065.1_2017	K02622	875.97	900.4	1.5e-271	topoisomerase IV subunit B [EC:5.6.2.2]
CP004065.1_2018	K06929	81.97	178.6	2.5e-53	uncharacterized protein
CP004065.1_2019	K08591	64.30	235.8	1.2e-70	acyl phosphate:glycerol-3-phosphate acyltransferase [EC:2.3.1.275]
CP004065.1_2022	K07107	72.43	105.4	1.2e-30	acyl-CoA thioester hydrolase [EC:3.1.2.-]
CP004065.1_2023	K06434	40.27	120.5	4.9e-36	small acid-soluble spore protein (thioredoxin-like protein)
CP004065.1_2024	K06431	48.43	57.3	2.5e-16	small acid-soluble spore protein N (minor)
CP004065.1_2025	K02199	121.53	133.5	1.8e-39	cytochrome c biogenesis protein CcmG, thiol:disulfide interchange protein DsbE
CP004065.1_2026	K27802	1411.80	1474.4	0	aconitate hydratase A / 2-methylisocitrate dehydratase [EC:4.2.1.3 4.2.1.99]
CP004065.1_2027	K06432	39.73	84.1	1.5e-24	small acid-soluble spore protein O (minor)
CP004065.1_2028	K06433	38.23	83.2	3e-24	small acid-soluble spore protein P (minor)
CP004065.1_2029	K06335	111.80	142.4	1.5e-42	spore coat protein M
CP004065.1_2033	K06196	133.63	234.5	4.7e-70	cytochrome c-type biogenesis protein
CP004065.1_2034	K06376	38.70	58.4	1.3e-16	stage 0 sporulation regulatory protein
CP004065.1_2035	K09976	58.87	116.1	1.5e-34	uncharacterized protein
CP004065.1_2037	K00615	214.20	592.5	3e-178	transketolase [EC:2.2.1.1]
CP004065.1_2041	K01356	128.77	278.0	2.2e-83	repressor LexA [EC:3.4.21.88]
CP004065.1_2047	K01520	47.27	77.8	1.9e-22	dUTP diphosphatase [EC:3.6.1.23]
CP004065.1_2051	K02435	35.93	71.8	1e-20	aspartyl-tRNA(Asn)/glutamyl-tRNA(Gln) amidotransferase subunit C [EC:6.3.5.6 6.3.5.7]
CP004065.1_2060	K13953	338.93	427.1	1.6e-128	alcohol dehydrogenase, propanol-preferring [EC:1.1.1.1]
CP004065.1_2068	K22579	503.63	838.9	1.4e-252	rifampicin phosphotransferase [EC:2.7.9.6]
CP004065.1_2069	K00854	306.67	366.0	9.6e-110	xylulokinase [EC:2.7.1.17]
CP004065.1_2071	K01805	102.00	714.3	2.8e-215	xylose isomerase [EC:5.3.1.5]
CP004065.1_2073	K01198	271.87	465.1	1.2e-139	xylan 1,4-beta-xylosidase [EC:3.2.1.37]
CP004065.1_2074	K03292	344.60	477.4	1e-143	glycoside/pentoside/hexuronide:cation symporter, GPH family
CP004065.1_2076	K01915	33.97	364.4	3e-109	glutamine synthetase [EC:6.3.1.2]
CP004065.1_2077	K03713	103.80	190.8	3.8e-57	MerR family transcriptional regulator, glutamine synthetase repressor
CP004065.1_2079	K03665	194.73	524.2	9.8e-158	GTPase
CP004065.1_2080	K06413	333.77	452.6	2.9e-136	stage V sporulation protein K
CP004065.1_2081	K01448	29.13	213.2	1.1e-63	N-acetylmuramoyl-L-alanine amidase [EC:3.5.1.28]
CP004065.1_2084	K00525	367.50	479.2	5.4e-144	ribonucleoside-diphosphate reductase alpha chain [EC:1.17.4.1]
CP004065.1_2085	K03647	75.17	120.9	1.2e-35	protein involved in ribonucleotide reduction
CP004065.1_2088	K03666	19.87	106.5	2.3e-31	host factor-I protein
CP004065.1_2089	K00791	183.90	417.3	2.3e-125	tRNA dimethylallyltransferase [EC:2.5.1.75]
CP004065.1_2092	K11814	148.07	152.9	5.9e-46	multidrug resistance protein EbrA
CP004065.1_2092	K03297	108.13	113.4	3.2e-33	small multidrug resistance pump
CP004065.1_2093	K11815	157.83	177.4	1.9e-53	multidrug resistance protein EbrB

CP004065.1_2093	K03297	108.13	141.9	6.3e-42	small multidrug resistance pump
CP004065.1_2094	K04063	57.63	71.7	1.6e-20	lipoyl-dependent peroxiredoxin [EC:1.11.1.28]
CP004065.1_2097	K17734	342.83	449.1	5.6e-135	serine protease AprX [EC:3.4.21.-]
CP004065.1_2101	K15468	837.40	889.5	5.1e-269	cytochrome P450 family 107 subfamily K polypeptide 1
CP004065.1_2102	K13615	4594.67	4792.1	0	bacillaene polyketide synthase PksR/BaeR
CP004065.1_2103	K13614	7067.73	7212.0	0	bacillaene biosynthesis, polyketide synthase / nonribosomal peptide synthetase PksN/BaeN
CP004065.1_2104	K13613	2486.10	4272.2	0	bacillaene polyketide synthase PksM/BaeM
CP004065.1_2105	K13612	6298.67	8293.8	0	bacillaene polyketide synthase PksL/BaeL
CP004065.1_2106	K13611	4676.47	5112.0	0	bacillaene biosynthesis, polyketide synthase / nonribosomal peptide synthetase PksJ/BaeJ
CP004065.1_2107	K15313	355.33	381.1	3.8e-115	4-carboxy-3-alkylbut-2-enoyl-[acp] decarboxylase [EC:4.1.1.125]
CP004065.1_2108	K15312	215.27	373.0	1.3e-112	3-carboxymethyl-3-hydroxy-acyl-[acp] dehydratase [EC:4.2.1.181]
CP004065.1_2109	K15311	506.87	720.3	1.3e-217	3-carboxymethyl-3-hydroxy-acyl-[acp] synthase [EC:2.3.3.22]
CP004065.1_2110	K15337	69.20	109.1	1.7e-32	polyketide biosynthesis acyl carrier protein
CP004065.1_2111	K15329	513.20	1181.5	0	trans-AT polyketide synthase, acyltransferase and oxidoreductase domains
CP004065.1_2111	K00645	247.17	318.2	2.7e-95	[acyl-carrier-protein] S-malonyltransferase [EC:2.3.1.39]
CP004065.1_2112	K15328	377.47	458.4	2.4e-138	bacillaene synthase trans-acting acyltransferase
CP004065.1_2113	K15327	458.17	466.9	4.4e-141	polyketide biosynthesis malonyl-CoA-[acyl-carrier-protein] transacylase
CP004065.1_2113	K00645	247.17	309.4	1.3e-92	[acyl-carrier-protein] S-malonyltransferase [EC:2.3.1.39]
CP004065.1_2114	K01069	139.83	152.3	4e-45	hydroxyacylglutathione hydrolase [EC:3.1.2.6]
CP004065.1_2116	K03572	418.80	675.1	1.9e-203	DNA mismatch repair protein MutL
CP004065.1_2117	K03555	677.40	1133.3	0	DNA mismatch repair protein MutS
CP004065.1_2118	K06328	90.60	256.6	1.9e-77	spore coat protein E
CP004065.1_2120	K06168	451.40	667.6	3.2e-201	tRNA-2-methylthio-N6-dimethylallyladenosine synthase [EC:2.8.4.3]
CP004065.1_2121	K00639	513.17	579.3	1.8e-174	glycine C-acetyltransferase [EC:2.3.1.29]
CP004065.1_2121	K00652	449.27	510.5	1.3e-153	8-amino-7-oxononanoate synthase [EC:2.3.1.47]
CP004065.1_2122	K00060	344.93	494.8	4.9e-149	threonine 3-dehydrogenase [EC:1.1.1.103]
CP004065.1_2123	K06416	55.20	163.7	2e-49	stage V sporulation protein S
CP004065.1_2124	K09769	137.70	428.7	2.2e-129	2',3'-cyclic-nucleotide 2'-phosphodiesterase [EC:3.1.4.16]
CP004065.1_2125	K18682	109.03	821.8	5.6e-248	ribonucrease Y [EC:3.1.-.-]
CP004065.1_2126	K22580	231.43	381.7	7.7e-115	teichoic acid D-alanine hydrolase [EC:3.1.1.103]
CP004065.1_2127	K03553	190.37	675.2	2.9e-203	recombination protein RecA
CP004065.1_2128	K03743	24.90	178.0	5.2e-53	nicotinamide-nucleotide amidase [EC:3.5.1.42]
CP004065.1_2129	K00995	120.80	184.0	1.1e-54	CDP-diacylglycerol---glycerol-3-phosphate 3-phosphatidyltransferase [EC:2.7.8.5]
CP004065.1_2130	K15539	70.70	164.9	8.8e-49	cytoskeleton protein RodZ
CP004065.1_2136	K06994	260.33	570.8	9.4e-172	putative drug exporter of the RND superfamily
CP004065.1_2138	K08221	232.17	413.7	1.5e-124	MFS transporter, ACDE family, multidrug resistance protein
CP004065.1_2140	K03466	191.80	790.2	9.3e-238	DNA segregation ATPase FtsK/SpoIIIE, S-DNA-T family
CP004065.1_2143	K12574	123.37	699.3	1e-210	ribonuclease J [EC:3.1.-.-]
CP004065.1_2144	K01714	259.73	388.9	7.5e-117	4-hydroxy-tetrahydrodipicolinate synthase [EC:4.3.3.7]
CP004065.1_2145	K00928	394.00	405.8	5.4e-122	aspartate kinase [EC:2.7.2.4]
CP004065.1_2146	K00133	214.73	487.0	1.6e-146	aspartate-semialdehyde dehydrogenase [EC:1.2.1.11]
CP004065.1_2147	K06411	197.03	319.3	1.5e-96	dipicolinate synthase subunit B
CP004065.1_2148	K06410	98.30	435.3	2.8e-131	dipicolinate synthase subunit A
CP004065.1_2150	K07263	220.23	258.0	5e-77	zinc protease [EC:3.4.24.-]
CP004065.1_2151	K22278	73.27	194.5	6.9e-58	peptidoglycan-N-acetylglucosamine deacetylase [EC:3.5.1.104]
CP004065.1_2152	K00962	326.70	1058.9	2.6e-319	polyribonucleotide nucleotidyltransferase [EC:2.7.7.8]
CP004065.1_2153	K02956	27.43	124.7	1.4e-36	small subunit ribosomal protein S15
CP004065.1_2154	K11753	132.60	366.1	3.6e-110	riboflavin kinase / FMN adenylyltransferase [EC:2.7.1.26 2.7.7.2]
CP004065.1_2155	K03177	194.47	353.0	6.1e-106	tRNA pseudouridine55 synthase [EC:5.4.99.25]
CP004065.1_2156	K02834	26.40	167.6	6.5e-50	ribosome-binding factor A
CP004065.1_2157	K09764	37.30	107.1	2.3e-31	uncharacterized protein
CP004065.1_2158	K02519	314.93	985.8	3.9e-297	translation initiation factor IF-2
CP004065.1_2160	K07742	20.17	85.7	7.3e-25	uncharacterized protein
CP004065.1_2161	K02600	35.63	594.5	4e-179	transcription termination/antitermination protein NusA
CP004065.1_2162	K09748	22.77	179.6	1.8e-53	ribosome maturation factor RimP
CP004065.1_2163	K03763	681.33	2197.8	0	DNA polymerase III subunit alpha, Gram-positive type [EC:2.7.7.7]

CP004065.1_2163	K02342	101.50	201.6	4.2e-60	DNA polymerase III subunit epsilon [EC:2.7.7.7]
CP004065.1_2163	K09951	46.77	83.0	6.6e-24	CRISPR-associated protein Cas2
CP004065.1_2164	K01881	163.60	530.7	1.1e-159	prolyl-tRNA synthetase [EC:6.1.1.15]
CP004065.1_2165	K11749	79.03	428.8	9.4e-129	regulator of sigma E protease [EC:3.4.24.-]
CP004065.1_2166	K00099	227.13	592.1	3e-178	1-deoxy-D-xylulose-5-phosphate reductoisomerase [EC:1.1.1.267]
CP004065.1_2167	K00981	43.57	204.8	5.8e-61	phosphatidate cytidylyltransferase [EC:2.7.7.41]
CP004065.1_2168	K00806	330.87	385.1	4.9e-116	undecaprenyl diphosphate synthase [EC:2.5.1.31]
CP004065.1_2169	K02838	25.83	243.3	7.1e-73	ribosome recycling factor
CP004065.1_2170	K09903	92.77	420.2	1.8e-126	uridylate kinase [EC:2.7.4.22]
CP004065.1_2171	K02357	112.87	379.7	6.9e-114	elongation factor Ts
CP004065.1_2172	K02967	165.67	443.7	2.3e-133	small subunit ribosomal protein S2
CP004065.1_2174	K02405	185.07	291.8	2.6e-87	RNA polymerase sigma factor FliA
CP004065.1_2175	K03411	62.07	167.9	5.8e-50	chemotaxis protein CheD [EC:3.5.1.44]
CP004065.1_2176	K03410	111.67	174.2	7.2e-52	chemotaxis protein CheC
CP004065.1_2177	K03408	105.87	125.8	4.8e-37	purine-binding chemotaxis protein CheW
CP004065.1_2178	K03407	558.90	772.5	1.8e-232	two-component system, chemotaxis family, sensor kinase CheA [EC:2.7.13.3]
CP004065.1_2179	K03412	203.57	437.8	1.9e-131	two-component system, chemotaxis family, protein-glutamate methylesterase/glutaminase [EC:3.1.1.61 3.5.1.44]
CP004065.1_2180	K04562	208.37	283.2	6.7e-85	flagellar biosynthesis protein FlhG
CP004065.1_2181	K02404	169.97	335.5	1.9e-100	flagellar biosynthesis protein FlhF
CP004065.1_2182	K02400	726.17	996.9	1.1e-300	flagellar biosynthesis protein FlhA
CP004065.1_2183	K02401	369.10	493.4	1.3e-148	flagellar biosynthesis protein FlhB
CP004065.1_2184	K02421	174.00	253.1	7.1e-76	flagellar biosynthesis protein FliR
CP004065.1_2185	K02420	87.50	126.0	1.7e-37	flagellar biosynthesis protein FliQ
CP004065.1_2186	K02419	282.17	393.2	4.1e-118	flagellar biosynthesis protein FliP
CP004065.1_2187	K02418	21.93	73.3	4.5e-21	flagellar protein FliO/FliZ
CP004065.1_2188	K03413	138.63	156.9	2e-46	two-component system, chemotaxis family, chemotaxis protein CheY
CP004065.1_2189	K02417	65.07	201.5	3.7e-60	flagellar motor switch protein FliN
CP004065.1_2190	K02416	93.77	271.2	5e-81	flagellar motor switch protein FliM
CP004065.1_2191	K02415	30.53	106.8	3e-31	flagellar protein FliL
CP004065.1_2192	K02392	292.90	297.7	3e-89	flagellar basal-body rod protein FlgG
CP004065.1_2192	K02390	168.33	169.6	2.1e-50	flagellar hook protein FlgE
CP004065.1_2193	K02389	30.80	128.2	1.4e-37	flagellar basal-body rod modification protein FlgD
CP004065.1_2194	K02414	61.60	85.1	1.8e-24	flagellar hook-length control protein FliK
CP004065.1_2195	K02383	68.77	82.0	9.7e-24	flagellar protein FlbB
CP004065.1_2196	K02413	36.40	78.0	2.4e-22	flagellar protein FliJ
CP004065.1_2197	K02412	640.17	716.9	4.7e-216	flagellum-specific ATP synthase [EC:7.4.2.8]
CP004065.1_2198	K02411	80.00	116.9	4.1e-34	flagellar assembly protein FliH
CP004065.1_2199	K02410	88.33	417.2	2.1e-125	flagellar motor switch protein FliG
CP004065.1_2200	K02409	124.47	409.0	7.4e-123	flagellar M-ring protein FliF
CP004065.1_2201	K02408	32.97	99.9	4.6e-29	flagellar hook-basal body complex protein FliE
CP004065.1_2202	K02388	55.97	181.3	4.8e-54	flagellar basal-body rod protein FlgC
CP004065.1_2203	K02387	71.43	123.3	1.8e-36	flagellar basal-body rod protein FlgB
CP004065.1_2204	K03706	127.13	401.0	4e-121	transcriptional pleiotropic repressor
CP004065.1_2205	K03667	285.07	702.1	1.3e-211	ATP-dependent HslUV protease ATP-binding subunit HslU
CP004065.1_2206	K01419	82.90	310.9	2.2e-93	ATP-dependent HslUV protease, peptidase subunit HslV [EC:3.4.25.2]
CP004065.1_2207	K03733	330.77	338.6	1.4e-101	integrase/recombinase XerC
CP004065.1_2207	K04763	301.10	307.8	3.6e-92	integrase/recombinase XerD
CP004065.1_2208	K04094	353.90	773.5	1.8e-233	methylenetetrahydrofolate--tRNA-(uracil-5-)-methyltransferase [EC:2.1.1.74]
CP004065.1_2209	K03168	448.53	974.3	1.9e-293	DNA topoisomerase I [EC:5.6.2.1]
CP004065.1_2210	K04096	28.87	322.9	1e-96	DNA processing protein
CP004065.1_2211	K01902	347.77	541.6	2.2e-163	succinyl-CoA synthetase alpha subunit [EC:6.2.1.5]
CP004065.1_2212	K01903	498.17	592.9	9e-179	succinyl-CoA synthetase beta subunit [EC:6.2.1.5]
CP004065.1_2213	K04061	105.87	121.4	7.7e-36	flagellar biosynthesis protein
CP004065.1_2215	K03470	126.33	285.3	1.2e-85	ribonuclease HII [EC:3.1.26.4]
CP004065.1_2216	K14540	297.00	435.4	3.5e-131	ribosome biogenesis GTPase A
CP004065.1_2217	K02884	25.43	194.3	4.9e-58	large subunit ribosomal protein L19

CP004065.1_2218	K00554	108.40	411.1	8.9e-124	tRNA (guanine37-N1)-methyltransferase [EC:2.1.1.228]
CP004065.1_2219	K02860	39.17	192.7	2.1e-57	16S rRNA processing protein RimM
CP004065.1_2221	K06960	46.00	98.5	8.7e-29	uncharacterized protein
CP004065.1_2222	K02959	27.40	150.3	1.3e-44	small subunit ribosomal protein S16
CP004065.1_2223	K03106	330.30	680.5	5.5e-205	signal recognition particle subunit SRP54 [EC:3.6.5.4]
CP004065.1_2224	K09787	52.33	144.6	8.3e-43	uncharacterized protein
CP004065.1_2226	K03110	289.73	488.6	3.8e-147	fused signal recognition particle receptor
CP004065.1_2227	K03529	574.17	1140.4	0	chromosome segregation protein
CP004065.1_2228	K03685	155.00	266.0	1.6e-79	ribonuclease III [EC:3.1.26.3]
CP004065.1_2229	K02078	39.80	94.5	2e-27	acyl carrier protein
CP004065.1_2230	K00059	269.80	381.7	9e-115	3-oxoacyl-[acyl-carrier protein] reductase [EC:1.1.1.100]
CP004065.1_2231	K00645	247.17	381.4	2e-114	[acyl-carrier-protein] S-malonyltransferase [EC:2.3.1.39]
CP004065.1_2232	K03621	111.17	479.2	2e-144	phosphate acyltransferase [EC:2.3.1.274]
CP004065.1_2234	K03655	393.80	761.9	4.2e-229	ATP-dependent DNA helicase RecG [EC:5.6.2.4]
CP004065.1_2235	K01752	73.17	336.9	6.3e-101	L-serine dehydratase [EC:4.3.1.17]
CP004065.1_2236	K01752	73.17	190.2	1.6e-56	L-serine dehydratase [EC:4.3.1.17]
CP004065.1_2237	K07030	292.60	593.0	2.2e-178	fatty acid kinase [EC:2.7.2.18]
CP004065.1_2239	K02902	22.37	57.2	4.6e-16	large subunit ribosomal protein L28
CP004065.1_2240	K00949	46.80	136.4	3.3e-40	thiamine pyrophosphokinase [EC:2.7.6.2]
CP004065.1_2241	K01783	248.20	368.0	1e-110	ribulose-phosphate 3-epimerase [EC:5.1.3.1]
CP004065.1_2242	K06949	90.53	342.1	9.7e-103	ribosome biogenesis GTPase / thiamine phosphate phosphatase [EC:3.6.1.- 3.1.3.100]
CP004065.1_2243	K12132	224.03	342.0	7.2e-103	eukaryotic-like serine/threonine-protein kinase [EC:2.7.11.1]
CP004065.1_2243	K11912	105.07	188.9	3.3e-56	serine/threonine-protein kinase PpkA [EC:2.7.11.1]
CP004065.1_2244	K20074	131.13	330.2	8.3e-99	PPM family protein phosphatase [EC:3.1.3.16]
CP004065.1_2245	K06941	65.87	522.5	2.9e-157	23S rRNA (adenine2503-C2)-methyltransferase [EC:2.1.1.192]
CP004065.1_2246	K03500	303.70	516.7	3.3e-155	16S rRNA (cytosine967-C5)-methyltransferase [EC:2.1.1.176]
CP004065.1_2247	K00604	267.47	381.5	1.4e-114	methionyl-tRNA formyltransferase [EC:2.1.2.9]
CP004065.1_2248	K01462	69.73	214.9	3.7e-64	peptide deformylase [EC:3.5.1.88]
CP004065.1_2249	K04066	138.70	945.6	7.5e-285	primosomal protein N' (replication factor Y) (superfamily II helicase) [EC:5.6.2.4]
CP004065.1_2250	K13038	251.03	562.7	1.3e-169	phosphopantothenoylcysteine decarboxylase / phosphopantothenate---cysteine ligase [EC:4.1.1.36 6.3.2.5]
CP004065.1_2251	K03060	24.57	60.3	5.9e-17	DNA-directed RNA polymerase subunit omega [EC:2.7.7.6]
CP004065.1_2252	K00942	178.33	242.5	1.3e-72	guanylate kinase [EC:2.7.4.8]
CP004065.1_2253	K09777	63.00	148.1	1.3e-44	extracellular matrix regulatory protein A
CP004065.1_2255	K01537	992.03	1138.2	0	P-type Ca2+ transporter type 2C [EC:7.2.2.10]
CP004065.1_2257	K24866	158.93	177.5	8e-53	precorrin-2 dehydrogenase [EC:1.3.1.76]
CP004065.1_2258	K03795	119.77	209.8	1.8e-62	sirohydrochlorin cobaltochelatase [EC:4.99.1.3]
CP004065.1_2258	K03794	134.27	141.3	7.6e-42	sirohydrochlorin ferrochelatase [EC:4.99.1.4]
CP004065.1_2259	K02303	255.53	391.5	9.8e-118	uroporphyrin-III C-methyltransferase [EC:2.1.1.107]
CP004065.1_2260	K00860	304.20	325.1	1.4e-97	adenylylsulfate kinase [EC:2.7.1.25]
CP004065.1_2261	K00958	267.50	548.8	3.4e-165	sulfate adenylyltransferase [EC:2.7.7.4]
CP004065.1_2262	K16331	359.10	437.0	6.5e-132	sulfate permease
CP004065.1_2263	K00390	157.87	302.9	6.2e-91	phosphoadenosine phosphosulfate reductase [EC:1.8.4.8 1.8.4.10]
CP004065.1_2265	K00762	105.33	195.7	2.5e-58	orotate phosphoribosyltransferase [EC:2.4.2.10]
CP004065.1_2266	K01591	129.83	225.5	3e-67	orotidine-5'-phosphate decarboxylase [EC:4.1.1.23]
CP004065.1_2267	K17828	251.20	495.4	1.9e-149	dihydroorotate dehydrogenase (NAD+) catalytic subunit [EC:1.3.1.14]
CP004065.1_2268	K02823	242.20	307.3	2.4e-92	dihydroorotate dehydrogenase electron transfer subunit
CP004065.1_2269	K01955	1479.70	1683.4	0	carbamoyl-phosphate synthase large subunit [EC:6.3.5.5]
CP004065.1_2270	K01956	530.93	620.2	1.7e-186	carbamoyl-phosphate synthase small subunit [EC:6.3.5.5]
CP004065.1_2271	K01465	336.03	474.7	1.2e-142	dihydroorotase [EC:3.5.2.3]
CP004065.1_2272	K00609	254.20	402.9	3.6e-121	aspartate carbamoyltransferase catalytic subunit [EC:2.1.3.2]
CP004065.1_2273	K02824	450.17	612.7	8.8e-185	uracil permease
CP004065.1_2274	K02825	96.10	284.1	2.2e-85	pyrimidine operon attenuation protein / uracil phosphoribosyltransferase [EC:2.4.2.9]
CP004065.1_2275	K06180	285.23	427.6	1.3e-128	23S rRNA pseudouridine1911/1915/1917 synthase [EC:5.4.99.23]
CP004065.1_2276	K03101	31.87	180.7	6.5e-54	signal peptidase II [EC:3.4.23.36]
CP004065.1_2277	K06204	35.73	43.3	9.3e-12	RNA polymerase-binding transcription factor
CP004065.1_2278	K01870	467.30	1135.7	0	isoleucyl-tRNA synthetase [EC:6.1.1.5]

CP004065.1_2279	K04074	47.10	212.4	1.7e-63	cell division initiation protein
CP004065.1_2281	K02221	21.77	99.3	5.5e-29	YggT family protein
CP004065.1_2282	K09772	38.13	159.7	1.6e-47	cell division inhibitor SepF
CP004065.1_2283	K06997	64.17	278.3	2.3e-83	PLP dependent protein
CP004065.1_2284	K05810	84.47	263.2	9.8e-79	purine-nucleoside/S-methyl-5'-thioadenosine phosphorylase / adenosine deaminase [EC:2.4.2.1 2.4.2.28 3.5.4.4]
CP004065.1_2286	K20895	468.37	701.6	5.3e-212	formylaminopyrimidine deformylase [EC:3.5.1.-]
CP004065.1_2288	K03091	189.70	268.4	1.7e-80	RNA polymerase sigma-E/F/G factor
CP004065.1_2289	K03091	189.70	355.2	6.8e-107	RNA polymerase sigma-E/F/G factor
CP004065.1_2290	K06383	109.60	329.3	5.4e-99	stage II sporulation protein GA (sporulation sigma-E factor processing peptidase) [EC:3.4.23.-]
CP004065.1_2291	K13276	906.03	2092.9	0	bacillopeptidase F [EC:3.4.21.-]
CP004065.1_2292	K03531	142.77	567.1	5.5e-171	cell division protein FtsZ
CP004065.1_2293	K03590	124.13	488.7	3.9e-147	cell division protein FtsA
CP004065.1_2294	K03589	42.57	131.7	1.1e-38	cell division protein FtsQ
CP004065.1_2295	K00075	66.30	342.7	5.8e-103	UDP-N-acetylmuramate dehydrogenase [EC:1.3.1.98]
CP004065.1_2296	K02563	193.67	398.3	1.2e-119	UDP-N-acetylglucosamine--N-acetylmuramyl-(pentapeptide) pyrophosphoryl-undecaprenol N-acetylglucosamine transferase [EC:2.4.1.227]
CP004065.1_2297	K03588	356.93	450.0	1.8e-135	cell division protein FtsW
CP004065.1_2298	K01925	363.80	475.8	3.8e-143	UDP-N-acetylmuramoylalanine--D-glutamate ligase [EC:6.3.2.9]
CP004065.1_2299	K01000	186.50	434.0	1.4e-130	phospho-N-acetylmuramoyl-pentapeptide-transferase [EC:2.7.8.13]
CP004065.1_2300	K01928	408.67	654.7	3e-197	UDP-N-acetylmuramoyl-L-alanyl-D-glutamate--2,6-diaminopimelate ligase [EC:6.3.2.13]
CP004065.1_2301	K08384	704.83	869.4	5.1e-262	stage V sporulation protein D (sporulation-specific penicillin-binding protein)
CP004065.1_2301	K03587	463.30	587.9	6.2e-177	cell division protein FtsI (penicillin-binding protein 3) [EC:3.4.16.4]
CP004065.1_2302	K08724	849.27	1029.3	1.7e-310	penicillin-binding protein 2B
CP004065.1_2302	K03587	463.30	522.4	4.1e-157	cell division protein FtsI (penicillin-binding protein 3) [EC:3.4.16.4]
CP004065.1_2303	K03586	43.53	50.2	7.1e-14	cell division protein FtsL
CP004065.1_2304	K03438	382.10	466.4	2.3e-140	16S rRNA (cytosine1402-N4)-methyltransferase [EC:2.1.1.199]
CP004065.1_2305	K03925	34.60	192.8	8.8e-58	transcriptional regulator MraZ
CP004065.1_2306	K22136	114.20	539.3	2.7e-162	bacillithiol synthase
CP004065.1_2307	K00077	75.90	231.3	5.5e-69	2-dehydropantoate 2-reductase [EC:1.1.1.169]
CP004065.1_2309	K06314	87.53	228.9	1.3e-68	prespore-specific regulator
CP004065.1_2310	K02911	22.10	72.8	7.8e-21	large subunit ribosomal protein L32
CP004065.1_2311	K07040	18.17	109.0	7.9e-32	DUF177 domain-containing protein
CP004065.1_2312	K27343	225.90	516.2	2e-155	tRNAmet cytidine acetate ligase [EC:6.3.1.22]
CP004065.1_2313	K07177	99.10	365.4	7.8e-110	Lon-like protease
CP004065.1_2314	K07001	95.57	256.9	7.2e-77	NTE family protein
CP004065.1_2316	K00954	81.30	263.8	3.3e-79	pantetheine-phosphate adenylyltransferase [EC:2.7.7.3]
CP004065.1_2317	K08316	94.87	205.2	2.7e-61	16S rRNA (guanine966-N2)-methyltransferase [EC:2.1.1.171]
CP004065.1_2326	K02862	202.27	365.7	4e-110	putative membrane protein
CP004065.1_2327	K02277	50.30	61.2	2.7e-17	cytochrome c oxidase subunit IV [EC:7.1.1.9]
CP004065.1_2329	K02274	750.43	803.4	4.8e-242	cytochrome c oxidase subunit I [EC:7.1.1.9]
CP004065.1_2330	K02275	207.23	225.1	4.4e-67	cytochrome c oxidase subunit II [EC:7.1.1.9]
CP004065.1_2330	K08738	50.00	50.3	5.9e-14	cytochrome c
CP004065.1_2331	K02257	126.90	267.4	5.5e-80	heme o synthase [EC:2.5.1.141]
CP004065.1_2332	K02259	62.60	156.9	1.4e-46	heme a synthase [EC:1.17.99.9]
CP004065.1_2333	K01958	801.27	1968.4	0	pyruvate carboxylase [EC:6.4.1.1]
CP004065.1_2334	K03588	356.93	424.4	1.1e-127	cell division protein FtsW
CP004065.1_2336	K01425	83.30	379.5	6.9e-114	glutaminase [EC:3.5.1.2]
CP004065.1_2338	K07175	228.10	616.8	1.1e-185	PhoH-like ATPase
CP004065.1_2342	K06207	327.10	1014.9	4e-306	GTP-binding protein
CP004065.1_2346	K01400	510.83	616.5	1.9e-185	bacillolysin [EC:3.4.24.28]
CP004065.1_2348	K01092	211.57	286.1	1.1e-85	myo-inositol-1(or 4)-monophosphatase [EC:3.1.3.25]
CP004065.1_2352	K01585	258.33	603.6	1.6e-181	arginine decarboxylase [EC:4.1.1.19]
CP004065.1_2353	K22278	73.27	147.8	1e-43	peptidoglycan-N-acetylglucosamine deacetylase [EC:3.5.1.104]
CP004065.1_2355	K00382	465.60	660.0	6.5e-199	dihydrolipoyl dehydrogenase [EC:1.8.1.4]
CP004065.1_2356	K00627	488.13	520.4	1.8e-156	pyruvate dehydrogenase E2 component (dihydrolipoyllysine-residue acetyltransferase) [EC:2.3.1.12]
CP004065.1_2356	K09699	429.73	506.8	1.2e-152	2-oxoisovalerate dehydrogenase E2 component (dihydrolipoyl transacylase) [EC:2.3.1.168]
CP004065.1_2360	K25848	3026.07	3028.3	0	macrolactin polyketide synthase MlnH

CP004065.1_2361	K25847	5006.00	5921.0	0	macrolactin polyketide synthase MlnG
CP004065.1_2362	K25846	4148.23	4579.2	0	macrolactin polyketide synthase MlnF
CP004065.1_2363	K25845	3127.50	3957.6	0	macrolactin polyketide synthase MlnE
CP004065.1_2366	K25842	9803.30	9813.3	0	macrolactin polyketide synthase MlnB
CP004065.1_2367	K15329	513.20	1135.4	0	trans-AT polyketide synthase, acyltransferase and oxidoreductase domains
CP004065.1_2367	K00645	247.17	337.9	3.1e-101	[acyl-carrier-protein] S-malonyltransferase [EC:2.3.1.39]
CP004065.1_2370	K01462	69.73	159.8	2.3e-47	peptide deformylase [EC:3.5.1.88]
CP004065.1_2371	K21064	166.97	176.3	1.8e-52	5-amino-6-(5-phospho-D-ribitylamino)uracil phosphatase [EC:3.1.3.104]
CP004065.1_2373	K12574	123.37	799.1	6.3e-241	ribonuclease J [EC:3.1.-.-]
CP004065.1_2374	K01486	325.67	756.0	5.9e-228	adenine deaminase [EC:3.5.4.2]
CP004065.1_2375	K03499	127.40	183.1	1.6e-54	trk/ktr system potassium uptake protein
CP004065.1_2377	K07698	415.90	521.9	5e-157	two-component system, sporulation sensor kinase C [EC:2.7.13.3]
CP004065.1_2378	K06284	86.83	109.9	1.4e-32	AbrB family transcriptional regulator, transcriptional pleiotropic regulator of transition state genes
CP004065.1_2379	K03569	108.73	491.3	4.3e-148	rod shape-determining protein MreB and related proteins
CP004065.1_2381	K19689	69.60	460.4	2.4e-138	aminopeptidase [EC:3.4.11.-]
CP004065.1_2382	K00077	75.90	244.2	6.5e-73	2-dehydropantoate 2-reductase [EC:1.1.1.169]
CP004065.1_2385	K03100	114.77	201.9	3.1e-60	signal peptidase I [EC:3.4.21.89]
CP004065.1_2386	K02770	649.43	875.5	5.8e-264	fructose PTS system EIIBC or EIIC component [EC:2.7.1.202]
CP004065.1_2386	K02768	144.33	178.6	3.4e-53	fructose PTS system EIIA component [EC:2.7.1.202]
CP004065.1_2387	K00882	316.47	357.7	1.7e-107	1-phosphofructokinase [EC:2.7.1.56]
CP004065.1_2388	K03436	282.67	289.3	5.9e-87	DeoR family transcriptional regulator, fructose operon transcriptional repressor
CP004065.1_2389	K24948	398.40	472.0	4.4e-142	antimicrobial peptide resistance transport system permease protein
CP004065.1_2389	K02004	52.33	130.8	1.1e-38	putative ABC transport system permease protein
CP004065.1_2390	K24949	338.63	356.6	8.5e-108	antimicrobial peptide resistance transport system ATP-binding protein [EC:7.6.2.-]
CP004065.1_2390	K02003	292.97	353.6	3e-106	putative ABC transport system ATP-binding protein
CP004065.1_2391	K24950	219.27	325.9	8.7e-98	membrane fusion protein, antimicrobial resistance system
CP004065.1_2392	K24951	84.43	269.3	5.3e-81	membrane protein, antimicrobial resistance system
CP004065.1_2393	K03636	36.60	55.6	1.5e-15	sulfur-carrier protein
CP004065.1_2394	K03635	25.83	200.6	9.6e-60	molybdopterin synthase catalytic subunit [EC:2.8.1.12]
CP004065.1_2395	K03753	70.57	127.8	1.3e-37	molybdopterin-guanine dinucleotide biosynthesis adapter protein
CP004065.1_2396	K03750	141.83	444.3	1.2e-133	molybdopterin molybdotransferase [EC:2.10.1.1]
CP004065.1_2396	K07219	145.43	149.3	3.5e-44	putative molybdopterin biosynthesis protein
CP004065.1_2397	K03148	333.67	345.9	5.5e-104	sulfur carrier protein ThiS adenylyltransferase [EC:2.7.7.73]
CP004065.1_2398	K03752	92.73	143.2	2.3e-42	molybdenum cofactor guanylyltransferase [EC:2.7.7.77]
CP004065.1_2399	K06437	239.87	438.1	3.8e-132	sigma-E controlled sporulation protein
CP004065.1_2400	K26248	122.53	245.1	8.7e-74	repressor Rok
CP004065.1_2402	K22044	182.20	275.8	1.6e-82	moderate conductance mechanosensitive channel
CP004065.1_2404	K05823	359.63	599.9	3.1e-181	N-acetyldiaminopimelate deacetylase [EC:3.5.1.47]
CP004065.1_2405	K05822	338.70	379.4	1.2e-114	tetrahydrodipicolinate N-acetyltransferase [EC:2.3.1.89]
CP004065.1_2406	K03839	94.63	142.0	5e-42	flavodoxin I
CP004065.1_2408	K03839	94.63	139.1	3.9e-41	flavodoxin I
CP004065.1_2412	K09776	96.80	198.4	1.2e-59	uncharacterized protein
CP004065.1_2417	K07098	96.03	274.9	2.5e-82	uncharacterized protein
CP004065.1_2421	K03415	178.73	324.5	2.4e-97	two-component system, chemotaxis family, chemotaxis protein CheV
CP004065.1_2423	K00841	489.23	556.1	1.7e-167	aminotransferase [EC:2.6.1.-]
CP004065.1_2424	K02491	453.23	489.3	3.6e-147	two-component system, sporulation sensor kinase A [EC:2.7.13.3]
CP004065.1_2425	K21466	973.00	1295.6	0	penicillin-binding protein H
CP004065.1_2428	K00020	348.20	378.3	1.7e-113	3-hydroxyisobutyrate dehydrogenase [EC:1.1.1.31]
CP004065.1_2429	K03406	65.50	276.8	8.9e-83	methyl-accepting chemotaxis protein
CP004065.1_2430	K03716	305.83	577.7	1.3e-174	spore photoproduct lyase [EC:4.1.99.14]
CP004065.1_2431	K06315	84.00	119.1	1.2e-35	transcriptional regulator of the spore photoproduct lyase operon
CP004065.1_2432	K08483	678.63	858.2	1.5e-258	phosphoenolpyruvate-protein phosphotransferase (PTS system enzyme I) [EC:2.7.3.9]
CP004065.1_2433	K02784	81.30	113.2	2.9e-33	phosphocarrier protein HPr
CP004065.1_2434	K20118	807.70	992.2	2.3e-299	glucose PTS system EIICBA or EIICB component [EC:2.7.1.199]
CP004065.1_2434	K02791	716.30	766.7	3.9e-231	maltose/glucose PTS system EIICB component [EC:2.7.1.199 2.7.1.208]
CP004065.1_2435	K03480	295.03	404.6	4.2e-122	transcriptional antiterminator

CP004065.1_2439	K01534	713.30	735.9	1.1e-221	Zn2+/Cd2+-exporting ATPase [EC:7.2.2.12 7.2.2.21]
CP004065.1_2442	K01449	48.60	174.4	6e-52	cell wall hydrolase
CP004065.1_2446	K09457	104.03	238.3	1.4e-71	7-cyano-7-deazaguanine reductase [EC:1.7.1.13]
CP004065.1_2447	K10026	81.40	193.3	1.1e-57	7-carboxy-7-deazaguanine synthase [EC:4.3.99.3]
CP004065.1_2448	K01737	24.80	119.9	2.7e-35	6-pyruvoyltetrahydropterin/6-carboxytetrahydropterin synthase [EC:4.2.3.12 4.1.2.50]
CP004065.1_2449	K06920	96.20	320.4	2.5e-96	7-cyano-7-deazaguanine synthase [EC:6.3.4.20]
CP004065.1_2452	K03697	944.33	1130.5	0	ATP-dependent Clp protease ATP-binding subunit ClpE
CP004065.1_2453	K02556	81.20	309.3	8.7e-93	chemotaxis protein MotA
CP004065.1_2454	K02557	144.00	221.7	2.9e-66	chemotaxis protein MotB
CP004065.1_2455	K15973	110.17	211.7	1.7e-63	MarR family transcriptional regulator, 2-MHQ and catechol-resistance regulon repressor
CP004065.1_2456	K13532	306.67	483.7	1.3e-145	two-component system, sporulation sensor kinase D [EC:2.7.13.3]
CP004065.1_2457	K06376	38.70	81.8	6.5e-24	stage 0 sporulation regulatory protein
CP004065.1_2458	K08967	33.60	117.8	1.2e-34	1,2-dihydroxy-3-keto-5-methylthiopentene dioxygenase [EC:1.13.11.53 1.13.11.54]
CP004065.1_2459	K08964	126.67	193.3	1.7e-57	methylthioribulose-1-phosphate dehydratase [EC:4.2.1.109]
CP004065.1_2460	K08966	123.57	240.4	3.9e-72	2-hydroxy-3-keto-5-methylthiopentenyl-1-phosphate phosphatase [EC:3.1.3.87]
CP004065.1_2461	K08965	375.83	633.6	1.9e-191	2,3-diketo-5-methylthiopentyl-1-phosphate enolase [EC:5.3.2.5]
CP004065.1_2462	K08969	544.47	666.7	2.3e-201	L-glutamine---4-(methylsulfanyl)-2-oxobutanoate aminotransferase [EC:2.6.1.117]
CP004065.1_2464	K00899	378.97	461.5	1.1e-138	5-methylthioribose kinase [EC:2.7.1.100]
CP004065.1_2465	K08963	276.73	496.7	1.9e-149	methylthioribose-1-phosphate isomerase [EC:5.3.1.23]
CP004065.1_2467	K00567	174.97	190.4	1.2e-56	methylated-DNA-[protein]-cysteine S-methyltransferase [EC:2.1.1.63]
CP004065.1_2468	K13533	408.10	456.5	2.7e-137	two-component system, sporulation sensor kinase E [EC:2.7.13.3]
CP004065.1_2472	K03498	117.37	410.8	1.8e-123	trk/ktr system potassium uptake protein
CP004065.1_2473	K03799	127.53	230.5	6.8e-69	heat shock protein HtpX [EC:3.4.24.-]
CP004065.1_2475	K06421	91.77	100.7	1e-29	small acid-soluble spore protein D (minor alpha/beta-type SASP)
CP004065.1_2477	K03093	118.63	252.6	1.5e-75	RNA polymerase sigma-I factor
CP004065.1_2479	K03975	59.97	93.4	3.1e-27	membrane-associated protein
CP004065.1_2480	K10979	98.23	376.5	2.8e-113	DNA end-binding protein Ku
CP004065.1_2481	K01971	209.27	496.5	4.5e-149	bifunctional non-homologous end joining protein LigD [EC:6.5.1.1]
CP004065.1_2483	K15973	110.17	179.4	1.4e-53	MarR family transcriptional regulator, 2-MHQ and catechol-resistance regulon repressor
CP004065.1_2486	K23776	96.67	142.0	1.2e-42	MerR family transcriptional regulator, global nitrogen regulator
CP004065.1_2487	K06213	106.03	519.9	1.9e-156	magnesium transporter
CP004065.1_2489	K06306	390.77	392.0	5.5e-118	cortical fragment-lytic enzyme [EC:3.2.1.-]
CP004065.1_2490	K16925	57.77	205.9	1.2e-61	energy-coupling factor transport system permease protein
CP004065.1_2491	K01552	403.80	599.4	1.9e-180	energy-coupling factor transport system ATP-binding protein [EC:7.-.-.-]
CP004065.1_2492	K16785	103.27	139.7	2.2e-41	energy-coupling factor transport system permease protein
CP004065.1_2493	K17763	101.07	191.4	7.1e-57	rsbT co-antagonist protein RsbR
CP004065.1_2494	K13275	341.67	448.3	3.1e-135	major intracellular serine protease [EC:3.4.21.-]
CP004065.1_2495	K00549	27.93	952.3	6.2e-287	5-methyltetrahydropteroyltriglutamate--homocysteine methyltransferase [EC:2.1.1.14]
CP004065.1_2496	K16326	165.60	286.7	2.3e-86	CRP/FNR family transcriptional regulator, putaive post-exponential-phase nitrogen-starvation regulator
CP004065.1_2497	K05820	181.27	282.2	1e-84	MFS transporter, PPP family, 3-phenylpropionic acid transporter
CP004065.1_2502	K04063	57.63	171.1	6.8e-51	lipoyl-dependent peroxiredoxin [EC:1.11.1.28]
CP004065.1_2503	K23775	99.10	181.3	6.9e-54	MarR family transcriptional regulator, organic hydroperoxide resistance regulator
CP004065.1_2504	K04063	57.63	167.4	9.1e-50	lipoyl-dependent peroxiredoxin [EC:1.11.1.28]
CP004065.1_2505	K00147	552.77	638.0	4.1e-192	glutamate-5-semialdehyde dehydrogenase [EC:1.2.1.41]
CP004065.1_2506	K00931	289.63	460.3	2.7e-138	glutamate 5-kinase [EC:2.7.2.11]
CP004065.1_2507	K01433	204.20	438.4	4.8e-132	formyltetrahydrofolate deformylase [EC:3.5.1.10]
CP004065.1_2508	K18925	114.07	135.5	1.6e-40	paired small multidrug resistance pump
CP004065.1_2509	K18924	121.87	181.0	2.1e-54	paired small multidrug resistance pump
CP004065.1_2509	K11741	110.40	110.5	1.5e-32	quaternary ammonium compound-resistance protein SugE
CP004065.1_2511	K01073	193.53	205.6	8e-62	acyl-CoA hydrolase [EC:3.1.2.20]
CP004065.1_2512	K01482	107.33	137.4	1.5e-40	dimethylargininase [EC:3.5.3.18]
CP004065.1_2513	K07404	130.17	426.4	3.6e-128	6-phosphogluconolactonase [EC:3.1.1.31]
CP004065.1_2514	K02032	420.67	523.5	2.4e-157	peptide/nickel transport system ATP-binding protein
CP004065.1_2515	K20742	170.83	326.8	4.9e-98	gamma-D-glutamyl-L-lysine dipeptidyl-peptidase [EC:3.4.14.13]
CP004065.1_2516	K19802	302.33	420.2	2.5e-126	L-Ala-D/L-Glu epimerase / N-acetyl-D-glutamate racemase [EC:5.1.1.20 5.1.1.25]
CP004065.1_2517	K01297	137.13	363.0	4e-109	muramoyltetrapeptide carboxypeptidase [EC:3.4.17.13]

CP004065.1_2518	K16199	703.73	910.5	6.5e-275	dipeptide transport system substrate-binding protein
CP004065.1_2518	K15580	368.50	546.3	2.7e-164	oligopeptide transport system substrate-binding protein
CP004065.1_2518	K02035	249.67	332.7	1.1e-99	peptide/nickel transport system substrate-binding protein
CP004065.1_2519	K16202	578.17	598.7	5e-181	dipeptide transport system ATP-binding protein
CP004065.1_2519	K15583	519.70	536.8	1e-161	oligopeptide transport system ATP-binding protein
CP004065.1_2519	K02031	412.13	507.8	9.6e-153	peptide/nickel transport system ATP-binding protein
CP004065.1_2520	K16201	523.57	568.0	7.7e-172	dipeptide transport system permease protein
CP004065.1_2520	K15582	364.50	432.3	2e-130	oligopeptide transport system permease protein
CP004065.1_2520	K02034	256.80	364.4	2e-109	peptide/nickel transport system permease protein
CP004065.1_2521	K16200	471.20	524.0	3e-158	dipeptide transport system permease protein
CP004065.1_2521	K15581	345.63	388.5	7.5e-117	oligopeptide transport system permease protein
CP004065.1_2521	K02033	263.63	367.5	2.4e-110	peptide/nickel transport system permease protein
CP004065.1_2522	K16203	84.30	322.0	8.2e-97	D-amino peptidase [EC:3.4.11.-]
CP004065.1_2526	K20534	338.93	509.3	2e-153	polyisoprenyl-phosphate glycosyltransferase [EC:2.4.-.-]
CP004065.1_2528	K15975	357.47	422.6	4.1e-127	glyoxalase family protein
CP004065.1_2530	K07220	65.73	177.5	8.5e-53	uncharacterized protein
CP004065.1_2531	K03306	385.20	461.2	9.7e-139	inorganic phosphate transporter, PiT family
CP004065.1_2532	K06388	185.57	392.0	2.1e-118	stage II sporulation protein SA
CP004065.1_2533	K06389	49.77	99.9	1.1e-29	stage II sporulation protein SB
CP004065.1_2534	K01447	118.50	240.8	4.5e-72	N-acetylmuramoyl-L-alanine amidase [EC:3.5.1.28]
CP004065.1_2561	K06909	42.80	354.0	4.2e-106	phage terminase large subunit
CP004065.1_2562	K07474	21.07	155.7	4.7e-46	phage terminase small subunit
CP004065.1_2563	K24072	90.50	193.1	6.1e-58	positive control factor
CP004065.1_2567	K02315	147.80	158.3	7.6e-47	DNA replication protein DnaC
CP004065.1_2574	K01447	118.50	229.5	1.3e-68	N-acetylmuramoyl-L-alanine amidase [EC:3.5.1.28]
CP004065.1_2575	K06352	97.03	104.4	4.5e-31	phosphatase RapA inhibitor
CP004065.1_2576	K06359	540.97	607.2	2.3e-183	response regulator aspartate phosphatase A (stage 0 sporulation protein L) [EC:3.1.-.-]
CP004065.1_2578	K07090	37.03	133.1	3e-39	uncharacterized protein
CP004065.1_2579	K27862	199.47	634.1	1.1e-190	class II lanthipeptide synthase [EC:3.13.2.4]
CP004065.1_2583	K27862	199.47	782.7	1.4e-235	class II lanthipeptide synthase [EC:3.13.2.4]
CP004065.1_2585	K07691	194.87	262.4	5.6e-79	two-component system, NarL family, competent response regulator ComA
CP004065.1_2586	K07680	232.70	255.5	2.3e-76	two-component system, NarL family, sensor histidine kinase ComP [EC:2.7.13.3]
CP004065.1_2592	K01685	552.00	750.2	2.2e-226	altronate hydrolase [EC:4.2.1.7]
CP004065.1_2593	K00041	288.63	635.3	1.7e-191	tagaturonate reductase [EC:1.1.1.58]
CP004065.1_2594	K26609	372.43	535.3	1.1e-161	LacI family transcriptional regulator, galacturonate utilization repressor
CP004065.1_2594	K02529	268.37	299.0	1.6e-89	LacI family transcriptional regulator, galactose operon repressor
CP004065.1_2595	K03292	344.60	434.2	1.3e-130	glycoside/pentoside/hexuronide:cation symporter, GPH family
CP004065.1_2596	K01812	195.40	656.5	7.6e-198	glucuronate isomerase [EC:5.3.1.12]
CP004065.1_2597	K03885	203.30	382.7	9.3e-115	NADH:quinone reductase (non-electrogenic) [EC:1.6.5.9]
CP004065.1_2602	K02069	66.73	266.2	1.5e-79	UDP-glucose/iron transport system permease protein
CP004065.1_2603	K01719	43.23	104.9	1.1e-30	uroporphyrinogen-III synthase [EC:4.2.1.75]
CP004065.1_2605	K25230	530.87	679.3	3e-205	NDP-glycosyltransferase [EC:2.4.1.384]
CP004065.1_2606	K23138	486.53	572.1	1.2e-172	cytochrome P450 family 109 [EC:1.14.-.-]
CP004065.1_2608	K00123	847.33	1064.9	0	formate dehydrogenase major subunit [EC:1.17.1.9]
CP004065.1_2613	K07507	42.27	217.4	6.7e-65	putative Mg2+ transporter-C (MgtC) family protein
CP004065.1_2617	K02530	240.17	340.6	1.1e-102	DeoR family transcriptional regulator, lactose phosphotransferase system repressor
CP004065.1_2618	K01220	556.97	787.2	8.6e-238	6-phospho-beta-galactosidase [EC:3.2.1.85]
CP004065.1_2619	K02786	128.13	155.9	5.7e-47	lactose PTS system EIIA component [EC:2.7.1.207]
CP004065.1_2619	K02759	33.93	114.0	1.2e-33	cellobiose PTS system EIIA component [EC:2.7.1.196 2.7.1.205]
CP004065.1_2620	K02788	455.87	887.2	8e-268	lactose PTS system EIICB component [EC:2.7.1.207]
CP004065.1_2620	K02761	189.70	357.4	2.6e-107	cellobiose PTS system EIIC component
CP004065.1_2621	K00849	368.57	465.0	1.1e-139	galactokinase [EC:2.7.1.6]
CP004065.1_2622	K01784	253.97	416.0	6e-125	UDP-glucose 4-epimerase [EC:5.1.3.2]
CP004065.1_2624	K01224	233.03	431.5	1.7e-129	arabinogalactan endo-1,4-beta-galactosidase [EC:3.2.1.89]
CP004065.1_2628	K17836	82.80	304.5	3.3e-91	beta-lactamase class A [EC:3.5.2.6]
CP004065.1_2633	K21497	52.47	57.4	2.7e-16	antitoxin

CP004065.1_2639	K01447	118.50	231.4	3.3e-69	N-acetylmuramoyl-L-alanine amidase [EC:3.5.1.28]
CP004065.1_2661	K06909	42.80	116.8	3e-34	phage terminase large subunit
CP004065.1_2667	K24072	90.50	215.8	6.4e-65	positive control factor
CP004065.1_2671	K06223	67.20	231.7	3.8e-69	DNA adenine methylase [EC:2.1.1.72]
CP004065.1_2676	K01160	31.43	61.5	2.3e-17	crossover junction endodeoxyribonuclease RusA [EC:3.1.21.10]
CP004065.1_2678	K02315	147.80	169.9	2.2e-50	DNA replication protein DnaC
CP004065.1_2679	K02086	54.33	57.7	2.5e-16	DNA replication protein
CP004065.1_2688	K07741	35.90	82.6	1.1e-23	anti-repressor protein
CP004065.1_2690	K07729	60.67	62.3	1e-17	putative transcriptional regulator
CP004065.1_2694	K14059	167.87	214.5	7.5e-64	integrase
CP004065.1_2695	K01760	493.17	556.4	1e-167	cysteine-S-conjugate beta-lyase [EC:4.4.1.13]
CP004065.1_2701	K01990	262.37	292.4	1.4e-87	ABC-2 type transport system ATP-binding protein
CP004065.1_2703	K17837	146.13	168.8	4.2e-50	metallo-beta-lactamase class B [EC:3.5.2.6]
CP004065.1_2705	K03657	367.57	542.9	3.3e-163	ATP-dependent DNA helicase UvrD/PcrA [EC:5.6.2.4]
CP004065.1_2711	K06340	163.97	213.9	1.2e-64	spore coat protein V
CP004065.1_2712	K06341	40.50	91.6	6.6e-27	spore coat protein W
CP004065.1_2713	K06342	103.30	178.9	2e-53	spore coat protein X
CP004065.1_2714	K06343	55.50	184.4	4.9e-55	CotY/CotZ family spore coat protein
CP004065.1_2715	K06343	55.50	153.7	1.2e-45	CotY/CotZ family spore coat protein
CP004065.1_2717	K00208	269.47	403.1	3e-121	enoyl-[acyl-carrier protein] reductase I [EC:1.3.1.9 1.3.1.10]
CP004065.1_2718	K00941	324.10	371.5	1.9e-111	hydroxymethylpyrimidine/phosphomethylpyrimidine kinase [EC:2.7.1.49 2.7.4.7]
CP004065.1_2719	K03148	333.67	356.7	3e-107	sulfur carrier protein ThiS adenylyltransferase [EC:2.7.7.73]
CP004065.1_2720	K03149	188.47	436.4	4.9e-131	thiazole synthase [EC:2.8.1.10]
CP004065.1_2721	K03154	35.33	64.8	1.5e-18	sulfur carrier protein
CP004065.1_2722	K03153	270.03	331.3	2e-99	glycine oxidase [EC:1.4.3.19]
CP004065.1_2723	K10810	208.97	269.2	6e-81	thiazole tautomerase (transcriptional regulator TenI) [EC:5.3.99.10]
CP004065.1_2724	K03707	196.20	268.5	1.6e-80	thiaminase (transcriptional activator TenA) [EC:3.5.99.2]
CP004065.1_2727	K06180	285.23	324.8	2.1e-97	23S rRNA pseudouridine1911/1915/1917 synthase [EC:5.4.99.23]
CP004065.1_2728	K00858	38.63	136.8	2e-40	NAD+ kinase [EC:2.7.1.23]
CP004065.1_2729	K07816	97.83	271.7	2.3e-81	GTP pyrophosphokinase [EC:2.7.6.5]
CP004065.1_2732	K08309	133.47	163.4	3e-48	peptidoglycan lytic transglycosylase [EC:4.2.2.29]
CP004065.1_2733	K06886	39.40	139.1	4.8e-41	hemoglobin
CP004065.1_2734	K07396	64.20	133.6	1.7e-39	putative protein-disulfide isomerase
CP004065.1_2736	K08602	113.03	846.6	4.6e-255	oligoendopeptidase F [EC:3.4.24.-]
CP004065.1_2737	K06198	66.63	239.8	1.4e-71	competence protein CoiA
CP004065.1_2738	K16511	72.03	243.7	5.2e-73	adapter protein MecA 1/2
CP004065.1_2740	K16509	84.43	231.9	1.5e-69	regulatory protein spx
CP004065.1_2743	K10823	502.03	538.5	5.6e-162	oligopeptide transport system ATP-binding protein
CP004065.1_2743	K02032	420.67	474.8	1.2e-142	peptide/nickel transport system ATP-binding protein
CP004065.1_2744	K15583	519.70	607.9	2.8e-183	oligopeptide transport system ATP-binding protein
CP004065.1_2744	K02031	412.13	501.0	1.1e-150	peptide/nickel transport system ATP-binding protein
CP004065.1_2745	K15582	364.50	439.9	1e-132	oligopeptide transport system permease protein
CP004065.1_2745	K02034	256.80	354.4	2.1e-106	peptide/nickel transport system permease protein
CP004065.1_2746	K15581	345.63	435.3	4.4e-131	oligopeptide transport system permease protein
CP004065.1_2746	K02033	263.63	372.2	9e-112	peptide/nickel transport system permease protein
CP004065.1_2747	K15580	368.50	611.4	5.6e-184	oligopeptide transport system substrate-binding protein
CP004065.1_2747	K02035	249.67	350.5	4.3e-105	peptide/nickel transport system substrate-binding protein
CP004065.1_2748	K01867	130.77	432.8	4.9e-130	tryptophanyl-tRNA synthetase [EC:6.1.1.2]
CP004065.1_2750	K02034	256.80	399.8	3.5e-120	peptide/nickel transport system permease protein
CP004065.1_2751	K02033	263.63	432.7	4e-130	peptide/nickel transport system permease protein
CP004065.1_2752	K02035	249.67	426.8	3.5e-128	peptide/nickel transport system substrate-binding protein
CP004065.1_2753	K02032	420.67	536.0	3.9e-161	peptide/nickel transport system ATP-binding protein
CP004065.1_2753	K12372	463.00	472.1	5.9e-142	dipeptide transport system ATP-binding protein
CP004065.1_2754	K02031	412.13	489.8	2.7e-147	peptide/nickel transport system ATP-binding protein
CP004065.1_2756	K09458	480.27	621.1	4.6e-187	3-oxoacyl-[acyl-carrier-protein] synthase II [EC:2.3.1.179]
CP004065.1_2757	K00648	229.33	452.6	3.4e-136	3-oxoacyl-[acyl-carrier-protein] synthase III [EC:2.3.1.180]

CP004065.1_2759	K02254	81.67	112.9	9.8e-34	competence protein ComZ
CP004065.1_2760	K05519	263.97	433.0	1.1e-130	transcriptional activator of comK gene
CP004065.1_2760	K07335	118.73	160.1	2e-47	basic membrane protein A and related proteins
CP004065.1_2765	K00611	353.27	519.0	2.1e-156	ornithine carbamoyltransferase [EC:2.1.3.3]
CP004065.1_2768	K00821	483.53	530.3	9.3e-160	acetylornithine/N-succinyldiaminopimelate aminotransferase [EC:2.6.1.11 2.6.1.17]
CP004065.1_2769	K00930	215.57	303.1	5.2e-91	acetylglutamate kinase [EC:2.7.2.8]
CP004065.1_2770	K00620	170.47	573.3	1.3e-172	glutamate N-acetyltransferase / amino-acid N-acetyltransferase [EC:2.3.1.35 2.3.1.1]
CP004065.1_2771	K00145	128.50	462.7	2.2e-139	N-acetyl-gamma-glutamyl-phosphate reductase [EC:1.2.1.38]
CP004065.1_2773	K16653	252.03	272.5	1.5e-81	decaprenylphospho-beta-D-ribofuranose 2-oxidase [EC:1.1.98.3]
CP004065.1_2774	K11210	123.53	243.7	1.4e-73	metallothiol transferase [EC:2.5.1.-]
CP004065.1_2777	K21064	166.97	310.7	2.4e-93	5-amino-6-(5-phospho-D-ribitylamino)uracil phosphatase [EC:3.1.3.104]
CP004065.1_2782	K25232	87.57	288.4	2e-86	fatty acid kinase fatty acid binding subunit
CP004065.1_2784	K03406	65.50	215.7	3e-64	methyl-accepting chemotaxis protein
CP004065.1_2785	K07335	118.73	317.3	4.9e-95	basic membrane protein A and related proteins
CP004065.1_2786	K00243	150.27	306.2	6.4e-92	uncharacterized protein
CP004065.1_2787	K09767	72.13	212.9	9.1e-64	cyclic-di-GMP-binding protein
CP004065.1_2788	K24042	407.17	841.7	6.1e-254	methionine synthase / methylenetetrahydrofolate reductase (NADH) [EC:2.1.1.13 1.5.1.54]
CP004065.1_2792	K16043	409.67	597.8	1e-180	scyllo-inositol 2-dehydrogenase (NAD+) [EC:1.1.1.370]
CP004065.1_2792	K00010	272.37	366.3	4.6e-110	myo-inositol 2-dehydrogenase / D-chiro-inositol 1-dehydrogenase [EC:1.1.1.18 1.1.1.369]
CP004065.1_2793	K05499	394.87	395.5	5.6e-119	LacI family transcriptional regulator, repressor for deo operon, udp, cdd, tsx, nupC, and nupG
CP004065.1_2793	K02529	268.37	391.6	1.4e-117	LacI family transcriptional regulator, galactose operon repressor
CP004065.1_2793	K03484	339.57	339.6	5e-102	LacI family transcriptional regulator, sucrose operon repressor
CP004065.1_2795	K26937	267.43	309.9	5.5e-93	MATE family, multidrug efflux pump
CP004065.1_2797	K01953	106.30	520.9	1.4e-156	asparagine synthase (glutamine-hydrolysing) [EC:6.3.5.4]
CP004065.1_2801	K06376	38.70	52.3	1e-14	stage 0 sporulation regulatory protein
CP004065.1_2802	K06299	95.63	113.2	8.3e-34	spore germination protein PA
CP004065.1_2803	K06300	53.50	98.3	1.2e-28	spore germination protein PB
CP004065.1_2804	K06301	73.93	230.0	7e-69	spore germination protein PC
CP004065.1_2805	K06302	40.33	96.5	1.1e-28	spore germination protein PD
CP004065.1_2806	K06303	95.90	142.4	1.5e-42	spore germination protein PE
CP004065.1_2807	K06304	91.83	109.7	1.3e-32	spore germination protein PF
CP004065.1_2809	K03546	183.10	469.3	7.7e-141	DNA repair protein SbcC/Rad50
CP004065.1_2810	K03547	134.00	294.5	3.4e-88	DNA repair protein SbcD/Mre11
CP004065.1_2811	K16898	483.47	1048.6	1.2e-315	ATP-dependent helicase/nuclease subunit A [EC:5.6.2.4 3.1.-.-]
CP004065.1_2812	K16899	216.03	649.6	3e-195	ATP-dependent helicase/nuclease subunit B [EC:5.6.2.4 3.1.-.-]
CP004065.1_2816	K08153	415.17	537.8	2.6e-162	MFS transporter, DHA1 family, multidrug resistance protein
CP004065.1_2817	K07120	60.57	289.5	7.5e-87	uncharacterized protein
CP004065.1_2821	K03100	114.77	145.4	4.8e-43	signal peptidase I [EC:3.4.21.89]
CP004065.1_2829	K05937	52.50	192.0	7.7e-58	uncharacterized protein
CP004065.1_2830	K05937	52.50	175.5	9.6e-53	uncharacterized protein
CP004065.1_2831	K02250	58.87	214.4	4.2e-64	competence protein ComK
CP004065.1_2835	K06595	325.93	518.2	3.9e-156	heam-based aerotactic trancducer
CP004065.1_2835	K03406	65.50	149.2	4e-44	methyl-accepting chemotaxis protein
CP004065.1_2836	K03523	54.23	155.7	2.9e-46	biotin transport system substrate-specific component
CP004065.1_2839	K15640	215.67	271.9	1.6e-81	uncharacterized phosphatase
CP004065.1_2841	K01342	376.70	468.1	7.6e-141	subtilisin [EC:3.4.21.62]
CP004065.1_2841	K14645	303.80	331.7	1.9e-99	serine protease [EC:3.4.21.-]
CP004065.1_2842	K06013	120.77	215.4	4e-64	STE24 endopeptidase [EC:3.4.24.84]
CP004065.1_2844	K01897	430.97	481.7	8.3e-145	long-chain acyl-CoA synthetase [EC:6.2.1.3]
CP004065.1_2846	K03800	254.90	357.3	4.1e-107	lipoate---protein ligase [EC:6.3.1.20]
CP004065.1_2847	K00784	70.63	86.0	4.5e-25	ribonuclease Z [EC:3.1.26.11]
CP004065.1_2849	K11102	518.13	632.4	5.1e-191	proton glutamate symport protein
CP004065.1_2850	K01269	358.40	570.2	3e-172	aminopeptidase [EC:3.4.11.-]
CP004065.1_2852	K00648	229.33	389.0	6.3e-117	3-oxoacyl-[acyl-carrier-protein] synthase III [EC:2.3.1.180]
CP004065.1_2853	K01421	140.40	679.2	2.1e-204	putative membrane protein
CP004065.1_2856	K00231	144.83	477.7	1.3e-143	protoporphyrinogen/coproporphyrinogen III oxidase [EC:1.3.3.4 1.3.3.15]

CP004065.1_2857	K01772	59.27	298.9	1.5e-89	protoporphyrin/coproporphyrin ferrochelatase [EC:4.98.1.1 4.99.1.9]
CP004065.1_2858	K01599	181.83	456.5	3.1e-137	uroporphyrinogen decarboxylase [EC:4.1.1.37]
CP004065.1_2859	K12555	770.43	878.2	1.3e-264	penicillin-binding protein 2A [EC:2.4.99.28 3.4.16.4]
CP004065.1_2859	K05366	639.07	689.0	2.4e-207	penicillin-binding protein 1A [EC:2.4.99.28 3.4.16.4]
CP004065.1_2862	K01436	522.37	632.5	4e-191	amidohydrolase [EC:3.5.1.-]
CP004065.1_2863	K25159	280.73	366.2	1.3e-110	protein EcsC
CP004065.1_2864	K25157	161.40	433.5	1.5e-130	ABC-2 type transport system permease protein
CP004065.1_2865	K25158	298.00	402.8	9e-122	ABC-2 type transport system ATP-binding protein
CP004065.1_2866	K02503	85.60	134.3	9.1e-40	histidine triad (HIT) family protein [EC:3.9.1.-]
CP004065.1_2867	K00831	137.77	519.6	1.4e-156	phosphoserine aminotransferase [EC:2.6.1.52]
CP004065.1_2871	K09682	201.47	316.7	7.8e-96	MarR family transcriptional regulator, protease production regulatory protein HPr
CP004065.1_2874	K07533	211.47	293.1	7.7e-88	foldase protein PrsA [EC:5.2.1.8]
CP004065.1_2876	K03698	131.33	428.3	7.1e-129	3'-5' exoribonuclease [EC:3.1.-.-]
CP004065.1_2878	K03547	134.00	172.5	3.7e-51	DNA repair protein SbcD/Mre11
CP004065.1_2880	K01990	262.37	268.4	2.6e-80	ABC-2 type transport system ATP-binding protein
CP004065.1_2883	K26733	125.17	178.2	8.9e-54	K+:H+ antiporter modulator KhtS
CP004065.1_2884	K07228	80.77	193.1	5.5e-58	K+:H+ antiporter subunit KhtT
CP004065.1_2885	K26732	261.93	463.1	1.6e-139	K+:H+ antiporter subunit KhtU
CP004065.1_2886	K27501	461.57	549.2	1.3e-165	putrescine N-hydroxylase [EC:1.14.13.252]
CP004065.1_2887	K02495	261.80	287.2	4.6e-86	oxygen-independent coproporphyrinogen III oxidase [EC:1.3.98.3]
CP004065.1_2888	K21064	166.97	177.8	6.3e-53	5-amino-6-(5-phospho-D-ribitylamino)uracil phosphatase [EC:3.1.3.104]
CP004065.1_2895	K06419	107.57	113.5	7.6e-34	small acid-soluble spore protein B (major beta-type SASP)
CP004065.1_2895	K06418	101.07	106.3	1.2e-31	small acid-soluble spore protein A (major alpha-type SASP)
CP004065.1_2897	K07118	124.57	167.1	1.2e-49	uncharacterized protein
CP004065.1_2898	K18890	703.17	818.1	1.5e-246	ATP-binding cassette, subfamily B, multidrug efflux pump
CP004065.1_2899	K18889	667.30	802.4	7e-242	ATP-binding cassette, subfamily B, multidrug efflux pump
CP004065.1_2904	K00824	296.37	394.9	5.3e-119	D-alanine transaminase [EC:2.6.1.21]
CP004065.1_2905	K22278	73.27	180.8	9.8e-54	peptidoglycan-N-acetylglucosamine deacetylase [EC:3.5.1.104]
CP004065.1_2906	K12410	226.47	263.3	6.5e-79	NAD-dependent protein deacetylase/lipoamidase [EC:2.3.1.286 2.3.1.313]
CP004065.1_2907	K16052	236.47	383.3	4.5e-115	MscS family membrane protein
CP004065.1_2909	K01126	152.53	231.7	2.4e-69	glycerophosphoryl diester phosphodiesterase [EC:3.1.4.46]
CP004065.1_2910	K06199	39.60	98.1	9.2e-29	fluoride exporter
CP004065.1_2911	K06199	39.60	72.1	7.2e-21	fluoride exporter
CP004065.1_2912	K03699	305.47	485.7	3.9e-146	magnesium and cobalt exporter, CNNM family
CP004065.1_2913	K00646	418.90	612.6	7.8e-185	malonyl-[acp] decarboxylase [EC:4.1.1.124]
CP004065.1_2914	K11358	327.47	562.8	1e-169	aspartate aminotransferase [EC:2.6.1.1]
CP004065.1_2915	K11923	88.47	176.5	6e-53	MerR family transcriptional regulator, copper efflux regulator
CP004065.1_2916	K03699	305.47	495.6	3.9e-149	magnesium and cobalt exporter, CNNM family
CP004065.1_2917	K00655	122.40	157.7	9.8e-47	1-acyl-sn-glycerol-3-phosphate acyltransferase [EC:2.3.1.51]
CP004065.1_2918	K03088	96.50	138.4	6.6e-41	RNA polymerase sigma-70 factor, ECF subfamily
CP004065.1_2921	K03308	387.23	493.3	1.8e-148	neurotransmitter:Na+ symporter, NSS family
CP004065.1_2922	K03294	476.00	602.6	1.7e-181	basic amino acid/polyamine antiporter, APA family
CP004065.1_2925	K19242	229.77	411.7	2.8e-124	LysR family transcriptional regulator, repressor for citA
CP004065.1_2926	K19224	198.37	311.1	2.6e-93	peptidoglycan DL-endopeptidase LytE [EC:3.4.-.-]
CP004065.1_2927	K01077	73.07	361.6	1.7e-108	alkaline phosphatase [EC:3.1.3.1]
CP004065.1_2928	K06415	220.90	652.5	2.6e-196	stage V sporulation protein R
CP004065.1_2930	K13771	131.13	229.1	9.1e-69	Rrf2 family transcriptional regulator, nitric oxide-sensitive transcriptional repressor
CP004065.1_2931	K19223	333.63	508.9	4.8e-153	peptidoglycan DL-endopeptidase LytF [EC:3.4.-.-]
CP004065.1_2931	K19224	198.37	281.8	2.1e-84	peptidoglycan DL-endopeptidase LytE [EC:3.4.-.-]
CP004065.1_2934	K03206	177.40	275.1	4e-83	azobenzene reductase [EC:1.7.1.6]
CP004065.1_2937	K01835	371.63	415.2	1.3e-124	phosphoglucomutase [EC:5.4.2.2]
CP004065.1_2938	K00111	151.87	536.2	2.2e-161	glycerol-3-phosphate dehydrogenase [EC:1.1.5.3]
CP004065.1_2939	K00864	510.13	794.7	1.3e-239	glycerol kinase [EC:2.7.1.30]
CP004065.1_2940	K02440	275.10	387.0	1.6e-116	glycerol uptake facilitator
CP004065.1_2941	K02443	84.13	264.8	9.4e-80	glycerol uptake operon antiterminator
CP004065.1_2942	K03851	556.53	725.5	4.3e-219	taurine-pyruvate aminotransferase [EC:2.6.1.77]

CP004065.1_2947	K06180	285.23	318.3	2e-95	23S rRNA pseudouridine1911/1915/1917 synthase [EC:5.4.99.23]
CP004065.1_2948	K07284	60.53	160.4	1.4e-47	sortase A [EC:3.4.22.70]
CP004065.1_2949	K01081	158.63	179.9	2.4e-53	5'-nucleotidase [EC:3.1.3.5]
CP004065.1_2952	K06345	62.77	123.0	3.3e-36	spore cortex protein
CP004065.1_2954	K06956	334.43	599.0	9e-181	uncharacterized protein
CP004065.1_2955	K18967	236.10	323.4	5.4e-97	diguanylate cyclase [EC:2.7.7.65]
CP004065.1_2956	K02073	57.57	227.3	8.3e-68	D-methionine transport system substrate-binding protein
CP004065.1_2957	K03704	80.73	115.3	6e-34	cold shock protein
CP004065.1_2958	K01992	16.23	24.0	2e-06	ABC-2 type transport system permease protein
CP004065.1_2959	K01990	262.37	304.3	3.3e-91	ABC-2 type transport system ATP-binding protein
CP004065.1_2961	K07979	106.93	120.2	1.6e-35	GntR family transcriptional regulator
CP004065.1_2964	K03809	60.03	163.0	2.2e-48	NAD(P)H dehydrogenase (quinone) [EC:1.6.5.2]
CP004065.1_2965	K03446	476.67	629.6	1.1e-189	MFS transporter, DHA2 family, multidrug resistance protein
CP004065.1_2968	K09786	182.07	543.9	7.5e-164	uncharacterized protein
CP004065.1_2969	K07180	288.33	905.1	5.6e-273	serine protein kinase
CP004065.1_2974	K03216	93.87	253.6	3.8e-76	tRNA (cytidine/uridine-2'-O-)-methyltransferase [EC:2.1.1.207]
CP004065.1_2976	K18979	73.50	418.2	1.2e-125	epoxyqueuosine reductase [EC:1.17.99.6]
CP004065.1_2978	K07729	60.67	91.9	8.1e-27	putative transcriptional regulator
CP004065.1_2980	K02954	26.53	130.4	1.5e-38	small subunit ribosomal protein S14
CP004065.1_2981	K04091	325.40	608.7	3e-183	alkanesulfonate monooxygenase [EC:1.14.14.5 1.14.14.34]
CP004065.1_2982	K15554	248.37	327.2	1.8e-98	sulfonate transport system permease protein
CP004065.1_2982	K02050	145.27	251.3	4.3e-75	NitT/TauT family transport system permease protein
CP004065.1_2983	K15553	187.27	294.3	5.3e-88	sulfonate transport system substrate-binding protein
CP004065.1_2983	K02051	157.20	191.1	9.7e-57	NitT/TauT family transport system substrate-binding protein
CP004065.1_2984	K15555	333.60	339.1	6.5e-102	sulfonate transport system ATP-binding protein [EC:7.6.2.14]
CP004065.1_2985	K07045	129.87	155.2	5.1e-46	uncharacterized protein
CP004065.1_2986	K03781	68.70	849.1	6.8e-256	catalase [EC:1.11.1.6]
CP004065.1_2987	K02032	420.67	502.0	7.3e-151	peptide/nickel transport system ATP-binding protein
CP004065.1_2988	K02031	412.13	496.7	2.2e-149	peptide/nickel transport system ATP-binding protein
CP004065.1_2989	K02034	256.80	347.7	2.1e-104	peptide/nickel transport system permease protein
CP004065.1_2990	K02033	263.63	389.9	3.8e-117	peptide/nickel transport system permease protein
CP004065.1_2991	K02035	249.67	353.7	4.6e-106	peptide/nickel transport system substrate-binding protein
CP004065.1_2994	K03147	598.13	1240.9	0	phosphomethylpyrimidine synthase [EC:4.1.99.17]
CP004065.1_2995	K05995	93.83	159.8	2.3e-47	dipeptidase E [EC:3.4.13.21]
CP004065.1_3000	K09825	159.17	213.7	4e-64	Fur family transcriptional regulator, peroxide stress response regulator
CP004065.1_3001	K03564	157.77	232.4	1.7e-69	thioredoxin-dependent peroxiredoxin [EC:1.11.1.24]
CP004065.1_3002	K01845	312.23	624.0	7.8e-188	glutamate-1-semialdehyde 2,1-aminomutase [EC:5.4.3.8]
CP004065.1_3004	K24708	756.00	919.0	2.1e-277	ATP-binding cassette, subfamily B, putative efflux pump [EC:7.6.2.-]
CP004065.1_3004	K11085	715.60	722.2	1.6e-217	ATP-binding cassette, subfamily B, bacterial MsbA [EC:7.5.2.6]
CP004065.1_3004	K06147	612.93	683.1	1.1e-205	ATP-binding cassette, subfamily B, bacterial
CP004065.1_3005	K07586	69.50	235.3	1.6e-70	uncharacterized protein
CP004065.1_3007	K06422	28.00	74.6	1.6e-21	small acid-soluble spore protein E (minor gamma-type SASP)
CP004065.1_3008	K10780	265.13	368.8	3.3e-111	enoyl-[acyl-carrier protein] reductase III [EC:1.3.1.104]
CP004065.1_3010	K03575	149.73	439.2	4.2e-132	A/G-specific adenine glycosylase [EC:3.2.2.31]
CP004065.1_3011	K07038	27.13	100.8	1.5e-29	inner membrane protein
CP004065.1_3012	K25619	344.20	968.9	6.9e-292	lipoteichoic acid glycosylation protein
CP004065.1_3013	K25617	482.30	529.9	3.3e-160	lipoteichoic acid polyisoprenyl-phosphate beta-N-acetylglucosaminyltransferase [EC:2.4.-.-]
CP004065.1_3013	K20534	338.93	440.9	1.2e-132	polyisoprenyl-phosphate glycosyltransferase [EC:2.4.-.-]
CP004065.1_3014	K22369	259.10	281.8	2.3e-84	epoxide hydrolase 4 [EC:3.3.-.-]
CP004065.1_3015	K26996	27.07	64.7	2.1e-18	immunity protein, SdpI family
CP004065.1_3018	K01318	117.10	280.6	4.7e-84	glutamyl endopeptidase [EC:3.4.21.19]
CP004065.1_3022	K03565	36.33	136.9	2.1e-40	regulatory protein
CP004065.1_3023	K07071	116.47	381.3	2.3e-114	uncharacterized protein
CP004065.1_3027	K06998	103.40	218.6	2.8e-65	trans-2,3-dihydro-3-hydroxyanthranilate isomerase [EC:5.3.3.17]
CP004065.1_3028	K14205	388.70	719.3	1.3e-216	phosphatidylglycerol lysyltransferase [EC:2.3.2.3]
CP004065.1_3032	K03623	37.70	102.5	2.1e-30	ribonuclease inhibitor

CP004065.1_3033	K22579	503.63	551.9	8.3e-166	rifampicin phosphotransferase [EC:2.7.9.6]
CP004065.1_3035	K00355	115.87	166.3	2.8e-49	NAD(P)H dehydrogenase (quinone) [EC:1.6.5.2]
CP004065.1_3036	K26739	181.10	256.2	3.6e-77	PadR family transcriptional regulator, phenolic acid-responsive transcriptional regulator
CP004065.1_3037	K02257	126.90	267.2	6e-80	heme o synthase [EC:2.5.1.141]
CP004065.1_3038	K07104	96.57	369.2	6.2e-111	catechol 2,3-dioxygenase [EC:1.13.11.2]
CP004065.1_3039	K15977	60.27	107.7	2.1e-31	putative oxidoreductase
CP004065.1_3040	K18888	717.83	872.2	7.2e-263	ATP-binding cassette, subfamily B, multidrug efflux pump
CP004065.1_3040	K18890	703.17	708.6	2.1e-213	ATP-binding cassette, subfamily B, multidrug efflux pump
CP004065.1_3040	K06147	612.93	647.5	6.7e-195	ATP-binding cassette, subfamily B, bacterial
CP004065.1_3041	K18887	708.60	760.4	4.6e-229	ATP-binding cassette, subfamily B, multidrug efflux pump
CP004065.1_3042	K02750	597.40	912.5	2.9e-275	alpha-glucoside PTS system EIICB component [EC:2.7.1.208 2.7.1.-]
CP004065.1_3043	K03481	148.27	352.8	1.9e-106	RpiR family transcriptional regulator, glv operon transcriptional regulator
CP004065.1_3044	K01232	474.00	787.7	5.8e-238	maltose-6'-phosphate glucosidase [EC:3.2.1.122]
CP004065.1_3045	K06425	45.60	81.9	5.1e-24	small acid-soluble spore protein H (minor)
CP004065.1_3046	K21405	471.50	585.8	3.3e-176	sigma-54 dependent transcriptional regulator, acetoin dehydrogenase operon transcriptional activator AcoR
CP004065.1_3047	K00382	465.60	557.8	5.7e-168	dihydrolipoyl dehydrogenase [EC:1.8.1.4]
CP004065.1_3049	K21417	462.07	487.9	2.5e-147	acetoin:2,6-dichlorophenolindophenol oxidoreductase subunit beta [EC:1.1.1.-]
CP004065.1_3050	K21416	442.50	509.9	7.3e-154	acetoin:2,6-dichlorophenolindophenol oxidoreductase subunit alpha [EC:1.1.1.-]
CP004065.1_3054	K03215	301.40	562.6	2.7e-169	23S rRNA (uracil1939-C5)-methyltransferase [EC:2.1.1.190]
CP004065.1_3055	K01247	173.13	217.4	7.1e-65	DNA-3-methyladenine glycosylase II [EC:3.2.2.21]
CP004065.1_3056	K03284	165.70	334.7	2.2e-100	magnesium transporter
CP004065.1_3059	K01567	253.73	373.3	1.7e-112	peptidoglycan-N-acetylmuramic acid deacetylase [EC:3.5.1.-]
CP004065.1_3059	K22278	73.27	212.3	2.8e-63	peptidoglycan-N-acetylglucosamine deacetylase [EC:3.5.1.104]
CP004065.1_3062	K22044	182.20	194.1	8.9e-58	moderate conductance mechanosensitive channel
CP004065.1_3064	K07300	76.70	410.5	2.3e-123	Ca2+:H+ antiporter
CP004065.1_3067	K07058	40.93	235.9	2.6e-70	membrane protein
CP004065.1_3069	K25307	127.47	196.2	1.5e-58	low molecular weight protein-tyrosine phosphatase [EC:3.1.3.48]
CP004065.1_3071	K26745	212.40	496.0	1.3e-149	MFS transporter, AAHS family, multidrug transporter
CP004065.1_3072	K03785	182.93	299.0	1.7e-89	3-dehydroquinate dehydratase I [EC:4.2.1.10]
CP004065.1_3074	K02529	268.37	335.2	1.7e-100	LacI family transcriptional regulator, galactose operon repressor
CP004065.1_3075	K05520	175.93	254.8	2.5e-76	deglycase [EC:3.5.1.124]
CP004065.1_3076	K08693	794.23	1281.3	0	2',3'-cyclic-nucleotide 2'-phosphodiesterase / 3'-nucleotidase / 5'-nucleotidase [EC:3.1.4.16 3.1.3.6 3.1.3.5]
CP004065.1_3076	K01119	358.37	841.8	1.1e-253	2',3'-cyclic-nucleotide 2'-phosphodiesterase / 3'-nucleotidase [EC:3.1.4.16 3.1.3.6]
CP004065.1_3076	K11751	484.37	540.4	1.3e-162	5'-nucleotidase / UDP-sugar diphosphatase [EC:3.1.3.5 3.6.1.45]
CP004065.1_3076	K01081	158.63	176.6	2.4e-52	5'-nucleotidase [EC:3.1.3.5]
CP004065.1_3080	K03486	214.00	359.6	1.7e-108	GntR family transcriptional regulator, trehalose operon transcriptional repressor
CP004065.1_3081	K01226	799.77	891.4	5.5e-269	trehalose-6-phosphate hydrolase [EC:3.2.1.93]
CP004065.1_3082	K02819	628.60	726.6	4e-219	trehalose PTS system EIIBC or EIIBCA component [EC:2.7.1.201]
CP004065.1_3087	K19005	299.10	808.3	2.2e-243	lipoteichoic acid synthase [EC:2.7.8.20]
CP004065.1_3089	K02804	644.17	706.1	6.8e-213	N-acetylglucosamine PTS system EIICBA or EIICB component [EC:2.7.1.193]
CP004065.1_3090	K01265	141.83	220.8	6.3e-66	methionyl aminopeptidase [EC:3.4.11.18]
CP004065.1_3095	K01512	72.83	96.9	3.5e-28	acylphosphatase [EC:3.6.1.7]
CP004065.1_3096	K00491	521.20	600.2	5.7e-181	nitric-oxide synthase, bacterial [EC:1.14.14.47]
CP004065.1_3098	K03300	129.87	603.6	4.8e-182	citrate-Mg2+:H+ or citrate-Ca2+:H+ symporter, CitMHS family
CP004065.1_3099	K07795	76.40	263.1	1.1e-78	putative tricarboxylic transport membrane protein
CP004065.1_3100	K11638	284.93	357.7	5.4e-108	two-component system, CitB family, response regulator CitT
CP004065.1_3101	K11637	628.00	740.2	2.3e-223	two-component system, CitB family, sensor histidine kinase CitS [EC:2.7.13.3]
CP004065.1_3102	K07284	60.53	110.5	2e-32	sortase A [EC:3.4.22.70]
CP004065.1_3104	K02477	121.47	147.3	1.4e-43	two-component system, LytTR family, response regulator
CP004065.1_3105	K19694	625.00	966.6	3.9e-291	two-component system, sensor histidine kinase ChiS
CP004065.1_3110	K03319	476.77	729.1	6.1e-220	divalent anion:Na+ symporter, DASS family
CP004065.1_3111	K01728	168.73	290.8	4.9e-87	pectate lyase [EC:4.2.2.2]
CP004065.1_3114	K00359	464.70	566.9	2.6e-171	NADPH:quinone reductase [EC:1.6.5.5]
CP004065.1_3118	K08221	232.17	512.7	1.5e-154	MFS transporter, ACDE family, multidrug resistance protein
CP004065.1_3119	K21902	113.00	211.2	3.7e-63	MerR family transcriptional regulator, repressor of the yfmOP operon
CP004065.1_3120	K15738	622.23	772.1	1e-232	ABC transport system ATP-binding/permease protein

CP004065.1_3123	K03406	65.50	110.9	1.6e-32	methyl-accepting chemotaxis protein
CP004065.1_3124	K21802	740.83	745.9	4.6e-225	vanillin dehydrogenase [EC:1.2.1.67]
CP004065.1_3125	K03294	476.00	581.4	4.6e-175	basic amino acid/polyamine antiporter, APA family
CP004065.1_3126	K08223	154.93	461.0	8.6e-139	MFS transporter, FSR family, fosmidomycin resistance protein
CP004065.1_3130	K01709	276.80	320.2	4e-96	CDP-glucose 4,6-dehydratase [EC:4.2.1.45]
CP004065.1_3131	K00978	156.63	348.7	4.1e-105	glucose-1-phosphate cytidylyltransferase [EC:2.7.7.33]
CP004065.1_3132	K19005	299.10	807.7	3.2e-243	lipoteichoic acid synthase [EC:2.7.8.20]
CP004065.1_3133	K14338	601.20	1466.1	0	cytochrome P450 / NADPH-cytochrome P450 reductase [EC:1.14.14.1 1.6.2.4]
CP004065.1_3136	K15974	111.57	119.8	2.8e-35	MarR family transcriptional regulator, negative regulator of the multidrug operon emrRAB
CP004065.1_3140	K17763	101.07	210.8	8.9e-63	rsbT co-antagonist protein RsbR
CP004065.1_3142	K07145	77.87	130.9	5.2e-39	heme oxygenase (staphylobilin-producing) [EC:1.14.99.48]
CP004065.1_3146	K06334	183.93	350.1	5.5e-106	spore coat protein JC
CP004065.1_3147	K06333	33.97	121.6	5e-36	spore coat protein JB
CP004065.1_3148	K06332	36.80	111.7	8.5e-33	spore coat protein JA
CP004065.1_3149	K05593	117.83	373.3	4.3e-112	aminoglycoside 6-adenylyltransferase [EC:2.7.7.-]
CP004065.1_3151	K18189	91.27	183.0	1.6e-54	translational activator of cytochrome c oxidase 1
CP004065.1_3155	K11051	170.13	314.0	1.8e-94	multidrug/hemolysin transport system permease protein
CP004065.1_3156	K11050	344.47	412.3	2.9e-124	multidrug/hemolysin transport system ATP-binding protein
CP004065.1_3156	K01990	262.37	273.0	1.1e-81	ABC-2 type transport system ATP-binding protein
CP004065.1_3158	K03215	301.40	680.8	4e-205	23S rRNA (uracil1939-C5)-methyltransferase [EC:2.1.1.190]
CP004065.1_3159	K07029	147.20	308.3	1.5e-92	diacylglycerol kinase (ATP) [EC:2.7.1.107]
CP004065.1_3160	K03296	1115.73	1156.1	0	hydrophobic/amphiphilic exporter-1 (mainly G- bacteria), HAE1 family
CP004065.1_3162	K02434	415.47	717.8	2.3e-216	aspartyl-tRNA(Asn)/glutamyl-tRNA(Gln) amidotransferase subunit B [EC:6.3.5.6 6.3.5.7]
CP004065.1_3163	K02433	450.63	709.0	1.6e-213	aspartyl-tRNA(Asn)/glutamyl-tRNA(Gln) amidotransferase subunit A [EC:6.3.5.6 6.3.5.7]
CP004065.1_3163	K01426	303.90	412.0	1.4e-123	amidase [EC:3.5.1.4]
CP004065.1_3164	K02435	35.93	104.4	1.2e-30	aspartyl-tRNA(Asn)/glutamyl-tRNA(Gln) amidotransferase subunit C [EC:6.3.5.6 6.3.5.7]
CP004065.1_3165	K11928	556.47	693.1	4.8e-209	sodium/proline symporter
CP004065.1_3165	K03307	271.33	347.2	4e-104	solute:Na+ symporter, SSS family
CP004065.1_3166	K07507	42.27	193.5	1.2e-57	putative Mg2+ transporter-C (MgtC) family protein
CP004065.1_3167	K23983	164.10	433.3	1e-130	amicoumacin kinase [EC:2.7.1.230]
CP004065.1_3169	K01972	119.00	1002.9	2.2e-302	DNA ligase (NAD+) [EC:6.5.1.2]
CP004065.1_3170	K03657	367.57	790.4	4.8e-238	ATP-dependent DNA helicase UvrD/PcrA [EC:5.6.2.4]
CP004065.1_3171	K07094	264.60	355.5	2.5e-107	heptaprenylglyceryl phosphate synthase [EC:2.5.1.-]
CP004065.1_3175	K01486	325.67	476.7	2.1e-143	adenine deaminase [EC:3.5.4.2]
CP004065.1_3177	K01945	149.87	658.3	2.2e-198	phosphoribosylamine---glycine ligase [EC:6.3.4.13]
CP004065.1_3178	K00602	115.70	815.6	5.8e-246	phosphoribosylaminoimidazolecarboxamide formyltransferase / IMP cyclohydrolase [EC:2.1.2.3 3.5.4.10]
CP004065.1_3179	K11175	195.63	271.0	3.3e-81	phosphoribosylglycinamide formyltransferase 1 [EC:2.1.2.2]
CP004065.1_3180	K01933	170.70	570.5	9.6e-172	phosphoribosylformylglycinamidine cyclo-ligase [EC:6.3.3.1]
CP004065.1_3181	K00764	178.17	706.5	6.3e-213	amidophosphoribosyltransferase [EC:2.4.2.14]
CP004065.1_3182	K23269	711.03	1181.1	0	phosphoribosylformylglycinamidine synthase subunit PurL [EC:6.3.5.3]
CP004065.1_3183	K23265	206.17	355.7	5.4e-107	phosphoribosylformylglycinamidine synthase subunit PurQ / glutaminase [EC:6.3.5.3 3.5.1.2]
CP004065.1_3184	K23264	41.33	123.8	1.6e-36	phosphoribosylformylglycinamidine synthase subunit PurS [EC:6.3.5.3]
CP004065.1_3185	K01923	194.90	255.5	2.2e-76	phosphoribosylaminoimidazole-succinocarboxamide synthase [EC:6.3.2.6]
CP004065.1_3186	K01756	328.93	526.1	4e-158	adenylosuccinate lyase [EC:4.3.2.2]
CP004065.1_3187	K01589	271.73	525.1	4.3e-158	5-(carboxyamino)imidazole ribonucleotide synthase [EC:6.3.4.18]
CP004065.1_3188	K01588	108.13	274.1	2.1e-82	5-(carboxyamino)imidazole ribonucleotide mutase [EC:5.4.99.18]
CP004065.1_3193	K06901	93.90	528.2	4.8e-159	adenine/guanine/hypoxanthine permease
CP004065.1_3195	K03088	96.50	111.9	7.4e-33	RNA polymerase sigma-70 factor, ECF subfamily
CP004065.1_3196	K01951	100.47	792.3	1.2e-238	GMP synthase (glutamine-hydrolysing) [EC:6.3.5.2]
CP004065.1_3197	K22452	104.63	276.4	1.4e-82	protein-glutamine gamma-glutamyltransferase [EC:2.3.2.13]
CP004065.1_3199	K03924	113.70	459.1	4.4e-138	MoxR-like ATPase [EC:3.6.3.-]
CP004065.1_3200	K11735	615.90	716.0	4.6e-216	GABA permease
CP004065.1_3201	K06324	547.93	808.1	1.2e-243	spore coat protein A, manganese oxidase [EC:1.16.3.3]
CP004065.1_3206	K00004	375.63	451.9	3.5e-136	(R,R)-butanediol dehydrogenase / meso-butanediol dehydrogenase / diacetyl reductase [EC:1.1.1.4 1.1.1.- 1.1.1.303]
CP004065.1_3207	K03088	96.50	102.6	5.2e-30	RNA polymerase sigma-70 factor, ECF subfamily
CP004065.1_3209	K16568	159.77	174.5	7.4e-52	exopolysaccharide production protein ExoZ

CP004065.1_3211	K06872	48.37	73.3	4e-21	uncharacterized protein
CP004065.1_3213	K03969	84.50	207.8	7.3e-62	phage shock protein A
CP004065.1_3215	K00847	190.50	271.4	3.6e-81	fructokinase [EC:2.7.1.4]
CP004065.1_3216	K16248	464.93	732.9	2.9e-221	probable glucitol transport protein GutA
CP004065.1_3216	K03292	344.60	446.2	2.8e-134	glycoside/pentoside/hexuronide:cation symporter, GPH family
CP004065.1_3217	K00008	408.47	470.1	1.3e-141	L-iditol 2-dehydrogenase [EC:1.1.1.14]
CP004065.1_3218	K16247	183.97	864.7	1.3e-260	LuxR family transcriptional regulator, glucitol operon activator
CP004065.1_3223	K04077	136.23	878.0	8e-265	chaperonin GroEL [EC:5.6.1.7]
CP004065.1_3224	K04078	31.27	148.1	5.6e-44	chaperonin GroES
CP004065.1_3225	K07052	31.30	74.9	1.2e-21	CAAX protease family protein
CP004065.1_3227	K03118	86.40	272.7	1.2e-81	sec-independent protein translocase protein TatC
CP004065.1_3228	K03116	54.03	66.8	5.3e-19	sec-independent protein translocase protein TatA
CP004065.1_3229	K01926	112.17	283.2	3.7e-85	redox-sensing transcriptional repressor
CP004065.1_3230	K03637	63.27	259.5	1.2e-77	cyclic pyranopterin monophosphate synthase [EC:4.6.1.17]
CP004065.1_3231	K06158	558.03	676.1	1.5e-203	ATP-binding cassette, subfamily F, member 3
CP004065.1_3232	K25706	413.33	519.2	2.7e-156	tRNA N6-adenosine threonylcarbamoyltransferase [EC:2.3.1.234]
CP004065.1_3233	K03789	102.70	176.4	1.5e-52	[ribosomal protein S18]-alanine N-acetyltransferase [EC:2.3.1.266]
CP004065.1_3234	K14742	83.37	199.5	2e-59	tRNA threonylcarbamoyladenosine biosynthesis protein TsaB
CP004065.1_3235	K06925	60.83	190.8	9e-57	tRNA threonylcarbamoyladenosine biosynthesis protein TsaE
CP004065.1_3236	K00946	141.30	366.0	8.6e-110	thiamine-monophosphate kinase [EC:2.7.4.16]
CP004065.1_3238	K06609	469.27	704.7	1.1e-212	MFS transporter, SP family, major inositol transporter
CP004065.1_3239	K19745	190.73	497.3	4.5e-150	acrylyl-CoA reductase (NADPH) [EC:1.3.1.-]
CP004065.1_3241	K07217	211.70	287.2	3.9e-86	manganese catalase [EC:1.11.1.6]
CP004065.1_3246	K03449	127.83	470.3	1.8e-141	MFS transporter, CP family, cyanate transporter
CP004065.1_3248	K03492	234.97	339.5	1.9e-102	GntR family transcriptional regulator, regulator of glucomannan utilization
CP004065.1_3248	K03710	178.10	181.2	8.8e-54	GntR family transcriptional regulator
CP004065.1_3249	K18567	389.93	494.0	5.1e-149	MFS transporter, DHA1 family, purine base/nucleoside efflux pump
CP004065.1_3253	K01286	175.57	196.9	1.6e-58	D-alanyl-D-alanine carboxypeptidase [EC:3.4.16.4]
CP004065.1_3258	K07246	481.40	601.2	2.1e-181	tartrate dehydrogenase/decarboxylase / D-malate dehydrogenase [EC:1.1.1.93 4.1.1.73 1.1.1.83]
CP004065.1_3259	K01561	202.30	321.8	1.2e-96	haloacetate dehalogenase [EC:3.8.1.3]
CP004065.1_3262	K19575	133.80	140.4	2.1e-41	MerR family transcriptional regulator, activator of bmr gene
CP004065.1_3264	K07090	37.03	126.5	3e-37	uncharacterized protein
CP004065.1_3266	K09458	480.27	564.2	8.1e-170	3-oxoacyl-[acyl-carrier-protein] synthase II [EC:2.3.1.179]
CP004065.1_3267	K07552	308.70	468.4	6.2e-141	MFS transporter, DHA1 family, multidrug resistance protein
CP004065.1_3268	K00005	325.77	516.1	9.5e-156	glycerol dehydrogenase [EC:1.1.1.6]
CP004065.1_3270	K02435	35.93	42.2	1e-11	aspartyl-tRNA(Asn)/glutamyl-tRNA(Gln) amidotransferase subunit C [EC:6.3.5.6 6.3.5.7]
CP004065.1_3271	K18231	444.33	621.3	2.7e-187	macrolide transport system ATP-binding/permease protein
CP004065.1_3272	K07010	123.83	274.8	2.4e-82	putative glutamine amidotransferase
CP004065.1_3273	K11625	900.13	1121.8	0	membrane protein YdfJ
CP004065.1_3273	K06994	260.33	771.6	1.9e-232	putative drug exporter of the RND superfamily
CP004065.1_3274	K11624	235.50	312.7	2.2e-94	two-component system, NarL family, response regulator YdfI
CP004065.1_3275	K11623	240.53	517.1	6.1e-156	two-component system, NarL family, sensor histidine kinase YdfH [EC:2.7.13.3]
CP004065.1_3276	K06440	60.37	116.5	8e-35	similar to spore coat protein
CP004065.1_3277	K06439	91.47	103.8	9.3e-31	similar to spore coat protein
CP004065.1_3279	K06440	60.37	85.7	3.3e-25	similar to spore coat protein
CP004065.1_3280	K06439	91.47	143.6	3.6e-43	similar to spore coat protein
CP004065.1_3284	K07734	100.87	221.7	3e-66	transcriptional regulator
CP004065.1_3285	K00375	343.30	474.0	1.5e-142	GntR family transcriptional regulator / MocR family aminotransferase
CP004065.1_3287	K21745	110.70	179.6	6.2e-54	MerR family transcriptional regulator, aldehyde-responsive regulator
CP004065.1_3288	K13979	530.93	592.9	6.4e-179	alcohol dehydrogenase (NADP+) [EC:1.1.1.2]
CP004065.1_3289	K03325	113.10	216.8	1.1e-64	arsenite transporter
CP004065.1_3291	K15632	431.50	602.6	4.8e-182	23S rRNA (adenine-C8)-methyltransferase [EC:2.1.1.224]
CP004065.1_3291	K06941	65.87	347.5	3.2e-104	23S rRNA (adenine2503-C2)-methyltransferase [EC:2.1.1.192]
CP004065.1_3297	K07222	277.37	363.5	3.2e-109	putative flavoprotein involved in K+ transport
CP004065.1_3298	K16264	312.40	432.6	4.5e-130	cobalt-zinc-cadmium efflux system protein
CP004065.1_3306	K06016	482.23	587.8	4.2e-177	beta-ureidopropionase / N-carbamoyl-L-amino-acid hydrolase [EC:3.5.1.6 3.5.1.87]

CP004065.1_3309	K03837	524.27	561.0	2.8e-169	serine transporter
CP004065.1_3310	K09022	107.97	183.6	1.3e-54	2-iminobutanoate/2-iminopropanoate deaminase [EC:3.5.99.10]
CP004065.1_3312	K01751	246.53	453.7	1.7e-136	diaminopropionate ammonia-lyase [EC:4.3.1.15]
CP004065.1_3314	K25519	418.90	711.0	3.1e-215	putative sporulation hydrolase
CP004065.1_3317	K01779	86.67	193.7	1.2e-57	aspartate racemase [EC:5.1.1.13]
CP004065.1_3318	K07090	37.03	121.4	1e-35	uncharacterized protein
CP004065.1_3319	K04085	48.00	100.3	1.6e-29	tRNA 2-thiouridine synthesizing protein A [EC:2.8.1.-]
CP004065.1_3321	K03972	95.13	100.5	2.1e-29	phage shock protein E
CP004065.1_3321	K04085	48.00	94.4	1e-27	tRNA 2-thiouridine synthesizing protein A [EC:2.8.1.-]
CP004065.1_3324	K03704	80.73	120.4	1.6e-35	cold shock protein
CP004065.1_3325	K07118	124.57	251.4	2.5e-75	uncharacterized protein
CP004065.1_3330	K23775	99.10	176.2	2.5e-52	MarR family transcriptional regulator, organic hydroperoxide resistance regulator
CP004065.1_3332	K04063	57.63	170.7	9.4e-51	lipoyl-dependent peroxiredoxin [EC:1.11.1.28]
CP004065.1_3334	K03762	459.97	590.7	8e-178	MFS transporter, MHS family, proline/betaine transporter
CP004065.1_3336	K21487	904.13	943.5	1.1e-284	toxin YobL [EC:3.1.-.-]
CP004065.1_3337	K21488	126.47	232.8	2.8e-70	antitoxin YobK
CP004065.1_3340	K06357	69.33	98.8	2.5e-29	phosphatase RapI regulator
CP004065.1_3345	K03095	90.87	151.6	3.5e-45	SprT-like protein
CP004065.1_3347	K06959	437.10	1079.1	0	protein Tex
CP004065.1_3348	K05518	113.90	219.9	4.5e-66	phosphoserine phosphatase RsbX [EC:3.1.3.3]
CP004065.1_3349	K03090	268.20	316.8	6e-95	RNA polymerase sigma-B factor
CP004065.1_3350	K04757	53.50	136.9	1.9e-40	serine/threonine-protein kinase RsbW [EC:2.7.11.1]
CP004065.1_3351	K04749	63.93	78.9	1.2e-22	anti-sigma B factor antagonist
CP004065.1_3352	K07315	145.67	251.5	3.5e-75	phosphoserine phosphatase RsbU/P [EC:3.1.3.3]
CP004065.1_3353	K17752	96.77	203.6	5.3e-61	serine/threonine-protein kinase RsbT [EC:2.7.11.1]
CP004065.1_3354	K17762	98.20	174.3	2.4e-52	rsbT antagonist protein RsbS
CP004065.1_3355	K17763	101.07	242.8	1.7e-72	rsbT co-antagonist protein RsbR
CP004065.1_3356	K07171	19.10	147.3	1.6e-43	mRNA interferase MazF [EC:3.1.-.-]
CP004065.1_3357	K07723	41.50	102.3	3.4e-30	CopG family transcriptional regulator / antitoxin EndoAI
CP004065.1_3358	K01775	106.13	437.5	1.1e-131	alanine racemase [EC:5.1.1.1]
CP004065.1_3360	K00997	102.37	143.4	2.4e-42	holo-[acyl-carrier protein] synthase [EC:2.7.8.7]
CP004065.1_3362	K08981	123.43	326.3	7.7e-98	putative membrane protein
CP004065.1_3363	K09167	107.60	165.7	3.4e-49	uncharacterized protein
CP004065.1_3364	K05592	531.43	650.1	7.7e-196	ATP-dependent RNA helicase DeaD [EC:5.6.2.7]
CP004065.1_3365	K01929	327.77	540.6	7.9e-163	UDP-N-acetylmuramoyl-tripeptide--D-alanyl-D-alanine ligase [EC:6.3.2.10]
CP004065.1_3366	K01921	226.63	464.3	1.6e-139	D-alanine-D-alanine ligase [EC:6.3.2.4]
CP004065.1_3368	K13283	187.13	188.4	3.3e-56	ferrous-iron efflux pump FieF
CP004065.1_3374	K01990	262.37	294.2	3.8e-88	ABC-2 type transport system ATP-binding protein
CP004065.1_3376	K11103	521.30	683.1	7.7e-206	aerobic C4-dicarboxylate transport protein
CP004065.1_3377	K11692	272.83	324.0	9.8e-98	two-component system, CitB family, response regulator DctR
CP004065.1_3378	K11691	621.43	837.7	6.8e-253	two-component system, CitB family, sensor histidine kinase DctS [EC:2.7.13.3]
CP004065.1_3379	K11688	349.97	372.3	1e-111	C4-dicarboxylate-binding protein DctP
CP004065.1_3379	K21395	93.57	269.4	1.5e-80	TRAP-type transport system periplasmic protein
CP004065.1_3380	K07217	211.70	394.9	7.3e-119	manganese catalase [EC:1.11.1.6]
CP004065.1_3383	K06884	16.83	129.1	5.1e-38	uncharacterized protein
CP004065.1_3385	K03297	108.13	132.8	3.7e-39	small multidrug resistance pump
CP004065.1_3390	K03322	514.07	570.5	6.4e-172	manganese transport protein
CP004065.1_3396	K00158	626.93	796.7	2e-240	pyruvate oxidase [EC:1.2.3.3]
CP004065.1_3397	K03574	70.57	75.2	1.1e-21	8-oxo-dGTP diphosphatase [EC:3.6.1.55]
CP004065.1_3400	K11936	261.37	293.6	5.2e-88	poly-beta-1,6-N-acetyl-D-glucosamine synthase [EC:2.4.1.-]
CP004065.1_3400	K19003	255.63	269.2	1.4e-80	1,2-diacylglycerol 3-beta-glucosyltransferase [EC:2.4.1.336]
CP004065.1_3404	K03169	533.50	846.5	4.9e-255	DNA topoisomerase III [EC:5.6.2.1]
CP004065.1_3405	K03719	131.40	149.1	3.2e-44	Lrp/AsnC family transcriptional regulator, leucine-responsive regulatory protein
CP004065.1_3409	K03817	131.60	219.3	1.7e-65	ribosomal-protein-serine acetyltransferase [EC:2.3.1.-]
CP004065.1_3409	K02435	35.93	37.6	2.7e-10	aspartyl-tRNA(Asn)/glutamyl-tRNA(Gln) amidotransferase subunit C [EC:6.3.5.6 6.3.5.7]
CP004065.1_3410	K09988	88.53	204.6	3.3e-61	D-lyxose ketol-isomerase [EC:5.3.1.15]

CP004065.1_3413	K03483	467.10	675.0	3.2e-203	mannitol operon transcriptional activator
CP004065.1_3414	K26057	314.60	320.6	1.8e-96	6-dehydroglucose reductase [EC:1.1.1.432]
CP004065.1_3415	K21467	750.03	910.4	1.2e-274	penicillin-binding protein 3
CP004065.1_3420	K06350	399.40	467.1	1.7e-140	antagonist of KipI
CP004065.1_3420	K23124	341.57	391.9	5.6e-118	5-oxoprolinase (ATP-hydrolysing) subunit C [EC:3.5.2.9]
CP004065.1_3421	K06351	275.63	353.5	1.8e-106	inhibitor of KinA
CP004065.1_3422	K22210	102.40	278.2	2.8e-83	D-glutamate cyclase [EC:4.2.1.48]
CP004065.1_3424	K07160	94.40	411.5	1.3e-123	5-oxoprolinase (ATP-hydrolysing) subunit A [EC:3.5.2.9]
CP004065.1_3425	K21064	166.97	235.0	2.5e-70	5-amino-6-(5-phospho-D-ribitylamino)uracil phosphatase [EC:3.1.3.104]
CP004065.1_3427	K02372	79.93	115.3	1.1e-33	3-hydroxyacyl-[acyl-carrier-protein] dehydratase [EC:4.2.1.59]
CP004065.1_3428	K00009	152.20	501.0	1.2e-150	mannitol-1-phosphate 5-dehydrogenase [EC:1.1.1.17]
CP004065.1_3429	K02798	165.87	197.4	1.8e-59	mannitol PTS system EIIA component [EC:2.7.1.197]
CP004065.1_3430	K02800	164.20	853.0	2.8e-257	mannitol PTS system EIICBA or EIICB component [EC:2.7.1.197]
CP004065.1_3432	K21601	138.37	293.9	1.1e-88	DeoR family transcriptional regulator, copper-sensing transcriptional repressor
CP004065.1_3433	K14166	176.60	414.1	2.4e-124	copper transport protein
CP004065.1_3433	K07156	71.17	100.6	2.7e-29	copper resistance protein C
CP004065.1_3435	K00034	340.40	391.3	5e-118	glucose 1-dehydrogenase [EC:1.1.1.47]
CP004065.1_3435	K00059	269.80	277.3	4.7e-83	3-oxoacyl-[acyl-carrier protein] reductase [EC:1.1.1.100]
CP004065.1_3436	K05340	244.30	353.4	3.6e-106	glucose uptake protein
CP004065.1_3437	K00135	580.20	727.3	3.4e-219	succinate-semialdehyde dehydrogenase / glutarate-semialdehyde dehydrogenase [EC:1.2.1.16 1.2.1.79 1.2.1.20]
CP004065.1_3438	K07250	641.20	645.9	7.2e-195	4-aminobutyrate aminotransferase / (S)-3-amino-2-methylpropionate transaminase / 5-aminovalerate transaminase [EC:2.6.1.19 2.6.1.22 2.6.1.48]
CP004065.1_3439	K00375	343.30	478.5	6.3e-144	GntR family transcriptional regulator / MocR family aminotransferase
CP004065.1_3442	K19286	287.07	363.0	1.2e-109	FMN reductase [NAD(P)H] [EC:1.5.1.39]
CP004065.1_3444	K03446	476.67	518.2	5.6e-156	MFS transporter, DHA2 family, multidrug resistance protein
CP004065.1_3445	K25282	200.83	436.3	1.4e-131	iron-siderophore transport system substrate-binding protein
CP004065.1_3446	K25285	297.63	416.8	9.1e-126	iron-siderophore transport system ATP-binding protein [EC:7.2.2.-]
CP004065.1_3446	K02013	238.30	272.7	1.3e-81	iron complex transport system ATP-binding protein [EC:7.2.2.-]
CP004065.1_3447	K25283	156.17	450.3	1.4e-135	iron-siderophore transport system permease protein
CP004065.1_3448	K25284	273.03	475.3	2.5e-143	iron-siderophore transport system permease protein
CP004065.1_3450	K06353	81.93	92.8	1.7e-27	phosphatase RapC regulator
CP004065.1_3451	K06361	446.60	497.4	4.9e-150	response regulator aspartate phosphatase C [EC:3.1.-.-]
CP004065.1_3453	K02483	242.00	270.7	5.6e-81	two-component system, OmpR family, response regulator
CP004065.1_3454	K02004	52.33	73.7	2.3e-21	putative ABC transport system permease protein
CP004065.1_3457	K21613	481.13	652.9	2.7e-197	N-acetylcysteine deacetylase [EC:3.5.1.-]
CP004065.1_3458	K02028	386.70	414.6	1.3e-124	polar amino acid transport system ATP-binding protein [EC:7.4.2.1]
CP004065.1_3458	K16963	391.73	403.7	4.4e-122	putative S-methylcysteine transport system ATP-binding protein
CP004065.1_3459	K16962	261.07	326.5	1.7e-98	putative S-methylcysteine transport system permease protein
CP004065.1_3459	K02029	214.53	264.5	3.7e-79	polar amino acid transport system permease protein
CP004065.1_3460	K16961	271.80	368.7	2.9e-111	putative S-methylcysteine transport system substrate-binding protein
CP004065.1_3460	K02030	76.77	167.1	1.4e-49	polar amino acid transport system substrate-binding protein
CP004065.1_3461	K24117	207.53	263.1	2e-79	S-(2-succino)cysteine N-acetyltransferase [EC:2.3.1.-]
CP004065.1_3462	K24116	523.57	714.8	8.2e-216	N-acetyl-S-(2-succino)cysteine monooxygenase [EC:1.14.-.-]
CP004065.1_3463	K06296	248.90	347.6	2.2e-104	spore germination protein KB
CP004065.1_3464	K06297	281.23	348.5	1.3e-104	spore germination protein KC
CP004065.1_3465	K06295	452.20	767.2	5e-231	spore germination protein KA
CP004065.1_3465	K06307	659.47	659.5	5.8e-199	spore germination protein
CP004065.1_3468	K03305	359.10	534.1	7.2e-161	proton-dependent oligopeptide transporter, POT family
CP004065.1_3470	K21759	32.37	113.8	5.4e-34	vanillate/4-hydroxybenzoate decarboxylase subunit D [EC:4.1.1.- 4.1.1.61]
CP004065.1_3471	K01612	577.73	849.8	8.6e-257	vanillate/4-hydroxybenzoate decarboxylase subunit C [EC:4.1.1.- 4.1.1.61]
CP004065.1_3472	K03186	92.57	326.4	5.1e-98	flavin prenyltransferase [EC:2.5.1.129]
CP004065.1_3473	K21755	243.37	384.4	6.8e-116	LysR family transcriptional regulator, salicylic acid-responsive activator of bsdBCD
CP004065.1_3474	K02424	298.53	347.8	2.1e-104	L-cystine transport system substrate-binding protein
CP004065.1_3474	K02030	76.77	145.9	4.1e-43	polar amino acid transport system substrate-binding protein
CP004065.1_3475	K10009	301.87	371.4	1.5e-111	L-cystine transport system permease protein
CP004065.1_3475	K02029	214.53	276.4	9.4e-83	polar amino acid transport system permease protein
CP004065.1_3476	K02028	386.70	431.5	9.8e-130	polar amino acid transport system ATP-binding protein [EC:7.4.2.1]

CP004065.1_3476	K10010	381.10	408.5	1.8e-123	L-cystine transport system ATP-binding protein [EC:7.4.2.1]
CP004065.1_3477	K07149	76.20	212.9	1e-63	uncharacterized protein
CP004065.1_3478	K06133	60.17	165.0	6e-49	4'-phosphopantetheinyl transferase [EC:2.7.8.-]
CP004065.1_3480	K15657	230.93	399.7	7.9e-121	external thioesterase TEII
CP004065.1_3481	K15656	2211.63	2616.8	0	surfactin/lichenysin synthetase C
CP004065.1_3482	K15654	6154.90	6597.6	0	surfactin/lichenysin synthetase A
CP004065.1_3484	K08093	178.23	284.2	2.7e-85	3-hexulose-6-phosphate synthase [EC:4.1.2.43]
CP004065.1_3485	K08094	107.17	246.1	7e-74	6-phospho-3-hexuloisomerase [EC:5.3.1.27]
CP004065.1_3493	K26139	720.47	1060.2	9.4e-320	nitrite reductase [NAD(P)H] large subunit [EC:1.7.1.4]
CP004065.1_3494	K26138	104.60	144.2	4.8e-43	nitrite reductase [NAD(P)H] small subunit [EC:1.7.1.4]
CP004065.1_3495	K13542	404.20	465.1	9.2e-140	uroporphyrinogen III methyltransferase / synthase [EC:2.1.1.107 4.2.1.75]
CP004065.1_3495	K02303	255.53	369.7	4.2e-111	uroporphyrin-III C-methyltransferase [EC:2.1.1.107]
CP004065.1_3496	K21567	376.93	454.7	3.9e-137	ferredoxin/flavodoxin---NADP+ reductase [EC:1.18.1.2 1.19.1.1]
CP004065.1_3497	K23253	221.73	467.2	9.3e-141	PucR family transcriptional regulator, proline-responsive transcriptional activator
CP004065.1_3498	K11928	556.47	738.5	8.6e-223	sodium/proline symporter
CP004065.1_3498	K03307	271.33	360.8	3.1e-108	solute:Na+ symporter, SSS family
CP004065.1_3499	K00294	478.63	507.1	1.4e-152	1-pyrroline-5-carboxylate dehydrogenase [EC:1.2.1.88]
CP004065.1_3500	K00318	179.80	198.5	4.2e-59	proline dehydrogenase [EC:1.5.5.2]
CP004065.1_3501	K07074	59.80	260.9	3.8e-78	uncharacterized protein
CP004065.1_3502	K01060	117.80	410.4	1.9e-123	cephalosporin-C deacetylase [EC:3.1.1.41]
CP004065.1_3503	K00891	128.77	131.1	1.7e-38	shikimate kinase [EC:2.7.1.71]
CP004065.1_3504	K08194	514.17	559.2	1.2e-168	MFS transporter, ACS family, D-galactonate transporter
CP004065.1_3508	K01916	281.93	360.0	4.6e-108	NAD+ synthase [EC:6.3.1.5]
CP004065.1_3509	K09967	177.80	239.3	1.6e-71	uncharacterized protein
CP004065.1_3511	K09190	62.83	279.0	1.2e-83	uncharacterized protein
CP004065.1_3512	K06895	83.83	97.0	2.1e-28	L-lysine exporter family protein LysE/ArgO
CP004065.1_3515	K00016	308.33	436.0	5.6e-131	L-lactate dehydrogenase [EC:1.1.1.27]
CP004065.1_3516	K01176	222.80	264.9	3.3e-79	alpha-amylase [EC:3.2.1.1]
CP004065.1_3519	K14665	331.20	586.5	4.1e-177	amidohydrolase [EC:3.5.1.-]
CP004065.1_3520	K02002	122.57	228.3	4.3e-68	glycine betaine/proline transport system substrate-binding protein
CP004065.1_3521	K02001	326.57	444.0	2.7e-133	glycine betaine/proline transport system permease protein
CP004065.1_3522	K02000	415.90	652.1	2.9e-196	glycine betaine/proline transport system ATP-binding protein [EC:7.6.2.9]
CP004065.1_3523	K08369	358.47	478.1	6.1e-144	MFS transporter, putative metabolite:H+ symporter
CP004065.1_3527	K05795	235.30	325.3	1.1e-97	tellurium resistance protein TerD
CP004065.1_3528	K05795	235.30	315.1	1.3e-94	tellurium resistance protein TerD
CP004065.1_3529	K05795	235.30	246.4	1e-73	tellurium resistance protein TerD
CP004065.1_3529	K05791	193.43	195.6	1.3e-58	tellurium resistance protein TerZ
CP004065.1_3531	K09816	273.47	296.8	4.5e-89	zinc transport system permease protein
CP004065.1_3533	K09815	268.47	395.4	1.1e-118	zinc transport system substrate-binding protein
CP004065.1_3535	K00034	340.40	372.5	2.6e-112	glucose 1-dehydrogenase [EC:1.1.1.47]
CP004065.1_3535	K00059	269.80	304.7	2.3e-91	3-oxoacyl-[acyl-carrier protein] reductase [EC:1.1.1.100]
CP004065.1_3536	K06368	538.80	585.4	9.6e-177	response regulator aspartate phosphatase J [EC:3.1.-.-]
CP004065.1_3537	K17733	58.10	174.5	5.9e-52	peptidoglycan LD-endopeptidase CwlK [EC:3.4.-.-]
CP004065.1_3541	K06607	318.77	428.9	2e-129	myo-inositol catabolism protein IolS [EC:1.1.1.-]
CP004065.1_3543	K01046	52.07	64.7	2.1e-18	triacylglycerol lipase [EC:3.1.1.3]
CP004065.1_3544	K01424	50.80	315.1	2.4e-94	L-asparaginase [EC:3.5.1.1]
CP004065.1_3546	K18939	163.23	262.3	5.5e-79	TetR/AcrR family transcriptional regulator, lmrAB and yxaGH operons repressor
CP004065.1_3546	K16137	104.80	115.1	1.1e-33	TetR/AcrR family transcriptional regulator, transcriptional repressor for nem operon
CP004065.1_3547	K18926	574.20	625.2	1.5e-188	MFS transporter, DHA2 family, lincomycin resistance protein
CP004065.1_3547	K03446	476.67	477.3	1.3e-143	MFS transporter, DHA2 family, multidrug resistance protein
CP004065.1_3549	K01304	52.83	196.4	1.5e-58	pyroglutamyl-peptidase [EC:3.4.19.3]
CP004065.1_3550	K03118	86.40	248.6	2.4e-74	sec-independent protein translocase protein TatC
CP004065.1_3551	K03116	54.03	71.1	2.6e-20	sec-independent protein translocase protein TatA
CP004065.1_3552	K01113	89.83	541.6	6.9e-163	alkaline phosphatase D [EC:3.1.3.1]
CP004065.1_3553	K01449	48.60	136.6	2.1e-40	cell wall hydrolase
CP004065.1_3556	K15977	60.27	95.2	1.3e-27	putative oxidoreductase

CP004065.1_3557	K15975	357.47	479.4	2.5e-144	glyoxalase family protein
CP004065.1_3558	K15976	141.90	350.8	4e-106	putative NAD(P)H nitroreductase [EC:1.-.-.-]
CP004065.1_3559	K13283	187.13	200.2	8.4e-60	ferrous-iron efflux pump FieF
CP004065.1_3561	K06979	169.83	305.9	1.3e-91	macrolide phosphotransferase
CP004065.1_3562	K07719	195.10	401.0	6e-121	two-component system, response regulator GlnL
CP004065.1_3563	K07717	229.97	556.6	6.3e-168	two-component system, sensor histidine kinase GlnK [EC:2.7.13.3]
CP004065.1_3564	K01425	83.30	360.1	5.2e-108	glutaminase [EC:3.5.1.2]
CP004065.1_3565	K11626	823.40	893.7	4.5e-270	putative sodium/glutamine symporter
CP004065.1_3565	K03310	262.87	580.8	6.1e-175	alanine or glycine:cation symporter, AGCS family
CP004065.1_3566	K00547	285.53	435.0	6.8e-131	homocysteine S-methyltransferase [EC:2.1.1.10]
CP004065.1_3567	K16235	689.77	789.1	2.7e-238	S-methylmethionine transporter
CP004065.1_3568	K00826	167.23	456.0	2.9e-137	branched-chain amino acid aminotransferase [EC:2.6.1.42]
CP004065.1_3569	K11102	518.13	536.4	7e-162	proton glutamate symport protein
CP004065.1_3570	K07146	171.23	351.1	2e-105	UPF0176 protein
CP004065.1_3572	K01613	33.77	207.9	6.2e-62	phosphatidylserine decarboxylase [EC:4.1.1.65]
CP004065.1_3573	K03975	59.97	109.2	4.6e-32	membrane-associated protein
CP004065.1_3574	K17103	126.47	183.7	1.5e-54	CDP-diacylglycerol---serine O-phosphatidyltransferase [EC:2.7.8.8]
CP004065.1_3575	K08289	265.07	694.0	7.4e-209	phosphoribosylglycinamide formyltransferase 2 [EC:6.3.1.21]
CP004065.1_3576	K08317	331.70	443.4	9.9e-134	hydroxycarboxylate dehydrogenase A [EC:1.1.1.-]
CP004065.1_3581	K00483	411.73	612.1	1.3e-184	4-hydroxyphenylacetate 3-monooxygenase [EC:1.14.14.9]
CP004065.1_3583	K02445	506.37	672.0	9.3e-203	MFS transporter, OPA family, glycerol-3-phosphate transporter
CP004065.1_3584	K01126	152.53	265.4	1.3e-79	glycerophosphoryl diester phosphodiesterase [EC:3.1.4.46]
CP004065.1_3593	K25142	220.57	224.3	5.4e-67	linearmycin/streptolysin S transport system permease protein
CP004065.1_3594	K25143	233.20	269.8	9.6e-81	linearmycin/streptolysin S transport system permease protein
CP004065.1_3595	K25144	376.07	412.9	3.9e-124	linearmycin/streptolysin S transport system ATP-binding protein
CP004065.1_3595	K01990	262.37	314.3	3.1e-94	ABC-2 type transport system ATP-binding protein
CP004065.1_3598	K07025	112.47	115.4	6.5e-34	putative hydrolase of the HAD superfamily
CP004065.1_3599	K03293	609.50	636.5	9.4e-192	amino acid transporter, AAT family
CP004065.1_3601	K06518	37.43	158.5	3.2e-47	holin-like protein
CP004065.1_3603	K06221	349.23	377.8	7.6e-114	2,5-diketo-D-gluconate reductase A [EC:1.1.1.346]
CP004065.1_3610	K00820	183.57	695.7	1.9e-209	glutamine---fructose-6-phosphate transaminase (isomerizing) [EC:2.6.1.16]
CP004065.1_3611	K03431	454.20	680.0	6.6e-205	phosphoglucosamine mutase [EC:5.4.2.10]
CP004065.1_3613	K18672	81.73	364.5	1.9e-109	diadenylate cyclase [EC:2.7.7.85]
CP004065.1_3615	K03088	96.50	178.8	3.3e-53	RNA polymerase sigma-70 factor, ECF subfamily
CP004065.1_3618	K07106	127.00	463.0	2.8e-139	N-acetylmuramic acid 6-phosphate etherase [EC:4.2.1.126]
CP004065.1_3620	K21469	465.57	519.2	2.9e-156	serine-type D-Ala-D-Ala carboxypeptidase [EC:3.4.16.4]
CP004065.1_3621	K01207	251.63	574.5	6.6e-173	beta-N-acetylhexosaminidase [EC:3.2.1.52]
CP004065.1_3623	K21701	333.73	545.4	3.3e-164	AraC family transcriptional regulator, transcriptional activator for feuABC-ybbA operon
CP004065.1_3629	K03321	345.37	516.4	2.7e-155	sulfate permease, SulP family
CP004065.1_3633	K08174	208.07	410.9	7.9e-124	MFS transporter, FHS family, glucose/mannose:H+ symporter
CP004065.1_3637	K22278	73.27	175.5	4.2e-52	peptidoglycan-N-acetylglucosamine deacetylase [EC:3.5.1.104]
CP004065.1_3638	K06349	23.47	273.4	2.5e-82	KinB signaling pathway activation protein
CP004065.1_3639	K06294	84.50	229.6	5.5e-69	spore germination protein D
CP004065.1_3640	K03593	111.47	361.0	2.7e-108	ATP-binding protein involved in chromosome partitioning
CP004065.1_3641	K01448	29.13	199.1	2.1e-59	N-acetylmuramoyl-L-alanine amidase [EC:3.5.1.28]
CP004065.1_3645	K02996	103.27	210.6	8.2e-63	small subunit ribosomal protein S9
CP004065.1_3646	K02871	66.60	236.1	1.3e-70	large subunit ribosomal protein L13
CP004065.1_3647	K06173	231.17	267.3	6.2e-80	tRNA pseudouridine38-40 synthase [EC:5.4.99.12]
CP004065.1_3648	K16785	103.27	234.9	2.5e-70	energy-coupling factor transport system permease protein
CP004065.1_3649	K16787	356.33	406.3	3.1e-122	energy-coupling factor transport system ATP-binding protein [EC:7.-.-.-]
CP004065.1_3650	K16786	352.00	418.7	7.9e-126	energy-coupling factor transport system ATP-binding protein [EC:7.-.-.-]
CP004065.1_3651	K02879	39.00	203.1	1.7e-60	large subunit ribosomal protein L17
CP004065.1_3652	K03040	50.70	411.9	5.1e-124	DNA-directed RNA polymerase subunit alpha [EC:2.7.7.6]
CP004065.1_3653	K02948	100.47	191.0	7e-57	small subunit ribosomal protein S11
CP004065.1_3654	K02952	113.37	189.7	1.4e-56	small subunit ribosomal protein S13
CP004065.1_3655	K02919	24.63	59.3	1.3e-16	large subunit ribosomal protein L36

CP004065.1_3656	K02518	37.43	154.5	3.2e-46	translation initiation factor IF-1
CP004065.1_3657	K01265	141.83	338.9	8.7e-102	methionyl aminopeptidase [EC:3.4.11.18]
CP004065.1_3658	K00939	185.70	251.9	2.6e-75	adenylate kinase [EC:2.7.4.3]
CP004065.1_3659	K03076	210.97	544.4	5.5e-164	preprotein translocase subunit SecY
CP004065.1_3660	K02876	42.80	210.3	1.1e-62	large subunit ribosomal protein L15
CP004065.1_3661	K02907	40.27	75.5	8.6e-22	large subunit ribosomal protein L30
CP004065.1_3662	K02988	134.97	261.0	3.6e-78	small subunit ribosomal protein S5
CP004065.1_3663	K02881	45.33	166.2	2e-49	large subunit ribosomal protein L18
CP004065.1_3664	K02933	107.80	267.2	3.9e-80	large subunit ribosomal protein L6
CP004065.1_3665	K02994	80.50	202.1	2.6e-60	small subunit ribosomal protein S8
CP004065.1_3666	K02954	26.53	71.1	2.5e-20	small subunit ribosomal protein S14
CP004065.1_3667	K02931	104.27	334.0	1.4e-100	large subunit ribosomal protein L5
CP004065.1_3668	K02895	56.73	150.6	1.3e-44	large subunit ribosomal protein L24
CP004065.1_3669	K02874	94.83	184.3	3.9e-55	large subunit ribosomal protein L14
CP004065.1_3670	K02961	80.63	131.7	5.4e-39	small subunit ribosomal protein S17
CP004065.1_3671	K02904	59.10	99.8	3.5e-29	large subunit ribosomal protein L29
CP004065.1_3672	K02878	90.63	243.4	6.4e-73	large subunit ribosomal protein L16
CP004065.1_3673	K02982	103.63	349.5	3.2e-105	small subunit ribosomal protein S3
CP004065.1_3674	K02890	79.73	186.0	1.7e-55	large subunit ribosomal protein L22
CP004065.1_3675	K02965	102.33	154.5	5.1e-46	small subunit ribosomal protein S19
CP004065.1_3676	K02886	164.10	414.9	7.3e-125	large subunit ribosomal protein L2
CP004065.1_3677	K02892	57.40	89.1	9e-26	large subunit ribosomal protein L23
CP004065.1_3678	K02926	92.37	295.6	1.1e-88	large subunit ribosomal protein L4
CP004065.1_3679	K02906	104.83	322.8	9.6e-97	large subunit ribosomal protein L3
CP004065.1_3680	K02946	61.53	161.9	5.9e-48	small subunit ribosomal protein S10
CP004065.1_3682	K02358	336.50	786.2	2.8e-237	elongation factor Tu
CP004065.1_3683	K02355	521.90	1087.4	0	elongation factor G
CP004065.1_3684	K02992	105.50	224.5	3.7e-67	small subunit ribosomal protein S7
CP004065.1_3685	K02950	75.93	241.3	1.9e-72	small subunit ribosomal protein S12
CP004065.1_3686	K07590	76.57	110.9	4.2e-33	large subunit ribosomal protein L7A
CP004065.1_3687	K03046	932.17	2161.0	0	DNA-directed RNA polymerase subunit beta' [EC:2.7.7.6]
CP004065.1_3688	K03043	656.80	1843.1	0	DNA-directed RNA polymerase subunit beta [EC:2.7.7.6]
CP004065.1_3689	K00564	110.40	209.9	1.4e-62	16S rRNA (guanine1207-N2)-methyltransferase [EC:2.1.1.172]
CP004065.1_3690	K02935	22.77	151.8	7.1e-45	large subunit ribosomal protein L7/L12
CP004065.1_3691	K02864	51.60	189.2	2.6e-56	large subunit ribosomal protein L10
CP004065.1_3692	K02863	85.67	319.3	8.1e-96	large subunit ribosomal protein L1
CP004065.1_3693	K02867	62.17	255.1	2.8e-76	large subunit ribosomal protein L11
CP004065.1_3694	K02601	101.20	280.8	2.8e-84	transcription termination/antitermination protein NusG
CP004065.1_3695	K03073	20.17	67.7	2.6e-19	preprotein translocase subunit SecE
CP004065.1_3696	K27000	107.77	352.2	4.2e-106	RNA polymerase sigma-H factor
CP004065.1_3697	K06962	56.63	176.1	2.4e-52	uncharacterized protein
CP004065.1_3698	K03218	212.77	360.8	1.5e-108	23S rRNA (guanosine2251-2'-O)-methyltransferase [EC:2.1.1.185]
CP004065.1_3699	K11145	45.73	175.1	2.3e-52	mini-ribonuclease III [EC:3.1.26.-]
CP004065.1_3700	K01883	400.67	675.1	3.5e-203	cysteinyl-tRNA synthetase [EC:6.1.1.16]
CP004065.1_3701	K00640	93.33	308.0	2.3e-92	serine O-acetyltransferase [EC:2.3.1.30]
CP004065.1_3702	K09698	647.87	767.1	2.3e-231	nondiscriminating glutamyl-tRNA synthetase [EC:6.1.1.24]
CP004065.1_3702	K01885	298.30	617.2	1.3e-185	glutamyl-tRNA synthetase [EC:6.1.1.17]
CP004065.1_3703	K01770	138.60	266.8	3.5e-80	2-C-methyl-D-erythritol 2,4-cyclodiphosphate synthase [EC:4.6.1.12]
CP004065.1_3704	K00991	252.03	308.2	1.6e-92	2-C-methyl-D-erythritol 4-phosphate cytidylyltransferase [EC:2.7.7.60]
CP004065.1_3706	K07067	327.90	492.4	2e-148	diadenylate cyclase [EC:2.7.7.85]
CP004065.1_3707	K04485	124.17	716.8	4.1e-216	DNA repair protein RadA/Sms
CP004065.1_3708	K03696	1063.70	1252.0	0	ATP-dependent Clp protease ATP-binding subunit ClpC
CP004065.1_3709	K19405	208.57	524.4	4.1e-158	protein arginine kinase [EC:2.7.14.1]
CP004065.1_3710	K19411	58.37	146.5	1.9e-43	protein arginine kinase activator
CP004065.1_3711	K03708	95.60	214.7	1.9e-64	transcriptional regulator of stress and heat shock response
CP004065.1_3714	K04567	319.33	803.9	3.6e-242	lysyl-tRNA synthetase, class II [EC:6.1.1.6]

CP004065.1_3715	K05540	281.20	474.4	6.2e-143	tRNA-dihydrouridine synthase B [EC:1.-.-.-]
CP004065.1_3717	K00950	38.63	207.0	6.6e-62	2-amino-4-hydroxy-6-hydroxymethyldihydropteridine diphosphokinase [EC:2.7.6.3]
CP004065.1_3718	K01633	94.10	166.6	1.6e-49	7,8-dihydroneopterin aldolase/epimerase/oxygenase [EC:4.1.2.25 5.1.99.8 1.13.11.81]
CP004065.1_3719	K00796	237.57	398.0	9.9e-120	dihydropteroate synthase [EC:2.5.1.15]
CP004065.1_3720	K02619	196.63	205.1	4.7e-61	4-amino-4-deoxychorismate lyase [EC:4.1.3.38]
CP004065.1_3721	K25575	332.40	346.5	6.5e-105	4-amino-4-deoxychorismate synthase (2-amino-4-deoxychorismate-forming) component II [EC:2.6.1.123]
CP004065.1_3721	K01664	320.30	335.0	8.7e-101	para-aminobenzoate synthetase component II [EC:2.6.1.85]
CP004065.1_3721	K01658	241.93	327.5	1e-98	anthranilate synthase component II [EC:4.1.3.27]
CP004065.1_3722	K25578	527.73	636.7	5.2e-192	4-amino-4-deoxychorismate synthase (2-amino-4-deoxychorismate-forming) component I [EC:2.6.1.123]
CP004065.1_3722	K01657	465.93	556.9	1.3e-167	anthranilate synthase component I [EC:4.1.3.27]
CP004065.1_3723	K01738	426.93	500.2	1.3e-150	cysteine synthase [EC:2.5.1.47]
CP004065.1_3725	K04083	58.73	348.6	8.2e-105	molecular chaperone Hsp33
CP004065.1_3726	K03525	120.67	287.9	2.9e-86	type III pantothenate kinase [EC:2.7.1.33]
CP004065.1_3727	K03798	767.57	966.2	3.8e-291	cell division protease FtsH [EC:3.4.24.-]
CP004065.1_3728	K00760	82.30	244.1	4.7e-73	hypoxanthine phosphoribosyltransferase [EC:2.4.2.8]
CP004065.1_3729	K04075	118.80	305.0	2e-91	tRNA(Ile)-lysidine synthase [EC:6.3.4.19]
CP004065.1_3732	K06382	265.27	1010.0	1.8e-304	stage II sporulation protein E [EC:3.1.3.16]
CP004065.1_3733	K07571	145.30	213.1	3.2e-64	S1 RNA binding domain protein
CP004065.1_3734	K13052	58.13	113.7	1.6e-33	cell division protein DivIC
CP004065.1_3737	K04762	59.53	63.5	5.2e-18	ribosome-associated heat shock protein Hsp15
CP004065.1_3738	K02499	400.47	732.4	5.3e-221	tetrapyrrole methylase family protein / MazG family protein
CP004065.1_3739	K03328	304.87	386.4	5.4e-116	polysaccharide transporter, PST family
CP004065.1_3740	K04769	147.60	293.0	2.2e-88	AbrB family transcriptional regulator, stage V sporulation protein T
CP004065.1_3741	K03723	555.70	1394.3	0	transcription-repair coupling factor (superfamily II helicase) [EC:5.6.2.4]
CP004065.1_3743	K01056	49.10	259.1	1.4e-77	peptidyl-tRNA hydrolase, PTH1 family [EC:3.1.1.29]
CP004065.1_3744	K02897	35.70	188.4	3.7e-56	large subunit ribosomal protein L25
CP004065.1_3745	K00948	62.27	495.2	3.7e-149	ribose-phosphate pyrophosphokinase [EC:2.7.6.1]
CP004065.1_3746	K04042	321.87	664.1	6e-200	bifunctional UDP-N-acetylglucosamine pyrophosphorylase / glucosamine-1-phosphate N-acetyltransferase [EC:2.7.7.23 2.3.1.157]
CP004065.1_3747	K06412	27.70	154.8	2.7e-46	stage V sporulation protein G
CP004065.1_3748	K09022	107.97	203.1	1.4e-60	2-iminobutanoate/2-iminopropanoate deaminase [EC:3.5.99.10]
CP004065.1_3749	K09685	258.10	423.7	4.3e-128	HTH-type transcriptional regulator, purine operon repressor
CP004065.1_3750	K00919	102.90	323.2	4.6e-97	4-diphosphocytidyl-2-C-methyl-D-erythritol kinase [EC:2.7.1.148]
CP004065.1_3751	K06423	62.50	120.4	1e-35	small acid-soluble spore protein F (minor alpha/beta-type SASP)
CP004065.1_3753	K06436	272.67	431.8	2.1e-130	spore coat assemly protein
CP004065.1_3754	K02528	256.10	354.0	2.4e-106	16S rRNA (adenine1518-N6/adenine1519-N6)-dimethyltransferase [EC:2.1.1.182]
CP004065.1_3755	K05985	117.63	284.5	1.3e-85	ribonuclease M5 [EC:3.1.26.8]
CP004065.1_3756	K21688	167.47	210.2	1.2e-62	resuscitation-promoting factor RpfB
CP004065.1_3757	K03424	86.70	330.8	3.2e-99	TatD DNase family protein [EC:3.1.21.-]
CP004065.1_3758	K01874	185.23	718.4	2.2e-216	methionyl-tRNA synthetase [EC:6.1.1.10]
CP004065.1_3759	K06284	86.83	129.2	1.5e-38	AbrB family transcriptional regulator, transcriptional pleiotropic regulator of transition state genes
CP004065.1_3760	K07056	92.80	395.6	4.3e-119	16S rRNA (cytidine1402-2'-O)-methyltransferase [EC:2.1.1.198]
CP004065.1_3761	K07461	67.27	126.0	3.5e-37	putative endonuclease
CP004065.1_3762	K15460	135.00	156.4	2.2e-46	tRNA1Val (adenine37-N6)-methyltransferase [EC:2.1.1.223]
CP004065.1_3765	K02341	230.10	279.5	1.4e-83	DNA polymerase III subunit delta' [EC:2.7.7.7]
CP004065.1_3766	K09770	68.60	146.7	1.6e-43	uncharacterized protein
CP004065.1_3768	K00943	96.67	230.6	6.8e-69	dTMP kinase [EC:2.7.4.9]
CP004065.1_3769	K01585	258.33	409.0	1e-122	arginine decarboxylase [EC:4.1.1.19]
CP004065.1_3774	K06317	31.03	82.5	6.5e-24	inhibitor of the pro-sigma K processing machinery
CP004065.1_3776	K06187	59.67	349.5	5.1e-105	recombination protein RecR
CP004065.1_3777	K09747	46.57	152.3	2.7e-45	nucleoid-associated protein EbfC
CP004065.1_3778	K02343	233.40	555.3	3.5e-167	DNA polymerase III subunit gamma/tau [EC:2.7.7.7]
CP004065.1_3779	K11991	164.80	269.2	2.2e-80	tRNA(adenine34) deaminase [EC:3.5.4.33]
CP004065.1_3781	K06306	390.77	659.6	3.4e-199	cortical fragment-lytic enzyme [EC:3.2.1.-]
CP004065.1_3782	K15518	191.63	318.8	3.9e-96	deoxyguanosine kinase [EC:2.7.1.113]
CP004065.1_3783	K15519	203.27	310.0	2.8e-93	deoxyadenosine/deoxycytidine kinase [EC:2.7.1.76 2.7.1.74]
CP004065.1_3784	K00865	57.57	526.1	1.9e-158	glycerate 2-kinase [EC:2.7.1.165]

CP004065.1_3785	K01875	89.73	616.4	1.7e-185	seryl-tRNA synthetase [EC:6.1.1.11]
CP004065.1_3786	K08681	77.37	294.1	1.7e-88	pyridoxal 5'-phosphate synthase pdxT subunit [EC:4.3.3.6]
CP004065.1_3787	K06215	123.87	538.2	1.9e-162	pyridoxal 5'-phosphate synthase pdxS subunit [EC:4.3.3.6]
CP004065.1_3788	K07258	223.10	340.4	4.2e-102	serine-type D-Ala-D-Ala carboxypeptidase (penicillin-binding protein 5/6) [EC:3.4.16.4]
CP004065.1_3789	K00088	375.03	765.4	1.7e-230	IMP dehydrogenase [EC:1.1.1.205]
CP004065.1_3792	K02469	1032.73	1325.9	0	DNA gyrase subunit A [EC:5.6.2.2]
CP004065.1_3793	K02470	944.40	1107.1	0	DNA gyrase subunit B [EC:5.6.2.2]
CP004065.1_3793	K02622	875.97	886.0	3.4e-267	topoisomerase IV subunit B [EC:5.6.2.2]
CP004065.1_3794	K24648	42.73	93.5	2.5e-27	extracellular matrix regulatory protein B
CP004065.1_3795	K03629	104.40	423.4	2.9e-127	DNA replication and repair protein RecF
CP004065.1_3796	K14761	48.70	99.4	5e-29	ribosome-associated protein
CP004065.1_3797	K02338	60.60	384.3	2.2e-115	DNA polymerase III subunit beta [EC:2.7.7.7]
CP004065.1_3798	K02313	129.33	703.2	6.7e-212	chromosomal replication initiator protein
CP004119.1_4	K03100	114.77	186.0	2.1e-55	signal peptidase I [EC:3.4.21.89]
