# Supplementary material for: Prediction of soil probiotics based on foundation model representation enhancement and stacked aggregation classifier
Source: Brief Bioinform. 2025 Oct 29;26(5):bbaf567. doi: 10.1093/bib/bbaf567 (PMC12570017; doi:10.1093/bib/bbaf567)
Supplement: Supplementary_Table_S6_R2_bbaf567 [file supplementary_table_s6_r2_bbaf567.pdf]

Supplementary Table S6. Genes and their KO identifiers of GCA_015710975.1.

Gene_ID	KO	Threshold	Score	*e* _value	KO_definition
CP049783.1_1	K23228	462.50	484.8	1.1e-145	ferric hydroxamate transport system permease protein
CP049783.1_2	K23227	189.93	314.7	2.2e-94	ferric hydroxamate transport system substrate-binding protein
CP049783.1_3	K02520	34.97	133.3	3.5e-39	translation initiation factor IF-3
CP049783.1_4	K17882	113.07	363.4	1.5e-109	kanamycin nucleotidyltransferase [EC:2.7.7.-]
CP049783.1_8	K25232	87.57	283.2	1e-84	fatty acid kinase fatty acid binding subunit
CP049783.1_11	K06133	60.17	158.8	6.6e-47	4'-phosphopantetheinyl transferase [EC:2.7.8.-]
CP049783.1_13	K01073	193.53	202.1	1.4e-60	acyl-CoA hydrolase [EC:3.1.2.20]
CP049783.1_14	K23779	92.83	187.9	7.9e-56	XRE family transcriptional regulator, regulator of sulfur utilization
CP049783.1_16	K26605	54.60	225.9	1.8e-67	branched chain amino acid efflux pump
CP049783.1_17	K26606	27.30	73.9	3.5e-21	branched chain amino acid efflux pump
CP049783.1_18	K20485	678.97	830.4	2.5e-250	ATP-binding cassette, subfamily B, bacterial NisT/SpaT
CP049783.1_19	K20483	108.60	935.9	1e-281	class I lanthipeptide synthase [EC:3.13.2.4]
CP049783.1_21	K20484	130.10	275.5	3.1e-82	class I lanthipeptide synthase [EC:3.13.2.4]
CP049783.1_23	K20484	130.10	331.2	4e-99	class I lanthipeptide synthase [EC:3.13.2.4]
CP049783.1_24	K20461	155.27	194.4	6.6e-58	lantibiotic transport system permease protein
CP049783.1_25	K20460	146.50	151.3	1e-44	lantibiotic transport system permease protein
CP049783.1_26	K20459	363.07	428.0	7.7e-129	lantibiotic transport system ATP-binding protein
CP049783.1_26	K01990	262.37	302.3	1.9e-90	ABC-2 type transport system ATP-binding protein
CP049783.1_29	K09786	182.07	556.2	2e-167	uncharacterized protein
CP049783.1_30	K06415	220.90	624.5	1.1e-187	stage V sporulation protein R
CP049783.1_33	K02483	242.00	249.7	2e-74	two-component system, OmpR family, response regulator
CP049783.1_34	K18941	299.00	327.9	7.6e-99	two-component system, OmpR family, response regulator ArlR
CP049783.1_34	K02483	242.00	269.7	1.6e-80	two-component system, OmpR family, response regulator
CP049783.1_35	K18940	370.43	474.6	1.5e-142	two-component system, OmpR family, sensor histidine kinase ArlS [EC:2.7.13.3]
CP049783.1_42	K03406	65.50	99.1	8.7e-29	methyl-accepting chemotaxis protein
CP049783.1_44	K07180	288.33	873.4	3.1e-263	serine protein kinase
CP049783.1_46	K05825	385.80	467.0	2.8e-140	2-aminoadipate transaminase [EC:2.6.1.-]
CP049783.1_50	K06284	86.83	117.4	1e-34	AbrB family transcriptional regulator, transcriptional pleiotropic regulator of transition state genes
CP049783.1_51	K03216	93.87	264.2	3.1e-79	tRNA (cytidine/uridine-2'-O-)-methyltransferase [EC:2.1.1.207]
CP049783.1_52	K00831	137.77	529.4	2.2e-159	phosphoserine aminotransferase [EC:2.6.1.52]
CP049783.1_54	K01915	33.97	337.1	7.9e-101	glutamine synthetase [EC:6.3.1.2]
CP049783.1_55	K03856	474.93	560.9	1.3e-168	3-deoxy-7-phosphoheptulonate synthase [EC:2.5.1.54]
CP049783.1_56	K03527	70.27	349.0	1.6e-104	4-hydroxy-3-methylbut-2-en-1-yl diphosphate reductase [EC:1.17.7.4]
CP049783.1_57	K02484	312.80	317.5	6.7e-95	two-component system, OmpR family, sensor kinase [EC:2.7.13.3]
CP049783.1_58	K07669	325.20	345.4	6.8e-104	two-component system, OmpR family, response regulator MprA
CP049783.1_58	K02483	242.00	298.8	2.4e-89	two-component system, OmpR family, response regulator
CP049783.1_65	K07228	80.77	184.0	5.1e-55	K+:H+ antiporter subunit KhtT
CP049783.1_66	K26732	261.93	464.2	1.1e-139	K+:H+ antiporter subunit KhtU
CP049783.1_68	K11784	435.33	580.3	8.6e-175	cyclic dehypoxanthinyl futalosine synthase [EC:1.21.98.1]
CP049783.1_69	K01760	493.17	526.9	1.3e-158	cysteine-S-conjugate beta-lyase [EC:4.4.1.13]
CP049783.1_71	K00651	244.77	485.0	4.3e-146	homoserine O-succinyltransferase/O-acetyltransferase [EC:2.3.1.46 2.3.1.31]
CP049783.1_92	K07171	19.10	79.2	1.4e-22	mRNA interferase MazF [EC:3.1.-.-]
CP049783.1_131	K22299	76.73	86.6	5.7e-25	HTH-type transcriptional regulator, competence development regulator
CP049783.1_134	K14059	167.87	244.1	1.1e-72	integrase
CP049783.1_135	K03284	165.70	308.6	2.6e-92	magnesium transporter
CP049783.1_138	K23786	338.90	416.7	1.3e-125	5'-3' exonuclease [EC:3.1.11.-]
CP049783.1_139	K00537	80.33	86.5	6.4e-25	arsenate reductase (glutaredoxin) [EC:1.20.4.1]
CP049783.1_140	K06180	285.23	356.4	7.5e-107	23S rRNA pseudouridine1911/1915/1917 synthase [EC:5.4.99.23]
CP049783.1_141	K00798	35.70	241.1	3.9e-72	cob(I)alamin adenosyltransferase [EC:2.5.1.17]
CP049783.1_144	K10907	500.80	600.7	6.7e-181	aminotransferase [EC:2.6.1.-]
CP049783.1_149	K08222	125.80	500.2	1.2e-150	MFS transporter, YQGE family, putative transporter
CP049783.1_151	K08989	42.47	153.5	1.1e-45	putative membrane protein
CP049783.1_153	K07456	353.83	810.5	7.5e-244	DNA mismatch repair protein MutS2
CP049783.1_154	K08972	34.93	54.6	4.8e-15	putative membrane protein

CP049783.1_155	K09888	23.30	46.0	1.7e-12	cell division protein ZapA
CP049783.1_156	K01890	122.80	653.8	1.2e-196	phenylalanyl-tRNA synthetase beta chain [EC:6.1.1.20]
CP049783.1_157	K01889	95.27	376.4	8.4e-113	phenylalanyl-tRNA synthetase alpha chain [EC:6.1.1.20]
CP049783.1_159	K01732	213.53	265.7	3.3e-79	pectin lyase [EC:4.2.2.10]
CP049783.1_163	K01185	73.43	180.9	1e-53	lysozyme [EC:3.2.1.17]
CP049783.1_182	K06904	50.60	156.8	3e-46	Escherichia/Staphylococcus phage prohead protease
CP049783.1_190	K24072	90.50	198.6	1.8e-59	positive control factor
CP049783.1_208	K14059	167.87	171.4	1.3e-50	integrase
CP049783.1_212	K03386	223.80	285.1	2.3e-85	peroxiredoxin 2/4 [EC:1.11.1.24]
CP049783.1_213	K00052	432.33	604.0	1.2e-181	3-isopropylmalate dehydrogenase [EC:1.1.1.85]
CP049783.1_214	K01649	498.33	650.1	2.4e-195	2-isopropylmalate synthase [EC:2.3.3.13]
CP049783.1_215	K00053	107.87	504.3	1.6e-151	ketol-acid reductoisomerase [EC:1.1.1.86]
CP049783.1_216	K01653	49.70	225.4	1.6e-67	acetolactate synthase I/III small subunit [EC:2.2.1.6]
CP049783.1_217	K01652	517.70	812.5	9.4e-245	acetolactate synthase I/II/III large subunit [EC:2.2.1.6]
CP049783.1_220	K23536	183.10	399.2	5.1e-120	general nucleoside transport system permease protein
CP049783.1_221	K23535	209.57	405.8	8.3e-122	general nucleoside transport system permease protein
CP049783.1_222	K23537	596.97	838.7	1.6e-252	general nucleoside transport system ATP-binding protein
CP049783.1_223	K07335	118.73	379.3	1.1e-113	basic membrane protein A and related proteins
CP049783.1_224	K02887	45.33	197.2	1.2e-58	large subunit ribosomal protein L20
CP049783.1_225	K02916	23.07	43.2	1.2e-11	large subunit ribosomal protein L35
CP049783.1_226	K02520	34.97	263.5	7.8e-79	translation initiation factor IF-3
CP049783.1_228	K19302	106.60	141.4	1.3e-41	undecaprenyl-diphosphatase [EC:3.6.1.27]
CP049783.1_229	K03429	296.23	375.0	2.3e-112	processive 1,2-diacylglycerol beta-glucosyltransferase [EC:2.4.1.315]
CP049783.1_230	K03439	68.23	173.8	2.4e-51	tRNA (guanine-N7-)-methyltransferase [EC:2.1.1.33]
CP049783.1_232	K07139	198.50	479.9	2.2e-144	uncharacterized protein
CP049783.1_234	K01809	33.17	208.4	8e-62	mannose-6-phosphate isomerase [EC:5.3.1.8]
CP049783.1_237	K00658	444.90	552.0	3.6e-166	2-oxoglutarate dehydrogenase E2 component (dihydrolipoamide succinyltransferase) [EC:2.3.1.61]
CP049783.1_243	K07727	44.53	84.6	2.2e-24	putative transcriptional regulator
CP049783.1_248	K02435	35.93	69.7	6.5e-20	aspartyl-tRNA(Asn)/glutamyl-tRNA(Gln) amidotransferase subunit C [EC:6.3.5.6 6.3.5.7]
CP049783.1_253	K03817	131.60	156.5	3.1e-46	ribosomal-protein-serine acetyltransferase [EC:2.3.1.-]
CP049783.1_254	K07025	112.47	160.7	1.6e-47	putative hydrolase of the HAD superfamily
CP049783.1_259	K15256	104.33	198.5	6.1e-59	tRNA (cmo5U34)-methyltransferase [EC:2.1.1.-]
CP049783.1_262	K00858	38.63	60.9	3.1e-17	NAD+ kinase [EC:2.7.1.23]
CP049783.1_264	K07017	254.87	350.6	4.4e-105	ferri-bacillibactin esterase [EC:3.1.-.-]
CP049783.1_269	K06950	47.43	226.6	2.1e-67	uncharacterized protein
CP049783.1_272	K21567	376.93	451.2	6.7e-136	ferredoxin/flavodoxin---NADP+ reductase [EC:1.18.1.2 1.19.1.1]
CP049783.1_281	K00570	92.93	132.4	6.6e-39	phosphatidylethanolamine/phosphatidyl-N-methylethanolamine N-methyltransferase [EC:2.1.1.17 2.1.1.71]
CP049783.1_282	K03975	59.97	106.5	4.3e-31	membrane-associated protein
CP049783.1_283	K01077	73.07	338.0	3.4e-101	alkaline phosphatase [EC:3.1.3.1]
CP049783.1_285	K03654	618.33	730.9	6.9e-220	ATP-dependent DNA helicase RecQ [EC:5.6.2.4]
CP049783.1_288	K01611	16.97	53.5	6.8e-15	S-adenosylmethionine decarboxylase [EC:4.1.1.50]
CP049783.1_289	K00027	507.30	574.1	1.6e-172	malate dehydrogenase (oxaloacetate-decarboxylating) [EC:1.1.1.38]
CP049783.1_291	K07025	112.47	128.2	1.2e-37	putative hydrolase of the HAD superfamily
CP049783.1_299	K09125	29.70	198.3	6.1e-59	queuosine precursor transporter
CP049783.1_300	K06956	334.43	611.2	2.6e-184	uncharacterized protein
CP049783.1_301	K02757	642.13	785.5	1.3e-236	beta-glucoside PTS system EIICBA component [EC:2.7.1.-]
CP049783.1_302	K03488	318.67	391.4	1.1e-117	beta-glucoside operon transcriptional antiterminator
CP049783.1_304	K03406	65.50	269.1	2.9e-80	methyl-accepting chemotaxis protein
CP049783.1_305	K02232	242.13	790.9	5.6e-238	adenosylcobyric acid synthase [EC:6.3.5.10]
CP049783.1_306	K02233	34.73	196.5	2.5e-58	adenosylcobinamide-GDP ribazoletransferase [EC:2.7.8.26]
CP049783.1_307	K02231	39.03	240.3	1.2e-71	adenosylcobinamide kinase / adenosylcobinamide-phosphate guanylyltransferase [EC:2.7.1.156 2.7.7.62]
CP049783.1_308	K00768	85.40	522.0	5.8e-157	nicotinate-nucleotide--dimethylbenzimidazole phosphoribosyltransferase [EC:2.4.2.21]
CP049783.1_309	K02013	238.30	328.5	2.1e-98	iron complex transport system ATP-binding protein [EC:7.2.2.-]
CP049783.1_310	K25027	379.47	411.1	1.4e-123	cobalamin transport system permease protein
CP049783.1_310	K02015	340.73	369.6	8.8e-111	iron complex transport system permease protein
CP049783.1_311	K25034	226.00	321.4	2.2e-96	cobalamin transport system substrate-binding protein

CP049783.1_311	K02016	154.03	227.6	9.1e-68	iron complex transport system substrate-binding protein
CP049783.1_312	K02226	154.57	179.2	5.3e-53	alpha-ribazole phosphatase [EC:3.1.3.73]
CP049783.1_313	K02227	90.90	411.7	9.6e-124	adenosylcobinamide-phosphate synthase [EC:6.3.1.10]
CP049783.1_314	K04720	316.27	427.7	1.7e-128	threonine-phosphate decarboxylase [EC:4.1.1.81]
CP049783.1_314	K00817	293.33	301.5	4.1e-90	histidinol-phosphate aminotransferase [EC:2.6.1.9]
CP049783.1_315	K03800	254.90	336.6	1.1e-100	lipoate---protein ligase [EC:6.3.1.20]
CP049783.1_316	K21064	166.97	213.5	1.3e-63	5-amino-6-(5-phospho-D-ribitylamino)uracil phosphatase [EC:3.1.3.104]
CP049783.1_319	K06167	156.33	241.5	4.4e-72	phosphoribosyl 1,2-cyclic phosphate phosphodiesterase [EC:3.1.4.55]
CP049783.1_320	K02045	442.53	488.1	1.1e-146	sulfate/thiosulfate transport system ATP-binding protein [EC:7.3.2.3]
CP049783.1_324	K01810	196.17	297.8	6.3e-89	glucose-6-phosphate isomerase [EC:5.3.1.9]
CP049783.1_326	K09913	55.00	141.5	3e-42	purine/pyrimidine-nucleoside phosphorylase [EC:2.4.2.1 2.4.2.2]
CP049783.1_327	K00615	214.20	577.8	1.3e-173	transketolase [EC:2.2.1.1]
CP049783.1_328	K01433	204.20	460.8	1.1e-138	formyltetrahydrofolate deformylase [EC:3.5.1.10]
CP049783.1_329	K01151	120.17	380.7	2.5e-114	deoxyribonuclease IV [EC:3.1.21.2]
CP049783.1_330	K07071	116.47	394.3	4e-118	uncharacterized protein
CP049783.1_332	K02278	85.57	107.0	3.7e-31	prepilin peptidase CpaA [EC:3.4.23.43]
CP049783.1_337	K12511	139.07	161.8	6.7e-48	tight adherence protein C
CP049783.1_338	K12510	83.50	117.3	2.3e-34	tight adherence protein B
CP049783.1_339	K02283	326.03	518.8	5e-156	pilus assembly protein CpaF [EC:7.4.2.8]
CP049783.1_342	K07646	228.07	485.9	8.9e-146	two-component system, OmpR family, sensor histidine kinase KdpD [EC:2.7.13.3]
CP049783.1_343	K11709	296.33	407.6	2.2e-122	manganese/zinc/iron transport system permease protein
CP049783.1_344	K11708	301.47	437.2	2.1e-131	manganese/zinc/iron transport system permease protein
CP049783.1_345	K11710	355.80	402.0	3.1e-121	manganese/zinc/iron transport system ATP- binding protein [EC:7.2.2.5]
CP049783.1_345	K09820	339.27	347.3	2.6e-104	manganese/iron transport system ATP-binding protein
CP049783.1_345	K09817	271.90	280.4	8.2e-84	zinc transport system ATP-binding protein [EC:7.2.2.20]
CP049783.1_345	K02013	238.30	269.9	1.2e-80	iron complex transport system ATP-binding protein [EC:7.2.2.-]
CP049783.1_346	K11707	324.40	452.4	3.2e-136	manganese/zinc/iron transport system substrate-binding protein
CP049783.1_350	K04063	57.63	201.6	4.9e-60	lipoyl-dependent peroxiredoxin [EC:1.11.1.28]
CP049783.1_351	K03322	514.07	648.0	2.9e-195	manganese transport protein
CP049783.1_354	K01548	33.50	268.6	1.3e-80	potassium-transporting ATPase KdpC subunit
CP049783.1_355	K01547	363.10	1146.5	0	potassium-transporting ATPase ATP-binding subunit [EC:7.2.2.6]
CP049783.1_356	K01546	126.87	828.9	8.3e-250	potassium-transporting ATPase potassium-binding subunit
CP049783.1_359	K00432	54.07	265.8	1.2e-79	glutathione peroxidase [EC:1.11.1.9]
CP049783.1_360	K03188	67.90	105.6	1e-30	urease accessory protein
CP049783.1_364	K22103	255.30	343.3	2.2e-103	DeoR family transcriptional regulator, carbon catabolite repression regulator
CP049783.1_365	K21064	166.97	217.6	7.5e-65	5-amino-6-(5-phospho-D-ribitylamino)uracil phosphatase [EC:3.1.3.104]
CP049783.1_366	K03095	90.87	225.2	1.2e-67	SprT-like protein
CP049783.1_367	K06959	437.10	1086.2	0	protein Tex
CP049783.1_368	K07171	19.10	99.5	9.3e-29	mRNA interferase MazF [EC:3.1.-.-]
CP049783.1_369	K07723	41.50	129.9	1.4e-38	CopG family transcriptional regulator / antitoxin EndoAI
CP049783.1_370	K01775	106.13	427.0	2.4e-128	alanine racemase [EC:5.1.1.1]
CP049783.1_374	K21701	333.73	470.7	1.9e-141	AraC family transcriptional regulator, transcriptional activator for feuABC-ybbA operon
CP049783.1_377	K02483	242.00	251.2	6.7e-75	two-component system, OmpR family, response regulator
CP049783.1_379	K01990	262.37	264.7	5e-79	ABC-2 type transport system ATP-binding protein
CP049783.1_382	K03406	65.50	276.9	1.2e-82	methyl-accepting chemotaxis protein
CP049783.1_383	K03929	467.47	544.9	8.9e-164	para-nitrobenzyl esterase [EC:3.1.1.-]
CP049783.1_384	K22109	186.30	234.7	2.1e-70	HTH-type transcriptional regulator, glycine betaine synthesis regulator
CP049783.1_385	K02000	415.90	653.5	1.6e-196	glycine betaine/proline transport system ATP-binding protein [EC:7.6.2.9]
CP049783.1_386	K02001	326.57	1152.3	0	glycine betaine/proline transport system permease protein
CP049783.1_386	K02002	122.57	374.9	2.4e-112	glycine betaine/proline transport system substrate-binding protein
CP049783.1_390	K05813	322.40	542.7	2.6e-163	sn-glycerol 3-phosphate transport system substrate-binding protein
CP049783.1_390	K02027	193.17	257.4	1.1e-76	multiple sugar transport system substrate-binding protein
CP049783.1_391	K05815	322.70	342.5	5.6e-103	sn-glycerol 3-phosphate transport system permease protein
CP049783.1_391	K02026	280.30	313.8	4.4e-94	multiple sugar transport system permease protein
CP049783.1_392	K05814	358.43	414.6	1.1e-124	sn-glycerol 3-phosphate transport system permease protein
CP049783.1_392	K02025	276.90	354.0	2.6e-106	multiple sugar transport system permease protein

CP049783.1_395	K22278	73.27	262.7	2e-78	peptidoglycan-N-acetylglucosamine deacetylase [EC:3.5.1.104]
CP049783.1_401	K05834	168.17	173.8	1.2e-51	homoserine/homoserine lactone efflux protein
CP049783.1_405	K07813	47.80	106.6	3.3e-31	accessory gene regulator B
CP049783.1_416	K04096	28.87	243.1	2.4e-72	DNA processing protein
CP049783.1_417	K02242	65.47	71.6	2.5e-20	competence protein ComFC
CP049783.1_420	K01447	118.50	138.3	1.1e-40	N-acetylmuramoyl-L-alanine amidase [EC:3.5.1.28]
CP049783.1_444	K01358	76.73	89.3	8.9e-26	ATP-dependent Clp protease, protease subunit [EC:3.4.21.92]
CP049783.1_454	K07451	41.70	45.8	1.4e-12	5-methylcytosine-specific restriction enzyme A [EC:3.1.21.-]
CP049783.1_459	K24072	90.50	203.8	4.5e-61	positive control factor
CP049783.1_473	K01160	31.43	95.4	1.4e-27	crossover junction endodeoxyribonuclease RusA [EC:3.1.21.10]
CP049783.1_476	K02315	147.80	149.3	5.9e-44	DNA replication protein DnaC
CP049783.1_479	K03111	28.83	153.1	4.3e-45	single-strand DNA-binding protein
CP049783.1_490	K04077	136.23	885.7	5.6e-267	chaperonin GroEL [EC:5.6.1.7]
CP049783.1_491	K04078	31.27	147.0	1.7e-43	chaperonin GroES
CP049783.1_492	K03118	86.40	246.3	1.8e-73	sec-independent protein translocase protein TatC
CP049783.1_493	K03116	54.03	70.0	8.4e-20	sec-independent protein translocase protein TatA
CP049783.1_494	K03831	218.90	219.9	1.8e-65	molybdopterin adenylyltransferase [EC:2.7.7.75]
CP049783.1_495	K03637	63.27	267.0	8.7e-80	cyclic pyranopterin monophosphate synthase [EC:4.6.1.17]
CP049783.1_496	K01934	93.97	172.8	3.5e-51	5-formyltetrahydrofolate cyclo-ligase [EC:6.3.3.2]
CP049783.1_497	K06158	558.03	662.7	2.4e-199	ATP-binding cassette, subfamily F, member 3
CP049783.1_498	K25706	413.33	509.2	4.1e-153	tRNA N6-adenosine threonylcarbamoyltransferase [EC:2.3.1.234]
CP049783.1_499	K03789	102.70	182.0	4.3e-54	[ribosomal protein S18]-alanine N-acetyltransferase [EC:2.3.1.266]
CP049783.1_501	K14742	83.37	109.0	9.9e-32	tRNA threonylcarbamoyladenosine biosynthesis protein TsaB
CP049783.1_502	K06925	60.83	163.6	2.5e-48	tRNA threonylcarbamoyladenosine biosynthesis protein TsaE
CP049783.1_504	K00626	488.13	558.7	5.1e-168	acetyl-CoA C-acetyltransferase [EC:2.3.1.9]
CP049783.1_505	K01574	109.27	345.8	3.7e-104	acetoacetate decarboxylase [EC:4.1.1.4]
CP049783.1_506	K01034	300.93	316.2	3e-95	acetate CoA/acetoacetate CoA-transferase alpha subunit [EC:2.8.3.8 2.8.3.9]
CP049783.1_507	K01035	312.93	316.7	2e-95	acetate CoA/acetoacetate CoA-transferase beta subunit [EC:2.8.3.8 2.8.3.9]
CP049783.1_508	K06425	45.60	78.2	1e-22	small acid-soluble spore protein H (minor)
CP049783.1_510	K10979	98.23	393.6	2.6e-118	DNA end-binding protein Ku
CP049783.1_511	K06880	109.97	378.8	1.6e-113	erythromycin esterase [EC:3.1.1.-]
CP049783.1_518	K01953	106.30	283.2	1.6e-84	asparagine synthase (glutamine-hydrolysing) [EC:6.3.5.4]
CP049783.1_519	K01021	89.10	91.4	1.5e-26	protein-tyrosine sulfotransferase [EC:2.8.2.20]
CP049783.1_520	K01971	209.27	283.7	1.3e-84	bifunctional non-homologous end joining protein LigD [EC:6.5.1.1]
CP049783.1_522	K06131	262.77	494.1	2.2e-148	cardiolipin synthase A/B [EC:2.7.8.-]
CP049783.1_523	K19304	164.03	174.1	1.7e-51	murein DD-endopeptidase [EC:3.4.24.-]
CP049783.1_527	K01990	262.37	309.0	1.8e-92	ABC-2 type transport system ATP-binding protein
CP049783.1_529	K01005	193.97	286.1	1.9e-85	polyisoprenyl-teichoic acid--peptidoglycan teichoic acid transferase [EC:2.7.8.-]
CP049783.1_530	K00012	358.77	623.0	1.4e-187	UDPglucose 6-dehydrogenase [EC:1.1.1.22]
CP049783.1_531	K05946	235.07	336.2	1.1e-100	N-acetylglucosaminyldiphosphoundecaprenol N-acetyl-beta-D-mannosaminyltransferase [EC:2.4.1.187]
CP049783.1_536	K01711	282.83	596.9	2.2e-179	GDPmannose 4,6-dehydratase [EC:4.2.1.47]
CP049783.1_537	K02377	232.90	509.6	1.8e-153	GDP-L-fucose synthase [EC:1.1.1.271]
CP049783.1_538	K16566	223.20	237.9	2.2e-71	exopolysaccharide production protein ExoY
CP049783.1_539	K00903	250.43	281.9	2.6e-84	protein-tyrosine kinase [EC:2.7.10.3]
CP049783.1_540	K19420	201.30	239.5	1.3e-71	protein tyrosine kinase modulator
CP049783.1_544	K01218	70.20	319.2	2.1e-95	mannan endo-1,4-beta-mannosidase [EC:3.2.1.78]
CP049783.1_545	K00971	203.67	335.1	3.1e-100	mannose-1-phosphate guanylyltransferase [EC:2.7.7.13]
CP049783.1_546	K00012	358.77	614.4	5.8e-185	UDPglucose 6-dehydrogenase [EC:1.1.1.22]
CP049783.1_553	K05946	235.07	330.2	7.7e-99	N-acetylglucosaminyldiphosphoundecaprenol N-acetyl-beta-D-mannosaminyltransferase [EC:2.4.1.187]
CP049783.1_555	K22844	181.20	257.0	9.6e-77	glucosyl-dolichyl phosphate glucuronosyltransferase [EC:2.4.1.356]
CP049783.1_556	K16566	223.20	233.6	4.3e-70	exopolysaccharide production protein ExoY
CP049783.1_557	K00963	76.77	151.1	1.5e-44	UTP--glucose-1-phosphate uridylyltransferase [EC:2.7.7.9]
CP049783.1_559	K19420	201.30	211.5	4.4e-63	protein tyrosine kinase modulator
CP049783.1_562	K00265	1712.27	2344.5	0	glutamate synthase (NADPH) large chain [EC:1.4.1.13]
CP049783.1_564	K01449	48.60	170.4	1.5e-50	cell wall hydrolase
CP049783.1_565	K01975	64.70	154.9	9.1e-46	RNA 2',3'-cyclic 3'-phosphodiesterase [EC:3.1.4.58]

CP049783.1_567	K07446	165.90	205.2	5.2e-61	tRNA (guanine10-N2)-dimethyltransferase [EC:2.1.1.213]
CP049783.1_569	K01129	92.73	352.2	2e-105	dGTPase [EC:3.1.5.1]
CP049783.1_570	K02034	256.80	403.3	4.6e-121	peptide/nickel transport system permease protein
CP049783.1_571	K02033	263.63	428.8	9e-129	peptide/nickel transport system permease protein
CP049783.1_572	K02035	249.67	482.9	5.4e-145	peptide/nickel transport system substrate-binding protein
CP049783.1_572	K15580	368.50	380.3	7.1e-114	oligopeptide transport system substrate-binding protein
CP049783.1_575	K00645	247.17	363.9	5.7e-109	[acyl-carrier-protein] S-malonyltransferase [EC:2.3.1.39]
CP049783.1_578	K00612	190.27	375.4	2.1e-112	carbamoyltransferase [EC:2.1.3.-]
CP049783.1_584	K06936	152.70	369.0	7.1e-111	archaeosine synthase beta-subunit [EC:2.6.1.-]
CP049783.1_587	K03701	341.57	1176.1	0	excinuclease ABC subunit A
CP049783.1_588	K01286	175.57	194.5	1.3e-57	D-alanyl-D-alanine carboxypeptidase [EC:3.4.16.4]
CP049783.1_590	K07273	48.73	274.8	3.1e-82	lysozyme
CP049783.1_607	K05593	117.83	372.4	1.1e-111	aminoglycoside 6-adenylyltransferase [EC:2.7.7.-]
CP049783.1_609	K22299	76.73	139.6	3e-41	HTH-type transcriptional regulator, competence development regulator
CP049783.1_611	K11751	484.37	628.1	5.2e-189	5'-nucleotidase / UDP-sugar diphosphatase [EC:3.1.3.5 3.6.1.45]
CP049783.1_611	K01081	158.63	182.8	4.8e-54	5'-nucleotidase [EC:3.1.3.5]
CP049783.1_618	K06920	96.20	317.4	3e-95	7-cyano-7-deazaguanine synthase [EC:6.3.4.20]
CP049783.1_619	K01737	24.80	132.8	4.6e-39	6-pyruvoyltetrahydropterin/6-carboxytetrahydropterin synthase [EC:4.2.3.12 4.1.2.50]
CP049783.1_620	K10026	81.40	197.4	8.3e-59	7-carboxy-7-deazaguanine synthase [EC:4.3.99.3]
CP049783.1_621	K09457	104.03	236.8	5.9e-71	7-cyano-7-deazaguanine reductase [EC:1.7.1.13]
CP049783.1_623	K01193	187.77	414.3	2.7e-124	beta-fructofuranosidase [EC:3.2.1.26]
CP049783.1_624	K05349	305.00	659.8	2.3e-198	beta-glucosidase [EC:3.2.1.21]
CP049783.1_625	K02810	596.80	640.5	1.3e-192	sucrose PTS system EIIBCA or EIIBC component [EC:2.7.1.211]
CP049783.1_629	K08223	154.93	479.3	3.7e-144	MFS transporter, FSR family, fosmidomycin resistance protein
CP049783.1_635	K10547	451.33	607.2	4.3e-183	putative multiple sugar transport system permease protein
CP049783.1_636	K10548	753.37	874.2	6.8e-264	putative multiple sugar transport system ATP-binding protein [EC:7.5.2.-]
CP049783.1_637	K10546	363.97	574.3	6.1e-173	putative multiple sugar transport system substrate-binding protein
CP049783.1_638	K07727	44.53	67.1	5.3e-19	putative transcriptional regulator
CP049783.1_639	K12132	224.03	227.2	8e-68	eukaryotic-like serine/threonine-protein kinase [EC:2.7.11.1]
CP049783.1_644	K05601	103.77	659.7	1e-198	hydroxylamine reductase [EC:1.7.99.1]
CP049783.1_645	K07720	156.87	336.2	1.4e-100	two-component system, response regulator YesN
CP049783.1_646	K07718	273.17	525.3	1e-157	two-component system, sensor histidine kinase YesM [EC:2.7.13.3]
CP049783.1_647	K10439	190.90	241.8	6.1e-72	ribose transport system substrate-binding protein
CP049783.1_649	K06376	38.70	38.8	2.3e-10	stage 0 sporulation regulatory protein
CP049783.1_657	K07697	304.97	436.8	3.6e-131	two-component system, sporulation sensor kinase B [EC:2.7.13.3]
CP049783.1_661	K13853	465.03	594.4	2.2e-179	3-deoxy-7-phosphoheptulonate synthase / chorismate mutase [EC:2.5.1.54 5.4.99.5]
CP049783.1_663	K23356	46.03	181.9	5.6e-54	HTH-type transcriptional regulator, sugar sensing transcriptional regulator
CP049783.1_666	K08161	244.40	460.3	1.9e-138	MFS transporter, DHA1 family, multidrug resistance protein
CP049783.1_669	K03768	202.90	222.3	2.4e-66	peptidyl-prolyl cis-trans isomerase B (cyclophilin B) [EC:5.2.1.8]
CP049783.1_670	K07507	42.27	215.0	5e-64	putative Mg2+ transporter-C (MgtC) family protein
CP049783.1_675	K03406	65.50	180.8	1.6e-53	methyl-accepting chemotaxis protein
CP049783.1_676	K03699	305.47	426.3	5.5e-128	magnesium and cobalt exporter, CNNM family
CP049783.1_681	K09684	188.00	383.3	8.3e-115	PucR family transcriptional regulator, purine catabolism regulatory protein
CP049783.1_682	K00259	287.10	586.4	1e-176	alanine dehydrogenase [EC:1.4.1.1]
CP049783.1_683	K03649	188.13	199.5	2.3e-59	double-stranded uracil-DNA glycosylase [EC:3.2.2.28]
CP049783.1_686	K02040	56.17	157.6	1.8e-46	phosphate transport system substrate-binding protein
CP049783.1_688	K16919	63.27	399.6	1e-119	acetoin utilization transport system permease protein
CP049783.1_689	K16921	296.27	423.7	9.1e-128	acetoin utilization transport system ATP-binding protein
CP049783.1_690	K07979	106.93	159.8	1.4e-47	GntR family transcriptional regulator
CP049783.1_692	K19668	224.60	935.3	1.3e-281	cellulose 1,4-beta-cellobiosidase [EC:3.2.1.91]
CP049783.1_693	K07406	369.43	591.9	4.7e-178	alpha-galactosidase [EC:3.2.1.22]
CP049783.1_694	K10439	190.90	198.4	8.4e-59	ribose transport system substrate-binding protein
CP049783.1_695	K07718	273.17	322.2	2.7e-96	two-component system, sensor histidine kinase YesM [EC:2.7.13.3]
CP049783.1_696	K07720	156.87	327.6	6e-98	two-component system, response regulator YesN
CP049783.1_697	K02100	553.43	612.0	2.3e-184	MFS transporter, SP family, arabinose:H+ symporter
CP049783.1_700	K08153	415.17	477.1	1.1e-143	MFS transporter, DHA1 family, multidrug resistance protein

CP049783.1_701	K18888	717.83	853.2	5.8e-257	ATP-binding cassette, subfamily B, multidrug efflux pump
CP049783.1_701	K06147	612.93	641.3	7.2e-193	ATP-binding cassette, subfamily B, bacterial
CP049783.1_702	K18887	708.60	747.7	4.6e-225	ATP-binding cassette, subfamily B, multidrug efflux pump
CP049783.1_703	K01212	646.57	968.6	1.5e-291	levanase [EC:3.2.1.65]
CP049783.1_703	K03332	524.40	792.0	2.9e-238	fructan beta-fructosidase [EC:3.2.1.80]
CP049783.1_703	K01193	187.77	345.2	2.3e-103	beta-fructofuranosidase [EC:3.2.1.26]
CP049783.1_704	K03332	524.40	777.3	8.2e-234	fructan beta-fructosidase [EC:3.2.1.80]
CP049783.1_704	K01193	187.77	420.3	4.3e-126	beta-fructofuranosidase [EC:3.2.1.26]
CP049783.1_705	K25670	672.53	719.3	1.1e-216	fructooligosaccharide transport system substrate-binding protein
CP049783.1_705	K17318	176.50	309.8	1.4e-92	putative aldouronate transport system substrate-binding protein
CP049783.1_706	K25672	433.47	444.5	4.4e-134	fructooligosaccharide transport system permease protein
CP049783.1_706	K17320	270.87	341.0	1.7e-102	putative aldouronate transport system permease protein
CP049783.1_707	K25671	470.77	531.8	1.6e-160	fructooligosaccharide transport system permease protein
CP049783.1_707	K17319	220.03	406.5	3.3e-122	putative aldouronate transport system permease protein
CP049783.1_709	K18198	1008.50	1031.6	5.6e-311	rhamnogalacturonan exolyase [EC:4.2.2.24]
CP049783.1_709	K18197	100.33	870.9	2.5e-262	rhamnogalacturonan endolyase [EC:4.2.2.23]
CP049783.1_710	K03088	96.50	114.1	2.2e-33	RNA polymerase sigma-70 factor, ECF subfamily
CP049783.1_714	K07406	369.43	753.5	6.2e-227	alpha-galactosidase [EC:3.2.1.22]
CP049783.1_717	K18908	503.07	645.3	1.5e-194	MATE family, multidrug efflux pump
CP049783.1_717	K26937	267.43	312.4	1.4e-93	MATE family, multidrug efflux pump
CP049783.1_721	K06878	128.33	141.3	9e-42	tRNA-binding protein
CP049783.1_724	K02028	386.70	430.0	4e-129	polar amino acid transport system ATP-binding protein [EC:7.4.2.1]
CP049783.1_725	K02029	214.53	264.7	4.8e-79	polar amino acid transport system permease protein
CP049783.1_726	K02030	76.77	186.7	2.3e-55	polar amino acid transport system substrate-binding protein
CP049783.1_727	K15581	345.63	386.2	5.2e-116	oligopeptide transport system permease protein
CP049783.1_727	K02033	263.63	361.8	1.8e-108	peptide/nickel transport system permease protein
CP049783.1_728	K15582	364.50	408.4	5.5e-123	oligopeptide transport system permease protein
CP049783.1_728	K02034	256.80	369.0	1.1e-110	peptide/nickel transport system permease protein
CP049783.1_730	K15580	368.50	593.3	2.3e-178	oligopeptide transport system substrate-binding protein
CP049783.1_730	K02035	249.67	370.8	4.3e-111	peptide/nickel transport system substrate-binding protein
CP049783.1_744	K05792	212.93	521.2	4.9e-157	tellurite resistance protein TerA
CP049783.1_744	K05795	235.30	236.2	1.9e-70	tellurium resistance protein TerD
CP049783.1_745	K05795	235.30	314.7	2.6e-94	tellurium resistance protein TerD
CP049783.1_746	K05795	235.30	325.5	1.3e-97	tellurium resistance protein TerD
CP049783.1_747	K05795	235.30	249.4	1.8e-74	tellurium resistance protein TerD
CP049783.1_748	K05793	69.17	211.4	5.3e-63	tellurite resistance protein TerB
CP049783.1_750	K01990	262.37	295.4	2.4e-88	ABC-2 type transport system ATP-binding protein
CP049783.1_753	K08217	225.00	249.2	1.9e-74	MFS transporter, DHA3 family, macrolide efflux protein
CP049783.1_758	K15661	3755.90	4232.4	0	iturin family lipopeptide synthetase A
CP049783.1_758	K01845	312.23	425.7	1.2e-127	glutamate-1-semialdehyde 2,1-aminomutase [EC:5.4.3.8]
CP049783.1_759	K00645	247.17	342.1	2.3e-102	[acyl-carrier-protein] S-malonyltransferase [EC:2.3.1.39]
CP049783.1_762	K02598	241.60	350.1	1.8e-105	nitrite transporter
CP049783.1_764	K23598	84.67	225.0	3.1e-67	iseA protein
CP049783.1_766	K18197	100.33	1073.6	0	rhamnogalacturonan endolyase [EC:4.2.2.23]
CP049783.1_775	K01201	204.70	493.5	2.8e-148	glucosylceramidase [EC:3.2.1.45]
CP049783.1_776	K05349	305.00	845.0	2.8e-254	beta-glucosidase [EC:3.2.1.21]
CP049783.1_777	K00078	265.20	420.5	3.6e-126	dihydrodiol dehydrogenase / D-xylose 1-dehydrogenase (NADP) [EC:1.3.1.20 1.1.1.179]
CP049783.1_777	K22230	271.20	273.3	1.1e-81	scyllo-inositol 2-dehydrogenase (NADP+) [EC:1.1.1.-]
CP049783.1_781	K02073	57.57	311.3	4.1e-93	D-methionine transport system substrate-binding protein
CP049783.1_782	K02072	119.67	305.7	9e-92	D-methionine transport system permease protein
CP049783.1_783	K02071	332.87	408.1	1.7e-122	D-methionine transport system ATP-binding protein
CP049783.1_784	K16785	103.27	163.2	2.3e-48	energy-coupling factor transport system permease protein
CP049783.1_785	K01552	403.80	526.3	3.7e-158	energy-coupling factor transport system ATP-binding protein [EC:7.-.-.-]
CP049783.1_786	K16925	57.77	210.8	5.1e-63	energy-coupling factor transport system permease protein
CP049783.1_787	K03424	86.70	307.1	7.1e-92	TatD DNase family protein [EC:3.1.21.-]
CP049783.1_789	K20798	86.27	167.0	2.4e-49	small RNA 2'-O-methyltransferase [EC:2.1.1.386]

CP049783.1_790	K07074	59.80	278.5	2.5e-83	uncharacterized protein
CP049783.1_797	K07707	139.50	249.2	1.2e-74	two-component system, LytTR family, response regulator AgrA
CP049783.1_798	K07706	112.00	269.0	2.2e-80	two-component system, LytTR family, sensor histidine kinase AgrC [EC:2.7.13.3]
CP049783.1_800	K07813	47.80	127.5	1.3e-37	accessory gene regulator B
CP049783.1_801	K18104	642.17	658.1	4.8e-198	ATP-binding cassette, subfamily B, bacterial AbcA/BmrA [EC:7.6.2.2]
CP049783.1_803	K20484	130.10	229.7	2.4e-68	class I lanthipeptide synthase [EC:3.13.2.4]
CP049783.1_804	K20483	108.60	626.0	4.2e-188	class I lanthipeptide synthase [EC:3.13.2.4]
CP049783.1_807	K01738	426.93	460.3	2.4e-138	cysteine synthase [EC:2.5.1.47]
CP049783.1_808	K19577	416.40	476.8	2.3e-143	MFS transporter, DHA1 family, inner membrane transport protein
CP049783.1_811	K19551	226.80	344.2	7.5e-104	pectate lyase C [EC:4.2.2.2 4.2.2.10]
CP049783.1_812	K00891	128.77	148.8	9.7e-44	shikimate kinase [EC:2.7.1.71]
CP049783.1_813	K03785	182.93	312.1	2.6e-93	3-dehydroquinate dehydratase I [EC:4.2.1.10]
CP049783.1_814	K26745	212.40	465.3	3.7e-140	MFS transporter, AAHS family, multidrug transporter
CP049783.1_816	K26745	212.40	473.0	1.8e-142	MFS transporter, AAHS family, multidrug transporter
CP049783.1_818	K14415	269.37	346.9	8.6e-104	tRNA-splicing ligase RtcB (3'-phosphate/5'-hydroxy nucleic acid ligase) [EC:6.5.1.8]
CP049783.1_819	K22390	122.97	178.4	7.2e-53	acid phosphatase type 7
CP049783.1_820	K26141	392.27	409.9	5.8e-123	Xaa-Arg dipeptidase [EC:3.4.13.4]
CP049783.1_821	K02073	57.57	322.0	2.5e-96	D-methionine transport system substrate-binding protein
CP049783.1_822	K02072	119.67	312.0	1.1e-93	D-methionine transport system permease protein
CP049783.1_823	K02071	332.87	502.5	3.8e-151	D-methionine transport system ATP-binding protein
CP049783.1_824	K22230	271.20	506.2	2.7e-152	scyllo-inositol 2-dehydrogenase (NADP+) [EC:1.1.1.-]
CP049783.1_825	K15519	203.27	305.8	8e-92	deoxyadenosine/deoxycytidine kinase [EC:2.7.1.76 2.7.1.74]
CP049783.1_826	K15518	191.63	316.9	2.1e-95	deoxyguanosine kinase [EC:2.7.1.113]
CP049783.1_827	K07693	228.97	295.8	1.2e-88	two-component system, NarL family, response regulator DesR
CP049783.1_828	K07778	170.90	294.7	4.1e-88	two-component system, NarL family, sensor histidine kinase DesK [EC:2.7.13.3]
CP049783.1_829	K10255	236.43	439.3	4.1e-132	acyl-lipid omega-6 desaturase (Delta-12 desaturase) [EC:1.14.19.23 1.14.19.45]
CP049783.1_831	K01500	203.23	226.0	1.2e-67	methenyltetrahydrofolate cyclohydrolase [EC:3.5.4.9]
CP049783.1_834	K25308	264.57	354.6	1.5e-106	ferric hydroxamate/heme transport system substrate-binding protein
CP049783.1_834	K02016	154.03	157.1	2.2e-46	iron complex transport system substrate-binding protein
CP049783.1_839	K21745	110.70	128.4	5.4e-38	MerR family transcriptional regulator, aldehyde-responsive regulator
CP049783.1_840	K13979	530.93	609.8	7e-184	alcohol dehydrogenase (NADP+) [EC:1.1.1.2]
CP049783.1_841	K03293	609.50	632.2	2.6e-190	amino acid transporter, AAT family
CP049783.1_842	K00059	269.80	273.0	1.4e-81	3-oxoacyl-[acyl-carrier protein] reductase [EC:1.1.1.100]
CP049783.1_843	K08997	199.53	692.7	2.8e-208	protein adenylyltransferase [EC:2.7.7.108]
CP049783.1_844	K04750	56.87	76.8	6.6e-22	PhnB protein
CP049783.1_847	K15534	957.87	1381.3	0	beta-D-galactosyl-(1->4)-L-rhamnose phosphorylase [EC:2.4.1.247]
CP049783.1_848	K15532	93.77	260.3	1.2e-77	unsaturated rhamnogalacturonyl hydrolase [EC:3.2.1.172]
CP049783.1_854	K03427	116.20	423.6	5.4e-127	type I restriction enzyme M protein [EC:2.1.1.72]
CP049783.1_855	K01154	44.73	156.7	3e-46	type I restriction enzyme, S subunit [EC:3.1.21.3]
CP049783.1_857	K01153	169.67	497.1	4.7e-149	type I restriction enzyme, R subunit [EC:3.1.21.3]
CP049783.1_859	K19092	34.90	63.8	6.6e-18	toxin ParE1/3/4
CP049783.1_860	K03427	116.20	211.5	8.6e-63	type I restriction enzyme M protein [EC:2.1.1.72]
CP049783.1_862	K03215	301.40	713.1	9.8e-215	23S rRNA (uracil1939-C5)-methyltransferase [EC:2.1.1.190]
CP049783.1_863	K07029	147.20	306.2	9.5e-92	diacylglycerol kinase (ATP) [EC:2.7.1.107]
CP049783.1_867	K03284	165.70	311.4	3.7e-93	magnesium transporter
CP049783.1_868	K02028	386.70	469.2	4.9e-141	polar amino acid transport system ATP-binding protein [EC:7.4.2.1]
CP049783.1_868	K23060	426.27	430.3	1.7e-129	arginine/lysine/histidine transport system ATP-binding protein [EC:7.4.2.1]
CP049783.1_868	K10038	397.10	412.3	1.4e-124	glutamine transport system ATP-binding protein [EC:7.4.2.1]
CP049783.1_869	K10036	469.53	756.0	6.7e-228	glutamine transport system substrate-binding protein
CP049783.1_869	K10037	544.33	751.8	8.7e-227	glutamine transport system permease protein
CP049783.1_869	K02029	214.53	304.1	5.3e-91	polar amino acid transport system permease protein
CP049783.1_869	K02030	76.77	165.4	6.7e-49	polar amino acid transport system substrate-binding protein
CP049783.1_870	K03306	385.20	461.9	9e-139	inorganic phosphate transporter, PiT family
CP049783.1_871	K07220	65.73	203.6	1.5e-60	uncharacterized protein
CP049783.1_873	K16937	70.10	112.5	6.5e-33	thiosulfate dehydrogenase (quinone) large subunit [EC:1.8.5.2]
CP049783.1_875	K15777	105.60	337.2	3.9e-101	4,5-DOPA dioxygenase extradiol [EC:1.13.11.-]

CP049783.1_876	K06889	113.67	140.1	2.7e-41	uncharacterized protein
CP049783.1_880	K25289	386.53	432.4	3.4e-130	ferric hydroxamate/heme transport system permease protein
CP049783.1_881	K25288	404.60	432.1	4e-130	ferric hydroxamate/heme transport system permease protein
CP049783.1_882	K06113	228.80	346.8	7.3e-104	arabinan endo-1,5-alpha-L-arabinosidase [EC:3.2.1.99]
CP049783.1_883	K01754	266.27	468.1	1.3e-140	threonine dehydratase [EC:4.3.1.19]
CP049783.1_884	K17236	335.93	417.7	6.5e-126	arabinooligosaccharide transport system permease protein
CP049783.1_884	K02026	280.30	295.3	1.9e-88	multiple sugar transport system permease protein
CP049783.1_885	K17235	341.27	447.3	8.7e-135	arabinooligosaccharide transport system permease protein
CP049783.1_885	K02025	276.90	294.6	2.9e-88	multiple sugar transport system permease protein
CP049783.1_886	K17234	307.53	617.5	3.2e-186	arabinooligosaccharide transport system substrate-binding protein
CP049783.1_887	K06434	40.27	94.2	1.1e-27	small acid-soluble spore protein (thioredoxin-like protein)
CP049783.1_888	K06999	93.57	167.7	1.2e-49	phospholipase/carboxylesterase
CP049783.1_889	K26993	108.47	155.8	2.7e-46	ArsR family transcriptional regulator, repressor of sdpIR and other operons
CP049783.1_890	K26996	27.07	136.0	4.9e-40	immunity protein, SdpI family
CP049783.1_892	K23257	397.27	441.2	3.6e-133	methylglyoxal/glyoxal reductase [EC:1.1.1.283 1.1.1.-]
CP049783.1_892	K06221	349.23	424.5	6.6e-128	2,5-diketo-D-gluconate reductase A [EC:1.1.1.346]
CP049783.1_893	K11530	74.90	82.0	8.5e-24	(4S)-4-hydroxy-5-phosphonooxypentane-2,3-dione isomerase [EC:5.3.1.32]
CP049783.1_896	K23181	346.30	372.3	4.8e-112	ferric citrate transport system substrate-binding protein
CP049783.1_896	K02016	154.03	175.2	7.3e-52	iron complex transport system substrate-binding protein
CP049783.1_897	K23183	376.77	398.5	6.6e-120	ferric citrate transport system permease protein
CP049783.1_898	K23182	369.60	424.3	9.1e-128	ferric citrate transport system permease protein
CP049783.1_899	K07397	72.77	97.3	3.8e-28	putative redox protein
CP049783.1_900	K02028	386.70	426.4	4.8e-128	polar amino acid transport system ATP-binding protein [EC:7.4.2.1]
CP049783.1_901	K10009	301.87	305.5	2.2e-91	L-cystine transport system permease protein
CP049783.1_901	K02029	214.53	287.6	5.3e-86	polar amino acid transport system permease protein
CP049783.1_902	K02030	76.77	184.7	9.1e-55	polar amino acid transport system substrate-binding protein
CP049783.1_904	K01945	149.87	667.4	5.6e-201	phosphoribosylamine---glycine ligase [EC:6.3.4.13]
CP049783.1_905	K00602	115.70	806.8	4e-243	phosphoribosylaminoimidazolecarboxamide formyltransferase / IMP cyclohydrolase [EC:2.1.2.3 3.5.4.10]
CP049783.1_906	K11175	195.63	297.3	4.5e-89	phosphoribosylglycinamide formyltransferase 1 [EC:2.1.2.2]
CP049783.1_907	K01933	170.70	596.0	2.7e-179	phosphoribosylformylglycinamidine cyclo-ligase [EC:6.3.3.1]
CP049783.1_909	K00764	178.17	703.5	7e-212	amidophosphoribosyltransferase [EC:2.4.2.14]
CP049783.1_910	K23269	711.03	1179.2	0	phosphoribosylformylglycinamidine synthase subunit PurL [EC:6.3.5.3]
CP049783.1_911	K23265	206.17	365.7	7.3e-110	phosphoribosylformylglycinamidine synthase subunit PurQ / glutaminase [EC:6.3.5.3 3.5.1.2]
CP049783.1_912	K23264	41.33	122.5	6.1e-36	phosphoribosylformylglycinamidine synthase subunit PurS [EC:6.3.5.3]
CP049783.1_913	K01923	194.90	380.6	4e-114	phosphoribosylaminoimidazole-succinocarboxamide synthase [EC:6.3.2.6]
CP049783.1_915	K01756	328.93	523.2	4.4e-157	adenylosuccinate lyase [EC:4.3.2.2]
CP049783.1_916	K01589	271.73	591.2	5.6e-178	5-(carboxyamino)imidazole ribonucleotide synthase [EC:6.3.4.18]
CP049783.1_917	K01588	108.13	269.2	9.3e-81	5-(carboxyamino)imidazole ribonucleotide mutase [EC:5.4.99.18]
CP049783.1_922	K01424	50.80	373.1	8.7e-112	L-asparaginase [EC:3.5.1.1]
CP049783.1_924	K03169	533.50	825.7	1.4e-248	DNA topoisomerase III [EC:5.6.2.1]
CP049783.1_925	K00648	229.33	397.0	3.4e-119	3-oxoacyl-[acyl-carrier-protein] synthase III [EC:2.3.1.180]
CP049783.1_927	K06901	93.90	507.6	1.2e-152	adenine/guanine/hypoxanthine permease
CP049783.1_933	K05995	93.83	157.6	1.6e-46	dipeptidase E [EC:3.4.13.21]
CP049783.1_934	K01951	100.47	781.9	2.2e-235	GMP synthase (glutamine-hydrolysing) [EC:6.3.5.2]
CP049783.1_935	K03975	59.97	108.3	1.2e-31	membrane-associated protein
CP049783.1_936	K06295	452.20	745.0	3.6e-224	spore germination protein KA
CP049783.1_937	K06297	281.23	322.2	1.8e-96	spore germination protein KC
CP049783.1_938	K06296	248.90	336.5	7.5e-101	spore germination protein KB
CP049783.1_940	K06296	248.90	299.0	1.8e-89	spore germination protein KB
CP049783.1_941	K03564	157.77	217.8	7e-65	thioredoxin-dependent peroxiredoxin [EC:1.11.1.24]
CP049783.1_942	K25154	114.47	252.7	1.8e-75	viologen exporter family transport system permease protein
CP049783.1_943	K25155	105.97	242.8	2.4e-72	viologen exporter family transport system permease protein
CP049783.1_946	K01005	193.97	310.9	5.7e-93	polyisoprenyl-teichoic acid--peptidoglycan teichoic acid transferase [EC:2.7.8.-]
CP049783.1_947	K01845	312.23	614.2	9.9e-185	glutamate-1-semialdehyde 2,1-aminomutase [EC:5.4.3.8]
CP049783.1_950	K15532	93.77	257.1	1.1e-76	unsaturated rhamnogalacturonyl hydrolase [EC:3.2.1.172]
CP049783.1_952	K25678	367.67	406.3	2.2e-122	pectin-derived oligosaccharide transport system permease protein

CP049783.1_952	K02026	280.30	312.4	1.2e-93	multiple sugar transport system permease protein
CP049783.1_953	K25677	436.07	487.6	1.2e-146	pectin-derived oligosaccharide transport system permease protein
CP049783.1_953	K02025	276.90	313.7	4.6e-94	multiple sugar transport system permease protein
CP049783.1_954	K25676	343.90	521.3	6.9e-157	pectin-derived oligosaccharide transport system substrate-binding protein
CP049783.1_955	K07720	156.87	351.8	2.7e-105	two-component system, response regulator YesN
CP049783.1_956	K07718	273.17	442.3	1.3e-132	two-component system, sensor histidine kinase YesM [EC:2.7.13.3]
CP049783.1_958	K01092	211.57	332.4	1.4e-99	myo-inositol-1(or 4)-monophosphatase [EC:3.1.3.25]
CP049783.1_959	K13281	143.30	357.6	4.1e-107	UV DNA damage endonuclease [EC:3.-.-.-]
CP049783.1_961	K01921	226.63	442.3	1e-132	D-alanine-D-alanine ligase [EC:6.3.2.4]
CP049783.1_963	K27802	1411.80	1512.0	0	aconitate hydratase A / 2-methylisocitrate dehydratase [EC:4.2.1.3 4.2.1.99]
CP049783.1_966	K03147	598.13	1218.3	0	phosphomethylpyrimidine synthase [EC:4.1.99.17]
CP049783.1_968	K06889	113.67	120.2	3.1e-35	uncharacterized protein
CP049783.1_969	K04564	41.50	313.4	3.6e-94	superoxide dismutase, Fe-Mn family [EC:1.15.1.1]
CP049783.1_970	K16869	116.30	312.8	7.7e-94	octanoyl-[GcvH]:protein N-octanoyltransferase [EC:2.3.1.204]
CP049783.1_971	K01495	58.13	311.1	2.4e-93	GTP cyclohydrolase IA [EC:3.5.4.16]
CP049783.1_972	K09976	58.87	95.7	4.6e-28	uncharacterized protein
CP049783.1_973	K18979	73.50	497.6	1.4e-149	epoxyqueuosine reductase [EC:1.17.99.6]
CP049783.1_973	K00567	174.97	207.1	1.3e-61	methylated-DNA-[protein]-cysteine S-methyltransferase [EC:2.1.1.63]
CP049783.1_974	K03469	83.37	176.9	1.7e-52	ribonuclease HI [EC:3.1.26.4]
CP049783.1_975	K03100	114.77	187.9	8.4e-56	signal peptidase I [EC:3.4.21.89]
CP049783.1_977	K04564	41.50	300.9	2.3e-90	superoxide dismutase, Fe-Mn family [EC:1.15.1.1]
CP049783.1_980	K07407	52.03	185.5	6.5e-55	alpha-galactosidase [EC:3.2.1.22]
CP049783.1_981	K02099	174.60	183.3	2.4e-54	AraC family transcriptional regulator, arabinose operon regulatory protein
CP049783.1_982	K03575	149.73	466.7	2.7e-140	A/G-specific adenine glycosylase [EC:3.2.2.31]
CP049783.1_983	K00997	102.37	136.3	5e-40	holo-[acyl-carrier protein] synthase [EC:2.7.8.7]
CP049783.1_985	K01916	281.93	319.9	9.8e-96	NAD+ synthase [EC:6.3.1.5]
CP049783.1_986	K26958	151.57	222.9	4.6e-67	bacilliredoxin
CP049783.1_989	K12574	123.37	749.9	7.3e-226	ribonuclease J [EC:3.1.-.-]
CP049783.1_992	K03710	178.10	215.0	6.5e-64	GntR family transcriptional regulator
CP049783.1_993	K10710	176.40	341.4	1.1e-102	fructoselysine 6-kinase [EC:2.7.1.218]
CP049783.1_994	K10708	341.97	437.2	1.1e-131	fructoselysine 6-phosphate deglycase [EC:3.5.-.-]
CP049783.1_997	K06439	91.47	140.3	5.5e-42	similar to spore coat protein
CP049783.1_998	K06440	60.37	84.5	1.1e-24	similar to spore coat protein
CP049783.1_1000	K15973	110.17	190.8	6.5e-57	MarR family transcriptional regulator, 2-MHQ and catechol-resistance regulon repressor
CP049783.1_1001	K01212	646.57	726.4	2e-218	levanase [EC:3.2.1.65]
CP049783.1_1001	K01193	187.77	299.7	1.3e-89	beta-fructofuranosidase [EC:3.2.1.26]
CP049783.1_1002	K00692	298.30	526.8	2.3e-158	levansucrase [EC:2.4.1.10]
CP049783.1_1004	K01552	403.80	496.9	2.9e-149	energy-coupling factor transport system ATP-binding protein [EC:7.-.-.-]
CP049783.1_1005	K16925	57.77	151.6	6.1e-45	energy-coupling factor transport system permease protein
CP049783.1_1006	K16785	103.27	144.9	8.4e-43	energy-coupling factor transport system permease protein
CP049783.1_1007	K02435	35.93	65.5	1.3e-18	aspartyl-tRNA(Asn)/glutamyl-tRNA(Gln) amidotransferase subunit C [EC:6.3.5.6 6.3.5.7]
CP049783.1_1009	K08968	91.93	251.7	2.8e-75	L-methionine (R)-S-oxide reductase [EC:1.8.4.14]
CP049783.1_1011	K21744	131.27	220.0	1.8e-65	MerR family transcriptional regulator, thiopeptide resistance regulator
CP049783.1_1012	K21745	110.70	126.7	1.9e-37	MerR family transcriptional regulator, aldehyde-responsive regulator
CP049783.1_1014	K06284	86.83	99.7	3e-29	AbrB family transcriptional regulator, transcriptional pleiotropic regulator of transition state genes
CP049783.1_1015	K00355	115.87	206.1	3.2e-61	NAD(P)H dehydrogenase (quinone) [EC:1.6.5.2]
CP049783.1_1022	K08221	232.17	531.1	5.5e-160	MFS transporter, ACDE family, multidrug resistance protein
CP049783.1_1023	K21902	113.00	165.3	5.4e-49	MerR family transcriptional regulator, repressor of the yfmOP operon
CP049783.1_1027	K07005	53.80	118.5	1.6e-34	uncharacterized protein
CP049783.1_1028	K00375	343.30	469.5	5e-141	GntR family transcriptional regulator / MocR family aminotransferase
CP049783.1_1035	K01218	70.20	143.3	4e-42	mannan endo-1,4-beta-mannosidase [EC:3.2.1.78]
CP049783.1_1036	K10119	296.93	319.0	1.1e-95	raffinose/stachyose/melibiose transport system permease protein
CP049783.1_1037	K10118	333.37	352.2	1.1e-105	raffinose/stachyose/melibiose transport system permease protein
CP049783.1_1037	K02025	276.90	313.2	6.5e-94	multiple sugar transport system permease protein
CP049783.1_1038	K10117	246.87	277.2	1.3e-82	raffinose/stachyose/melibiose transport system substrate-binding protein
CP049783.1_1039	K07720	156.87	313.1	1.4e-93	two-component system, response regulator YesN

CP049783.1_1040	K07718	273.17	439.3	1.1e-131	two-component system, sensor histidine kinase YesM [EC:2.7.13.3]
CP049783.1_1045	K06979	169.83	297.1	8.5e-89	macrolide phosphotransferase
CP049783.1_1047	K15975	357.47	452.2	6.4e-136	glyoxalase family protein
CP049783.1_1048	K15976	141.90	334.8	4.6e-101	putative NAD(P)H nitroreductase [EC:1.-.-.-]
CP049783.1_1050	K00077	75.90	243.2	1.9e-72	2-dehydropantoate 2-reductase [EC:1.1.1.169]
CP049783.1_1051	K19784	108.27	180.6	1.7e-53	chromate reductase, NAD(P)H dehydrogenase (quinone)
CP049783.1_1052	K08978	70.23	94.6	2.4e-27	bacterial/archaeal transporter family protein
CP049783.1_1054	K13256	42.57	134.5	1e-39	protein PsiE
CP049783.1_1055	K03975	59.97	95.2	1.2e-27	membrane-associated protein
CP049783.1_1056	K17103	126.47	190.8	1.5e-56	CDP-diacylglycerol---serine O-phosphatidyltransferase [EC:2.7.8.8]
CP049783.1_1057	K18345	349.00	545.6	3.5e-164	two-component system, OmpR family, sensor histidine kinase VanS [EC:2.7.13.3]
CP049783.1_1058	K02483	242.00	258.9	3.2e-77	two-component system, OmpR family, response regulator
CP049783.1_1059	K01201	204.70	400.5	4e-120	glucosylceramidase [EC:3.2.1.45]
CP049783.1_1062	K13727	152.17	272.1	3.8e-82	phenacrylate decarboxylase [EC:4.1.1.102]
CP049783.1_1063	K26739	181.10	235.9	8.7e-71	PadR family transcriptional regulator, phenolic acid-responsive transcriptional regulator
CP049783.1_1063	K10947	65.70	66.1	1.5e-18	PadR family transcriptional regulator
CP049783.1_1064	K06312	355.23	439.2	3.2e-132	spore germination protein
CP049783.1_1065	K06311	313.40	368.5	1e-110	spore germination protein
CP049783.1_1066	K06310	662.87	695.5	1e-209	spore germination protein
CP049783.1_1066	K06295	452.20	644.1	1.2e-193	spore germination protein KA
CP049783.1_1070	K26138	104.60	127.4	1e-37	nitrite reductase [NAD(P)H] small subunit [EC:1.7.1.4]
CP049783.1_1071	K26139	720.47	986.7	2.1e-297	nitrite reductase [NAD(P)H] large subunit [EC:1.7.1.4]
CP049783.1_1072	K06310	662.87	718.0	1.6e-216	spore germination protein
CP049783.1_1072	K06295	452.20	668.6	4.3e-201	spore germination protein KA
CP049783.1_1073	K06311	313.40	398.2	9.8e-120	spore germination protein
CP049783.1_1074	K06312	355.23	465.3	4e-140	spore germination protein
CP049783.1_1075	K03486	214.00	346.7	2.1e-104	GntR family transcriptional regulator, trehalose operon transcriptional repressor
CP049783.1_1076	K02819	628.60	760.4	3.4e-229	trehalose PTS system EIIBC or EIIBCA component [EC:2.7.1.201]
CP049783.1_1076	K02810	596.80	681.3	6e-205	sucrose PTS system EIIBCA or EIIBC component [EC:2.7.1.211]
CP049783.1_1077	K01226	799.77	878.5	6.4e-265	trehalose-6-phosphate hydrolase [EC:3.2.1.93]
CP049783.1_1078	K11249	109.63	252.0	9.6e-76	cysteine/O-acetylserine efflux protein
CP049783.1_1079	K23779	92.83	226.9	9.8e-68	XRE family transcriptional regulator, regulator of sulfur utilization
CP049783.1_1080	K00661	129.97	314.9	1.3e-94	maltose O-acetyltransferase [EC:2.3.1.79]
CP049783.1_1082	K00382	465.60	570.7	9.9e-172	dihydrolipoyl dehydrogenase [EC:1.8.1.4]
CP049783.1_1083	K07397	72.77	83.9	4.6e-24	putative redox protein
CP049783.1_1089	K01190	365.30	545.7	6.4e-164	beta-galactosidase [EC:3.2.1.23]
CP049783.1_1097	K02099	174.60	205.9	3.3e-61	AraC family transcriptional regulator, arabinose operon regulatory protein
CP049783.1_1102	K13955	330.60	458.0	4.2e-138	zinc-binding alcohol dehydrogenase/oxidoreductase
CP049783.1_1102	K00344	279.00	313.0	1.2e-93	NADPH:quinone reductase [EC:1.6.5.5]
CP049783.1_1103	K21745	110.70	123.4	1.9e-36	MerR family transcriptional regulator, aldehyde-responsive regulator
CP049783.1_1104	K06221	349.23	422.0	4.1e-127	2,5-diketo-D-gluconate reductase A [EC:1.1.1.346]
CP049783.1_1105	K23107	334.20	560.8	8.5e-169	1-deoxyxylulose-5-phosphate synthase [EC:1.1.-.-]
CP049783.1_1106	K11537	625.17	649.2	8.3e-196	MFS transporter, NHS family, xanthosine permease
CP049783.1_1107	K01839	156.37	645.6	2.2e-194	phosphopentomutase [EC:5.4.2.7]
CP049783.1_1110	K03783	165.87	419.2	7.8e-126	purine-nucleoside phosphorylase [EC:2.4.2.1]
CP049783.1_1113	K05555	160.80	248.4	3.3e-74	cyclase [EC:4.-.-.-]
CP049783.1_1117	K01990	262.37	272.1	2.8e-81	ABC-2 type transport system ATP-binding protein
CP049783.1_1122	K09155	95.00	585.6	3.2e-176	uncharacterized protein
CP049783.1_1124	K01265	141.83	272.1	2.4e-81	methionyl aminopeptidase [EC:3.4.11.18]
CP049783.1_1127	K03429	296.23	372.0	2e-111	processive 1,2-diacylglycerol beta-glucosyltransferase [EC:2.4.1.315]
CP049783.1_1131	K03088	96.50	97.7	2.2e-28	RNA polymerase sigma-70 factor, ECF subfamily
CP049783.1_1133	K03282	37.57	174.3	9.7e-52	large conductance mechanosensitive channel
CP049783.1_1136	K01823	34.03	73.7	5.7e-21	isopentenyl-diphosphate Delta-isomerase [EC:5.3.3.2]
CP049783.1_1137	K23356	46.03	158.8	5.6e-47	HTH-type transcriptional regulator, sugar sensing transcriptional regulator
CP049783.1_1139	K03657	367.57	482.7	7.6e-145	ATP-dependent DNA helicase UvrD/PcrA [EC:5.6.2.4]
CP049783.1_1140	K14189	194.93	328.1	1.5e-98	uncharacterized oxidoreductase [EC:1.-.-.-]

CP049783.1_1143	K01218	70.20	325.1	3.2e-97	mannan endo-1,4-beta-mannosidase [EC:3.2.1.78]
CP049783.1_1144	K17318	176.50	287.1	1.1e-85	putative aldouronate transport system substrate-binding protein
CP049783.1_1145	K17320	270.87	352.5	5.6e-106	putative aldouronate transport system permease protein
CP049783.1_1146	K17319	220.03	430.8	1.4e-129	putative aldouronate transport system permease protein
CP049783.1_1147	K07720	156.87	386.4	8.9e-116	two-component system, response regulator YesN
CP049783.1_1148	K07718	273.17	419.7	8.8e-126	two-component system, sensor histidine kinase YesM [EC:2.7.13.3]
CP049783.1_1149	K07407	52.03	182.1	7.2e-54	alpha-galactosidase [EC:3.2.1.22]
CP049783.1_1150	K02099	174.60	191.4	8.6e-57	AraC family transcriptional regulator, arabinose operon regulatory protein
CP049783.1_1151	K02073	57.57	261.4	5.6e-78	D-methionine transport system substrate-binding protein
CP049783.1_1153	K07173	101.70	246.4	1.1e-73	S-ribosylhomocysteine lyase [EC:4.4.1.21]
CP049783.1_1154	K07816	97.83	272.6	1.8e-81	GTP pyrophosphokinase [EC:2.7.6.5]
CP049783.1_1155	K01868	151.50	962.3	9.5e-290	threonyl-tRNA synthetase [EC:6.1.1.3]
CP049783.1_1162	K08989	42.47	117.4	1.4e-34	putative membrane protein
CP049783.1_1165	K07260	64.77	278.0	4.8e-83	zinc D-Ala-D-Ala carboxypeptidase [EC:3.4.17.14]
CP049783.1_1171	K25086	282.43	471.3	1.1e-141	maltose transport system substrate-binding protein
CP049783.1_1171	K10117	246.87	303.0	2e-90	raffinose/stachyose/melibiose transport system substrate-binding protein
CP049783.1_1171	K02027	193.17	202.1	6.4e-60	multiple sugar transport system substrate-binding protein
CP049783.1_1172	K25088	335.73	377.9	9.3e-114	maltose transport system permease protein
CP049783.1_1172	K10119	296.93	322.5	9.6e-97	raffinose/stachyose/melibiose transport system permease protein
CP049783.1_1173	K25087	353.50	425.5	3e-128	maltose transport system permease protein
CP049783.1_1174	K07718	273.17	465.4	1.4e-139	two-component system, sensor histidine kinase YesM [EC:2.7.13.3]
CP049783.1_1175	K07720	156.87	339.3	1.6e-101	two-component system, response regulator YesN
CP049783.1_1176	K03790	144.10	234.4	7.7e-70	[ribosomal protein S5]-alanine N-acetyltransferase [EC:2.3.1.267]
CP049783.1_1178	K03453	223.63	365.2	2.2e-109	bile acid:Na+ symporter, BASS family
CP049783.1_1180	K02563	193.67	265.0	3.8e-79	UDP-N-acetylglucosamine--N-acetylmuramyl-(pentapeptide) pyrophosphoryl-undecaprenol N-acetylglucosamine transferase [EC:2.4.1.227]
CP049783.1_1181	K09022	107.97	207.5	9.1e-62	2-iminobutanoate/2-iminopropanoate deaminase [EC:3.5.99.10]
CP049783.1_1182	K03088	96.50	134.7	1.3e-39	RNA polymerase sigma-70 factor, ECF subfamily
CP049783.1_1184	K17290	100.67	201.7	5.8e-60	oxidoreductase [EC:1.1.1.-]
CP049783.1_1187	K18979	73.50	87.3	3.4e-25	epoxyqueuosine reductase [EC:1.17.99.6]
CP049783.1_1192	K04750	56.87	76.9	6.1e-22	PhnB protein
CP049783.1_1194	K01740	412.90	818.8	9.8e-247	O-acetylhomoserine (thiol)-lyase [EC:2.5.1.49]
CP049783.1_1198	K01061	89.07	93.9	4.2e-27	carboxymethylenebutenolidase [EC:3.1.1.45]
CP049783.1_1199	K03790	144.10	232.0	4e-69	[ribosomal protein S5]-alanine N-acetyltransferase [EC:2.3.1.267]
CP049783.1_1205	K10711	243.70	322.1	6e-97	GntR family transcriptional regulator, frlABCD operon transcriptional regulator
CP049783.1_1205	K03710	178.10	211.2	9.8e-63	GntR family transcriptional regulator
CP049783.1_1206	K10710	176.40	319.2	6.2e-96	fructoselysine 6-kinase [EC:2.7.1.218]
CP049783.1_1207	K19510	373.60	508.2	3e-153	fructoselysine-6-phosphate deglycase
CP049783.1_1208	K19540	379.77	542.0	2.6e-163	fructoselysine transporter
CP049783.1_1209	K10709	198.50	347.2	1.3e-104	fructoselysine 3-epimerase [EC:5.1.3.41]
CP049783.1_1218	K19575	133.80	330.3	3.8e-99	MerR family transcriptional regulator, activator of bmr gene
CP049783.1_1229	K07486	31.47	232.7	2.7e-69	transposase
CP049783.1_1237	K00525	367.50	569.6	3.4e-171	ribonucleoside-diphosphate reductase alpha chain [EC:1.17.4.1]
CP049783.1_1239	K03406	65.50	191.8	7.4e-57	methyl-accepting chemotaxis protein
CP049783.1_1240	K05306	195.77	234.0	5.3e-70	phosphonoacetaldehyde hydrolase [EC:3.11.1.1]
CP049783.1_1242	K23997	307.27	563.6	2e-169	ADP-dependent NAD(P)H-hydrate dehydratase / NAD(P)H-hydrate epimerase [EC:4.2.1.136 5.1.99.6]
CP049783.1_1242	K17758	277.03	309.7	8.7e-93	ADP-dependent NAD(P)H-hydrate dehydratase [EC:4.2.1.136]
CP049783.1_1243	K07118	124.57	270.3	6.1e-81	uncharacterized protein
CP049783.1_1247	K07404	130.17	427.5	2.4e-128	6-phosphogluconolactonase [EC:3.1.1.31]
CP049783.1_1248	K08153	415.17	515.8	1.8e-155	MFS transporter, DHA1 family, multidrug resistance protein
CP049783.1_1252	K14205	388.70	392.7	9.6e-118	phosphatidylglycerol lysyltransferase [EC:2.3.2.3]
CP049783.1_1253	K15976	141.90	149.9	1.9e-44	putative NAD(P)H nitroreductase [EC:1.-.-.-]
CP049783.1_1262	K01005	193.97	304.9	3.7e-91	polyisoprenyl-teichoic acid--peptidoglycan teichoic acid transferase [EC:2.7.8.-]
CP049783.1_1264	K07148	83.90	107.6	1.6e-31	uncharacterized protein
CP049783.1_1268	K01265	141.83	326.8	5.8e-98	methionyl aminopeptidase [EC:3.4.11.18]
CP049783.1_1269	K11476	166.83	299.5	4.3e-90	GntR family transcriptional regulator, gluconate operon transcriptional repressor
CP049783.1_1270	K25031	463.90	790.4	2.3e-238	gluconokinase [EC:2.7.1.12]

CP049783.1_1274	K00016	308.33	340.5	6.4e-102	L-lactate dehydrogenase [EC:1.1.1.27]
CP049783.1_1275	K20461	155.27	225.2	2.7e-67	lantibiotic transport system permease protein
CP049783.1_1276	K20460	146.50	199.1	2.7e-59	lantibiotic transport system permease protein
CP049783.1_1276	K20461	155.27	160.8	1.2e-47	lantibiotic transport system permease protein
CP049783.1_1277	K20459	363.07	426.4	2.2e-128	lantibiotic transport system ATP-binding protein
CP049783.1_1277	K01990	262.37	316.5	9.8e-95	ABC-2 type transport system ATP-binding protein
CP049783.1_1280	K01208	509.17	684.9	3.4e-206	cyclomaltodextrinase / maltogenic alpha-amylase / neopullulanase [EC:3.2.1.54 3.2.1.133 3.2.1.135]
CP049783.1_1280	K01176	222.80	225.0	5.8e-67	alpha-amylase [EC:3.2.1.1]
CP049783.1_1281	K02477	121.47	177.0	1.8e-52	two-component system, LytTR family, response regulator
CP049783.1_1282	K19694	625.00	1119.0	0	two-component system, sensor histidine kinase ChiS
CP049783.1_1284	K02072	119.67	323.5	3.5e-97	D-methionine transport system permease protein
CP049783.1_1285	K02071	332.87	491.7	7.4e-148	D-methionine transport system ATP-binding protein
CP049783.1_1286	K01567	253.73	390.5	1.5e-117	peptidoglycan-N-acetylmuramic acid deacetylase [EC:3.5.1.-]
CP049783.1_1286	K22278	73.27	212.8	2.9e-63	peptidoglycan-N-acetylglucosamine deacetylase [EC:3.5.1.104]
CP049783.1_1288	K01182	875.10	913.3	4.9e-275	oligo-1,6-glucosidase [EC:3.2.1.10]
CP049783.1_1291	K01119	358.37	889.2	6.6e-268	2',3'-cyclic-nucleotide 2'-phosphodiesterase / 3'-nucleotidase [EC:3.1.4.16 3.1.3.6]
CP049783.1_1293	K24987	519.03	533.5	5e-161	putative thiamine transport system substrate-binding protein
CP049783.1_1293	K02051	157.20	189.5	4.4e-56	NitT/TauT family transport system substrate-binding protein
CP049783.1_1295	K24988	354.90	356.9	1.8e-107	putative thiamine transport system permease protein
CP049783.1_1295	K02050	145.27	227.2	1.3e-67	NitT/TauT family transport system permease protein
CP049783.1_1296	K02049	352.60	357.9	3.9e-107	NitT/TauT family transport system ATP-binding protein
CP049783.1_1297	K03424	86.70	163.8	2.2e-48	TatD DNase family protein [EC:3.1.21.-]
CP049783.1_1298	K19285	297.90	325.3	1.6e-97	FMN reductase (NADPH) [EC:1.5.1.38]
CP049783.1_1299	K17686	799.73	1141.8	0	P-type Cu+ transporter [EC:7.2.2.8]
CP049783.1_1299	K01533	923.57	977.9	2.8e-294	P-type Cu2+ transporter [EC:7.2.2.9]
CP049783.1_1300	K07213	66.63	69.0	1.9e-19	copper chaperone
CP049783.1_1301	K21600	85.87	133.8	2.4e-39	CsoR family transcriptional regulator, copper-sensing transcriptional repressor
CP049783.1_1302	K07552	308.70	506.1	3.4e-152	MFS transporter, DHA1 family, multidrug resistance protein
CP049783.1_1307	K14166	176.60	394.3	3.4e-118	copper transport protein
CP049783.1_1307	K07156	71.17	127.1	3.1e-37	copper resistance protein C
CP049783.1_1316	K07481	59.83	75.0	2.6e-21	transposase, IS5 family
CP049783.1_1318	K07284	60.53	166.9	2.1e-49	sortase A [EC:3.4.22.70]
CP049783.1_1319	K09705	33.63	185.9	5e-55	uncharacterized protein
CP049783.1_1320	K02028	386.70	450.0	3.4e-135	polar amino acid transport system ATP-binding protein [EC:7.4.2.1]
CP049783.1_1320	K10041	409.80	440.1	4.6e-133	aspartate/glutamate/glutamine transport system ATP-binding protein [EC:7.4.2.1]
CP049783.1_1321	K10039	279.17	388.2	5.5e-117	aspartate/glutamate/glutamine transport system substrate-binding protein
CP049783.1_1321	K02030	76.77	151.5	1.1e-44	polar amino acid transport system substrate-binding protein
CP049783.1_1322	K10040	255.97	298.6	1.4e-89	aspartate/glutamate/glutamine transport system permease protein
CP049783.1_1322	K02029	214.53	262.0	3.2e-78	polar amino acid transport system permease protein
CP049783.1_1323	K10040	255.97	279.4	9.4e-84	aspartate/glutamate/glutamine transport system permease protein
CP049783.1_1323	K02029	214.53	245.5	3.1e-73	polar amino acid transport system permease protein
CP049783.1_1324	K11921	265.87	279.1	2.2e-83	LysR family transcriptional regulator, cyn operon transcriptional activator
CP049783.1_1325	K02035	249.67	298.8	2.7e-89	peptide/nickel transport system substrate-binding protein
CP049783.1_1326	K02034	256.80	276.5	1.2e-82	peptide/nickel transport system permease protein
CP049783.1_1327	K02033	263.63	344.8	2.6e-103	peptide/nickel transport system permease protein
CP049783.1_1328	K02032	420.67	451.4	2.1e-135	peptide/nickel transport system ATP-binding protein
CP049783.1_1329	K02031	412.13	464.1	2.4e-139	peptide/nickel transport system ATP-binding protein
CP049783.1_1331	K01752	73.17	152.8	4.6e-45	L-serine dehydratase [EC:4.3.1.17]
CP049783.1_1332	K01752	73.17	330.7	6.9e-99	L-serine dehydratase [EC:4.3.1.17]
CP049783.1_1336	K21759	32.37	103.5	1.3e-30	vanillate/4-hydroxybenzoate decarboxylase subunit D [EC:4.1.1.- 4.1.1.61]
CP049783.1_1337	K01612	577.73	824.9	4.3e-249	vanillate/4-hydroxybenzoate decarboxylase subunit C [EC:4.1.1.- 4.1.1.61]
CP049783.1_1338	K03186	92.57	324.4	3.1e-97	flavin prenyltransferase [EC:2.5.1.129]
CP049783.1_1339	K21755	243.37	377.6	1.2e-113	LysR family transcriptional regulator, salicylic acid-responsive activator of bsdBCD
CP049783.1_1340	K07720	156.87	302.0	3.4e-90	two-component system, response regulator YesN
CP049783.1_1341	K07718	273.17	507.0	3.6e-152	two-component system, sensor histidine kinase YesM [EC:2.7.13.3]
CP049783.1_1342	K10119	296.93	341.4	1.7e-102	raffinose/stachyose/melibiose transport system permease protein

CP049783.1_1343	K10118	333.37	354.6	2e-106	raffinose/stachyose/melibiose transport system permease protein
CP049783.1_1344	K10117	246.87	345.9	1.9e-103	raffinose/stachyose/melibiose transport system substrate-binding protein
CP049783.1_1344	K02027	193.17	222.4	4.4e-66	multiple sugar transport system substrate-binding protein
CP049783.1_1348	K25026	274.23	303.0	1.1e-90	glucokinase [EC:2.7.1.2]
CP049783.1_1350	K01788	97.90	348.9	6.9e-105	N-acylglucosamine-6-phosphate 2-epimerase [EC:5.1.3.9]
CP049783.1_1353	K17246	330.13	352.6	4.7e-106	putative chitobiose transport system permease protein
CP049783.1_1353	K02026	280.30	289.1	1.5e-86	multiple sugar transport system permease protein
CP049783.1_1354	K17245	346.60	380.8	1.5e-114	putative chitobiose transport system permease protein
CP049783.1_1354	K02025	276.90	340.9	2.6e-102	multiple sugar transport system permease protein
CP049783.1_1355	K17244	293.00	467.1	1.7e-140	putative chitobiose transport system substrate-binding protein
CP049783.1_1355	K02027	193.17	219.6	3.2e-65	multiple sugar transport system substrate-binding protein
CP049783.1_1356	K08976	36.27	224.2	5.5e-67	putative membrane protein
CP049783.1_1357	K06975	44.10	78.9	1.6e-22	uncharacterized protein
CP049783.1_1358	K01971	209.27	249.9	2e-74	bifunctional non-homologous end joining protein LigD [EC:6.5.1.1]
CP049783.1_1361	K15986	162.20	432.1	3.5e-130	manganese-dependent inorganic pyrophosphatase [EC:3.6.1.1]
CP049783.1_1362	K14647	588.50	797.5	6e-240	minor extracellular serine protease Vpr [EC:3.4.21.-]
CP049783.1_1363	K02484	312.80	349.3	1.5e-104	two-component system, OmpR family, sensor kinase [EC:2.7.13.3]
CP049783.1_1364	K02483	242.00	261.2	6.3e-78	two-component system, OmpR family, response regulator
CP049783.1_1366	K20534	338.93	498.9	4.3e-150	polyisoprenyl-phosphate glycosyltransferase [EC:2.4.-.-]
CP049783.1_1368	K00963	76.77	142.3	7.3e-42	UTP--glucose-1-phosphate uridylyltransferase [EC:2.7.7.9]
CP049783.1_1369	K22438	107.73	115.5	1.1e-33	trans-aconitate 3-methyltransferase [EC:2.1.1.145]
CP049783.1_1370	K01126	152.53	249.4	1.4e-74	glycerophosphoryl diester phosphodiesterase [EC:3.1.4.46]
CP049783.1_1372	K04079	91.80	280.4	9.9e-84	molecular chaperone HtpG
CP049783.1_1373	K01209	85.37	467.7	2.4e-140	alpha-L-arabinofuranosidase [EC:3.2.1.55]
CP049783.1_1378	K06871	174.27	254.6	6.9e-76	uncharacterized protein
CP049783.1_1383	K02026	280.30	349.6	5.9e-105	multiple sugar transport system permease protein
CP049783.1_1384	K02025	276.90	348.2	1.6e-104	multiple sugar transport system permease protein
CP049783.1_1385	K02027	193.17	247.0	1.6e-73	multiple sugar transport system substrate-binding protein
CP049783.1_1386	K05349	305.00	716.0	2.5e-215	beta-glucosidase [EC:3.2.1.21]
CP049783.1_1387	K21298	765.33	1608.9	0	1,2-beta-oligoglucan phosphorylase [EC:2.4.1.333]
CP049783.1_1388	K02529	268.37	293.0	1.5e-87	LacI family transcriptional regulator, galactose operon repressor
CP049783.1_1394	K07979	106.93	108.1	1.2e-31	GntR family transcriptional regulator
CP049783.1_1398	K00433	197.80	239.4	2.3e-71	non-heme chloroperoxidase [EC:1.11.1.10]
CP049783.1_1400	K03704	80.73	121.0	1.6e-35	cold shock protein
CP049783.1_1401	K06113	228.80	416.8	4.3e-125	arabinan endo-1,5-alpha-L-arabinosidase [EC:3.2.1.99]
CP049783.1_1403	K06295	452.20	771.2	4.3e-232	spore germination protein KA
CP049783.1_1404	K06297	281.23	401.3	2e-120	spore germination protein KC
CP049783.1_1406	K06296	248.90	385.1	1.3e-115	spore germination protein KB
CP049783.1_1408	K00873	49.90	645.5	2.9e-194	pyruvate kinase [EC:2.7.1.40]
CP049783.1_1410	K02810	596.80	670.0	1.6e-201	sucrose PTS system EIIBCA or EIIBC component [EC:2.7.1.211]
CP049783.1_1411	K01193	187.77	477.9	1.5e-143	beta-fructofuranosidase [EC:3.2.1.26]
CP049783.1_1412	K03484	339.57	386.1	5.3e-116	LacI family transcriptional regulator, sucrose operon repressor
CP049783.1_1413	K23257	397.27	463.6	5.4e-140	methylglyoxal/glyoxal reductase [EC:1.1.1.283 1.1.1.-]
CP049783.1_1413	K06221	349.23	413.2	1.9e-124	2,5-diketo-D-gluconate reductase A [EC:1.1.1.346]
CP049783.1_1416	K13953	338.93	453.8	1.7e-136	alcohol dehydrogenase, propanol-preferring [EC:1.1.1.1]
CP049783.1_1417	K01674	238.53	310.9	4.2e-93	carbonic anhydrase [EC:4.2.1.1]
CP049783.1_1420	K18954	149.17	168.5	9e-50	AraC family transcriptional regulator, transcriptional activator of pobA
CP049783.1_1420	K07506	141.00	142.2	7.6e-42	AraC family transcriptional regulator
CP049783.1_1421	K02026	280.30	361.2	1.8e-108	multiple sugar transport system permease protein
CP049783.1_1421	K10119	296.93	298.0	2.7e-89	raffinose/stachyose/melibiose transport system permease protein
CP049783.1_1422	K02025	276.90	386.1	4.8e-116	multiple sugar transport system permease protein
CP049783.1_1422	K05814	358.43	373.5	3.2e-112	sn-glycerol 3-phosphate transport system permease protein
CP049783.1_1422	K10118	333.37	339.8	6.2e-102	raffinose/stachyose/melibiose transport system permease protein
CP049783.1_1423	K02027	193.17	225.2	6.5e-67	multiple sugar transport system substrate-binding protein
CP049783.1_1427	K02032	420.67	466.3	6.8e-140	peptide/nickel transport system ATP-binding protein
CP049783.1_1429	K02034	256.80	355.6	1.3e-106	peptide/nickel transport system permease protein

CP049783.1_1430	K02033	263.63	383.7	4.2e-115	peptide/nickel transport system permease protein
CP049783.1_1431	K02035	249.67	382.6	1.2e-114	peptide/nickel transport system substrate-binding protein
CP049783.1_1432	K08987	33.40	168.0	8e-50	putative membrane protein
CP049783.1_1433	K03406	65.50	130.1	3.5e-38	methyl-accepting chemotaxis protein
CP049783.1_1434	K11102	518.13	655.2	8.4e-198	proton glutamate symport protein
CP049783.1_1435	K07717	229.97	497.2	1e-149	two-component system, sensor histidine kinase GlnK [EC:2.7.13.3]
CP049783.1_1436	K07719	195.10	384.0	1.3e-115	two-component system, response regulator GlnL
CP049783.1_1441	K06896	115.07	322.6	6.6e-97	maltose 6'-phosphate phosphatase [EC:3.1.3.90]
CP049783.1_1442	K20108	530.20	957.2	9e-289	maltose PTS system EIICB or EIICBA component [EC:2.7.1.208]
CP049783.1_1443	K02529	268.37	292.3	2.5e-87	LacI family transcriptional regulator, galactose operon repressor
CP049783.1_1444	K00691	826.17	1196.4	0	maltose phosphorylase [EC:2.4.1.8]
CP049783.1_1445	K01838	220.33	312.0	1.5e-93	beta-phosphoglucomutase [EC:5.4.2.6]
CP049783.1_1446	K06295	452.20	667.6	8.6e-201	spore germination protein KA
CP049783.1_1446	K06310	662.87	663.5	5.1e-200	spore germination protein
CP049783.1_1447	K06312	355.23	360.7	2.4e-108	spore germination protein
CP049783.1_1448	K06311	313.40	455.0	5.3e-137	spore germination protein
CP049783.1_1448	K06296	248.90	250.8	7.1e-75	spore germination protein KB
CP049783.1_1452	K01728	168.73	188.3	7.8e-56	pectate lyase [EC:4.2.2.2]
CP049783.1_1454	K00876	144.07	269.1	2.1e-80	uridine kinase [EC:2.7.1.48]
CP049783.1_1455	K07720	156.87	358.7	2.2e-107	two-component system, response regulator YesN
CP049783.1_1456	K07718	273.17	427.7	3.5e-128	two-component system, sensor histidine kinase YesM [EC:2.7.13.3]
CP049783.1_1457	K10188	333.70	528.3	4.4e-159	lactose/L-arabinose transport system substrate-binding protein
CP049783.1_1458	K10189	353.00	439.8	1.4e-132	lactose/L-arabinose transport system permease protein
CP049783.1_1458	K02025	276.90	344.3	2.3e-103	multiple sugar transport system permease protein
CP049783.1_1459	K10190	328.17	358.0	9.8e-108	lactose/L-arabinose transport system permease protein
CP049783.1_1460	K01190	365.30	619.3	3.7e-186	beta-galactosidase [EC:3.2.1.23]
CP049783.1_1464	K09955	70.47	717.8	5.8e-216	uncharacterized protein
CP049783.1_1466	K21959	245.10	393.6	1.4e-118	LysR family transcriptional regulator, cell division regulator
CP049783.1_1467	K13955	330.60	463.9	6.9e-140	zinc-binding alcohol dehydrogenase/oxidoreductase
CP049783.1_1469	K03292	344.60	401.4	1.5e-120	glycoside/pentoside/hexuronide:cation symporter, GPH family
CP049783.1_1470	K09955	70.47	523.6	2.8e-157	uncharacterized protein
CP049783.1_1472	K01083	115.73	479.4	5.3e-144	3-phytase [EC:3.1.3.8]
CP049783.1_1473	K01209	85.37	288.3	4.9e-86	alpha-L-arabinofuranosidase [EC:3.2.1.55]
CP049783.1_1475	K06872	48.37	77.1	3.9e-22	uncharacterized protein
CP049783.1_1477	K03969	84.50	178.5	7.9e-53	phage shock protein A
CP049783.1_1478	K06221	349.23	360.7	1.8e-108	2,5-diketo-D-gluconate reductase A [EC:1.1.1.346]
CP049783.1_1479	K01621	304.80	1340.5	0	xylulose-5-phosphate/fructose-6-phosphate phosphoketolase [EC:4.1.2.9 4.1.2.22]
CP049783.1_1480	K03492	234.97	277.2	2.9e-83	GntR family transcriptional regulator, regulator of glucomannan utilization
CP049783.1_1482	K15975	357.47	463.3	2.6e-139	glyoxalase family protein
CP049783.1_1483	K15977	60.27	121.4	2e-35	putative oxidoreductase
CP049783.1_1495	K03664	42.43	240.8	3.5e-72	SsrA-binding protein
CP049783.1_1496	K12573	484.07	1086.1	0	ribonuclease R [EC:3.1.13.1]
CP049783.1_1497	K03075	26.13	68.7	2.1e-19	preprotein translocase subunit SecG
CP049783.1_1498	K01689	269.20	780.5	6.6e-235	enolase 1/2/3 [EC:4.2.1.11]
CP049783.1_1499	K15633	73.30	804.4	1.8e-242	2,3-bisphosphoglycerate-independent phosphoglycerate mutase [EC:5.4.2.12]
CP049783.1_1500	K01803	24.57	331.5	1.9e-99	triosephosphate isomerase (TIM) [EC:5.3.1.1]
CP049783.1_1501	K00927	104.93	611.7	3.3e-184	phosphoglycerate kinase [EC:2.7.2.3]
CP049783.1_1502	K00134	414.10	485.7	5e-146	glyceraldehyde 3-phosphate dehydrogenase (phosphorylating) [EC:1.2.1.12]
CP049783.1_1503	K05311	215.10	457.2	7.4e-138	central glycolytic genes regulator
CP049783.1_1504	K01358	76.73	373.7	2.4e-112	ATP-dependent Clp protease, protease subunit [EC:3.4.21.92]
CP049783.1_1506	K09807	88.93	204.9	6.2e-61	uncharacterized protein
CP049783.1_1507	K11184	111.63	129.6	8.2e-39	catabolite repression HPr-like protein
CP049783.1_1507	K02784	81.30	104.0	2.9e-30	phosphocarrier protein HPr
CP049783.1_1508	K09762	93.53	416.7	2.5e-125	cell division protein WhiA
CP049783.1_1510	K06958	140.17	413.9	1.8e-124	RNase adapter protein RapZ
CP049783.1_1511	K25026	274.23	349.6	8.3e-105	glucokinase [EC:2.7.1.2]

CP049783.1_1512	K00384	332.10	496.1	3e-149	thioredoxin reductase (NADPH) [EC:1.8.1.9]
CP049783.1_1514	K04486	126.10	285.0	3e-85	histidinol-phosphatase (PHP family) [EC:3.1.3.15]
CP049783.1_1515	K11755	228.33	323.8	5.4e-97	phosphoribosyl-AMP cyclohydrolase / phosphoribosyl-ATP pyrophosphohydrolase [EC:3.5.4.19 3.6.1.31]
CP049783.1_1516	K02500	339.30	468.5	9.3e-141	imidazole glycerol-phosphate synthase subunit HisF [EC:4.3.2.10]
CP049783.1_1517	K01814	249.67	313.3	1e-93	phosphoribosylformimino-5-aminoimidazole carboxamide ribotide isomerase [EC:5.3.1.16]
CP049783.1_1518	K02501	231.60	298.6	1.1e-89	imidazole glycerol-phosphate synthase subunit HisH [EC:4.3.2.10]
CP049783.1_1519	K01693	300.33	322.3	6.9e-97	imidazoleglycerol-phosphate dehydratase [EC:4.2.1.19]
CP049783.1_1520	K00013	519.37	638.4	4.7e-192	histidinol dehydrogenase [EC:1.1.1.23]
CP049783.1_1521	K00765	43.93	233.2	1.5e-69	ATP phosphoribosyltransferase [EC:2.4.2.17]
CP049783.1_1522	K02502	242.30	395.8	8e-119	ATP phosphoribosyltransferase regulatory subunit
CP049783.1_1524	K24872	135.27	290.7	7.6e-88	heptaprenylglycerol acetyltransferase [EC:2.3.1.-]
CP049783.1_1525	K06019	203.97	277.4	3.1e-83	pyrophosphatase PpaX [EC:3.6.1.1]
CP049783.1_1525	K01091	112.83	157.4	2e-46	phosphoglycolate phosphatase [EC:3.1.3.18]
CP049783.1_1526	K13292	50.50	299.9	7.2e-90	phosphatidylglycerol---prolipoprotein diacylglyceryl transferase [EC:2.5.1.145]
CP049783.1_1527	K06023	97.50	459.0	6e-138	HPr kinase/phosphorylase [EC:2.7.11.- 2.7.4.-]
CP049783.1_1528	K10112	546.20	588.3	5.5e-177	multiple sugar transport system ATP-binding protein [EC:7.5.2.-]
CP049783.1_1531	K06317	31.03	60.8	4.5e-17	inhibitor of the pro-sigma K processing machinery
CP049783.1_1533	K06187	59.67	358.8	1.1e-107	recombination protein RecR
CP049783.1_1534	K09747	46.57	155.2	5.1e-46	nucleoid-associated protein EbfC
CP049783.1_1535	K02343	233.40	564.4	8.7e-170	DNA polymerase III subunit gamma/tau [EC:2.7.7.7]
CP049783.1_1535	K02341	230.10	242.8	3e-72	DNA polymerase III subunit delta' [EC:2.7.7.7]
CP049783.1_1536	K02909	21.83	111.5	1.2e-32	large subunit ribosomal protein L31
CP049783.1_1538	K03628	186.43	717.2	3.4e-216	transcription termination factor Rho
CP049783.1_1539	K00790	172.73	584.1	6.4e-176	UDP-N-acetylglucosamine 1-carboxyvinyltransferase [EC:2.5.1.7]
CP049783.1_1540	K01624	67.57	373.2	1e-111	fructose-bisphosphate aldolase, class II [EC:4.1.2.13]
CP049783.1_1541	K02490	154.10	192.8	1.3e-57	two-component system, response regulator, stage 0 sporulation protein F
CP049783.1_1542	K01937	133.63	898.9	1.1e-270	CTP synthase [EC:6.3.4.2]
CP049783.1_1543	K03048	43.67	147.9	9.4e-44	DNA-directed RNA polymerase subunit delta
CP049783.1_1546	K02011	336.03	461.5	1.2e-138	iron(III) transport system permease protein
CP049783.1_1547	K02012	205.27	289.1	2.6e-86	iron(III) transport system substrate-binding protein
CP049783.1_1551	K01887	107.30	465.1	1.5e-139	arginyl-tRNA synthetase [EC:6.1.1.19]
CP049783.1_1553	K21755	243.37	279.1	1.2e-83	LysR family transcriptional regulator, salicylic acid-responsive activator of bsdBCD
CP049783.1_1554	K18887	708.60	817.9	2.8e-246	ATP-binding cassette, subfamily B, multidrug efflux pump
CP049783.1_1555	K18888	717.83	850.0	5.6e-256	ATP-binding cassette, subfamily B, multidrug efflux pump
CP049783.1_1555	K06147	612.93	629.7	2.4e-189	ATP-binding cassette, subfamily B, bacterial
CP049783.1_1557	K00797	93.83	282.1	2.5e-84	spermidine synthase [EC:2.5.1.16]
CP049783.1_1558	K21464	776.57	906.3	4.1e-273	penicillin-binding protein 2D [EC:2.4.99.28 3.4.16.4]
CP049783.1_1558	K05366	639.07	729.2	2.4e-219	penicillin-binding protein 1A [EC:2.4.99.28 3.4.16.4]
CP049783.1_1561	K04750	56.87	86.8	5.6e-25	PhnB protein
CP049783.1_1571	K01724	20.90	86.9	5.1e-25	4a-hydroxytetrahydrobiopterin dehydratase [EC:4.2.1.96]
CP049783.1_1573	K02556	81.20	283.7	7.3e-85	chemotaxis protein MotA
CP049783.1_1574	K02557	144.00	221.0	7e-66	chemotaxis protein MotB
CP049783.1_1575	K06182	241.13	339.8	5.9e-102	23S rRNA pseudouridine2604 synthase [EC:5.4.99.21]
CP049783.1_1576	K02491	453.23	480.6	2.3e-144	two-component system, sporulation sensor kinase A [EC:2.7.13.3]
CP049783.1_1576	K13533	408.10	443.3	3.9e-133	two-component system, sporulation sensor kinase E [EC:2.7.13.3]
CP049783.1_1577	K11991	164.80	275.5	3.9e-82	tRNA(adenine34) deaminase [EC:3.5.4.33]
CP049783.1_1578	K03790	144.10	239.4	2.2e-71	[ribosomal protein S5]-alanine N-acetyltransferase [EC:2.3.1.267]
CP049783.1_1580	K06433	38.23	53.3	8.8e-15	small acid-soluble spore protein P (minor)
CP049783.1_1583	K01875	89.73	608.4	6.5e-183	seryl-tRNA synthetase [EC:6.1.1.11]
CP049783.1_1584	K08681	77.37	300.3	3.3e-90	pyridoxal 5'-phosphate synthase pdxT subunit [EC:4.3.3.6]
CP049783.1_1585	K06215	123.87	531.7	2.4e-160	pyridoxal 5'-phosphate synthase pdxS subunit [EC:4.3.3.6]
CP049783.1_1586	K07258	223.10	359.7	8.5e-108	serine-type D-Ala-D-Ala carboxypeptidase (penicillin-binding protein 5/6) [EC:3.4.16.4]
CP049783.1_1587	K00088	375.03	749.4	1.6e-225	IMP dehydrogenase [EC:1.1.1.205]
CP049783.1_1591	K16118	1359.93	1843.7	0	pristinamycin I synthase 3 and 4
CP049783.1_1592	K02372	79.93	115.6	1.3e-33	3-hydroxyacyl-[acyl-carrier-protein] dehydratase [EC:4.2.1.59]
CP049783.1_1593	K09458	480.27	566.4	2.4e-170	3-oxoacyl-[acyl-carrier-protein] synthase II [EC:2.3.1.179]

CP049783.1_1597	K00208	269.47	387.2	3e-116	enoyl-[acyl-carrier protein] reductase I [EC:1.3.1.9 1.3.1.10]
CP049783.1_1599	K03498	117.37	463.2	3.6e-139	trk/ktr system potassium uptake protein
CP049783.1_1600	K04567	319.33	779.1	1.6e-234	lysyl-tRNA synthetase, class II [EC:6.1.1.6]
CP049783.1_1602	K05540	281.20	463.5	1.8e-139	tRNA-dihydrouridine synthase B [EC:1.-.-.-]
CP049783.1_1604	K00950	38.63	198.5	3.9e-59	2-amino-4-hydroxy-6-hydroxymethyldihydropteridine diphosphokinase [EC:2.7.6.3]
CP049783.1_1605	K01633	94.10	142.9	4.1e-42	7,8-dihydroneopterin aldolase/epimerase/oxygenase [EC:4.1.2.25 5.1.99.8 1.13.11.81]
CP049783.1_1606	K00796	237.57	396.0	5.6e-119	dihydropteroate synthase [EC:2.5.1.15]
CP049783.1_1607	K02619	196.63	232.1	4.1e-69	4-amino-4-deoxychorismate lyase [EC:4.1.3.38]
CP049783.1_1608	K01664	320.30	345.7	6.9e-104	para-aminobenzoate synthetase component II [EC:2.6.1.85]
CP049783.1_1608	K01658	241.93	344.0	1.3e-103	anthranilate synthase component II [EC:4.1.3.27]
CP049783.1_1608	K25575	332.40	338.1	3.5e-102	4-amino-4-deoxychorismate synthase (2-amino-4-deoxychorismate-forming) component II [EC:2.6.1.123]
CP049783.1_1609	K25578	527.73	650.2	6e-196	4-amino-4-deoxychorismate synthase (2-amino-4-deoxychorismate-forming) component I [EC:2.6.1.123]
CP049783.1_1609	K01657	465.93	550.0	2.4e-165	anthranilate synthase component I [EC:4.1.3.27]
CP049783.1_1610	K01738	426.93	501.0	1.1e-150	cysteine synthase [EC:2.5.1.47]
CP049783.1_1612	K04083	58.73	353.6	3.3e-106	molecular chaperone Hsp33
CP049783.1_1613	K03525	120.67	287.0	8.2e-86	type III pantothenate kinase [EC:2.7.1.33]
CP049783.1_1614	K00767	217.50	421.6	1.1e-126	nicotinate-nucleotide pyrophosphorylase (carboxylating) [EC:2.4.2.19]
CP049783.1_1615	K00278	526.70	753.0	9.7e-227	L-aspartate oxidase [EC:1.4.3.16]
CP049783.1_1616	K03798	767.57	941.2	1.9e-283	cell division protease FtsH [EC:3.4.24.-]
CP049783.1_1617	K00760	82.30	245.1	3.3e-73	hypoxanthine phosphoribosyltransferase [EC:2.4.2.8]
CP049783.1_1618	K04075	118.80	319.5	1.2e-95	tRNA(Ile)-lysidine synthase [EC:6.3.4.19]
CP049783.1_1621	K06382	265.27	1003.9	1.7e-302	stage II sporulation protein E [EC:3.1.3.16]
CP049783.1_1622	K07571	145.30	204.9	1.5e-61	S1 RNA binding domain protein
CP049783.1_1623	K13052	58.13	77.3	3.5e-22	cell division protein DivIC
CP049783.1_1626	K04762	59.53	67.8	3.5e-19	ribosome-associated heat shock protein Hsp15
CP049783.1_1627	K03530	115.33	141.4	1.4e-41	DNA-binding protein HU-beta
CP049783.1_1628	K02499	400.47	520.9	9.6e-157	tetrapyrrole methylase family protein / MazG family protein
CP049783.1_1628	K04765	371.10	377.9	1.7e-113	nucleoside triphosphate diphosphatase [EC:3.6.1.9]
CP049783.1_1630	K06409	388.70	428.3	1.3e-128	stage V sporulation protein B
CP049783.1_1631	K04769	147.60	287.0	2.2e-86	AbrB family transcriptional regulator, stage V sporulation protein T
CP049783.1_1634	K03723	555.70	1365.6	0	transcription-repair coupling factor (superfamily II helicase) [EC:5.6.2.4]
CP049783.1_1636	K01056	49.10	261.3	4.2e-78	peptidyl-tRNA hydrolase, PTH1 family [EC:3.1.1.29]
CP049783.1_1638	K00948	62.27	482.7	3.5e-145	ribose-phosphate pyrophosphokinase [EC:2.7.6.1]
CP049783.1_1639	K04042	321.87	680.8	7.4e-205	bifunctional UDP-N-acetylglucosamine pyrophosphorylase / glucosamine-1-phosphate N-acetyltransferase [EC:2.7.7.23 2.3.1.157]
CP049783.1_1640	K06412	27.70	159.3	1.6e-47	stage V sporulation protein G
CP049783.1_1641	K09685	258.10	403.3	1e-121	HTH-type transcriptional regulator, purine operon repressor
CP049783.1_1642	K00919	102.90	316.1	9.6e-95	4-diphosphocytidyl-2-C-methyl-D-erythritol kinase [EC:2.7.1.148]
CP049783.1_1643	K06423	62.50	103.0	3.4e-30	small acid-soluble spore protein F (minor alpha/beta-type SASP)
CP049783.1_1645	K06436	272.67	379.6	2.5e-114	spore coat assemly protein
CP049783.1_1646	K02528	256.10	373.5	4.1e-112	16S rRNA (adenine1518-N6/adenine1519-N6)-dimethyltransferase [EC:2.1.1.182]
CP049783.1_1647	K05985	117.63	277.6	2.4e-83	ribonuclease M5 [EC:3.1.26.8]
CP049783.1_1649	K03424	86.70	316.0	1.4e-94	TatD DNase family protein [EC:3.1.21.-]
CP049783.1_1650	K06885	290.33	454.9	1.5e-136	uncharacterized protein
CP049783.1_1651	K06284	86.83	141.2	4.2e-42	AbrB family transcriptional regulator, transcriptional pleiotropic regulator of transition state genes
CP049783.1_1652	K07056	92.80	372.7	5.7e-112	16S rRNA (cytidine1402-2'-O)-methyltransferase [EC:2.1.1.198]
CP049783.1_1653	K15460	135.00	152.3	5.4e-45	tRNA1Val (adenine37-N6)-methyltransferase [EC:2.1.1.223]
CP049783.1_1656	K02341	230.10	261.5	6e-78	DNA polymerase III subunit delta' [EC:2.7.7.7]
CP049783.1_1657	K09770	68.60	185.7	2.5e-55	uncharacterized protein
CP049783.1_1659	K00943	96.67	257.2	8e-77	dTMP kinase [EC:2.7.4.9]
CP049783.1_1660	K01585	258.33	406.9	6.3e-122	arginine decarboxylase [EC:4.1.1.19]
CP049783.1_1666	K02469	1032.73	1335.4	0	DNA gyrase subunit A [EC:5.6.2.2]
CP049783.1_1668	K02470	944.40	1106.1	0	DNA gyrase subunit B [EC:5.6.2.2]
CP049783.1_1669	K24648	42.73	102.6	5.6e-30	extracellular matrix regulatory protein B
CP049783.1_1670	K03629	104.40	158.0	1.2e-46	DNA replication and repair protein RecF
CP049783.1_1671	K03629	104.40	258.3	4.6e-77	DNA replication and repair protein RecF
CP049783.1_1672	K14761	48.70	108.2	1.5e-31	ribosome-associated protein

CP049783.1_1673	K02338	60.60	373.7	5.1e-112	DNA polymerase III subunit beta [EC:2.7.7.7]
CP049783.1_1674	K02313	129.33	684.6	4.1e-206	chromosomal replication initiator protein
CP049783.1_1675	K02914	23.97	71.1	3e-20	large subunit ribosomal protein L34
CP049783.1_1676	K03536	40.13	113.0	6.3e-33	ribonuclease P protein component [EC:3.1.26.5]
CP049783.1_1677	K03217	121.07	267.7	4.9e-80	YidC/Oxa1 family membrane protein insertase
CP049783.1_1678	K06346	71.00	252.7	1.6e-75	spoIIIJ-associated protein
CP049783.1_1679	K03650	165.63	538.8	7.1e-162	tRNA modification GTPase [EC:3.6.-.-]
CP049783.1_1680	K03495	422.67	1043.0	4.3e-314	tRNA uridine 5-carboxymethylaminomethyl modification enzyme
CP049783.1_1681	K03501	59.80	266.6	1.1e-79	16S rRNA (guanine527-N7)-methyltransferase [EC:2.1.1.170]
CP049783.1_1682	K03497	54.23	242.5	2.8e-72	ParB family transcriptional regulator, chromosome partitioning protein
CP049783.1_1683	K03496	131.53	349.6	9.9e-105	chromosome partitioning protein
CP049783.1_1684	K03497	54.23	273.2	1.3e-81	ParB family transcriptional regulator, chromosome partitioning protein
CP049783.1_1687	K22044	182.20	280.8	6.9e-84	moderate conductance mechanosensitive channel
CP049783.1_1691	K02990	24.13	100.5	2.8e-29	small subunit ribosomal protein S6
CP049783.1_1692	K03111	28.83	162.0	8.1e-48	single-strand DNA-binding protein
CP049783.1_1693	K02963	75.13	100.9	2.6e-29	small subunit ribosomal protein S18
CP049783.1_1694	K02035	249.67	366.9	6.9e-110	peptide/nickel transport system substrate-binding protein
CP049783.1_1695	K02033	263.63	395.2	1.4e-118	peptide/nickel transport system permease protein
CP049783.1_1696	K02034	256.80	365.4	1.3e-109	peptide/nickel transport system permease protein
CP049783.1_1697	K02031	412.13	489.8	3.9e-147	peptide/nickel transport system ATP-binding protein
CP049783.1_1697	K02032	420.67	421.7	2.1e-126	peptide/nickel transport system ATP-binding protein
CP049783.1_1698	K02032	420.67	531.7	1.1e-159	peptide/nickel transport system ATP-binding protein
CP049783.1_1699	K07258	223.10	275.0	4.1e-82	serine-type D-Ala-D-Ala carboxypeptidase (penicillin-binding protein 5/6) [EC:3.4.16.4]
CP049783.1_1700	K01005	193.97	256.6	1.7e-76	polyisoprenyl-teichoic acid--peptidoglycan teichoic acid transferase [EC:2.7.8.-]
CP049783.1_1704	K22927	277.33	844.6	1.7e-254	cyclic-di-AMP phosphodiesterase [EC:3.1.4.59]
CP049783.1_1705	K02939	60.20	142.7	5.4e-42	large subunit ribosomal protein L9
CP049783.1_1706	K02314	110.70	653.5	1.1e-196	replicative DNA helicase [EC:5.6.2.3]
CP049783.1_1707	K01939	131.03	653.5	9.5e-197	adenylosuccinate synthase [EC:6.3.4.4]
CP049783.1_1710	K07668	340.87	372.4	3.2e-112	two-component system, OmpR family, response regulator VicR
CP049783.1_1710	K02483	242.00	266.9	1.2e-79	two-component system, OmpR family, response regulator
CP049783.1_1711	K07652	474.87	628.9	4.6e-189	two-component system, OmpR family, sensor histidine kinase VicK [EC:2.7.13.3]
CP049783.1_1711	K07636	310.93	376.5	6.3e-113	two-component system, OmpR family, phosphate regulon sensor histidine kinase PhoR [EC:2.7.13.3]
CP049783.1_1711	K02484	312.80	327.1	8.3e-98	two-component system, OmpR family, sensor kinase [EC:2.7.13.3]
CP049783.1_1717	K01179	20.50	44.7	2.8e-12	endoglucanase [EC:3.2.1.4]
CP049783.1_1723	K07729	60.67	64.9	2.5e-18	putative transcriptional regulator
CP049783.1_1725	K00783	35.63	203.1	2e-60	23S rRNA (pseudouridine1915-N3)-methyltransferase [EC:2.1.1.177]
CP049783.1_1727	K01990	262.37	327.3	5e-98	ABC-2 type transport system ATP-binding protein
CP049783.1_1736	K03406	65.50	227.2	1.4e-67	methyl-accepting chemotaxis protein
CP049783.1_1740	K04750	56.87	122.0	1e-35	PhnB protein
CP049783.1_1741	K18220	608.17	853.5	4.1e-257	ribosomal protection tetracycline resistance protein
CP049783.1_1749	K18926	574.20	576.0	1.7e-173	MFS transporter, DHA2 family, lincomycin resistance protein
CP049783.1_1752	K02103	274.17	471.9	5.9e-142	GntR family transcriptional regulator, arabinose operon transcriptional repressor
CP049783.1_1754	K03077	237.90	360.3	3.1e-108	L-ribulose-5-phosphate 4-epimerase [EC:5.1.3.4]
CP049783.1_1755	K01804	182.77	676.3	5.8e-204	L-arabinose isomerase [EC:5.3.1.4]
CP049783.1_1757	K03088	96.50	97.7	2.2e-28	RNA polymerase sigma-70 factor, ECF subfamily
CP049783.1_1760	K00116	212.20	848.8	8.5e-256	malate dehydrogenase (quinone) [EC:1.1.5.4]
CP049783.1_1761	K16137	104.80	133.2	5e-39	TetR/AcrR family transcriptional regulator, transcriptional repressor for nem operon
CP049783.1_1765	K03088	96.50	114.4	1.9e-33	RNA polymerase sigma-70 factor, ECF subfamily
CP049783.1_1767	K05592	531.43	550.8	1.3e-165	ATP-dependent RNA helicase DeaD [EC:5.6.2.7]
CP049783.1_1769	K16137	104.80	127.3	3e-37	TetR/AcrR family transcriptional regulator, transcriptional repressor for nem operon
CP049783.1_1771	K14731	186.50	202.6	3.7e-60	epsilon-lactone hydrolase [EC:3.1.1.83]
CP049783.1_1772	K01181	91.70	268.1	4.1e-80	endo-1,4-beta-xylanase [EC:3.2.1.8]
CP049783.1_1774	K03297	108.13	141.2	1.4e-41	small multidrug resistance pump
CP049783.1_1775	K03299	88.80	513.2	2.1e-154	gluconate:H+ symporter, GntP family
CP049783.1_1776	K25031	463.90	763.7	2.9e-230	gluconokinase [EC:2.7.1.12]
CP049783.1_1777	K11476	166.83	354.6	6.1e-107	GntR family transcriptional regulator, gluconate operon transcriptional repressor

CP049783.1_1778	K03299	88.80	201.8	4.8e-60	gluconate:H+ symporter, GntP family
CP049783.1_1779	K00865	57.57	546.3	2.1e-164	glycerate 2-kinase [EC:2.7.1.165]
CP049783.1_1780	K02647	173.57	288.1	3.8e-86	carbohydrate diacid regulator
CP049783.1_1782	K02435	35.93	47.8	2.9e-13	aspartyl-tRNA(Asn)/glutamyl-tRNA(Gln) amidotransferase subunit C [EC:6.3.5.6 6.3.5.7]
CP049783.1_1784	K02538	499.37	875.4	7.4e-264	mannose operon transcriptional activator
CP049783.1_1785	K25814	717.47	1026.7	1.1e-309	mannose PTS system EIIBCA component [EC:2.7.1.191]
CP049783.1_1785	K02768	144.33	172.2	4.5e-51	fructose PTS system EIIA component [EC:2.7.1.202]
CP049783.1_1787	K00981	43.57	82.7	9.4e-24	phosphatidate cytidylyltransferase [EC:2.7.7.41]
CP049783.1_1789	K07029	147.20	254.9	3.6e-76	diacylglycerol kinase (ATP) [EC:2.7.1.107]
CP049783.1_1790	K19267	112.87	349.6	9.7e-105	NAD(P)H dehydrogenase (quinone) [EC:1.6.5.2]
CP049783.1_1791	K06193	38.80	207.8	2.4e-62	protein PhnA
CP049783.1_1796	K07092	58.83	66.9	4.7e-19	uncharacterized protein
CP049783.1_1799	K23107	334.20	345.6	1.5e-103	1-deoxyxylulose-5-phosphate synthase [EC:1.1.-.-]
CP049783.1_1799	K19265	304.10	337.9	3.3e-101	L-glyceraldehyde 3-phosphate reductase [EC:1.1.1.-]
CP049783.1_1801	K11632	471.07	611.2	1e-183	bacitracin transport system permease protein
CP049783.1_1802	K11631	388.27	416.0	1.4e-125	bacitracin transport system ATP-binding protein
CP049783.1_1802	K02003	292.97	300.6	5.6e-90	putative ABC transport system ATP-binding protein
CP049783.1_1803	K11629	365.30	519.6	1e-156	two-component system, OmpR family, bacitracin resistance sensor histidine kinase BceS [EC:2.7.13.3]
CP049783.1_1804	K11630	356.10	430.8	3.1e-130	two-component system, OmpR family, bacitracin resistance response regulator BceR
CP049783.1_1807	K05275	257.70	314.4	3.8e-94	pyridoxine 4-dehydrogenase [EC:1.1.1.65]
CP049783.1_1809	K07034	31.40	129.2	6.3e-38	uncharacterized protein
CP049783.1_1815	K00059	269.80	310.9	4.1e-93	3-oxoacyl-[acyl-carrier protein] reductase [EC:1.1.1.100]
CP049783.1_1817	K00432	54.07	216.1	1.7e-64	glutathione peroxidase [EC:1.11.1.9]
CP049783.1_1819	K00059	269.80	274.9	3.5e-82	3-oxoacyl-[acyl-carrier protein] reductase [EC:1.1.1.100]
CP049783.1_1820	K21960	245.43	424.3	7.2e-128	LysR family transcriptional regulator, regulator of the ytmI operon
CP049783.1_1821	K13653	208.93	324.9	2.7e-97	AraC family transcriptional regulator
CP049783.1_1823	K06941	65.87	466.8	3e-140	23S rRNA (adenine2503-C2)-methyltransferase [EC:2.1.1.192]
CP049783.1_1824	K02823	242.20	249.0	1.9e-74	dihydroorotate dehydrogenase electron transfer subunit
CP049783.1_1825	K17828	251.20	424.8	7.5e-128	dihydroorotate dehydrogenase (NAD+) catalytic subunit [EC:1.3.1.14]
CP049783.1_1829	K25634	420.53	538.8	1.2e-162	2-oxoglutarate reductase [EC:1.1.1.399]
CP049783.1_1830	K24180	175.77	232.0	3.5e-69	malate permease and related proteins
CP049783.1_1831	K00547	285.53	444.3	1.6e-133	homocysteine S-methyltransferase [EC:2.1.1.10]
CP049783.1_1834	K01629	182.03	412.8	1.4e-124	rhamnulose-1-phosphate aldolase [EC:4.1.2.19]
CP049783.1_1835	K01813	527.17	724.7	1.1e-218	L-rhamnose isomerase [EC:5.3.1.14]
CP049783.1_1836	K00848	300.13	663.5	9.6e-200	rhamnulokinase [EC:2.7.1.5]
CP049783.1_1837	K03816	140.40	292.7	2.7e-88	xanthine phosphoribosyltransferase [EC:2.4.2.22]
CP049783.1_1838	K16169	599.97	644.7	1.7e-194	xanthine permease
CP049783.1_1838	K24206	593.73	596.1	2.5e-179	uric acid transporter
CP049783.1_1839	K02035	249.67	280.0	1.3e-83	peptide/nickel transport system substrate-binding protein
CP049783.1_1840	K02033	263.63	356.2	9.4e-107	peptide/nickel transport system permease protein
CP049783.1_1841	K02034	256.80	312.6	1.4e-93	peptide/nickel transport system permease protein
CP049783.1_1844	K15584	473.80	746.0	5.9e-225	nickel transport system substrate-binding protein
CP049783.1_1844	K02035	249.67	364.5	3.6e-109	peptide/nickel transport system substrate-binding protein
CP049783.1_1845	K15585	403.27	457.4	6.3e-138	nickel transport system permease protein
CP049783.1_1845	K02033	263.63	388.9	1.1e-116	peptide/nickel transport system permease protein
CP049783.1_1846	K15586	362.70	380.8	9.8e-115	nickel transport system permease protein
CP049783.1_1846	K02034	256.80	326.9	6.4e-98	peptide/nickel transport system permease protein
CP049783.1_1847	K15587	339.47	373.6	1.4e-112	nickel transport system ATP-binding protein [EC:7.2.2.11]
CP049783.1_1848	K10824	311.43	359.2	4.1e-108	nickel transport system ATP-binding protein [EC:7.2.2.11]
CP049783.1_1849	K02030	76.77	157.8	1.4e-46	polar amino acid transport system substrate-binding protein
CP049783.1_1850	K16959	256.10	277.3	2.6e-83	L-cystine transport system permease protein
CP049783.1_1850	K02029	214.53	230.0	1.6e-68	polar amino acid transport system permease protein
CP049783.1_1851	K02028	386.70	416.1	6.3e-125	polar amino acid transport system ATP-binding protein [EC:7.4.2.1]
CP049783.1_1852	K01424	50.80	310.6	7.7e-93	L-asparaginase [EC:3.5.1.1]
CP049783.1_1857	K11925	310.70	599.8	2e-180	SgrR family transcriptional regulator
CP049783.1_1863	K02760	71.37	133.9	1.7e-39	cellobiose PTS system EIIB component [EC:2.7.1.196 2.7.1.205]

CP049783.1_1864	K02759	33.93	131.3	8.5e-39	cellobiose PTS system EIIA component [EC:2.7.1.196 2.7.1.205]
CP049783.1_1865	K02761	189.70	502.4	4.6e-151	cellobiose PTS system EIIC component
CP049783.1_1866	K01223	479.40	544.2	8.8e-164	6-phospho-beta-glucosidase [EC:3.2.1.86]
CP049783.1_1867	K03492	234.97	326.4	2.7e-98	GntR family transcriptional regulator, regulator of glucomannan utilization
CP049783.1_1869	K05916	302.67	617.1	8.6e-186	nitric oxide dioxygenase [EC:1.14.12.17]
CP049783.1_1870	K01875	89.73	506.7	3.9e-152	seryl-tRNA synthetase [EC:6.1.1.11]
CP049783.1_1873	K03308	387.23	500.6	1.6e-150	neurotransmitter:Na+ symporter, NSS family
CP049783.1_1875	K22278	73.27	134.2	2e-39	peptidoglycan-N-acetylglucosamine deacetylase [EC:3.5.1.104]
CP049783.1_1877	K15532	93.77	472.6	5.9e-142	unsaturated rhamnogalacturonyl hydrolase [EC:3.2.1.172]
CP049783.1_1878	K16210	366.43	674.8	2.5e-203	oligogalacturonide transporter
CP049783.1_1881	K03100	114.77	209.5	2.2e-62	signal peptidase I [EC:3.4.21.89]
CP049783.1_1883	K10119	296.93	311.8	1.7e-93	raffinose/stachyose/melibiose transport system permease protein
CP049783.1_1884	K10118	333.37	333.4	5.3e-100	raffinose/stachyose/melibiose transport system permease protein
CP049783.1_1884	K02025	276.90	284.4	3.7e-85	multiple sugar transport system permease protein
CP049783.1_1885	K07718	273.17	428.2	2.4e-128	two-component system, sensor histidine kinase YesM [EC:2.7.13.3]
CP049783.1_1886	K07720	156.87	328.9	2.4e-98	two-component system, response regulator YesN
CP049783.1_1887	K10117	246.87	268.1	7.2e-80	raffinose/stachyose/melibiose transport system substrate-binding protein
CP049783.1_1890	K06871	174.27	298.0	4.6e-89	uncharacterized protein
CP049783.1_1892	K19309	416.63	416.9	1.5e-125	bacitracin transport system ATP-binding protein
CP049783.1_1892	K01990	262.37	293.8	7.6e-88	ABC-2 type transport system ATP-binding protein
CP049783.1_1893	K19310	132.03	144.5	1.2e-42	bacitracin transport system permease protein
CP049783.1_1894	K19310	132.03	174.6	8.3e-52	bacitracin transport system permease protein
CP049783.1_1895	K01176	222.80	285.0	3.8e-85	alpha-amylase [EC:3.2.1.1]
CP049783.1_1896	K01235	519.60	1094.0	0	alpha-glucuronidase [EC:3.2.1.139]
CP049783.1_1897	K22268	169.53	1120.2	0	xylan 1,4-beta-xylosidase [EC:3.2.1.37]
CP049783.1_1903	K06884	16.83	86.7	7.7e-25	uncharacterized protein
CP049783.1_1904	K02069	66.73	238.1	7e-71	UDP-glucose/iron transport system permease protein
CP049783.1_1905	K02068	249.20	292.1	2e-87	UDP-glucose/iron transport system ATP-binding protein
CP049783.1_1910	K21562	197.07	241.2	4.7e-72	CRP/FNR family transcriptional regulator, anaerobic regulatory protein
CP049783.1_1910	K10914	140.63	160.7	2.3e-47	CRP/FNR family transcriptional regulator, cyclic AMP receptor protein
CP049783.1_1915	K07720	156.87	377.2	5.4e-113	two-component system, response regulator YesN
CP049783.1_1916	K07718	273.17	435.9	1.1e-130	two-component system, sensor histidine kinase YesM [EC:2.7.13.3]
CP049783.1_1917	K17318	176.50	273.9	1.1e-81	putative aldouronate transport system substrate-binding protein
CP049783.1_1918	K17319	220.03	434.5	1.1e-130	putative aldouronate transport system permease protein
CP049783.1_1920	K17320	270.87	354.6	1.3e-106	putative aldouronate transport system permease protein
CP049783.1_1921	K01181	91.70	372.3	1.1e-111	endo-1,4-beta-xylanase [EC:3.2.1.8]
CP049783.1_1922	K08094	107.17	243.9	4.7e-73	6-phospho-3-hexuloisomerase [EC:5.3.1.27]
CP049783.1_1923	K08093	178.23	277.2	5.2e-83	3-hexulose-6-phosphate synthase [EC:4.1.2.43]
CP049783.1_1926	K03406	65.50	95.4	1.1e-27	methyl-accepting chemotaxis protein
CP049783.1_1927	K01258	167.60	507.5	1.3e-152	tripeptide aminopeptidase [EC:3.4.11.4]
CP049783.1_1930	K01834	148.40	327.9	2.5e-98	2,3-bisphosphoglycerate-dependent phosphoglycerate mutase [EC:5.4.2.11]
CP049783.1_1931	K01176	222.80	263.5	1.3e-78	alpha-amylase [EC:3.2.1.1]
CP049783.1_1935	K03885	203.30	350.4	7.5e-105	NADH:quinone reductase (non-electrogenic) [EC:1.6.5.9]
CP049783.1_1936	K06113	228.80	412.0	1.3e-123	arabinan endo-1,5-alpha-L-arabinosidase [EC:3.2.1.99]
CP049783.1_1943	K16212	542.30	737.8	3e-222	4-O-beta-D-mannosyl-D-glucose phosphorylase [EC:2.4.1.281]
CP049783.1_1944	K16213	365.10	528.1	5.2e-159	cellobiose epimerase [EC:5.1.3.11]
CP049783.1_1945	K10117	246.87	249.3	3.8e-74	raffinose/stachyose/melibiose transport system substrate-binding protein
CP049783.1_1948	K18785	371.53	464.5	3.6e-140	beta-1,4-mannooligosaccharide/beta-1,4-mannosyl-N-acetylglucosamine phosphorylase [EC:2.4.1.319 2.4.1.320]
CP049783.1_1949	K01060	117.80	361.5	1.8e-108	cephalosporin-C deacetylase [EC:3.1.1.41]
CP049783.1_1950	K18567	389.93	488.8	2.8e-147	MFS transporter, DHA1 family, purine base/nucleoside efflux pump
CP049783.1_1952	K06158	558.03	596.2	3.1e-179	ATP-binding cassette, subfamily F, member 3
CP049783.1_1953	K01990	262.37	273.6	9.8e-82	ABC-2 type transport system ATP-binding protein
CP049783.1_1956	K10117	246.87	303.8	1.1e-90	raffinose/stachyose/melibiose transport system substrate-binding protein
CP049783.1_1957	K02025	276.90	281.2	3.3e-84	multiple sugar transport system permease protein
CP049783.1_1958	K10119	296.93	306.8	5.4e-92	raffinose/stachyose/melibiose transport system permease protein
CP049783.1_1960	K03088	96.50	130.7	2.1e-38	RNA polymerase sigma-70 factor, ECF subfamily

CP049783.1_1961	K19745	190.73	486.5	1.3e-146	acrylyl-CoA reductase (NADPH) [EC:1.3.1.-]
CP049783.1_1962	K26937	267.43	322.1	1.6e-96	MATE family, multidrug efflux pump
CP049783.1_1963	K02525	324.67	360.8	2.3e-108	LacI family transcriptional regulator, kdg operon repressor
CP049783.1_1963	K02529	268.37	292.3	2.5e-87	LacI family transcriptional regulator, galactose operon repressor
CP049783.1_1964	K00874	245.93	365.3	2.6e-109	2-dehydro-3-deoxygluconokinase [EC:2.7.1.45]
CP049783.1_1965	K01815	116.77	443.0	1.1e-133	4-deoxy-L-threo-5-hexosulose-uronate ketol-isomerase [EC:5.3.1.17]
CP049783.1_1966	K01625	193.93	253.0	1.9e-75	2-dehydro-3-deoxyphosphogluconate aldolase / (4S)-4-hydroxy-2-oxoglutarate aldolase [EC:4.1.2.14 4.1.3.42]
CP049783.1_1967	K00065	334.50	408.2	8.1e-123	2-dehydro-3-deoxy-D-gluconate 5-dehydrogenase [EC:1.1.1.127]
CP049783.1_1968	K03449	127.83	454.1	2.2e-136	MFS transporter, CP family, cyanate transporter
CP049783.1_1972	K07486	31.47	230.1	1.7e-68	transposase
CP049783.1_1974	K26730	335.73	427.6	2.6e-128	Na+:H+ antiporter
CP049783.1_1975	K07118	124.57	267.8	3.6e-80	uncharacterized protein
CP049783.1_1976	K14731	186.50	190.9	1.4e-56	epsilon-lactone hydrolase [EC:3.1.1.83]
CP049783.1_1978	K02443	84.13	255.2	1.2e-76	glycerol uptake operon antiterminator
CP049783.1_1979	K00864	510.13	770.2	4.8e-232	glycerol kinase [EC:2.7.1.30]
CP049783.1_1980	K00111	151.87	540.5	1.6e-162	glycerol-3-phosphate dehydrogenase [EC:1.1.5.3]
CP049783.1_1982	K06518	37.43	148.0	7.8e-44	holin-like protein
CP049783.1_1984	K18967	236.10	305.8	1.8e-91	diguanylate cyclase [EC:2.7.7.65]
CP049783.1_1985	K10844	382.27	486.9	3.7e-146	DNA excision repair protein ERCC-2 [EC:5.6.2.3]
CP049783.1_2005	K02055	194.27	200.6	1.9e-59	putative spermidine/putrescine transport system substrate-binding protein
CP049783.1_2007	K02054	291.63	302.8	1.3e-90	putative spermidine/putrescine transport system permease protein
CP049783.1_2008	K02053	239.17	267.0	6.2e-80	putative spermidine/putrescine transport system permease protein
CP049783.1_2010	K01486	325.67	629.5	1.6e-189	adenine deaminase [EC:3.5.4.2]
CP049783.1_2011	K04047	77.30	176.0	4.2e-52	starvation-inducible DNA-binding protein
CP049783.1_2012	K00491	521.20	612.8	1.3e-184	nitric-oxide synthase, bacterial [EC:1.14.14.47]
CP049783.1_2015	K06284	86.83	107.1	1.5e-31	AbrB family transcriptional regulator, transcriptional pleiotropic regulator of transition state genes
CP049783.1_2016	K07124	220.10	220.5	1.1e-65	uncharacterized protein
CP049783.1_2017	K11621	70.10	115.9	5.9e-34	lia operon protein LiaG
CP049783.1_2019	K10947	65.70	122.1	1.1e-35	PadR family transcriptional regulator
CP049783.1_2021	K00549	27.93	1005.4	7.7e-303	5-methyltetrahydropteroyltriglutamate--homocysteine methyltransferase [EC:2.1.1.14]
CP049783.1_2022	K04068	118.80	193.1	1.9e-57	anaerobic ribonucleoside-triphosphate reductase activating protein [EC:1.97.1.4]
CP049783.1_2023	K21636	237.07	498.4	1.1e-149	ribonucleoside-triphosphate reductase (formate) [EC:1.1.98.6]
CP049783.1_2024	K06284	86.83	113.2	2e-33	AbrB family transcriptional regulator, transcriptional pleiotropic regulator of transition state genes
CP049783.1_2025	K05349	305.00	717.7	7.7e-216	beta-glucosidase [EC:3.2.1.21]
CP049783.1_2026	K19587	106.40	108.6	6.7e-32	Rrf2 family transcriptional regulator, repressor of oqxAB
CP049783.1_2027	K18967	236.10	321.5	2.9e-96	diguanylate cyclase [EC:2.7.7.65]
CP049783.1_2028	K06295	452.20	725.4	3e-218	spore germination protein KA
CP049783.1_2029	K06297	281.23	353.4	6.4e-106	spore germination protein KC
CP049783.1_2030	K06296	248.90	305.3	2.2e-91	spore germination protein KB
CP049783.1_2031	K06296	248.90	301.8	2.4e-90	spore germination protein KB
CP049783.1_2035	K02171	45.30	81.8	1.8e-23	BlaI family transcriptional regulator, penicillinase repressor
CP049783.1_2036	K02172	69.77	87.0	2.8e-25	bla regulator protein blaR1
CP049783.1_2038	K03406	65.50	311.2	5.1e-93	methyl-accepting chemotaxis protein
CP049783.1_2040	K21959	245.10	327.6	1.9e-98	LysR family transcriptional regulator, cell division regulator
CP049783.1_2041	K06911	36.97	161.1	1.5e-47	quercetin 2,3-dioxygenase [EC:1.13.11.24]
CP049783.1_2043	K06307	659.47	728.0	1.4e-219	spore germination protein
CP049783.1_2043	K06295	452.20	727.5	7.1e-219	spore germination protein KA
CP049783.1_2044	K06297	281.23	337.0	6e-101	spore germination protein KC
CP049783.1_2044	K06308	307.50	314.9	2.2e-94	spore germination protein
CP049783.1_2047	K06309	283.30	286.1	1.2e-85	spore germination protein
CP049783.1_2048	K01719	43.23	91.8	1.6e-26	uroporphyrinogen-III synthase [EC:4.2.1.75]
CP049783.1_2049	K22522	96.03	230.8	9.3e-69	cytokinin riboside 5'-monophosphate phosphoribohydrolase [EC:3.2.2.-]
CP049783.1_2050	K21479	737.20	950.7	2.6e-286	cobalt-factor III methyltransferase [EC:2.1.1.272]
CP049783.1_2050	K05934	360.60	443.0	4.3e-133	precorrin-3B C17-methyltransferase / cobalt-factor III methyltransferase [EC:2.1.1.131 2.1.1.272]
CP049783.1_2051	K03795	119.77	404.1	2.7e-121	sirohydrochlorin cobaltochelatase [EC:4.99.1.3]
CP049783.1_2051	K03794	134.27	143.2	3e-42	sirohydrochlorin ferrochelatase [EC:4.99.1.4]

CP049783.1_2052	K05895	127.10	251.3	5.1e-75	precorrin-6A/cobalt-precorrin-6A reductase [EC:1.3.1.54 1.3.1.106]
CP049783.1_2053	K06042	81.73	275.1	1.9e-82	precorrin-8X/cobalt-precorrin-8 methylmutase [EC:5.4.99.61 5.4.99.60]
CP049783.1_2054	K02188	136.40	474.1	1.7e-142	cobalt-precorrin-5B (C1)-methyltransferase [EC:2.1.1.195]
CP049783.1_2055	K00595	224.23	465.5	7.2e-140	precorrin-6B C5,15-methyltransferase / cobalt-precorrin-6B C5,C15-methyltransferase [EC:2.1.1.132 2.1.1.289 2.1.1.196]
CP049783.1_2056	K03394	133.17	306.6	6.3e-92	precorrin-2/cobalt-factor-2 C20-methyltransferase [EC:2.1.1.130 2.1.1.151]
CP049783.1_2057	K05936	234.40	402.2	8.5e-121	precorrin-4/cobalt-precorrin-4 C11-methyltransferase [EC:2.1.1.133 2.1.1.271]
CP049783.1_2058	K02189	39.97	366.6	4.8e-110	cobalt-precorrin 5A hydrolase [EC:3.7.1.12]
CP049783.1_2059	K02224	108.67	665.2	2.4e-200	cobyrinic acid a,c-diamide synthase [EC:6.3.5.9 6.3.5.11]
CP049783.1_2060	K06284	86.83	113.5	1.6e-33	AbrB family transcriptional regulator, transcriptional pleiotropic regulator of transition state genes
CP049783.1_2061	K09992	76.67	157.5	1.6e-46	uncharacterized protein
CP049783.1_2063	K03688	417.90	602.7	2.4e-181	ubiquinone biosynthesis protein
CP049783.1_2063	K08869	325.10	335.6	1.6e-100	aarF domain-containing kinase
CP049783.1_2067	K03321	345.37	531.4	1.1e-159	sulfate permease, SulP family
CP049783.1_2070	K02028	386.70	441.7	1.1e-132	polar amino acid transport system ATP-binding protein [EC:7.4.2.1]
CP049783.1_2071	K02030	76.77	128.1	1.5e-37	polar amino acid transport system substrate-binding protein
CP049783.1_2088	K05592	531.43	577.9	8.3e-174	ATP-dependent RNA helicase DeaD [EC:5.6.2.7]
CP049783.1_2089	K13283	187.13	199.3	2.3e-59	ferrous-iron efflux pump FieF
CP049783.1_2090	K01005	193.97	320.2	8.3e-96	polyisoprenyl-teichoic acid--peptidoglycan teichoic acid transferase [EC:2.7.8.-]
CP049783.1_2093	K25290	398.40	434.6	3.5e-131	ferric hydroxamate/heme transport system ATP-binding protein [EC:7.2.2.16]
CP049783.1_2093	K02013	238.30	331.7	2.4e-99	iron complex transport system ATP-binding protein [EC:7.2.2.-]
CP049783.1_2094	K00432	54.07	244.7	3.4e-73	glutathione peroxidase [EC:1.11.1.9]
CP049783.1_2095	K05910	482.57	506.7	1.6e-152	NADH peroxidase [EC:1.11.1.1]
CP049783.1_2097	K21993	293.73	360.3	3.3e-108	formate transporter
CP049783.1_2102	K03817	131.60	232.3	2.8e-69	ribosomal-protein-serine acetyltransferase [EC:2.3.1.-]
CP049783.1_2104	K01886	500.40	909.0	1e-273	glutaminyl-tRNA synthetase [EC:6.1.1.18]
CP049783.1_2105	K05820	181.27	321.9	1.4e-96	MFS transporter, PPP family, 3-phenylpropionic acid transporter
CP049783.1_2108	K03752	92.73	213.1	1.8e-63	molybdenum cofactor guanylyltransferase [EC:2.7.7.77]
CP049783.1_2109	K06895	83.83	180.7	8.8e-54	L-lysine exporter family protein LysE/ArgO
CP049783.1_2113	K00917	332.87	339.0	6.1e-102	tagatose 6-phosphate kinase [EC:2.7.1.144]
CP049783.1_2114	K18925	114.07	116.3	2.2e-34	paired small multidrug resistance pump
CP049783.1_2115	K18924	121.87	169.4	1.2e-50	paired small multidrug resistance pump
CP049783.1_2117	K06131	262.77	508.1	1.3e-152	cardiolipin synthase A/B [EC:2.7.8.-]
CP049783.1_2119	K09936	41.73	95.2	1.3e-27	bacterial/archaeal transporter family-2 protein
CP049783.1_2120	K16326	165.60	236.7	6.2e-71	CRP/FNR family transcriptional regulator, putaive post-exponential-phase nitrogen-starvation regulator
CP049783.1_2121	K09936	41.73	107.2	2.9e-31	bacterial/archaeal transporter family-2 protein
CP049783.1_2122	K01823	34.03	49.8	1.1e-13	isopentenyl-diphosphate Delta-isomerase [EC:5.3.3.2]
CP049783.1_2124	K06942	430.83	551.7	5.9e-166	ribosome-binding ATPase
CP049783.1_2125	K02835	355.53	508.4	8.3e-153	peptide chain release factor 1
CP049783.1_2126	K02493	233.73	314.3	5e-94	release factor glutamine methyltransferase [EC:2.1.1.297]
CP049783.1_2128	K06387	65.47	238.7	2e-71	stage II sporulation protein R
CP049783.1_2129	K07566	93.50	428.8	9.7e-129	L-threonylcarbamoyladenylate synthase [EC:2.7.7.87]
CP049783.1_2130	K23242	61.23	175.0	5.6e-52	manganese efflux pump family protein
CP049783.1_2131	K20201	148.33	198.9	1.8e-59	protein arginine phosphatase [EC:3.9.1.2]
CP049783.1_2131	K25307	127.47	132.7	4.8e-39	low molecular weight protein-tyrosine phosphatase [EC:3.1.3.48]
CP049783.1_2133	K00600	75.20	683.5	9.2e-206	glycine hydroxymethyltransferase [EC:2.1.2.1]
CP049783.1_2134	K25087	353.50	418.3	4.5e-126	maltose transport system permease protein
CP049783.1_2135	K25088	335.73	368.9	5.2e-111	maltose transport system permease protein
CP049783.1_2135	K10119	296.93	309.2	1.1e-92	raffinose/stachyose/melibiose transport system permease protein
CP049783.1_2136	K25086	282.43	446.3	4.2e-134	maltose transport system substrate-binding protein
CP049783.1_2136	K10117	246.87	308.9	3.3e-92	raffinose/stachyose/melibiose transport system substrate-binding protein
CP049783.1_2136	K02027	193.17	201.2	1.2e-59	multiple sugar transport system substrate-binding protein
CP049783.1_2137	K00761	217.77	338.8	1.1e-101	uracil phosphoribosyltransferase [EC:2.4.2.9]
CP049783.1_2138	K01791	432.27	553.1	3e-166	UDP-N-acetylglucosamine 2-epimerase (non-hydrolysing) [EC:5.1.3.14]
CP049783.1_2141	K02108	158.57	225.7	3.4e-67	F-type H+-transporting ATPase subunit a
CP049783.1_2142	K02110	68.60	90.1	4.9e-26	F-type H+-transporting ATPase subunit c
CP049783.1_2143	K02109	43.13	127.4	2.8e-37	F-type H+-transporting ATPase subunit b

CP049783.1_2144	K02113	45.90	137.3	2.3e-40	F-type H+-transporting ATPase subunit delta
CP049783.1_2145	K02111	432.10	949.2	5.4e-286	F-type H+/Na+-transporting ATPase subunit alpha [EC:7.1.2.2 7.2.2.1]
CP049783.1_2146	K02115	246.97	403.0	3.5e-121	F-type H+-transporting ATPase subunit gamma
CP049783.1_2147	K02112	465.83	895.2	8.1e-270	F-type H+/Na+-transporting ATPase subunit beta [EC:7.1.2.2 7.2.2.1]
CP049783.1_2148	K02114	99.10	160.3	1.5e-47	F-type H+-transporting ATPase subunit epsilon
CP049783.1_2151	K00790	172.73	649.9	7.5e-196	UDP-N-acetylglucosamine 1-carboxyvinyltransferase [EC:2.5.1.7]
CP049783.1_2152	K06381	107.00	407.1	3.4e-122	stage II sporulation protein D (peptidoglycan lytic transglycosylase) [EC:4.2.2.29]
CP049783.1_2153	K06386	162.03	330.8	2.9e-99	stage II sporulation protein Q
CP049783.1_2154	K06283	55.80	135.5	1.6e-40	putative DeoR family transcriptional regulator, stage III sporulation protein D
CP049783.1_2155	K03569	108.73	525.6	2.4e-158	rod shape-determining protein MreB and related proteins
CP049783.1_2156	K02391	233.33	258.9	1.8e-77	flagellar basal-body rod protein FlgF
CP049783.1_2159	K02372	79.93	224.1	1.2e-66	3-hydroxyacyl-[acyl-carrier-protein] dehydratase [EC:4.2.1.59]
CP049783.1_2160	K01835	371.63	411.2	3e-123	phosphoglucomutase [EC:5.4.2.2]
CP049783.1_2162	K16710	141.17	179.0	4.2e-53	colanic acid/amylovoran biosynthesis protein WcaK/AmsJ
CP049783.1_2163	K05946	235.07	323.3	9.2e-97	N-acetylglucosaminyldiphosphoundecaprenol N-acetyl-beta-D-mannosaminyltransferase [EC:2.4.1.187]
CP049783.1_2164	K02851	237.03	401.7	1.2e-120	UDP-GlcNAc:undecaprenyl-phosphate/decaprenyl-phosphate GlcNAc-1-phosphate transferase [EC:2.7.8.33 2.7.8.35]
CP049783.1_2166	K18138	1073.20	1149.2	0	multidrug efflux pump
CP049783.1_2167	K03585	261.90	284.9	4.2e-85	membrane fusion protein, multidrug efflux system
CP049783.1_2167	K02005	227.43	229.9	2e-68	HlyD family secretion protein
CP049783.1_2172	K00789	19.03	681.1	3.7e-205	S-adenosylmethionine synthetase [EC:2.5.1.6]
CP049783.1_2177	K07777	248.37	422.4	8e-127	two-component system, NarL family, sensor histidine kinase DegS [EC:2.7.13.3]
CP049783.1_2178	K07692	255.17	325.7	5.3e-98	two-component system, NarL family, response regulator DegU
CP049783.1_2180	K02240	272.23	754.5	3.4e-227	competence protein ComFA
CP049783.1_2181	K02242	65.47	239.4	1.9e-71	competence protein ComFC
CP049783.1_2185	K02398	24.90	60.0	1.2e-16	negative regulator of flagellin synthesis FlgM
CP049783.1_2187	K02396	100.10	477.1	4.8e-143	flagellar hook-associated protein 1
CP049783.1_2188	K02397	144.10	273.0	1.3e-81	flagellar hook-associated protein 3 FlgL
CP049783.1_2189	K13626	52.33	138.7	6.7e-41	flagellar assembly factor FliW
CP049783.1_2190	K03563	23.50	94.5	1.8e-27	carbon storage regulator
CP049783.1_2192	K02406	132.37	336.8	4e-101	flagellin
CP049783.1_2193	K06603	27.00	90.3	5e-26	flagellar protein FlaG
CP049783.1_2194	K02407	69.27	364.2	5.1e-109	flagellar hook-associated protein 2
CP049783.1_2195	K02422	28.13	128.0	1.5e-37	flagellar secretion chaperone FliS
CP049783.1_2198	K15894	314.93	419.8	2.1e-126	UDP-N-acetylglucosamine 4,6-dehydratase/5-epimerase [EC:4.2.1.115 5.1.3.-]
CP049783.1_2199	K07257	112.70	337.6	2.2e-101	spore coat polysaccharide biosynthesis protein SpsF
CP049783.1_2200	K15897	137.87	343.6	4.7e-103	UDP-2,4-diacetamido-2,4,6-trideoxy-beta-L-altropyranose hydrolase [EC:3.6.1.57]
CP049783.1_2202	K15898	418.30	502.9	1.3e-151	pseudaminic acid synthase [EC:2.5.1.97]
CP049783.1_2204	K13010	413.10	436.5	5.3e-131	perosamine synthetase [EC:2.6.1.102]
CP049783.1_2205	K03704	80.73	117.9	1.4e-34	cold shock protein
CP049783.1_2207	K05808	97.77	239.8	6.5e-72	ribosome hibernation promoting factor
CP049783.1_2209	K03070	231.23	1243.8	0	preprotein translocase subunit SecA [EC:7.4.2.8]
CP049783.1_2210	K02836	378.07	559.4	3.1e-168	peptide chain release factor 2
CP049783.1_2212	K01053	182.60	221.4	7.1e-66	gluconolactonase [EC:3.1.1.17]
CP049783.1_2213	K00145	128.50	478.6	4.8e-144	N-acetyl-gamma-glutamyl-phosphate reductase [EC:1.2.1.38]
CP049783.1_2214	K00620	170.47	598.7	3.8e-180	glutamate N-acetyltransferase / amino-acid N-acetyltransferase [EC:2.3.1.35 2.3.1.1]
CP049783.1_2215	K00930	215.57	337.4	2.9e-101	acetylglutamate kinase [EC:2.7.2.8]
CP049783.1_2216	K00821	483.53	573.2	1.4e-172	acetylornithine/N-succinyldiaminopimelate aminotransferase [EC:2.6.1.11 2.6.1.17]
CP049783.1_2217	K00611	353.27	544.2	7.1e-164	ornithine carbamoyltransferase [EC:2.1.3.3]
CP049783.1_2218	K01940	115.50	689.2	2.1e-207	argininosuccinate synthase [EC:6.3.4.5]
CP049783.1_2219	K01755	169.37	761.5	3.7e-229	argininosuccinate lyase [EC:4.3.2.1]
CP049783.1_2221	K20327	722.70	726.7	7.1e-219	glycosyltransferase XagB
CP049783.1_2222	K20534	338.93	396.5	4.8e-119	polyisoprenyl-phosphate glycosyltransferase [EC:2.4.-.-]
CP049783.1_2226	K03930	171.97	361.7	8.6e-109	putative tributyrin esterase [EC:3.1.1.-]
CP049783.1_2228	K18346	73.17	161.7	7.4e-48	vancomycin resistance protein VanW
CP049783.1_2229	K09812	287.03	386.1	3.9e-116	cell division transport system ATP-binding protein
CP049783.1_2230	K09811	79.70	271.4	2.8e-81	cell division transport system permease protein

CP049783.1_2231	K22719	166.07	210.6	1.1e-62	murein hydrolase activator
CP049783.1_2232	K03797	127.93	411.8	1.8e-123	carboxyl-terminal processing protease [EC:3.4.21.102]
CP049783.1_2234	K00570	92.93	137.7	1.6e-40	phosphatidylethanolamine/phosphatidyl-N-methylethanolamine N-methyltransferase [EC:2.1.1.17 2.1.1.71]
CP049783.1_2236	K01218	70.20	345.0	3e-103	mannan endo-1,4-beta-mannosidase [EC:3.2.1.78]
CP049783.1_2240	K06376	38.70	49.8	8.4e-14	stage 0 sporulation regulatory protein
CP049783.1_2241	K02557	144.00	235.4	2.9e-70	chemotaxis protein MotB
CP049783.1_2242	K02556	81.20	318.5	2e-95	chemotaxis protein MotA
CP049783.1_2243	K03702	343.33	1161.7	0	excinuclease ABC subunit B
CP049783.1_2244	K03701	341.57	1480.7	0	excinuclease ABC subunit A
CP049783.1_2246	K00366	598.17	692.4	1.7e-208	ferredoxin-nitrite reductase [EC:1.7.7.1]
CP049783.1_2253	K20534	338.93	452.8	3.9e-136	polyisoprenyl-phosphate glycosyltransferase [EC:2.4.-.-]
CP049783.1_2254	K00963	76.77	146.4	4e-43	UTP--glucose-1-phosphate uridylyltransferase [EC:2.7.7.9]
CP049783.1_2255	K20483	108.60	926.5	7.1e-279	class I lanthipeptide synthase [EC:3.13.2.4]
CP049783.1_2256	K20484	130.10	327.1	7.1e-98	class I lanthipeptide synthase [EC:3.13.2.4]
CP049783.1_2259	K09692	218.47	271.0	4.1e-81	teichoic acid transport system permease protein
CP049783.1_2260	K09691	395.27	444.6	1.9e-133	homopolymeric O-antigen transport system ATP-binding protein [EC:7.5.2.14]
CP049783.1_2260	K09693	367.70	379.7	4.9e-114	teichoic acid transport system ATP-binding protein [EC:7.5.2.4]
CP049783.1_2261	K01953	106.30	412.1	1.7e-123	asparagine synthase (glutamine-hydrolysing) [EC:6.3.5.4]
CP049783.1_2262	K00980	122.07	185.0	3e-55	glycerol-3-phosphate cytidylyltransferase [EC:2.7.7.39]
CP049783.1_2263	K22844	181.20	193.6	1.7e-57	glucosyl-dolichyl phosphate glucuronosyltransferase [EC:2.4.1.356]
CP049783.1_2265	K00973	234.80	316.5	1.2e-94	glucose-1-phosphate thymidylyltransferase [EC:2.7.7.24]
CP049783.1_2266	K13663	168.23	256.7	1.1e-76	acyltransferase [EC:2.3.1.-]
CP049783.1_2268	K01790	200.03	291.6	2.4e-87	dTDP-4-dehydrorhamnose 3,5-epimerase [EC:5.1.3.13]
CP049783.1_2269	K01710	493.40	599.4	1.8e-180	dTDP-glucose 4,6-dehydratase [EC:4.2.1.46]
CP049783.1_2270	K00067	299.30	385.0	1.9e-115	dTDP-4-dehydrorhamnose reductase [EC:1.1.1.133]
CP049783.1_2272	K16870	147.83	287.9	5.3e-86	N-acetylglucosaminyl-diphospho-decaprenol L-rhamnosyltransferase [EC:2.4.1.289]
CP049783.1_2275	K07094	264.60	337.8	9.2e-102	heptaprenylglyceryl phosphate synthase [EC:2.5.1.-]
CP049783.1_2276	K03657	367.57	788.1	3.4e-237	ATP-dependent DNA helicase UvrD/PcrA [EC:5.6.2.4]
CP049783.1_2277	K01972	119.00	789.8	8.3e-238	DNA ligase (NAD+) [EC:6.5.1.2]
CP049783.1_2281	K03708	95.60	212.2	1.7e-63	transcriptional regulator of stress and heat shock response
CP049783.1_2282	K19411	58.37	225.9	1.4e-67	protein arginine kinase activator
CP049783.1_2283	K19405	208.57	503.3	1.5e-151	protein arginine kinase [EC:2.7.14.1]
CP049783.1_2284	K03696	1063.70	1259.9	0	ATP-dependent Clp protease ATP-binding subunit ClpC
CP049783.1_2285	K04485	124.17	698.4	2.3e-210	DNA repair protein RadA/Sms
CP049783.1_2286	K07067	327.90	509.1	2.4e-153	diadenylate cyclase [EC:2.7.7.85]
CP049783.1_2287	K17103	126.47	254.6	6.2e-76	CDP-diacylglycerol---serine O-phosphatidyltransferase [EC:2.7.8.8]
CP049783.1_2287	K19665	179.87	182.6	2.8e-54	archaetidylserine synthase [EC:2.7.8.38]
CP049783.1_2289	K00991	252.03	310.0	6.4e-93	2-C-methyl-D-erythritol 4-phosphate cytidylyltransferase [EC:2.7.7.60]
CP049783.1_2290	K01770	138.60	256.9	5.2e-77	2-C-methyl-D-erythritol 2,4-cyclodiphosphate synthase [EC:4.6.1.12]
CP049783.1_2291	K09698	647.87	746.5	5.8e-225	nondiscriminating glutamyl-tRNA synthetase [EC:6.1.1.24]
CP049783.1_2291	K01885	298.30	609.8	3.2e-183	glutamyl-tRNA synthetase [EC:6.1.1.17]
CP049783.1_2292	K00640	93.33	308.3	2.8e-92	serine O-acetyltransferase [EC:2.3.1.30]
CP049783.1_2293	K01883	400.67	663.4	1.8e-199	cysteinyl-tRNA synthetase [EC:6.1.1.16]
CP049783.1_2294	K11145	45.73	168.7	3e-50	mini-ribonuclease III [EC:3.1.26.-]
CP049783.1_2295	K03218	212.77	348.1	1.6e-104	23S rRNA (guanosine2251-2'-O)-methyltransferase [EC:2.1.1.185]
CP049783.1_2296	K06962	56.63	174.3	1.3e-51	uncharacterized protein
CP049783.1_2297	K27000	107.77	364.8	8.2e-110	RNA polymerase sigma-H factor
CP049783.1_2298	K03073	20.17	70.5	5.1e-20	preprotein translocase subunit SecE
CP049783.1_2299	K02601	101.20	261.0	4.1e-78	transcription termination/antitermination protein NusG
CP049783.1_2300	K02867	62.17	244.4	6.9e-73	large subunit ribosomal protein L11
CP049783.1_2301	K02863	85.67	314.0	4.6e-94	large subunit ribosomal protein L1
CP049783.1_2302	K02864	51.60	174.0	1.6e-51	large subunit ribosomal protein L10
CP049783.1_2303	K02935	22.77	155.8	5.9e-46	large subunit ribosomal protein L7/L12
CP049783.1_2304	K00564	110.40	225.9	2.8e-67	16S rRNA (guanine1207-N2)-methyltransferase [EC:2.1.1.172]
CP049783.1_2305	K03043	656.80	1844.6	0	DNA-directed RNA polymerase subunit beta [EC:2.7.7.6]
CP049783.1_2306	K03046	932.17	2139.8	0	DNA-directed RNA polymerase subunit beta' [EC:2.7.7.6]

CP049783.1_2307	K07590	76.57	100.7	8.9e-30	large subunit ribosomal protein L7A
CP049783.1_2308	K02950	75.93	244.5	2.9e-73	small subunit ribosomal protein S12
CP049783.1_2309	K02992	105.50	221.5	4.4e-66	small subunit ribosomal protein S7
CP049783.1_2310	K02355	521.90	1076.8	0	elongation factor G
CP049783.1_2311	K02358	336.50	780.3	2.4e-235	elongation factor Tu
CP049783.1_2312	K02946	61.53	156.7	3.3e-46	small subunit ribosomal protein S10
CP049783.1_2313	K02906	104.83	318.6	2.7e-95	large subunit ribosomal protein L3
CP049783.1_2314	K02926	92.37	288.3	2.7e-86	large subunit ribosomal protein L4
CP049783.1_2315	K02892	57.40	87.2	4.8e-25	large subunit ribosomal protein L23
CP049783.1_2316	K02886	164.10	422.3	5.9e-127	large subunit ribosomal protein L2
CP049783.1_2317	K02965	102.33	151.9	4.7e-45	small subunit ribosomal protein S19
CP049783.1_2318	K02890	79.73	180.9	8.8e-54	large subunit ribosomal protein L22
CP049783.1_2319	K02982	103.63	352.6	5.2e-106	small subunit ribosomal protein S3
CP049783.1_2320	K02878	90.63	243.7	7.7e-73	large subunit ribosomal protein L16
CP049783.1_2321	K02904	59.10	93.9	3.4e-27	large subunit ribosomal protein L29
CP049783.1_2322	K02961	80.63	130.2	2.4e-38	small subunit ribosomal protein S17
CP049783.1_2323	K02874	94.83	189.5	1.5e-56	large subunit ribosomal protein L14
CP049783.1_2324	K02895	56.73	148.5	8.4e-44	large subunit ribosomal protein L24
CP049783.1_2325	K02931	104.27	329.0	6.3e-99	large subunit ribosomal protein L5
CP049783.1_2326	K02954	26.53	71.6	2.5e-20	small subunit ribosomal protein S14
CP049783.1_2327	K02994	80.50	199.5	2.2e-59	small subunit ribosomal protein S8
CP049783.1_2328	K02933	107.80	266.4	9.7e-80	large subunit ribosomal protein L6
CP049783.1_2329	K02881	45.33	157.9	1e-46	large subunit ribosomal protein L18
CP049783.1_2330	K02988	134.97	254.0	6.9e-76	small subunit ribosomal protein S5
CP049783.1_2331	K02907	40.27	73.4	5.5e-21	large subunit ribosomal protein L30
CP049783.1_2332	K02876	42.80	216.9	1.5e-64	large subunit ribosomal protein L15
CP049783.1_2333	K03076	210.97	570.4	1.1e-171	preprotein translocase subunit SecY
CP049783.1_2334	K00939	185.70	238.6	4e-71	adenylate kinase [EC:2.7.4.3]
CP049783.1_2337	K02518	37.43	144.2	6.9e-43	translation initiation factor IF-1
CP049783.1_2338	K02919	24.63	59.5	1.5e-16	large subunit ribosomal protein L36
CP049783.1_2339	K02952	113.37	195.3	3.9e-58	small subunit ribosomal protein S13
CP049783.1_2340	K02948	100.47	195.8	3.3e-58	small subunit ribosomal protein S11
CP049783.1_2341	K03040	50.70	420.9	1.3e-126	DNA-directed RNA polymerase subunit alpha [EC:2.7.7.6]
CP049783.1_2342	K02879	39.00	200.5	1.5e-59	large subunit ribosomal protein L17
CP049783.1_2343	K06173	231.17	253.4	1.5e-75	tRNA pseudouridine38-40 synthase [EC:5.4.99.12]
CP049783.1_2344	K02871	66.60	221.4	5.6e-66	large subunit ribosomal protein L13
CP049783.1_2345	K02996	103.27	215.2	4.9e-64	small subunit ribosomal protein S9
CP049783.1_2346	K00390	157.87	335.3	1.2e-100	phosphoadenosine phosphosulfate reductase [EC:1.8.4.8 1.8.4.10]
CP049783.1_2347	K00958	267.50	545.7	4.4e-164	sulfate adenylyltransferase [EC:2.7.7.4]
CP049783.1_2348	K01448	29.13	204.0	9.9e-61	N-acetylmuramoyl-L-alanine amidase [EC:3.5.1.28]
CP049783.1_2349	K03593	111.47	370.4	5.3e-111	ATP-binding protein involved in chromosome partitioning
CP049783.1_2350	K06294	84.50	271.6	1.1e-81	spore germination protein D
CP049783.1_2351	K22278	73.27	203.6	1.8e-60	peptidoglycan-N-acetylglucosamine deacetylase [EC:3.5.1.104]
CP049783.1_2352	K06384	34.00	123.4	3.6e-36	stage II sporulation protein M
CP049783.1_2357	K01595	49.70	1097.4	0	phosphoenolpyruvate carboxylase [EC:4.1.1.31]
CP049783.1_2358	K03088	96.50	191.7	5.8e-57	RNA polymerase sigma-70 factor, ECF subfamily
CP049783.1_2360	K18672	81.73	354.8	2.3e-106	diadenylate cyclase [EC:2.7.7.85]
CP049783.1_2362	K03431	454.20	674.5	4.4e-203	phosphoglucosamine mutase [EC:5.4.2.10]
CP049783.1_2363	K00820	183.57	813.3	7.4e-245	glutamine---fructose-6-phosphate transaminase (isomerizing) [EC:2.6.1.16]
CP049783.1_2387	K03484	339.57	397.8	1.5e-119	LacI family transcriptional regulator, sucrose operon repressor
CP049783.1_2387	K02529	268.37	310.7	6.4e-93	LacI family transcriptional regulator, galactose operon repressor
CP049783.1_2388	K25086	282.43	380.8	3e-114	maltose transport system substrate-binding protein
CP049783.1_2389	K25087	353.50	399.5	2.5e-120	maltose transport system permease protein
CP049783.1_2390	K25088	335.73	380.5	1.5e-114	maltose transport system permease protein
CP049783.1_2390	K10119	296.93	322.7	8e-97	raffinose/stachyose/melibiose transport system permease protein
CP049783.1_2391	K01193	187.77	505.0	9.3e-152	beta-fructofuranosidase [EC:3.2.1.26]

CP049783.1_2393	K06425	45.60	87.9	9.8e-26	small acid-soluble spore protein H (minor)
CP049783.1_2395	K11623	240.53	512.4	2.5e-154	two-component system, NarL family, sensor histidine kinase YdfH [EC:2.7.13.3]
CP049783.1_2396	K11624	235.50	332.3	3.1e-100	two-component system, NarL family, response regulator YdfI
CP049783.1_2397	K03585	261.90	262.5	2.7e-78	membrane fusion protein, multidrug efflux system
CP049783.1_2400	K01620	94.73	185.3	7.6e-55	threonine aldolase [EC:4.1.2.48]
CP049783.1_2401	K18954	149.17	165.6	6.8e-49	AraC family transcriptional regulator, transcriptional activator of pobA
CP049783.1_2402	K01051	75.37	390.6	4e-117	pectinesterase [EC:3.1.1.11]
CP049783.1_2404	K03291	164.97	404.2	2.3e-121	MFS transporter, SET family, sugar efflux transporter
CP049783.1_2407	K03892	89.73	101.1	2.5e-29	ArsR family transcriptional regulator, arsenate/arsenite/antimonite-responsive transcriptional repressor
CP049783.1_2408	K03893	199.80	567.3	9.3e-171	arsenical pump membrane protein
CP049783.1_2409	K03741	124.07	170.3	2e-50	arsenate reductase (thioredoxin) [EC:1.20.4.4]
CP049783.1_2412	K15974	111.57	116.0	6.1e-34	MarR family transcriptional regulator, negative regulator of the multidrug operon emrRAB
CP049783.1_2412	K15973	110.17	113.5	3e-33	MarR family transcriptional regulator, 2-MHQ and catechol-resistance regulon repressor
CP049783.1_2413	K01872	199.53	231.2	8e-69	alanyl-tRNA synthetase [EC:6.1.1.7]
CP049783.1_2420	K11752	189.40	508.0	1.8e-152	diaminohydroxyphosphoribosylaminopyrimidine deaminase / 5-amino-6-(5-phosphoribosylamino)uracil reductase [EC:3.5.4.26 1.1.1.193]
CP049783.1_2421	K01180	175.60	483.3	4.8e-145	endo-1,3(4)-beta-glucanase [EC:3.2.1.6]
CP049783.1_2426	K01619	39.37	338.9	1.3e-101	deoxyribose-phosphate aldolase [EC:4.1.2.4]
CP049783.1_2427	K01489	31.20	214.9	4.9e-64	cytidine deaminase [EC:3.5.4.5]
CP049783.1_2428	K11535	487.87	635.8	7.7e-192	nucleoside transport protein
CP049783.1_2429	K01839	156.37	636.6	1.1e-191	phosphopentomutase [EC:5.4.2.7]
CP049783.1_2430	K03784	226.90	405.7	3e-122	purine-nucleoside phosphorylase [EC:2.4.2.1]
CP049783.1_2431	K00756	568.60	710.2	3.8e-214	pyrimidine-nucleoside phosphorylase [EC:2.4.2.2]
CP049783.1_2435	K23163	494.43	583.4	7.1e-176	sulfate/thiosulfate transport system substrate-binding protein
CP049783.1_2436	K02046	286.67	465.0	4.1e-140	sulfate/thiosulfate transport system permease protein
CP049783.1_2437	K02047	295.30	491.5	4.2e-148	sulfate/thiosulfate transport system permease protein
CP049783.1_2438	K01556	182.23	190.2	1.5e-56	kynureninase [EC:3.7.1.3]
CP049783.1_2439	K02042	106.83	318.2	3.3e-95	phosphonate transport system permease protein
CP049783.1_2440	K02042	106.83	338.2	2.7e-101	phosphonate transport system permease protein
CP049783.1_2441	K02041	268.77	395.6	5.2e-119	phosphonate transport system ATP-binding protein [EC:7.3.2.2]
CP049783.1_2442	K02044	121.03	308.0	4.1e-92	phosphonate transport system substrate-binding protein
CP049783.1_2443	K01119	358.37	814.7	2.3e-245	2',3'-cyclic-nucleotide 2'-phosphodiesterase / 3'-nucleotidase [EC:3.1.4.16 3.1.3.6]
CP049783.1_2447	K08602	113.03	194.5	1e-57	oligoendopeptidase F [EC:3.4.24.-]
CP049783.1_2448	K15975	357.47	433.2	3.5e-130	glyoxalase family protein
CP049783.1_2450	K02004	52.33	133.7	2e-39	putative ABC transport system permease protein
CP049783.1_2451	K02003	292.97	344.7	2.2e-103	putative ABC transport system ATP-binding protein
CP049783.1_2454	K25284	273.03	475.3	3.6e-143	iron-siderophore transport system permease protein
CP049783.1_2455	K25283	156.17	430.1	2.7e-129	iron-siderophore transport system permease protein
CP049783.1_2456	K25285	297.63	413.4	1.4e-124	iron-siderophore transport system ATP-binding protein [EC:7.2.2.-]
CP049783.1_2456	K02013	238.30	282.2	2.4e-84	iron complex transport system ATP-binding protein [EC:7.2.2.-]
CP049783.1_2457	K25282	200.83	444.3	7.4e-134	iron-siderophore transport system substrate-binding protein
CP049783.1_2461	K06153	46.03	315.2	2.2e-94	undecaprenyl-diphosphatase [EC:3.6.1.27]
CP049783.1_2462	K03699	305.47	507.5	1.4e-152	magnesium and cobalt exporter, CNNM family
CP049783.1_2467	K19431	359.93	526.5	6.6e-159	pyruvyl transferase EpsO [EC:2.-.-.-]
CP049783.1_2468	K01462	69.73	137.0	2.8e-40	peptide deformylase [EC:3.5.1.88]
CP049783.1_2469	K07718	273.17	451.9	1.6e-135	two-component system, sensor histidine kinase YesM [EC:2.7.13.3]
CP049783.1_2470	K07720	156.87	373.4	7.7e-112	two-component system, response regulator YesN
CP049783.1_2472	K00425	142.17	616.6	1.7e-185	cytochrome bd ubiquinol oxidase subunit I [EC:7.1.1.7]
CP049783.1_2473	K00426	28.43	407.0	3.2e-122	cytochrome bd ubiquinol oxidase subunit II [EC:7.1.1.7]
CP049783.1_2474	K16013	477.03	691.8	3.7e-208	ATP-binding cassette, subfamily C, bacterial CydD
CP049783.1_2475	K16012	468.53	589.0	3.6e-177	ATP-binding cassette, subfamily C, bacterial CydC
CP049783.1_2478	K17319	220.03	443.9	1.5e-133	putative aldouronate transport system permease protein
CP049783.1_2479	K17320	270.87	358.2	9.8e-108	putative aldouronate transport system permease protein
CP049783.1_2480	K17318	176.50	317.8	5.6e-95	putative aldouronate transport system substrate-binding protein
CP049783.1_2481	K15923	183.83	215.6	3.6e-64	alpha-L-fucosidase 2 [EC:3.2.1.51]
CP049783.1_2482	K15923	183.83	860.1	7.4e-259	alpha-L-fucosidase 2 [EC:3.2.1.51]
CP049783.1_2483	K05350	554.93	636.0	3.2e-191	beta-glucosidase [EC:3.2.1.21]

CP049783.1_2485	K02297	326.83	387.1	2.5e-116	cytochrome o ubiquinol oxidase subunit II [EC:7.1.1.3]
CP049783.1_2486	K02827	976.50	1098.0	0	cytochrome aa3-600 menaquinol oxidase subunit I [EC:7.1.1.5]
CP049783.1_2487	K02299	266.80	291.3	2.8e-87	cytochrome o ubiquinol oxidase subunit III
CP049783.1_2488	K02300	81.93	126.3	2.3e-37	cytochrome o ubiquinol oxidase subunit IV
CP049783.1_2488	K02829	99.33	99.9	2.4e-29	cytochrome aa3-600 menaquinol oxidase subunit IV [EC:7.1.1.5]
CP049783.1_2490	K21903	126.23	150.3	1.8e-44	ArsR family transcriptional regulator, lead/cadmium/zinc/bismuth-responsive transcriptional repressor
CP049783.1_2491	K01534	713.30	896.8	3.8e-270	Zn2+/Cd2+-exporting ATPase [EC:7.2.2.12 7.2.2.21]
CP049783.1_2493	K01534	713.30	728.0	3.9e-219	Zn2+/Cd2+-exporting ATPase [EC:7.2.2.12 7.2.2.21]
CP049783.1_2494	K03446	476.67	611.7	4e-184	MFS transporter, DHA2 family, multidrug resistance protein
CP049783.1_2496	K03809	60.03	177.0	1.7e-52	NAD(P)H dehydrogenase (quinone) [EC:1.6.5.2]
CP049783.1_2498	K07238	128.17	202.5	3.6e-60	zinc transporter, ZIP family
CP049783.1_2499	K00384	332.10	492.9	2.8e-148	thioredoxin reductase (NADPH) [EC:1.8.1.9]
CP049783.1_2501	K25026	274.23	275.7	2.2e-82	glucokinase [EC:2.7.1.2]
CP049783.1_2502	K01805	102.00	687.1	7e-207	xylose isomerase [EC:5.3.1.5]
CP049783.1_2503	K00854	306.67	395.3	1.8e-118	xylulokinase [EC:2.7.1.17]
CP049783.1_2504	K04759	94.50	512.4	6e-154	ferrous iron transport protein B
CP049783.1_2505	K04759	94.50	238.8	3.3e-71	ferrous iron transport protein B
CP049783.1_2506	K04758	39.27	50.4	7.8e-14	ferrous iron transport protein A
CP049783.1_2509	K11733	633.90	837.8	7.5e-253	lysine-specific permease
CP049783.1_2509	K16261	613.47	620.8	8.8e-187	yeast amino acid transporter
CP049783.1_2511	K08224	171.37	262.6	1.6e-78	MFS transporter, YNFM family, putative membrane transport protein
CP049783.1_2512	K15974	111.57	159.8	2.5e-47	MarR family transcriptional regulator, negative regulator of the multidrug operon emrRAB
CP049783.1_2514	K00425	142.17	454.6	1.9e-136	cytochrome bd ubiquinol oxidase subunit I [EC:7.1.1.7]
CP049783.1_2515	K00426	28.43	125.8	6.5e-37	cytochrome bd ubiquinol oxidase subunit II [EC:7.1.1.7]
CP049783.1_2516	K13985	99.67	300.4	8.1e-90	N-acyl-phosphatidylethanolamine-hydrolysing phospholipase D [EC:3.1.4.54]
CP049783.1_2517	K07145	77.87	135.2	3.5e-40	heme oxygenase (staphylobilin-producing) [EC:1.14.99.48]
CP049783.1_2518	K02013	238.30	324.5	3.4e-97	iron complex transport system ATP-binding protein [EC:7.2.2.-]
CP049783.1_2519	K02015	340.73	343.2	8.6e-103	iron complex transport system permease protein
CP049783.1_2520	K02016	154.03	182.4	4.8e-54	iron complex transport system substrate-binding protein
CP049783.1_2521	K25118	112.03	203.6	1e-60	iron-regulated surface determinant protein C
CP049783.1_2522	K25118	112.03	139.7	3.4e-41	iron-regulated surface determinant protein C
CP049783.1_2523	K25118	112.03	140.0	2.8e-41	iron-regulated surface determinant protein C
CP049783.1_2524	K25118	112.03	122.1	8.6e-36	iron-regulated surface determinant protein C
CP049783.1_2525	K25113	235.23	426.8	1.1e-128	heme transport system substrate-binding protein
CP049783.1_2526	K25114	375.03	462.4	2.4e-139	heme transport system permease protein
CP049783.1_2527	K25115	347.47	386.6	1.3e-116	heme transport system ATP-binding protein [EC:7.6.2.5]
CP049783.1_2527	K02013	238.30	325.7	1.5e-97	iron complex transport system ATP-binding protein [EC:7.2.2.-]
CP049783.1_2530	K13283	187.13	201.7	4.3e-60	ferrous-iron efflux pump FieF
CP049783.1_2536	K07315	145.67	198.6	5.5e-59	phosphoserine phosphatase RsbU/P [EC:3.1.3.3]
CP049783.1_2538	K00575	241.67	270.3	1.3e-80	chemotaxis protein methyltransferase CheR [EC:2.1.1.80]
CP049783.1_2539	K11527	622.20	639.4	2.8e-192	two-component system, sensor histidine kinase and response regulator [EC:2.7.13.3]
CP049783.1_2539	K20974	520.23	561.4	7.4e-169	two-component system, sensor histidine kinase [EC:2.7.13.3]
CP049783.1_2540	K04749	63.93	72.3	1.7e-20	anti-sigma B factor antagonist
CP049783.1_2541	K04757	53.50	118.8	9.2e-35	serine/threonine-protein kinase RsbW [EC:2.7.11.1]
CP049783.1_2547	K19302	106.60	126.4	4.9e-37	undecaprenyl-diphosphatase [EC:3.6.1.27]
CP049783.1_2548	K06911	36.97	159.6	4.2e-47	quercetin 2,3-dioxygenase [EC:1.13.11.24]
CP049783.1_2553	K07006	34.40	46.6	9.8e-13	uncharacterized protein
CP049783.1_2558	K03975	59.97	110.0	3.7e-32	membrane-associated protein
CP049783.1_2563	K02435	35.93	106.3	4.7e-31	aspartyl-tRNA(Asn)/glutamyl-tRNA(Gln) amidotransferase subunit C [EC:6.3.5.6 6.3.5.7]
CP049783.1_2564	K02433	450.63	715.9	1.9e-215	aspartyl-tRNA(Asn)/glutamyl-tRNA(Gln) amidotransferase subunit A [EC:6.3.5.6 6.3.5.7]
CP049783.1_2564	K01426	303.90	407.8	3.7e-122	amidase [EC:3.5.1.4]
CP049783.1_2565	K02434	415.47	741.6	2.1e-223	aspartyl-tRNA(Asn)/glutamyl-tRNA(Gln) amidotransferase subunit B [EC:6.3.5.6 6.3.5.7]
CP049783.1_2567	K07507	42.27	227.6	7.4e-68	putative Mg2+ transporter-C (MgtC) family protein
CP049783.1_2569	K11621	70.10	109.5	5.2e-32	lia operon protein LiaG
CP049783.1_2570	K09825	159.17	208.5	2.3e-62	Fur family transcriptional regulator, peroxide stress response regulator
CP049783.1_2570	K03711	142.10	152.9	4.3e-45	Fur family transcriptional regulator, ferric uptake regulator

CP049783.1_2577	K09013	161.43	418.3	4.6e-126	Fe-S cluster assembly ATP-binding protein
CP049783.1_2578	K09015	272.00	304.1	6.8e-91	Fe-S cluster assembly protein SufD
CP049783.1_2579	K09014	377.87	816.5	4.1e-246	Fe-S cluster assembly protein SufB
CP049783.1_2584	K08153	415.17	547.4	4.4e-165	MFS transporter, DHA1 family, multidrug resistance protein
CP049783.1_2586	K03969	84.50	122.9	6.1e-36	phage shock protein A
CP049783.1_2588	K03969	84.50	174.3	1.5e-51	phage shock protein A
CP049783.1_2589	K11622	83.40	160.2	2e-47	lia operon protein LiaF
CP049783.1_2590	K11617	255.57	398.1	1.3e-119	two-component system, NarL family, sensor histidine kinase LiaS [EC:2.7.13.3]
CP049783.1_2591	K11618	268.20	282.3	1.4e-84	two-component system, NarL family, response regulator LiaR
CP049783.1_2594	K00036	396.33	654.9	5e-197	glucose-6-phosphate 1-dehydrogenase [EC:1.1.1.49 1.1.1.363]
CP049783.1_2595	K02837	383.73	832.6	4.5e-251	peptide chain release factor 3
CP049783.1_2596	K06426	67.10	87.4	1.7e-25	small acid-soluble spore protein I (minor)
CP049783.1_2597	K03499	127.40	204.9	5.5e-61	trk/ktr system potassium uptake protein
CP049783.1_2598	K03437	214.63	296.0	1.1e-88	RNA methyltransferase, TrmH family
CP049783.1_2599	K03088	96.50	117.7	1.8e-34	RNA polymerase sigma-70 factor, ECF subfamily
CP049783.1_2602	K11634	298.93	355.3	4.4e-107	two-component system, OmpR family, response regulator YxdJ
CP049783.1_2603	K11633	303.53	437.5	1.1e-131	two-component system, OmpR family, sensor histidine kinase YxdK [EC:2.7.13.3]
CP049783.1_2607	K02005	227.43	291.9	3.2e-87	HlyD family secretion protein
CP049783.1_2608	K02003	292.97	340.2	5.3e-102	putative ABC transport system ATP-binding protein
CP049783.1_2609	K02004	52.33	111.2	1.4e-32	putative ABC transport system permease protein
CP049783.1_2611	K22278	73.27	173.6	2.2e-51	peptidoglycan-N-acetylglucosamine deacetylase [EC:3.5.1.104]
CP049783.1_2612	K03088	96.50	116.6	3.9e-34	RNA polymerase sigma-70 factor, ECF subfamily
CP049783.1_2614	K02761	189.70	423.2	4.3e-127	cellobiose PTS system EIIC component
CP049783.1_2615	K02760	71.37	146.1	3.1e-43	cellobiose PTS system EIIB component [EC:2.7.1.196 2.7.1.205]
CP049783.1_2616	K02759	33.93	138.6	4.9e-41	cellobiose PTS system EIIA component [EC:2.7.1.196 2.7.1.205]
CP049783.1_2617	K03491	482.23	673.8	8.6e-203	probable licABCH operon transcriptional regulator
CP049783.1_2618	K00864	510.13	650.9	6e-196	glycerol kinase [EC:2.7.1.30]
CP049783.1_2620	K00615	214.20	271.1	7.3e-81	transketolase [EC:2.2.1.1]
CP049783.1_2622	K01223	479.40	786.0	5.6e-237	6-phospho-beta-glucosidase [EC:3.2.1.86]
CP049783.1_2623	K03488	318.67	385.0	9.7e-116	beta-glucoside operon transcriptional antiterminator
CP049783.1_2624	K02757	642.13	821.5	1.7e-247	beta-glucoside PTS system EIICBA component [EC:2.7.1.-]
CP049783.1_2625	K03310	262.87	570.0	1.7e-171	alanine or glycine:cation symporter, AGCS family
CP049783.1_2626	K13530	199.90	282.0	1.3e-84	AraC family transcriptional regulator, regulatory protein of adaptative response / methylphosphotriester-DNA alkyltransferase methyltransferase [EC:2.1.1.-]
CP049783.1_2627	K13531	197.13	258.3	8.9e-78	methylated-DNA-[protein]-cysteine S-methyltransferase [EC:2.1.1.63]
CP049783.1_2627	K00567	174.97	175.8	4.9e-52	methylated-DNA-[protein]-cysteine S-methyltransferase [EC:2.1.1.63]
CP049783.1_2629	K01247	173.13	194.4	1e-57	DNA-3-methyladenine glycosylase II [EC:3.2.2.21]
CP049783.1_2631	K07720	156.87	323.1	1.4e-96	two-component system, response regulator YesN
CP049783.1_2632	K07718	273.17	449.9	6.5e-135	two-component system, sensor histidine kinase YesM [EC:2.7.13.3]
CP049783.1_2633	K02027	193.17	230.6	1.4e-68	multiple sugar transport system substrate-binding protein
CP049783.1_2634	K02025	276.90	343.0	5.7e-103	multiple sugar transport system permease protein
CP049783.1_2635	K02026	280.30	331.3	2.2e-99	multiple sugar transport system permease protein
CP049783.1_2636	K06607	318.77	472.4	1.6e-142	myo-inositol catabolism protein IolS [EC:1.1.1.-]
CP049783.1_2639	K03644	109.93	503.1	2.8e-151	lipoyl synthase [EC:2.8.1.8]
CP049783.1_2641	K00858	38.63	117.5	2.1e-34	NAD+ kinase [EC:2.7.1.23]
CP049783.1_2644	K06886	39.40	127.3	2.8e-37	hemoglobin
CP049783.1_2645	K09766	83.00	275.7	1.9e-82	uncharacterized protein
CP049783.1_2648	K08602	113.03	424.2	3.5e-127	oligoendopeptidase F [EC:3.4.24.-]
CP049783.1_2651	K01785	42.83	140.2	3.4e-41	aldose 1-epimerase [EC:5.1.3.3]
CP049783.1_2652	K01867	130.77	437.2	3.3e-131	tryptophanyl-tRNA synthetase [EC:6.1.1.2]
CP049783.1_2656	K07038	27.13	92.6	7.3e-27	inner membrane protein
CP049783.1_2657	K02257	126.90	304.4	4.5e-91	heme o synthase [EC:2.5.1.141]
CP049783.1_2658	K07476	82.00	139.9	1e-41	toprim domain protein
CP049783.1_2664	K21064	166.97	242.3	2.3e-72	5-amino-6-(5-phospho-D-ribitylamino)uracil phosphatase [EC:3.1.3.104]
CP049783.1_2665	K09793	59.33	153.3	2e-45	uncharacterized protein
CP049783.1_2666	K06409	388.70	468.9	6.3e-141	stage V sporulation protein B
CP049783.1_2669	K02259	62.60	126.5	3.1e-37	heme a synthase [EC:1.17.99.9]

CP049783.1_2670	K16239	730.73	1023.5	6.8e-309	4-hydroxybenzoate decarboxylase subunit C [EC:4.1.1.61]
CP049783.1_2670	K03182	505.87	507.6	1.2e-152	4-hydroxy-3-polyprenylbenzoate decarboxylase [EC:4.1.1.98]
CP049783.1_2673	K02071	332.87	503.8	1.5e-151	D-methionine transport system ATP-binding protein
CP049783.1_2673	K02065	294.10	295.5	2.6e-88	phospholipid/cholesterol/gamma-HCH transport system ATP-binding protein
CP049783.1_2674	K02072	119.67	325.0	1.2e-97	D-methionine transport system permease protein
CP049783.1_2675	K02073	57.57	404.4	2.4e-121	D-methionine transport system substrate-binding protein
CP049783.1_2677	K22074	86.17	102.2	1e-29	NFU1 iron-sulfur cluster scaffold homolog, mitochondrial
CP049783.1_2679	K03885	203.30	280.2	1.3e-83	NADH:quinone reductase (non-electrogenic) [EC:1.6.5.9]
CP049783.1_2680	K18285	441.40	515.2	2.2e-155	aminodeoxyfutalosine synthase [EC:2.5.1.120]
CP049783.1_2681	K13628	138.77	147.2	1.5e-43	iron-sulfur cluster assembly protein
CP049783.1_2685	K15269	141.60	154.2	1.2e-45	probable blue pigment (indigoidine) exporter
CP049783.1_2690	K11785	251.53	434.2	6.4e-131	5,8-dihydroxy-2-naphthoate synthase [EC:4.1.99.29]
CP049783.1_2691	K11783	110.30	295.1	2.4e-88	futalosine hydrolase [EC:3.2.2.26]
CP049783.1_2693	K00937	243.67	982.5	6.3e-296	polyphosphate kinase [EC:2.7.4.1]
CP049783.1_2694	K01524	159.03	307.2	6.5e-92	exopolyphosphatase / guanosine-5'-triphosphate,3'-diphosphate pyrophosphatase [EC:3.6.1.11 3.6.1.40]
CP049783.1_2696	K06371	34.23	55.4	2.1e-15	developmental checkpoint coupling sporulation initiation to replication initiation
CP049783.1_2697	K21567	376.93	515.7	1.6e-155	ferredoxin/flavodoxin---NADP+ reductase [EC:1.18.1.2 1.19.1.1]
CP049783.1_2698	K03885	203.30	389.8	9.2e-117	NADH:quinone reductase (non-electrogenic) [EC:1.6.5.9]
CP049783.1_2699	K00435	268.83	292.8	6.1e-88	hydrogen peroxide-dependent heme synthase [EC:1.3.98.5]
CP049783.1_2700	K03346	83.40	255.5	3.6e-76	replication initiation and membrane attachment protein
CP049783.1_2701	K11144	162.27	322.3	1.4e-96	primosomal protein DnaI
CP049783.1_2702	K03671	112.43	153.3	3.3e-45	thioredoxin
CP049783.1_2703	K03703	236.70	743.3	9.2e-224	excinuclease ABC subunit C
CP049783.1_2704	K07052	31.30	54.5	2.8e-15	CAAX protease family protein
CP049783.1_2705	K03498	117.37	478.4	9e-144	trk/ktr system potassium uptake protein
CP049783.1_2706	K03499	127.40	201.1	7.9e-60	trk/ktr system potassium uptake protein
CP049783.1_2709	K00241	50.80	130.3	3e-38	succinate dehydrogenase cytochrome b subunit
CP049783.1_2710	K00239	665.27	724.0	4.4e-218	succinate dehydrogenase flavoprotein subunit [EC:1.3.5.1]
CP049783.1_2711	K00240	157.40	252.0	2.1e-75	succinate dehydrogenase iron-sulfur subunit [EC:1.3.5.1]
CP049783.1_2712	K00662	43.03	301.7	2.8e-90	aminoglycoside 3-N-acetyltransferase [EC:2.3.1.81]
CP049783.1_2713	K07098	96.03	298.2	3e-89	uncharacterized protein
CP049783.1_2719	K07816	97.83	313.5	6.5e-94	GTP pyrophosphokinase [EC:2.7.6.5]
CP049783.1_2720	K04486	126.10	258.0	4.8e-77	histidinol-phosphatase (PHP family) [EC:3.1.3.15]
CP049783.1_2722	K03409	53.67	120.0	4.9e-35	chemotaxis protein CheX
CP049783.1_2723	K07124	220.10	245.3	3e-73	uncharacterized protein
CP049783.1_2725	K25158	298.00	334.9	6.8e-101	ABC-2 type transport system ATP-binding protein
CP049783.1_2726	K25157	161.40	376.7	3.7e-113	ABC-2 type transport system permease protein
CP049783.1_2728	K07697	304.97	428.6	1.1e-128	two-component system, sporulation sensor kinase B [EC:2.7.13.3]
CP049783.1_2730	K02099	174.60	180.6	1.7e-53	AraC family transcriptional regulator, arabinose operon regulatory protein
CP049783.1_2731	K01613	33.77	244.3	7.8e-73	phosphatidylserine decarboxylase [EC:4.1.1.65]
CP049783.1_2733	K06338	256.40	421.9	9.2e-127	spore coat protein SA
CP049783.1_2737	K01400	510.83	671.7	5.6e-202	bacillolysin [EC:3.4.24.28]
CP049783.1_2740	K16870	147.83	169.1	5.5e-50	N-acetylglucosaminyl-diphospho-decaprenol L-rhamnosyltransferase [EC:2.4.1.289]
CP049783.1_2741	K00973	234.80	280.2	1.2e-83	glucose-1-phosphate thymidylyltransferase [EC:2.7.7.24]
CP049783.1_2748	K06320	85.60	307.2	5.1e-92	spore maturation protein CgeB
CP049783.1_2749	K06320	85.60	301.1	3.6e-90	spore maturation protein CgeB
CP049783.1_2752	K15894	314.93	407.6	1e-122	UDP-N-acetylglucosamine 4,6-dehydratase/5-epimerase [EC:4.2.1.115 5.1.3.-]
CP049783.1_2766	K01784	253.97	256.1	2.6e-76	UDP-glucose 4-epimerase [EC:5.1.3.2]
CP049783.1_2767	K01223	479.40	505.8	3.9e-152	6-phospho-beta-glucosidase [EC:3.2.1.86]
CP049783.1_2774	K02757	642.13	671.7	3.4e-202	beta-glucoside PTS system EIICBA component [EC:2.7.1.-]
CP049783.1_2779	K25152	333.13	454.4	2.9e-136	gliding motility-associated transport system ATP-binding protein
CP049783.1_2779	K01990	262.37	295.1	3e-88	ABC-2 type transport system ATP-binding protein
CP049783.1_2780	K25151	181.30	234.0	9.8e-70	gliding motility-associated transport system permease protein
CP049783.1_2781	K25153	161.87	335.2	2.7e-100	gliding motility-associatede transport system auxiliary component
CP049783.1_2786	K01046	52.07	87.7	3.1e-25	triacylglycerol lipase [EC:3.1.1.3]
CP049783.1_2788	K01443	131.87	466.1	6.2e-140	N-acetylglucosamine-6-phosphate deacetylase [EC:3.5.1.25]

CP049783.1_2789	K02564	97.07	339.4	8.3e-102	glucosamine-6-phosphate deaminase [EC:3.5.99.6]
CP049783.1_2791	K02078	39.80	45.6	2.5e-12	acyl carrier protein
CP049783.1_2794	K02078	39.80	44.3	6.1e-12	acyl carrier protein
CP049783.1_2796	K25156	291.53	453.1	5.4e-136	viologen exporter family transport system ATP-binding protein
CP049783.1_2797	K25155	105.97	187.7	1.2e-55	viologen exporter family transport system permease protein
CP049783.1_2798	K25154	114.47	207.6	8.8e-62	viologen exporter family transport system permease protein
CP049783.1_2801	K13327	343.67	374.9	6.8e-113	dTDP-3,4-didehydro-2,6-dideoxy-alpha-D-glucose 3-reductase [EC:1.1.1.384]
CP049783.1_2802	K01586	276.10	395.0	2.4e-118	diaminopimelate decarboxylase [EC:4.1.1.20]
CP049783.1_2803	K07243	31.57	272.3	1.9e-81	high-affinity iron transporter
CP049783.1_2804	K16301	470.73	580.4	7.8e-175	deferrochelatase/peroxidase EfeB [EC:1.11.1.-]
CP049783.1_2805	K07224	230.17	346.2	9.3e-104	iron uptake system component EfeO
CP049783.1_2806	K01994	89.63	132.4	1.3e-39	LuxR family transcriptional regulator, transcriptional regulator of spore coat protein
CP049783.1_2810	K00563	111.90	263.5	7.1e-79	23S rRNA (guanine745-N1)-methyltransferase [EC:2.1.1.187]
CP049783.1_2813	K03406	65.50	296.1	1.9e-88	methyl-accepting chemotaxis protein
CP049783.1_2818	K15894	314.93	406.3	2.7e-122	UDP-N-acetylglucosamine 4,6-dehydratase/5-epimerase [EC:4.2.1.115 5.1.3.-]
CP049783.1_2819	K13015	437.70	633.9	6.8e-191	UDP-N-acetyl-D-glucosamine dehydrogenase [EC:1.1.1.136]
CP049783.1_2825	K07118	124.57	168.1	8.5e-50	uncharacterized protein
CP049783.1_2827	K07124	220.10	322.2	1.4e-96	uncharacterized protein
CP049783.1_2828	K22477	98.13	191.9	1.6e-57	N-acetylglutamate synthase [EC:2.3.1.1]
CP049783.1_2830	K06298	94.10	301.7	2.7e-90	germination protein M
CP049783.1_2831	K00989	189.03	434.9	9.6e-131	ribonuclease PH [EC:2.7.7.56]
CP049783.1_2832	K01519	53.90	223.2	2.1e-66	XTP/dITP diphosphohydrolase [EC:3.6.1.66]
CP049783.1_2833	K01953	106.30	515.3	1e-154	asparagine synthase (glutamine-hydrolysing) [EC:6.3.5.4]
CP049783.1_2835	K09940	37.80	37.8	3.7e-10	uncharacterized protein
CP049783.1_2836	K01222	394.63	615.1	2.5e-185	6-phospho-beta-glucosidase [EC:3.2.1.86]
CP049783.1_2841	K00788	164.63	240.9	6.2e-72	thiamine-phosphate pyrophosphorylase [EC:2.5.1.3]
CP049783.1_2842	K00941	324.10	407.6	3.3e-122	hydroxymethylpyrimidine/phosphomethylpyrimidine kinase [EC:2.7.1.49 2.7.4.7]
CP049783.1_2843	K00878	112.23	341.9	1.6e-102	hydroxyethylthiazole kinase [EC:2.7.1.50]
CP049783.1_2844	K03707	196.20	269.7	1e-80	thiaminase (transcriptional activator TenA) [EC:3.5.99.2]
CP049783.1_2845	K00029	287.77	610.1	1.5e-183	malate dehydrogenase (oxaloacetate-decarboxylating)(NADP+) [EC:1.1.1.40]
CP049783.1_2845	K00027	507.30	539.7	4.3e-162	malate dehydrogenase (oxaloacetate-decarboxylating) [EC:1.1.1.38]
CP049783.1_2855	K00971	203.67	286.0	2.1e-85	mannose-1-phosphate guanylyltransferase [EC:2.7.7.13]
CP049783.1_2857	K02757	642.13	821.6	1.6e-247	beta-glucoside PTS system EIICBA component [EC:2.7.1.-]
CP049783.1_2858	K03488	318.67	382.8	4.7e-115	beta-glucoside operon transcriptional antiterminator
CP049783.1_2860	K01223	479.40	798.6	8.6e-241	6-phospho-beta-glucosidase [EC:3.2.1.86]
CP049783.1_2861	K03310	262.87	584.2	7.9e-176	alanine or glycine:cation symporter, AGCS family
CP049783.1_2863	K02477	121.47	140.8	1.8e-41	two-component system, LytTR family, response regulator
CP049783.1_2864	K06871	174.27	272.5	2.5e-81	uncharacterized protein
CP049783.1_2867	K06871	174.27	224.0	1.3e-66	uncharacterized protein
CP049783.1_2868	K10242	321.97	398.8	3e-120	cellobiose transport system permease protein
CP049783.1_2868	K02026	280.30	313.8	4.4e-94	multiple sugar transport system permease protein
CP049783.1_2869	K10241	361.87	416.0	4.2e-125	cellobiose transport system permease protein
CP049783.1_2869	K02025	276.90	302.6	1e-90	multiple sugar transport system permease protein
CP049783.1_2870	K10240	310.77	471.2	9.8e-142	cellobiose transport system substrate-binding protein
CP049783.1_2871	K02804	644.17	665.6	1.9e-200	N-acetylglucosamine PTS system EIICBA or EIICB component [EC:2.7.1.193]
CP049783.1_2872	K02440	275.10	375.8	5.9e-113	glycerol uptake facilitator
CP049783.1_2874	K00864	510.13	754.7	2.4e-227	glycerol kinase [EC:2.7.1.30]
CP049783.1_2875	K02529	268.37	279.3	2.1e-83	LacI family transcriptional regulator, galactose operon repressor
CP049783.1_2876	K10117	246.87	307.2	1.1e-91	raffinose/stachyose/melibiose transport system substrate-binding protein
CP049783.1_2879	K05350	554.93	609.5	3.4e-183	beta-glucosidase [EC:3.2.1.21]
CP049783.1_2881	K01142	228.27	362.3	1.5e-108	exodeoxyribonuclease III [EC:3.1.11.2]
CP049783.1_2883	K03545	69.90	487.8	1.5e-146	trigger factor
CP049783.1_2884	K01358	76.73	367.8	1.5e-110	ATP-dependent Clp protease, protease subunit [EC:3.4.21.92]
CP049783.1_2885	K03544	145.17	652.0	2.9e-196	ATP-dependent Clp protease ATP-binding subunit ClpX
CP049783.1_2886	K03526	92.70	553.0	2.3e-166	(E)-4-hydroxy-3-methylbut-2-enyl-diphosphate synthase [EC:1.17.7.1 1.17.7.3]
CP049783.1_2887	K04076	407.83	525.0	1e-157	ATP-dependent Lon protease [EC:3.4.21.53]

CP049783.1_2888	K01338	863.10	1116.9	0	ATP-dependent Lon protease [EC:3.4.21.53]
CP049783.1_2889	K03978	95.90	277.4	7.2e-83	GTP-binding protein
CP049783.1_2891	K01611	16.97	50.7	4.7e-14	S-adenosylmethionine decarboxylase [EC:4.1.1.50]
CP049783.1_2892	K02492	71.10	535.3	5.9e-161	glutamyl-tRNA reductase [EC:1.2.1.70]
CP049783.1_2893	K02497	138.90	275.7	1e-82	HemX protein
CP049783.1_2894	K24866	158.93	240.7	7.5e-72	precorrin-2 dehydrogenase [EC:1.3.1.76]
CP049783.1_2895	K01749	105.33	459.3	4.3e-138	hydroxymethylbilane synthase [EC:2.5.1.61]
CP049783.1_2896	K13542	404.20	678.6	4.1e-204	uroporphyrinogen III methyltransferase / synthase [EC:2.1.1.107 4.2.1.75]
CP049783.1_2896	K02303	255.53	370.1	4.4e-111	uroporphyrin-III C-methyltransferase [EC:2.1.1.107]
CP049783.1_2896	K01719	43.23	183.9	1.6e-54	uroporphyrinogen-III synthase [EC:4.2.1.75]
CP049783.1_2897	K01698	94.97	553.6	1.5e-166	porphobilinogen synthase [EC:4.2.1.24]
CP049783.1_2898	K01845	312.23	689.1	2.2e-207	glutamate-1-semialdehyde 2,1-aminomutase [EC:5.4.3.8]
CP049783.1_2900	K06417	121.47	287.6	3.9e-86	stage VI sporulation protein D
CP049783.1_2901	K01873	582.43	1351.8	0	valyl-tRNA synthetase [EC:6.1.1.9]
CP049783.1_2902	K11754	379.57	506.7	5e-152	dihydrofolate synthase / folylpolyglutamate synthase [EC:6.3.2.12 6.3.2.17]
CP049783.1_2903	K01924	374.10	601.3	7.6e-181	UDP-N-acetylmuramate--alanine ligase [EC:6.3.2.8]
CP049783.1_2904	K06380	120.37	276.0	1.8e-82	stage II sporulation protein B
CP049783.1_2906	K06287	207.20	277.1	6.4e-83	nucleoside triphosphate pyrophosphatase [EC:3.6.1.-]
CP049783.1_2907	K03630	40.07	328.9	1.7e-98	DNA repair protein RadC
CP049783.1_2908	K03569	108.73	560.0	9.1e-169	rod shape-determining protein MreB and related proteins
CP049783.1_2909	K03570	52.93	160.0	3.5e-47	rod shape-determining protein MreC
CP049783.1_2910	K03571	31.87	68.3	2.9e-19	rod shape-determining protein MreD
CP049783.1_2911	K03610	34.13	148.9	6.3e-44	septum site-determining protein MinC
CP049783.1_2912	K03609	203.03	326.5	1.1e-97	septum site-determining protein MinD
CP049783.1_2913	K06401	141.23	231.2	4.5e-69	stage IV sporulation protein FA
CP049783.1_2914	K06402	102.07	262.3	1.9e-78	stage IV sporulation protein FB [EC:3.4.24.-]
CP049783.1_2916	K02888	28.73	146.6	2.5e-43	large subunit ribosomal protein L21
CP049783.1_2917	K07584	34.67	122.8	4.1e-36	uncharacterized protein
CP049783.1_2918	K02899	36.30	150.9	1.3e-44	large subunit ribosomal protein L27
CP049783.1_2919	K06375	74.23	179.3	2.6e-53	stage 0 sporulation protein B (sporulation initiation phosphotransferase) [EC:2.7.-.-]
CP049783.1_2920	K03979	149.20	527.9	7.1e-159	GTPase [EC:3.6.5.-]
CP049783.1_2921	K06209	123.30	198.1	1.7e-59	chorismate mutase [EC:5.4.99.5]
CP049783.1_2922	K00003	285.10	546.2	4e-164	homoserine dehydrogenase [EC:1.1.1.3]
CP049783.1_2923	K00872	123.40	381.0	1.9e-114	homoserine kinase [EC:2.7.1.39]
CP049783.1_2925	K00826	167.23	212.5	3.1e-63	branched-chain amino acid aminotransferase [EC:2.6.1.42]
CP049783.1_2926	K06370	69.50	93.2	7.9e-27	morphogenetic protein associated with SpoVID
CP049783.1_2929	K06318	82.87	192.2	2e-57	forespore regulator of the sigma-K checkpoint
CP049783.1_2930	K01159	32.43	246.7	1.4e-73	crossover junction endodeoxyribonuclease RuvC [EC:3.1.21.10]
CP049783.1_2931	K03550	46.37	233.0	1.3e-69	holliday junction DNA helicase RuvA
CP049783.1_2932	K03551	186.47	609.8	1.1e-183	holliday junction DNA helicase RuvB [EC:5.6.2.4]
CP049783.1_2933	K06381	107.00	272.9	1.7e-81	stage II sporulation protein D (peptidoglycan lytic transglycosylase) [EC:4.2.2.29]
CP049783.1_2934	K07568	161.50	538.6	3.1e-162	S-adenosylmethionine:tRNA ribosyltransferase-isomerase [EC:2.4.99.17]
CP049783.1_2935	K00773	528.43	654.8	2.8e-197	queuine tRNA-ribosyltransferase [EC:2.4.2.29]
CP049783.1_2936	K03210	32.57	132.4	7.6e-39	preprotein translocase subunit YajC
CP049783.1_2939	K06409	388.70	608.7	3.1e-183	stage V sporulation protein B
CP049783.1_2944	K07462	66.97	758.7	2.4e-228	single-stranded-DNA-specific exonuclease [EC:3.1.-.-]
CP049783.1_2945	K00759	92.70	255.1	2.4e-76	adenine phosphoribosyltransferase [EC:2.4.2.7]
CP049783.1_2946	K03406	65.50	260.6	1.1e-77	methyl-accepting chemotaxis protein
CP049783.1_2947	K02824	450.17	547.2	9e-165	uracil permease
CP049783.1_2948	K01139	801.03	1024.2	1.1e-308	GTP diphosphokinase / guanosine-3',5'-bis(diphosphate) 3'-diphosphatase [EC:2.7.6.5 3.1.7.2]
CP049783.1_2949	K07560	40.67	211.7	4.6e-63	D-aminoacyl-tRNA deacylase [EC:3.1.1.96]
CP049783.1_2951	K05520	175.93	241.6	3.9e-72	deglycase [EC:3.5.1.124]
CP049783.1_2953	K01892	275.30	362.3	1.3e-108	histidyl-tRNA synthetase [EC:6.1.1.21]
CP049783.1_2954	K01876	301.23	912.7	1.1e-274	aspartyl-tRNA synthetase [EC:6.1.1.12]
CP049783.1_2955	K22132	163.63	314.2	5.6e-94	tRNA threonylcarbamoyladenosine dehydratase
CP049783.1_2956	K06901	93.90	391.5	1.8e-117	adenine/guanine/hypoxanthine permease

CP049783.1_2957	K18104	642.17	878.9	7.7e-265	ATP-binding cassette, subfamily B, bacterial AbcA/BmrA [EC:7.6.2.2]
CP049783.1_2959	K07478	187.97	594.1	1.4e-178	putative ATPase
CP049783.1_2960	K06199	39.60	110.8	1.8e-32	fluoride exporter
CP049783.1_2961	K06199	39.60	103.6	2.9e-30	fluoride exporter
CP049783.1_2965	K00566	424.50	563.5	1e-169	tRNA-uridine 2-sulfurtransferase [EC:2.8.1.13]
CP049783.1_2966	K17472	166.73	201.4	2.6e-60	Rrf2 family transcriptional regulator, cysteine metabolism repressor
CP049783.1_2967	K04487	287.40	563.1	1.8e-169	cysteine desulfurase [EC:2.8.1.7]
CP049783.1_2969	K03406	65.50	259.8	1.8e-77	methyl-accepting chemotaxis protein
CP049783.1_2971	K01872	199.53	1158.6	0	alanyl-tRNA synthetase [EC:6.1.1.7]
CP049783.1_2973	K07447	43.93	194.0	1.1e-57	putative pre-16S rRNA nuclease [EC:3.1.-.-]
CP049783.1_2976	K07082	42.70	355.5	1e-106	peptidoglycan lytic transglycosylase G [EC:4.2.2.29]
CP049783.1_2978	K08303	406.50	492.3	8e-148	U32 family peptidase [EC:3.4.-.-]
CP049783.1_2979	K03406	65.50	216.8	2e-64	methyl-accepting chemotaxis protein
CP049783.1_2981	K21468	498.27	641.3	4.4e-193	penicillin-binding protein 4B
CP049783.1_2983	K22278	73.27	214.7	7.7e-64	peptidoglycan-N-acetylglucosamine deacetylase [EC:3.5.1.104]
CP049783.1_2984	K01687	599.53	953.1	4.9e-287	dihydroxy-acid dehydratase [EC:4.2.1.9]
CP049783.1_2985	K06884	16.83	72.7	1.5e-20	uncharacterized protein
CP049783.1_2988	K01091	112.83	145.6	7.4e-43	phosphoglycolate phosphatase [EC:3.1.3.18]
CP049783.1_2989	K12410	226.47	294.6	2.8e-88	NAD-dependent protein deacetylase/lipoamidase [EC:2.3.1.286 2.3.1.313]
CP049783.1_2990	K19265	304.10	614.1	6.7e-185	L-glyceraldehyde 3-phosphate reductase [EC:1.1.1.-]
CP049783.1_2991	K02099	174.60	178.8	5.6e-53	AraC family transcriptional regulator, arabinose operon regulatory protein
CP049783.1_2992	K00849	368.57	469.5	7.2e-141	galactokinase [EC:2.7.1.6]
CP049783.1_2993	K01784	253.97	442.1	1e-132	UDP-glucose 4-epimerase [EC:5.1.3.2]
CP049783.1_2995	K19955	440.33	582.2	1.2e-175	alcohol dehydrogenase [EC:1.1.1.-]
CP049783.1_2997	K07192	64.60	484.8	1.6e-145	flotillin
CP049783.1_2998	K03480	295.03	379.5	2.7e-114	transcriptional antiterminator
CP049783.1_2999	K20118	807.70	966.3	2.3e-291	glucose PTS system EIICBA or EIICB component [EC:2.7.1.199]
CP049783.1_2999	K02791	716.30	758.8	1.3e-228	maltose/glucose PTS system EIICB component [EC:2.7.1.199 2.7.1.208]
CP049783.1_3000	K02784	81.30	88.0	2.2e-25	phosphocarrier protein HPr
CP049783.1_3001	K08483	678.63	847.0	5.2e-255	phosphoenolpyruvate-protein phosphotransferase (PTS system enzyme I) [EC:2.7.3.9]
CP049783.1_3002	K00283	537.67	883.8	2.4e-266	glycine cleavage system P protein (glycine dehydrogenase) subunit 2 [EC:1.4.4.2]
CP049783.1_3003	K00282	413.50	672.5	8.8e-203	glycine cleavage system P protein (glycine dehydrogenase) subunit 1 [EC:1.4.4.2]
CP049783.1_3004	K00605	295.73	493.6	2e-148	glycine cleavage system T protein (aminomethyltransferase) [EC:2.1.2.10]
CP049783.1_3005	K02437	35.70	179.0	2.8e-53	glycine cleavage system H protein
CP049783.1_3006	K02800	164.20	815.7	7.5e-246	mannitol PTS system EIICBA or EIICB component [EC:2.7.1.197]
CP049783.1_3007	K03483	467.10	628.9	4e-189	mannitol operon transcriptional activator
CP049783.1_3008	K02798	165.87	184.0	3.4e-55	mannitol PTS system EIIA component [EC:2.7.1.197]
CP049783.1_3009	K00009	152.20	512.0	8.1e-154	mannitol-1-phosphate 5-dehydrogenase [EC:1.1.1.17]
CP049783.1_3010	K09790	35.20	130.8	1.5e-38	uncharacterized protein
CP049783.1_3011	K06148	558.33	747.0	7.3e-225	ATP-binding cassette, subfamily C, bacterial
CP049783.1_3012	K01200	408.90	923.0	7e-278	pullulanase [EC:3.2.1.41]
CP049783.1_3013	K02897	35.70	185.2	5.1e-55	large subunit ribosomal protein L25
CP049783.1_3014	K03406	65.50	166.4	3.6e-49	methyl-accepting chemotaxis protein
CP049783.1_3015	K01297	137.13	254.4	5.1e-76	muramoyltetrapeptide carboxypeptidase [EC:3.4.17.13]
CP049783.1_3016	K27884	638.20	942.7	1.3e-284	two-component system, LytTR family, high-concentration pyruvate sensor kinase [EC:2.7.13.3]
CP049783.1_3017	K27885	310.23	395.7	1.8e-119	two-component system, LytTR family, high-concentration pyruvate response regulator
CP049783.1_3017	K02477	121.47	228.8	3.3e-68	two-component system, LytTR family, response regulator
CP049783.1_3018	K08177	233.33	452.8	4.1e-136	MFS transporter, OFA family, oxalate/formate antiporter
CP049783.1_3026	K20074	131.13	167.1	2.6e-49	PPM family protein phosphatase [EC:3.1.3.16]
CP049783.1_3036	K00005	325.77	509.7	1.2e-153	glycerol dehydrogenase [EC:1.1.1.6]
CP049783.1_3037	K05878	498.43	550.2	2e-165	phosphoenolpyruvate---glycerone phosphotransferase subunit DhaK [EC:2.7.1.121]
CP049783.1_3037	K00863	487.77	503.9	2.7e-151	triose/dihydroxyacetone kinase / FAD-AMP lyase (cyclizing) [EC:2.7.1.28 2.7.1.29 4.6.1.15]
CP049783.1_3041	K01060	117.80	381.8	1.3e-114	cephalosporin-C deacetylase [EC:3.1.1.41]
CP049783.1_3043	K08217	225.00	225.8	2.5e-67	MFS transporter, DHA3 family, macrolide efflux protein
CP049783.1_3044	K02424	298.53	375.7	1e-112	L-cystine transport system substrate-binding protein
CP049783.1_3044	K02030	76.77	162.2	6.5e-48	polar amino acid transport system substrate-binding protein

CP049783.1_3045	K10009	301.87	374.5	2.4e-112	L-cystine transport system permease protein
CP049783.1_3045	K02029	214.53	282.5	1.9e-84	polar amino acid transport system permease protein
CP049783.1_3046	K02028	386.70	444.9	1.2e-133	polar amino acid transport system ATP-binding protein [EC:7.4.2.1]
CP049783.1_3046	K10010	381.10	399.6	1.4e-120	L-cystine transport system ATP-binding protein [EC:7.4.2.1]
CP049783.1_3047	K08153	415.17	491.6	4e-148	MFS transporter, DHA1 family, multidrug resistance protein
CP049783.1_3049	K14415	269.37	384.6	3.4e-115	tRNA-splicing ligase RtcB (3'-phosphate/5'-hydroxy nucleic acid ligase) [EC:6.5.1.8]
CP049783.1_3051	K03321	345.37	525.3	7.9e-158	sulfate permease, SulP family
CP049783.1_3053	K13276	906.03	1280.4	0	bacillopeptidase F [EC:3.4.21.-]
CP049783.1_3054	K22960	74.87	115.9	2.3e-34	gallate decarboxylase subunit D
CP049783.1_3058	K00209	135.60	612.8	7e-185	enoyl-[acyl-carrier protein] reductase / trans-2-enoyl-CoA reductase (NAD+) [EC:1.3.1.9 1.3.1.44]
CP049783.1_3063	K15583	519.70	601.4	4e-181	oligopeptide transport system ATP-binding protein
CP049783.1_3063	K02031	412.13	496.3	4.1e-149	peptide/nickel transport system ATP-binding protein
CP049783.1_3064	K01728	168.73	287.7	6.1e-86	pectate lyase [EC:4.2.2.2]
CP049783.1_3070	K00355	115.87	157.1	2.5e-46	NAD(P)H dehydrogenase (quinone) [EC:1.6.5.2]
CP049783.1_3071	K05982	231.57	338.4	2.1e-101	deoxyribonuclease V [EC:3.1.21.7]
CP049783.1_3074	K03475	164.47	244.5	4.9e-73	ascorbate PTS system EIIC component
CP049783.1_3075	K26933	74.77	128.1	1.2e-37	unsaturated pyranuronate lyase [EC:4.2.99.25]
CP049783.1_3077	K07718	273.17	414.0	4.7e-124	two-component system, sensor histidine kinase YesM [EC:2.7.13.3]
CP049783.1_3078	K07720	156.87	282.3	3.1e-84	two-component system, response regulator YesN
CP049783.1_3079	K10117	246.87	357.4	6.3e-107	raffinose/stachyose/melibiose transport system substrate-binding protein
CP049783.1_3079	K02027	193.17	200.9	1.5e-59	multiple sugar transport system substrate-binding protein
CP049783.1_3080	K10118	333.37	358.4	1.3e-107	raffinose/stachyose/melibiose transport system permease protein
CP049783.1_3080	K02025	276.90	301.3	2.7e-90	multiple sugar transport system permease protein
CP049783.1_3081	K10119	296.93	355.2	1.1e-106	raffinose/stachyose/melibiose transport system permease protein
CP049783.1_3087	K04750	56.87	100.6	3.5e-29	PhnB protein
CP049783.1_3088	K01062	142.57	147.0	1.9e-43	platelet-activating factor acetylhydrolase [EC:3.1.1.47]
CP049783.1_3089	K01593	357.00	439.4	7.3e-132	aromatic-L-amino-acid/L-tryptophan decarboxylase [EC:4.1.1.28 4.1.1.105]
CP049783.1_3089	K13745	417.70	424.0	4e-127	L-2,4-diaminobutyrate decarboxylase [EC:4.1.1.86]
CP049783.1_3097	K07459	132.73	370.3	7.2e-111	putative ATP-dependent endonuclease of the OLD family
CP049783.1_3099	K07459	132.73	136.9	2.8e-40	putative ATP-dependent endonuclease of the OLD family
CP049783.1_3100	K00986	84.27	159.4	4.9e-47	RNA-directed DNA polymerase [EC:2.7.7.49]
CP049783.1_3103	K03568	311.90	541.6	9.4e-163	TldD protein
CP049783.1_3104	K03592	304.20	420.9	3e-126	PmbA protein
CP049783.1_3105	K01733	243.03	315.7	1.9e-94	threonine synthase [EC:4.2.3.1]
CP049783.1_3106	K00262	473.37	848.1	3.2e-255	glutamate dehydrogenase (NADP+) [EC:1.4.1.4]
CP049783.1_3111	K25308	264.57	359.0	7.3e-108	ferric hydroxamate/heme transport system substrate-binding protein
CP049783.1_3111	K02016	154.03	170.5	2e-50	iron complex transport system substrate-binding protein
CP049783.1_3112	K00004	375.63	481.6	4.9e-145	(R,R)-butanediol dehydrogenase / meso-butanediol dehydrogenase / diacetyl reductase [EC:1.1.1.4 1.1.1.- 1.1.1.303]
CP049783.1_3113	K02529	268.37	280.7	8.1e-84	LacI family transcriptional regulator, galactose operon repressor
CP049783.1_3118	K08164	409.17	552.9	9.9e-167	MFS transporter, DHA1 family, putative efflux transporter
CP049783.1_3119	K06976	72.13	109.3	9.4e-32	uncharacterized protein
CP049783.1_3123	K13530	199.90	234.8	3.2e-70	AraC family transcriptional regulator, regulatory protein of adaptative response / methylphosphotriester-DNA alkyltransferase methyltransferase [EC:2.1.1.-]
CP049783.1_3124	K00567	174.97	199.5	2.8e-59	methylated-DNA-[protein]-cysteine S-methyltransferase [EC:2.1.1.63]
CP049783.1_3127	K02073	57.57	307.2	7.1e-92	D-methionine transport system substrate-binding protein
CP049783.1_3128	K08969	544.47	579.5	1e-174	L-glutamine---4-(methylsulfanyl)-2-oxobutanoate aminotransferase [EC:2.6.1.117]
CP049783.1_3133	K03528	63.83	103.7	3.6e-30	cell division protein ZipA
CP049783.1_3140	K22227	372.93	510.1	2.3e-153	AdoMet-dependent heme synthase [EC:1.3.98.6]
CP049783.1_3141	K00231	144.83	483.0	4.3e-145	protoporphyrinogen/coproporphyrinogen III oxidase [EC:1.3.3.4 1.3.3.15]
CP049783.1_3142	K07322	56.20	179.1	3.7e-53	regulator of cell morphogenesis and NO signaling
CP049783.1_3143	K02020	109.77	286.1	1.7e-85	molybdate transport system substrate-binding protein
CP049783.1_3144	K02018	245.63	277.7	6.5e-83	molybdate transport system permease protein
CP049783.1_3145	K00370	817.30	1987.2	0	nitrate reductase / nitrite oxidoreductase, alpha subunit [EC:1.7.5.1 1.7.99.-]
CP049783.1_3146	K00371	338.43	953.7	4.3e-287	nitrate reductase / nitrite oxidoreductase, beta subunit [EC:1.7.5.1 1.7.99.-]
CP049783.1_3147	K00373	70.20	137.5	2.1e-40	nitrate reductase molybdenum cofactor assembly chaperone NarJ/NarW
CP049783.1_3148	K00374	179.63	294.9	3e-88	nitrate reductase gamma subunit [EC:1.7.5.1 1.7.99.-]
CP049783.1_3149	K10851	107.67	156.3	2.2e-46	nitrogen regulatory protein A

CP049783.1_3150	K07683	254.13	394.5	1.2e-118	two-component system, NarL family, sensor histidine kinase NreB [EC:2.7.13.3]
CP049783.1_3151	K07696	252.77	322.7	2.7e-97	two-component system, NarL family, response regulator NreC
CP049783.1_3152	K02575	149.60	330.7	6e-99	MFS transporter, NNP family, nitrate/nitrite transporter
CP049783.1_3154	K03635	25.83	189.2	3.9e-56	molybdopterin synthase catalytic subunit [EC:2.8.1.12]
CP049783.1_3155	K03636	36.60	62.9	1.2e-17	sulfur-carrier protein
CP049783.1_3156	K03639	198.23	423.4	4.5e-127	GTP 3',8-cyclase [EC:4.1.99.22]
CP049783.1_3157	K03488	318.67	343.6	3.7e-103	beta-glucoside operon transcriptional antiterminator
CP049783.1_3158	K02757	642.13	827.1	3.4e-249	beta-glucoside PTS system EIICBA component [EC:2.7.1.-]
CP049783.1_3159	K01223	479.40	797.9	1.3e-240	6-phospho-beta-glucosidase [EC:3.2.1.86]
CP049783.1_3160	K03406	65.50	91.0	2.5e-26	methyl-accepting chemotaxis protein
CP049783.1_3162	K19707	148.60	419.6	1.3e-126	sigma-B regulation protein RsbQ
CP049783.1_3167	K03406	65.50	284.8	4.8e-85	methyl-accepting chemotaxis protein
CP049783.1_3173	K05989	232.07	1102.2	0	alpha-L-rhamnosidase [EC:3.2.1.40]
CP049783.1_3176	K02204	139.27	176.0	4.4e-52	homoserine kinase type II [EC:2.7.1.39]
CP049783.1_3184	K07718	273.17	384.4	4.2e-115	two-component system, sensor histidine kinase YesM [EC:2.7.13.3]
CP049783.1_3185	K07720	156.87	369.5	1.2e-110	two-component system, response regulator YesN
CP049783.1_3186	K17318	176.50	327.3	7.4e-98	putative aldouronate transport system substrate-binding protein
CP049783.1_3187	K17319	220.03	370.8	2.4e-111	putative aldouronate transport system permease protein
CP049783.1_3188	K17320	270.87	353.8	2.2e-106	putative aldouronate transport system permease protein
CP049783.1_3189	K05349	305.00	800.2	9e-241	beta-glucosidase [EC:3.2.1.21]
CP049783.1_3190	K01191	442.83	928.4	1.9e-279	alpha-mannosidase [EC:3.2.1.24]
CP049783.1_3191	K09704	233.57	569.3	4.2e-171	uncharacterized protein
CP049783.1_3194	K07002	58.47	140.7	2.4e-41	serine hydrolase [EC:3.-.-.-]
CP049783.1_3195	K03406	65.50	198.3	7.9e-59	methyl-accepting chemotaxis protein
CP049783.1_3202	K05795	235.30	237.3	8.7e-71	tellurium resistance protein TerD
CP049783.1_3205	K03406	65.50	241.0	9.2e-72	methyl-accepting chemotaxis protein
CP049783.1_3211	K07727	44.53	67.1	5.3e-19	putative transcriptional regulator
CP049783.1_3233	K07453	167.57	185.6	4.8e-55	putative restriction endonuclease
CP049783.1_3233	K07451	41.70	75.3	1.5e-21	5-methylcytosine-specific restriction enzyme A [EC:3.1.21.-]
CP049783.1_3242	K07727	44.53	67.8	3.3e-19	putative transcriptional regulator
CP049783.1_3246	K02016	154.03	157.5	1.7e-46	iron complex transport system substrate-binding protein
CP049783.1_3249	K14189	194.93	309.1	9.2e-93	uncharacterized oxidoreductase [EC:1.-.-.-]
CP049783.1_3270	K01011	189.10	297.9	3.1e-89	thiosulfate/3-mercaptopyruvate sulfurtransferase [EC:2.8.1.1 2.8.1.2]
CP049783.1_3273	K05822	338.70	355.4	3.9e-107	tetrahydrodipicolinate N-acetyltransferase [EC:2.3.1.89]
CP049783.1_3274	K05823	359.63	623.4	3.3e-188	N-acetyldiaminopimelate deacetylase [EC:3.5.1.47]
CP049783.1_3275	K11072	493.17	564.7	6.1e-170	spermidine/putrescine transport system ATP-binding protein [EC:7.6.2.11]
CP049783.1_3276	K11071	289.37	312.4	1e-93	spermidine/putrescine transport system permease protein
CP049783.1_3277	K11070	283.70	330.7	4.6e-99	spermidine/putrescine transport system permease protein
CP049783.1_3278	K11069	354.53	434.2	2e-130	spermidine/putrescine transport system substrate-binding protein
CP049783.1_3279	K00841	489.23	530.3	1.5e-159	aminotransferase [EC:2.6.1.-]
CP049783.1_3279	K10907	500.80	520.9	1e-156	aminotransferase [EC:2.6.1.-]
CP049783.1_3280	K07032	75.30	151.3	8.8e-45	uncharacterized protein
CP049783.1_3281	K19113	120.03	184.5	2.9e-55	acetyltransferase [EC:2.3.1.-]
CP049783.1_3282	K01744	667.80	760.7	3.4e-229	aspartate ammonia-lyase [EC:4.3.1.1]
CP049783.1_3283	K07448	34.87	113.0	4.2e-33	restriction system protein
CP049783.1_3284	K14338	601.20	1493.7	0	cytochrome P450 / NADPH-cytochrome P450 reductase [EC:1.14.14.1 1.6.2.4]
CP049783.1_3285	K07335	118.73	303.9	7.9e-91	basic membrane protein A and related proteins
CP049783.1_3286	K03406	65.50	213.4	2.1e-63	methyl-accepting chemotaxis protein
CP049783.1_3287	K07720	156.87	184.3	1.4e-54	two-component system, response regulator YesN
CP049783.1_3288	K07795	76.40	331.1	3.8e-99	putative tricarboxylic transport membrane protein
CP049783.1_3289	K02526	159.17	433.7	1.1e-130	2-keto-3-deoxygluconate permease
CP049783.1_3292	K00123	847.33	1025.7	5.2e-309	formate dehydrogenase major subunit [EC:1.17.1.9]
CP049783.1_3293	K03639	198.23	425.0	1.5e-127	GTP 3',8-cyclase [EC:4.1.99.22]
CP049783.1_3294	K02379	83.90	294.5	4.2e-88	FdhD protein
CP049783.1_3295	K03636	36.60	40.8	7.7e-11	sulfur-carrier protein
CP049783.1_3296	K21142	218.23	248.2	2.6e-74	MoaE-MoaD fusion protein [EC:2.8.1.12]

CP049783.1_3296	K03635	25.83	198.4	6.2e-59	molybdopterin synthase catalytic subunit [EC:2.8.1.12]
CP049783.1_3297	K03148	333.67	387.7	1.7e-116	sulfur carrier protein ThiS adenylyltransferase [EC:2.7.7.73]
CP049783.1_3298	K02020	109.77	297.1	8.1e-89	molybdate transport system substrate-binding protein
CP049783.1_3299	K02018	245.63	275.9	2.3e-82	molybdate transport system permease protein
CP049783.1_3301	K10761	94.13	138.7	6.6e-41	tRNA(His) guanylyltransferase [EC:2.7.7.79]
CP049783.1_3311	K03091	189.70	235.8	2.2e-70	RNA polymerase sigma-E/F/G factor
CP049783.1_3319	K17319	220.03	370.5	2.9e-111	putative aldouronate transport system permease protein
CP049783.1_3320	K17320	270.87	342.3	6.9e-103	putative aldouronate transport system permease protein
CP049783.1_3321	K17318	176.50	286.9	1.2e-85	putative aldouronate transport system substrate-binding protein
CP049783.1_3322	K07720	156.87	185.8	5e-55	two-component system, response regulator YesN
CP049783.1_3323	K20628	101.80	264.5	5.2e-79	expansin
CP049783.1_3324	K07038	27.13	76.8	4.7e-22	inner membrane protein
CP049783.1_3325	K01261	363.67	495.9	1.4e-149	glutamyl aminopeptidase [EC:3.4.11.7]
CP049783.1_3326	K06405	98.60	227.6	1.6e-68	stage V sporulation protein AC
CP049783.1_3327	K06406	180.47	532.8	7.3e-161	stage V sporulation protein AD
CP049783.1_3328	K06407	58.07	138.8	2.6e-41	stage V sporulation protein AE
CP049783.1_3329	K03924	113.70	438.5	1.2e-131	MoxR-like ATPase [EC:3.6.3.-]
CP049783.1_3331	K22452	104.63	243.1	2.5e-72	protein-glutamine gamma-glutamyltransferase [EC:2.3.2.13]
CP049783.1_3332	K07015	105.17	248.0	4e-74	putative phosphatase [EC:3.1.3.-]
CP049783.1_3333	K06948	254.17	498.7	2.8e-150	30S ribosome assembly GTPase
CP049783.1_3334	K00014	294.53	367.3	4e-110	shikimate dehydrogenase [EC:1.1.1.25]
CP049783.1_3335	K07574	51.13	123.7	2.2e-36	RNA-binding protein
CP049783.1_3339	K09710	113.50	162.8	2.9e-48	ribosome-associated protein
CP049783.1_3340	K00243	150.27	309.7	8e-93	uncharacterized protein
CP049783.1_3342	K01821	25.90	34.9	4.6e-09	4-oxalocrotonate tautomerase [EC:5.3.2.6]
CP049783.1_3343	K01869	385.87	1293.5	0	leucyl-tRNA synthetase [EC:6.1.1.4]
CP049783.1_3344	K02239	266.50	389.5	2e-117	competence protein ComER
CP049783.1_3344	K00286	164.07	193.6	2e-57	pyrroline-5-carboxylate reductase [EC:1.5.1.2]
CP049783.1_3345	K02237	75.73	110.0	5.3e-32	competence protein ComEA
CP049783.1_3346	K01493	80.20	146.1	5e-43	dCMP deaminase [EC:3.5.4.12]
CP049783.1_3347	K02238	64.77	507.5	2.6e-152	competence protein ComEC
CP049783.1_3348	K03088	96.50	158.2	8.8e-47	RNA polymerase sigma-70 factor, ECF subfamily
CP049783.1_3351	K02340	96.93	256.5	1.8e-76	DNA polymerase III subunit delta [EC:2.7.7.7]
CP049783.1_3352	K02968	24.97	108.4	1.4e-31	small subunit ribosomal protein S20
CP049783.1_3353	K06012	187.50	450.1	1.2e-135	spore protease [EC:3.4.24.78]
CP049783.1_3354	K06385	107.47	346.0	1.1e-103	stage II sporulation protein P
CP049783.1_3356	K03596	927.43	1148.9	0	GTP-binding protein LepA
CP049783.1_3357	K02495	261.80	311.9	2.2e-93	oxygen-independent coproporphyrinogen III oxidase [EC:1.3.98.3]
CP049783.1_3360	K03705	67.57	406.9	4.1e-122	heat-inducible transcriptional repressor
CP049783.1_3361	K03687	31.27	212.9	2.4e-63	molecular chaperone GrpE
CP049783.1_3362	K04043	801.23	935.0	1.3e-281	molecular chaperone DnaK
CP049783.1_3363	K03686	406.37	525.2	8.3e-158	molecular chaperone DnaJ
CP049783.1_3364	K11614	633.27	693.9	3.6e-209	two-component system, CitB family, sensor histidine kinase MalK [EC:2.7.13.3]
CP049783.1_3365	K11615	291.70	312.2	5.8e-94	two-component system, CitB family, response regulator MalR
CP049783.1_3366	K11616	246.63	546.1	1.3e-164	malate:Na+ symporter
CP049783.1_3367	K00027	507.30	588.8	5.8e-177	malate dehydrogenase (oxaloacetate-decarboxylating) [EC:1.1.1.38]
CP049783.1_3370	K02687	125.73	344.5	3e-103	ribosomal protein L11 methyltransferase [EC:2.1.1.-]
CP049783.1_3372	K09761	57.73	261.7	2.7e-78	16S rRNA (uracil1498-N3)-methyltransferase [EC:2.1.1.193]
CP049783.1_3373	K18707	413.13	605.5	2.5e-182	threonylcarbamoyladenosine tRNA methylthiotransferase MtaB [EC:2.8.4.5]
CP049783.1_3375	K03324	151.43	334.1	5.6e-100	phosphate:Na+ symporter
CP049783.1_3376	K06969	218.10	522.3	6.6e-157	23S rRNA (cytosine1962-C5)-methyltransferase [EC:2.1.1.191]
CP049783.1_3377	K16899	216.03	627.1	2.6e-188	ATP-dependent helicase/nuclease subunit B [EC:5.6.2.4 3.1.-.-]
CP049783.1_3378	K16898	483.47	1062.4	1.1e-319	ATP-dependent helicase/nuclease subunit A [EC:5.6.2.4 3.1.-.-]
CP049783.1_3379	K03547	134.00	291.4	4.2e-87	DNA repair protein SbcD/Mre11
CP049783.1_3380	K03546	183.10	465.9	1.2e-139	DNA repair protein SbcC/Rad50
CP049783.1_3383	K02503	85.60	135.8	4.8e-40	histidine triad (HIT) family protein [EC:3.9.1.-]

CP049783.1_3384	K02970	28.27	67.8	3.4e-19	small subunit ribosomal protein S21
CP049783.1_3385	K09117	49.67	182.6	3.9e-54	uncharacterized protein
CP049783.1_3386	K07403	121.90	379.1	1.3e-113	membrane-bound serine protease (ClpP class)
CP049783.1_3389	K03294	476.00	603.2	1.7e-181	basic amino acid/polyamine antiporter, APA family
CP049783.1_3391	K06438	146.87	440.7	1.9e-132	similar to stage IV sporulation protein
CP049783.1_3392	K06217	216.80	529.1	5.7e-159	phosphate starvation-inducible protein PhoH and related proteins
CP049783.1_3393	K07037	135.10	761.7	2.7e-229	cyclic-di-AMP phosphodiesterase PgpH [EC:3.1.4.-]
CP049783.1_3394	K07042	60.10	173.3	2.2e-51	probable rRNA maturation factor
CP049783.1_3395	K00887	148.73	166.4	1.5e-49	undecaprenol kinase [EC:2.7.1.66]
CP049783.1_3396	K03595	144.20	441.6	1.4e-132	GTPase
CP049783.1_3398	K03584	28.20	156.6	3.1e-46	DNA repair protein RecO (recombination protein O)
CP049783.1_3400	K01878	507.67	595.8	2.3e-179	glycyl-tRNA synthetase alpha chain [EC:6.1.1.14]
CP049783.1_3401	K01879	573.03	879.9	8.3e-265	glycyl-tRNA synthetase beta chain [EC:6.1.1.14]
CP049783.1_3402	K09768	74.23	170.5	1.5e-50	uncharacterized protein
CP049783.1_3403	K02316	140.00	528.6	8.1e-159	DNA primase [EC:2.7.7.101]
CP049783.1_3404	K03086	466.80	588.1	7.1e-177	RNA polymerase primary sigma factor
CP049783.1_3404	K03087	413.53	419.3	4.6e-126	RNA polymerase nonessential primary-like sigma factor
CP049783.1_3404	K03093	118.63	130.7	2.6e-38	RNA polymerase sigma-I factor
CP049783.1_3406	K06967	59.70	293.6	6.7e-88	tRNA (adenine22-N1)-methyltransferase [EC:2.1.1.217]
CP049783.1_3407	K22391	167.17	186.8	1.7e-55	GTP cyclohydrolase I [EC:3.5.4.16]
CP049783.1_3409	K05825	385.80	503.7	2.2e-151	2-aminoadipate transaminase [EC:2.6.1.-]
CP049783.1_3410	K05825	385.80	394.2	3.3e-118	2-aminoadipate transaminase [EC:2.6.1.-]
CP049783.1_3412	K08651	401.13	489.8	3.2e-147	thermitase [EC:3.4.21.66]
CP049783.1_3412	K14645	303.80	372.2	1.4e-111	serine protease [EC:3.4.21.-]
CP049783.1_3417	K04487	287.40	505.5	5e-152	cysteine desulfurase [EC:2.8.1.7]
CP049783.1_3418	K03151	126.60	519.9	3.1e-156	tRNA uracil 4-sulfurtransferase [EC:2.8.1.4]
CP049783.1_3422	K06207	327.10	1023.5	1.4e-308	GTP-binding protein
CP049783.1_3423	K01005	193.97	262.4	3.1e-78	polyisoprenyl-teichoic acid--peptidoglycan teichoic acid transferase [EC:2.7.8.-]
CP049783.1_3425	K07175	228.10	475.7	7.7e-143	PhoH-like ATPase
CP049783.1_3427	K06314	87.53	253.8	4.5e-76	prespore-specific regulator
CP049783.1_3428	K00077	75.90	255.3	4.1e-76	2-dehydropantoate 2-reductase [EC:1.1.1.169]
CP049783.1_3430	K15580	368.50	632.9	2.4e-190	oligopeptide transport system substrate-binding protein
CP049783.1_3430	K02035	249.67	377.6	4e-113	peptide/nickel transport system substrate-binding protein
CP049783.1_3431	K15581	345.63	383.5	3.4e-115	oligopeptide transport system permease protein
CP049783.1_3431	K02033	263.63	353.1	8.2e-106	peptide/nickel transport system permease protein
CP049783.1_3434	K10823	502.03	522.0	8e-157	oligopeptide transport system ATP-binding protein
CP049783.1_3434	K02032	420.67	478.0	1.9e-143	peptide/nickel transport system ATP-binding protein
CP049783.1_3435	K22136	114.20	529.0	4.8e-159	bacillithiol synthase
CP049783.1_3436	K01251	60.67	483.5	2.4e-145	adenosylhomocysteinase [EC:3.13.2.1]
CP049783.1_3437	K03925	34.60	194.9	2.8e-58	transcriptional regulator MraZ
CP049783.1_3441	K08384	704.83	771.8	2.4e-232	stage V sporulation protein D (sporulation-specific penicillin-binding protein)
CP049783.1_3441	K03587	463.30	572.8	3.4e-172	cell division protein FtsI (penicillin-binding protein 3) [EC:3.4.16.4]
CP049783.1_3442	K08384	704.83	848.9	1.2e-255	stage V sporulation protein D (sporulation-specific penicillin-binding protein)
CP049783.1_3442	K03587	463.30	609.9	2.1e-183	cell division protein FtsI (penicillin-binding protein 3) [EC:3.4.16.4]
CP049783.1_3443	K01928	408.67	655.7	2.1e-197	UDP-N-acetylmuramoyl-L-alanyl-D-glutamate--2,6-diaminopimelate ligase [EC:6.3.2.13]
CP049783.1_3444	K01929	327.77	531.7	5.6e-160	UDP-N-acetylmuramoyl-tripeptide--D-alanyl-D-alanine ligase [EC:6.3.2.10]
CP049783.1_3445	K01000	186.50	417.6	2e-125	phospho-N-acetylmuramoyl-pentapeptide-transferase [EC:2.7.8.13]
CP049783.1_3446	K01925	363.80	508.3	8e-153	UDP-N-acetylmuramoylalanine--D-glutamate ligase [EC:6.3.2.9]
CP049783.1_3447	K03588	356.93	454.9	8.4e-137	cell division protein FtsW
CP049783.1_3448	K02563	193.67	395.8	9.2e-119	UDP-N-acetylglucosamine--N-acetylmuramyl-(pentapeptide) pyrophosphoryl-undecaprenol N-acetylglucosamine transferase [EC:2.4.1.227]
CP049783.1_3449	K00075	66.30	216.4	1.7e-64	UDP-N-acetylmuramate dehydrogenase [EC:1.3.1.98]
CP049783.1_3450	K00790	172.73	608.3	3.1e-183	UDP-N-acetylglucosamine 1-carboxyvinyltransferase [EC:2.5.1.7]
CP049783.1_3451	K03589	42.57	89.9	7.8e-26	cell division protein FtsQ
CP049783.1_3452	K03590	124.13	491.1	1e-147	cell division protein FtsA
CP049783.1_3453	K03531	142.77	573.1	1.2e-172	cell division protein FtsZ
CP049783.1_3454	K06383	109.60	364.8	1.2e-109	stage II sporulation protein GA (sporulation sigma-E factor processing peptidase) [EC:3.4.23.-]

CP049783.1_3455	K03091	189.70	348.7	9e-105	RNA polymerase sigma-E/F/G factor
CP049783.1_3456	K03091	189.70	268.1	3.2e-80	RNA polymerase sigma-E/F/G factor
CP049783.1_3458	K05810	84.47	306.4	1.1e-91	purine-nucleoside/S-methyl-5'-thioadenosine phosphorylase / adenosine deaminase [EC:2.4.2.1 2.4.2.28 3.5.4.4]
CP049783.1_3459	K06997	64.17	270.8	6.1e-81	PLP dependent protein
CP049783.1_3460	K09772	38.13	150.4	1.5e-44	cell division inhibitor SepF
CP049783.1_3461	K02221	21.77	90.4	4.3e-26	YggT family protein
CP049783.1_3463	K04074	47.10	210.8	7.6e-63	cell division initiation protein
CP049783.1_3464	K01870	467.30	1118.9	0	isoleucyl-tRNA synthetase [EC:6.1.1.5]
CP049783.1_3466	K06204	35.73	81.1	4.4e-23	RNA polymerase-binding transcription factor
CP049783.1_3467	K03101	31.87	179.0	3e-53	signal peptidase II [EC:3.4.23.36]
CP049783.1_3468	K06180	285.23	419.3	6.3e-126	23S rRNA pseudouridine1911/1915/1917 synthase [EC:5.4.99.23]
CP049783.1_3469	K02825	96.10	285.3	1.4e-85	pyrimidine operon attenuation protein / uracil phosphoribosyltransferase [EC:2.4.2.9]
CP049783.1_3470	K00609	254.20	410.8	2.1e-123	aspartate carbamoyltransferase catalytic subunit [EC:2.1.3.2]
CP049783.1_3471	K01465	336.03	481.9	1.1e-144	dihydroorotase [EC:3.5.2.3]
CP049783.1_3472	K01956	530.93	619.4	4.2e-186	carbamoyl-phosphate synthase small subunit [EC:6.3.5.5]
CP049783.1_3473	K01955	1479.70	1705.2	0	carbamoyl-phosphate synthase large subunit [EC:6.3.5.5]
CP049783.1_3474	K01591	129.83	265.1	4.3e-79	orotidine-5'-phosphate decarboxylase [EC:4.1.1.23]
CP049783.1_3475	K00762	105.33	204.3	9.1e-61	orotate phosphoribosyltransferase [EC:2.4.2.10]
CP049783.1_3480	K07720	156.87	184.0	1.9e-54	two-component system, response regulator YesN
CP049783.1_3483	K25674	464.17	468.3	3.3e-141	polygalacturonan/rhamnogalacturonan transport system permease protein
CP049783.1_3483	K17319	220.03	436.4	2.9e-131	putative aldouronate transport system permease protein
CP049783.1_3485	K25673	546.47	662.2	1.5e-199	polygalacturonan/rhamnogalacturonan transport system substrate-binding protein
CP049783.1_3485	K17318	176.50	316.0	2e-94	putative aldouronate transport system substrate-binding protein
CP049783.1_3488	K01051	75.37	301.0	5.5e-90	pectinesterase [EC:3.1.1.11]
CP049783.1_3489	K01198	271.87	450.8	3.8e-135	xylan 1,4-beta-xylosidase [EC:3.2.1.37]
CP049783.1_3490	K21900	290.47	377.6	2.2e-113	LysR family transcriptional regulator, transcriptional activator of the cysJI operon
CP049783.1_3490	K21703	291.57	295.8	1.4e-88	LysR family transcriptional regulator, low CO2-responsive transcriptional regulator
CP049783.1_3492	K07273	48.73	282.8	1.2e-84	lysozyme
CP049783.1_3497	K27245	246.57	278.4	3.5e-83	peptidoglycan lytic transglycosylase [EC:4.2.2.29]
CP049783.1_3497	K01449	48.60	175.5	4e-52	cell wall hydrolase
CP049783.1_3501	K00005	325.77	534.7	3e-161	glycerol dehydrogenase [EC:1.1.1.6]
CP049783.1_3503	K01218	70.20	273.7	1.3e-81	mannan endo-1,4-beta-mannosidase [EC:3.2.1.78]
CP049783.1_3506	K09771	35.27	137.1	1.8e-40	small multidrug resistance family-3 protein
CP049783.1_3512	K01167	44.03	56.5	1.2e-15	ribonuclease T1 [EC:4.6.1.24]
CP049783.1_3513	K03623	37.70	103.6	1.3e-30	ribonuclease inhibitor
CP049783.1_3515	K01069	139.83	143.0	3.9e-42	hydroxyacylglutathione hydrolase [EC:3.1.2.6]
CP049783.1_3517	K06133	60.17	73.5	5.7e-21	4'-phosphopantetheinyl transferase [EC:2.7.8.-]
CP049783.1_3518	K15337	69.20	109.4	2e-32	polyketide biosynthesis acyl carrier protein
CP049783.1_3519	K00646	418.90	624.0	3.9e-188	malonyl-[acp] decarboxylase [EC:4.1.1.124]
CP049783.1_3520	K15311	506.87	726.6	2.3e-219	3-carboxymethyl-3-hydroxy-acyl-[acp] synthase [EC:2.3.3.22]
CP049783.1_3521	K15312	215.27	370.6	1e-111	3-carboxymethyl-3-hydroxy-acyl-[acp] dehydratase [EC:4.2.1.181]
CP049783.1_3522	K15313	355.33	388.1	3.9e-117	4-carboxy-3-alkylbut-2-enoyl-[acp] decarboxylase [EC:4.1.1.125]
CP049783.1_3524	K13614	7067.73	8570.3	0	bacillaene biosynthesis, polyketide synthase / nonribosomal peptide synthetase PksN/BaeN
CP049783.1_3525	K13613	2486.10	2565.7	0	bacillaene polyketide synthase PksM/BaeM
CP049783.1_3529	K15328	377.47	456.7	1.2e-137	bacillaene synthase trans-acting acyltransferase
CP049783.1_3530	K15329	513.20	1206.7	0	trans-AT polyketide synthase, acyltransferase and oxidoreductase domains
CP049783.1_3530	K00645	247.17	330.2	9.1e-99	[acyl-carrier-protein] S-malonyltransferase [EC:2.3.1.39]
CP049783.1_3531	K01876	301.23	840.2	8.9e-253	aspartyl-tRNA synthetase [EC:6.1.1.12]
CP049783.1_3533	K03648	63.37	378.8	1.2e-113	uracil-DNA glycosylase [EC:3.2.2.27]
CP049783.1_3540	K07718	273.17	418.9	1.5e-125	two-component system, sensor histidine kinase YesM [EC:2.7.13.3]
CP049783.1_3541	K07720	156.87	342.7	1.5e-102	two-component system, response regulator YesN
CP049783.1_3542	K17318	176.50	293.9	9.5e-88	putative aldouronate transport system substrate-binding protein
CP049783.1_3543	K17319	220.03	406.4	3.7e-122	putative aldouronate transport system permease protein
CP049783.1_3544	K17320	270.87	338.2	1.2e-101	putative aldouronate transport system permease protein
CP049783.1_3545	K12308	328.20	412.2	1.6e-123	beta-galactosidase [EC:3.2.1.23]
CP049783.1_3546	K01224	233.03	398.7	2e-119	arabinogalactan endo-1,4-beta-galactosidase [EC:3.2.1.89]

CP049783.1_3547	K03406	65.50	284.2	7.4e-85	methyl-accepting chemotaxis protein
CP049783.1_3548	K13936	190.93	211.2	7.4e-63	malonate transporter and related proteins
CP049783.1_3549	K00029	287.77	626.1	2.2e-188	malate dehydrogenase (oxaloacetate-decarboxylating)(NADP+) [EC:1.1.1.40]
CP049783.1_3549	K00027	507.30	539.3	5.6e-162	malate dehydrogenase (oxaloacetate-decarboxylating) [EC:1.1.1.38]
CP049783.1_3550	K02575	149.60	149.6	3.1e-44	MFS transporter, NNP family, nitrate/nitrite transporter
CP049783.1_3551	K02575	149.60	162.1	5.2e-48	MFS transporter, NNP family, nitrate/nitrite transporter
CP049783.1_3552	K26139	720.47	816.9	4.9e-246	nitrite reductase [NAD(P)H] large subunit [EC:1.7.1.4]
CP049783.1_3554	K00766	109.93	231.0	7.6e-69	anthranilate phosphoribosyltransferase [EC:2.4.2.18]
CP049783.1_3556	K26139	720.47	1027.3	1.1e-309	nitrite reductase [NAD(P)H] large subunit [EC:1.7.1.4]
CP049783.1_3557	K26138	104.60	152.5	1.9e-45	nitrite reductase [NAD(P)H] small subunit [EC:1.7.1.4]
CP049783.1_3563	K01585	258.33	580.4	2.4e-174	arginine decarboxylase [EC:4.1.1.19]
CP049783.1_3567	K00033	157.37	832.4	9.3e-251	6-phosphogluconate dehydrogenase [EC:1.1.1.44 1.1.1.343]
CP049783.1_3569	K00800	150.47	416.8	4.6e-125	3-phosphoshikimate 1-carboxyvinyltransferase [EC:2.5.1.19]
CP049783.1_3570	K06929	81.97	201.5	3.6e-60	uncharacterized protein
CP049783.1_3572	K08483	678.63	785.6	1.8e-236	phosphoenolpyruvate-protein phosphotransferase (PTS system enzyme I) [EC:2.7.3.9]
CP049783.1_3573	K05520	175.93	242.1	2.6e-72	deglycase [EC:3.5.1.124]
CP049783.1_3575	K26959	82.33	143.9	1.5e-42	monothiol bacilliredoxin
CP049783.1_3576	K02529	268.37	373.0	8.6e-112	LacI family transcriptional regulator, galactose operon repressor
CP049783.1_3577	K01243	103.73	260.6	7.7e-78	adenosylhomocysteine nucleosidase [EC:3.2.2.9]
CP049783.1_3578	K04766	139.97	326.3	1.5e-98	acetoin utilization protein AcuA [EC:2.3.1.-]
CP049783.1_3580	K03693	751.63	923.0	6.5e-278	penicillin-binding protein 1B
CP049783.1_3581	K01866	88.87	383.2	8.8e-115	tyrosyl-tRNA synthetase [EC:6.1.1.1]
CP049783.1_3582	K13653	208.93	260.9	7.2e-78	AraC family transcriptional regulator
CP049783.1_3583	K02986	67.73	206.4	2.7e-61	small subunit ribosomal protein S4
CP049783.1_3586	K19689	69.60	390.7	4.4e-117	aminopeptidase [EC:3.4.11.-]
CP049783.1_3589	K07300	76.70	379.5	7.8e-114	Ca2+:H+ antiporter
CP049783.1_3591	K03588	356.93	421.7	1e-126	cell division protein FtsW
CP049783.1_3592	K01436	522.37	600.0	4.4e-181	amidohydrolase [EC:3.5.1.-]
CP049783.1_3594	K10843	221.37	648.5	4.5e-195	DNA excision repair protein ERCC-3 [EC:5.6.2.4]
CP049783.1_3599	K21900	290.47	334.8	2.3e-100	LysR family transcriptional regulator, transcriptional activator of the cysJI operon
CP049783.1_3599	K21703	291.57	316.9	5.2e-95	LysR family transcriptional regulator, low CO2-responsive transcriptional regulator
CP049783.1_3600	K07053	178.93	360.1	7.5e-108	3',5'-nucleoside bisphosphate phosphatase [EC:3.1.3.97]
CP049783.1_3602	K01537	992.03	1193.8	0	P-type Ca2+ transporter type 2C [EC:7.2.2.10]
CP049783.1_3603	K01778	61.00	375.0	1.2e-112	diaminopimelate epimerase [EC:5.1.1.7]
CP049783.1_3605	K24042	407.17	854.5	1.2e-257	methionine synthase / methylenetetrahydrofolate reductase (NADH) [EC:2.1.1.13 1.5.1.54]
CP049783.1_3607	K09777	63.00	152.1	1.1e-45	extracellular matrix regulatory protein A
CP049783.1_3608	K00942	178.33	197.2	1.1e-58	guanylate kinase [EC:2.7.4.8]
CP049783.1_3609	K03060	24.57	76.8	7.1e-22	DNA-directed RNA polymerase subunit omega [EC:2.7.7.6]
CP049783.1_3610	K13038	251.03	567.8	5.2e-171	phosphopantothenoylcysteine decarboxylase / phosphopantothenate---cysteine ligase [EC:4.1.1.36 6.3.2.5]
CP049783.1_3611	K04066	138.70	936.3	6.9e-282	primosomal protein N' (replication factor Y) (superfamily II helicase) [EC:5.6.2.4]
CP049783.1_3612	K01462	69.73	222.1	3.6e-66	peptide deformylase [EC:3.5.1.88]
CP049783.1_3613	K00604	267.47	387.6	2.8e-116	methionyl-tRNA formyltransferase [EC:2.1.2.9]
CP049783.1_3615	K03500	303.70	353.1	1.5e-105	16S rRNA (cytosine967-C5)-methyltransferase [EC:2.1.1.176]
CP049783.1_3616	K06941	65.87	512.6	4.1e-154	23S rRNA (adenine2503-C2)-methyltransferase [EC:2.1.1.192]
CP049783.1_3617	K20074	131.13	306.1	2.3e-91	PPM family protein phosphatase [EC:3.1.3.16]
CP049783.1_3618	K12132	224.03	353.1	4.4e-106	eukaryotic-like serine/threonine-protein kinase [EC:2.7.11.1]
CP049783.1_3618	K11912	105.07	184.8	8.5e-55	serine/threonine-protein kinase PpkA [EC:2.7.11.1]
CP049783.1_3619	K06949	90.53	347.8	2.6e-104	ribosome biogenesis GTPase / thiamine phosphate phosphatase [EC:3.6.1.- 3.1.3.100]
CP049783.1_3620	K01783	248.20	358.3	1.4e-107	ribulose-phosphate 3-epimerase [EC:5.1.3.1]
CP049783.1_3622	K02902	22.37	63.9	6e-18	large subunit ribosomal protein L28
CP049783.1_3623	K07030	292.60	561.5	1.1e-168	fatty acid kinase [EC:2.7.2.18]
CP049783.1_3624	K25232	87.57	333.5	5.8e-100	fatty acid kinase fatty acid binding subunit
CP049783.1_3625	K03655	393.80	752.8	3.5e-226	ATP-dependent DNA helicase RecG [EC:5.6.2.4]
CP049783.1_3627	K03292	344.60	412.1	8.5e-124	glycoside/pentoside/hexuronide:cation symporter, GPH family
CP049783.1_3629	K02099	174.60	182.1	5.8e-54	AraC family transcriptional regulator, arabinose operon regulatory protein
CP049783.1_3630	K05340	244.30	249.0	2.2e-74	glucose uptake protein

CP049783.1_3631	K18954	149.17	174.3	1.5e-51	AraC family transcriptional regulator, transcriptional activator of pobA
CP049783.1_3637	K03693	751.63	889.7	7.6e-268	penicillin-binding protein 1B
CP049783.1_3644	K12267	284.57	522.5	3.3e-157	peptide methionine sulfoxide reductase msrA/msrB [EC:1.8.4.11 1.8.4.12]
CP049783.1_3644	K07304	43.27	282.5	1.6e-84	peptide-methionine (S)-S-oxide reductase [EC:1.8.4.11]
CP049783.1_3644	K07305	92.37	198.3	4.1e-59	peptide-methionine (R)-S-oxide reductase [EC:1.8.4.12]
CP049783.1_3647	K03810	165.37	311.0	2.5e-93	virulence factor
CP049783.1_3650	K01209	85.37	479.0	9e-144	alpha-L-arabinofuranosidase [EC:3.2.1.55]
CP049783.1_3651	K02099	174.60	202.0	4.9e-60	AraC family transcriptional regulator, arabinose operon regulatory protein
CP049783.1_3659	K15777	105.60	343.2	6e-103	4,5-DOPA dioxygenase extradiol [EC:1.13.11.-]
CP049783.1_3661	K04072	644.50	1421.3	0	acetaldehyde dehydrogenase / alcohol dehydrogenase [EC:1.2.1.10 1.1.1.1]
CP049783.1_3662	K00656	301.47	1364.9	0	formate C-acetyltransferase [EC:2.3.1.54]
CP049783.1_3663	K04069	127.27	253.5	1.1e-75	pyruvate formate lyase activating enzyme [EC:1.97.1.4]
CP049783.1_3665	K02076	141.93	225.9	7.8e-68	Fur family transcriptional regulator, zinc uptake regulator
CP049783.1_3666	K08998	23.87	132.8	4.6e-39	uncharacterized protein
CP049783.1_3667	K01874	185.23	720.3	8.9e-217	methionyl-tRNA synthetase [EC:6.1.1.10]
CP049783.1_3668	K06196	133.63	224.3	8.5e-67	cytochrome c-type biogenesis protein
CP049783.1_3669	K03716	305.83	547.3	3.2e-165	spore photoproduct lyase [EC:4.1.99.14]
CP049783.1_3670	K11924	157.13	217.0	9.3e-65	DtxR family transcriptional regulator, manganese transport regulator
CP049783.1_3672	K07001	95.57	146.9	2.4e-43	NTE family protein
CP049783.1_3675	K23227	189.93	298.2	2.2e-89	ferric hydroxamate transport system substrate-binding protein
CP049783.1_3677	K01262	268.13	295.0	4.4e-88	Xaa-Pro aminopeptidase [EC:3.4.11.9]
CP049783.1_3678	K02356	149.23	283.9	5.4e-85	elongation factor P
CP049783.1_3679	K00928	394.00	524.3	9.7e-158	aspartate kinase [EC:2.7.2.4]
CP049783.1_3680	K06390	237.00	452.9	2.4e-136	stage III sporulation protein AA
CP049783.1_3681	K06391	68.77	196.4	1.2e-58	stage III sporulation protein AB
CP049783.1_3682	K06393	50.83	169.7	7.3e-51	stage III sporulation protein AD
CP049783.1_3683	K06394	111.20	524.1	7.2e-158	stage III sporulation protein AE
CP049783.1_3684	K06395	34.30	174.7	8.2e-52	stage III sporulation protein AF
CP049783.1_3685	K06396	67.37	212.5	1.7e-63	stage III sporulation protein AG
CP049783.1_3686	K06397	56.83	131.9	6.7e-39	stage III sporulation protein AH
CP049783.1_3687	K02160	93.20	181.2	7.7e-54	acetyl-CoA carboxylase biotin carboxyl carrier protein
CP049783.1_3688	K01961	729.57	814.5	1.3e-245	acetyl-CoA carboxylase, biotin carboxylase subunit [EC:6.4.1.2 6.3.4.14]
CP049783.1_3693	K03625	88.60	173.6	2e-51	transcription antitermination protein NusB
CP049783.1_3694	K01491	414.73	470.7	1.4e-141	methylenetetrahydrofolate dehydrogenase (NADP+) / methenyltetrahydrofolate cyclohydrolase [EC:1.5.1.5 3.5.4.9]
CP049783.1_3695	K03601	56.10	562.0	5.3e-169	exodeoxyribonuclease VII large subunit [EC:3.1.11.6]
CP049783.1_3696	K03602	21.00	90.9	2.2e-26	exodeoxyribonuclease VII small subunit [EC:3.1.11.6]
CP049783.1_3697	K13789	367.20	387.9	1.6e-116	geranylgeranyl diphosphate synthase, type II [EC:2.5.1.1 2.5.1.10 2.5.1.29]
CP049783.1_3698	K01662	325.00	921.4	1.1e-277	1-deoxy-D-xylulose-5-phosphate synthase [EC:2.2.1.7]
CP049783.1_3699	K06442	67.40	388.0	1.7e-116	23S rRNA (cytidine1920-2'-O)/16S rRNA (cytidine1409-2'-O)-methyltransferase [EC:2.1.1.226 2.1.1.227]
CP049783.1_3701	K03402	48.50	186.6	1.5e-55	transcriptional regulator of arginine metabolism
CP049783.1_3702	K03631	130.47	696.4	1.2e-209	DNA repair protein RecN (Recombination protein N)
CP049783.1_3703	K06399	223.57	546.2	2.2e-164	stage IV sporulation protein B [EC:3.4.21.116]
CP049783.1_3704	K07699	109.57	414.5	5.3e-125	two-component system, response regulator, stage 0 sporulation protein A
CP049783.1_3706	K03406	65.50	322.3	2.2e-96	methyl-accepting chemotaxis protein
CP049783.1_3709	K06377	63.37	265.6	1.9e-79	sporulation-control protein
CP049783.1_3710	K00857	82.33	147.6	1.7e-43	thymidine kinase [EC:2.7.1.21]
CP049783.1_3712	K00382	465.60	610.7	8e-184	dihydrolipoyl dehydrogenase [EC:1.8.1.4]
CP049783.1_3713	K00166	419.63	438.1	2.1e-131	2-oxoisovalerate dehydrogenase E1 component subunit alpha [EC:1.2.4.4]
CP049783.1_3714	K00167	490.37	495.6	8e-149	2-oxoisovalerate dehydrogenase E1 component subunit beta [EC:1.2.4.4]
CP049783.1_3716	K03801	234.97	249.9	1.5e-74	lipoyl(octanoyl) transferase [EC:2.3.1.181]
CP049783.1_3718	K01258	167.60	406.5	5.7e-122	tripeptide aminopeptidase [EC:3.4.11.4]
CP049783.1_3720	K01515	150.10	214.2	1.1e-63	ADP-ribose diphosphatase [EC:3.6.1.13 3.6.1.-]
CP049783.1_3722	K06384	34.00	132.5	6.1e-39	stage II sporulation protein M
CP049783.1_3723	K03711	142.10	179.2	4.3e-53	Fur family transcriptional regulator, ferric uptake regulator
CP049783.1_3725	K04763	301.10	338.7	2.3e-101	integrase/recombinase XerD
CP049783.1_3726	K03783	165.87	446.2	5e-134	purine-nucleoside phosphorylase [EC:2.4.2.1]

CP049783.1_3727	K16567	120.87	206.6	1.9e-61	exopolysaccharide production protein ExoQ
CP049783.1_3729	K07258	223.10	368.7	1.5e-110	serine-type D-Ala-D-Ala carboxypeptidase (penicillin-binding protein 5/6) [EC:3.4.16.4]
CP049783.1_3730	K06378	115.47	136.1	3.6e-40	stage II sporulation protein AA (anti-sigma F factor antagonist)
CP049783.1_3730	K04749	63.93	93.6	5.1e-27	anti-sigma B factor antagonist
CP049783.1_3731	K06379	153.40	230.5	3.5e-69	stage II sporulation protein AB (anti-sigma F factor) [EC:2.7.11.1]
CP049783.1_3731	K04757	53.50	90.7	3.5e-26	serine/threonine-protein kinase RsbW [EC:2.7.11.1]
CP049783.1_3732	K03091	189.70	243.6	8.9e-73	RNA polymerase sigma-E/F/G factor
CP049783.1_3733	K06403	121.77	236.2	8.7e-71	stage V sporulation protein AA
CP049783.1_3733	K06404	101.63	161.7	3.6e-48	stage V sporulation protein AB
CP049783.1_3735	K06408	458.97	820.3	2.1e-247	stage V sporulation protein AF
CP049783.1_3736	K01586	276.10	546.2	4.4e-164	diaminopimelate decarboxylase [EC:4.1.1.20]
CP049783.1_3738	K00172	151.47	320.1	3.9e-96	pyruvate ferredoxin oxidoreductase gamma subunit [EC:1.2.7.1]
CP049783.1_3739	K00169	292.47	522.4	2.3e-157	pyruvate ferredoxin oxidoreductase alpha subunit [EC:1.2.7.1]
CP049783.1_3741	K00793	67.77	286.8	5.3e-86	riboflavin synthase [EC:2.5.1.9]
CP049783.1_3743	K00794	39.60	249.5	1.4e-74	6,7-dimethyl-8-ribityllumazine synthase [EC:2.5.1.78]
CP049783.1_3744	K05896	40.37	257.5	5.9e-77	segregation and condensation protein A
CP049783.1_3745	K06024	48.53	235.5	2.6e-70	segregation and condensation protein B
CP049783.1_3748	K00686	95.80	293.9	4.3e-88	protein-glutamine gamma-glutamyltransferase [EC:2.3.2.13]
CP049783.1_3749	K07258	223.10	350.1	7e-105	serine-type D-Ala-D-Ala carboxypeptidase (penicillin-binding protein 5/6) [EC:3.4.16.4]
CP049783.1_3750	K06373	144.10	340.8	1.6e-102	spore maturation protein A
CP049783.1_3751	K06374	151.90	267.4	1.7e-80	spore maturation protein B
CP049783.1_3752	K06178	253.10	341.3	3e-102	23S rRNA pseudouridine2605 synthase [EC:5.4.99.22]
CP049783.1_3752	K06183	293.40	308.8	1.6e-92	16S rRNA pseudouridine516 synthase [EC:5.4.99.19]
CP049783.1_3753	K07775	311.73	359.8	2.3e-108	two-component system, OmpR family, response regulator ResD
CP049783.1_3753	K02483	242.00	279.6	1.6e-83	two-component system, OmpR family, response regulator
CP049783.1_3754	K07651	523.70	621.3	6e-187	two-component system, OmpR family, sensor histidine kinase ResE [EC:2.7.13.3]
CP049783.1_3754	K07636	310.93	350.8	3.9e-105	two-component system, OmpR family, phosphate regulon sensor histidine kinase PhoR [EC:2.7.13.3]
CP049783.1_3755	K00058	329.93	534.5	7.7e-161	D-3-phosphoglycerate dehydrogenase / 2-oxoglutarate reductase [EC:1.1.1.95 1.1.1.399]
CP049783.1_3756	K07052	31.30	72.3	1.1e-20	CAAX protease family protein
CP049783.1_3758	K22278	73.27	203.7	1.7e-60	peptidoglycan-N-acetylglucosamine deacetylase [EC:3.5.1.104]
CP049783.1_3760	K16511	72.03	214.9	4.6e-64	adapter protein MecA 1/2
CP049783.1_3761	K24131	48.00	144.1	1.9e-42	protease PrsW [EC:3.4.-.-]
CP049783.1_3763	K06313	156.80	515.1	5.4e-155	spore germination protein
CP049783.1_3765	K00945	42.83	292.7	1.4e-87	CMP/dCMP kinase [EC:2.7.4.25]
CP049783.1_3766	K00655	122.40	180.9	1.3e-53	1-acyl-sn-glycerol-3-phosphate acyltransferase [EC:2.3.1.51]
CP049783.1_3767	K02945	147.87	458.3	1.2e-137	small subunit ribosomal protein S1
CP049783.1_3768	K03977	233.47	643.0	1.1e-193	GTPase
CP049783.1_3769	K08591	64.30	235.7	1.8e-70	acyl phosphate:glycerol-3-phosphate acyltransferase [EC:2.3.1.275]
CP049783.1_3770	K00057	279.33	499.8	3.4e-150	glycerol-3-phosphate dehydrogenase (NAD(P)+) [EC:1.1.1.94]
CP049783.1_3774	K06398	484.37	830.3	1.8e-250	stage IV sporulation protein A
CP049783.1_3775	K02058	208.73	221.1	9.7e-66	simple sugar transport system substrate-binding protein
CP049783.1_3778	K03530	115.33	144.8	1.3e-42	DNA-binding protein HU-beta
CP049783.1_3780	K06285	79.87	131.9	1.4e-39	transcription attenuation protein (tryptophan RNA-binding attenuator protein)
CP049783.1_3782	K00805	56.80	201.7	5.4e-60	heptaprenyl diphosphate synthase component 1 [EC:2.5.1.30]
CP049783.1_3783	K03183	266.63	346.6	5.4e-104	demethylmenaquinone methyltransferase / 2-methoxy-6-polyprenyl-1,4-benzoquinol methylase [EC:2.1.1.163 2.1.1.201]
CP049783.1_3785	K03179	196.07	261.4	3.4e-78	4-hydroxybenzoate polyprenyltransferase [EC:2.5.1.39]
CP049783.1_3786	K03186	92.57	310.1	6.6e-93	flavin prenyltransferase [EC:2.5.1.129]
CP049783.1_3787	K11782	90.73	324.6	2.1e-97	chorismate dehydratase [EC:4.2.1.151]
CP049783.1_3788	K24873	367.43	453.2	1e-136	heptaprenyl diphosphate synthase component 2 [EC:2.5.1.30]
CP049783.1_3789	K00940	149.93	229.1	2.6e-68	nucleoside-diphosphate kinase [EC:2.7.4.6]
CP049783.1_3790	K00575	241.67	298.1	5.1e-89	chemotaxis protein methyltransferase CheR [EC:2.1.1.80]
CP049783.1_3791	K01736	306.20	483.4	4.3e-145	chorismate synthase [EC:4.2.3.5]
CP049783.1_3792	K01735	305.30	495.1	8.1e-149	3-dehydroquinate synthase [EC:4.2.3.4]
CP049783.1_3793	K06208	80.70	191.1	3.4e-57	chorismate mutase [EC:5.4.99.5]
CP049783.1_3795	K01657	465.93	584.4	9.4e-176	anthranilate synthase component I [EC:4.1.3.27]
CP049783.1_3796	K00766	109.93	469.9	2.5e-141	anthranilate phosphoribosyltransferase [EC:2.4.2.18]

CP049783.1_3797	K01609	315.40	385.4	1.5e-115	indole-3-glycerol phosphate synthase [EC:4.1.1.48]
CP049783.1_3798	K01817	210.37	220.6	1.1e-65	phosphoribosylanthranilate isomerase [EC:5.3.1.24]
CP049783.1_3799	K01696	625.47	832.2	2.9e-250	tryptophan synthase beta chain [EC:4.2.1.20]
CP049783.1_3800	K01695	85.97	350.4	3.4e-105	tryptophan synthase alpha chain [EC:4.2.1.20]
CP049783.1_3801	K00817	293.33	421.6	1.8e-126	histidinol-phosphate aminotransferase [EC:2.6.1.9]
CP049783.1_3801	K04517	224.97	290.6	7.8e-87	prephenate dehydrogenase [EC:1.3.1.12]
CP049783.1_3804	K03088	96.50	169.8	2.7e-50	RNA polymerase sigma-70 factor, ECF subfamily
CP049783.1_3808	K01726	184.57	185.3	6.4e-55	gamma-carbonic anhydrase [EC:4.2.1.-]
CP049783.1_3817	K00215	135.50	278.1	3.6e-83	4-hydroxy-tetrahydrodipicolinate reductase [EC:1.17.1.8]
CP049783.1_3818	K01734	76.13	209.3	1.1e-62	methylglyoxal synthase [EC:4.2.3.3]
CP049783.1_3819	K01463	161.63	238.5	3.2e-71	N-acetylglucosamine malate deacetylase 1 [EC:3.5.1.-]
CP049783.1_3820	K00754	262.90	367.3	5.1e-110	L-malate glycosyltransferase [EC:2.4.1.-]
CP049783.1_3821	K00974	272.67	327.5	4.2e-98	tRNA nucleotidyltransferase (CCA-adding enzyme) [EC:2.7.7.72 3.1.3.- 3.1.4.-]
CP049783.1_3822	K03524	154.23	280.3	7.5e-84	BirA family transcriptional regulator, biotin operon repressor / biotin---[acetyl-CoA-carboxylase] ligase [EC:6.3.4.15]
CP049783.1_3823	K00606	82.33	429.0	4.6e-129	3-methyl-2-oxobutanoate hydroxymethyltransferase [EC:2.1.2.11]
CP049783.1_3824	K01918	67.70	401.1	1.5e-120	pantoate--beta-alanine ligase [EC:6.3.2.1]
CP049783.1_3825	K01579	52.57	243.2	6.9e-73	aspartate 1-decarboxylase [EC:4.1.1.11]
CP049783.1_3827	K03722	230.90	660.9	9.2e-199	ATP-dependent DNA helicase DinG [EC:5.6.2.3]
CP049783.1_3827	K02342	101.50	169.7	2.9e-50	DNA polymerase III subunit epsilon [EC:2.7.7.7]
CP049783.1_3827	K09951	46.77	70.6	5.7e-20	CRISPR-associated protein Cas2
CP049783.1_3828	K01926	112.17	294.2	2.5e-88	redox-sensing transcriptional repressor
CP049783.1_3829	K12960	445.40	553.7	2.2e-166	5-methylthioadenosine/S-adenosylhomocysteine deaminase [EC:3.5.4.31 3.5.4.28]
CP049783.1_3834	K00925	190.80	632.4	3.9e-190	acetate kinase [EC:2.7.2.1]
CP049783.1_3835	K01893	373.80	525.2	5.8e-158	asparaginyl-tRNA synthetase [EC:6.1.1.22]
CP049783.1_3836	K02086	54.33	275.3	1.8e-82	DNA replication protein
CP049783.1_3837	K03088	96.50	142.1	6.9e-42	RNA polymerase sigma-70 factor, ECF subfamily
CP049783.1_3842	K08964	126.67	173.1	3.2e-51	methylthioribulose-1-phosphate dehydratase [EC:4.2.1.109]
CP049783.1_3843	K08966	123.57	240.0	7.2e-72	2-hydroxy-3-keto-5-methylthiopentenyl-1-phosphate phosphatase [EC:3.1.3.87]
CP049783.1_3844	K08965	375.83	630.7	2.1e-190	2,3-diketo-5-methylthiopentyl-1-phosphate enolase [EC:5.3.2.5]
CP049783.1_3845	K00286	164.07	311.0	6.4e-93	pyrroline-5-carboxylate reductase [EC:1.5.1.2]
CP049783.1_3846	K00147	552.77	687.0	8.6e-207	glutamate-5-semialdehyde dehydrogenase [EC:1.2.1.41]
CP049783.1_3847	K00931	289.63	480.9	2.2e-144	glutamate 5-kinase [EC:2.7.2.11]
CP049783.1_3848	K08969	544.47	644.3	2.1e-194	L-glutamine---4-(methylsulfanyl)-2-oxobutanoate aminotransferase [EC:2.6.1.117]
CP049783.1_3849	K13566	281.87	287.1	7.4e-86	omega-amidase [EC:3.5.1.3]
CP049783.1_3854	K04761	248.97	265.3	3.2e-79	LysR family transcriptional regulator, hydrogen peroxide-inducible genes activator
CP049783.1_3855	K01077	73.07	327.9	3.8e-98	alkaline phosphatase [EC:3.1.3.1]
CP049783.1_3856	K01012	173.23	205.5	4.6e-61	biotin synthase [EC:2.8.1.6]
CP049783.1_3857	K03150	159.63	490.2	1.5e-147	2-iminoacetate synthase [EC:4.1.99.19]
CP049783.1_3860	K25124	205.07	243.7	2.7e-73	FeS-containing electron transfer protein
CP049783.1_3860	K00196	150.00	152.0	3.7e-45	anaerobic carbon-monoxide dehydrogenase iron sulfur subunit
CP049783.1_3861	K25123	631.60	740.0	2.8e-223	iron hydrogenase HydA2 [EC:1.12.7.-]
CP049783.1_3862	K25124	205.07	234.8	1.4e-70	FeS-containing electron transfer protein
CP049783.1_3863	K22015	873.80	1291.2	0	formate dehydrogenase (hydrogenase) [EC:1.17.98.4 1.17.98.-]
CP049783.1_3866	K01703	454.33	640.1	8.7e-193	3-isopropylmalate/(R)-2-methylmalate dehydratase large subunit [EC:4.2.1.33 4.2.1.35]
CP049783.1_3867	K01704	131.03	287.2	4.8e-86	3-isopropylmalate/(R)-2-methylmalate dehydratase small subunit [EC:4.2.1.33 4.2.1.35]
CP049783.1_3868	K01448	29.13	232.1	2.9e-69	N-acetylmuramoyl-L-alanine amidase [EC:3.5.1.28]
CP049783.1_3869	K01448	29.13	254.9	3.4e-76	N-acetylmuramoyl-L-alanine amidase [EC:3.5.1.28]
CP049783.1_3871	K10773	133.83	272.9	1.7e-81	endonuclease III [EC:3.2.2.- 4.2.99.18]
CP049783.1_3873	K00432	54.07	243.0	1.1e-72	glutathione peroxidase [EC:1.11.1.9]
CP049783.1_3875	K18889	667.30	749.8	8.1e-226	ATP-binding cassette, subfamily B, multidrug efflux pump
CP049783.1_3875	K06147	612.93	634.7	7.3e-191	ATP-binding cassette, subfamily B, bacterial
CP049783.1_3876	K18890	703.17	835.0	1.7e-251	ATP-binding cassette, subfamily B, multidrug efflux pump
CP049783.1_3876	K06147	612.93	681.7	4.4e-205	ATP-binding cassette, subfamily B, bacterial
CP049783.1_3877	K08289	265.07	700.2	1.4e-210	phosphoribosylglycinamide formyltransferase 2 [EC:6.3.1.21]
CP049783.1_3882	K01990	262.37	331.0	3.7e-99	ABC-2 type transport system ATP-binding protein
CP049783.1_3883	K01992	16.23	23.1	5.3e-06	ABC-2 type transport system permease protein

CP049783.1_3885	K02621	874.50	874.9	2.3e-263	topoisomerase IV subunit A [EC:5.6.2.2]
CP049783.1_3888	K03446	476.67	583.9	1e-175	MFS transporter, DHA2 family, multidrug resistance protein
CP049783.1_3890	K06969	218.10	222.7	3.9e-66	23S rRNA (cytosine1962-C5)-methyltransferase [EC:2.1.1.191]
CP049783.1_3892	K03839	94.63	154.3	1.2e-45	flavodoxin I
CP049783.1_3895	K03446	476.67	508.5	7.2e-153	MFS transporter, DHA2 family, multidrug resistance protein
CP049783.1_3896	K03585	261.90	289.5	1.7e-86	membrane fusion protein, multidrug efflux system
CP049783.1_3896	K02005	227.43	235.8	3.4e-70	HlyD family secretion protein
CP049783.1_3900	K02495	261.80	420.9	2.1e-126	oxygen-independent coproporphyrinogen III oxidase [EC:1.3.98.3]
CP049783.1_3904	K26741	173.60	248.4	1.2e-74	PadR family transcriptional regulator, multidrug transcriptional repressor LadR
CP049783.1_3904	K10947	65.70	70.8	5.6e-20	PadR family transcriptional regulator
CP049783.1_3905	K01673	19.40	73.8	3e-21	carbonic anhydrase [EC:4.2.1.1]
CP049783.1_3906	K01299	201.97	525.6	4.6e-158	carboxypeptidase Taq [EC:3.4.17.19]
CP049783.1_3908	K02051	157.20	178.9	7e-53	NitT/TauT family transport system substrate-binding protein
CP049783.1_3910	K02050	145.27	217.9	8.5e-65	NitT/TauT family transport system permease protein
CP049783.1_3912	K16263	263.73	517.4	9.5e-156	amino acid efflux transporter
CP049783.1_3914	K06328	90.60	231.5	1.4e-69	spore coat protein E
CP049783.1_3916	K03555	677.40	1135.8	0	DNA mismatch repair protein MutS
CP049783.1_3917	K03572	418.80	694.1	5e-209	DNA mismatch repair protein MutL
CP049783.1_3918	K15984	88.30	152.0	8.1e-45	16S rRNA (guanine1516-N2)-methyltransferase [EC:2.1.1.242]
CP049783.1_3919	K00791	183.90	397.4	3.5e-119	tRNA dimethylallyltransferase [EC:2.5.1.75]
CP049783.1_3920	K03666	19.87	111.5	9.1e-33	host factor-I protein
CP049783.1_3921	K12555	770.43	942.5	6.6e-284	penicillin-binding protein 2A [EC:2.4.99.28 3.4.16.4]
CP049783.1_3921	K05366	639.07	692.8	2.5e-208	penicillin-binding protein 1A [EC:2.4.99.28 3.4.16.4]
CP049783.1_3922	K09145	75.17	246.9	4.5e-74	uncharacterized protein
CP049783.1_3923	K03748	108.27	116.1	5.8e-34	SanA protein
CP049783.1_3963	K06320	85.60	159.9	2.6e-47	spore maturation protein CgeB
CP049783.1_3982	K03530	115.33	138.7	1e-40	DNA-binding protein HU-beta
CP049783.1_4045	K06314	87.53	139.8	3.4e-41	prespore-specific regulator
CP049783.1_4046	K07171	19.10	74.8	3.1e-21	mRNA interferase MazF [EC:3.1.-.-]
CP049783.1_4047	K07727	44.53	47.8	4.5e-13	putative transcriptional regulator
CP049783.1_4055	K07813	47.80	107.9	1.3e-31	accessory gene regulator B
CP049783.1_4141	K07462	66.97	231.0	9.7e-69	single-stranded-DNA-specific exonuclease [EC:3.1.-.-]
CP049783.1_4163	K21636	237.07	272.0	3.1e-81	ribonucleoside-triphosphate reductase (formate) [EC:1.1.98.6]
CP049783.1_4164	K03647	75.17	124.7	1.2e-36	protein involved in ribonucleotide reduction
CP049783.1_4165	K00525	367.50	463.9	3.1e-139	ribonucleoside-diphosphate reductase alpha chain [EC:1.17.4.1]
CP049783.1_4169	K00560	113.10	283.2	1.2e-84	thymidylate synthase [EC:2.1.1.45]
CP049783.1_4170	K00287	64.20	207.4	1.3e-61	dihydrofolate reductase [EC:1.5.1.3]
CP049783.1_4192	K18640	87.00	155.2	9.8e-46	plasmid segregation protein ParM
CP049783.1_4194	K06413	333.77	443.2	3e-133	stage V sporulation protein K
CP049783.1_4199	K01159	32.43	61.5	3.2e-17	crossover junction endodeoxyribonuclease RuvC [EC:3.1.21.10]
CP049783.1_4201	K06400	148.90	284.4	6.5e-85	site-specific DNA recombinase
CP049783.1_4202	K03665	194.73	545.3	5.4e-164	GTPase
CP049783.1_4204	K03713	103.80	181.9	3e-54	MerR family transcriptional regulator, glutamine synthetase repressor
CP049783.1_4205	K01915	33.97	366.4	1.1e-109	glutamine synthetase [EC:6.3.1.2]
CP049783.1_4206	K14059	167.87	220.0	2.3e-65	integrase
CP049783.1_4211	K03497	54.23	92.6	9.3e-27	ParB family transcriptional regulator, chromosome partitioning protein
CP049783.1_4218	K11144	162.27	269.9	1.2e-80	primosomal protein DnaI
CP049783.1_4220	K02338	60.60	286.7	1.3e-85	DNA polymerase III subunit beta [EC:2.7.7.7]
CP049783.1_4241	K18554	56.63	178.8	4.6e-53	chloramphenicol 3-O phosphotransferase [EC:2.7.1.-]
CP049783.1_4247	K06909	42.80	109.3	7.7e-32	phage terminase large subunit
CP049783.1_4269	K07052	31.30	64.9	1.9e-18	CAAX protease family protein
CP049783.1_4272	K07273	48.73	265.9	1.6e-79	lysozyme
CP049783.1_4292	K07727	44.53	44.6	4.3e-12	putative transcriptional regulator
CP049783.1_4300	K07486	31.47	228.2	6.4e-68	transposase
CP049783.1_4303	K21701	333.73	404.0	3.1e-121	AraC family transcriptional regulator, transcriptional activator for feuABC-ybbA operon
CP049783.1_4306	K19350	392.07	769.9	3.4e-232	lincosamide and streptogramin A transport system ATP-binding/permease protein

CP049783.1_4320	K08164	409.17	454.4	7.8e-137	MFS transporter, DHA1 family, putative efflux transporter
CP049783.1_4323	K19273	177.90	204.5	3.4e-61	streptothricin acetyltransferase [EC:2.3.-.-]
CP049783.1_4329	K18925	114.07	141.8	2.5e-42	paired small multidrug resistance pump
CP049783.1_4330	K18924	121.87	169.6	1e-50	paired small multidrug resistance pump
CP049783.1_4342	K01356	128.77	281.0	3.9e-84	repressor LexA [EC:3.4.21.88]
CP049783.1_4346	K25528	212.23	424.2	4.6e-128	phosphoserine phosphatase [EC:3.1.3.3]
CP049783.1_4346	K07025	112.47	138.2	1.1e-40	putative hydrolase of the HAD superfamily
CP049783.1_4347	K08967	33.60	106.6	4.5e-31	1,2-dihydroxy-3-keto-5-methylthiopentene dioxygenase [EC:1.13.11.53 1.13.11.54]
CP049783.1_4348	K00548	263.53	1388.3	0	5-methyltetrahydrofolate--homocysteine methyltransferase [EC:2.1.1.13]
CP049783.1_4349	K00784	70.63	263.2	1.3e-78	ribonuclease Z [EC:3.1.26.11]
CP049783.1_4350	K02566	258.43	262.2	3.3e-78	5'-nucleotidase [EC:3.1.3.5]
CP049783.1_4351	K10563	196.13	217.4	1.2e-64	formamidopyrimidine-DNA glycosylase [EC:3.2.2.23 4.2.99.18]
CP049783.1_4352	K01151	120.17	285.2	2.5e-85	deoxyribonuclease IV [EC:3.1.21.2]
CP049783.1_4355	K02013	238.30	250.1	1.3e-74	iron complex transport system ATP-binding protein [EC:7.2.2.-]
CP049783.1_4356	K06968	119.80	278.5	3.9e-83	23S rRNA (cytidine2498-2'-O)-methyltransferase [EC:2.1.1.186]
CP049783.1_4369	K10810	208.97	231.2	3.4e-69	thiazole tautomerase (transcriptional regulator TenI) [EC:5.3.99.10]
CP049783.1_4370	K03153	270.03	307.8	3.8e-92	glycine oxidase [EC:1.4.3.19]
CP049783.1_4371	K03154	35.33	67.5	3e-19	sulfur carrier protein
CP049783.1_4372	K03149	188.47	425.5	1.4e-127	thiazole synthase [EC:2.8.1.10]
CP049783.1_4373	K03148	333.67	344.0	3.1e-103	sulfur carrier protein ThiS adenylyltransferase [EC:2.7.7.73]
CP049783.1_4376	K00949	46.80	134.6	1.6e-39	thiamine pyrophosphokinase [EC:2.7.6.2]
CP049783.1_4377	K25307	127.47	195.0	5e-58	low molecular weight protein-tyrosine phosphatase [EC:3.1.3.48]
CP049783.1_4380	K00167	490.37	500.5	2.6e-150	2-oxoisovalerate dehydrogenase E1 component subunit beta [EC:1.2.4.4]
CP049783.1_4381	K00627	488.13	510.5	2.5e-153	pyruvate dehydrogenase E2 component (dihydrolipoyllysine-residue acetyltransferase) [EC:2.3.1.12]
CP049783.1_4381	K09699	429.73	489.8	2.3e-147	2-oxoisovalerate dehydrogenase E2 component (dihydrolipoyl transacylase) [EC:2.3.1.168]
CP049783.1_4382	K00382	465.60	638.2	3.6e-192	dihydrolipoyl dehydrogenase [EC:1.8.1.4]
CP049783.1_4383	K00560	113.10	470.6	1.6e-141	thymidylate synthase [EC:2.1.1.45]
CP049783.1_4384	K00287	64.20	244.9	5.4e-73	dihydrofolate reductase [EC:1.5.1.3]
CP049783.1_4385	K00266	509.03	612.8	2.1e-184	glutamate synthase (NADPH) small chain [EC:1.4.1.13]
CP049783.1_4386	K03169	533.50	921.0	2e-277	DNA topoisomerase III [EC:5.6.2.1]
CP049783.1_4394	K00975	202.07	470.5	1.6e-141	glucose-1-phosphate adenylyltransferase [EC:2.7.7.27]
CP049783.1_4395	K00700	301.20	959.2	1.1e-288	1,4-alpha-glucan branching enzyme [EC:2.4.1.18]
CP049783.1_4396	K00703	126.53	630.0	1.6e-189	starch synthase [EC:2.4.1.21]
CP049783.1_4398	K26141	392.27	416.7	5e-125	Xaa-Arg dipeptidase [EC:3.4.13.4]
CP049783.1_4399	K23059	441.80	509.4	5e-153	arginine/lysine/histidine transporter system substrate-binding protein
CP049783.1_4399	K17077	397.37	470.5	1.3e-141	arginine/lysine/histidine transport system permease protein
CP049783.1_4399	K02029	214.53	300.6	6.3e-90	polar amino acid transport system permease protein
CP049783.1_4399	K02030	76.77	155.4	7.5e-46	polar amino acid transport system substrate-binding protein
CP049783.1_4400	K02028	386.70	467.4	1.7e-140	polar amino acid transport system ATP-binding protein [EC:7.4.2.1]
CP049783.1_4400	K23060	426.27	439.5	2.7e-132	arginine/lysine/histidine transport system ATP-binding protein [EC:7.4.2.1]
CP049783.1_4402	K22278	73.27	276.3	1.5e-82	peptidoglycan-N-acetylglucosamine deacetylase [EC:3.5.1.104]
CP049783.1_4404	K03969	84.50	226.5	2.1e-67	phage shock protein A
CP049783.1_4405	K08989	42.47	75.7	9.8e-22	putative membrane protein
CP049783.1_4410	K07813	47.80	91.4	1.4e-26	accessory gene regulator B
CP049783.1_4415	K03750	141.83	429.6	4.7e-129	molybdopterin molybdotransferase [EC:2.10.1.1]
CP049783.1_4416	K03753	70.57	161.3	1.2e-47	molybdopterin-guanine dinucleotide biosynthesis adapter protein
CP049783.1_4419	K01436	522.37	621.7	1.1e-187	amidohydrolase [EC:3.5.1.-]
CP049783.1_4420	K18578	573.83	1303.1	0	xyloglucan-specific exo-beta-1,4-glucanase [EC:3.2.1.155]
CP049783.1_4421	K24180	175.77	206.5	1.9e-61	malate permease and related proteins
CP049783.1_4423	K00059	269.80	272.3	2.1e-81	3-oxoacyl-[acyl-carrier protein] reductase [EC:1.1.1.100]
CP049783.1_4425	K02529	268.37	319.4	1.5e-95	LacI family transcriptional regulator, galactose operon repressor
CP049783.1_4426	K00852	237.13	365.7	1.6e-109	ribokinase [EC:2.7.1.15]
CP049783.1_4427	K06726	111.10	204.3	3.5e-61	D-ribose pyranase [EC:5.4.99.62]
CP049783.1_4428	K10441	693.10	738.2	2.7e-222	ribose transport system ATP-binding protein [EC:7.5.2.7]
CP049783.1_4429	K10440	333.83	418.5	1.1e-125	ribose transport system permease protein
CP049783.1_4429	K02057	301.83	310.6	5.4e-93	simple sugar transport system permease protein

CP049783.1_4430	K10439	190.90	319.9	1.3e-95	ribose transport system substrate-binding protein
CP049783.1_4431	K25308	264.57	317.4	3.2e-95	ferric hydroxamate/heme transport system substrate-binding protein
CP049783.1_4431	K02016	154.03	169.3	4.4e-50	iron complex transport system substrate-binding protein
CP049783.1_4432	K06147	612.93	674.4	6.9e-203	ATP-binding cassette, subfamily B, bacterial
CP049783.1_4433	K25308	264.57	371.3	1.3e-111	ferric hydroxamate/heme transport system substrate-binding protein
CP049783.1_4434	K25288	404.60	484.3	5.3e-146	ferric hydroxamate/heme transport system permease protein
CP049783.1_4435	K25289	386.53	475.0	4e-143	ferric hydroxamate/heme transport system permease protein
CP049783.1_4437	K13075	114.07	227.6	5.4e-68	N-acyl homoserine lactone hydrolase [EC:3.1.1.81]
CP049783.1_4444	K07456	353.83	603.4	2.3e-181	DNA mismatch repair protein MutS2
CP049783.1_4445	K12243	190.10	247.6	8.8e-74	AraC family transcriptional regulator, transcriptional activator of the genes for pyochelin and ferripyochelin receptors
CP049783.1_4446	K14698	624.83	733.1	1.3e-220	ATP-binding cassette, subfamily B, bacterial IrtA/YbtP [EC:7.-.-.-]
CP049783.1_4447	K14699	597.47	747.5	6.2e-225	ATP-binding cassette, subfamily B, bacterial IrtB/YbtQ [EC:7.-.-.-]
CP049783.1_4448	K08217	225.00	277.4	5.6e-83	MFS transporter, DHA3 family, macrolide efflux protein
CP049783.1_4451	K00016	308.33	422.9	7.9e-127	L-lactate dehydrogenase [EC:1.1.1.27]
CP049783.1_4452	K03294	476.00	596.1	2.4e-179	basic amino acid/polyamine antiporter, APA family
CP049783.1_4458	K01449	48.60	107.4	2.6e-31	cell wall hydrolase
CP049783.1_4459	K06305	74.63	203.5	8.5e-61	spore germination protein Q
CP049783.1_4460	K03699	305.47	543.3	2e-163	magnesium and cobalt exporter, CNNM family
CP049783.1_4461	K01012	173.23	413.6	3.6e-124	biotin synthase [EC:2.8.1.6]
CP049783.1_4462	K00005	325.77	518.7	2.3e-156	glycerol dehydrogenase [EC:1.1.1.6]
CP049783.1_4464	K19689	69.60	456.1	7e-137	aminopeptidase [EC:3.4.11.-]
CP049783.1_4467	K01785	42.83	90.9	3e-26	aldose 1-epimerase [EC:5.1.3.3]
CP049783.1_4469	K01649	498.33	612.6	4.9e-184	2-isopropylmalate synthase [EC:2.3.3.13]
CP049783.1_4470	K01679	639.40	839.9	7.7e-253	fumarate hydratase, class II [EC:4.2.1.2]
CP049783.1_4471	K11105	246.37	628.9	2.5e-189	potassium/hydrogen antiporter
CP049783.1_4474	K23779	92.83	100.2	4.7e-29	XRE family transcriptional regulator, regulator of sulfur utilization
CP049783.1_4475	K15977	60.27	101.4	2.5e-29	putative oxidoreductase
CP049783.1_4476	K07104	96.57	366.2	7.3e-110	catechol 2,3-dioxygenase [EC:1.13.11.2]
CP049783.1_4477	K01425	83.30	382.8	9.8e-115	glutaminase [EC:3.5.1.2]
CP049783.1_4483	K00860	304.20	306.8	7e-92	adenylylsulfate kinase [EC:2.7.1.25]
CP049783.1_4484	K03781	68.70	853.4	4.8e-257	catalase [EC:1.11.1.6]
CP049783.1_4485	K26937	267.43	271.6	3.2e-81	MATE family, multidrug efflux pump
CP049783.1_4488	K02034	256.80	321.2	3.4e-96	peptide/nickel transport system permease protein
CP049783.1_4489	K02033	263.63	373.1	7.1e-112	peptide/nickel transport system permease protein
CP049783.1_4490	K02035	249.67	306.8	9.9e-92	peptide/nickel transport system substrate-binding protein
CP049783.1_4491	K18926	574.20	603.5	7.8e-182	MFS transporter, DHA2 family, lincomycin resistance protein
CP049783.1_4492	K18939	163.23	237.8	2.5e-71	TetR/AcrR family transcriptional regulator, lmrAB and yxaGH operons repressor
CP049783.1_4498	K07155	100.47	422.9	6.5e-127	quercetin 2,3-dioxygenase [EC:1.13.11.24]
CP049783.1_4499	K08168	286.97	480.3	1.7e-144	MFS transporter, DHA2 family, metal-tetracycline-proton antiporter
CP049783.1_4500	K20487	242.97	450.7	1.9e-135	two-component system, OmpR family, lantibiotic biosynthesis sensor histidine kinase NisK/SpaK [EC:2.7.13.3]
CP049783.1_4501	K20488	263.17	303.5	2.3e-91	two-component system, OmpR family, lantibiotic biosynthesis response regulator NisR/SpaR
CP049783.1_4502	K20492	117.57	265.2	1.5e-79	lantibiotic transport system permease protein
CP049783.1_4503	K20491	99.07	217.2	7.5e-65	lantibiotic transport system permease protein
CP049783.1_4504	K20490	293.47	356.2	2e-107	lantibiotic transport system ATP-binding protein
CP049783.1_4504	K01990	262.37	273.8	9e-82	ABC-2 type transport system ATP-binding protein
CP049783.1_4510	K22230	271.20	390.2	3.9e-117	scyllo-inositol 2-dehydrogenase (NADP+) [EC:1.1.1.-]
CP049783.1_4513	K05275	257.70	312.0	2.1e-93	pyridoxine 4-dehydrogenase [EC:1.1.1.65]
CP049783.1_4514	K07407	52.03	177.4	1.8e-52	alpha-galactosidase [EC:3.2.1.22]
CP049783.1_4516	K02529	268.37	297.8	5.2e-89	LacI family transcriptional regulator, galactose operon repressor
CP049783.1_4517	K24995	424.33	461.5	5.2e-139	levoglucosan dehydrogenase [EC:1.1.1.425]
CP049783.1_4518	K09992	76.67	176.6	2.6e-52	uncharacterized protein
CP049783.1_4527	K19587	106.40	137.5	8.6e-41	Rrf2 family transcriptional regulator, repressor of oqxAB
CP049783.1_4528	K15976	141.90	144.4	9e-43	putative NAD(P)H nitroreductase [EC:1.-.-.-]
CP049783.1_4533	K03671	112.43	136.0	6.2e-40	thioredoxin
CP049783.1_4541	K07718	273.17	356.2	1.4e-106	two-component system, sensor histidine kinase YesM [EC:2.7.13.3]
CP049783.1_4542	K07720	156.87	294.0	8.7e-88	two-component system, response regulator YesN

CP049783.1_4543	K17318	176.50	269.0	3.2e-80	putative aldouronate transport system substrate-binding protein
CP049783.1_4544	K17319	220.03	413.6	2.3e-124	putative aldouronate transport system permease protein
CP049783.1_4545	K17320	270.87	317.6	2.3e-95	putative aldouronate transport system permease protein
CP049783.1_4546	K01215	875.10	885.7	3.4e-267	glucan 1,6-alpha-glucosidase [EC:3.2.1.70]
CP049783.1_4546	K01182	875.10	880.5	3.9e-265	oligo-1,6-glucosidase [EC:3.2.1.10]
CP049783.1_4548	K22579	503.63	820.6	6.9e-247	rifampicin phosphotransferase [EC:2.7.9.6]
CP049783.1_4550	K20488	263.17	266.1	6.3e-80	two-component system, OmpR family, lantibiotic biosynthesis response regulator NisR/SpaR
CP049783.1_4550	K02483	242.00	245.7	3.1e-73	two-component system, OmpR family, response regulator
CP049783.1_4554	K07481	59.83	69.5	1.2e-19	transposase, IS5 family
CP049783.1_4555	K07481	59.83	85.6	1.6e-24	transposase, IS5 family
CP049783.1_4562	K16124	6234.30	6822.4	0	tyrocidine synthetase III
CP049783.1_4562	K03367	499.93	504.8	7.5e-152	D-alanine--poly(phosphoribitol) ligase subunit 1 [EC:6.1.1.13]
CP049783.1_4563	K15395	3208.50	3677.6	0	hybrid polyketide synthase / nonribosomal peptide synthetase FtdB
CP049783.1_4577	K03292	344.60	430.2	3e-129	glycoside/pentoside/hexuronide:cation symporter, GPH family
CP049783.1_4578	K02529	268.37	318.7	2.4e-95	LacI family transcriptional regulator, galactose operon repressor
CP049783.1_4579	K01193	187.77	508.7	7.1e-153	beta-fructofuranosidase [EC:3.2.1.26]
CP049783.1_4580	K02532	177.13	580.8	3.7e-175	MFS transporter, OHS family, lactose permease
CP049783.1_4581	K03484	339.57	405.0	9.8e-122	LacI family transcriptional regulator, sucrose operon repressor
CP049783.1_4581	K02529	268.37	304.8	4e-91	LacI family transcriptional regulator, galactose operon repressor
CP049783.1_4584	K02477	121.47	135.0	1.1e-39	two-component system, LytTR family, response regulator
CP049783.1_4585	K19575	133.80	183.0	3e-54	MerR family transcriptional regulator, activator of bmr gene
CP049783.1_4587	K01216	265.47	409.6	5.3e-123	licheninase [EC:3.2.1.73]
CP049783.1_4588	K15921	357.33	745.6	1.6e-224	arabinoxylan arabinofuranohydrolase [EC:3.2.1.55]
CP049783.1_4590	K01951	100.47	115.9	6.6e-34	GMP synthase (glutamine-hydrolysing) [EC:6.3.5.2]
CP049783.1_4592	K18888	717.83	865.8	9e-261	ATP-binding cassette, subfamily B, multidrug efflux pump
CP049783.1_4593	K18887	708.60	815.6	1.3e-245	ATP-binding cassette, subfamily B, multidrug efflux pump
CP049783.1_4598	K06296	248.90	290.1	8.7e-87	spore germination protein KB
CP049783.1_4601	K06295	452.20	694.4	7.1e-209	spore germination protein KA
CP049783.1_4602	K01426	303.90	315.6	3e-94	amidase [EC:3.5.1.4]
CP049783.1_4603	K22441	138.10	227.8	5.4e-68	diamine N-acetyltransferase [EC:2.3.1.57]
CP049783.1_4605	K07149	76.20	163.2	2e-48	uncharacterized protein
CP049783.1_4609	K01686	137.10	164.2	1.3e-48	mannonate dehydratase [EC:4.2.1.8]
CP049783.1_4611	K01686	137.10	481.6	8.5e-145	mannonate dehydratase [EC:4.2.1.8]
CP049783.1_4614	K08174	208.07	382.9	3.8e-115	MFS transporter, FHS family, glucose/mannose:H+ symporter
CP049783.1_4617	K01091	112.83	129.1	7.9e-38	phosphoglycolate phosphatase [EC:3.1.3.18]
CP049783.1_4619	K09702	62.87	186.9	1.1e-55	uncharacterized protein
CP049783.1_4620	K19273	177.90	239.3	7.1e-72	streptothricin acetyltransferase [EC:2.3.-.-]
CP049783.1_4621	K01921	226.63	396.0	1.1e-118	D-alanine-D-alanine ligase [EC:6.3.2.4]
CP049783.1_4627	K19265	304.10	547.9	7.4e-165	L-glyceraldehyde 3-phosphate reductase [EC:1.1.1.-]
CP049783.1_4628	K02483	242.00	248.0	6.4e-74	two-component system, OmpR family, response regulator
CP049783.1_4631	K02529	268.37	297.4	7.3e-89	LacI family transcriptional regulator, galactose operon repressor
CP049783.1_4633	K22278	73.27	220.4	1.4e-65	peptidoglycan-N-acetylglucosamine deacetylase [EC:3.5.1.104]
CP049783.1_4634	K08168	286.97	500.8	1e-150	MFS transporter, DHA2 family, metal-tetracycline-proton antiporter
CP049783.1_4647	K10119	296.93	332.0	1.2e-99	raffinose/stachyose/melibiose transport system permease protein
CP049783.1_4648	K02025	276.90	289.1	1.4e-86	multiple sugar transport system permease protein
CP049783.1_4649	K10117	246.87	266.3	2.6e-79	raffinose/stachyose/melibiose transport system substrate-binding protein
CP049783.1_4650	K07720	156.87	333.0	1.3e-99	two-component system, response regulator YesN
CP049783.1_4651	K07718	273.17	455.2	1.7e-136	two-component system, sensor histidine kinase YesM [EC:2.7.13.3]
CP049783.1_4652	K02529	268.37	302.8	1.6e-90	LacI family transcriptional regulator, galactose operon repressor
CP049783.1_4653	K07407	52.03	190.2	2.5e-56	alpha-galactosidase [EC:3.2.1.22]
CP049783.1_4655	K07025	112.47	122.4	7e-36	putative hydrolase of the HAD superfamily
CP049783.1_4660	K06295	452.20	714.9	4.5e-215	spore germination protein KA
CP049783.1_4661	K06297	281.23	310.8	5e-93	spore germination protein KC
CP049783.1_4662	K06296	248.90	287.8	4.4e-86	spore germination protein KB
CP049783.1_4665	K07092	58.83	68.1	1.9e-19	uncharacterized protein
CP049783.1_4666	K04085	48.00	92.3	6.6e-27	tRNA 2-thiouridine synthesizing protein A [EC:2.8.1.-]

CP049783.1_4671	K19689	69.60	437.0	4e-131	aminopeptidase [EC:3.4.11.-]
CP049783.1_4674	K01303	141.47	147.5	1.7e-43	acylaminoacyl-peptidase [EC:3.4.19.1]
CP049783.1_4676	K24117	207.53	228.8	9.7e-69	S-(2-succino)cysteine N-acetyltransferase [EC:2.3.1.-]
CP049783.1_4677	K02217	147.03	214.4	7e-64	ferritin [EC:1.16.3.2]
CP049783.1_4682	K00702	869.57	1180.6	0	cellobiose phosphorylase [EC:2.4.1.20]
CP049783.1_4685	K00836	439.63	581.7	5.2e-175	diaminobutyrate-2-oxoglutarate transaminase [EC:2.6.1.76]
CP049783.1_4688	K03406	65.50	319.5	1.5e-95	methyl-accepting chemotaxis protein
CP049783.1_4689	K06888	62.67	434.8	3.2e-130	uncharacterized protein
CP049783.1_4690	K06888	62.67	550.3	4.2e-165	uncharacterized protein
CP049783.1_4695	K01265	141.83	296.8	7.6e-89	methionyl aminopeptidase [EC:3.4.11.18]
CP049783.1_4698	K03088	96.50	122.3	7.3e-36	RNA polymerase sigma-70 factor, ECF subfamily
CP049783.1_4699	K01358	76.73	350.5	2.8e-105	ATP-dependent Clp protease, protease subunit [EC:3.4.21.92]
CP049783.1_4708	K11249	109.63	231.6	1.7e-69	cysteine/O-acetylserine efflux protein
CP049783.1_4709	K06295	452.20	664.6	7.3e-200	spore germination protein KA
CP049783.1_4710	K06297	281.23	337.6	4e-101	spore germination protein KC
CP049783.1_4712	K06296	248.90	291.0	4.6e-87	spore germination protein KB
CP049783.1_4713	K06182	241.13	316.9	5.4e-95	23S rRNA pseudouridine2604 synthase [EC:5.4.99.21]
CP049783.1_4717	K04750	56.87	148.4	8.8e-44	PhnB protein
CP049783.1_4719	K25289	386.53	407.8	1e-122	ferric hydroxamate/heme transport system permease protein
CP049783.1_4720	K25288	404.60	433.1	2e-130	ferric hydroxamate/heme transport system permease protein
CP049783.1_4721	K25308	264.57	296.5	7.3e-89	ferric hydroxamate/heme transport system substrate-binding protein
CP049783.1_4727	K12251	343.87	478.8	5.8e-144	N-carbamoylputrescine amidase [EC:3.5.1.53]
CP049783.1_4728	K10536	118.97	492.0	6.9e-148	agmatine deiminase [EC:3.5.3.12]
CP049783.1_4729	K07095	94.43	125.9	4.3e-37	uncharacterized protein
CP049783.1_4730	K00661	129.97	274.1	3.5e-82	maltose O-acetyltransferase [EC:2.3.1.79]
CP049783.1_4731	K01223	479.40	775.3	9.5e-234	6-phospho-beta-glucosidase [EC:3.2.1.86]
CP049783.1_4733	K02935	22.77	38.9	2.7e-10	large subunit ribosomal protein L7/L12
CP049783.1_4734	K03976	99.40	199.5	2.8e-59	Cys-tRNA(Pro)/Cys-tRNA(Cys) deacylase [EC:3.1.1.-]
CP049783.1_4735	K01569	93.33	625.0	1.7e-188	oxalate decarboxylase [EC:4.1.1.2]
CP049783.1_4736	K19267	112.87	174.6	1.4e-51	NAD(P)H dehydrogenase (quinone) [EC:1.6.5.2]
CP049783.1_4739	K18189	91.27	179.5	2.6e-53	translational activator of cytochrome c oxidase 1
CP049783.1_4741	K01881	163.60	532.7	4e-160	prolyl-tRNA synthetase [EC:6.1.1.15]
CP049783.1_4744	K07407	52.03	297.5	8.2e-89	alpha-galactosidase [EC:3.2.1.22]
CP049783.1_4745	K07146	171.23	337.1	4.9e-101	UPF0176 protein
CP049783.1_4746	K24180	175.77	204.4	8.6e-61	malate permease and related proteins
CP049783.1_4747	K02013	238.30	239.9	1.7e-71	iron complex transport system ATP-binding protein [EC:7.2.2.-]
CP049783.1_4748	K01569	93.33	428.8	6.3e-129	oxalate decarboxylase [EC:4.1.1.2]
CP049783.1_4751	K00971	203.67	238.5	4.5e-71	mannose-1-phosphate guanylyltransferase [EC:2.7.7.13]
CP049783.1_4752	K06987	88.07	117.5	2.3e-34	uncharacterized protein
CP049783.1_4755	K18940	370.43	453.6	3.2e-136	two-component system, OmpR family, sensor histidine kinase ArlS [EC:2.7.13.3]
CP049783.1_4756	K18941	299.00	331.1	8.4e-100	two-component system, OmpR family, response regulator ArlR
CP049783.1_4756	K02483	242.00	267.8	6.4e-80	two-component system, OmpR family, response regulator
CP049783.1_4757	K19302	106.60	149.3	4.9e-44	undecaprenyl-diphosphatase [EC:3.6.1.27]
CP049783.1_4758	K19302	106.60	148.3	1e-43	undecaprenyl-diphosphatase [EC:3.6.1.27]
CP049783.1_4761	K03217	121.07	213.9	1e-63	YidC/Oxa1 family membrane protein insertase
CP049783.1_4763	K03517	105.40	390.6	3e-117	quinolinate synthase [EC:2.5.1.72]
CP049783.1_4764	K04487	287.40	401.2	1.9e-120	cysteine desulfurase [EC:2.8.1.7]
CP049783.1_4765	K07105	96.27	211.0	4.4e-63	uncharacterized protein
CP049783.1_4766	K03624	159.57	184.2	8.9e-55	transcription elongation factor GreA
CP049783.1_4772	K16118	1359.93	1368.8	0	pristinamycin I synthase 3 and 4
CP049783.1_4773	K16118	1359.93	1368.2	0	pristinamycin I synthase 3 and 4
CP049783.1_4774	K16118	1359.93	1371.7	0	pristinamycin I synthase 3 and 4
CP049783.1_4774	K03367	499.93	513.0	2.4e-154	D-alanine--poly(phosphoribitol) ligase subunit 1 [EC:6.1.1.13]
CP049783.1_4775	K16095	6976.90	7938.3	0	bacitracin synthase 3
CP049783.1_4775	K03367	499.93	517.9	7.9e-156	D-alanine--poly(phosphoribitol) ligase subunit 1 [EC:6.1.1.13]
CP049783.1_4778	K12242	248.83	249.2	9.1e-75	pyochelin biosynthesis protein PchC

CP049783.1_4779	K02446	462.67	474.9	6.9e-143	fructose-1,6-bisphosphatase II [EC:3.1.3.11]
CP049783.1_4780	K09773	264.67	334.1	2.6e-100	[pyruvate, water dikinase]-phosphate phosphotransferase / [pyruvate, water dikinase] kinase [EC:2.7.4.28 2.7.11.33]
CP049783.1_4781	K23774	112.50	234.7	2.9e-70	DeoR family transcriptional regulator, catabolite repression regulator
CP049783.1_4782	K02004	52.33	114.3	1.5e-33	putative ABC transport system permease protein
CP049783.1_4783	K02003	292.97	354.4	2.5e-106	putative ABC transport system ATP-binding protein
CP049783.1_4784	K02005	227.43	288.2	4.1e-86	HlyD family secretion protein
CP049783.1_4787	K07443	96.07	147.1	1.8e-43	methylated-DNA-protein-cysteine methyltransferase related protein
CP049783.1_4792	K18349	294.43	337.8	8.4e-102	two-component system, OmpR family, response regulator VanR
CP049783.1_4792	K02483	242.00	248.8	3.7e-74	two-component system, OmpR family, response regulator
CP049783.1_4793	K18350	230.63	285.1	3e-85	two-component system, OmpR family, sensor histidine kinase VanS [EC:2.7.13.3]
CP049783.1_4794	K00782	116.30	157.8	1.4e-46	L-lactate dehydrogenase complex protein LldG
CP049783.1_4796	K18929	437.67	537.8	1.1e-161	L-lactate dehydrogenase complex protein LldF
CP049783.1_4797	K18928	254.60	385.8	3.4e-116	L-lactate dehydrogenase complex protein LldE
CP049783.1_4798	K02430	242.13	298.1	1.5e-89	DeoR family transcriptional regulator, L-fucose operon activator
CP049783.1_4798	K03436	282.67	287.1	3.8e-86	DeoR family transcriptional regulator, fructose operon transcriptional repressor
CP049783.1_4799	K00848	300.13	723.9	4.8e-218	rhamnulokinase [EC:2.7.1.5]
CP049783.1_4805	K04083	58.73	335.2	1.3e-100	molecular chaperone Hsp33
CP049783.1_4809	K05593	117.83	388.2	1.9e-116	aminoglycoside 6-adenylyltransferase [EC:2.7.7.-]
CP049783.1_4815	K09815	268.47	411.6	1.9e-123	zinc transport system substrate-binding protein
CP049783.1_4816	K09816	273.47	293.3	7.2e-88	zinc transport system permease protein
CP049783.1_4818	K02016	154.03	194.1	1.3e-57	iron complex transport system substrate-binding protein
CP049783.1_4819	K02013	238.30	350.4	4.8e-105	iron complex transport system ATP-binding protein [EC:7.2.2.-]
CP049783.1_4820	K02015	340.73	407.0	4.1e-122	iron complex transport system permease protein
CP049783.1_4820	K25027	379.47	403.6	2.7e-121	cobalamin transport system permease protein
CP049783.1_4822	K07089	69.23	264.0	7.1e-79	uncharacterized protein
CP049783.1_4823	K08986	64.53	258.3	2.4e-77	putative membrane protein
CP049783.1_4827	K07481	59.83	75.0	2.6e-21	transposase, IS5 family
CP049783.1_4828	K20276	24.33	35.0	7.7e-10	large repetitive protein
CP049783.1_4829	K20276	24.33	52.1	5.3e-15	large repetitive protein
CP049783.1_4830	K02909	21.83	89.9	5.1e-26	large subunit ribosomal protein L31
CP049783.1_4831	K02913	23.23	50.9	4.3e-14	large subunit ribosomal protein L33
CP049783.1_4833	K08168	286.97	520.9	8.1e-157	MFS transporter, DHA2 family, metal-tetracycline-proton antiporter
CP049783.1_4845	K17217	607.37	645.2	7.6e-195	cystathionine gamma-lyase / homocysteine desulfhydrase [EC:4.4.1.1 4.4.1.2]
CP049783.1_4845	K01760	493.17	559.3	1.9e-168	cysteine-S-conjugate beta-lyase [EC:4.4.1.13]
CP049783.1_4845	K01739	420.37	434.1	2.5e-130	cystathionine gamma-synthase [EC:2.5.1.48]
CP049783.1_4846	K17216	447.97	501.6	2e-151	cystathionine beta-synthase (O-acetyl-L-serine) [EC:2.5.1.134]
CP049783.1_4849	K03722	230.90	393.0	1e-117	ATP-dependent DNA helicase DinG [EC:5.6.2.3]
CP049783.1_4850	K25308	264.57	265.6	1.7e-79	ferric hydroxamate/heme transport system substrate-binding protein
CP049783.1_4852	K18581	134.60	431.9	1.2e-129	unsaturated chondroitin disaccharide hydrolase [EC:3.2.1.180]
CP049783.1_4856	K02954	26.53	131.7	8.8e-39	small subunit ribosomal protein S14
CP049783.1_4857	K02032	420.67	516.3	5.2e-155	peptide/nickel transport system ATP-binding protein
CP049783.1_4858	K02031	412.13	501.1	1.5e-150	peptide/nickel transport system ATP-binding protein
CP049783.1_4861	K02033	263.63	385.5	1.3e-115	peptide/nickel transport system permease protein
CP049783.1_4862	K02035	249.67	327.2	6.9e-98	peptide/nickel transport system substrate-binding protein
CP049783.1_4866	K21613	481.13	642.9	4.2e-194	N-acetylcysteine deacetylase [EC:3.5.1.-]
CP049783.1_4868	K02028	386.70	437.1	2.7e-131	polar amino acid transport system ATP-binding protein [EC:7.4.2.1]
CP049783.1_4868	K16960	403.47	432.6	1e-130	L-cystine transport system ATP-binding protein [EC:7.4.2.1]
CP049783.1_4868	K10010	381.10	397.9	4.5e-120	L-cystine transport system ATP-binding protein [EC:7.4.2.1]
CP049783.1_4869	K16959	256.10	313.1	3e-94	L-cystine transport system permease protein
CP049783.1_4869	K02029	214.53	223.6	1.4e-66	polar amino acid transport system permease protein
CP049783.1_4870	K16958	278.73	322.9	2.9e-97	L-cystine transport system permease protein
CP049783.1_4870	K02029	214.53	252.6	2.2e-75	polar amino acid transport system permease protein
CP049783.1_4871	K16957	256.73	367.2	2.1e-110	L-cystine transport system substrate-binding protein
CP049783.1_4871	K02030	76.77	120.1	4.1e-35	polar amino acid transport system substrate-binding protein
CP049783.1_4873	K00299	91.57	176.8	2.3e-52	FMN reductase [EC:1.5.1.38]
CP049783.1_4874	K24116	523.57	606.0	1.2e-182	N-acetyl-S-(2-succino)cysteine monooxygenase [EC:1.14.-.-]
CP049783.1_4883	K01421	140.40	491.9	1.3e-147	putative membrane protein
CP049783.1_4885	K00633	275.93	309.0	1.7e-92	galactoside O-acetyltransferase [EC:2.3.1.18]
CP049783.1_4885	K00661	129.97	266.2	9.3e-80	maltose O-acetyltransferase [EC:2.3.1.79]
CP049783.1_4890	K09928	51.63	59.1	1.5e-16	uncharacterized protein
CP049783.1_4891	K00688	78.53	1122.6	0	glycogen phosphorylase [EC:2.4.1.1]
CP049783.1_4892	K00975	202.07	272.7	1.6e-81	glucose-1-phosphate adenylyltransferase [EC:2.7.7.27]
CP049783.1_4893	K00975	202.07	487.9	8.7e-147	glucose-1-phosphate adenylyltransferase [EC:2.7.7.27]
CP049783.1_4896	K07720	156.87	275.2	4.2e-82	two-component system, response regulator YesN

CP049783.1_4897	K07718	273.17	395.7	1.7e-118	two-component system, sensor histidine kinase YesM [EC:2.7.13.3]
CP049783.1_4898	K17318	176.50	280.2	1.3e-83	putative aldouronate transport system substrate-binding protein
CP049783.1_4899	K17320	270.87	370.7	1.5e-111	putative aldouronate transport system permease protein
CP049783.1_4900	K17319	220.03	421.0	1.4e-126	putative aldouronate transport system permease protein
CP049783.1_4904	K03827	132.00	189.6	3.4e-56	putative acetyltransferase [EC:2.3.1.-]
CP049783.1_4905	K01685	552.00	758.3	1.1e-228	altronate hydrolase [EC:4.2.1.7]
CP049783.1_4906	K00041	288.63	700.1	5.5e-211	tagaturonate reductase [EC:1.1.1.58]
CP049783.1_4907	K00156	698.60	805.8	9.7e-243	pyruvate dehydrogenase (quinone) [EC:1.2.5.1]
CP049783.1_4908	K26609	372.43	523.9	4.8e-158	LacI family transcriptional regulator, galacturonate utilization repressor
CP049783.1_4908	K02529	268.37	308.6	2.8e-92	LacI family transcriptional regulator, galactose operon repressor
CP049783.1_4909	K03292	344.60	444.3	1.6e-133	glycoside/pentoside/hexuronide:cation symporter, GPH family
CP049783.1_4910	K01812	195.40	673.7	6.6e-203	glucuronate isomerase [EC:5.3.1.12]
CP049783.1_4912	K01575	93.43	347.3	1.7e-104	acetolactate decarboxylase [EC:4.1.1.5]
CP049783.1_4914	K13275	341.67	469.3	1.9e-141	major intracellular serine protease [EC:3.4.21.-]
CP049783.1_4915	K26937	267.43	298.7	1.9e-89	MATE family, multidrug efflux pump
CP049783.1_4916	K11210	123.53	238.6	7.5e-72	metallothiol transferase [EC:2.5.1.-]
CP049783.1_4918	K01776	98.97	281.5	3.4e-84	glutamate racemase [EC:5.1.1.3]
CP049783.1_4919	K01118	93.70	198.2	6.6e-59	FMN-dependent NADH-azoreductase [EC:1.7.1.17]
CP049783.1_4922	K07404	130.17	435.6	8.4e-131	6-phosphogluconolactonase [EC:3.1.1.31]
CP049783.1_4923	K04063	57.63	73.0	8.9e-21	lipoyl-dependent peroxiredoxin [EC:1.11.1.28]
CP049783.1_4924	K08369	358.47	493.1	2.6e-148	MFS transporter, putative metabolite:H+ symporter
CP049783.1_4926	K00033	157.37	346.1	1.3e-103	6-phosphogluconate dehydrogenase [EC:1.1.1.44 1.1.1.343]
CP049783.1_4927	K00036	396.33	611.2	8.7e-184	glucose-6-phosphate 1-dehydrogenase [EC:1.1.1.49 1.1.1.363]
CP049783.1_4931	K07078	120.47	314.6	2.5e-94	uncharacterized protein
CP049783.1_4934	K07095	94.43	153.4	1.6e-45	uncharacterized protein
CP049783.1_4936	K04074	47.10	67.1	5.4e-19	cell division initiation protein
CP049783.1_4937	K06204	35.73	100.9	3.9e-29	RNA polymerase-binding transcription factor
CP049783.1_4938	K14273	314.30	447.7	8.6e-135	D-xylose 1-dehydrogenase (NADP+, D-xylono-1,5-lactone-forming) [EC:1.1.1.179]
CP049783.1_4942	K03579	572.67	1067.7	0	ATP-dependent RNA helicase HrpB [EC:5.6.2.6]
CP049783.1_4943	K01885	298.30	389.3	1.5e-116	glutamyl-tRNA synthetase [EC:6.1.1.17]
CP049783.1_4943	K01894	315.00	317.6	3.4e-95	glutamyl-Q tRNA(Asp) synthetase [EC:6.1.1.-]
CP049783.1_4946	K06376	38.70	43.1	1.1e-11	stage 0 sporulation regulatory protein
CP049783.1_4953	K07497	13.93	115.7	9.6e-34	putative transposase
CP049783.1_4961	K14956	27.87	37.0	1.3e-09	ESAT-6 family protein
CP049783.1_4964	K03466	191.80	234.6	7.7e-70	DNA segregation ATPase FtsK/SpoIIIE, S-DNA-T family
CP049783.1_4970	K06949	90.53	345.2	1.6e-103	ribosome biogenesis GTPase / thiamine phosphate phosphatase [EC:3.6.1.- 3.1.3.100]
CP049783.1_4972	K26960	268.90	472.8	1.2e-142	bacillithiol disulfide reductase [EC:1.8.1.-]
CP049783.1_4976	K18889	667.30	781.6	1.9e-235	ATP-binding cassette, subfamily B, multidrug efflux pump
CP049783.1_4977	K18890	703.17	819.6	7.8e-247	ATP-binding cassette, subfamily B, multidrug efflux pump
CP049783.1_4979	K01887	107.30	567.1	2.1e-170	arginyl-tRNA synthetase [EC:6.1.1.19]
CP049783.1_4981	K03638	158.63	256.0	1.5e-76	molybdopterin adenylyltransferase [EC:2.7.7.75]
CP049783.1_4982	K00763	105.10	587.7	1.3e-176	nicotinate phosphoribosyltransferase [EC:6.3.4.21]
CP049783.1_4983	K08281	104.80	110.6	2.9e-32	nicotinamidase/pyrazinamidase [EC:3.5.1.19 3.5.1.-]
CP049783.1_4987	K06168	451.40	658.7	2.1e-198	tRNA-2-methylthio-N6-dimethylallyladenosine synthase [EC:2.8.4.3]
CP049783.1_4988	K15024	265.87	304.1	1.4e-91	putative phosphotransacetylase [EC:2.3.1.8]
CP049783.1_4990	K01273	59.53	229.3	2.1e-68	membrane dipeptidase [EC:3.4.13.19]
CP049783.1_4991	K06416	55.20	160.8	2.2e-48	stage V sporulation protein S
CP049783.1_4992	K09769	137.70	343.1	3.7e-103	2',3'-cyclic-nucleotide 2'-phosphodiesterase [EC:3.1.4.16]
CP049783.1_4993	K18682	109.03	814.6	1.1e-245	ribonucrease Y [EC:3.1.-.-]
CP049783.1_4994	K03565	36.33	162.9	3.7e-48	regulatory protein
CP049783.1_4995	K03553	190.37	691.2	6.1e-208	recombination protein RecA
CP049783.1_4996	K03743	24.90	184.5	7.7e-55	nicotinamide-nucleotide amidase [EC:3.5.1.42]
CP049783.1_4997	K00995	120.80	140.8	2.1e-41	CDP-diacylglycerol---glycerol-3-phosphate 3-phosphatidyltransferase [EC:2.7.8.5]
CP049783.1_4999	K09767	72.13	230.8	4.6e-69	cyclic-di-GMP-binding protein
CP049783.1_5001	K15539	70.70	190.3	2.4e-56	cytoskeleton protein RodZ
CP049783.1_5004	K00059	269.80	300.0	8.4e-90	3-oxoacyl-[acyl-carrier protein] reductase [EC:1.1.1.100]
CP049783.1_5007	K27245	246.57	349.8	7e-105	peptidoglycan lytic transglycosylase [EC:4.2.2.29]
CP049783.1_5007	K01449	48.60	197.5	8e-59	cell wall hydrolase
CP049783.1_5008	K03466	191.80	799.2	2.6e-240	DNA segregation ATPase FtsK/SpoIIIE, S-DNA-T family
CP049783.1_5011	K12574	123.37	788.6	1.4e-237	ribonuclease J [EC:3.1.-.-]
CP049783.1_5012	K01714	259.73	369.0	1.2e-110	4-hydroxy-tetrahydrodipicolinate synthase [EC:4.3.3.7]
CP049783.1_5013	K00928	394.00	411.5	1.5e-123	aspartate kinase [EC:2.7.2.4]
CP049783.1_5014	K00133	214.73	495.7	5.4e-149	aspartate-semialdehyde dehydrogenase [EC:1.2.1.11]
CP049783.1_5015	K06411	197.03	322.2	2.9e-97	dipicolinate synthase subunit B
CP049783.1_5016	K06410	98.30	420.5	1.2e-126	dipicolinate synthase subunit A
CP049783.1_5017	K01520	47.27	189.7	2.2e-56	dUTP diphosphatase [EC:3.6.1.23]
CP049783.1_5018	K07263	220.23	258.8	4.3e-77	zinc protease [EC:3.4.24.-]

CP049783.1_5019	K22278	73.27	180.7	1.6e-53	peptidoglycan-N-acetylglucosamine deacetylase [EC:3.5.1.104]
CP049783.1_5020	K00962	326.70	1038.9	4.5e-313	polyribonucleotide nucleotidyltransferase [EC:2.7.7.8]
CP049783.1_5021	K02956	27.43	130.0	4.9e-38	small subunit ribosomal protein S15
CP049783.1_5022	K11753	132.60	381.0	1.5e-114	riboflavin kinase / FMN adenylyltransferase [EC:2.7.1.26 2.7.7.2]
CP049783.1_5023	K03177	194.47	338.8	1.8e-101	tRNA pseudouridine55 synthase [EC:5.4.99.25]
CP049783.1_5024	K06881	205.10	354.0	3.3e-106	bifunctional oligoribonuclease and PAP phosphatase NrnA [EC:3.1.3.7 3.1.13.3]
CP049783.1_5025	K02834	26.40	164.2	1e-48	ribosome-binding factor A
CP049783.1_5026	K02519	314.93	1004.6	1.2e-302	translation initiation factor IF-2
CP049783.1_5028	K07742	20.17	94.1	2.8e-27	uncharacterized protein
CP049783.1_5029	K02600	35.63	566.4	1.9e-170	transcription termination/antitermination protein NusA
CP049783.1_5030	K09748	22.77	167.0	1.8e-49	ribosome maturation factor RimP
CP049783.1_5031	K03763	681.33	1991.0	0	DNA polymerase III subunit alpha, Gram-positive type [EC:2.7.7.7]
CP049783.1_5031	K02342	101.50	206.8	1.6e-61	DNA polymerase III subunit epsilon [EC:2.7.7.7]
CP049783.1_5031	K09951	46.77	88.7	1.7e-25	CRISPR-associated protein Cas2
CP049783.1_5032	K01881	163.60	466.4	4.8e-140	prolyl-tRNA synthetase [EC:6.1.1.15]
CP049783.1_5033	K11749	79.03	371.6	2.9e-111	regulator of sigma E protease [EC:3.4.24.-]
CP049783.1_5034	K00099	227.13	608.4	5e-183	1-deoxy-D-xylulose-5-phosphate reductoisomerase [EC:1.1.1.267]
CP049783.1_5035	K00981	43.57	190.4	2e-56	phosphatidate cytidylyltransferase [EC:2.7.7.41]
CP049783.1_5036	K00806	330.87	366.7	2.9e-110	undecaprenyl diphosphate synthase [EC:2.5.1.31]
CP049783.1_5037	K02838	25.83	250.9	5.1e-75	ribosome recycling factor
CP049783.1_5038	K09903	92.77	422.9	4e-127	uridylate kinase [EC:2.7.4.22]
CP049783.1_5039	K02357	112.87	166.7	2.7e-49	elongation factor Ts
CP049783.1_5040	K02967	165.67	431.5	1.7e-129	small subunit ribosomal protein S2
CP049783.1_5041	K07082	42.70	45.6	1.2e-12	peptidoglycan lytic transglycosylase G [EC:4.2.2.29]
CP049783.1_5044	K09749	102.50	427.4	2.8e-128	uncharacterized protein
CP049783.1_5045	K02405	185.07	309.5	1.5e-92	RNA polymerase sigma factor FliA
CP049783.1_5047	K03411	62.07	179.0	3.6e-53	chemotaxis protein CheD [EC:3.5.1.44]
CP049783.1_5048	K03410	111.67	211.4	5e-63	chemotaxis protein CheC
CP049783.1_5049	K03408	105.87	162.4	4.7e-48	purine-binding chemotaxis protein CheW
CP049783.1_5050	K03407	558.90	764.7	6.1e-230	two-component system, chemotaxis family, sensor kinase CheA [EC:2.7.13.3]
CP049783.1_5051	K03412	203.57	441.2	2.6e-132	two-component system, chemotaxis family, protein-glutamate methylesterase/glutaminase [EC:3.1.1.61 3.5.1.44]
CP049783.1_5052	K04562	208.37	333.1	7e-100	flagellar biosynthesis protein FlhG
CP049783.1_5053	K02404	169.97	376.3	1.3e-112	flagellar biosynthesis protein FlhF
CP049783.1_5054	K02400	726.17	981.4	7.7e-296	flagellar biosynthesis protein FlhA
CP049783.1_5055	K02401	369.10	498.4	5.9e-150	flagellar biosynthesis protein FlhB
CP049783.1_5056	K02421	174.00	262.8	1.2e-78	flagellar biosynthesis protein FliR
CP049783.1_5057	K02420	87.50	124.7	6e-37	flagellar biosynthesis protein FliQ
CP049783.1_5058	K02419	282.17	400.5	3.6e-120	flagellar biosynthesis protein FliP
CP049783.1_5059	K02418	21.93	69.7	8.3e-20	flagellar protein FliO/FliZ
CP049783.1_5060	K03413	138.63	154.6	1.4e-45	two-component system, chemotaxis family, chemotaxis protein CheY
CP049783.1_5061	K02417	65.07	222.8	1.7e-66	flagellar motor switch protein FliN
CP049783.1_5062	K02416	93.77	272.9	2.2e-81	flagellar motor switch protein FliM
CP049783.1_5063	K02415	30.53	60.3	7.9e-17	flagellar protein FliL
CP049783.1_5064	K02385	31.73	87.2	1.7e-25	flagellar protein FlbD
CP049783.1_5065	K02390	168.33	171.6	7.1e-51	flagellar hook protein FlgE
CP049783.1_5067	K02389	30.80	128.4	1.7e-37	flagellar basal-body rod modification protein FlgD
CP049783.1_5068	K02414	61.60	117.5	3.9e-34	flagellar hook-length control protein FliK
CP049783.1_5069	K02383	68.77	84.0	3.6e-24	flagellar protein FlbB
CP049783.1_5070	K02413	36.40	67.6	5e-19	flagellar protein FliJ
CP049783.1_5071	K02412	640.17	707.3	5.5e-213	flagellum-specific ATP synthase [EC:7.4.2.8]
CP049783.1_5072	K02411	80.00	117.3	4.3e-34	flagellar assembly protein FliH
CP049783.1_5073	K02410	88.33	426.6	4.2e-128	flagellar motor switch protein FliG
CP049783.1_5074	K02409	124.47	342.5	1.5e-102	flagellar M-ring protein FliF
CP049783.1_5075	K02408	32.97	93.6	5.9e-27	flagellar hook-basal body complex protein FliE
CP049783.1_5076	K02388	55.97	195.8	2.6e-58	flagellar basal-body rod protein FlgC
CP049783.1_5077	K02387	71.43	113.3	3e-33	flagellar basal-body rod protein FlgB
CP049783.1_5078	K03667	285.07	706.3	9.7e-213	ATP-dependent HslUV protease ATP-binding subunit HslU
CP049783.1_5079	K01419	82.90	303.0	7.6e-91	ATP-dependent HslUV protease, peptidase subunit HslV [EC:3.4.25.2]
CP049783.1_5080	K04094	353.90	776.9	2.4e-234	methylenetetrahydrofolate--tRNA-(uracil-5-)-methyltransferase [EC:2.1.1.74]
CP049783.1_5081	K03168	448.53	980.1	4.7e-295	DNA topoisomerase I [EC:5.6.2.1]
CP049783.1_5082	K04096	28.87	370.5	5.1e-111	DNA processing protein
CP049783.1_5083	K01902	347.77	507.8	5.7e-153	succinyl-CoA synthetase alpha subunit [EC:6.2.1.5]
CP049783.1_5084	K01903	498.17	590.4	7.5e-178	succinyl-CoA synthetase beta subunit [EC:6.2.1.5]
CP049783.1_5085	K23775	99.10	184.4	1.1e-54	MarR family transcriptional regulator, organic hydroperoxide resistance regulator
CP049783.1_5086	K04063	57.63	181.2	8.1e-54	lipoyl-dependent peroxiredoxin [EC:1.11.1.28]
CP049783.1_5087	K07391	246.20	749.5	1.5e-225	magnesium chelatase family protein
CP049783.1_5091	K07460	34.73	144.2	1.1e-42	putative endonuclease

CP049783.1_5092	K04061	105.87	131.2	1.1e-38	flagellar biosynthesis protein
CP049783.1_5094	K03470	126.33	271.6	2.6e-81	ribonuclease HII [EC:3.1.26.4]
CP049783.1_5095	K14540	297.00	433.9	1.5e-130	ribosome biogenesis GTPase A
CP049783.1_5096	K03100	114.77	209.3	2.5e-62	signal peptidase I [EC:3.4.21.89]
CP049783.1_5097	K02884	25.43	197.0	1.1e-58	large subunit ribosomal protein L19
CP049783.1_5098	K00554	108.40	427.7	1.1e-128	tRNA (guanine37-N1)-methyltransferase [EC:2.1.1.228]
CP049783.1_5099	K02860	39.17	185.6	4.3e-55	16S rRNA processing protein RimM
CP049783.1_5100	K06960	46.00	99.7	5.1e-29	uncharacterized protein
CP049783.1_5101	K02959	27.40	149.5	3.4e-44	small subunit ribosomal protein S16
CP049783.1_5102	K03106	330.30	670.0	1.2e-201	signal recognition particle subunit SRP54 [EC:3.6.5.4]
CP049783.1_5103	K09787	52.33	141.0	1.5e-41	uncharacterized protein
CP049783.1_5104	K11068	170.70	290.6	4.6e-87	hemolysin III
CP049783.1_5105	K03110	289.73	497.7	9.4e-150	fused signal recognition particle receptor
CP049783.1_5106	K03529	574.17	1133.7	0	chromosome segregation protein
CP049783.1_5107	K03685	155.00	277.9	5.5e-83	ribonuclease III [EC:3.1.26.3]
CP049783.1_5108	K09458	480.27	649.9	1.3e-195	3-oxoacyl-[acyl-carrier-protein] synthase II [EC:2.3.1.179]
CP049783.1_5109	K02078	39.80	96.5	7.2e-28	acyl carrier protein
CP049783.1_5110	K00059	269.80	374.6	1.9e-112	3-oxoacyl-[acyl-carrier protein] reductase [EC:1.1.1.100]
CP049783.1_5111	K00645	247.17	388.7	1.9e-116	[acyl-carrier-protein] S-malonyltransferase [EC:2.3.1.39]
CP049783.1_5112	K00648	229.33	464.6	1.1e-139	3-oxoacyl-[acyl-carrier-protein] synthase III [EC:2.3.1.180]
CP049783.1_5113	K03621	111.17	466.4	2.2e-140	phosphate acyltransferase [EC:2.3.1.274]
CP049783.1_5115	K02911	22.10	72.6	1.3e-20	large subunit ribosomal protein L32
CP049783.1_5116	K07040	18.17	108.2	2.1e-31	DUF177 domain-containing protein
CP049783.1_5117	K27343	225.90	478.2	9.3e-144	tRNAmet cytidine acetate ligase [EC:6.3.1.22]
CP049783.1_5118	K07177	99.10	316.7	6.5e-95	Lon-like protease
CP049783.1_5120	K00954	81.30	262.4	1.3e-78	pantetheine-phosphate adenylyltransferase [EC:2.7.7.3]
CP049783.1_5121	K08316	94.87	214.1	7.5e-64	16S rRNA (guanine966-N2)-methyltransferase [EC:2.1.1.171]
CP049783.1_5122	K07800	25.63	26.8	1.4e-06	AgrD protein
CP049783.1_5123	K07813	47.80	105.9	5.4e-31	accessory gene regulator B
CP049783.1_5125	K16568	159.77	169.4	3.6e-50	exopolysaccharide production protein ExoZ
CP049783.1_5128	K12308	328.20	428.2	2.2e-128	beta-galactosidase [EC:3.2.1.23]
CP049783.1_5129	K08174	208.07	414.1	1.2e-124	MFS transporter, FHS family, glucose/mannose:H+ symporter
CP049783.1_5131	K25232	87.57	189.4	3.1e-56	fatty acid kinase fatty acid binding subunit
CP049783.1_5133	K00355	115.87	181.7	7.9e-54	NAD(P)H dehydrogenase (quinone) [EC:1.6.5.2]
CP049783.1_5137	K02435	35.93	38.4	2.1e-10	aspartyl-tRNA(Asn)/glutamyl-tRNA(Gln) amidotransferase subunit C [EC:6.3.5.6 6.3.5.7]
CP049783.1_5138	K05937	52.50	207.1	2.2e-62	uncharacterized protein
CP049783.1_5143	K09962	63.23	250.1	5.1e-75	uncharacterized protein
CP049783.1_5144	K08152	196.47	197.6	6.8e-59	MFS transporter, DHA1 family, multidrug resistance protein B
CP049783.1_5145	K20976	72.77	74.7	3.4e-21	histidine phosphotransfer protein HptB
CP049783.1_5146	K22491	123.17	143.1	5.1e-42	MerR family transcriptional regulator, light-induced transcriptional regulator
CP049783.1_5147	K15973	110.17	208.1	3.1e-62	MarR family transcriptional regulator, 2-MHQ and catechol-resistance regulon repressor
CP049783.1_5149	K01807	126.13	329.2	2.1e-98	ribose 5-phosphate isomerase A [EC:5.3.1.6]
CP049783.1_5150	K03829	91.77	139.1	5.8e-41	putative acetyltransferase [EC:2.3.1.-]
CP049783.1_5151	K03406	65.50	278.0	5.6e-83	methyl-accepting chemotaxis protein
CP049783.1_5155	K23774	112.50	267.9	1.9e-80	DeoR family transcriptional regulator, catabolite repression regulator
CP049783.1_5156	K00616	134.00	182.5	5.7e-54	transaldolase [EC:2.2.1.2]
CP049783.1_5157	K06075	115.10	130.2	3.6e-38	MarR family transcriptional regulator, transcriptional regulator for hemolysin
CP049783.1_5157	K15974	111.57	116.9	3.1e-34	MarR family transcriptional regulator, negative regulator of the multidrug operon emrRAB
CP049783.1_5157	K15973	110.17	112.0	8.8e-33	MarR family transcriptional regulator, 2-MHQ and catechol-resistance regulon repressor
CP049783.1_5160	K08221	232.17	306.4	7.7e-92	MFS transporter, ACDE family, multidrug resistance protein
CP049783.1_5161	K03406	65.50	254.0	1e-75	methyl-accepting chemotaxis protein
CP049783.1_5163	K01811	628.73	763.4	9.8e-230	alpha-D-xyloside xylohydrolase [EC:3.2.1.177]
CP049783.1_5165	K03311	209.80	563.9	8.7e-170	branched-chain amino acid:cation transporter, LIVCS family
CP049783.1_5166	K02532	177.13	520.9	5.6e-157	MFS transporter, OHS family, lactose permease
CP049783.1_5167	K03406	65.50	246.2	2.5e-73	methyl-accepting chemotaxis protein
CP049783.1_5168	K25086	282.43	349.5	8.6e-105	maltose transport system substrate-binding protein
CP049783.1_5172	K01486	325.67	693.8	5.8e-209	adenine deaminase [EC:3.5.4.2]
CP049783.1_5175	K12583	346.67	373.1	5e-112	phosphatidylinositol alpha 1,6-mannosyltransferase [EC:2.4.1.-]
CP049783.1_5175	K19002	285.93	306.9	8.5e-92	1,2-diacylglycerol 3-alpha-glucosyltransferase [EC:2.4.1.337]
CP049783.1_5176	K08984	86.30	305.2	8.7e-92	putative membrane protein
CP049783.1_5179	K01192	389.27	932.2	1.3e-280	beta-mannosidase [EC:3.2.1.25]
CP049783.1_5181	K17318	176.50	306.2	1.8e-91	putative aldouronate transport system substrate-binding protein
CP049783.1_5185	K03647	75.17	139.7	3.1e-41	protein involved in ribonucleotide reduction
CP049783.1_5186	K00525	367.50	436.8	5e-131	ribonucleoside-diphosphate reductase alpha chain [EC:1.17.4.1]
CP049783.1_5187	K00526	310.40	316.0	1.2e-94	ribonucleoside-diphosphate reductase beta chain [EC:1.17.4.1]
CP049783.1_5188	K08151	342.73	444.3	1.4e-133	MFS transporter, DHA1 family, tetracycline resistance protein
CP049783.1_5190	K04750	56.87	134.6	1.4e-39	PhnB protein

CP049783.1_5195	K06113	228.80	378.7	1.6e-113	arabinan endo-1,5-alpha-L-arabinosidase [EC:3.2.1.99]
CP049783.1_5196	K25064	365.20	495.3	1.7e-149	arabinooligosaccharide transport system permease protein
CP049783.1_5196	K02026	280.30	320.0	6e-96	multiple sugar transport system permease protein
CP049783.1_5197	K25063	422.37	525.7	1.1e-158	arabinooligosaccharide transport system permease protein
CP049783.1_5197	K02025	276.90	367.3	2.4e-110	multiple sugar transport system permease protein
CP049783.1_5198	K25062	497.50	740.1	2.2e-223	arabinooligosaccharide transport system substrate-binding protein
CP049783.1_5198	K02027	193.17	263.5	1.5e-78	multiple sugar transport system substrate-binding protein
CP049783.1_5201	K09702	62.87	193.9	8.1e-58	uncharacterized protein
CP049783.1_5202	K10907	500.80	577.6	6.5e-174	aminotransferase [EC:2.6.1.-]
CP049783.1_5204	K23518	203.53	249.4	2.7e-74	O-acetyl-ADP-ribose deacetylase [EC:3.1.1.106]
CP049783.1_5206	K16137	104.80	140.7	2.6e-41	TetR/AcrR family transcriptional regulator, transcriptional repressor for nem operon
CP049783.1_5207	K08224	171.37	188.1	6.2e-56	MFS transporter, YNFM family, putative membrane transport protein
CP049783.1_5211	K09458	480.27	600.5	1.1e-180	3-oxoacyl-[acyl-carrier-protein] synthase II [EC:2.3.1.179]
CP049783.1_5213	K03580	286.07	340.1	1.1e-101	ATP-dependent helicase HepA [EC:5.6.2.-]
CP049783.1_5217	K01448	29.13	140.0	2.8e-41	N-acetylmuramoyl-L-alanine amidase [EC:3.5.1.28]
CP049783.1_5218	K09798	36.03	281.3	3.4e-84	uncharacterized protein
CP049783.1_5219	K07304	43.27	244.3	6.6e-73	peptide-methionine (S)-S-oxide reductase [EC:1.8.4.11]
CP049783.1_5224	K03816	140.40	208.2	2.2e-62	xanthine phosphoribosyltransferase [EC:2.4.2.22]
CP049783.1_5226	K03975	59.97	87.1	3.4e-25	membrane-associated protein
CP049783.1_5230	K06183	293.40	358.6	1.1e-107	16S rRNA pseudouridine516 synthase [EC:5.4.99.19]
CP049783.1_5231	K11392	340.27	352.2	1.4e-105	16S rRNA (cytosine1407-C5)-methyltransferase [EC:2.1.1.178]
CP049783.1_5233	K09763	44.57	136.6	3.7e-40	uncharacterized protein
CP049783.1_5234	K07816	97.83	264.9	4e-79	GTP pyrophosphokinase [EC:2.7.6.5]
CP049783.1_5235	K00254	173.83	535.7	5.1e-161	dihydroorotate dehydrogenase [EC:1.3.5.2]
CP049783.1_5238	K05337	42.27	71.2	2.8e-20	ferredoxin
CP049783.1_5239	K02346	368.87	451.9	7.5e-136	DNA polymerase IV [EC:2.7.7.7]
CP049783.1_5240	K01649	498.33	591.7	1.1e-177	2-isopropylmalate synthase [EC:2.3.3.13]
CP049783.1_5242	K02342	101.50	156.9	2.2e-46	DNA polymerase III subunit epsilon [EC:2.7.7.7]
CP049783.1_5242	K09951	46.77	76.7	7.9e-22	CRISPR-associated protein Cas2
CP049783.1_5243	K07182	138.87	314.7	3.9e-94	CBS domain-containing protein
CP049783.1_5244	K03320	145.33	504.6	1.5e-151	ammonium transporter, Amt family
CP049783.1_5246	K11923	88.47	183.3	6.7e-55	MerR family transcriptional regulator, copper efflux regulator
CP049783.1_5247	K06973	70.50	324.0	2.8e-97	uncharacterized protein
CP049783.1_5248	K09681	263.80	486.1	1.1e-146	LysR family transcriptional regulator, transcription activator of glutamate synthase operon
CP049783.1_5250	K11065	132.27	256.6	6.9e-77	thioredoxin-dependent peroxiredoxin [EC:1.11.1.24]
CP049783.1_5253	K05592	531.43	670.1	9.8e-202	ATP-dependent RNA helicase DeaD [EC:5.6.2.7]
CP049783.1_5253	K11927	549.30	569.1	3.2e-171	ATP-dependent RNA helicase RhlE [EC:5.6.2.7]
CP049783.1_5257	K26939	355.77	450.9	1.5e-135	MATE family, multidrug efflux pump
CP049783.1_5259	K00850	359.57	399.8	7.2e-120	6-phosphofructokinase 1 [EC:2.7.1.11]
CP049783.1_5260	K16188	206.80	535.0	2.6e-161	tetraprenyl-beta-curcumene synthase [EC:4.2.3.130]
CP049783.1_5261	K26937	267.43	388.6	1.2e-116	MATE family, multidrug efflux pump
CP049783.1_5262	K03704	80.73	123.6	2.5e-36	cold shock protein
CP049783.1_5265	K08602	113.03	776.0	1.5e-233	oligoendopeptidase F [EC:3.4.24.-]
CP049783.1_5266	K08992	32.97	69.2	1.5e-19	lipopolysaccharide assembly protein A
CP049783.1_5267	K01269	358.40	543.9	4.2e-164	aminopeptidase [EC:3.4.11.-]
CP049783.1_5268	K15738	622.23	791.0	2.8e-238	ABC transport system ATP-binding/permease protein
CP049783.1_5269	K13770	123.73	134.4	1.5e-39	TetR/AcrR family transcriptional regulator, fatty acid metabolism regulator protein
CP049783.1_5272	K01126	152.53	217.2	8.7e-65	glycerophosphoryl diester phosphodiesterase [EC:3.1.4.46]
CP049783.1_5273	K07282	220.47	249.9	1.1e-74	gamma-polyglutamate biosynthesis protein CapA
CP049783.1_5274	K00231	144.83	468.3	1.2e-140	protoporphyrinogen/coproporphyrinogen III oxidase [EC:1.3.3.4 1.3.3.15]
CP049783.1_5275	K01772	59.27	312.6	1.6e-93	protoporphyrin/coproporphyrin ferrochelatase [EC:4.98.1.1 4.99.1.9]
CP049783.1_5276	K01599	181.83	445.1	1.3e-133	uroporphyrinogen decarboxylase [EC:4.1.1.37]
CP049783.1_5277	K08161	244.40	523.6	1.2e-157	MFS transporter, DHA1 family, multidrug resistance protein
CP049783.1_5279	K07444	419.40	569.8	1.9e-171	putative N6-adenine-specific DNA methylase [EC:2.1.1.-]
CP049783.1_5280	K19005	299.10	643.8	1.6e-193	lipoteichoic acid synthase [EC:2.7.8.20]
CP049783.1_5285	K01308	202.97	436.6	5.5e-131	g-D-glutamyl-meso-diaminopimelate peptidase [EC:3.4.19.11]
CP049783.1_5286	K01776	98.97	345.0	1.8e-103	glutamate racemase [EC:5.1.1.3]
CP049783.1_5288	K09012	140.67	195.3	3e-58	DeoR family transcriptional regulator, suf operon transcriptional repressor
CP049783.1_5292	K22278	73.27	202.5	3.7e-60	peptidoglycan-N-acetylglucosamine deacetylase [EC:3.5.1.104]
CP049783.1_5297	K02103	274.17	459.8	2.7e-138	GntR family transcriptional regulator, arabinose operon transcriptional repressor
CP049783.1_5298	K00096	164.17	418.7	1e-125	glycerol-1-phosphate dehydrogenase [NAD(P)+] [EC:1.1.1.261]
CP049783.1_5300	K00209	135.60	627.1	3.2e-189	enoyl-[acyl-carrier protein] reductase / trans-2-enoyl-CoA reductase (NAD+) [EC:1.3.1.9 1.3.1.44]
CP049783.1_5303	K07481	59.83	74.9	2.7e-21	transposase, IS5 family
CP049783.1_5305	K03826	152.20	182.2	5.5e-54	putative acetyltransferase [EC:2.3.1.-]
CP049783.1_5310	K27684	451.23	613.5	3.1e-185	carboxyaminopropylagmatine decarboxylase [EC:4.1.1.127]
CP049783.1_5311	K27502	571.50	753.7	1.7e-227	carboxyaminopropylagmatine dehydrogenase [EC:1.5.1.55]
CP049783.1_5313	K04079	91.80	693.6	1.6e-208	molecular chaperone HtpG

CP049783.1_5322	K01841	353.47	799.1	6.7e-241	phosphoenolpyruvate phosphomutase [EC:5.4.2.9]
CP049783.1_5323	K09459	328.73	487.3	2e-146	phosphonopyruvate decarboxylase [EC:4.1.1.82]
CP049783.1_5325	K25574	858.67	983.1	1.6e-296	(2-aminoethyl)phosphonate cytidylyltransferase [EC:2.7.7.107]
CP049783.1_5325	K03430	335.47	562.1	6.1e-169	2-aminoethylphosphonate-pyruvate transaminase [EC:2.6.1.37]
CP049783.1_5327	K00549	27.93	103.6	3.2e-30	5-methyltetrahydropteroyltriglutamate--homocysteine methyltransferase [EC:2.1.1.14]
CP049783.1_5329	K01223	479.40	616.1	1.6e-185	6-phospho-beta-glucosidase [EC:3.2.1.86]
CP049783.1_5330	K02757	642.13	742.4	1.4e-223	beta-glucoside PTS system EIICBA component [EC:2.7.1.-]
CP049783.1_5331	K03488	318.67	357.6	2.2e-107	beta-glucoside operon transcriptional antiterminator
CP049783.1_5332	K01607	17.10	132.7	6.4e-39	4-carboxymuconolactone decarboxylase [EC:4.1.1.44]
CP049783.1_5333	K01821	25.90	36.1	1.9e-09	4-oxalocrotonate tautomerase [EC:5.3.2.6]
CP049783.1_5334	K21960	245.43	375.6	4.7e-113	LysR family transcriptional regulator, regulator of the ytmI operon
CP049783.1_5336	K02768	144.33	172.3	4e-51	fructose PTS system EIIA component [EC:2.7.1.202]
CP049783.1_5337	K00882	316.47	378.6	1.1e-113	1-phosphofructokinase [EC:2.7.1.56]
CP049783.1_5338	K03436	282.67	290.1	4.8e-87	DeoR family transcriptional regulator, fructose operon transcriptional repressor
CP049783.1_5340	K10914	140.63	145.8	7.6e-43	CRP/FNR family transcriptional regulator, cyclic AMP receptor protein
CP049783.1_5343	K03406	65.50	263.9	1e-78	methyl-accepting chemotaxis protein
CP049783.1_5344	K25109	348.87	360.2	4.6e-108	iron-siderophore transport system substrate-binding protein
CP049783.1_5344	K25308	264.57	296.0	1e-88	ferric hydroxamate/heme transport system substrate-binding protein
CP049783.1_5348	K06889	113.67	121.8	1.1e-35	uncharacterized protein
CP049783.1_5351	K07738	41.80	244.0	4.3e-73	transcriptional repressor NrdR
CP049783.1_5352	K06418	101.07	107.2	9.5e-32	small acid-soluble spore protein A (major alpha-type SASP)
CP049783.1_5353	K08309	133.47	197.7	1.8e-58	peptidoglycan lytic transglycosylase [EC:4.2.2.29]
CP049783.1_5354	K00859	53.27	280.9	8.5e-84	dephospho-CoA kinase [EC:2.7.1.24]
CP049783.1_5355	K23242	61.23	69.8	6.6e-20	manganese efflux pump family protein
CP049783.1_5356	K10563	196.13	331.2	3.4e-99	formamidopyrimidine-DNA glycosylase [EC:3.2.2.23 4.2.99.18]
CP049783.1_5357	K02335	481.67	1109.4	0	DNA polymerase I [EC:2.7.7.7]
CP049783.1_5358	K02039	139.13	234.3	7.4e-70	phosphate transport system protein
CP049783.1_5359	K02036	260.70	451.9	3.4e-136	phosphate transport system ATP-binding protein [EC:7.3.2.1]
CP049783.1_5360	K03406	65.50	95.9	8.4e-28	methyl-accepting chemotaxis protein
CP049783.1_5361	K02483	242.00	256.8	1.3e-76	two-component system, OmpR family, response regulator
CP049783.1_5362	K07636	310.93	392.2	1e-117	two-component system, OmpR family, phosphate regulon sensor histidine kinase PhoR [EC:2.7.13.3]
CP049783.1_5365	K00024	377.73	408.3	1.3e-122	malate dehydrogenase [EC:1.1.1.37]
CP049783.1_5366	K00031	117.90	190.3	2.2e-56	isocitrate dehydrogenase [EC:1.1.1.42]
CP049783.1_5367	K01659	471.93	544.7	4.9e-164	2-methylcitrate synthase [EC:2.3.3.5]
CP049783.1_5369	K07113	64.97	145.1	5.3e-43	UPF0716 protein FxsA
CP049783.1_5370	K07107	72.43	119.9	6.4e-35	acyl-CoA thioester hydrolase [EC:3.1.2.-]
CP049783.1_5372	K02036	260.70	434.8	5.4e-131	phosphate transport system ATP-binding protein [EC:7.3.2.1]
CP049783.1_5373	K02038	250.80	293.1	8.5e-88	phosphate transport system permease protein
CP049783.1_5374	K02037	256.00	322.9	1.4e-96	phosphate transport system permease protein
CP049783.1_5375	K02040	56.17	245.3	4.5e-73	phosphate transport system substrate-binding protein
CP049783.1_5377	K00873	49.90	712.4	1.7e-214	pyruvate kinase [EC:2.7.1.40]
CP049783.1_5378	K01962	408.77	557.4	2.2e-167	acetyl-CoA carboxylase carboxyl transferase subunit alpha [EC:6.4.1.2 2.1.3.15]
CP049783.1_5379	K01963	380.60	456.7	3.2e-137	acetyl-CoA carboxylase carboxyl transferase subunit beta [EC:6.4.1.2 2.1.3.15]
CP049783.1_5382	K02337	932.93	1658.2	0	DNA polymerase III subunit alpha [EC:2.7.7.7]
CP049783.1_5398	K06334	183.93	339.7	1.2e-102	spore coat protein JC
CP049783.1_5399	K06333	33.97	130.4	1.4e-38	spore coat protein JB
CP049783.1_5400	K06332	36.80	117.6	1.8e-34	spore coat protein JA
CP049783.1_5401	K03699	305.47	509.0	4.9e-153	magnesium and cobalt exporter, CNNM family
CP049783.1_5403	K25135	249.93	273.0	1.5e-81	cyclic di-GMP phosphodiesterase [EC:3.1.4.-]
CP049783.1_5406	K07217	211.70	275.6	2e-82	manganese catalase [EC:1.11.1.6]
CP049783.1_5407	K07217	211.70	381.8	9.9e-115	manganese catalase [EC:1.11.1.6]
CP049783.1_5409	K09014	377.87	845.4	7.6e-255	Fe-S cluster assembly protein SufB
CP049783.1_5410	K04488	144.00	197.6	7.2e-59	nitrogen fixation protein NifU and related proteins
CP049783.1_5411	K11717	566.53	718.8	1.9e-216	cysteine desulfurase / selenocysteine lyase [EC:2.8.1.7 4.4.1.16]
CP049783.1_5412	K09015	272.00	323.7	7.3e-97	Fe-S cluster assembly protein SufD
CP049783.1_5413	K09013	161.43	420.0	1.4e-126	Fe-S cluster assembly ATP-binding protein
CP049783.1_5414	K04047	77.30	202.9	2.6e-60	starvation-inducible DNA-binding protein
CP049783.1_5415	K00899	378.97	544.6	1.2e-163	5-methylthioribose kinase [EC:2.7.1.100]
CP049783.1_5416	K08963	276.73	517.3	1.6e-155	methylthioribose-1-phosphate isomerase [EC:5.3.1.23]
CP049783.1_5418	K07559	140.20	228.4	3.1e-68	putative RNA 2'-phosphotransferase [EC:2.7.1.-]
CP049783.1_5423	K01426	303.90	402.7	1.3e-120	amidase [EC:3.5.1.4]
CP049784.1_8	K06284	86.83	95.0	9e-28	AbrB family transcriptional regulator, transcriptional pleiotropic regulator of transition state genes
CP049784.1_27	K06376	38.70	50.1	7.1e-14	stage 0 sporulation regulatory protein
CP049784.1_30	K18640	87.00	190.1	2.6e-56	plasmid segregation protein ParM
