# Supplementary material for: Prediction of soil probiotics based on foundation model representation enhancement and stacked aggregation classifier
Source: Brief Bioinform. 2025 Oct 29;26(5):bbaf567. doi: 10.1093/bib/bbaf567 (PMC12570017; doi:10.1093/bib/bbaf567)
Supplement: Supplementary_Table_S7_R2_bbaf567 [file supplementary_table_s7_r2_bbaf567.pdf]

Supplementary Table S7. Genes and their KO identifiers of GCA_000385945.1.

Gene_ID	KO	Threshold	Score	*e* _value	KO_definition
CP005080.1_2	K17836	82.80	303.3	1.4e-90	beta-lactamase class A [EC:3.5.2.6]
CP005080.1_4	K17836	82.80	238.5	6.3e-71	beta-lactamase class A [EC:3.5.2.6]
CP005080.1_9	K01624	67.57	308.1	6.8e-92	fructose-bisphosphate aldolase, class II [EC:4.1.2.13]
CP005080.1_14	K02027	193.17	252.7	3.7e-75	multiple sugar transport system substrate-binding protein
CP005080.1_16	K01727	501.77	1027.2	3.7e-309	hyaluronate lyase [EC:4.2.2.1]
CP005080.1_24	K09733	170.63	262.3	2.5e-78	(5-formylfuran-3-yl)methyl phosphate synthase [EC:4.2.3.153]
CP005080.1_26	K00055	388.33	478.8	7.1e-144	aryl-alcohol dehydrogenase [EC:1.1.1.90]
CP005080.1_31	K13787	299.27	374.5	3.7e-112	geranylgeranyl diphosphate synthase, type I [EC:2.5.1.1 2.5.1.10 2.5.1.29]
CP005080.1_32	K10027	520.10	739.2	1.6e-222	phytoene desaturase [EC:1.3.99.26 1.3.99.28 1.3.99.29 1.3.99.31]
CP005080.1_33	K02291	166.43	363.0	1.4e-108	15-cis-phytoene synthase [EC:2.5.1.32]
CP005080.1_35	K09879	561.80	797.3	6.4e-240	carotenoid phi-ring synthase / carotenoid chi-ring synthase [EC:1.3.99.39 1.3.99.40]
CP005080.1_37	K06443	105.03	315.0	3.4e-94	lycopene beta-cyclase [EC:5.5.1.19]
CP005080.1_41	K06377	63.37	410.6	1.9e-123	sporulation-control protein
CP005080.1_42	K03699	305.47	489.7	4.2e-147	magnesium and cobalt exporter, CNNM family
CP005080.1_43	K03699	305.47	348.2	3.2e-104	magnesium and cobalt exporter, CNNM family
CP005080.1_44	K07566	93.50	114.3	2.6e-33	L-threonylcarbamoyladenylate synthase [EC:2.7.7.87]
CP005080.1_50	K14339	391.03	485.0	1.3e-145	alpha-1,6-mannosyltransferase [EC:2.4.1.-]
CP005080.1_52	K00064	256.83	434.2	2e-130	D-threo-aldose 1-dehydrogenase [EC:1.1.1.122]
CP005080.1_56	K07315	145.67	193.4	2.6e-57	phosphoserine phosphatase RsbU/P [EC:3.1.3.3]
CP005080.1_63	K02274	750.43	928.7	1.2e-279	cytochrome c oxidase subunit I [EC:7.1.1.9]
CP005080.1_64	K06988	161.77	235.0	4.9e-70	8-hydroxy-5-deazaflavin:NADPH oxidoreductase [EC:1.5.1.40]
CP005080.1_67	K03088	96.50	182.2	5.6e-54	RNA polymerase sigma-70 factor, ECF subfamily
CP005080.1_75	K05605	174.27	389.2	1.1e-116	3-hydroxyisobutyryl-CoA hydrolase [EC:3.1.2.4]
CP005080.1_76	K07315	145.67	208.9	5.1e-62	phosphoserine phosphatase RsbU/P [EC:3.1.3.3]
CP005080.1_80	K07131	45.27	104.5	2.2e-30	uncharacterized protein
CP005080.1_82	K06945	72.87	301.0	5.9e-90	uncharacterized protein
CP005080.1_84	K01531	710.20	1137.6	0	P-type Mg2+ transporter [EC:7.2.2.14]
CP005080.1_85	K03116	54.03	70.4	7.7e-20	sec-independent protein translocase protein TatA
CP005080.1_86	K05795	235.30	359.4	8.7e-108	tellurium resistance protein TerD
CP005080.1_89	K10947	65.70	66.0	2.1e-18	PadR family transcriptional regulator
CP005080.1_98	K00138	763.17	1101.2	0	aldehyde dehydrogenase [EC:1.2.1.-]
CP005080.1_100	K07497	13.93	177.4	2.1e-52	putative transposase
CP005080.1_101	K07483	40.77	86.6	8.9e-25	transposase
CP005080.1_102	K05349	305.00	484.7	2.4e-145	beta-glucosidase [EC:3.2.1.21]
CP005080.1_104	K07483	40.77	80.1	8.7e-23	transposase
CP005080.1_105	K07497	13.93	166.7	4e-49	putative transposase
CP005080.1_108	K01990	262.37	342.4	1.6e-102	ABC-2 type transport system ATP-binding protein
CP005080.1_120	K18955	70.43	106.1	7.9e-31	WhiB family transcriptional regulator, redox-sensing transcriptional regulator
CP005080.1_124	K08224	171.37	175.4	5.6e-52	MFS transporter, YNFM family, putative membrane transport protein
CP005080.1_131	K07485	110.23	233.4	1.8e-69	transposase
CP005080.1_132	K07493	56.73	191.3	1.5e-56	putative transposase
CP005080.1_138	K00019	183.03	238.5	5.5e-71	3-hydroxybutyrate dehydrogenase [EC:1.1.1.30]
CP005080.1_146	K07493	56.73	160.9	2.4e-47	putative transposase
CP005080.1_152	K00134	414.10	454.9	1.5e-136	glyceraldehyde 3-phosphate dehydrogenase (phosphorylating) [EC:1.2.1.12]
CP005080.1_153	K13633	271.97	406.8	3.4e-122	AraC family transcriptional regulator, transcriptional activator FtrA
CP005080.1_157	K13936	190.93	292.7	1.7e-87	malonate transporter and related proteins
CP005080.1_160	K22770	293.13	478.2	8.1e-144	stearoyl-CoA 9-desaturase NADPH oxidoreductase
CP005080.1_161	K22769	317.50	658.9	1.6e-198	NADPH-dependent stearoyl-CoA 9-desaturase [EC:1.14.19.-]
CP005080.1_177	K22278	73.27	213.2	2.7e-63	peptidoglycan-N-acetylglucosamine deacetylase [EC:3.5.1.104]
CP005080.1_180	K05343	524.60	768.4	5.3e-231	maltose alpha-D-glucosyltransferase / alpha-amylase [EC:5.4.99.16 3.2.1.1]
CP005080.1_182	K01182	875.10	922.5	1e-277	oligo-1,6-glucosidase [EC:3.2.1.10]
CP005080.1_182	K01187	286.07	293.0	2.2e-87	alpha-glucosidase [EC:3.2.1.20]
CP005080.1_185	K13633	271.97	411.1	1.6e-123	AraC family transcriptional regulator, transcriptional activator FtrA
CP005080.1_186	K06718	104.57	206.8	1.3e-61	L-2,4-diaminobutyric acid acetyltransferase [EC:2.3.1.178]

CP005080.1_187	K06720	84.00	192.2	1.6e-57	L-ectoine synthase [EC:4.2.1.108]
CP005080.1_188	K02078	39.80	42.7	2.3e-11	acyl carrier protein
CP005080.1_197	K01183	115.40	251.7	6.3e-75	chitinase [EC:3.2.1.14]
CP005080.1_201	K07058	40.93	132.2	1.2e-38	membrane protein
CP005080.1_202	K07029	147.20	261.7	3.9e-78	diacylglycerol kinase (ATP) [EC:2.7.1.107]
CP005080.1_203	K07034	31.40	87.6	3.4e-25	uncharacterized protein
CP005080.1_214	K07452	78.20	316.2	1.3e-94	5-methylcytosine-specific restriction enzyme B [EC:3.1.21.-]
CP005080.1_215	K19147	46.97	292.0	3.6e-87	5-methylcytosine-specific restriction enzyme subunit McrC
CP005080.1_216	K01083	115.73	423.9	4.3e-127	3-phytase [EC:3.1.3.8]
CP005080.1_218	K01062	142.57	162.6	4.4e-48	platelet-activating factor acetylhydrolase [EC:3.1.1.47]
CP005080.1_221	K06994	260.33	801.9	2.3e-241	putative drug exporter of the RND superfamily
CP005080.1_222	K01673	19.40	128.8	8.6e-38	carbonic anhydrase [EC:4.2.1.1]
CP005080.1_225	K09992	76.67	282.0	3.1e-84	uncharacterized protein
CP005080.1_228	K17734	342.83	358.4	3.1e-107	serine protease AprX [EC:3.4.21.-]
CP005080.1_230	K07654	329.50	487.0	3.9e-146	two-component system, OmpR family, sensor histidine kinase MtrB [EC:2.7.13.3]
CP005080.1_233	K01990	262.37	338.1	3.4e-101	ABC-2 type transport system ATP-binding protein
CP005080.1_241	K05337	42.27	68.1	3.2e-19	ferredoxin
CP005080.1_242	K17876	616.67	681.9	9.2e-206	pentalenic acid synthase [EC:1.14.15.11]
CP005080.1_247	K01975	64.70	154.9	1.1e-45	RNA 2',3'-cyclic 3'-phosphodiesterase [EC:3.1.4.58]
CP005080.1_251	K23775	99.10	197.1	1.9e-58	MarR family transcriptional regulator, organic hydroperoxide resistance regulator
CP005080.1_252	K04063	57.63	184.2	1.3e-54	lipoyl-dependent peroxiredoxin [EC:1.11.1.28]
CP005080.1_258	K01201	204.70	445.8	9.8e-134	glucosylceramidase [EC:3.2.1.45]
CP005080.1_260	K00803	391.27	653.2	3.1e-196	alkyldihydroxyacetonephosphate synthase [EC:2.5.1.26]
CP005080.1_264	K13979	530.93	594.9	2.8e-179	alcohol dehydrogenase (NADP+) [EC:1.1.1.2]
CP005080.1_267	K22462	334.83	449.8	1.4e-135	(4-alkanoyl-5-oxo-2,5-dihydrofuran-3-yl)methyl phosphate reductase [EC:1.3.1.113]
CP005080.1_267	K19267	112.87	216.0	4.6e-64	NAD(P)H dehydrogenase (quinone) [EC:1.6.5.2]
CP005080.1_268	K22463	82.37	328.0	2.8e-98	2-oxo-3-(phosphooxy)propyl 3-oxoalkanoate synthase [EC:2.3.1.277]
CP005080.1_270	K06895	83.83	275.3	1.6e-82	L-lysine exporter family protein LysE/ArgO
CP005080.1_271	K05596	133.53	449.2	5.4e-135	LysR family transcriptional regulator, chromosome initiation inhibitor
CP005080.1_275	K06996	53.13	117.6	3.9e-34	uncharacterized protein
CP005080.1_277	K04343	77.20	341.7	2.9e-102	streptomycin 6-kinase [EC:2.7.1.72]
CP005080.1_278	K25706	413.33	527.7	1.3e-158	tRNA N6-adenosine threonylcarbamoyltransferase [EC:2.3.1.234]
CP005080.1_279	K01187	286.07	328.1	5.3e-98	alpha-glucosidase [EC:3.2.1.20]
CP005080.1_280	K02026	280.30	284.1	6e-85	multiple sugar transport system permease protein
CP005080.1_281	K02025	276.90	317.5	4e-95	multiple sugar transport system permease protein
CP005080.1_282	K02027	193.17	202.5	5.9e-60	multiple sugar transport system substrate-binding protein
CP005080.1_287	K00320	221.33	278.8	3.6e-83	5,10-methylenetetrahydromethanopterin reductase [EC:1.5.98.2]
CP005080.1_290	K01286	175.57	271.7	5.9e-81	D-alanyl-D-alanine carboxypeptidase [EC:3.4.16.4]
CP005080.1_293	K03436	282.67	337.9	1.7e-101	DeoR family transcriptional regulator, fructose operon transcriptional repressor
CP005080.1_294	K00882	316.47	389.6	6.3e-117	1-phosphofructokinase [EC:2.7.1.56]
CP005080.1_295	K02770	649.43	889.5	6.3e-268	fructose PTS system EIIBC or EIIC component [EC:2.7.1.202]
CP005080.1_295	K02768	144.33	177.0	1.8e-52	fructose PTS system EIIA component [EC:2.7.1.202]
CP005080.1_296	K03100	114.77	245.2	3.7e-73	signal peptidase I [EC:3.4.21.89]
CP005080.1_300	K27108	396.97	497.7	8.7e-150	epoxide hydrolase A/B [EC:3.3.2.-]
CP005080.1_309	K02909	21.83	90.4	4.5e-26	large subunit ribosomal protein L31
CP005080.1_310	K02913	23.23	72.5	1.1e-20	large subunit ribosomal protein L33
CP005080.1_312	K07315	145.67	192.9	3.6e-57	phosphoserine phosphatase RsbU/P [EC:3.1.3.3]
CP005080.1_316	K10187	462.13	1315.6	0	germacradienol/geosmin synthase [EC:4.2.3.22 4.2.3.75 4.1.99.16]
CP005080.1_323	K18688	403.13	762.4	1.4e-229	3-oxocholest-4-en-26-oate---CoA ligase [EC:6.2.1.42]
CP005080.1_325	K01912	291.17	724.5	4.9e-218	phenylacetate-CoA ligase [EC:6.2.1.30]
CP005080.1_326	K02030	76.77	103.1	7.4e-30	polar amino acid transport system substrate-binding protein
CP005080.1_327	K02029	214.53	227.3	1.3e-67	polar amino acid transport system permease protein
CP005080.1_328	K02028	386.70	415.5	1.2e-124	polar amino acid transport system ATP-binding protein [EC:7.4.2.1]
CP005080.1_330	K07116	433.37	839.3	1.6e-252	acyl-homoserine-lactone acylase [EC:3.5.1.97]
CP005080.1_336	K12132	224.03	339.7	6.7e-102	eukaryotic-like serine/threonine-protein kinase [EC:2.7.11.1]
CP005080.1_336	K11912	105.07	147.0	2.9e-43	serine/threonine-protein kinase PpkA [EC:2.7.11.1]

CP005080.1_341	K03701	341.57	831.4	3.9e-250	excinuclease ABC subunit A
CP005080.1_342	K06996	53.13	58.5	3.7e-16	uncharacterized protein
CP005080.1_343	K07506	141.00	142.3	9.2e-42	AraC family transcriptional regulator
CP005080.1_345	K00645	247.17	313.0	1.9e-93	[acyl-carrier-protein] S-malonyltransferase [EC:2.3.1.39]
CP005080.1_350	K22818	529.90	680.5	3.1e-205	3-oxocholest-4-en-26-oyl-CoA dehydrogenase alpha subunit [EC:1.3.99.-]
CP005080.1_351	K11391	321.67	591.0	8.3e-178	23S rRNA (guanine1835-N2)-methyltransferase [EC:2.1.1.174]
CP005080.1_351	K00564	110.40	245.9	3e-73	16S rRNA (guanine1207-N2)-methyltransferase [EC:2.1.1.172]
CP005080.1_353	K07273	48.73	142.5	6.4e-42	lysozyme
CP005080.1_355	K01425	83.30	438.5	1.7e-131	glutaminase [EC:3.5.1.2]
CP005080.1_359	K01621	304.80	1500.7	0	xylulose-5-phosphate/fructose-6-phosphate phosphoketolase [EC:4.1.2.9 4.1.2.22]
CP005080.1_360	K00135	580.20	620.8	1.1e-186	succinate-semialdehyde dehydrogenase / glutarate-semialdehyde dehydrogenase [EC:1.2.1.16 1.2.1.79 1.2.1.20]
CP005080.1_362	K11177	664.90	962.7	9.2e-290	xanthine dehydrogenase YagR molybdenum-binding subunit [EC:1.17.1.4]
CP005080.1_363	K11178	253.73	532.5	3.4e-160	xanthine dehydrogenase YagS FAD-binding subunit [EC:1.17.1.4]
CP005080.1_364	K13483	259.73	323.5	5.6e-97	xanthine dehydrogenase YagT iron-sulfur-binding subunit
CP005080.1_369	K06996	53.13	140.8	3.4e-41	uncharacterized protein
CP005080.1_376	K01104	65.07	127.4	2.7e-37	protein-tyrosine phosphatase [EC:3.1.3.48]
CP005080.1_378	K01847	612.40	618.5	7.2e-186	methylmalonyl-CoA mutase [EC:5.4.99.2]
CP005080.1_379	K01847	612.40	1080.3	0	methylmalonyl-CoA mutase [EC:5.4.99.2]
CP005080.1_380	K07588	261.13	543.4	4.6e-163	GTPase [EC:3.6.5.-]
CP005080.1_381	K02013	238.30	345.5	1.9e-103	iron complex transport system ATP-binding protein [EC:7.2.2.-]
CP005080.1_382	K25286	277.13	285.6	3.4e-85	iron-siderophore transport system substrate-binding protein
CP005080.1_383	K26141	392.27	420.7	3.8e-126	Xaa-Arg dipeptidase [EC:3.4.13.4]
CP005080.1_384	K04780	2024.83	3341.0	0	glyine---[glycyl-carrier protein] ligase [EC:6.2.1.66]
CP005080.1_385	K05375	34.70	79.6	6.4e-23	MbtH protein
CP005080.1_387	K07214	260.37	289.8	1.5e-86	iron(III)-enterobactin esterase [EC:3.1.1.108]
CP005080.1_388	K23186	409.23	1040.5	1.3e-313	iron-siderophore transport system permease protein
CP005080.1_389	K00216	256.33	416.8	3.8e-125	2,3-dihydro-2,3-dihydroxybenzoate dehydrogenase [EC:1.3.1.28]
CP005080.1_390	K02361	375.43	465.2	1e-139	isochorismate synthase [EC:5.4.4.2]
CP005080.1_391	K02363	778.17	930.3	1.5e-280	2,3-dihydroxybenzoate---[aryl-carrier protein] ligase [EC:6.3.2.14 6.2.1.71]
CP005080.1_392	K01252	314.80	331.4	1.5e-99	bifunctional isochorismate lyase / aryl carrier protein [EC:3.3.2.1 6.3.2.14]
CP005080.1_394	K03628	186.43	570.3	1.5e-171	transcription termination factor Rho
CP005080.1_395	K01835	371.63	412.7	1.3e-123	phosphoglucomutase [EC:5.4.2.2]
CP005080.1_397	K00074	404.50	412.0	1.5e-123	3-hydroxybutyryl-CoA dehydrogenase [EC:1.1.1.157]
CP005080.1_398	K01638	129.17	799.7	1.2e-240	malate synthase [EC:2.3.3.9]
CP005080.1_399	K01637	273.47	758.9	2.5e-228	isocitrate/methylisocitrate lyase [EC:4.1.3.1 4.1.3.30]
CP005080.1_400	K07110	619.40	684.2	4.7e-206	XRE family transcriptional regulator, fatty acid utilization regulator
CP005080.1_400	K21686	429.70	601.6	7.3e-181	XRE family transcriptional regulator, fatty acid utilization regulator
CP005080.1_404	K16785	103.27	164.7	1e-48	energy-coupling factor transport system permease protein
CP005080.1_405	K01552	403.80	609.1	4.1e-183	energy-coupling factor transport system ATP-binding protein [EC:7.-.-.-]
CP005080.1_406	K16927	47.27	298.7	2.4e-89	energy-coupling factor transport system substrate-specific component
CP005080.1_412	K17818	313.93	325.3	1.5e-97	D-arabinitol dehydrogenase (NADP+) [EC:1.1.1.287]
CP005080.1_413	K10229	324.37	401.1	9.1e-121	polyol transport system permease protein
CP005080.1_414	K10228	302.23	417.5	8.8e-126	polyol transport system permease protein
CP005080.1_415	K10227	439.80	647.4	4.8e-195	polyol transport system substrate-binding protein
CP005080.1_415	K02027	193.17	234.2	1.5e-69	multiple sugar transport system substrate-binding protein
CP005080.1_416	K03436	282.67	295.9	1e-88	DeoR family transcriptional regulator, fructose operon transcriptional repressor
CP005080.1_418	K07695	247.47	407.3	2.3e-122	two-component system, NarL family, response regulator DevR
CP005080.1_419	K07682	177.80	794.0	7.3e-239	two-component system, NarL family, sensor histidine kinase DevS [EC:2.7.13.3]
CP005080.1_423	K07005	53.80	71.3	4.6e-20	uncharacterized protein
CP005080.1_428	K25286	277.13	348.1	3.9e-104	iron-siderophore transport system substrate-binding protein
CP005080.1_429	K23188	469.87	488.1	2e-146	iron-siderophore transport system ATP-binding protein [EC:7.2.2.17 7.2.2.-]
CP005080.1_429	K02013	238.30	351.8	2.4e-105	iron complex transport system ATP-binding protein [EC:7.2.2.-]
CP005080.1_430	K23187	358.37	415.2	1.9e-124	iron-siderophore transport system permease protein
CP005080.1_431	K23186	409.23	463.5	4.3e-139	iron-siderophore transport system permease protein
CP005080.1_432	K10531	363.87	604.2	7.4e-182	L-ornithine N5-monooxygenase [EC:1.14.13.195 1.14.13.196]
CP005080.1_437	K02019	70.20	101.8	1.8e-29	molybdate transport system regulatory protein

CP005080.1_438	K03325	113.10	238.7	4.5e-71	arsenite transporter
CP005080.1_439	K07315	145.67	197.3	1.7e-58	phosphoserine phosphatase RsbU/P [EC:3.1.3.3]
CP005080.1_440	K22895	341.53	476.6	1.2e-143	renierapurpurin 18,18'-hydroxylase
CP005080.1_441	K03892	89.73	126.0	7.4e-37	ArsR family transcriptional regulator, arsenate/arsenite/antimonite-responsive transcriptional repressor
CP005080.1_443	K17686	799.73	980.5	3.4e-295	P-type Cu+ transporter [EC:7.2.2.8]
CP005080.1_453	K09919	57.70	75.1	1.9e-21	uncharacterized protein
CP005080.1_459	K15633	73.30	459.2	7.1e-138	2,3-bisphosphoglycerate-independent phosphoglycerate mutase [EC:5.4.2.12]
CP005080.1_460	K00800	150.47	531.4	1.2e-159	3-phosphoshikimate 1-carboxyvinyltransferase [EC:2.5.1.19]
CP005080.1_462	K03892	89.73	122.8	7.2e-36	ArsR family transcriptional regulator, arsenate/arsenite/antimonite-responsive transcriptional repressor
CP005080.1_465	K03892	89.73	101.8	1.8e-29	ArsR family transcriptional regulator, arsenate/arsenite/antimonite-responsive transcriptional repressor
CP005080.1_468	K02040	56.17	215.0	8.8e-64	phosphate transport system substrate-binding protein
CP005080.1_469	K01845	312.23	417.2	5.3e-125	glutamate-1-semialdehyde 2,1-aminomutase [EC:5.4.3.8]
CP005080.1_469	K23464	200.83	216.2	2.8e-64	flavin-dependent trigonelline monooxygenase, oxygenase component [EC:1.14.14.-]
CP005080.1_472	K03892	89.73	131.1	2e-38	ArsR family transcriptional regulator, arsenate/arsenite/antimonite-responsive transcriptional repressor
CP005080.1_475	K07222	277.37	410.1	4e-123	putative flavoprotein involved in K+ transport
CP005080.1_476	K03325	113.10	514.9	8.4e-155	arsenite transporter
CP005080.1_477	K03892	89.73	92.2	1.7e-26	ArsR family transcriptional regulator, arsenate/arsenite/antimonite-responsive transcriptional repressor
CP005080.1_478	K03741	124.07	239.5	2.1e-71	arsenate reductase (thioredoxin) [EC:1.20.4.4]
CP005080.1_480	K05878	498.43	545.8	5.3e-164	phosphoenolpyruvate---glycerone phosphotransferase subunit DhaK [EC:2.7.1.121]
CP005080.1_481	K05879	193.57	290.5	6.4e-87	phosphoenolpyruvate---glycerone phosphotransferase subunit DhaL [EC:2.7.1.121]
CP005080.1_482	K05881	93.30	154.1	2.1e-45	phosphoenolpyruvate---glycerone phosphotransferase subunit DhaM [EC:2.7.1.121]
CP005080.1_484	K18230	466.20	718.1	5.5e-216	macrolide transport system ATP-binding/permease protein
CP005080.1_485	K13953	338.93	348.9	1.5e-104	alcohol dehydrogenase, propanol-preferring [EC:1.1.1.1]
CP005080.1_487	K24699	97.17	100.0	4.9e-29	TetR/AcrR family transcriptional regulator, regulator of mycofactocin system
CP005080.1_490	K03090	268.20	399.7	7.6e-120	RNA polymerase sigma-B factor
CP005080.1_491	K01740	412.90	777.7	3.4e-234	O-acetylhomoserine (thiol)-lyase [EC:2.5.1.49]
CP005080.1_492	K00641	81.47	493.5	2.3e-148	homoserine O-acetyltransferase/O-succinyltransferase [EC:2.3.1.31 2.3.1.46]
CP005080.1_494	K08260	208.47	217.0	1.8e-64	adenosylcobinamide hydrolase [EC:3.5.1.90]
CP005080.1_496	K02013	238.30	352.3	1.6e-105	iron complex transport system ATP-binding protein [EC:7.2.2.-]
CP005080.1_497	K02015	340.73	418.4	1.8e-125	iron complex transport system permease protein
CP005080.1_498	K02016	154.03	189.6	3.8e-56	iron complex transport system substrate-binding protein
CP005080.1_500	K02078	39.80	42.2	3.3e-11	acyl carrier protein
CP005080.1_501	K00232	166.57	432.7	1.2e-129	acyl-CoA oxidase [EC:1.3.3.6]
CP005080.1_503	K00648	229.33	369.0	1.4e-110	3-oxoacyl-[acyl-carrier-protein] synthase III [EC:2.3.1.180]
CP005080.1_504	K00847	190.50	268.6	4.9e-80	fructokinase [EC:2.7.1.4]
CP005080.1_511	K23743	119.40	195.5	7.8e-58	peptidyl-Asp metalloendopeptidase [EC:3.4.24.33]
CP005080.1_513	K07058	40.93	224.6	1.3e-66	membrane protein
CP005080.1_515	K02529	268.37	271.8	5e-81	LacI family transcriptional regulator, galactose operon repressor
CP005080.1_518	K19580	527.47	641.1	1.5e-193	1,3,6,8-tetrahydroxynaphthalene synthase [EC:2.3.1.233]
CP005080.1_520	K23188	469.87	522.2	8.6e-157	iron-siderophore transport system ATP-binding protein [EC:7.2.2.17 7.2.2.-]
CP005080.1_520	K02013	238.30	349.4	1.2e-104	iron complex transport system ATP-binding protein [EC:7.2.2.-]
CP005080.1_521	K23187	358.37	435.6	1.2e-130	iron-siderophore transport system permease protein
CP005080.1_522	K23186	409.23	492.1	9.7e-148	iron-siderophore transport system permease protein
CP005080.1_523	K25286	277.13	358.1	3.8e-107	iron-siderophore transport system substrate-binding protein
CP005080.1_529	K00232	166.57	439.0	1.5e-131	acyl-CoA oxidase [EC:1.3.3.6]
CP005080.1_530	K07107	72.43	91.5	3.6e-26	acyl-CoA thioester hydrolase [EC:3.1.2.-]
CP005080.1_532	K18220	608.17	844.0	4e-254	ribosomal protection tetracycline resistance protein
CP005080.1_533	K01785	42.83	421.1	2.8e-126	aldose 1-epimerase [EC:5.1.3.3]
CP005080.1_534	K02529	268.37	335.6	2.4e-100	LacI family transcriptional regulator, galactose operon repressor
CP005080.1_535	K23509	334.60	345.5	1.2e-103	galactofuranose transport system permease protein
CP005080.1_537	K10820	647.93	790.1	2.8e-238	galactofuranose transport system ATP-binding protein [EC:7.5.2.9]
CP005080.1_537	K10441	693.10	698.8	3e-210	ribose transport system ATP-binding protein [EC:7.5.2.7]
CP005080.1_538	K23508	305.43	412.1	7.2e-124	galactofuranose transport system substrate-binding protein
CP005080.1_538	K10439	190.90	254.3	1.3e-75	ribose transport system substrate-binding protein
CP005080.1_539	K09955	70.47	176.5	3e-52	uncharacterized protein
CP005080.1_542	K01259	136.77	143.3	5.4e-42	proline iminopeptidase [EC:3.4.11.5]

CP005080.1_544	K04757	53.50	68.3	2.9e-19	serine/threonine-protein kinase RsbW [EC:2.7.11.1]
CP005080.1_545	K21397	347.03	1045.1	9.5e-315	ABC transport system ATP-binding/permease protein
CP005080.1_547	K12132	224.03	316.0	1e-94	eukaryotic-like serine/threonine-protein kinase [EC:2.7.11.1]
CP005080.1_547	K11912	105.07	159.9	3.7e-47	serine/threonine-protein kinase PpkA [EC:2.7.11.1]
CP005080.1_551	K18955	70.43	119.6	5.6e-35	WhiB family transcriptional regulator, redox-sensing transcriptional regulator
CP005080.1_552	K07315	145.67	161.4	1.3e-47	phosphoserine phosphatase RsbU/P [EC:3.1.3.3]
CP005080.1_555	K03469	83.37	149.7	4.5e-44	ribonuclease HI [EC:3.1.26.4]
CP005080.1_563	K01673	19.40	87.7	2.3e-25	carbonic anhydrase [EC:4.2.1.1]
CP005080.1_565	K13993	68.50	106.2	7.8e-31	HSP20 family protein
CP005080.1_571	K03574	70.57	78.0	2.6e-22	8-oxo-dGTP diphosphatase [EC:3.6.1.55]
CP005080.1_572	K03820	110.57	499.8	4.1e-150	apolipoprotein N-acyltransferase [EC:2.3.1.269]
CP005080.1_574	K01776	98.97	218.0	8.6e-65	glutamate racemase [EC:5.1.1.3]
CP005080.1_576	K05792	212.93	600.6	4.9e-181	tellurite resistance protein TerA
CP005080.1_578	K07140	274.73	313.4	8.6e-94	MOSC domain-containing protein
CP005080.1_580	K06413	333.77	367.9	2.7e-110	stage V sporulation protein K
CP005080.1_589	K04757	53.50	56.2	1.4e-15	serine/threonine-protein kinase RsbW [EC:2.7.11.1]
CP005080.1_598	K23980	470.93	726.6	5e-219	cysteinylglycine-S-conjugate dipeptidase [EC:3.4.13.23]
CP005080.1_601	K07315	145.67	167.3	2.1e-49	phosphoserine phosphatase RsbU/P [EC:3.1.3.3]
CP005080.1_601	K04757	53.50	59.1	1.9e-16	serine/threonine-protein kinase RsbW [EC:2.7.11.1]
CP005080.1_603	K25026	274.23	355.2	2.1e-106	glucokinase [EC:2.7.1.2]
CP005080.1_604	K02529	268.37	295.4	3.7e-88	LacI family transcriptional regulator, galactose operon repressor
CP005080.1_609	K03522	225.07	351.0	6.2e-105	electron transfer flavoprotein alpha subunit
CP005080.1_610	K03521	77.00	264.5	7.5e-79	electron transfer flavoprotein beta subunit
CP005080.1_613	K00655	122.40	181.2	1.3e-53	1-acyl-sn-glycerol-3-phosphate acyltransferase [EC:2.3.1.51]
CP005080.1_615	K01620	94.73	288.5	5.2e-86	threonine aldolase [EC:4.1.2.48]
CP005080.1_618	K01126	152.53	171.5	9.1e-51	glycerophosphoryl diester phosphodiesterase [EC:3.1.4.46]
CP005080.1_621	K02172	69.77	84.7	1.9e-24	bla regulator protein blaR1
CP005080.1_626	K00528	215.37	499.2	7.2e-150	ferredoxin/flavodoxin---NADP+ reductase [EC:1.18.1.2 1.19.1.1]
CP005080.1_632	K03648	63.37	273.2	1.8e-81	uracil-DNA glycosylase [EC:3.2.2.27]
CP005080.1_635	K07404	130.17	381.3	3e-114	6-phosphogluconolactonase [EC:3.1.1.31]
CP005080.1_638	K01091	112.83	113.1	7.2e-33	phosphoglycolate phosphatase [EC:3.1.3.18]
CP005080.1_639	K12132	224.03	294.8	2.9e-88	eukaryotic-like serine/threonine-protein kinase [EC:2.7.11.1]
CP005080.1_639	K11912	105.07	113.3	4.7e-33	serine/threonine-protein kinase PpkA [EC:2.7.11.1]
CP005080.1_644	K21401	242.60	551.9	4.4e-166	menaquinone-9 beta-reductase [EC:1.3.99.38]
CP005080.1_648	K03699	305.47	431.6	1.7e-129	magnesium and cobalt exporter, CNNM family
CP005080.1_650	K02171	45.30	57.7	5.2e-16	BlaI family transcriptional regulator, penicillinase repressor
CP005080.1_651	K05795	235.30	357.7	2.7e-107	tellurium resistance protein TerD
CP005080.1_652	K05791	193.43	283.7	2.8e-85	tellurium resistance protein TerZ
CP005080.1_654	K07010	123.83	282.5	1.9e-84	putative glutamine amidotransferase
CP005080.1_655	K16238	275.50	647.1	8.4e-195	ethanolamine permease
CP005080.1_657	K01915	33.97	299.6	2.2e-89	glutamine synthetase [EC:6.3.1.2]
CP005080.1_665	K16648	150.63	1498.5	0	arabinofuranan 3-O-arabinosyltransferase [EC:2.4.2.-]
CP005080.1_669	K09791	36.30	78.1	2.3e-22	uncharacterized protein
CP005080.1_675	K25286	277.13	326.4	1.5e-97	iron-siderophore transport system substrate-binding protein
CP005080.1_676	K23186	409.23	477.4	2.7e-143	iron-siderophore transport system permease protein
CP005080.1_677	K23187	358.37	396.9	6.6e-119	iron-siderophore transport system permease protein
CP005080.1_682	K12349	108.70	818.6	2e-246	neutral ceramidase [EC:3.5.1.23]
CP005080.1_687	K24160	182.33	326.2	1.7e-97	sodium/hydrogen antiporter
CP005080.1_688	K06978	225.60	477.6	3e-143	uncharacterized protein
CP005080.1_693	K01091	112.83	122.1	1.3e-35	phosphoglycolate phosphatase [EC:3.1.3.18]
CP005080.1_693	K07058	40.93	56.5	1.1e-15	membrane protein
CP005080.1_695	K07695	247.47	393.4	4e-118	two-component system, NarL family, response regulator DevR
CP005080.1_696	K07682	177.80	775.7	2.5e-233	two-component system, NarL family, sensor histidine kinase DevS [EC:2.7.13.3]
CP005080.1_701	K03090	268.20	397.7	3e-119	RNA polymerase sigma-B factor
CP005080.1_705	K03892	89.73	126.7	4.7e-37	ArsR family transcriptional regulator, arsenate/arsenite/antimonite-responsive transcriptional repressor
CP005080.1_706	K03892	89.73	104.0	4e-30	ArsR family transcriptional regulator, arsenate/arsenite/antimonite-responsive transcriptional repressor

CP005080.1_710	K03466	191.80	219.5	3.6e-65	DNA segregation ATPase FtsK/SpoIIIE, S-DNA-T family
CP005080.1_715	K14956	27.87	35.9	3.6e-09	ESAT-6 family protein
CP005080.1_719	K22894	131.70	297.3	8.1e-89	SARP family transcriptional regulator, regulator of embCAB operon
CP005080.1_720	K03088	96.50	129.9	4.4e-38	RNA polymerase sigma-70 factor, ECF subfamily
CP005080.1_722	K22894	131.70	241.3	8.2e-72	SARP family transcriptional regulator, regulator of embCAB operon
CP005080.1_723	K22297	134.93	139.0	6.8e-41	Fur family transcriptional regulator, stress-responsive regulator
CP005080.1_724	K03782	521.77	1432.0	0	catalase-peroxidase [EC:1.11.1.21]
CP005080.1_725	K23187	358.37	410.6	4.5e-123	iron-siderophore transport system permease protein
CP005080.1_726	K23186	409.23	442.0	1.4e-132	iron-siderophore transport system permease protein
CP005080.1_728	K23185	221.67	275.0	3.2e-82	ferric enterobactin transport system substrate-binding protein
CP005080.1_729	K07229	152.23	190.9	9.7e-57	ferric-chelate reductase (NADPH) [EC:1.16.1.9]
CP005080.1_732	K08177	233.33	381.1	2.7e-114	MFS transporter, OFA family, oxalate/formate antiporter
CP005080.1_734	K00232	166.57	615.9	4.9e-185	acyl-CoA oxidase [EC:1.3.3.6]
CP005080.1_738	K23778	108.13	123.1	6.2e-36	TetR/AcrR family transcriptional regulator, regulator of biofilm formation and stress response
CP005080.1_741	K15554	248.37	307.0	4.4e-92	sulfonate transport system permease protein
CP005080.1_741	K02050	145.27	205.5	6.5e-61	NitT/TauT family transport system permease protein
CP005080.1_742	K15555	333.60	343.5	5.5e-103	sulfonate transport system ATP-binding protein [EC:7.6.2.14]
CP005080.1_743	K15553	187.27	290.3	1.6e-86	sulfonate transport system substrate-binding protein
CP005080.1_744	K04091	325.40	630.8	1.1e-189	alkanesulfonate monooxygenase [EC:1.14.14.5 1.14.14.34]
CP005080.1_746	K07082	42.70	255.7	2.5e-76	peptidoglycan lytic transglycosylase G [EC:4.2.2.29]
CP005080.1_748	K06147	612.93	658.3	6.7e-198	ATP-binding cassette, subfamily B, bacterial
CP005080.1_750	K18930	469.13	1165.0	0	D-lactate dehydrogenase
CP005080.1_758	K03885	203.30	465.5	1.5e-139	NADH:quinone reductase (non-electrogenic) [EC:1.6.5.9]
CP005080.1_760	K07038	27.13	68.6	2e-19	inner membrane protein
CP005080.1_762	K10805	357.83	442.3	8.6e-133	acyl-CoA thioesterase II [EC:3.1.2.-]
CP005080.1_764	K22934	296.37	643.9	1.4e-193	alpha,alpha-trehalase [EC:3.2.1.28]
CP005080.1_768	K19113	120.03	183.2	9.5e-55	acetyltransferase [EC:2.3.1.-]
CP005080.1_769	K00432	54.07	221.2	6.3e-66	glutathione peroxidase [EC:1.11.1.9]
CP005080.1_772	K01462	69.73	229.7	2.1e-68	peptide deformylase [EC:3.5.1.88]
CP005080.1_773	K23393	162.27	485.3	8.3e-146	lipid II isoglutaminyl synthase (glutamine-hydrolysing) [EC:6.3.5.13]
CP005080.1_774	K07009	104.50	344.4	4.3e-103	lipid II isoglutaminyl synthase (glutamine-hydrolysing) [EC:6.3.5.13]
CP005080.1_775	K21071	345.93	446.1	6.7e-134	ATP-dependent phosphofructokinase / diphosphate-dependent phosphofructokinase [EC:2.7.1.11 2.7.1.90]
CP005080.1_776	K02351	164.43	323.8	6.6e-97	putative membrane protein
CP005080.1_778	K00655	122.40	253.7	1.2e-75	1-acyl-sn-glycerol-3-phosphate acyltransferase [EC:2.3.1.51]
CP005080.1_781	K03190	56.23	210.1	2.5e-62	urease accessory protein
CP005080.1_782	K03189	157.07	384.5	2.3e-115	urease accessory protein
CP005080.1_783	K03188	67.90	190.8	1.6e-56	urease accessory protein
CP005080.1_784	K01428	617.27	1064.2	0	urease subunit alpha [EC:3.5.1.5]
CP005080.1_785	K01429	171.47	179.3	3.8e-53	urease subunit beta [EC:3.5.1.5]
CP005080.1_786	K01430	175.63	204.5	3.1e-61	urease subunit gamma [EC:3.5.1.5]
CP005080.1_787	K21962	144.53	173.8	2.5e-51	TetR/AcrR family transcriptional regulator, transcriptional repressor of aconitase
CP005080.1_791	K01358	76.73	243.2	1.6e-72	ATP-dependent Clp protease, protease subunit [EC:3.4.21.92]
CP005080.1_794	K05835	156.00	169.6	2.3e-50	threonine efflux protein
CP005080.1_796	K04757	53.50	68.7	2.2e-19	serine/threonine-protein kinase RsbW [EC:2.7.11.1]
CP005080.1_799	K00652	449.27	482.3	8.8e-145	8-amino-7-oxononanoate synthase [EC:2.3.1.47]
CP005080.1_800	K01012	173.23	470.2	3e-141	biotin synthase [EC:2.8.1.6]
CP005080.1_801	K00833	407.37	662.8	1.3e-199	adenosylmethionine---8-amino-7-oxononanoate aminotransferase [EC:2.6.1.62]
CP005080.1_802	K01935	123.07	245.6	3.9e-73	dethiobiotin synthetase [EC:6.3.3.3]
CP005080.1_807	K03699	305.47	484.4	1.8e-145	magnesium and cobalt exporter, CNNM family
CP005080.1_808	K03699	305.47	362.3	1.8e-108	magnesium and cobalt exporter, CNNM family
CP005080.1_810	K01756	328.93	473.7	5.3e-142	adenylosuccinate lyase [EC:4.3.2.2]
CP005080.1_811	K03649	188.13	289.7	9e-87	double-stranded uracil-DNA glycosylase [EC:3.2.2.28]
CP005080.1_816	K03088	96.50	125.3	1.2e-36	RNA polymerase sigma-70 factor, ECF subfamily
CP005080.1_819	K00375	343.30	586.0	3.2e-176	GntR family transcriptional regulator / MocR family aminotransferase
CP005080.1_820	K01113	89.83	277.8	8.4e-83	alkaline phosphatase D [EC:3.1.3.1]
CP005080.1_824	K01761	494.87	515.8	3.2e-155	methionine-gamma-lyase [EC:4.4.1.11]

CP005080.1_825	K03719	131.40	147.6	1.7e-43	Lrp/AsnC family transcriptional regulator, leucine-responsive regulatory protein
CP005080.1_829	K03719	131.40	175.5	5e-52	Lrp/AsnC family transcriptional regulator, leucine-responsive regulatory protein
CP005080.1_830	K03546	183.10	827.9	6.5e-249	DNA repair protein SbcC/Rad50
CP005080.1_831	K03547	134.00	357.3	5.6e-107	DNA repair protein SbcD/Mre11
CP005080.1_833	K06929	81.97	187.6	8.2e-56	uncharacterized protein
CP005080.1_834	K00662	43.03	322.2	2e-96	aminoglycoside 3-N-acetyltransferase [EC:2.3.1.81]
CP005080.1_838	K01726	184.57	219.5	3.3e-65	gamma-carbonic anhydrase [EC:4.2.1.-]
CP005080.1_842	K02358	336.50	646.4	1.2e-194	elongation factor Tu
CP005080.1_845	K06153	46.03	365.2	1.8e-109	undecaprenyl-diphosphatase [EC:3.6.1.27]
CP005080.1_851	K03088	96.50	135.5	9e-40	RNA polymerase sigma-70 factor, ECF subfamily
CP005080.1_856	K03820	110.57	399.1	1.3e-119	apolipoprotein N-acyltransferase [EC:2.3.1.269]
CP005080.1_857	K03810	165.37	400.7	1.6e-120	virulence factor
CP005080.1_860	K03648	63.37	370.4	5.7e-111	uracil-DNA glycosylase [EC:3.2.2.27]
CP005080.1_861	K02035	249.67	296.1	2.3e-88	peptide/nickel transport system substrate-binding protein
CP005080.1_863	K00059	269.80	312.0	2.4e-93	3-oxoacyl-[acyl-carrier protein] reductase [EC:1.1.1.100]
CP005080.1_866	K00384	332.10	372.2	1.4e-111	thioredoxin reductase (NADPH) [EC:1.8.1.9]
CP005080.1_875	K07005	53.80	234.8	8.4e-70	uncharacterized protein
CP005080.1_876	K03298	170.77	306.2	1.4e-91	drug/metabolite transporter, DME family
CP005080.1_888	K06998	103.40	145.8	6.7e-43	trans-2,3-dihydro-3-hydroxyanthranilate isomerase [EC:5.3.3.17]
CP005080.1_889	K07052	31.30	42.0	2.4e-11	CAAX protease family protein
CP005080.1_894	K00281	631.07	1773.5	0	glycine cleavage system P protein (glycine dehydrogenase) [EC:1.4.4.2]
CP005080.1_896	K02346	368.87	555.6	3.6e-167	DNA polymerase IV [EC:2.7.7.7]
CP005080.1_898	K08999	28.90	209.2	3.2e-62	uncharacterized protein
CP005080.1_904	K16881	547.10	1160.0	0	mannose-1-phosphate guanylyltransferase / phosphomannomutase [EC:2.7.7.13 5.4.2.8]
CP005080.1_904	K00966	307.27	329.8	8.1e-99	mannose-1-phosphate guanylyltransferase [EC:2.7.7.13]
CP005080.1_905	K08744	154.77	180.2	2.3e-53	cardiolipin synthase (CMP-forming) [EC:2.7.8.41]
CP005080.1_907	K08483	678.63	705.7	3.3e-212	phosphoenolpyruvate-protein phosphotransferase (PTS system enzyme I) [EC:2.7.3.9]
CP005080.1_909	K01907	543.53	979.0	7.8e-295	acetoacetyl-CoA synthetase [EC:6.2.1.16]
CP005080.1_912	K08641	65.87	291.8	3e-87	zinc D-Ala-D-Ala dipeptidase [EC:3.4.13.22]
CP005080.1_916	K06945	72.87	317.0	7.8e-95	uncharacterized protein
CP005080.1_918	K07131	45.27	113.7	3.4e-33	uncharacterized protein
CP005080.1_924	K01469	916.37	1828.1	0	5-oxoprolinase (ATP-hydrolysing) [EC:3.5.2.9]
CP005080.1_931	K07149	76.20	161.7	7.2e-48	uncharacterized protein
CP005080.1_932	K01126	152.53	217.6	8.2e-65	glycerophosphoryl diester phosphodiesterase [EC:3.1.4.46]
CP005080.1_933	K06902	142.93	490.8	1.5e-147	MFS transporter, UMF1 family
CP005080.1_935	K07113	64.97	140.8	1.4e-41	UPF0716 protein FxsA
CP005080.1_936	K00721	182.03	355.6	1.7e-106	dolichol-phosphate mannosyltransferase [EC:2.4.1.83]
CP005080.1_938	K03719	131.40	176.1	3.4e-52	Lrp/AsnC family transcriptional regulator, leucine-responsive regulatory protein
CP005080.1_941	K00249	382.07	413.3	7.2e-124	acyl-CoA dehydrogenase [EC:1.3.8.7]
CP005080.1_942	K01183	115.40	258.1	7.2e-77	chitinase [EC:3.2.1.14]
CP005080.1_950	K03699	305.47	367.9	3.5e-110	magnesium and cobalt exporter, CNNM family
CP005080.1_951	K03699	305.47	491.7	1e-147	magnesium and cobalt exporter, CNNM family
CP005080.1_953	K00765	43.93	304.1	5.3e-91	ATP phosphoribosyltransferase [EC:2.4.2.17]
CP005080.1_955	K00794	39.60	204.2	1.2e-60	6,7-dimethyl-8-ribityllumazine synthase [EC:2.5.1.78]
CP005080.1_956	K14652	367.03	732.5	1.9e-220	3,4-dihydroxy 2-butanone 4-phosphate synthase / GTP cyclohydrolase II [EC:4.1.99.12 3.5.4.25]
CP005080.1_956	K01497	257.40	313.0	1.3e-93	GTP cyclohydrolase II [EC:3.5.4.25]
CP005080.1_957	K03811	46.70	160.8	1.9e-47	nicotinamide mononucleotide transporter
CP005080.1_958	K00793	67.77	321.4	1.9e-96	riboflavin synthase [EC:2.5.1.9]
CP005080.1_959	K11752	189.40	516.0	8.6e-155	diaminohydroxyphosphoribosylaminopyrimidine deaminase / 5-amino-6-(5-phosphoribosylamino)uracil reductase [EC:3.5.4.26 1.1.1.193]
CP005080.1_960	K01183	115.40	258.8	4.6e-77	chitinase [EC:3.2.1.14]
CP005080.1_961	K19267	112.87	114.8	2.4e-33	NAD(P)H dehydrogenase (quinone) [EC:1.6.5.2]
CP005080.1_967	K00468	612.63	996.4	1.3e-300	lysine 2-monooxygenase [EC:1.13.12.2]
CP005080.1_968	K01506	276.80	424.3	6.4e-128	5-aminopentanamidase [EC:3.5.1.30]
CP005080.1_969	K03719	131.40	144.6	1.3e-42	Lrp/AsnC family transcriptional regulator, leucine-responsive regulatory protein
CP005080.1_971	K26110	516.30	876.6	2.2e-264	GMP reductase [EC:1.7.1.7]
CP005080.1_974	K01783	248.20	350.2	5.1e-105	ribulose-phosphate 3-epimerase [EC:5.1.3.1]

CP005080.1_975	K03500	303.70	497.3	4.3e-149	16S rRNA (cytosine967-C5)-methyltransferase [EC:2.1.1.176]
CP005080.1_976	K00604	267.47	344.3	4.9e-103	methionyl-tRNA formyltransferase [EC:2.1.2.9]
CP005080.1_978	K04066	138.70	646.7	2.6e-194	primosomal protein N' (replication factor Y) (superfamily II helicase) [EC:5.6.2.4]
CP005080.1_979	K00789	19.03	619.9	1.7e-186	S-adenosylmethionine synthetase [EC:2.5.1.6]
CP005080.1_980	K13038	251.03	589.2	2.2e-177	phosphopantothenoylcysteine decarboxylase / phosphopantothenate---cysteine ligase [EC:4.1.1.36 6.3.2.5]
CP005080.1_981	K03060	24.57	98.3	2.2e-28	DNA-directed RNA polymerase subunit omega [EC:2.7.7.6]
CP005080.1_982	K00942	178.33	249.1	2.4e-74	guanylate kinase [EC:2.7.4.8]
CP005080.1_984	K01591	129.83	213.0	3.4e-63	orotidine-5'-phosphate decarboxylase [EC:4.1.1.23]
CP005080.1_985	K00254	173.83	494.9	1.4e-148	dihydroorotate dehydrogenase [EC:1.3.5.2]
CP005080.1_986	K01955	1479.70	1721.5	0	carbamoyl-phosphate synthase large subunit [EC:6.3.5.5]
CP005080.1_987	K01956	530.93	690.5	1.8e-207	carbamoyl-phosphate synthase small subunit [EC:6.3.5.5]
CP005080.1_989	K01465	336.03	470.2	5.2e-141	dihydroorotase [EC:3.5.2.3]
CP005080.1_990	K00609	254.20	485.5	5.6e-146	aspartate carbamoyltransferase catalytic subunit [EC:2.1.3.2]
CP005080.1_991	K02825	96.10	313.4	4.6e-94	pyrimidine operon attenuation protein / uracil phosphoribosyltransferase [EC:2.4.2.9]
CP005080.1_993	K03625	88.60	155.2	1e-45	transcription antitermination protein NusB
CP005080.1_994	K02356	149.23	299.7	1.1e-89	elongation factor P
CP005080.1_997	K03786	187.37	219.4	3e-65	3-dehydroquinate dehydratase II [EC:4.2.1.10]
CP005080.1_998	K13829	515.30	668.9	3e-201	shikimate kinase / 3-dehydroquinate synthase [EC:2.7.1.71 4.2.3.4]
CP005080.1_998	K01735	305.30	452.3	1e-135	3-dehydroquinate synthase [EC:4.2.3.4]
CP005080.1_998	K00891	128.77	194.3	1.8e-57	shikimate kinase [EC:2.7.1.71]
CP005080.1_999	K01736	306.20	509.1	9.2e-153	chorismate synthase [EC:4.2.3.5]
CP005080.1_1001	K07082	42.70	360.4	4.2e-108	peptidoglycan lytic transglycosylase G [EC:4.2.2.29]
CP005080.1_1002	K07447	43.93	192.1	5.3e-57	putative pre-16S rRNA nuclease [EC:3.1.-.-]
CP005080.1_1003	K01872	199.53	1055.3	1.6e-317	alanyl-tRNA synthetase [EC:6.1.1.7]
CP005080.1_1007	K02986	67.73	165.0	1.3e-48	small subunit ribosomal protein S4
CP005080.1_1009	K07478	187.97	684.2	9.9e-206	putative ATPase
CP005080.1_1013	K01892	275.30	348.3	2.9e-104	histidyl-tRNA synthetase [EC:6.1.1.21]
CP005080.1_1014	K01069	139.83	176.9	2.6e-52	hydroxyacylglutathione hydrolase [EC:3.1.2.6]
CP005080.1_1017	K01139	801.03	1179.3	0	GTP diphosphokinase / guanosine-3',5'-bis(diphosphate) 3'-diphosphatase [EC:2.7.6.5 3.1.7.2]
CP005080.1_1019	K00759	92.70	242.4	2.4e-72	adenine phosphoribosyltransferase [EC:2.4.2.7]
CP005080.1_1020	K03074	325.10	375.8	7.9e-113	preprotein translocase subunit SecF
CP005080.1_1021	K03072	438.53	497.8	1e-149	preprotein translocase subunit SecD
CP005080.1_1022	K03210	32.57	120.4	4.6e-35	preprotein translocase subunit YajC
CP005080.1_1023	K03551	186.47	587.2	9.9e-177	holliday junction DNA helicase RuvB [EC:5.6.2.4]
CP005080.1_1024	K03550	46.37	238.6	3.4e-71	holliday junction DNA helicase RuvA
CP005080.1_1025	K01159	32.43	289.6	1.6e-86	crossover junction endodeoxyribonuclease RuvC [EC:3.1.21.10]
CP005080.1_1026	K18189	91.27	220.5	1.2e-65	translational activator of cytochrome c oxidase 1
CP005080.1_1027	K08681	77.37	314.4	2.1e-94	pyridoxal 5'-phosphate synthase pdxT subunit [EC:4.3.3.6]
CP005080.1_1028	K06215	123.87	548.6	2.4e-165	pyridoxal 5'-phosphate synthase pdxS subunit [EC:4.3.3.6]
CP005080.1_1029	K03744	30.13	40.8	7.4e-11	LemA protein
CP005080.1_1030	K08256	249.30	618.6	5.1e-186	phosphatidyl-myo-inositol alpha-mannosyltransferase [EC:2.4.1.345]
CP005080.1_1031	K22311	200.50	450.8	1.3e-135	phosphatidylinositol dimannoside acyltransferase [EC:2.3.1.265]
CP005080.1_1032	K27681	191.30	307.1	3.5e-92	phosphatidylinositol phosphate synthase [EC:2.7.8.-]
CP005080.1_1033	K02355	521.90	627.6	1.1e-188	elongation factor G
CP005080.1_1035	K19710	136.70	326.4	4.7e-98	ATP adenylyltransferase [EC:2.7.7.53]
CP005080.1_1037	K01868	151.50	853.5	8.8e-257	threonyl-tRNA synthetase [EC:6.1.1.3]
CP005080.1_1040	K02342	101.50	137.0	3.1e-40	DNA polymerase III subunit epsilon [EC:2.7.7.7]
CP005080.1_1047	K15520	99.47	100.0	7.3e-29	mycothiol synthase [EC:2.3.1.189]
CP005080.1_1048	K02619	196.63	200.4	2.3e-59	4-amino-4-deoxychorismate lyase [EC:4.1.3.38]
CP005080.1_1050	K09981	36.93	86.2	5.3e-25	uncharacterized protein
CP005080.1_1053	K12132	224.03	250.3	9.6e-75	eukaryotic-like serine/threonine-protein kinase [EC:2.7.11.1]
CP005080.1_1055	K02302	420.43	518.1	1e-155	uroporphyrin-III C-methyltransferase / precorrin-2 dehydrogenase / sirohydrochlorin ferrochelatase [EC:2.1.1.107 1.3.1.76 4.99.1.4]
CP005080.1_1055	K02303	255.53	344.6	3.1e-103	uroporphyrin-III C-methyltransferase [EC:2.1.1.107]
CP005080.1_1056	K00768	85.40	736.9	5.4e-222	nicotinate-nucleotide--dimethylbenzimidazole phosphoribosyltransferase [EC:2.4.2.21]
CP005080.1_1056	K04719	217.37	310.7	8.3e-93	5,6-dimethylbenzimidazole synthase [EC:1.13.11.79]
CP005080.1_1057	K00595	224.23	336.1	1.5e-100	precorrin-6B C5,15-methyltransferase / cobalt-precorrin-6B C5,C15-methyltransferase [EC:2.1.1.132 2.1.1.289 2.1.1.196]

CP005080.1_1059	K02073	57.57	383.9	5.3e-115	D-methionine transport system substrate-binding protein
CP005080.1_1060	K02072	119.67	341.7	1.2e-102	D-methionine transport system permease protein
CP005080.1_1061	K02071	332.87	498.7	6.9e-150	D-methionine transport system ATP-binding protein
CP005080.1_1066	K01126	152.53	233.0	1.8e-69	glycerophosphoryl diester phosphodiesterase [EC:3.1.4.46]
CP005080.1_1067	K00655	122.40	242.1	4e-72	1-acyl-sn-glycerol-3-phosphate acyltransferase [EC:2.3.1.51]
CP005080.1_1068	K08167	383.90	723.8	7.2e-218	MFS transporter, DHA2 family, multidrug resistance protein
CP005080.1_1069	K18294	129.40	259.8	5e-78	TetR/AcrR family transcriptional regulator, mexCD-oprJ operon repressor
CP005080.1_1070	K21267	666.47	825.9	3.9e-249	rifampicin monooxygenase [EC:1.14.13.211]
CP005080.1_1073	K01755	169.37	702.2	4.2e-211	argininosuccinate lyase [EC:4.3.2.1]
CP005080.1_1074	K01940	115.50	583.4	2.8e-175	argininosuccinate synthase [EC:6.3.4.5]
CP005080.1_1078	K03402	48.50	243.3	9.1e-73	transcriptional regulator of arginine metabolism
CP005080.1_1079	K00821	483.53	596.6	1.4e-179	acetylornithine/N-succinyldiaminopimelate aminotransferase [EC:2.6.1.11 2.6.1.17]
CP005080.1_1080	K00930	215.57	463.9	1.4e-139	acetylglutamate kinase [EC:2.7.2.8]
CP005080.1_1081	K00620	170.47	518.3	1.1e-155	glutamate N-acetyltransferase / amino-acid N-acetyltransferase [EC:2.3.1.35 2.3.1.1]
CP005080.1_1082	K00145	128.50	446.5	3.3e-134	N-acetyl-gamma-glutamyl-phosphate reductase [EC:1.2.1.38]
CP005080.1_1084	K03453	223.63	246.5	2.3e-73	bile acid:Na+ symporter, BASS family
CP005080.1_1091	K03768	202.90	209.0	3.6e-62	peptidyl-prolyl cis-trans isomerase B (cyclophilin B) [EC:5.2.1.8]
CP005080.1_1098	K00074	404.50	426.3	7e-128	3-hydroxybutyryl-CoA dehydrogenase [EC:1.1.1.157]
CP005080.1_1101	K01890	122.80	623.4	2.5e-187	phenylalanyl-tRNA synthetase beta chain [EC:6.1.1.20]
CP005080.1_1102	K01889	95.27	371.8	2.6e-111	phenylalanyl-tRNA synthetase alpha chain [EC:6.1.1.20]
CP005080.1_1104	K03437	214.63	311.8	2.3e-93	RNA methyltransferase, TrmH family
CP005080.1_1105	K02887	45.33	198.5	6.2e-59	large subunit ribosomal protein L20
CP005080.1_1106	K02916	23.07	56.7	1.1e-15	large subunit ribosomal protein L35
CP005080.1_1107	K02520	34.97	202.2	4.5e-60	translation initiation factor IF-3
CP005080.1_1110	K14743	282.67	307.3	1e-91	membrane-anchored mycosin MYCP [EC:3.4.21.-]
CP005080.1_1118	K02001	326.57	1338.3	0	glycine betaine/proline transport system permease protein
CP005080.1_1118	K02002	122.57	354.4	5e-106	glycine betaine/proline transport system substrate-binding protein
CP005080.1_1119	K02000	415.90	615.6	5.9e-185	glycine betaine/proline transport system ATP-binding protein [EC:7.6.2.9]
CP005080.1_1121	K07229	152.23	158.4	7.7e-47	ferric-chelate reductase (NADPH) [EC:1.16.1.9]
CP005080.1_1125	K03088	96.50	138.6	1.1e-40	RNA polymerase sigma-70 factor, ECF subfamily
CP005080.1_1127	K01669	377.10	414.3	4.1e-124	deoxyribodipyrimidine photo-lyase [EC:4.1.99.3]
CP005080.1_1129	K00574	329.57	404.0	3e-121	cyclopropane-fatty-acyl-phospholipid synthase [EC:2.1.1.79]
CP005080.1_1130	K00574	329.57	500.8	1.4e-150	cyclopropane-fatty-acyl-phospholipid synthase [EC:2.1.1.79]
CP005080.1_1131	K09701	94.93	316.7	4.6e-95	uncharacterized protein
CP005080.1_1132	K06954	511.20	648.8	2.1e-195	uncharacterized protein
CP005080.1_1135	K03727	707.80	1492.6	0	ATP-dependent RNA helicase HelY [EC:5.6.2.6]
CP005080.1_1136	K07029	147.20	310.7	5.1e-93	diacylglycerol kinase (ATP) [EC:2.7.1.107]
CP005080.1_1137	K03118	86.40	304.2	6e-91	sec-independent protein translocase protein TatC
CP005080.1_1138	K03116	54.03	76.7	9.4e-22	sec-independent protein translocase protein TatA
CP005080.1_1141	K13573	239.87	419.7	5.4e-126	proteasome accessory factor C
CP005080.1_1142	K13572	215.13	413.4	4.4e-124	proteasome accessory factor B
CP005080.1_1143	K01802	113.30	123.9	4.3e-36	peptidylprolyl isomerase [EC:5.2.1.8]
CP005080.1_1145	K13571	501.60	784.6	2.3e-236	proteasome accessory factor A [EC:6.3.1.19]
CP005080.1_1147	K02529	268.37	288.3	5.1e-86	LacI family transcriptional regulator, galactose operon repressor
CP005080.1_1148	K03432	203.97	385.7	7.7e-116	proteasome alpha subunit [EC:3.4.25.1]
CP005080.1_1149	K03433	190.10	415.9	4.6e-125	proteasome beta subunit [EC:3.4.25.1]
CP005080.1_1150	K13570	31.83	88.2	1.8e-25	prokaryotic ubiquitin-like protein Pup
CP005080.1_1151	K20814	512.20	793.9	3.7e-239	Pup amidohydrolase [EC:3.5.1.119]
CP005080.1_1152	K13527	462.80	941.4	9.4e-284	proteasome-associated ATPase
CP005080.1_1153	K05337	42.27	79.6	8.8e-23	ferredoxin
CP005080.1_1155	K07442	95.33	380.1	8.8e-114	tRNA (adenine57-N1/adenine58-N1)-methyltransferase catalytic subunit [EC:2.1.1.219 2.1.1.220]
CP005080.1_1156	K06402	102.07	149.5	5.1e-44	stage IV sporulation protein FB [EC:3.4.24.-]
CP005080.1_1157	K07465	129.57	454.1	1.4e-136	putative RecB family exonuclease
CP005080.1_1159	K02035	249.67	300.5	1.1e-89	peptide/nickel transport system substrate-binding protein
CP005080.1_1161	K00548	263.53	1219.0	0	5-methyltetrahydrofolate--homocysteine methyltransferase [EC:2.1.1.13]
CP005080.1_1163	K02440	275.10	365.6	9.8e-110	glycerol uptake facilitator

CP005080.1_1164	K00864	510.13	835.4	1.2e-251	glycerol kinase [EC:2.7.1.30]
CP005080.1_1165	K00111	151.87	456.3	6.4e-137	glycerol-3-phosphate dehydrogenase [EC:1.1.5.3]
CP005080.1_1169	K15526	422.90	712.6	1.3e-214	L-cysteine:1D-myo-inositol 2-amino-2-deoxy-alpha-D-glucopyranoside ligase [EC:6.3.1.13]
CP005080.1_1173	K03284	165.70	418.1	2.2e-125	magnesium transporter
CP005080.1_1175	K00320	221.33	234.4	1.1e-69	5,10-methylenetetrahydromethanopterin reductase [EC:1.5.98.2]
CP005080.1_1184	K06177	231.00	315.6	2.1e-94	tRNA pseudouridine32 synthase / 23S rRNA pseudouridine746 synthase [EC:5.4.99.28 5.4.99.29]
CP005080.1_1185	K01069	139.83	146.2	5.5e-43	hydroxyacylglutathione hydrolase [EC:3.1.2.6]
CP005080.1_1186	K00153	387.43	693.1	3.6e-209	S-(hydroxymethyl)mycothiol dehydrogenase [EC:1.1.1.306]
CP005080.1_1187	K19776	192.53	192.9	2.1e-57	GntR family transcriptional regulator, galactonate operon transcriptional repressor
CP005080.1_1188	K00851	59.63	256.6	8.8e-77	gluconokinase [EC:2.7.1.12]
CP005080.1_1189	K03299	88.80	563.0	2.1e-169	gluconate:H+ symporter, GntP family
CP005080.1_1193	K07077	403.13	1020.4	9.6e-308	uncharacterized protein
CP005080.1_1195	K00389	46.53	146.7	3.3e-43	inner membrane protein YidH
CP005080.1_1197	K00344	279.00	311.0	6.5e-93	NADPH:quinone reductase [EC:1.6.5.5]
CP005080.1_1198	K01706	596.43	653.4	5.3e-197	glucarate dehydratase [EC:4.2.1.40]
CP005080.1_1200	K00249	382.07	471.2	2e-141	acyl-CoA dehydrogenase [EC:1.3.8.7]
CP005080.1_1201	K19702	277.27	668.9	2.4e-201	aminopeptidase S [EC:3.4.11.24]
CP005080.1_1202	K22108	142.27	149.2	6.5e-44	TetR/AcrR family transcriptional regulator, cholesterol catabolism regulator
CP005080.1_1202	K09017	119.20	124.3	4.2e-36	TetR/AcrR family transcriptional regulator
CP005080.1_1209	K13639	104.47	286.0	9e-86	MerR family transcriptional regulator, redox-sensitive transcriptional activator SoxR
CP005080.1_1211	K11963	286.53	299.2	5.8e-90	urea transport system ATP-binding protein
CP005080.1_1212	K11962	324.20	372.9	9.7e-112	urea transport system ATP-binding protein
CP005080.1_1213	K11961	295.13	426.3	5e-128	urea transport system permease protein
CP005080.1_1214	K11960	261.53	438.9	1.5e-131	urea transport system permease protein
CP005080.1_1214	K01997	149.83	256.6	1.8e-76	branched-chain amino acid transport system permease protein
CP005080.1_1215	K11959	254.37	577.7	6.1e-174	urea transport system substrate-binding protein
CP005080.1_1215	K01999	141.80	219.7	3.7e-65	branched-chain amino acid transport system substrate-binding protein
CP005080.1_1217	K11959	254.37	386.9	4.7e-116	urea transport system substrate-binding protein
CP005080.1_1217	K01999	141.80	151.3	2.1e-44	branched-chain amino acid transport system substrate-binding protein
CP005080.1_1220	K00249	382.07	437.4	3.6e-131	acyl-CoA dehydrogenase [EC:1.3.8.7]
CP005080.1_1223	K22468	274.30	510.6	1.5e-153	polyphosphate kinase [EC:2.7.4.34]
CP005080.1_1228	K03300	129.87	702.2	1.1e-211	citrate-Mg2+:H+ or citrate-Ca2+:H+ symporter, CitMHS family
CP005080.1_1231	K00451	47.47	700.8	4.6e-211	homogentisate 1,2-dioxygenase [EC:1.13.11.5]
CP005080.1_1234	K18289	445.63	631.5	2.3e-190	itaconate CoA-transferase [EC:2.8.3.- 2.8.3.22]
CP005080.1_1242	K07793	64.27	688.4	4.8e-207	putative tricarboxylic transport membrane protein
CP005080.1_1243	K07794	50.90	118.2	1.6e-34	putative tricarboxylic transport membrane protein
CP005080.1_1244	K07795	76.40	348.6	2.4e-104	putative tricarboxylic transport membrane protein
CP005080.1_1248	K07171	19.10	44.3	8.1e-12	mRNA interferase MazF [EC:3.1.-.-]
CP005080.1_1257	K03933	237.30	477.8	1.2e-143	chitin-binding protein
CP005080.1_1258	K01046	52.07	52.3	2.2e-14	triacylglycerol lipase [EC:3.1.1.3]
CP005080.1_1260	K14162	1024.53	1211.8	0	error-prone DNA polymerase [EC:2.7.7.7]
CP005080.1_1264	K18547	423.13	580.4	1.2e-174	streptogrisin D [EC:3.4.21.-]
CP005080.1_1268	K06871	174.27	236.5	2.5e-70	uncharacterized protein
CP005080.1_1271	K18545	376.10	476.0	3.6e-143	streptogrisin B [EC:3.4.21.81]
CP005080.1_1272	K09164	81.67	310.6	6.9e-93	uncharacterized protein
CP005080.1_1275	K02030	76.77	125.2	1.4e-36	polar amino acid transport system substrate-binding protein
CP005080.1_1276	K02028	386.70	471.9	9.7e-142	polar amino acid transport system ATP-binding protein [EC:7.4.2.1]
CP005080.1_1277	K02029	214.53	287.3	8.4e-86	polar amino acid transport system permease protein
CP005080.1_1278	K25562	450.07	527.9	1.7e-158	L-aspartate N-monooxygenase (nitrosuccinate-forming) [EC:1.14.13.248]
CP005080.1_1279	K02029	214.53	300.2	1.1e-89	polar amino acid transport system permease protein
CP005080.1_1280	K02030	76.77	141.5	1.6e-41	polar amino acid transport system substrate-binding protein
CP005080.1_1282	K09772	38.13	149.3	4.3e-44	cell division inhibitor SepF
CP005080.1_1283	K00252	509.73	656.6	2.3e-197	glutaryl-CoA dehydrogenase [EC:1.3.8.6]
CP005080.1_1284	K08167	383.90	723.9	6.4e-218	MFS transporter, DHA2 family, multidrug resistance protein
CP005080.1_1288	K00712	180.83	198.2	9.4e-59	poly(glycerol-phosphate) alpha-glucosyltransferase [EC:2.4.1.52]
CP005080.1_1291	K03977	233.47	672.1	2.2e-202	GTPase

CP005080.1_1292	K00655	122.40	155.5	8.2e-46	1-acyl-sn-glycerol-3-phosphate acyltransferase [EC:2.3.1.51]
CP005080.1_1293	K00945	42.83	295.7	2.2e-88	CMP/dCMP kinase [EC:2.7.4.25]
CP005080.1_1294	K04517	224.97	295.3	3.8e-88	prephenate dehydrogenase [EC:1.3.1.12]
CP005080.1_1295	K06208	80.70	167.5	7.2e-50	chorismate mutase [EC:5.4.99.5]
CP005080.1_1297	K08987	33.40	157.7	1.4e-46	putative membrane protein
CP005080.1_1299	K07074	59.80	274.3	5.8e-82	uncharacterized protein
CP005080.1_1303	K06211	137.30	441.2	1.6e-132	HTH-type transcriptional regulator, transcriptional repressor of NAD biosynthesis genes [EC:2.7.7.1 2.7.1.22]
CP005080.1_1304	K03811	46.70	231.9	4e-69	nicotinamide mononucleotide transporter
CP005080.1_1305	K06178	253.10	341.3	3.9e-102	23S rRNA pseudouridine2605 synthase [EC:5.4.99.22]
CP005080.1_1306	K06024	48.53	265.7	2.1e-79	segregation and condensation protein B
CP005080.1_1307	K05896	40.37	334.1	3.7e-100	segregation and condensation protein A
CP005080.1_1309	K03496	131.53	342.5	1.8e-102	chromosome partitioning protein
CP005080.1_1310	K00259	287.10	593.7	7.4e-179	alanine dehydrogenase [EC:1.4.1.1]
CP005080.1_1312	K01554	208.57	304.8	2e-91	8-oxo-dGDP phosphatase [EC:3.6.1.58]
CP005080.1_1313	K01937	133.63	846.9	7.4e-255	CTP synthase [EC:6.3.4.2]
CP005080.1_1314	K22934	296.37	645.9	3.6e-194	alpha,alpha-trehalase [EC:3.2.1.28]
CP005080.1_1325	K13444	191.60	397.8	3.1e-119	formylglycine-generating enzyme [EC:1.8.3.7]
CP005080.1_1326	K02031	412.13	827.7	2.3e-249	peptide/nickel transport system ATP-binding protein
CP005080.1_1326	K02032	420.67	445.2	2.1e-133	peptide/nickel transport system ATP-binding protein
CP005080.1_1328	K02033	263.63	358.5	2.4e-107	peptide/nickel transport system permease protein
CP005080.1_1329	K02035	249.67	321.5	4.7e-96	peptide/nickel transport system substrate-binding protein
CP005080.1_1331	K22303	376.93	493.9	1.4e-148	alkyl sulfatase [EC:1.14.11.77]
CP005080.1_1332	K09684	188.00	510.1	4e-153	PucR family transcriptional regulator, purine catabolism regulatory protein
CP005080.1_1335	K03631	130.47	709.9	1.3e-213	DNA repair protein RecN (Recombination protein N)
CP005080.1_1336	K00858	38.63	225.3	5.3e-67	NAD+ kinase [EC:2.7.1.23]
CP005080.1_1337	K06442	67.40	408.5	1.2e-122	23S rRNA (cytidine1920-2'-O)/16S rRNA (cytidine1409-2'-O)-methyltransferase [EC:2.1.1.226 2.1.1.227]
CP005080.1_1340	K23188	469.87	533.7	2.9e-160	iron-siderophore transport system ATP-binding protein [EC:7.2.2.17 7.2.2.-]
CP005080.1_1340	K02013	238.30	350.1	7.8e-105	iron complex transport system ATP-binding protein [EC:7.2.2.-]
CP005080.1_1341	K06117	279.40	498.0	1.6e-149	glycerol-1-phosphatase [EC:3.1.3.21]
CP005080.1_1345	K03652	52.00	250.5	7.2e-75	DNA-3-methyladenine glycosylase [EC:3.2.2.21]
CP005080.1_1346	K06377	63.37	340.8	3.1e-102	sporulation-control protein
CP005080.1_1349	K07090	37.03	103.0	7.6e-30	uncharacterized protein
CP005080.1_1352	K02013	238.30	242.6	3.1e-72	iron complex transport system ATP-binding protein [EC:7.2.2.-]
CP005080.1_1358	K07568	161.50	381.6	1.7e-114	S-adenosylmethionine:tRNA ribosyltransferase-isomerase [EC:2.4.99.17]
CP005080.1_1360	K21397	347.03	1089.7	0	ABC transport system ATP-binding/permease protein
CP005080.1_1361	K01079	145.33	364.9	4.5e-109	phosphoserine phosphatase [EC:3.1.3.3]
CP005080.1_1362	K08296	84.07	193.5	2.9e-57	phosphohistidine phosphatase [EC:3.1.3.-]
CP005080.1_1365	K03449	127.83	559.5	3.2e-168	MFS transporter, CP family, cyanate transporter
CP005080.1_1368	K11611	284.47	431.9	3.5e-130	meromycolic acid enoyl-[acyl-carrier protein] reductase [EC:1.3.1.118]
CP005080.1_1368	K00208	269.47	269.9	1.4e-80	enoyl-[acyl-carrier protein] reductase I [EC:1.3.1.9 1.3.1.10]
CP005080.1_1369	K00059	269.80	332.4	1.6e-99	3-oxoacyl-[acyl-carrier protein] reductase [EC:1.1.1.100]
CP005080.1_1370	K03568	311.90	511.3	1.8e-153	TldD protein
CP005080.1_1372	K01866	88.87	397.7	4.4e-119	tyrosyl-tRNA synthetase [EC:6.1.1.1]
CP005080.1_1376	K03639	198.23	466.2	6.2e-140	GTP 3',8-cyclase [EC:4.1.99.22]
CP005080.1_1377	K14393	287.90	704.1	5.8e-212	cation/acetate symporter
CP005080.1_1379	K20486	286.50	373.0	1.1e-111	lantibiotic leader peptide-processing serine protease [EC:3.4.21.-]
CP005080.1_1381	K18575	103.13	111.8	1.5e-32	alkenylglycerophosphocholine hydrolase [EC:3.3.2.2]
CP005080.1_1382	K15537	201.77	315.6	2.4e-94	alkylglycerol monooxygenase [EC:1.14.16.5]
CP005080.1_1384	K02342	101.50	188.7	6.3e-56	DNA polymerase III subunit epsilon [EC:2.7.7.7]
CP005080.1_1386	K14998	36.50	149.8	5.4e-44	surfeit locus 1 family protein
CP005080.1_1387	K22934	296.37	641.1	1e-192	alpha,alpha-trehalase [EC:3.2.1.28]
CP005080.1_1398	K07054	48.17	332.7	9.8e-100	uncharacterized protein
CP005080.1_1402	K01628	215.43	285.1	3.6e-85	L-fuculose-phosphate aldolase [EC:4.1.2.17]
CP005080.1_1403	K03306	385.20	533.1	3e-160	inorganic phosphate transporter, PiT family
CP005080.1_1405	K02227	90.90	399.6	5.8e-120	adenosylcobinamide-phosphate synthase [EC:6.3.1.10]
CP005080.1_1406	K02232	242.13	782.7	2.1e-235	adenosylcobyric acid synthase [EC:6.3.5.10]

CP005080.1_1407	K03404	479.33	895.7	8.4e-270	magnesium chelatase subunit D [EC:6.6.1.1]
CP005080.1_1407	K03405	224.73	467.9	1.6e-140	magnesium chelatase subunit I [EC:6.6.1.1]
CP005080.1_1408	K19221	120.03	239.9	1.4e-71	cob(I)alamin adenosyltransferase [EC:2.5.1.17]
CP005080.1_1409	K02224	108.67	683.0	1.3e-205	cobyrinic acid a,c-diamide synthase [EC:6.3.5.9 6.3.5.11]
CP005080.1_1410	K07238	128.17	140.7	2.6e-41	zinc transporter, ZIP family
CP005080.1_1411	K00817	293.33	297.1	1.1e-88	histidinol-phosphate aminotransferase [EC:2.6.1.9]
CP005080.1_1411	K02189	39.97	143.9	2.6e-42	cobalt-precorrin 5A hydrolase [EC:3.7.1.12]
CP005080.1_1415	K06718	104.57	268.0	2.6e-80	L-2,4-diaminobutyric acid acetyltransferase [EC:2.3.1.178]
CP005080.1_1416	K00836	439.63	642.5	2.7e-193	diaminobutyrate-2-oxoglutarate transaminase [EC:2.6.1.76]
CP005080.1_1417	K06720	84.00	197.4	3.8e-59	L-ectoine synthase [EC:4.2.1.108]
CP005080.1_1418	K10674	318.53	465.6	2.5e-140	ectoine hydroxylase [EC:1.14.11.55]
CP005080.1_1420	K10680	514.00	568.1	9.9e-171	N-ethylmaleimide reductase [EC:1.-.-.-]
CP005080.1_1421	K23775	99.10	109.9	8.1e-32	MarR family transcriptional regulator, organic hydroperoxide resistance regulator
CP005080.1_1425	K09702	62.87	140.8	1.7e-41	uncharacterized protein
CP005080.1_1426	K14519	647.33	760.5	5.1e-229	NADP-dependent aldehyde dehydrogenase [EC:1.2.1.4]
CP005080.1_1431	K05364	378.67	585.4	7.2e-176	penicillin-binding protein A
CP005080.1_1432	K06901	93.90	504.8	1.1e-151	adenine/guanine/hypoxanthine permease
CP005080.1_1434	K04091	325.40	648.2	5.7e-195	alkanesulfonate monooxygenase [EC:1.14.14.5 1.14.14.34]
CP005080.1_1436	K00299	91.57	176.3	4e-52	FMN reductase [EC:1.5.1.38]
CP005080.1_1438	K17228	340.70	621.6	1.8e-187	dimethylsulfone monooxygenase [EC:1.14.14.35]
CP005080.1_1441	K01707	248.40	446.7	1.1e-134	5-dehydro-4-deoxyglucarate dehydratase [EC:4.2.1.41]
CP005080.1_1443	K05792	212.93	255.5	2.9e-76	tellurite resistance protein TerA
CP005080.1_1443	K05791	193.43	196.0	1.8e-58	tellurium resistance protein TerZ
CP005080.1_1446	K12132	224.03	226.7	1.4e-67	eukaryotic-like serine/threonine-protein kinase [EC:2.7.11.1]
CP005080.1_1447	K12132	224.03	255.0	3.5e-76	eukaryotic-like serine/threonine-protein kinase [EC:2.7.11.1]
CP005080.1_1451	K01714	259.73	308.6	3.2e-92	4-hydroxy-tetrahydrodipicolinate synthase [EC:4.3.3.7]
CP005080.1_1452	K00674	156.17	470.2	3e-141	2,3,4,5-tetrahydropyridine-2,6-dicarboxylate N-succinyltransferase [EC:2.3.1.117]
CP005080.1_1455	K23778	108.13	218.7	4e-65	TetR/AcrR family transcriptional regulator, regulator of biofilm formation and stress response
CP005080.1_1456	K03297	108.13	149.5	5.4e-44	small multidrug resistance pump
CP005080.1_1460	K04488	144.00	205.2	4.1e-61	nitrogen fixation protein NifU and related proteins
CP005080.1_1461	K11717	566.53	684.8	4.5e-206	cysteine desulfurase / selenocysteine lyase [EC:2.8.1.7 4.4.1.16]
CP005080.1_1462	K09013	161.43	405.1	6.2e-122	Fe-S cluster assembly ATP-binding protein
CP005080.1_1463	K05710	109.17	164.2	1.2e-48	3-phenylpropionate/trans-cinnamate dioxygenase ferredoxin component
CP005080.1_1464	K09015	272.00	417.1	4.4e-125	Fe-S cluster assembly protein SufD
CP005080.1_1465	K09014	377.87	910.1	2.4e-274	Fe-S cluster assembly protein SufB
CP005080.1_1467	K01990	262.37	312.0	2.8e-93	ABC-2 type transport system ATP-binding protein
CP005080.1_1469	K02259	62.60	195.5	4.6e-58	heme a synthase [EC:1.17.99.9]
CP005080.1_1471	K02257	126.90	332.1	2.3e-99	heme o synthase [EC:2.5.1.141]
CP005080.1_1472	K00615	214.20	554.9	1.3e-166	transketolase [EC:2.2.1.1]
CP005080.1_1473	K00616	134.00	257.8	1e-76	transaldolase [EC:2.2.1.2]
CP005080.1_1474	K00036	396.33	683.3	1.6e-205	glucose-6-phosphate 1-dehydrogenase [EC:1.1.1.49 1.1.1.363]
CP005080.1_1476	K01057	120.43	294.5	7e-88	6-phosphogluconolactonase [EC:3.1.1.31]
CP005080.1_1479	K08981	123.43	339.4	1.5e-101	putative membrane protein
CP005080.1_1480	K09167	107.60	156.2	5e-46	uncharacterized protein
CP005080.1_1481	K01810	196.17	937.3	4.3e-282	glucose-6-phosphate isomerase [EC:5.3.1.9]
CP005080.1_1483	K03075	26.13	62.6	1.8e-17	preprotein translocase subunit SecG
CP005080.1_1484	K01803	24.57	332.4	1.2e-99	triosephosphate isomerase (TIM) [EC:5.3.1.1]
CP005080.1_1485	K00927	104.93	558.6	5.1e-168	phosphoglycerate kinase [EC:2.7.2.3]
CP005080.1_1486	K00134	414.10	512.3	5.8e-154	glyceraldehyde 3-phosphate dehydrogenase (phosphorylating) [EC:1.2.1.12]
CP005080.1_1487	K09762	93.53	411.1	1.6e-123	cell division protein WhiA
CP005080.1_1489	K06958	140.17	477.8	8.8e-144	RNase adapter protein RapZ
CP005080.1_1490	K03703	236.70	950.0	3.2e-286	excinuclease ABC subunit C
CP005080.1_1495	K03701	341.57	1458.4	0	excinuclease ABC subunit A
CP005080.1_1496	K16163	65.57	278.2	3.5e-83	maleylpyruvate isomerase [EC:5.2.1.4]
CP005080.1_1500	K05791	193.43	302.7	4.3e-91	tellurium resistance protein TerZ
CP005080.1_1501	K03702	343.33	1137.1	0	excinuclease ABC subunit B

CP005080.1_1503	K01126	152.53	222.5	2.7e-66	glycerophosphoryl diester phosphodiesterase [EC:3.1.4.46]
CP005080.1_1504	K00567	174.97	184.1	1.8e-54	methylated-DNA-[protein]-cysteine S-methyltransferase [EC:2.1.1.63]
CP005080.1_1506	K06995	49.53	96.0	1e-27	uncharacterized protein
CP005080.1_1507	K16329	279.93	432.8	3.9e-130	pseudouridylate synthase [EC:4.2.1.70]
CP005080.1_1511	K14260	327.23	741.9	3.5e-223	alanine-synthesizing transaminase [EC:2.6.1.66 2.6.1.2]
CP005080.1_1519	K00859	53.27	293.5	1.6e-87	dephospho-CoA kinase [EC:2.7.1.24]
CP005080.1_1522	K02945	147.87	573.7	1.8e-172	small subunit ribosomal protein S1
CP005080.1_1524	K03579	572.67	1169.8	0	ATP-dependent RNA helicase HrpB [EC:5.6.2.6]
CP005080.1_1528	K07038	27.13	32.5	2.1e-08	inner membrane protein
CP005080.1_1529	K02335	481.67	1083.1	0	DNA polymerase I [EC:2.7.7.7]
CP005080.1_1531	K19222	83.07	138.1	1.2e-40	1,4-dihydroxy-2-naphthoyl-CoA hydrolase [EC:3.1.2.28]
CP005080.1_1532	K01999	141.80	284.1	1.1e-84	branched-chain amino acid transport system substrate-binding protein
CP005080.1_1533	K01997	149.83	331.7	3.2e-99	branched-chain amino acid transport system permease protein
CP005080.1_1534	K01998	245.40	351.9	2.2e-105	branched-chain amino acid transport system permease protein
CP005080.1_1535	K01995	319.03	449.7	8.7e-135	branched-chain amino acid transport system ATP-binding protein
CP005080.1_1536	K01996	317.37	397.4	3.7e-119	branched-chain amino acid transport system ATP-binding protein
CP005080.1_1537	K22010	136.53	276.2	1.3e-82	two-component system, response regulator PdtaR
CP005080.1_1539	K00873	49.90	617.9	8.2e-186	pyruvate kinase [EC:2.7.1.40]
CP005080.1_1540	K09807	88.93	151.7	1.3e-44	uncharacterized protein
CP005080.1_1541	K01119	358.37	665.1	5.1e-200	2',3'-cyclic-nucleotide 2'-phosphodiesterase / 3'-nucleotidase [EC:3.1.4.16 3.1.3.6]
CP005080.1_1542	K27501	461.57	708.5	1.3e-213	putrescine N-hydroxylase [EC:1.14.13.252]
CP005080.1_1545	K03321	345.37	453.2	6.2e-136	sulfate permease, SulP family
CP005080.1_1547	K06193	38.80	99.9	4.2e-29	protein PhnA
CP005080.1_1549	K01256	435.67	869.3	2.4e-261	aminopeptidase N [EC:3.4.11.2]
CP005080.1_1551	K04092	86.50	203.3	2e-60	chorismate mutase [EC:5.4.99.5]
CP005080.1_1553	K12308	328.20	440.4	5.7e-132	beta-galactosidase [EC:3.2.1.23]
CP005080.1_1557	K06996	53.13	146.5	6.2e-43	uncharacterized protein
CP005080.1_1560	K01039	171.33	239.2	3.5e-71	glutaconate CoA-transferase, subunit A [EC:2.8.3.12]
CP005080.1_1561	K01040	120.73	217.1	9.4e-65	glutaconate CoA-transferase, subunit B [EC:2.8.3.12]
CP005080.1_1562	K00459	256.27	279.8	2.6e-83	nitronate monooxygenase [EC:1.13.12.16]
CP005080.1_1564	K03704	80.73	122.2	8.6e-36	cold shock protein
CP005080.1_1566	K22108	142.27	325.1	2e-97	TetR/AcrR family transcriptional regulator, cholesterol catabolism regulator
CP005080.1_1567	K00059	269.80	270.7	8.7e-81	3-oxoacyl-[acyl-carrier protein] reductase [EC:1.1.1.100]
CP005080.1_1571	K09975	44.63	203.6	1.7e-60	uncharacterized protein
CP005080.1_1573	K00266	509.03	625.5	4e-188	glutamate synthase (NADPH) small chain [EC:1.4.1.13]
CP005080.1_1574	K00265	1712.27	2389.4	0	glutamate synthase (NADPH) large chain [EC:1.4.1.13]
CP005080.1_1575	K22736	39.70	253.4	1.6e-75	vacuolar iron transporter family protein
CP005080.1_1580	K00852	237.13	399.4	1.2e-119	ribokinase [EC:2.7.1.15]
CP005080.1_1582	K01644	132.63	301.0	6e-90	citrate lyase subunit beta / citryl-CoA lyase [EC:4.1.3.34]
CP005080.1_1583	K13292	50.50	385.2	1.1e-115	phosphatidylglycerol---prolipoprotein diacylglyceryl transferase [EC:2.5.1.145]
CP005080.1_1585	K01695	85.97	338.2	2.3e-101	tryptophan synthase alpha chain [EC:4.2.1.20]
CP005080.1_1586	K01696	625.47	812.8	2.6e-244	tryptophan synthase beta chain [EC:4.2.1.20]
CP005080.1_1588	K01609	315.40	354.1	6.3e-106	indole-3-glycerol phosphate synthase [EC:4.1.1.48]
CP005080.1_1592	K01657	465.93	719.6	1.3e-216	anthranilate synthase component I [EC:4.1.3.27]
CP005080.1_1593	K01496	194.93	246.0	2.9e-73	phosphoribosyl-AMP cyclohydrolase [EC:3.5.4.19]
CP005080.1_1598	K02500	339.30	458.3	1.4e-137	imidazole glycerol-phosphate synthase subunit HisF [EC:4.3.2.10]
CP005080.1_1600	K24017	280.60	381.8	1.7e-114	phosphoribosyl isomerase A [EC:5.3.1.16 5.3.1.24]
CP005080.1_1601	K02501	231.60	282.7	9.9e-85	imidazole glycerol-phosphate synthase subunit HisH [EC:4.3.2.10]
CP005080.1_1603	K01693	300.33	319.3	7.2e-96	imidazoleglycerol-phosphate dehydratase [EC:4.2.1.19]
CP005080.1_1604	K00817	293.33	419.7	8e-126	histidinol-phosphate aminotransferase [EC:2.6.1.9]
CP005080.1_1605	K00013	519.37	613.0	3e-184	histidinol dehydrogenase [EC:1.1.1.23]
CP005080.1_1608	K07157	140.47	216.6	1.7e-64	uncharacterized protein
CP005080.1_1609	K03976	99.40	245.3	4.3e-73	Cys-tRNA(Pro)/Cys-tRNA(Cys) deacylase [EC:3.1.1.-]
CP005080.1_1612	K01990	262.37	281.3	5.9e-84	ABC-2 type transport system ATP-binding protein
CP005080.1_1615	K02337	932.93	1546.0	0	DNA polymerase III subunit alpha [EC:2.7.7.7]
CP005080.1_1619	K01113	89.83	508.3	1.4e-152	alkaline phosphatase D [EC:3.1.3.1]

CP005080.1_1622	K24163	379.43	533.6	3.8e-160	monovalent cation/hydrogen antiporter
CP005080.1_1623	K02348	108.20	112.2	1e-32	ElaA protein
CP005080.1_1624	K06180	285.23	332.2	2.2e-99	23S rRNA pseudouridine1911/1915/1917 synthase [EC:5.4.99.23]
CP005080.1_1625	K03101	31.87	173.9	1.4e-51	signal peptidase II [EC:3.4.23.36]
CP005080.1_1626	K06204	35.73	211.4	1.1e-62	RNA polymerase-binding transcription factor
CP005080.1_1627	K01870	467.30	1086.1	0	isoleucyl-tRNA synthetase [EC:6.1.1.5]
CP005080.1_1628	K04074	47.10	68.7	2.2e-19	cell division initiation protein
CP005080.1_1629	K02221	21.77	50.9	6.7e-14	YggT family protein
CP005080.1_1630	K09772	38.13	198.0	5.1e-59	cell division inhibitor SepF
CP005080.1_1631	K06997	64.17	247.6	8.6e-74	PLP dependent protein
CP005080.1_1632	K05810	84.47	280.0	1.4e-83	purine-nucleoside/S-methyl-5'-thioadenosine phosphorylase / adenosine deaminase [EC:2.4.2.1 2.4.2.28 3.5.4.4]
CP005080.1_1633	K03531	142.77	575.8	2.3e-173	cell division protein FtsZ
CP005080.1_1634	K03589	42.57	146.2	7.6e-43	cell division protein FtsQ
CP005080.1_1635	K02563	193.67	431.6	1.7e-129	UDP-N-acetylglucosamine--N-acetylmuramyl-(pentapeptide) pyrophosphoryl-undecaprenol N-acetylglucosamine transferase [EC:2.4.1.227]
CP005080.1_1636	K03588	356.93	519.3	3.2e-156	cell division protein FtsW
CP005080.1_1637	K01925	363.80	569.6	2.7e-171	UDP-N-acetylmuramoylalanine--D-glutamate ligase [EC:6.3.2.9]
CP005080.1_1638	K01000	186.50	431.2	1.9e-129	phospho-N-acetylmuramoyl-pentapeptide-transferase [EC:2.7.8.13]
CP005080.1_1639	K01929	327.77	563.2	2.1e-169	UDP-N-acetylmuramoyl-tripeptide--D-alanyl-D-alanine ligase [EC:6.3.2.10]
CP005080.1_1640	K01928	408.67	641.3	6e-193	UDP-N-acetylmuramoyl-L-alanyl-D-glutamate--2,6-diaminopimelate ligase [EC:6.3.2.13]
CP005080.1_1641	K03587	463.30	706.9	1.2e-212	cell division protein FtsI (penicillin-binding protein 3) [EC:3.4.16.4]
CP005080.1_1642	K03586	43.53	43.7	1.3e-11	cell division protein FtsL
CP005080.1_1643	K03438	382.10	435.1	1.3e-130	16S rRNA (cytosine1402-N4)-methyltransferase [EC:2.1.1.199]
CP005080.1_1644	K01673	19.40	87.8	2.1e-25	carbonic anhydrase [EC:4.2.1.1]
CP005080.1_1645	K03924	113.70	483.6	3.1e-145	MoxR-like ATPase [EC:3.6.3.-]
CP005080.1_1647	K22452	104.63	226.8	2.7e-67	protein-glutamine gamma-glutamyltransferase [EC:2.3.2.13]
CP005080.1_1652	K01421	140.40	661.8	7.4e-199	putative membrane protein
CP005080.1_1653	K09017	119.20	119.7	1.1e-34	TetR/AcrR family transcriptional regulator
CP005080.1_1657	K00297	201.37	355.9	1.6e-106	methylenetetrahydrofolate reductase (NADH) [EC:1.5.1.54]
CP005080.1_1658	K00788	164.63	251.9	3.8e-75	thiamine-phosphate pyrophosphorylase [EC:2.5.1.3]
CP005080.1_1660	K00529	395.20	510.9	1.8e-153	3-phenylpropionate/trans-cinnamate dioxygenase ferredoxin reductase component [EC:1.18.1.3]
CP005080.1_1662	K03153	270.03	461.2	1.4e-138	glycine oxidase [EC:1.4.3.19]
CP005080.1_1663	K03154	35.33	80.4	4.2e-23	sulfur carrier protein
CP005080.1_1664	K03149	188.47	463.3	6.5e-139	thiazole synthase [EC:2.8.1.10]
CP005080.1_1665	K12132	224.03	320.0	6.5e-96	eukaryotic-like serine/threonine-protein kinase [EC:2.7.11.1]
CP005080.1_1665	K11912	105.07	166.0	5.4e-49	serine/threonine-protein kinase PpkA [EC:2.7.11.1]
CP005080.1_1666	K01151	120.17	351.1	3.2e-105	deoxyribonuclease IV [EC:3.1.21.2]
CP005080.1_1668	K03594	84.07	184.7	8.8e-55	bacterioferritin [EC:1.16.3.1]
CP005080.1_1669	K02192	60.67	95.4	1.3e-27	bacterioferritin-associated ferredoxin
CP005080.1_1670	K01626	124.93	496.0	7.1e-149	3-deoxy-7-phosphoheptulonate synthase [EC:2.5.1.54]
CP005080.1_1671	K13063	492.93	1014.3	8.1e-306	2-amino-4-deoxychorismate synthase [EC:2.6.1.86]
CP005080.1_1672	K03778	351.47	448.1	1.7e-134	D-lactate dehydrogenase [EC:1.1.1.28]
CP005080.1_1675	K00655	122.40	255.7	2.9e-76	1-acyl-sn-glycerol-3-phosphate acyltransferase [EC:2.3.1.51]
CP005080.1_1676	K03928	134.07	359.1	1.1e-107	carboxylesterase [EC:3.1.1.1]
CP005080.1_1679	K25026	274.23	372.2	1.4e-111	glucokinase [EC:2.7.1.2]
CP005080.1_1681	K01551	66.67	185.9	5.7e-55	arsenite/tail-anchored protein-transporting ATPase [EC:7.3.2.7 7.3.-.-]
CP005080.1_1683	K07096	83.70	113.2	5e-33	uncharacterized protein
CP005080.1_1684	K01897	430.97	537.1	2.8e-161	long-chain acyl-CoA synthetase [EC:6.2.1.3]
CP005080.1_1685	K13668	266.63	511.0	1.5e-153	phosphatidyl-myo-inositol dimannoside synthase [EC:2.4.1.346]
CP005080.1_1688	K21471	158.57	250.2	2.6e-74	peptidoglycan DL-endopeptidase CwlO [EC:3.4.-.-]
CP005080.1_1689	K21471	158.57	263.2	3.1e-78	peptidoglycan DL-endopeptidase CwlO [EC:3.4.-.-]
CP005080.1_1690	K06962	56.63	77.7	4.6e-22	uncharacterized protein
CP005080.1_1692	K07059	155.80	224.4	8.1e-67	rhomboid family protein
CP005080.1_1695	K00766	109.93	454.3	1.7e-136	anthranilate phosphoribosyltransferase [EC:2.4.2.18]
CP005080.1_1696	K03891	461.00	883.6	2.2e-266	quinol---cytochrome-c reductase cytochrome b subunit [EC:7.1.1.8]
CP005080.1_1697	K03890	179.53	467.1	1.8e-140	quinol---cytochrome c reductase iron-sulfur subunit [EC:7.1.1.8]
CP005080.1_1698	K03889	192.90	413.9	1.8e-124	quinol---cytochrome-c reductase cytochrome c subunit [EC:7.1.1.8]

CP005080.1_1699	K02276	184.90	234.3	8e-70	cytochrome c oxidase subunit III [EC:7.1.1.9]
CP005080.1_1702	K27109	124.73	174.2	4.2e-52	cytochrome c oxidase subunit IV [EC:7.1.1.9]
CP005080.1_1703	K02274	750.43	934.9	1.6e-281	cytochrome c oxidase subunit I [EC:7.1.1.9]
CP005080.1_1704	K02275	207.23	255.5	4.8e-76	cytochrome c oxidase subunit II [EC:7.1.1.9]
CP005080.1_1705	K04487	287.40	412.1	1.3e-123	cysteine desulfurase [EC:2.8.1.7]
CP005080.1_1705	K04085	48.00	75.3	1.5e-21	tRNA 2-thiouridine synthesizing protein A [EC:2.8.1.-]
CP005080.1_1710	K03517	105.40	404.3	2.6e-121	quinolinate synthase [EC:2.5.1.72]
CP005080.1_1712	K03296	1115.73	1337.8	0	hydrophobic/amphiphilic exporter-1 (mainly G- bacteria), HAE1 family
CP005080.1_1714	K07778	170.90	187.3	2e-55	two-component system, NarL family, sensor histidine kinase DesK [EC:2.7.13.3]
CP005080.1_1716	K03969	84.50	225.8	4.6e-67	phage shock protein A
CP005080.1_1721	K02231	39.03	217.7	1.1e-64	adenosylcobinamide kinase / adenosylcobinamide-phosphate guanylyltransferase [EC:2.7.1.156 2.7.7.62]
CP005080.1_1722	K00768	85.40	386.7	7.2e-116	nicotinate-nucleotide--dimethylbenzimidazole phosphoribosyltransferase [EC:2.4.2.21]
CP005080.1_1726	K02233	34.73	223.1	2.8e-66	adenosylcobinamide-GDP ribazoletransferase [EC:2.7.8.26]
CP005080.1_1727	K01255	422.07	598.2	9.3e-180	leucyl aminopeptidase [EC:3.4.11.1]
CP005080.1_1728	K00382	465.60	532.1	6.2e-160	dihydrolipoyl dehydrogenase [EC:1.8.1.4]
CP005080.1_1729	K00627	488.13	531.6	1.3e-159	pyruvate dehydrogenase E2 component (dihydrolipoyllysine-residue acetyltransferase) [EC:2.3.1.12]
CP005080.1_1731	K00163	290.30	1386.1	0	pyruvate dehydrogenase E1 component [EC:1.2.4.1]
CP005080.1_1735	K07071	116.47	411.0	4.2e-123	uncharacterized protein
CP005080.1_1738	K03801	234.97	305.8	1.9e-91	lipoyl(octanoyl) transferase [EC:2.3.1.181]
CP005080.1_1739	K03644	109.93	465.5	8.5e-140	lipoyl synthase [EC:2.8.1.8]
CP005080.1_1743	K01915	33.97	319.3	2.5e-95	glutamine synthetase [EC:6.3.1.2]
CP005080.1_1745	K02342	101.50	123.5	4e-36	DNA polymerase III subunit epsilon [EC:2.7.7.7]
CP005080.1_1749	K00537	80.33	125.6	9.5e-37	arsenate reductase (glutaredoxin) [EC:1.20.4.1]
CP005080.1_1751	K00375	343.30	596.5	2.1e-179	GntR family transcriptional regulator / MocR family aminotransferase
CP005080.1_1755	K01915	33.97	358.2	4.1e-107	glutamine synthetase [EC:6.3.1.2]
CP005080.1_1758	K06994	260.33	551.6	1.1e-165	putative drug exporter of the RND superfamily
CP005080.1_1764	K07044	90.60	371.9	1.4e-111	uncharacterized protein
CP005080.1_1769	K01200	408.90	881.0	4.1e-265	pullulanase [EC:3.2.1.41]
CP005080.1_1769	K01176	222.80	287.2	1.1e-85	alpha-amylase [EC:3.2.1.1]
CP005080.1_1770	K01176	222.80	411.6	2.2e-123	alpha-amylase [EC:3.2.1.1]
CP005080.1_1771	K02529	268.37	294.7	5.8e-88	LacI family transcriptional regulator, galactose operon repressor
CP005080.1_1772	K01187	286.07	383.7	8.1e-115	alpha-glucosidase [EC:3.2.1.20]
CP005080.1_1773	K15772	293.47	340.0	4.9e-102	arabinogalactan oligomer / maltooligosaccharide transport system permease protein
CP005080.1_1774	K02025	276.90	322.2	1.6e-96	multiple sugar transport system permease protein
CP005080.1_1775	K02027	193.17	274.2	1.1e-81	multiple sugar transport system substrate-binding protein
CP005080.1_1776	K02529	268.37	307.5	7.5e-92	LacI family transcriptional regulator, galactose operon repressor
CP005080.1_1781	K00982	237.27	1204.8	0	[glutamine synthetase] adenylyltransferase / [glutamine synthetase]-adenylyl-L-tyrosine phosphorylase [EC:2.7.7.42 2.7.7.89]
CP005080.1_1783	K02493	233.73	258.0	8e-77	release factor glutamine methyltransferase [EC:2.1.1.297]
CP005080.1_1785	K24699	97.17	110.6	2.9e-32	TetR/AcrR family transcriptional regulator, regulator of mycofactocin system
CP005080.1_1790	K07454	78.40	95.3	2.1e-27	putative restriction endonuclease
CP005080.1_1801	K01915	33.97	321.0	7.6e-96	glutamine synthetase [EC:6.3.1.2]
CP005080.1_1806	K22552	322.37	829.3	6.6e-250	multicopper oxidase [EC:1.16.3.1]
CP005080.1_1808	K19577	416.40	527.9	9.4e-159	MFS transporter, DHA1 family, inner membrane transport protein
CP005080.1_1809	K18353	128.17	481.6	9.6e-145	vancomycin resistance protein VanJ
CP005080.1_1811	K05557	639.00	683.6	6.2e-206	MFS transporter, DHA2 family, integral membrane protein
CP005080.1_1811	K08167	383.90	532.4	5.9e-160	MFS transporter, DHA2 family, multidrug resistance protein
CP005080.1_1812	K00606	82.33	448.7	6.3e-135	3-methyl-2-oxobutanoate hydroxymethyltransferase [EC:2.1.2.11]
CP005080.1_1813	K18232	464.53	515.7	2e-155	oleandomycin transport system ATP-binding protein
CP005080.1_1813	K01990	262.37	338.6	2.4e-101	ABC-2 type transport system ATP-binding protein
CP005080.1_1814	K18233	335.33	413.2	1.2e-124	oleandomycin transport system permease protein
CP005080.1_1814	K01992	16.23	22.9	7.8e-06	ABC-2 type transport system permease protein
CP005080.1_1816	K22894	131.70	270.2	1.4e-80	SARP family transcriptional regulator, regulator of embCAB operon
CP005080.1_1817	K06400	148.90	262.8	2.9e-78	site-specific DNA recombinase
CP005080.1_1821	K06988	161.77	201.1	1e-59	8-hydroxy-5-deazaflavin:NADPH oxidoreductase [EC:1.5.1.40]
CP005080.1_1823	K08225	199.57	310.7	6.1e-93	MFS transporter, ENTS family, enterobactin (siderophore) exporter
CP005080.1_1825	K01265	141.83	398.5	1.3e-119	methionyl aminopeptidase [EC:3.4.11.18]

CP005080.1_1826	K21480	248.47	272.2	2.2e-81	heme oxygenase (biliverdin-producing, ferredoxin) [EC:1.14.15.20]
CP005080.1_1827	K06998	103.40	104.2	2.9e-30	trans-2,3-dihydro-3-hydroxyanthranilate isomerase [EC:5.3.3.17]
CP005080.1_1830	K25132	229.57	361.7	1.2e-108	heme transport system substrate-binding protein
CP005080.1_1831	K25133	388.87	518.3	5e-156	heme transport system permease protein
CP005080.1_1831	K25027	379.47	403.9	2.7e-121	cobalamin transport system permease protein
CP005080.1_1831	K02015	340.73	391.9	2e-117	iron complex transport system permease protein
CP005080.1_1832	K10834	317.57	405.7	1.1e-121	heme transport system ATP-binding protein [EC:7.6.2.5]
CP005080.1_1832	K02013	238.30	309.4	1.7e-92	iron complex transport system ATP-binding protein [EC:7.2.2.-]
CP005080.1_1836	K27545	366.50	445.3	4.8e-134	arginine dihydrolase [EC:3.5.3.27]
CP005080.1_1837	K27906	161.30	195.5	7e-58	ribosome assembly protein METTL17/RSM22, mitochondrial
CP005080.1_1839	K07552	308.70	560.4	1.5e-168	MFS transporter, DHA1 family, multidrug resistance protein
CP005080.1_1840	K00078	265.20	364.5	4.1e-109	dihydrodiol dehydrogenase / D-xylose 1-dehydrogenase (NADP) [EC:1.3.1.20 1.1.1.179]
CP005080.1_1841	K01113	89.83	491.5	1.7e-147	alkaline phosphatase D [EC:3.1.3.1]
CP005080.1_1844	K06998	103.40	200.5	1.7e-59	trans-2,3-dihydro-3-hydroxyanthranilate isomerase [EC:5.3.3.17]
CP005080.1_1854	K09861	102.20	300.2	1e-89	uncharacterized protein
CP005080.1_1855	K01625	193.93	310.7	7.4e-93	2-dehydro-3-deoxyphosphogluconate aldolase / (4S)-4-hydroxy-2-oxoglutarate aldolase [EC:4.1.2.14 4.1.3.42]
CP005080.1_1856	K07090	37.03	88.9	1.4e-25	uncharacterized protein
CP005080.1_1857	K22316	330.73	620.0	7.8e-187	ribonuclease H / adenosylcobalamin/alpha-ribazole phosphatase [EC:3.1.26.4 3.1.3.73]
CP005080.1_1858	K07164	74.07	178.2	7.7e-53	uncharacterized protein
CP005080.1_1860	K25286	277.13	332.8	1.8e-99	iron-siderophore transport system substrate-binding protein
CP005080.1_1871	K02575	149.60	395.1	2.8e-118	MFS transporter, NNP family, nitrate/nitrite transporter
CP005080.1_1873	K24288	87.97	385.5	3.1e-116	MMP 1-O-methyltransferase [EC:2.1.1.365]
CP005080.1_1874	K01448	29.13	135.5	8.1e-40	N-acetylmuramoyl-L-alanine amidase [EC:3.5.1.28]
CP005080.1_1880	K27194	359.80	553.7	5.8e-167	MMP endo-(1,4)-3-O-methyl-alpha-D-mannosidase [EC:3.2.1.221]
CP005080.1_1882	K26815	473.27	766.6	2.5e-231	MMP alpha-(1->4)-mannosyltransferase [EC:2.4.1.393]
CP005080.1_1883	K22107	96.03	345.4	1.4e-103	TetR/AcrR family transcriptional regulator, cholesterol catabolism regulator
CP005080.1_1887	K05337	42.27	75.7	1.4e-21	ferredoxin
CP005080.1_1892	K16785	103.27	182.5	3.9e-54	energy-coupling factor transport system permease protein
CP005080.1_1893	K01552	403.80	657.9	6.8e-198	energy-coupling factor transport system ATP-binding protein [EC:7.-.-.-]
CP005080.1_1894	K16927	47.27	345.9	1e-103	energy-coupling factor transport system substrate-specific component
CP005080.1_1896	K15981	498.67	679.9	3.8e-205	cholest-4-en-3-one 26-monooxygenase [EC:1.14.15.29]
CP005080.1_1902	K19270	224.67	284.5	3.6e-85	mannitol-1-/sugar-/sorbitol-6-phosphatase [EC:3.1.3.22 3.1.3.23 3.1.3.50]
CP005080.1_1903	K02069	66.73	240.2	1.9e-71	UDP-glucose/iron transport system permease protein
CP005080.1_1905	K01113	89.83	128.3	1.5e-37	alkaline phosphatase D [EC:3.1.3.1]
CP005080.1_1906	K15977	60.27	91.3	3.5e-26	putative oxidoreductase
CP005080.1_1908	K03975	59.97	94.0	3.5e-27	membrane-associated protein
CP005080.1_1913	K06204	35.73	88.6	2.8e-25	RNA polymerase-binding transcription factor
CP005080.1_1924	K09799	176.10	572.7	4.3e-172	uncharacterized protein
CP005080.1_1925	K05795	235.30	338.0	2.7e-101	tellurium resistance protein TerD
CP005080.1_1926	K05795	235.30	349.6	8e-105	tellurium resistance protein TerD
CP005080.1_1927	K24120	173.97	271.5	2.7e-81	mycoredoxin-dependent peroxiredoxin [EC:1.11.1.29]
CP005080.1_1929	K00163	290.30	1467.7	0	pyruvate dehydrogenase E1 component [EC:1.2.4.1]
CP005080.1_1931	K08167	383.90	399.0	1.3e-119	MFS transporter, DHA2 family, multidrug resistance protein
CP005080.1_1932	K24699	97.17	98.5	1.5e-28	TetR/AcrR family transcriptional regulator, regulator of mycofactocin system
CP005080.1_1939	K05275	257.70	275.5	3.2e-82	pyridoxine 4-dehydrogenase [EC:1.1.1.65]
CP005080.1_1940	K21745	110.70	126.8	2.2e-37	MerR family transcriptional regulator, aldehyde-responsive regulator
CP005080.1_1945	K06911	36.97	136.2	6.9e-40	quercetin 2,3-dioxygenase [EC:1.13.11.24]
CP005080.1_1947	K00645	247.17	257.1	1.5e-76	[acyl-carrier-protein] S-malonyltransferase [EC:2.3.1.39]
CP005080.1_1948	K00648	229.33	365.6	1.5e-109	3-oxoacyl-[acyl-carrier-protein] synthase III [EC:2.3.1.180]
CP005080.1_1949	K02078	39.80	79.7	1.2e-22	acyl carrier protein
CP005080.1_1950	K09458	480.27	512.9	4.9e-154	3-oxoacyl-[acyl-carrier-protein] synthase II [EC:2.3.1.179]
CP005080.1_1953	K01785	42.83	420.7	3.9e-126	aldose 1-epimerase [EC:5.1.3.3]
CP005080.1_1954	K05349	305.00	734.8	6.8e-221	beta-glucosidase [EC:3.2.1.21]
CP005080.1_1956	K05835	156.00	221.0	4.8e-66	threonine efflux protein
CP005080.1_1957	K06203	50.93	287.5	4.7e-86	CysZ protein
CP005080.1_1958	K04063	57.63	198.6	5.2e-59	lipoyl-dependent peroxiredoxin [EC:1.11.1.28]

CP005080.1_1963	K03569	108.73	421.9	9.2e-127	rod shape-determining protein MreB and related proteins
CP005080.1_1964	K07682	177.80	493.9	4.2e-148	two-component system, NarL family, sensor histidine kinase DevS [EC:2.7.13.3]
CP005080.1_1965	K03719	131.40	148.6	8.2e-44	Lrp/AsnC family transcriptional regulator, leucine-responsive regulatory protein
CP005080.1_1966	K02575	149.60	319.4	2.1e-95	MFS transporter, NNP family, nitrate/nitrite transporter
CP005080.1_1967	K09992	76.67	288.0	4.6e-86	uncharacterized protein
CP005080.1_1967	K15538	133.70	277.9	7e-83	glycoprotein endo-alpha-1,2-mannosidase [EC:3.2.1.130]
CP005080.1_1969	K18661	554.63	718.5	5.1e-216	malonyl-CoA/methylmalonyl-CoA synthetase [EC:6.2.1.76 6.2.1.-]
CP005080.1_1970	K04104	374.50	594.5	1.2e-178	acyl-CoA carboxylase subunit beta [EC:6.4.1.-]
CP005080.1_1971	K22041	192.13	202.5	2.9e-60	TetR/AcrR family transcriptional regulator, copper-responsive repressor
CP005080.1_1971	K16137	104.80	107.4	4.5e-31	TetR/AcrR family transcriptional regulator, transcriptional repressor for nem operon
CP005080.1_1972	K00059	269.80	282.0	3.1e-84	3-oxoacyl-[acyl-carrier protein] reductase [EC:1.1.1.100]
CP005080.1_1973	K08225	199.57	309.0	2e-92	MFS transporter, ENTS family, enterobactin (siderophore) exporter
CP005080.1_1975	K04757	53.50	94.4	3.3e-27	serine/threonine-protein kinase RsbW [EC:2.7.11.1]
CP005080.1_1976	K04749	63.93	90.0	8.4e-26	anti-sigma B factor antagonist
CP005080.1_1982	K18887	708.60	942.9	5.6e-284	ATP-binding cassette, subfamily B, multidrug efflux pump
CP005080.1_1983	K18888	717.83	1018.6	7.4e-307	ATP-binding cassette, subfamily B, multidrug efflux pump
CP005080.1_1983	K06147	612.93	634.9	7.7e-191	ATP-binding cassette, subfamily B, bacterial
CP005080.1_1984	K03086	466.80	490.1	4.4e-147	RNA polymerase primary sigma factor
CP005080.1_1985	K02316	140.00	588.6	7.2e-177	DNA primase [EC:2.7.7.101]
CP005080.1_1988	K08167	383.90	420.1	5.6e-126	MFS transporter, DHA2 family, multidrug resistance protein
CP005080.1_1989	K00529	395.20	546.9	2.3e-164	3-phenylpropionate/trans-cinnamate dioxygenase ferredoxin reductase component [EC:1.18.1.3]
CP005080.1_1990	K01129	92.73	446.4	7.1e-134	dGTPase [EC:3.1.5.1]
CP005080.1_1991	K03795	119.77	169.4	5.7e-50	sirohydrochlorin cobaltochelatase [EC:4.99.1.3]
CP005080.1_1992	K03748	108.27	219.0	3.6e-65	SanA protein
CP005080.1_1993	K00372	1054.60	1115.2	0	assimilatory nitrate reductase catalytic subunit [EC:1.7.99.-]
CP005080.1_1995	K03926	32.57	134.0	3e-39	periplasmic divalent cation tolerance protein
CP005080.1_1996	K19784	108.27	123.7	4.1e-36	chromate reductase, NAD(P)H dehydrogenase (quinone)
CP005080.1_2001	K07090	37.03	102.4	1.2e-29	uncharacterized protein
CP005080.1_2002	K00360	569.70	604.2	1.4e-181	assimilatory nitrate reductase electron transfer subunit [EC:1.7.99.-]
CP005080.1_2003	K00362	1299.73	1545.8	0	nitrite reductase (NADH) large subunit [EC:1.7.1.15]
CP005080.1_2004	K00363	142.30	163.6	2.3e-48	nitrite reductase (NADH) small subunit [EC:1.7.1.15]
CP005080.1_2005	K07093	112.23	251.1	1.1e-74	uncharacterized protein
CP005080.1_2006	K07032	75.30	162.2	5.3e-48	uncharacterized protein
CP005080.1_2008	K01006	344.53	1370.8	0	pyruvate, orthophosphate dikinase [EC:2.7.9.1]
CP005080.1_2010	K02052	502.83	504.0	2.6e-151	putative spermidine/putrescine transport system ATP-binding protein
CP005080.1_2010	K02010	438.13	442.6	6.9e-133	iron(III) transport system ATP-binding protein [EC:7.2.2.7]
CP005080.1_2013	K02055	194.27	268.7	5.4e-80	putative spermidine/putrescine transport system substrate-binding protein
CP005080.1_2014	K03710	178.10	195.8	5.9e-58	GntR family transcriptional regulator
CP005080.1_2015	K05540	281.20	380.1	4.8e-114	tRNA-dihydrouridine synthase B [EC:1.-.-.-]
CP005080.1_2016	K00661	129.97	173.3	2.5e-51	maltose O-acetyltransferase [EC:2.3.1.79]
CP005080.1_2024	K01425	83.30	369.7	1.1e-110	glutaminase [EC:3.5.1.2]
CP005080.1_2026	K03574	70.57	74.2	3.9e-21	8-oxo-dGTP diphosphatase [EC:3.6.1.55]
CP005080.1_2029	K01061	89.07	123.7	4.5e-36	carboxymethylenebutenolidase [EC:3.1.1.45]
CP005080.1_2030	K07727	44.53	116.8	3.6e-34	putative transcriptional regulator
CP005080.1_2036	K27108	396.97	406.5	4.5e-122	epoxide hydrolase A/B [EC:3.3.2.-]
CP005080.1_2052	K20332	413.20	803.2	3.7e-242	toxoflavin biosynthesis protein ToxC
CP005080.1_2056	K01497	257.40	281.2	6e-84	GTP cyclohydrolase II [EC:3.5.4.25]
CP005080.1_2058	K20333	229.57	303.8	7.1e-91	toxoflavin biosynthesis protein ToxD
CP005080.1_2061	K01880	307.53	620.7	2.3e-186	glycyl-tRNA synthetase [EC:6.1.1.14]
CP005080.1_2062	K09815	268.47	357.2	7.5e-107	zinc transport system substrate-binding protein
CP005080.1_2063	K09817	271.90	288.9	2.8e-86	zinc transport system ATP-binding protein [EC:7.2.2.20]
CP005080.1_2064	K09816	273.47	377.8	1.9e-113	zinc transport system permease protein
CP005080.1_2065	K03711	142.10	156.7	3.9e-46	Fur family transcriptional regulator, ferric uptake regulator
CP005080.1_2066	K00806	330.87	392.1	7.1e-118	undecaprenyl diphosphate synthase [EC:2.5.1.31]
CP005080.1_2067	K03584	28.20	249.2	2.7e-74	DNA repair protein RecO (recombination protein O)
CP005080.1_2069	K01649	498.33	702.8	3.6e-211	2-isopropylmalate synthase [EC:2.3.3.13]

CP005080.1_2072	K03595	144.20	441.3	2.2e-132	GTPase
CP005080.1_2077	K03699	305.47	413.5	5.3e-124	magnesium and cobalt exporter, CNNM family
CP005080.1_2078	K07042	60.10	169.8	3.1e-50	probable rRNA maturation factor
CP005080.1_2079	K06217	216.80	582.5	4.9e-175	phosphate starvation-inducible protein PhoH and related proteins
CP005080.1_2082	K21053	376.57	557.9	4.1e-168	adenine deaminase [EC:3.5.4.2]
CP005080.1_2083	K00784	70.63	203.6	1.8e-60	ribonuclease Z [EC:3.1.26.11]
CP005080.1_2084	K02503	85.60	159.4	3.4e-47	histidine triad (HIT) family protein [EC:3.9.1.-]
CP005080.1_2085	K09761	57.73	248.1	4.7e-74	16S rRNA (uracil1498-N3)-methyltransferase [EC:2.1.1.193]
CP005080.1_2086	K00459	256.27	416.3	1.1e-124	nitronate monooxygenase [EC:1.13.12.16]
CP005080.1_2087	K03686	406.37	477.9	2.2e-143	molecular chaperone DnaJ
CP005080.1_2088	K03705	67.57	444.7	1.9e-133	heat-inducible transcriptional repressor
CP005080.1_2092	K02495	261.80	269.5	1.9e-80	oxygen-independent coproporphyrinogen III oxidase [EC:1.3.98.3]
CP005080.1_2093	K07315	145.67	197.3	1.7e-58	phosphoserine phosphatase RsbU/P [EC:3.1.3.3]
CP005080.1_2094	K01897	430.97	511.5	1.4e-153	long-chain acyl-CoA synthetase [EC:6.2.1.3]
CP005080.1_2095	K03596	927.43	1081.9	0	GTP-binding protein LepA
CP005080.1_2096	K02968	24.97	113.4	5.1e-33	small subunit ribosomal protein S20
CP005080.1_2097	K02340	96.93	258.0	7.8e-77	DNA polymerase III subunit delta [EC:2.7.7.7]
CP005080.1_2100	K02238	64.77	541.9	1.3e-162	competence protein ComEC
CP005080.1_2101	K02237	75.73	199.9	3e-59	competence protein ComEA
CP005080.1_2102	K25232	87.57	355.0	2.1e-106	fatty acid kinase fatty acid binding subunit
CP005080.1_2104	K01869	385.87	1133.2	0	leucyl-tRNA synthetase [EC:6.1.1.4]
CP005080.1_2106	K05337	42.27	56.6	1.1e-15	ferredoxin
CP005080.1_2109	K09710	113.50	158.1	1e-46	ribosome-associated protein
CP005080.1_2111	K00969	121.70	318.3	3.3e-95	nicotinate-nucleotide adenylyltransferase [EC:2.7.7.18]
CP005080.1_2117	K00147	552.77	703.0	1.6e-211	glutamate-5-semialdehyde dehydrogenase [EC:1.2.1.41]
CP005080.1_2119	K00931	289.63	536.4	4.5e-161	glutamate 5-kinase [EC:2.7.2.11]
CP005080.1_2120	K09809	276.63	530.2	5.3e-159	CDP-glycerol glycerophosphotransferase [EC:2.7.8.12]
CP005080.1_2121	K22708	268.57	411.5	3.4e-123	poly(ribitol-phosphate) beta-N-acetylglucosaminyltransferase [EC:2.4.1.355]
CP005080.1_2123	K21681	351.83	782.8	3.5e-236	ribitol-5-phosphate 2-dehydrogenase (NADP+) / D-ribitol-5-phosphate cytidylyltransferase [EC:1.1.1.405 2.7.7.40]
CP005080.1_2124	K03979	149.20	494.1	1.6e-148	GTPase [EC:3.6.5.-]
CP005080.1_2125	K02899	36.30	136.2	5.3e-40	large subunit ribosomal protein L27
CP005080.1_2126	K02888	28.73	111.9	1.3e-32	large subunit ribosomal protein L21
CP005080.1_2127	K23356	46.03	52.1	1.9e-14	HTH-type transcriptional regulator, sugar sensing transcriptional regulator
CP005080.1_2131	K21687	174.20	181.3	2e-53	resuscitation-promoting factor RpfA
CP005080.1_2132	K13018	127.73	284.7	3.5e-85	UDP-2-acetamido-3-amino-2,3-dideoxy-glucuronate N-acetyltransferase [EC:2.3.1.201]
CP005080.1_2133	K16568	159.77	171.6	1e-50	exopolysaccharide production protein ExoZ
CP005080.1_2138	K22708	268.57	475.7	1.2e-142	poly(ribitol-phosphate) beta-N-acetylglucosaminyltransferase [EC:2.4.1.355]
CP005080.1_2140	K13010	413.10	442.2	1.2e-132	perosamine synthetase [EC:2.6.1.102]
CP005080.1_2141	K08300	609.20	746.7	1e-224	ribonuclease E [EC:3.1.26.12]
CP005080.1_2146	K05837	385.70	415.0	1.7e-124	rod shape determining protein RodA
CP005080.1_2147	K05515	509.37	646.0	3.9e-194	penicillin-binding protein 2 [EC:3.4.16.4]
CP005080.1_2148	K03571	31.87	52.5	2.5e-14	rod shape-determining protein MreD
CP005080.1_2149	K03570	52.93	251.7	7.3e-75	rod shape-determining protein MreC
CP005080.1_2150	K03569	108.73	574.6	4.3e-173	rod shape-determining protein MreB and related proteins
CP005080.1_2151	K00940	149.93	194.2	1.4e-57	nucleoside-diphosphate kinase [EC:2.7.4.6]
CP005080.1_2153	K11754	379.57	557.1	3.8e-167	dihydrofolate synthase / folylpolyglutamate synthase [EC:6.3.2.12 6.3.2.17]
CP005080.1_2154	K01873	582.43	1233.8	0	valyl-tRNA synthetase [EC:6.1.1.9]
CP005080.1_2156	K03544	145.17	647.7	7.4e-195	ATP-dependent Clp protease ATP-binding subunit ClpX
CP005080.1_2157	K01358	76.73	300.6	5.5e-90	ATP-dependent Clp protease, protease subunit [EC:3.4.21.92]
CP005080.1_2158	K01358	76.73	288.4	2.7e-86	ATP-dependent Clp protease, protease subunit [EC:3.4.21.92]
CP005080.1_2159	K03545	69.90	495.3	1.1e-148	trigger factor
CP005080.1_2163	K26732	261.93	468.9	4.9e-141	K+:H+ antiporter subunit KhtU
CP005080.1_2166	K05522	197.43	324.5	3e-97	endonuclease VIII [EC:3.2.2.- 4.2.99.18]
CP005080.1_2167	K01808	185.87	211.1	1.1e-62	ribose 5-phosphate isomerase B [EC:5.3.1.6]
CP005080.1_2168	K11735	615.90	739.9	4.8e-223	GABA permease
CP005080.1_2169	K03523	54.23	190.0	1.9e-56	biotin transport system substrate-specific component

CP005080.1_2170	K16237	628.43	719.7	4.2e-217	aromatic amino acid permease
CP005080.1_2170	K11735	615.90	636.5	1.1e-191	GABA permease
CP005080.1_2173	K01126	152.53	214.5	7.3e-64	glycerophosphoryl diester phosphodiesterase [EC:3.1.4.46]
CP005080.1_2174	K04047	77.30	219.5	2.9e-65	starvation-inducible DNA-binding protein
CP005080.1_2175	K00615	214.20	546.4	5e-164	transketolase [EC:2.2.1.1]
CP005080.1_2177	K04564	41.50	264.5	3.7e-79	superoxide dismutase, Fe-Mn family [EC:1.15.1.1]
CP005080.1_2180	K18234	238.93	259.7	1.3e-77	virginiamycin A acetyltransferase [EC:2.3.1.-]
CP005080.1_2182	K01634	365.70	558.2	9e-168	sphinganine-1-phosphate aldolase [EC:4.1.2.27]
CP005080.1_2183	K03292	344.60	355.3	1.9e-106	glycoside/pentoside/hexuronide:cation symporter, GPH family
CP005080.1_2186	K01256	435.67	926.3	1.5e-278	aminopeptidase N [EC:3.4.11.2]
CP005080.1_2188	K01280	273.03	324.8	4.4e-97	tripeptidyl-peptidase II [EC:3.4.14.10]
CP005080.1_2189	K00133	214.73	469.5	6e-141	aspartate-semialdehyde dehydrogenase [EC:1.2.1.11]
CP005080.1_2190	K07171	19.10	107.6	3.9e-31	mRNA interferase MazF [EC:3.1.-.-]
CP005080.1_2191	K26989	67.07	115.9	2.6e-34	antitoxin MazE9
CP005080.1_2192	K01256	435.67	935.7	2.1e-281	aminopeptidase N [EC:3.4.11.2]
CP005080.1_2194	K01477	148.47	489.8	4.8e-147	allantoicase [EC:3.5.3.4]
CP005080.1_2197	K00705	118.23	842.1	1.8e-253	4-alpha-glucanotransferase [EC:2.4.1.25]
CP005080.1_2201	K22044	182.20	331.4	4e-99	moderate conductance mechanosensitive channel
CP005080.1_2203	K02027	193.17	229.8	3.1e-68	multiple sugar transport system substrate-binding protein
CP005080.1_2204	K02025	276.90	285.7	1.8e-85	multiple sugar transport system permease protein
CP005080.1_2205	K02026	280.30	326.6	7.6e-98	multiple sugar transport system permease protein
CP005080.1_2206	K01222	394.63	592.6	2.1e-178	6-phospho-beta-glucosidase [EC:3.2.1.86]
CP005080.1_2209	K02030	76.77	134.2	2.6e-39	polar amino acid transport system substrate-binding protein
CP005080.1_2211	K14949	217.50	1154.8	0	serine/threonine-protein kinase PknG [EC:2.7.11.1]
CP005080.1_2212	K20074	131.13	176.2	5.9e-52	PPM family protein phosphatase [EC:3.1.3.16]
CP005080.1_2215	K00573	87.43	180.3	2.2e-53	protein-L-isoaspartate(D-aspartate) O-methyltransferase [EC:2.1.1.77]
CP005080.1_2216	K06886	39.40	151.4	1.6e-44	hemoglobin
CP005080.1_2218	K07107	72.43	150.9	2.8e-44	acyl-CoA thioester hydrolase [EC:3.1.2.-]
CP005080.1_2219	K06020	679.63	933.5	1.5e-281	energy-dependent translational throttle protein EttA
CP005080.1_2221	K03111	28.83	81.7	3.1e-23	single-strand DNA-binding protein
CP005080.1_2226	K01990	262.37	328.1	3.7e-98	ABC-2 type transport system ATP-binding protein
CP005080.1_2231	K07114	102.73	113.6	4e-33	Ca-activated chloride channel homolog
CP005080.1_2233	K01990	262.37	338.7	2.2e-101	ABC-2 type transport system ATP-binding protein
CP005080.1_2236	K03574	70.57	72.3	1.5e-20	8-oxo-dGTP diphosphatase [EC:3.6.1.55]
CP005080.1_2241	K00140	503.87	873.1	8.8e-263	malonate-semialdehyde dehydrogenase (acetylating) / methylmalonate-semialdehyde dehydrogenase [EC:1.2.1.18 1.2.1.27]
CP005080.1_2242	K03336	427.33	1001.6	5.5e-302	3D-(3,5/4)-trihydroxycyclohexane-1,2-dione acylhydrolase (decyclizing) [EC:3.7.1.22]
CP005080.1_2243	K03337	142.03	481.0	6.6e-145	5-deoxy-glucuronate isomerase [EC:5.3.1.30]
CP005080.1_2245	K03338	276.17	590.2	8.2e-178	5-dehydro-2-deoxygluconokinase [EC:2.7.1.92]
CP005080.1_2246	K03335	136.63	346.4	8.2e-104	inosose dehydratase [EC:4.2.1.44]
CP005080.1_2249	K16868	93.13	98.1	2.2e-28	tellurite methyltransferase [EC:2.1.1.265]
CP005080.1_2250	K06994	260.33	683.9	1.1e-205	putative drug exporter of the RND superfamily
CP005080.1_2253	K17686	799.73	1029.0	7.3e-310	P-type Cu+ transporter [EC:7.2.2.8]
CP005080.1_2254	K01647	417.47	473.7	3e-142	citrate synthase [EC:2.3.3.1]
CP005080.1_2256	K03581	242.43	500.0	4.6e-150	exodeoxyribonuclease V alpha subunit [EC:3.1.11.5]
CP005080.1_2275	K02529	268.37	334.1	6.6e-100	LacI family transcriptional regulator, galactose operon repressor
CP005080.1_2276	K27862	199.47	1101.4	0	class II lanthipeptide synthase [EC:3.13.2.4]
CP005080.1_2279	K22894	131.70	296.3	1.7e-88	SARP family transcriptional regulator, regulator of embCAB operon
CP005080.1_2280	K24914	586.77	958.1	2e-288	class III lanthionine synthetase [EC:3.13.2.4]
CP005080.1_2293	K00232	166.57	267.3	1.2e-79	acyl-CoA oxidase [EC:1.3.3.6]
CP005080.1_2294	K00648	229.33	335.4	2.2e-100	3-oxoacyl-[acyl-carrier-protein] synthase III [EC:2.3.1.180]
CP005080.1_2296	K00648	229.33	384.0	3.8e-115	3-oxoacyl-[acyl-carrier-protein] synthase III [EC:2.3.1.180]
CP005080.1_2297	K02078	39.80	55.9	2.1e-15	acyl carrier protein
CP005080.1_2299	K01207	251.63	574.8	9.8e-173	beta-N-acetylhexosaminidase [EC:3.2.1.52]
CP005080.1_2302	K06147	612.93	705.9	2.7e-212	ATP-binding cassette, subfamily B, bacterial
CP005080.1_2305	K01652	517.70	535.0	1.1e-160	acetolactate synthase I/II/III large subunit [EC:2.2.1.6]
CP005080.1_2306	K25365	384.40	458.1	1.9e-137	guanidinobutyrase / D-arginase [EC:3.5.3.7 3.5.3.10]

CP005080.1_2306	K01480	221.23	342.5	1.5e-102	agmatinase [EC:3.5.3.11]
CP005080.1_2307	K03307	271.33	378.7	2.2e-113	solute:Na+ symporter, SSS family
CP005080.1_2308	K09684	188.00	218.3	9.5e-65	PucR family transcriptional regulator, purine catabolism regulatory protein
CP005080.1_2323	K10805	357.83	454.5	1.7e-136	acyl-CoA thioesterase II [EC:3.1.2.-]
CP005080.1_2329	K19267	112.87	138.3	1.8e-40	NAD(P)H dehydrogenase (quinone) [EC:1.6.5.2]
CP005080.1_2330	K07148	83.90	325.0	2.2e-97	uncharacterized protein
CP005080.1_2333	K01969	776.53	1055.8	7.3e-318	3-methylcrotonyl-CoA carboxylase beta subunit [EC:6.4.1.4]
CP005080.1_2334	K01968	923.57	987.0	3.8e-297	3-methylcrotonyl-CoA carboxylase alpha subunit [EC:6.4.1.4]
CP005080.1_2335	K01640	135.00	477.4	2e-143	hydroxymethylglutaryl-CoA lyase [EC:4.1.3.4]
CP005080.1_2336	K11410	517.13	694.1	1.9e-209	short-chain 2-methylacyl-CoA dehydrogenase [EC:1.3.8.5]
CP005080.1_2336	K00249	382.07	390.2	7.2e-117	acyl-CoA dehydrogenase [EC:1.3.8.7]
CP005080.1_2338	K22390	122.97	126.5	4.9e-37	acid phosphatase type 7
CP005080.1_2343	K13745	417.70	678.7	4.5e-204	L-2,4-diaminobutyrate decarboxylase [EC:4.1.1.86]
CP005080.1_2344	K27501	461.57	645.3	1.9e-194	putrescine N-hydroxylase [EC:1.14.13.252]
CP005080.1_2347	K12373	123.03	446.9	4.2e-134	hexosaminidase [EC:3.2.1.52]
CP005080.1_2350	K00820	183.57	796.0	1.6e-239	glutamine---fructose-6-phosphate transaminase (isomerizing) [EC:2.6.1.16]
CP005080.1_2352	K13633	271.97	458.2	8.4e-138	AraC family transcriptional regulator, transcriptional activator FtrA
CP005080.1_2353	K13288	60.80	280.3	6.7e-84	oligoribonuclease [EC:3.1.-.-]
CP005080.1_2356	K02529	268.37	352.4	1.9e-105	LacI family transcriptional regulator, galactose operon repressor
CP005080.1_2357	K05350	554.93	661.9	6.2e-199	beta-glucosidase [EC:3.2.1.21]
CP005080.1_2358	K10242	321.97	381.6	6.8e-115	cellobiose transport system permease protein
CP005080.1_2358	K02026	280.30	280.4	7.9e-84	multiple sugar transport system permease protein
CP005080.1_2359	K10241	361.87	481.3	7.9e-145	cellobiose transport system permease protein
CP005080.1_2359	K02025	276.90	320.1	6.4e-96	multiple sugar transport system permease protein
CP005080.1_2360	K10240	310.77	494.8	9.2e-149	cellobiose transport system substrate-binding protein
CP005080.1_2361	K07085	209.40	555.5	4.6e-167	putative transport protein
CP005080.1_2362	K09758	465.47	860.9	1.4e-259	aspartate 4-decarboxylase [EC:4.1.1.12]
CP005080.1_2380	K01799	79.27	126.5	6.2e-37	maleate isomerase [EC:5.2.1.1]
CP005080.1_2382	K02030	76.77	122.2	1.2e-35	polar amino acid transport system substrate-binding protein
CP005080.1_2383	K02029	214.53	236.3	2.4e-70	polar amino acid transport system permease protein
CP005080.1_2384	K02029	214.53	225.0	6.4e-67	polar amino acid transport system permease protein
CP005080.1_2385	K02028	386.70	406.1	8.8e-122	polar amino acid transport system ATP-binding protein [EC:7.4.2.1]
CP005080.1_2388	K25776	749.23	910.8	1e-274	steroid-22-oyl-CoA synthetase [EC:6.2.1.-]
CP005080.1_2397	K02566	258.43	507.2	2.1e-152	5'-nucleotidase [EC:3.1.3.5]
CP005080.1_2399	K11081	423.60	560.0	5.5e-169	2-aminoethylphosphonate transport system substrate-binding protein
CP005080.1_2400	K11082	269.50	400.7	4.4e-120	2-aminoethylphosphonate transport system permease protein
CP005080.1_2401	K11083	301.53	475.1	2.4e-143	2-aminoethylphosphonate transport system permease protein
CP005080.1_2402	K11084	436.93	583.7	4.9e-176	2-aminoethylphosphonate transport system ATP-binding protein
CP005080.1_2404	K27877	276.37	392.7	5.3e-118	2-(methylaminoethyl)phosphonate oxidase [EC:1.5.3.27]
CP005080.1_2405	K03710	178.10	215.7	5e-64	GntR family transcriptional regulator
CP005080.1_2411	K18336	331.50	397.5	2.5e-119	2,4-didehydro-3-deoxy-L-rhamnonate hydrolase [EC:3.7.1.26]
CP005080.1_2412	K03217	121.07	161.5	1e-47	YidC/Oxa1 family membrane protein insertase
CP005080.1_2414	K07284	60.53	212.9	2.6e-63	sortase A [EC:3.4.22.70]
CP005080.1_2421	K01295	220.93	452.3	9.7e-136	glutamate carboxypeptidase [EC:3.4.17.11]
CP005080.1_2424	K18353	128.17	394.2	3.3e-118	vancomycin resistance protein VanJ
CP005080.1_2427	K21744	131.27	201.3	1.1e-59	MerR family transcriptional regulator, thiopeptide resistance regulator
CP005080.1_2434	K01872	199.53	355.0	4.1e-106	alanyl-tRNA synthetase [EC:6.1.1.7]
CP005080.1_2438	K00761	217.77	220.7	1.1e-65	uracil phosphoribosyltransferase [EC:2.4.2.9]
CP005080.1_2439	K15269	141.60	429.9	2.8e-129	probable blue pigment (indigoidine) exporter
CP005080.1_2440	K01091	112.83	190.7	1.9e-56	phosphoglycolate phosphatase [EC:3.1.3.18]
CP005080.1_2441	K27871	1128.67	1842.0	0	indigoidine synthase [EC:4.3.3.9]
CP005080.1_2442	K01821	25.90	45.6	2.9e-12	4-oxalocrotonate tautomerase [EC:5.3.2.6]
CP005080.1_2443	K16329	279.93	386.5	4.6e-116	pseudouridylate synthase [EC:4.2.1.70]
CP005080.1_2447	K25156	291.53	534.9	1.1e-160	viologen exporter family transport system ATP-binding protein
CP005080.1_2448	K25154	114.47	335.2	1.7e-100	viologen exporter family transport system permease protein
CP005080.1_2449	K25155	105.97	324.6	5.1e-97	viologen exporter family transport system permease protein

CP005080.1_2451	K11741	110.40	141.8	7e-42	quaternary ammonium compound-resistance protein SugE
CP005080.1_2452	K04078	31.27	69.5	1.1e-19	chaperonin GroES
CP005080.1_2454	K03564	157.77	221.2	8.2e-66	thioredoxin-dependent peroxiredoxin [EC:1.11.1.24]
CP005080.1_2455	K03762	459.97	691.2	5.5e-208	MFS transporter, MHS family, proline/betaine transporter
CP005080.1_2457	K01519	53.90	265.1	4.8e-79	XTP/dITP diphosphohydrolase [EC:3.6.1.66]
CP005080.1_2459	K00989	189.03	445.7	6.3e-134	ribonuclease PH [EC:2.7.7.56]
CP005080.1_2460	K02803	96.17	130.1	7.1e-39	N-acetylglucosamine PTS system EIIB component [EC:2.7.1.193]
CP005080.1_2463	K00784	70.63	112.2	9.5e-33	ribonuclease Z [EC:3.1.26.11]
CP005080.1_2464	K07171	19.10	36.9	1.5e-09	mRNA interferase MazF [EC:3.1.-.-]
CP005080.1_2465	K21148	399.80	558.0	2.2e-168	[CysO sulfur-carrier protein]-thiocarboxylate-dependent cysteine synthase [EC:2.5.1.113]
CP005080.1_2466	K03636	36.60	83.5	7.2e-24	sulfur-carrier protein
CP005080.1_2467	K02013	238.30	368.3	2.2e-110	iron complex transport system ATP-binding protein [EC:7.2.2.-]
CP005080.1_2468	K02015	340.73	380.9	4.3e-114	iron complex transport system permease protein
CP005080.1_2469	K02016	154.03	208.7	6.3e-62	iron complex transport system substrate-binding protein
CP005080.1_2470	K21140	125.90	208.4	2e-62	[CysO sulfur-carrier protein]-S-L-cysteine hydrolase [EC:3.13.1.6]
CP005080.1_2471	K03293	609.50	625.8	2.9e-188	amino acid transporter, AAT family
CP005080.1_2473	K06891	42.13	141.0	1.5e-41	ATP-dependent Clp protease adaptor protein ClpS
CP005080.1_2474	K00763	105.10	453.9	4.8e-136	nicotinate phosphoribosyltransferase [EC:6.3.4.21]
CP005080.1_2475	K08281	104.80	294.7	4.2e-88	nicotinamidase/pyrazinamidase [EC:3.5.1.19 3.5.1.-]
CP005080.1_2477	K09607	125.47	817.5	6.8e-246	immune inhibitor A [EC:3.4.24.-]
CP005080.1_2482	K00104	535.33	654.8	5.3e-197	glycolate dehydrogenase FAD-linked subunit [EC:1.1.99.14]
CP005080.1_2484	K00457	312.93	547.8	1.4e-164	4-hydroxyphenylpyruvate dioxygenase [EC:1.13.11.27]
CP005080.1_2486	K05846	171.73	237.7	1.1e-70	osmoprotectant transport system permease protein
CP005080.1_2487	K05847	352.27	507.9	9.3e-153	osmoprotectant transport system ATP-binding protein [EC:7.6.2.9]
CP005080.1_2488	K05846	171.73	295.0	4.9e-88	osmoprotectant transport system permease protein
CP005080.1_2489	K05845	152.73	342.7	1.2e-102	osmoprotectant transport system substrate-binding protein
CP005080.1_2493	K07177	99.10	170.3	2.4e-50	Lon-like protease
CP005080.1_2504	K12373	123.03	495.4	9e-149	hexosaminidase [EC:3.2.1.52]
CP005080.1_2505	K17331	321.87	411.6	7.1e-124	N,N'-diacetylchitobiose transport system permease protein
CP005080.1_2505	K02026	280.30	284.7	4.1e-85	multiple sugar transport system permease protein
CP005080.1_2506	K17330	299.37	395.3	6.7e-119	N,N'-diacetylchitobiose transport system permease protein
CP005080.1_2507	K17329	313.30	485.6	6.1e-146	N,N'-diacetylchitobiose transport system substrate-binding protein
CP005080.1_2507	K02027	193.17	235.2	7.5e-70	multiple sugar transport system substrate-binding protein
CP005080.1_2509	K07735	37.57	182.1	4.2e-54	putative transcriptional regulator
CP005080.1_2510	K00790	172.73	579.1	2.7e-174	UDP-N-acetylglucosamine 1-carboxyvinyltransferase [EC:2.5.1.7]
CP005080.1_2514	K00027	507.30	575.0	1.1e-172	malate dehydrogenase (oxaloacetate-decarboxylating) [EC:1.1.1.38]
CP005080.1_2517	K03088	96.50	144.2	2.1e-42	RNA polymerase sigma-70 factor, ECF subfamily
CP005080.1_2520	K01719	43.23	108.0	2.4e-31	uroporphyrinogen-III synthase [EC:4.2.1.75]
CP005080.1_2521	K02575	149.60	411.3	3.5e-123	MFS transporter, NNP family, nitrate/nitrite transporter
CP005080.1_2523	K11936	261.37	389.4	8.7e-117	poly-beta-1,6-N-acetyl-D-glucosamine synthase [EC:2.4.1.-]
CP005080.1_2523	K22278	73.27	241.4	7.7e-72	peptidoglycan-N-acetylglucosamine deacetylase [EC:3.5.1.104]
CP005080.1_2528	K03664	42.43	257.1	4.6e-77	SsrA-binding protein
CP005080.1_2529	K03797	127.93	285.8	3.4e-85	carboxyl-terminal processing protease [EC:3.4.21.102]
CP005080.1_2530	K09811	79.70	253.6	8.9e-76	cell division transport system permease protein
CP005080.1_2531	K09812	287.03	422.3	4.9e-127	cell division transport system ATP-binding protein
CP005080.1_2534	K02836	378.07	561.3	1e-168	peptide chain release factor 2
CP005080.1_2535	K12132	224.03	245.1	3.7e-73	eukaryotic-like serine/threonine-protein kinase [EC:2.7.11.1]
CP005080.1_2536	K12132	224.03	292.7	1.3e-87	eukaryotic-like serine/threonine-protein kinase [EC:2.7.11.1]
CP005080.1_2538	K10232	198.57	524.5	2.1e-157	alpha-glucoside transport system substrate-binding protein
CP005080.1_2539	K10233	301.17	429.4	6.3e-129	alpha-glucoside transport system permease protein
CP005080.1_2539	K02025	276.90	296.5	9.8e-89	multiple sugar transport system permease protein
CP005080.1_2540	K10234	291.17	418.4	5.3e-126	alpha-glucoside transport system permease protein
CP005080.1_2542	K09809	276.63	340.5	1.1e-101	CDP-glycerol glycerophosphotransferase [EC:2.7.8.12]
CP005080.1_2543	K00712	180.83	193.8	2.1e-57	poly(glycerol-phosphate) alpha-glucosyltransferase [EC:2.4.1.52]
CP005080.1_2544	K09809	276.63	497.1	5.3e-149	CDP-glycerol glycerophosphotransferase [EC:2.7.8.12]
CP005080.1_2545	K09809	276.63	763.3	2.3e-229	CDP-glycerol glycerophosphotransferase [EC:2.7.8.12]

CP005080.1_2546	K05303	104.90	260.4	1.4e-77	O-methyltransferase [EC:2.1.1.-]
CP005080.1_2548	K09809	276.63	447.2	6.4e-134	CDP-glycerol glycerophosphotransferase [EC:2.7.8.12]
CP005080.1_2549	K09809	276.63	685.3	8e-206	CDP-glycerol glycerophosphotransferase [EC:2.7.8.12]
CP005080.1_2552	K09692	218.47	375.1	1.2e-112	teichoic acid transport system permease protein
CP005080.1_2553	K09693	367.70	419.3	5.8e-126	teichoic acid transport system ATP-binding protein [EC:7.5.2.4]
CP005080.1_2554	K15371	275.47	2229.3	0	glutamate dehydrogenase [EC:1.4.1.2]
CP005080.1_2555	K05520	175.93	214.3	9.6e-64	deglycase [EC:3.5.1.124]
CP005080.1_2559	K03070	231.23	1297.3	0	preprotein translocase subunit SecA [EC:7.4.2.8]
CP005080.1_2561	K09927	56.00	448.5	1.4e-134	uncharacterized protein
CP005080.1_2562	K07684	245.47	253.3	1.2e-75	two-component system, NarL family, nitrate/nitrite response regulator NarL
CP005080.1_2563	K05808	97.77	130.0	3.2e-38	ribosome hibernation promoting factor
CP005080.1_2564	K02242	65.47	74.9	3.2e-21	competence protein ComFC
CP005080.1_2566	K07654	329.50	816.9	5.4e-246	two-component system, OmpR family, sensor histidine kinase MtrB [EC:2.7.13.3]
CP005080.1_2567	K07670	316.43	427.1	2.2e-128	two-component system, OmpR family, response regulator MtrA
CP005080.1_2567	K02483	242.00	263.3	1.9e-78	two-component system, OmpR family, response regulator
CP005080.1_2568	K08963	276.73	427.4	3.5e-128	methylthioribose-1-phosphate isomerase [EC:5.3.1.23]
CP005080.1_2572	K03924	113.70	473.1	4.7e-142	MoxR-like ATPase [EC:3.6.3.-]
CP005080.1_2574	K06384	34.00	81.3	3.3e-23	stage II sporulation protein M
CP005080.1_2577	K01251	60.67	783.4	4.4e-236	adenosylhomocysteinase [EC:3.13.2.1]
CP005080.1_2579	K01809	33.17	429.0	1.5e-128	mannose-6-phosphate isomerase [EC:5.3.1.8]
CP005080.1_2581	K09791	36.30	84.9	1.9e-24	uncharacterized protein
CP005080.1_2582	K01840	449.03	484.9	1.9e-145	phosphomannomutase [EC:5.4.2.8]
CP005080.1_2587	K18955	70.43	143.0	3.6e-42	WhiB family transcriptional regulator, redox-sensing transcriptional regulator
CP005080.1_2589	K11212	149.23	538.1	3.7e-162	LPPG:FO 2-phospho-L-lactate transferase [EC:2.7.8.28]
CP005080.1_2590	K24998	440.20	735.6	1.8e-221	dehydro coenzyme F420 reductase / coenzyme F420-0:L-glutamate ligase / coenzyme F420-1:gamma-L-glutamate ligase [EC:1.3.8.17 6.3.2.31 6.3.2.34]
CP005080.1_2590	K12234	165.70	370.8	5.7e-111	coenzyme F420-0:L-glutamate ligase / coenzyme F420-1:gamma-L-glutamate ligase [EC:6.3.2.31 6.3.2.34]
CP005080.1_2592	K00966	307.27	325.5	1.7e-97	mannose-1-phosphate guanylyltransferase [EC:2.7.7.13]
CP005080.1_2593	K01446	92.93	121.6	1.5e-35	peptidoglycan recognition protein
CP005080.1_2595	K01005	193.97	231.7	8e-69	polyisoprenyl-teichoic acid--peptidoglycan teichoic acid transferase [EC:2.7.8.-]
CP005080.1_2598	K16557	245.83	422.3	5.2e-127	succinoglycan biosynthesis protein ExoA [EC:2.4.-.-]
CP005080.1_2599	K01005	193.97	222.1	6.7e-66	polyisoprenyl-teichoic acid--peptidoglycan teichoic acid transferase [EC:2.7.8.-]
CP005080.1_2600	K10806	103.53	135.5	7.1e-40	acyl-CoA thioesterase YciA [EC:3.1.2.-]
CP005080.1_2601	K01005	193.97	220.8	1.6e-65	polyisoprenyl-teichoic acid--peptidoglycan teichoic acid transferase [EC:2.7.8.-]
CP005080.1_2610	K00249	382.07	428.5	1.8e-128	acyl-CoA dehydrogenase [EC:1.3.8.7]
CP005080.1_2611	K00012	358.77	582.9	2.5e-175	UDPglucose 6-dehydrogenase [EC:1.1.1.22]
CP005080.1_2614	K01273	59.53	505.0	7.4e-152	membrane dipeptidase [EC:3.4.13.19]
CP005080.1_2615	K01588	108.13	280.9	3.1e-84	5-(carboxyamino)imidazole ribonucleotide mutase [EC:5.4.99.18]
CP005080.1_2616	K01589	271.73	577.4	1.1e-173	5-(carboxyamino)imidazole ribonucleotide synthase [EC:6.3.4.18]
CP005080.1_2619	K02483	242.00	265.0	5.7e-79	two-component system, OmpR family, response regulator
CP005080.1_2620	K03305	359.10	500.0	2.9e-150	proton-dependent oligopeptide transporter, POT family
CP005080.1_2625	K04757	53.50	58.6	2.7e-16	serine/threonine-protein kinase RsbW [EC:2.7.11.1]
CP005080.1_2626	K06378	115.47	118.3	1.2e-34	stage II sporulation protein AA (anti-sigma F factor antagonist)
CP005080.1_2626	K04749	63.93	109.7	7.9e-32	anti-sigma B factor antagonist
CP005080.1_2627	K03090	268.20	462.1	8.4e-139	RNA polymerase sigma-B factor
CP005080.1_2628	K01468	322.67	595.5	7.8e-179	imidazolonepropionase [EC:3.5.2.7]
CP005080.1_2629	K05603	334.33	410.0	7.7e-123	formimidoylglutamate deiminase [EC:3.5.3.13]
CP005080.1_2630	K06016	482.23	548.4	6.8e-165	beta-ureidopropionase / N-carbamoyl-L-amino-acid hydrolase [EC:3.5.1.6 3.5.1.87]
CP005080.1_2631	K01712	290.83	811.0	4.2e-244	urocanate hydratase [EC:4.2.1.49]
CP005080.1_2634	K07131	45.27	184.2	9.4e-55	uncharacterized protein
CP005080.1_2640	K01697	450.53	657.7	1.2e-197	cystathionine beta-synthase [EC:4.2.1.22]
CP005080.1_2647	K02003	292.97	362.9	8.6e-109	putative ABC transport system ATP-binding protein
CP005080.1_2648	K02004	52.33	189.0	4.4e-56	putative ABC transport system permease protein
CP005080.1_2649	K00574	329.57	453.0	4.3e-136	cyclopropane-fatty-acyl-phospholipid synthase [EC:2.1.1.79]
CP005080.1_2650	K03885	203.30	516.7	4.8e-155	NADH:quinone reductase (non-electrogenic) [EC:1.6.5.9]
CP005080.1_2651	K01524	159.03	434.7	2e-130	exopolyphosphatase / guanosine-5'-triphosphate,3'-diphosphate pyrophosphatase [EC:3.6.1.11 3.6.1.40]
CP005080.1_2652	K09009	43.83	283.2	1.2e-84	uncharacterized protein

CP005080.1_2654	K01689	269.20	769.2	2.2e-231	enolase 1/2/3 [EC:4.2.1.11]
CP005080.1_2655	K21687	174.20	247.1	1.9e-73	resuscitation-promoting factor RpfA
CP005080.1_2656	K21687	174.20	246.7	2.5e-73	resuscitation-promoting factor RpfA
CP005080.1_2660	K12132	224.03	326.5	6.9e-98	eukaryotic-like serine/threonine-protein kinase [EC:2.7.11.1]
CP005080.1_2660	K11912	105.07	136.5	4.3e-40	serine/threonine-protein kinase PpkA [EC:2.7.11.1]
CP005080.1_2669	K03723	555.70	1446.2	0	transcription-repair coupling factor (superfamily II helicase) [EC:5.6.2.4]
CP005080.1_2670	K23356	46.03	50.5	6.1e-14	HTH-type transcriptional regulator, sugar sensing transcriptional regulator
CP005080.1_2674	K09136	53.07	409.3	9.7e-123	ribosomal protein S12 methylthiotransferase accessory factor
CP005080.1_2676	K01990	262.37	311.7	3.5e-93	ABC-2 type transport system ATP-binding protein
CP005080.1_2677	K16922	76.20	158.9	7.2e-47	putative peptide zinc metalloprotease protein
CP005080.1_2679	K20483	108.60	214.5	1e-63	class I lanthipeptide synthase [EC:3.13.2.4]
CP005080.1_2681	K09136	53.07	440.2	4.1e-132	ribosomal protein S12 methylthiotransferase accessory factor
CP005080.1_2683	K22894	131.70	247.6	1e-73	SARP family transcriptional regulator, regulator of embCAB operon
CP005080.1_2685	K02004	52.33	201.2	9e-60	putative ABC transport system permease protein
CP005080.1_2686	K02003	292.97	364.7	2.4e-109	putative ABC transport system ATP-binding protein
CP005080.1_2690	K10947	65.70	136.7	4.8e-40	PadR family transcriptional regulator
CP005080.1_2691	K04757	53.50	68.1	3.4e-19	serine/threonine-protein kinase RsbW [EC:2.7.11.1]
CP005080.1_2702	K04042	321.87	653.3	2e-196	bifunctional UDP-N-acetylglucosamine pyrophosphorylase / glucosamine-1-phosphate N-acetyltransferase [EC:2.7.7.23 2.3.1.157]
CP005080.1_2703	K00948	62.27	421.5	1.7e-126	ribose-phosphate pyrophosphokinase [EC:2.7.6.1]
CP005080.1_2704	K02897	35.70	211.2	7.5e-63	large subunit ribosomal protein L25
CP005080.1_2705	K01056	49.10	264.0	7.8e-79	peptidyl-tRNA hydrolase, PTH1 family [EC:3.1.1.29]
CP005080.1_2707	K01595	49.70	1049.3	6.7e-316	phosphoenolpyruvate carboxylase [EC:4.1.1.31]
CP005080.1_2708	K00507	82.53	298.2	5.3e-89	stearoyl-CoA desaturase (Delta-9 desaturase) [EC:1.14.19.1]
CP005080.1_2712	K00598	120.07	387.9	1.8e-116	trans-aconitate 2-methyltransferase [EC:2.1.1.144]
CP005080.1_2716	K00849	368.57	510.5	3.5e-153	galactokinase [EC:2.7.1.6]
CP005080.1_2717	K01784	253.97	391.7	2.4e-117	UDP-glucose 4-epimerase [EC:5.1.3.2]
CP005080.1_2718	K00965	84.47	408.0	3.1e-122	UDPglucose--hexose-1-phosphate uridylyltransferase [EC:2.7.7.12]
CP005080.1_2719	K03307	271.33	407.2	5.1e-122	solute:Na+ symporter, SSS family
CP005080.1_2725	K15738	622.23	756.2	1.2e-227	ABC transport system ATP-binding/permease protein
CP005080.1_2726	K19159	55.30	93.1	7.4e-27	antitoxin YefM
CP005080.1_2727	K19158	43.63	143.1	2.8e-42	toxin YoeB [EC:3.1.-.-]
CP005080.1_2732	K00919	102.90	296.4	1.2e-88	4-diphosphocytidyl-2-C-methyl-D-erythritol kinase [EC:2.7.1.148]
CP005080.1_2733	K02528	256.10	401.4	1.7e-120	16S rRNA (adenine1518-N6/adenine1519-N6)-dimethyltransferase [EC:2.1.1.182]
CP005080.1_2734	K21688	167.47	568.8	4e-171	resuscitation-promoting factor RpfB
CP005080.1_2735	K03424	86.70	254.2	9.9e-76	TatD DNase family protein [EC:3.1.21.-]
CP005080.1_2742	K07056	92.80	381.7	1.4e-114	16S rRNA (cytidine1402-2'-O)-methyltransferase [EC:2.1.1.198]
CP005080.1_2743	K00728	246.07	428.4	2.6e-128	dolichyl-phosphate-mannose-protein mannosyltransferase [EC:2.4.1.109]
CP005080.1_2756	K04757	53.50	55.8	2e-15	serine/threonine-protein kinase RsbW [EC:2.7.11.1]
CP005080.1_2761	K07052	31.30	54.2	4.3e-15	CAAX protease family protein
CP005080.1_2763	K00059	269.80	279.2	2.2e-83	3-oxoacyl-[acyl-carrier protein] reductase [EC:1.1.1.100]
CP005080.1_2765	K02006	337.97	397.6	3.3e-119	cobalt/nickel transport system ATP-binding protein
CP005080.1_2766	K02008	152.13	256.7	1.2e-76	cobalt/nickel transport system permease protein
CP005080.1_2767	K02007	104.90	372.5	1.7e-111	cobalt/nickel transport system permease protein
CP005080.1_2769	K06994	260.33	877.0	4.7e-264	putative drug exporter of the RND superfamily
CP005080.1_2771	K08676	518.47	1579.2	0	tricorn protease [EC:3.4.21.-]
CP005080.1_2779	K01142	228.27	343.3	1e-102	exodeoxyribonuclease III [EC:3.1.11.2]
CP005080.1_2780	K03829	91.77	116.7	4.9e-34	putative acetyltransferase [EC:2.3.1.-]
CP005080.1_2785	K03790	144.10	280.5	9.3e-84	[ribosomal protein S5]-alanine N-acetyltransferase [EC:2.3.1.267]
CP005080.1_2787	K03637	63.27	285.3	3.2e-85	cyclic pyranopterin monophosphate synthase [EC:4.6.1.17]
CP005080.1_2788	K03750	141.83	506.6	2.8e-152	molybdopterin molybdotransferase [EC:2.10.1.1]
CP005080.1_2789	K00963	76.77	143.6	3.5e-42	UTP--glucose-1-phosphate uridylyltransferase [EC:2.7.7.9]
CP005080.1_2790	K01934	93.97	187.4	1.6e-55	5-formyltetrahydrofolate cyclo-ligase [EC:6.3.3.2]
CP005080.1_2791	K01434	390.80	927.5	4.5e-279	penicillin G amidase [EC:3.5.1.11]
CP005080.1_2792	K11105	246.37	652.8	1.9e-196	potassium/hydrogen antiporter
CP005080.1_2795	K00772	304.10	379.5	9.7e-114	5'-methylthioadenosine phosphorylase [EC:2.4.2.28]
CP005080.1_2797	K03282	37.57	153.9	2.2e-45	large conductance mechanosensitive channel

CP005080.1_2810	K15777	105.60	331.8	2.1e-99	4,5-DOPA dioxygenase extradiol [EC:1.13.11.-]
CP005080.1_2818	K07448	34.87	160.6	1.7e-47	restriction system protein
CP005080.1_2824	K07258	223.10	340.3	8.2e-102	serine-type D-Ala-D-Ala carboxypeptidase (penicillin-binding protein 5/6) [EC:3.4.16.4]
CP005080.1_2830	K03307	271.33	298.3	4.6e-89	solute:Na+ symporter, SSS family
CP005080.1_2838	K11779	530.17	1486.5	0	FO synthase [EC:2.5.1.147 4.3.1.32]
CP005080.1_2841	K05364	378.67	546.4	4.6e-164	penicillin-binding protein A
CP005080.1_2844	K02483	242.00	279.8	1.7e-83	two-component system, OmpR family, response regulator
CP005080.1_2848	K01434	390.80	800.4	1.1e-240	penicillin G amidase [EC:3.5.1.11]
CP005080.1_2852	K06015	126.97	360.3	7.2e-108	N-acyl-D-amino-acid deacylase [EC:3.5.1.81]
CP005080.1_2854	K00038	304.30	358.5	7.2e-108	3alpha(or 20beta)-hydroxysteroid dehydrogenase [EC:1.1.1.53]
CP005080.1_2857	K23107	334.20	358.5	2.2e-107	1-deoxyxylulose-5-phosphate synthase [EC:1.1.-.-]
CP005080.1_2858	K01728	168.73	344.8	3.9e-103	pectate lyase [EC:4.2.2.2]
CP005080.1_2859	K01051	75.37	257.7	8.7e-77	pectinesterase [EC:3.1.1.11]
CP005080.1_2860	K01728	168.73	289.3	2.5e-86	pectate lyase [EC:4.2.2.2]
CP005080.1_2861	K02027	193.17	228.3	9.5e-68	multiple sugar transport system substrate-binding protein
CP005080.1_2866	K02025	276.90	290.6	6.2e-87	multiple sugar transport system permease protein
CP005080.1_2870	K18981	273.63	390.7	4.2e-117	uronate dehydrogenase [EC:1.1.1.203]
CP005080.1_2883	K07065	61.47	90.6	4e-26	uncharacterized protein
CP005080.1_2884	K01897	430.97	485.4	1.2e-145	long-chain acyl-CoA synthetase [EC:6.2.1.3]
CP005080.1_2886	K00059	269.80	290.0	1.2e-86	3-oxoacyl-[acyl-carrier protein] reductase [EC:1.1.1.100]
CP005080.1_2893	K05375	34.70	100.4	2.2e-29	MbtH protein
CP005080.1_2896	K08217	225.00	244.4	7.4e-73	MFS transporter, DHA3 family, macrolide efflux protein
CP005080.1_2901	K18285	441.40	547.9	3.1e-165	aminodeoxyfutalosine synthase [EC:2.5.1.120]
CP005080.1_2902	K03719	131.40	178.5	6e-53	Lrp/AsnC family transcriptional regulator, leucine-responsive regulatory protein
CP005080.1_2904	K03186	92.57	277.4	7.7e-83	flavin prenyltransferase [EC:2.5.1.129]
CP005080.1_2906	K03179	196.07	298.1	3.1e-89	4-hydroxybenzoate polyprenyltransferase [EC:2.5.1.39]
CP005080.1_2907	K03182	505.87	752.4	1.3e-226	4-hydroxy-3-polyprenylbenzoate decarboxylase [EC:4.1.1.98]
CP005080.1_2911	K07399	102.77	683.0	1.5e-205	cytochrome c biogenesis protein
CP005080.1_2912	K06196	133.63	270.0	1.5e-80	cytochrome c-type biogenesis protein
CP005080.1_2913	K02199	121.53	130.9	2e-38	cytochrome c biogenesis protein CcmG, thiol:disulfide interchange protein DsbE
CP005080.1_2915	K07214	260.37	278.1	5.4e-83	iron(III)-enterobactin esterase [EC:3.1.1.108]
CP005080.1_2917	K01845	312.23	654.6	7.5e-197	glutamate-1-semialdehyde 2,1-aminomutase [EC:5.4.3.8]
CP005080.1_2922	K09122	69.30	252.7	1.9e-75	uncharacterized protein
CP005080.1_2927	K14415	269.37	467.9	2.7e-140	tRNA-splicing ligase RtcB (3'-phosphate/5'-hydroxy nucleic acid ligase) [EC:6.5.1.8]
CP005080.1_2931	K04566	242.43	563.5	2e-169	lysyl-tRNA synthetase, class I [EC:6.1.1.6]
CP005080.1_2932	K01887	107.30	614.9	9.2e-185	arginyl-tRNA synthetase [EC:6.1.1.19]
CP005080.1_2934	K06131	262.77	408.5	2.4e-122	cardiolipin synthase A/B [EC:2.7.8.-]
CP005080.1_2943	K01698	94.97	571.4	7.9e-172	porphobilinogen synthase [EC:4.2.1.24]
CP005080.1_2944	K13542	404.20	602.8	4.2e-181	uroporphyrinogen III methyltransferase / synthase [EC:2.1.1.107 4.2.1.75]
CP005080.1_2944	K01719	43.23	188.0	1.2e-55	uroporphyrinogen-III synthase [EC:4.2.1.75]
CP005080.1_2945	K01749	105.33	411.4	1.8e-123	hydroxymethylbilane synthase [EC:2.5.1.61]
CP005080.1_2946	K02492	71.10	595.9	3.1e-179	glutamyl-tRNA reductase [EC:1.2.1.70]
CP005080.1_2947	K01926	112.17	378.2	7.9e-114	redox-sensing transcriptional repressor
CP005080.1_2950	K03088	96.50	174.7	1.1e-51	RNA polymerase sigma-70 factor, ECF subfamily
CP005080.1_2957	K04768	357.53	544.4	2e-163	acetoin utilization protein AcuC
CP005080.1_2960	K00286	164.07	370.6	7.1e-111	pyrroline-5-carboxylate reductase [EC:1.5.1.2]
CP005080.1_2961	K01992	16.23	22.5	1e-05	ABC-2 type transport system permease protein
CP005080.1_2962	K01990	262.37	262.8	2.5e-78	ABC-2 type transport system ATP-binding protein
CP005080.1_2965	K12132	224.03	265.2	2.9e-79	eukaryotic-like serine/threonine-protein kinase [EC:2.7.11.1]
CP005080.1_2966	K01687	599.53	889.1	1.3e-267	dihydroxy-acid dehydratase [EC:4.2.1.9]
CP005080.1_2969	K01524	159.03	308.3	3.8e-92	exopolyphosphatase / guanosine-5'-triphosphate,3'-diphosphate pyrophosphatase [EC:3.6.1.11 3.6.1.40]
CP005080.1_2972	K04485	124.17	668.2	4.1e-201	DNA repair protein RadA/Sms
CP005080.1_2973	K07067	327.90	563.5	9.1e-170	diadenylate cyclase [EC:2.7.7.85]
CP005080.1_2974	K07497	13.93	84.0	5.1e-24	putative transposase
CP005080.1_2976	K03575	149.73	310.1	1.1e-92	A/G-specific adenine glycosylase [EC:3.2.2.31]
CP005080.1_2977	K03088	96.50	107.7	2.6e-31	RNA polymerase sigma-70 factor, ECF subfamily

CP005080.1_2979	K02483	242.00	264.1	1.1e-78	two-component system, OmpR family, response regulator
CP005080.1_2984	K03696	1063.70	1276.8	0	ATP-dependent Clp protease ATP-binding subunit ClpC
CP005080.1_2986	K26249	49.90	161.8	3.7e-48	nucleoid-associated protein Lsr2
CP005080.1_2987	K00619	168.23	233.7	5.6e-70	amino-acid N-acetyltransferase [EC:2.3.1.1]
CP005080.1_2988	K02171	45.30	89.8	8.3e-26	BlaI family transcriptional regulator, penicillinase repressor
CP005080.1_2991	K03525	120.67	277.7	6.6e-83	type III pantothenate kinase [EC:2.7.1.33]
CP005080.1_2992	K00767	217.50	395.3	1.3e-118	nicotinate-nucleotide pyrophosphorylase (carboxylating) [EC:2.4.2.19]
CP005080.1_2993	K00278	526.70	799.3	1.2e-240	L-aspartate oxidase [EC:1.4.3.16]
CP005080.1_2994	K01918	67.70	421.3	1.4e-126	pantoate--beta-alanine ligase [EC:6.3.2.1]
CP005080.1_2996	K01620	94.73	140.8	3e-41	threonine aldolase [EC:4.1.2.48]
CP005080.1_3000	K07778	170.90	171.1	1.6e-50	two-component system, NarL family, sensor histidine kinase DesK [EC:2.7.13.3]
CP005080.1_3004	K05845	152.73	300.4	8e-90	osmoprotectant transport system substrate-binding protein
CP005080.1_3005	K05846	171.73	245.4	4.9e-73	osmoprotectant transport system permease protein
CP005080.1_3006	K05846	171.73	233.5	2.1e-69	osmoprotectant transport system permease protein
CP005080.1_3007	K05847	352.27	484.2	1.4e-145	osmoprotectant transport system ATP-binding protein [EC:7.6.2.9]
CP005080.1_3010	K00796	237.57	398.5	1.2e-119	dihydropteroate synthase [EC:2.5.1.15]
CP005080.1_3012	K01633	94.10	161.2	1.3e-47	7,8-dihydroneopterin aldolase/epimerase/oxygenase [EC:4.1.2.25 5.1.99.8 1.13.11.81]
CP005080.1_3013	K00950	38.63	222.6	2.1e-66	2-amino-4-hydroxy-6-hydroxymethyldihydropteridine diphosphokinase [EC:2.7.6.3]
CP005080.1_3015	K01495	58.13	300.9	3.9e-90	GTP cyclohydrolase IA [EC:3.5.4.16]
CP005080.1_3016	K03798	767.57	968.6	1.3e-291	cell division protease FtsH [EC:3.4.24.-]
CP005080.1_3017	K00760	82.30	232.9	2.2e-69	hypoxanthine phosphoribosyltransferase [EC:2.4.2.8]
CP005080.1_3018	K04075	118.80	345.2	2.4e-103	tRNA(Ile)-lysidine synthase [EC:6.3.4.19]
CP005080.1_3020	K07259	126.90	381.3	3.3e-114	serine-type D-Ala-D-Ala carboxypeptidase/endopeptidase (penicillin-binding protein 4) [EC:3.4.16.4 3.4.21.-]
CP005080.1_3021	K01507	19.10	154.0	2.2e-45	inorganic pyrophosphatase [EC:3.6.1.1]
CP005080.1_3023	K03975	59.97	374.7	9.7e-113	membrane-associated protein
CP005080.1_3026	K03809	60.03	210.3	1.6e-62	NAD(P)H dehydrogenase (quinone) [EC:1.6.5.2]
CP005080.1_3027	K01580	317.70	394.1	4.3e-118	glutamate decarboxylase [EC:4.1.1.15]
CP005080.1_3029	K01990	262.37	342.0	2.1e-102	ABC-2 type transport system ATP-binding protein
CP005080.1_3033	K04757	53.50	71.0	4.3e-20	serine/threonine-protein kinase RsbW [EC:2.7.11.1]
CP005080.1_3038	K00344	279.00	291.1	7.1e-87	NADPH:quinone reductase [EC:1.6.5.5]
CP005080.1_3041	K03088	96.50	99.2	1e-28	RNA polymerase sigma-70 factor, ECF subfamily
CP005080.1_3043	K18546	339.70	500.7	2e-150	streptogrisin C [EC:3.4.21.-]
CP005080.1_3048	K12132	224.03	269.8	1.1e-80	eukaryotic-like serine/threonine-protein kinase [EC:2.7.11.1]
CP005080.1_3052	K06133	60.17	98.8	1.5e-28	4'-phosphopantetheinyl transferase [EC:2.7.8.-]
CP005080.1_3054	K22278	73.27	270.9	8.5e-81	peptidoglycan-N-acetylglucosamine deacetylase [EC:3.5.1.104]
CP005080.1_3057	K02078	39.80	45.1	4.4e-12	acyl carrier protein
CP005080.1_3061	K00997	102.37	114.1	3.6e-33	holo-[acyl-carrier protein] synthase [EC:2.7.8.7]
CP005080.1_3064	K07006	34.40	122.2	1.2e-35	uncharacterized protein
CP005080.1_3067	K22894	131.70	210.3	2.2e-62	SARP family transcriptional regulator, regulator of embCAB operon
CP005080.1_3068	K04757	53.50	63.0	1.2e-17	serine/threonine-protein kinase RsbW [EC:2.7.11.1]
CP005080.1_3070	K07315	145.67	152.3	7.1e-45	phosphoserine phosphatase RsbU/P [EC:3.1.3.3]
CP005080.1_3075	K03704	80.73	110.0	4.6e-32	cold shock protein
CP005080.1_3077	K11068	170.70	237.1	1.1e-70	hemolysin III
CP005080.1_3080	K02341	230.10	345.9	1.7e-103	DNA polymerase III subunit delta' [EC:2.7.7.7]
CP005080.1_3081	K00943	96.67	332.3	1.6e-99	dTMP kinase [EC:2.7.4.9]
CP005080.1_3082	K03168	448.53	938.2	2.8e-282	DNA topoisomerase I [EC:5.6.2.1]
CP005080.1_3086	K15987	877.33	969.3	5.3e-292	K(+)-stimulated pyrophosphate-energized sodium pump [EC:7.2.3.1]
CP005080.1_3087	K04757	53.50	73.8	6.2e-21	serine/threonine-protein kinase RsbW [EC:2.7.11.1]
CP005080.1_3088	K04749	63.93	146.5	4.7e-43	anti-sigma B factor antagonist
CP005080.1_3088	K06378	115.47	117.5	2.3e-34	stage II sporulation protein AA (anti-sigma F factor antagonist)
CP005080.1_3089	K06877	273.53	1135.2	0	DEAD/DEAH box helicase domain-containing protein
CP005080.1_3094	K12510	83.50	173.9	1.9e-51	tight adherence protein B
CP005080.1_3095	K02283	326.03	501.6	1.1e-150	pilus assembly protein CpaF [EC:7.4.2.8]
CP005080.1_3099	K07321	111.80	321.8	2e-96	CO dehydrogenase maturation factor
CP005080.1_3101	K01673	19.40	227.7	1e-67	carbonic anhydrase [EC:4.2.1.1]
CP005080.1_3102	K01895	785.77	1039.7	3e-313	acetyl-CoA synthetase [EC:6.2.1.1]

CP005080.1_3103	K03313	52.27	548.1	6.4e-165	Na+:H+ antiporter, NhaA family
CP005080.1_3106	K03558	39.73	57.8	4.8e-16	membrane protein required for colicin V production
CP005080.1_3108	K10773	133.83	331.0	5.5e-99	endonuclease III [EC:3.2.2.- 4.2.99.18]
CP005080.1_3109	K10914	140.63	229.4	3.7e-68	CRP/FNR family transcriptional regulator, cyclic AMP receptor protein
CP005080.1_3114	K01551	66.67	115.0	1.8e-33	arsenite/tail-anchored protein-transporting ATPase [EC:7.3.2.7 7.3.-.-]
CP005080.1_3115	K01551	66.67	152.1	1e-44	arsenite/tail-anchored protein-transporting ATPase [EC:7.3.2.7 7.3.-.-]
CP005080.1_3116	K18955	70.43	121.4	1.6e-35	WhiB family transcriptional regulator, redox-sensing transcriptional regulator
CP005080.1_3118	K09117	49.67	173.9	2.1e-51	uncharacterized protein
CP005080.1_3119	K07098	96.03	235.5	4.3e-70	uncharacterized protein
CP005080.1_3133	K01866	88.87	269.3	3.3e-80	tyrosyl-tRNA synthetase [EC:6.1.1.1]
CP005080.1_3134	K03574	70.57	81.0	3.2e-23	8-oxo-dGTP diphosphatase [EC:3.6.1.55]
CP005080.1_3135	K00273	130.37	353.2	6.7e-106	D-amino-acid oxidase [EC:1.4.3.3]
CP005080.1_3143	K00612	190.27	536.9	4.4e-161	carbamoyltransferase [EC:2.1.3.-]
CP005080.1_3146	K01885	298.30	468.9	1.7e-140	glutamyl-tRNA synthetase [EC:6.1.1.17]
CP005080.1_3149	K03427	116.20	407.8	3.9e-122	type I restriction enzyme M protein [EC:2.1.1.72]
CP005080.1_3150	K01154	44.73	66.3	9.3e-19	type I restriction enzyme, S subunit [EC:3.1.21.3]
CP005080.1_3151	K01153	169.67	520.5	5e-156	type I restriction enzyme, R subunit [EC:3.1.21.3]
CP005080.1_3161	K10914	140.63	154.9	1.7e-45	CRP/FNR family transcriptional regulator, cyclic AMP receptor protein
CP005080.1_3166	K14998	36.50	88.5	2.2e-25	surfeit locus 1 family protein
CP005080.1_3168	K03088	96.50	98.9	1.3e-28	RNA polymerase sigma-70 factor, ECF subfamily
CP005080.1_3169	K00133	214.73	454.6	1.9e-136	aspartate-semialdehyde dehydrogenase [EC:1.2.1.11]
CP005080.1_3170	K00928	394.00	516.0	3.9e-155	aspartate kinase [EC:2.7.2.4]
CP005080.1_3173	K19267	112.87	195.9	5.9e-58	NAD(P)H dehydrogenase (quinone) [EC:1.6.5.2]
CP005080.1_3175	K06187	59.67	376.2	7.4e-113	recombination protein RecR
CP005080.1_3176	K09747	46.57	156.5	2.6e-46	nucleoid-associated protein EbfC
CP005080.1_3180	K15372	445.43	837.8	2.1e-252	taurine---2-oxoglutarate transaminase [EC:2.6.1.55]
CP005080.1_3183	K12132	224.03	263.0	1.3e-78	eukaryotic-like serine/threonine-protein kinase [EC:2.7.11.1]
CP005080.1_3186	K23778	108.13	256.5	1.1e-76	TetR/AcrR family transcriptional regulator, regulator of biofilm formation and stress response
CP005080.1_3188	K00573	87.43	150.8	2e-44	protein-L-isoaspartate(D-aspartate) O-methyltransferase [EC:2.1.1.77]
CP005080.1_3200	K02338	60.60	330.6	7.9e-99	DNA polymerase III subunit beta [EC:2.7.7.7]
CP005080.1_3203	K13787	299.27	322.4	2.3e-96	geranylgeranyl diphosphate synthase, type I [EC:2.5.1.1 2.5.1.10 2.5.1.29]
CP005080.1_3205	K02040	56.17	183.5	3.4e-54	phosphate transport system substrate-binding protein
CP005080.1_3206	K01939	131.03	651.3	5.5e-196	adenylosuccinate synthase [EC:6.3.4.4]
CP005080.1_3213	K19267	112.87	180.9	2e-53	NAD(P)H dehydrogenase (quinone) [EC:1.6.5.2]
CP005080.1_3215	K01556	182.23	519.8	3e-156	kynureninase [EC:3.7.1.3]
CP005080.1_3216	K00453	59.60	317.9	4.5e-95	tryptophan 2,3-dioxygenase [EC:1.13.11.11]
CP005080.1_3218	K08166	431.20	470.6	1.8e-141	MFS transporter, DHA2 family, methylenomycin A resistance protein
CP005080.1_3219	K14155	287.10	481.1	2e-144	cysteine-S-conjugate beta-lyase [EC:4.4.1.13]
CP005080.1_3220	K01624	67.57	336.7	1.5e-100	fructose-bisphosphate aldolase, class II [EC:4.1.2.13]
CP005080.1_3221	K00762	105.33	202.3	4.5e-60	orotate phosphoribosyltransferase [EC:2.4.2.10]
CP005080.1_3222	K01785	42.83	107.3	4.1e-31	aldose 1-epimerase [EC:5.1.3.3]
CP005080.1_3224	K00797	93.83	240.5	1.4e-71	spermidine synthase [EC:2.5.1.16]
CP005080.1_3227	K14267	453.57	631.1	5.7e-190	N-succinyldiaminopimelate aminotransferase [EC:2.6.1.17]
CP005080.1_3229	K03695	1055.40	1258.0	0	ATP-dependent Clp protease ATP-binding subunit ClpB
CP005080.1_3230	K23825	78.57	262.0	2.4e-78	F420H(2)-dependent biliverdin reductase [EC:1.3.98.-]
CP005080.1_3236	K13640	95.33	195.6	4.2e-58	MerR family transcriptional regulator, heat shock protein HspR
CP005080.1_3237	K03686	406.37	427.1	5.5e-128	molecular chaperone DnaJ
CP005080.1_3238	K03687	31.27	198.4	7.6e-59	molecular chaperone GrpE
CP005080.1_3239	K04043	801.23	949.4	7.8e-286	molecular chaperone DnaK
CP005080.1_3241	K08264	318.53	348.0	2.6e-104	heterodisulfide reductase subunit D [EC:1.8.98.1]
CP005080.1_3244	K07101	118.10	178.4	5.5e-53	uncharacterized protein
CP005080.1_3245	K01494	81.60	295.0	2.2e-88	dCTP deaminase [EC:3.5.4.13]
CP005080.1_3253	K07654	329.50	480.5	3.5e-144	two-component system, OmpR family, sensor histidine kinase MtrB [EC:2.7.13.3]
CP005080.1_3254	K02483	242.00	252.3	3.9e-75	two-component system, OmpR family, response regulator
CP005080.1_3255	K03980	202.87	513.4	3.8e-154	putative peptidoglycan lipid II flippase
CP005080.1_3259	K01286	175.57	306.9	1.2e-91	D-alanyl-D-alanine carboxypeptidase [EC:3.4.16.4]

CP005080.1_3263	K00344	279.00	288.4	4.5e-86	NADPH:quinone reductase [EC:1.6.5.5]
CP005080.1_3266	K25562	450.07	1045.9	3.7e-315	L-aspartate N-monooxygenase (nitrosuccinate-forming) [EC:1.14.13.248]
CP005080.1_3267	K25597	641.80	747.6	1.8e-225	nitrosuccinate lyase [EC:4.3.99.5]
CP005080.1_3267	K01857	377.87	613.5	2e-184	3-carboxy-cis,cis-muconate cycloisomerase [EC:5.5.1.2]
CP005080.1_3269	K13063	492.93	989.8	2.2e-298	2-amino-4-deoxychorismate synthase [EC:2.6.1.86]
CP005080.1_3270	K20261	314.27	350.8	8.1e-106	trans-2,3-dihydro-3-hydroxyanthranilic acid synthase [EC:3.3.2.15]
CP005080.1_3271	K00216	256.33	381.5	2e-114	2,3-dihydro-2,3-dihydroxybenzoate dehydrogenase [EC:1.3.1.28]
CP005080.1_3272	K01626	124.93	377.8	4.5e-113	3-deoxy-7-phosphoheptulonate synthase [EC:2.5.1.54]
CP005080.1_3274	K01953	106.30	512.6	8.3e-154	asparagine synthase (glutamine-hydrolysing) [EC:6.3.5.4]
CP005080.1_3275	K00675	229.53	285.6	2.4e-85	N-hydroxyarylamine O-acetyltransferase [EC:2.3.1.118]
CP005080.1_3277	K10680	514.00	574.5	1.2e-172	N-ethylmaleimide reductase [EC:1.-.-.-]
CP005080.1_3279	K09684	188.00	195.0	1.1e-57	PucR family transcriptional regulator, purine catabolism regulatory protein
CP005080.1_3284	K07258	223.10	263.9	1.2e-78	serine-type D-Ala-D-Ala carboxypeptidase (penicillin-binding protein 5/6) [EC:3.4.16.4]
CP005080.1_3286	K02483	242.00	256.6	1.9e-76	two-component system, OmpR family, response regulator
CP005080.1_3287	K18353	128.17	338.3	3e-101	vancomycin resistance protein VanJ
CP005080.1_3288	K22894	131.70	266.7	1.6e-79	SARP family transcriptional regulator, regulator of embCAB operon
CP005080.1_3293	K01799	79.27	133.0	6.5e-39	maleate isomerase [EC:5.2.1.1]
CP005080.1_3295	K05826	31.73	43.0	1.2e-11	alpha-aminoadipate/glutamate carrier protein LysW
CP005080.1_3297	K00145	128.50	330.8	4.2e-99	N-acetyl-gamma-glutamyl-phosphate reductase [EC:1.2.1.38]
CP005080.1_3298	K05828	217.33	235.5	2.4e-70	[amino group carrier protein]-L-2-aminoadipate/L-glutamate 6-kinase [EC:2.7.2.17 2.7.2.19]
CP005080.1_3301	K04116	602.50	639.6	2e-192	cyclohexanecarboxylate-CoA ligase [EC:6.2.1.-]
CP005080.1_3307	K10947	65.70	106.1	1.1e-30	PadR family transcriptional regulator
CP005080.1_3309	K05275	257.70	363.4	6.3e-109	pyridoxine 4-dehydrogenase [EC:1.1.1.65]
CP005080.1_3310	K12251	343.87	385.2	1.7e-115	N-carbamoylputrescine amidase [EC:3.5.1.53]
CP005080.1_3312	K02199	121.53	122.5	7.3e-36	cytochrome c biogenesis protein CcmG, thiol:disulfide interchange protein DsbE
CP005080.1_3314	K07131	45.27	81.9	1.9e-23	uncharacterized protein
CP005080.1_3316	K06945	72.87	281.5	5e-84	uncharacterized protein
CP005080.1_3321	K06996	53.13	78.0	4.5e-22	uncharacterized protein
CP005080.1_3323	K02020	109.77	261.2	8.4e-78	molybdate transport system substrate-binding protein
CP005080.1_3324	K02018	245.63	600.4	2.1e-180	molybdate transport system permease protein
CP005080.1_3324	K02017	371.23	491.9	1.8e-147	molybdate transport system ATP-binding protein [EC:7.3.2.5]
CP005080.1_3326	K01446	92.93	117.2	3.3e-34	peptidoglycan recognition protein
CP005080.1_3331	K06994	260.33	716.1	2.1e-215	putative drug exporter of the RND superfamily
CP005080.1_3336	K03704	80.73	125.1	1e-36	cold shock protein
CP005080.1_3342	K17947	292.43	625.1	5.3e-188	dTDP-L-rhamnose 4-epimerase [EC:5.1.3.25]
CP005080.1_3345	K02483	242.00	296.5	1.5e-88	two-component system, OmpR family, response regulator
CP005080.1_3346	K17947	292.43	389.0	1.7e-116	dTDP-L-rhamnose 4-epimerase [EC:5.1.3.25]
CP005080.1_3347	K09931	82.73	230.9	7.8e-69	uncharacterized protein
CP005080.1_3351	K23176	55.07	645.6	2.1e-194	methylthioxylose transferase
CP005080.1_3354	K07315	145.67	190.6	1.8e-56	phosphoserine phosphatase RsbU/P [EC:3.1.3.3]
CP005080.1_3358	K07192	64.60	374.5	4.3e-112	flotillin
CP005080.1_3365	K05275	257.70	279.9	1.5e-83	pyridoxine 4-dehydrogenase [EC:1.1.1.65]
CP005080.1_3371	K07228	80.77	186.2	1.3e-55	K+:H+ antiporter subunit KhtT
CP005080.1_3372	K26732	261.93	611.5	2.8e-184	K+:H+ antiporter subunit KhtU
CP005080.1_3373	K05793	69.17	229.3	2.4e-68	tellurite resistance protein TerB
CP005080.1_3379	K01867	130.77	317.1	1e-94	tryptophanyl-tRNA synthetase [EC:6.1.1.2]
CP005080.1_3383	K00299	91.57	293.9	6.6e-88	FMN reductase [EC:1.5.1.38]
CP005080.1_3386	K25140	340.77	432.4	1.7e-130	daunorubicin/doxorubicin transport system permease protein
CP005080.1_3387	K25141	458.23	525.8	1.5e-158	daunorubicin/doxorubicin transport system ATP-binding protein
CP005080.1_3387	K01990	262.37	340.3	7.5e-102	ABC-2 type transport system ATP-binding protein
CP005080.1_3391	K04762	59.53	195.2	5.2e-58	ribosome-associated heat shock protein Hsp15
CP005080.1_3392	K07093	112.23	576.7	3.8e-173	uncharacterized protein
CP005080.1_3394	K18351	281.33	319.2	1.1e-95	two-component system, OmpR family, sensor histidine kinase VanS [EC:2.7.13.3]
CP005080.1_3396	K01874	185.23	650.9	1e-195	methionyl-tRNA synthetase [EC:6.1.1.10]
CP005080.1_3398	K01876	301.23	827.6	7.6e-249	aspartyl-tRNA synthetase [EC:6.1.1.12]
CP005080.1_3399	K07315	145.67	220.3	1.8e-65	phosphoserine phosphatase RsbU/P [EC:3.1.3.3]

CP005080.1_3400	K20469	203.00	346.7	7.8e-104	putative heme transporter
CP005080.1_3401	K06911	36.97	302.6	1.8e-90	quercetin 2,3-dioxygenase [EC:1.13.11.24]
CP005080.1_3407	K22894	131.70	245.5	4.4e-73	SARP family transcriptional regulator, regulator of embCAB operon
CP005080.1_3416	K21885	95.73	167.5	1.2e-49	ArsR family transcriptional regulator, cadmium/lead-responsive transcriptional repressor
CP005080.1_3418	K07258	223.10	276.0	2.5e-82	serine-type D-Ala-D-Ala carboxypeptidase (penicillin-binding protein 5/6) [EC:3.4.16.4]
CP005080.1_3419	K09699	429.73	549.9	1.7e-165	2-oxoisovalerate dehydrogenase E2 component (dihydrolipoyl transacylase) [EC:2.3.1.168]
CP005080.1_3420	K00167	490.37	549.2	6e-165	2-oxoisovalerate dehydrogenase E1 component subunit beta [EC:1.2.4.4]
CP005080.1_3421	K00166	419.63	509.8	5.5e-153	2-oxoisovalerate dehydrogenase E1 component subunit alpha [EC:1.2.4.4]
CP005080.1_3422	K07695	247.47	389.2	7.6e-117	two-component system, NarL family, response regulator DevR
CP005080.1_3425	K12132	224.03	354.8	1.7e-106	eukaryotic-like serine/threonine-protein kinase [EC:2.7.11.1]
CP005080.1_3425	K11912	105.07	165.4	8e-49	serine/threonine-protein kinase PpkA [EC:2.7.11.1]
CP005080.1_3426	K12132	224.03	350.4	3.8e-105	eukaryotic-like serine/threonine-protein kinase [EC:2.7.11.1]
CP005080.1_3426	K11912	105.07	191.1	1.3e-56	serine/threonine-protein kinase PpkA [EC:2.7.11.1]
CP005080.1_3429	K02003	292.97	323.0	1.1e-96	putative ABC transport system ATP-binding protein
CP005080.1_3430	K02004	52.33	65.5	1.2e-18	putative ABC transport system permease protein
CP005080.1_3432	K00344	279.00	329.6	1.5e-98	NADPH:quinone reductase [EC:1.6.5.5]
CP005080.1_3433	K03426	141.57	196.0	5e-58	NAD+ diphosphatase [EC:3.6.1.22]
CP005080.1_3434	K03750	141.83	475.2	9.6e-143	molybdopterin molybdotransferase [EC:2.10.1.1]
CP005080.1_3435	K03752	92.73	116.4	5.7e-34	molybdenum cofactor guanylyltransferase [EC:2.7.7.77]
CP005080.1_3436	K09699	429.73	528.8	4.5e-159	2-oxoisovalerate dehydrogenase E2 component (dihydrolipoyl transacylase) [EC:2.3.1.168]
CP005080.1_3437	K00167	490.37	543.4	3.4e-163	2-oxoisovalerate dehydrogenase E1 component subunit beta [EC:1.2.4.4]
CP005080.1_3438	K00166	419.63	423.1	9.7e-127	2-oxoisovalerate dehydrogenase E1 component subunit alpha [EC:1.2.4.4]
CP005080.1_3439	K03719	131.40	137.7	1.7e-40	Lrp/AsnC family transcriptional regulator, leucine-responsive regulatory protein
CP005080.1_3445	K02609	138.77	620.8	2e-187	ring-1,2-phenylacetyl-CoA epoxidase subunit PaaA [EC:1.14.13.149]
CP005080.1_3446	K02610	62.37	178.9	6.6e-53	ring-1,2-phenylacetyl-CoA epoxidase subunit PaaB
CP005080.1_3447	K02611	174.47	368.8	7.3e-111	ring-1,2-phenylacetyl-CoA epoxidase subunit PaaC [EC:1.14.13.149]
CP005080.1_3448	K02612	119.03	262.6	1.7e-78	ring-1,2-phenylacetyl-CoA epoxidase subunit PaaD
CP005080.1_3449	K02613	368.53	455.4	3.9e-137	ring-1,2-phenylacetyl-CoA epoxidase subunit PaaE
CP005080.1_3450	K22819	332.90	529.9	1.5e-159	3-oxocholest-4-en-26-oyl-CoA dehydrogenase beta subunit [EC:1.3.99.-]
CP005080.1_3455	K01118	93.70	192.6	4.1e-57	FMN-dependent NADH-azoreductase [EC:1.7.1.17]
CP005080.1_3459	K20074	131.13	388.1	4.3e-116	PPM family protein phosphatase [EC:3.1.3.16]
CP005080.1_3461	K05364	378.67	610.3	2.2e-183	penicillin-binding protein A
CP005080.1_3462	K12132	224.03	370.7	2.6e-111	eukaryotic-like serine/threonine-protein kinase [EC:2.7.11.1]
CP005080.1_3462	K08884	367.07	369.0	1.9e-110	serine/threonine protein kinase, bacterial [EC:2.7.11.1]
CP005080.1_3462	K11912	105.07	168.4	9.7e-50	serine/threonine-protein kinase PpkA [EC:2.7.11.1]
CP005080.1_3463	K07284	60.53	183.8	1.9e-54	sortase A [EC:3.4.22.70]
CP005080.1_3464	K07284	60.53	262.5	2.3e-78	sortase A [EC:3.4.22.70]
CP005080.1_3465	K01664	320.30	337.0	3.9e-101	para-aminobenzoate synthetase component II [EC:2.6.1.85]
CP005080.1_3465	K01658	241.93	297.1	3.4e-89	anthranilate synthase component II [EC:4.1.3.27]
CP005080.1_3467	K07284	60.53	202.7	3.5e-60	sortase A [EC:3.4.22.70]
CP005080.1_3474	K23778	108.13	110.3	5e-32	TetR/AcrR family transcriptional regulator, regulator of biofilm formation and stress response
CP005080.1_3495	K14059	167.87	226.6	3e-67	integrase
CP005080.1_3497	K02469	1032.73	1260.9	0	DNA gyrase subunit A [EC:5.6.2.2]
CP005080.1_3498	K02470	944.40	1011.3	8e-305	DNA gyrase subunit B [EC:5.6.2.2]
CP005080.1_3500	K03629	104.40	542.7	3.6e-163	DNA replication and repair protein RecF
CP005080.1_3501	K00033	157.37	362.1	2.4e-108	6-phosphogluconate dehydrogenase [EC:1.1.1.44 1.1.1.343]
CP005080.1_3502	K02338	60.60	424.8	2.1e-127	DNA polymerase III subunit beta [EC:2.7.7.7]
CP005080.1_3503	K02313	129.33	800.3	4.9e-241	chromosomal replication initiator protein
CP005080.1_3504	K02914	23.97	69.4	1.3e-19	large subunit ribosomal protein L34
CP005080.1_3505	K08998	23.87	142.8	5.1e-42	uncharacterized protein
CP005080.1_3506	K03217	121.07	290.7	6.1e-87	YidC/Oxa1 family membrane protein insertase
CP005080.1_3507	K06346	71.00	196.5	2.4e-58	spoIIIJ-associated protein
CP005080.1_3508	K03501	59.80	244.9	5.1e-73	16S rRNA (guanine527-N7)-methyltransferase [EC:2.1.1.170]
CP005080.1_3509	K03496	131.53	371.6	2.8e-111	chromosome partitioning protein
CP005080.1_3510	K03497	54.23	310.8	6.2e-93	ParB family transcriptional regulator, chromosome partitioning protein
CP005080.1_3512	K03671	112.43	145.9	7.7e-43	thioredoxin

CP005080.1_3513	K00384	332.10	501.4	9.7e-151	thioredoxin reductase (NADPH) [EC:1.8.1.9]
CP005080.1_3515	K03088	96.50	181.9	7.2e-54	RNA polymerase sigma-70 factor, ECF subfamily
CP005080.1_3517	K03980	202.87	652.5	3e-196	putative peptidoglycan lipid II flippase
CP005080.1_3519	K00970	344.20	503.7	3e-151	poly(A) polymerase [EC:2.7.7.19]
CP005080.1_3519	K00974	272.67	298.1	4.4e-89	tRNA nucleotidyltransferase (CCA-adding enzyme) [EC:2.7.7.72 3.1.3.- 3.1.4.-]
CP005080.1_3521	K01858	73.77	199.5	3.9e-59	myo-inositol-1-phosphate synthase [EC:5.5.1.4]
CP005080.1_3522	K10947	65.70	68.3	4.2e-19	PadR family transcriptional regulator
CP005080.1_3528	K02990	24.13	108.6	1.2e-31	small subunit ribosomal protein S6
CP005080.1_3529	K03111	28.83	141.1	2.4e-41	single-strand DNA-binding protein
CP005080.1_3530	K02963	75.13	97.9	2.8e-28	small subunit ribosomal protein S18
CP005080.1_3531	K02939	60.20	140.5	3.1e-41	large subunit ribosomal protein L9
CP005080.1_3532	K26937	267.43	368.3	2e-110	MATE family, multidrug efflux pump
CP005080.1_3533	K02314	110.70	746.8	8.2e-225	replicative DNA helicase [EC:5.6.2.3]
CP005080.1_3536	K01286	175.57	195.5	8e-58	D-alanyl-D-alanine carboxypeptidase [EC:3.4.16.4]
CP005080.1_3539	K19577	416.40	527.1	1.5e-158	MFS transporter, DHA1 family, inner membrane transport protein
CP005080.1_3544	K08972	34.93	145.9	7.4e-43	putative membrane protein
CP005080.1_3550	K03147	598.13	1188.1	0	phosphomethylpyrimidine synthase [EC:4.1.99.17]
CP005080.1_3556	K00817	293.33	419.1	1.3e-125	histidinol-phosphate aminotransferase [EC:2.6.1.9]
CP005080.1_3557	K00425	142.17	646.8	1.7e-194	cytochrome bd ubiquinol oxidase subunit I [EC:7.1.1.7]
CP005080.1_3558	K00426	28.43	450.4	2.8e-135	cytochrome bd ubiquinol oxidase subunit II [EC:7.1.1.7]
CP005080.1_3559	K16014	937.93	1720.5	0	ATP-binding cassette, subfamily C, bacterial CydCD
CP005080.1_3559	K16013	477.03	779.6	1.2e-234	ATP-binding cassette, subfamily C, bacterial CydD
CP005080.1_3559	K16012	468.53	663.5	1.3e-199	ATP-binding cassette, subfamily C, bacterial CydC
CP005080.1_3560	K07682	177.80	735.0	4.9e-221	two-component system, NarL family, sensor histidine kinase DevS [EC:2.7.13.3]
CP005080.1_3561	K21064	166.97	177.6	1.4e-52	5-amino-6-(5-phospho-D-ribitylamino)uracil phosphatase [EC:3.1.3.104]
CP005080.1_3563	K07559	140.20	282.6	1.2e-84	putative RNA 2'-phosphotransferase [EC:2.7.1.-]
CP005080.1_3567	K01990	262.37	279.3	2.4e-83	ABC-2 type transport system ATP-binding protein
CP005080.1_3569	K01990	262.37	267.8	7.4e-80	ABC-2 type transport system ATP-binding protein
CP005080.1_3572	K21064	166.97	176.9	2.1e-52	5-amino-6-(5-phospho-D-ribitylamino)uracil phosphatase [EC:3.1.3.104]
CP005080.1_3573	K01875	89.73	565.1	1e-169	seryl-tRNA synthetase [EC:6.1.1.11]
CP005080.1_3574	K04518	330.13	374.4	3.7e-112	prephenate dehydratase [EC:4.2.1.51]
CP005080.1_3575	K15733	491.53	616.5	2.3e-185	dye decolorizing peroxidase [EC:1.11.1.19]
CP005080.1_3576	K14166	176.60	694.4	6.6e-209	copper transport protein
CP005080.1_3576	K07245	176.93	178.1	9.7e-53	copper resistance protein D
CP005080.1_3576	K07156	71.17	118.7	1.5e-34	copper resistance protein C
CP005080.1_3577	K09796	24.97	141.0	2.5e-41	periplasmic copper chaperone A
CP005080.1_3578	K07152	55.90	159.3	6.9e-47	protein SCO1
CP005080.1_3581	K04757	53.50	136.1	5.8e-40	serine/threonine-protein kinase RsbW [EC:2.7.11.1]
CP005080.1_3582	K01262	268.13	328.5	4.2e-98	Xaa-Pro aminopeptidase [EC:3.4.11.9]
CP005080.1_3587	K04757	53.50	59.7	1.2e-16	serine/threonine-protein kinase RsbW [EC:2.7.11.1]
CP005080.1_3588	K01126	152.53	242.1	3.1e-72	glycerophosphoryl diester phosphodiesterase [EC:3.1.4.46]
CP005080.1_3589	K08372	423.63	532.0	6.3e-160	putative serine protease PepD [EC:3.4.21.-]
CP005080.1_3590	K03710	178.10	185.0	1.1e-54	GntR family transcriptional regulator
CP005080.1_3599	K03574	70.57	94.3	2.8e-27	8-oxo-dGTP diphosphatase [EC:3.6.1.55]
CP005080.1_3600	K03710	178.10	209.2	4.9e-62	GntR family transcriptional regulator
CP005080.1_3603	K22818	529.90	698.3	1.2e-210	3-oxocholest-4-en-26-oyl-CoA dehydrogenase alpha subunit [EC:1.3.99.-]
CP005080.1_3604	K25621	137.70	558.7	2.8e-168	3-oxo-4,17-pregnadiene-20-carboxyl-CoA hydratase alpha subunit [EC:4.2.1.-]
CP005080.1_3604	K25622	168.53	405.1	1.7e-121	3-oxo-4,17-pregnadiene-20-carboxyl-CoA hydratase beta subunit [EC:4.2.1.-]
CP005080.1_3605	K22819	332.90	531.3	6e-160	3-oxocholest-4-en-26-oyl-CoA dehydrogenase beta subunit [EC:1.3.99.-]
CP005080.1_3606	K25622	168.53	252.0	4.6e-75	3-oxo-4,17-pregnadiene-20-carboxyl-CoA hydratase beta subunit [EC:4.2.1.-]
CP005080.1_3607	K25623	440.10	703.4	2.8e-212	17-hydroxy-3-oxo-4-pregnene-20-carboxyl-CoA lyase [EC:4.1.3.-]
CP005080.1_3608	K03088	96.50	106.9	4.4e-31	RNA polymerase sigma-70 factor, ECF subfamily
CP005080.1_3613	K14059	167.87	171.6	1.4e-50	integrase
CP005080.1_3625	K06223	67.20	216.6	2.6e-64	DNA adenine methylase [EC:2.1.1.72]
CP005080.1_3684	K03885	203.30	305.0	5.2e-91	NADH:quinone reductase (non-electrogenic) [EC:1.6.5.9]
CP005080.1_3685	K03088	96.50	111.5	1.7e-32	RNA polymerase sigma-70 factor, ECF subfamily

CP005080.1_3688	K07045	129.87	204.7	8.8e-61	uncharacterized protein
CP005080.1_3689	K02483	242.00	320.7	6.8e-96	two-component system, OmpR family, response regulator
CP005080.1_3690	K02484	312.80	464.0	3.6e-139	two-component system, OmpR family, sensor kinase [EC:2.7.13.3]
CP005080.1_3695	K08167	383.90	484.1	2.3e-145	MFS transporter, DHA2 family, multidrug resistance protein
CP005080.1_3704	K03090	268.20	469.3	5.4e-141	RNA polymerase sigma-B factor
CP005080.1_3705	K03090	268.20	434.7	1.8e-130	RNA polymerase sigma-B factor
CP005080.1_3728	K14059	167.87	401.0	3.9e-120	integrase
CP005080.1_3729	K11991	164.80	250.4	2.1e-74	tRNA(adenine34) deaminase [EC:3.5.4.33]
CP005080.1_3732	K00761	217.77	332.7	9.8e-100	uracil phosphoribosyltransferase [EC:2.4.2.9]
CP005080.1_3736	K19702	277.27	319.4	2e-95	aminopeptidase S [EC:3.4.11.24]
CP005080.1_3736	K19701	238.97	276.8	1.9e-82	aminopeptidase YwaD [EC:3.4.11.6 3.4.11.10]
CP005080.1_3740	K00595	224.23	589.8	2e-177	precorrin-6B C5,15-methyltransferase / cobalt-precorrin-6B C5,C15-methyltransferase [EC:2.1.1.132 2.1.1.289 2.1.1.196]
CP005080.1_3741	K05936	234.40	383.4	5.3e-115	precorrin-4/cobalt-precorrin-4 C11-methyltransferase [EC:2.1.1.133 2.1.1.271]
CP005080.1_3749	K02188	136.40	492.0	8e-148	cobalt-precorrin-5B (C1)-methyltransferase [EC:2.1.1.195]
CP005080.1_3750	K05895	127.10	361.6	1.9e-108	precorrin-6A/cobalt-precorrin-6A reductase [EC:1.3.1.54 1.3.1.106]
CP005080.1_3751	K13540	532.73	866.2	2.7e-261	precorrin-2 C20-methyltransferase / precorrin-3B C17-methyltransferase [EC:2.1.1.130 2.1.1.131]
CP005080.1_3751	K03394	133.17	301.2	3.5e-90	precorrin-2/cobalt-factor-2 C20-methyltransferase [EC:2.1.1.130 2.1.1.151]
CP005080.1_3752	K06042	81.73	317.7	2.5e-95	precorrin-8X/cobalt-precorrin-8 methylmutase [EC:5.4.99.61 5.4.99.60]
CP005080.1_3753	K02229	250.23	491.4	1.1e-147	precorrin-3B synthase [EC:1.14.13.83]
CP005080.1_3754	K02230	1013.77	1625.8	0	cobaltochelatase CobN [EC:6.6.1.2]
CP005080.1_3762	K01286	175.57	340.7	6.8e-102	D-alanyl-D-alanine carboxypeptidase [EC:3.4.16.4]
CP005080.1_3763	K01286	175.57	330.5	8.5e-99	D-alanyl-D-alanine carboxypeptidase [EC:3.4.16.4]
CP005080.1_3764	K02343	233.40	739.8	9e-223	DNA polymerase III subunit gamma/tau [EC:2.7.7.7]
CP005080.1_3765	K01945	149.87	686.6	1.1e-206	phosphoribosylamine---glycine ligase [EC:6.3.4.13]
CP005080.1_3768	K01923	194.90	458.4	1.4e-137	phosphoribosylaminoimidazole-succinocarboxamide synthase [EC:6.3.2.6]
CP005080.1_3769	K07693	228.97	343.2	5.8e-103	two-component system, NarL family, response regulator DesR
CP005080.1_3770	K07778	170.90	348.8	1.9e-104	two-component system, NarL family, sensor histidine kinase DesK [EC:2.7.13.3]
CP005080.1_3772	K01990	262.37	298.5	3.4e-89	ABC-2 type transport system ATP-binding protein
CP005080.1_3773	K26249	49.90	151.1	7.2e-45	nucleoid-associated protein Lsr2
CP005080.1_3774	K23264	41.33	137.4	2.1e-40	phosphoribosylformylglycinamidine synthase subunit PurS [EC:6.3.5.3]
CP005080.1_3775	K23265	206.17	378.5	1.2e-113	phosphoribosylformylglycinamidine synthase subunit PurQ / glutaminase [EC:6.3.5.3 3.5.1.2]
CP005080.1_3776	K23269	711.03	1179.0	0	phosphoribosylformylglycinamidine synthase subunit PurL [EC:6.3.5.3]
CP005080.1_3780	K03668	49.83	109.6	7.1e-32	heat shock protein HslJ
CP005080.1_3781	K00764	178.17	735.6	1.8e-221	amidophosphoribosyltransferase [EC:2.4.2.14]
CP005080.1_3782	K01933	170.70	530.4	2.5e-159	phosphoribosylformylglycinamidine cyclo-ligase [EC:6.3.3.1]
CP005080.1_3784	K00271	491.07	643.6	2.9e-194	valine dehydrogenase (NAD+) [EC:1.4.1.23]
CP005080.1_3788	K03578	924.57	2064.3	0	ATP-dependent RNA helicase HrpA [EC:5.6.2.6]
CP005080.1_3791	K06196	133.63	143.9	2.5e-42	cytochrome c-type biogenesis protein
CP005080.1_3794	K06199	39.60	110.1	3.9e-32	fluoride exporter
CP005080.1_3795	K06199	39.60	94.7	1.8e-27	fluoride exporter
CP005080.1_3799	K21744	131.27	205.4	6.3e-61	MerR family transcriptional regulator, thiopeptide resistance regulator
CP005080.1_3800	K07315	145.67	148.1	1.4e-43	phosphoserine phosphatase RsbU/P [EC:3.1.3.3]
CP005080.1_3803	K24131	48.00	370.4	5.5e-111	protease PrsW [EC:3.4.-.-]
CP005080.1_3803	K07052	31.30	31.9	2.9e-08	CAAX protease family protein
CP005080.1_3804	K03439	68.23	281.5	6.2e-84	tRNA (guanine-N7-)-methyltransferase [EC:2.1.1.33]
CP005080.1_3805	K15736	575.30	710.4	1.4e-213	(S)-2-hydroxyglutarate dehydrogenase [EC:1.1.5.13]
CP005080.1_3807	K01953	106.30	115.0	1.7e-33	asparagine synthase (glutamine-hydrolysing) [EC:6.3.5.4]
CP005080.1_3810	K03885	203.30	537.3	2.8e-161	NADH:quinone reductase (non-electrogenic) [EC:1.6.5.9]
CP005080.1_3811	K07315	145.67	207.8	1.1e-61	phosphoserine phosphatase RsbU/P [EC:3.1.3.3]
CP005080.1_3831	K19302	106.60	167.7	1.5e-49	undecaprenyl-diphosphatase [EC:3.6.1.27]
CP005080.1_3834	K21600	85.87	163.5	2.2e-48	CsoR family transcriptional regulator, copper-sensing transcriptional repressor
CP005080.1_3835	K07220	65.73	240.5	1.2e-71	uncharacterized protein
CP005080.1_3836	K03306	385.20	463.5	3.6e-139	inorganic phosphate transporter, PiT family
CP005080.1_3837	K02036	260.70	432.1	4.8e-130	phosphate transport system ATP-binding protein [EC:7.3.2.1]
CP005080.1_3838	K02038	250.80	332.7	1e-99	phosphate transport system permease protein
CP005080.1_3839	K02037	256.00	347.2	7.5e-104	phosphate transport system permease protein

CP005080.1_3840	K02040	56.17	213.6	2.4e-63	phosphate transport system substrate-binding protein
CP005080.1_3843	K00937	243.67	1169.8	0	polyphosphate kinase [EC:2.7.4.1]
CP005080.1_3848	K15520	99.47	396.1	6.8e-119	mycothiol synthase [EC:2.3.1.189]
CP005080.1_3849	K01081	158.63	242.4	5.1e-72	5'-nucleotidase [EC:3.1.3.5]
CP005080.1_3853	K01426	303.90	374.8	4.4e-112	amidase [EC:3.5.1.4]
CP005080.1_3858	K13633	271.97	306.6	8.8e-92	AraC family transcriptional regulator, transcriptional activator FtrA
CP005080.1_3861	K08167	383.90	572.2	5e-172	MFS transporter, DHA2 family, multidrug resistance protein
CP005080.1_3862	K04757	53.50	60.7	6.4e-17	serine/threonine-protein kinase RsbW [EC:2.7.11.1]
CP005080.1_3868	K10680	514.00	522.0	8.8e-157	N-ethylmaleimide reductase [EC:1.-.-.-]
CP005080.1_3870	K01771	93.33	287.5	7.6e-86	1-phosphatidylinositol phosphodiesterase [EC:4.6.1.13]
CP005080.1_3874	K07653	371.23	598.7	4e-180	two-component system, OmpR family, sensor histidine kinase MprB [EC:2.7.13.3]
CP005080.1_3874	K02484	312.80	323.5	1.3e-96	two-component system, OmpR family, sensor kinase [EC:2.7.13.3]
CP005080.1_3875	K07669	325.20	399.0	3.9e-120	two-component system, OmpR family, response regulator MprA
CP005080.1_3875	K02483	242.00	294.5	6.1e-88	two-component system, OmpR family, response regulator
CP005080.1_3878	K02529	268.37	294.6	6.3e-88	LacI family transcriptional regulator, galactose operon repressor
CP005080.1_3881	K03636	36.60	66.2	1.5e-18	sulfur-carrier protein
CP005080.1_3884	K01011	189.10	284.4	5e-85	thiosulfate/3-mercaptopyruvate sulfurtransferase [EC:2.8.1.1 2.8.1.2]
CP005080.1_3887	K07092	58.83	75.2	1.6e-21	uncharacterized protein
CP005080.1_3893	K03711	142.10	146.8	3.9e-43	Fur family transcriptional regulator, ferric uptake regulator
CP005080.1_3894	K06980	199.53	339.9	1.2e-101	tRNA-modifying protein YgfZ
CP005080.1_3895	K07560	40.67	184.1	1.4e-54	D-aminoacyl-tRNA deacylase [EC:3.1.1.96]
CP005080.1_3900	K01990	262.37	329.7	1.2e-98	ABC-2 type transport system ATP-binding protein
CP005080.1_3904	K06075	115.10	125.1	1.6e-36	MarR family transcriptional regulator, transcriptional regulator for hemolysin
CP005080.1_3905	K07025	112.47	114.2	2.7e-33	putative hydrolase of the HAD superfamily
CP005080.1_3913	K26731	279.73	422.0	1.8e-126	K+:H+ antiporter
CP005080.1_3916	K22489	139.50	150.4	3.1e-44	MarR family transcriptional regulator, temperature-dependent positive regulator of motility
CP005080.1_3916	K03712	111.03	147.3	1.1e-43	MarR family transcriptional regulator, multiple antibiotic resistance protein MarR
CP005080.1_3917	K14257	326.77	733.4	5.6e-221	tetracycline 7-halogenase / FADH2 O2-dependent halogenase [EC:1.14.19.49 1.14.19.-]
CP005080.1_3918	K21185	185.03	185.4	2.6e-55	flavin reductase
CP005080.1_3919	K11263	803.03	985.1	1.5e-296	acetyl-CoA/propionyl-CoA/long-chain acyl-CoA carboxylase, biotin carboxylase, biotin carboxyl carrier protein [EC:6.4.1.2 6.4.1.3 6.4.1.- 6.3.4.14]
CP005080.1_3921	K15928	691.10	853.7	2.2e-257	bifunctional hydroxylase/dehydrase
CP005080.1_3922	K15926	154.40	189.9	3.7e-57	cyclase
CP005080.1_3923	K05551	671.33	734.4	2e-221	minimal PKS ketosynthase (KS/KS alpha) [EC:2.3.1.- 2.3.1.260 2.3.1.235]
CP005080.1_3924	K05552	565.17	686.5	3.6e-207	minimal PKS chain-length factor (CLF/KS beta) [EC:2.3.1.- 2.3.1.260 2.3.1.235]
CP005080.1_3925	K05553	64.33	129.2	1.7e-38	minimal PKS acyl carrier protein
CP005080.1_3926	K12420	314.90	461.4	3.1e-139	ketoreductase [EC:1.1.1.-]
CP005080.1_3926	K00059	269.80	287.0	9.4e-86	3-oxoacyl-[acyl-carrier protein] reductase [EC:1.1.1.100]
CP005080.1_3927	K05554	179.97	483.3	1.1e-145	aromatase [EC:4.2.1.-]
CP005080.1_3927	K15885	304.37	468.9	2.5e-141	C7-C12 aromatase (ARO/CYC) [EC:4.2.1.-]
CP005080.1_3928	K15930	799.23	1309.6	0	bifunctional oxygenase/reductase
CP005080.1_3930	K06133	60.17	164.9	1.1e-48	4'-phosphopantetheinyl transferase [EC:2.7.8.-]
CP005080.1_3932	K27095	37.00	48.7	4e-13	acyl-CoA carboxylase epsilon subunit
CP005080.1_3944	K22299	76.73	87.8	3.1e-25	HTH-type transcriptional regulator, competence development regulator
CP005080.1_3945	K16645	40.23	72.2	3.2e-20	heparin binding hemagglutinin HbhA
CP005080.1_3947	K07315	145.67	150.1	3.3e-44	phosphoserine phosphatase RsbU/P [EC:3.1.3.3]
CP005080.1_3948	K21471	158.57	225.5	8.4e-67	peptidoglycan DL-endopeptidase CwlO [EC:3.4.-.-]
CP005080.1_3950	K15521	322.10	646.1	3.3e-194	D-inositol-3-phosphate glycosyltransferase [EC:2.4.1.250]
CP005080.1_3960	K01834	148.40	377.6	2.5e-113	2,3-bisphosphoglycerate-dependent phosphoglycerate mutase [EC:5.4.2.11]
CP005080.1_3963	K02039	139.13	224.2	1.1e-66	phosphate transport system protein
CP005080.1_3964	K07768	355.67	622.7	2.6e-187	two-component system, OmpR family, sensor histidine kinase SenX3 [EC:2.7.13.3]
CP005080.1_3965	K07776	317.20	384.1	1.7e-115	two-component system, OmpR family, response regulator RegX3
CP005080.1_3965	K02483	242.00	273.9	1.1e-81	two-component system, OmpR family, response regulator
CP005080.1_3967	K07736	47.23	239.6	2.1e-71	CarD family transcriptional regulator, regulator of rRNA transcription
CP005080.1_3968	K00991	252.03	272.7	1.8e-81	2-C-methyl-D-erythritol 4-phosphate cytidylyltransferase [EC:2.7.7.60]
CP005080.1_3969	K01770	138.60	211.6	4.5e-63	2-C-methyl-D-erythritol 2,4-cyclodiphosphate synthase [EC:4.6.1.12]
CP005080.1_3971	K01883	400.67	638.8	6.2e-192	cysteinyl-tRNA synthetase [EC:6.1.1.16]

CP005080.1_3972	K03218	212.77	324.8	2.4e-97	23S rRNA (guanosine2251-2'-O)-methyltransferase [EC:2.1.1.185]
CP005080.1_3973	K16937	70.10	106.7	5.1e-31	thiosulfate dehydrogenase (quinone) large subunit [EC:1.8.5.2]
CP005080.1_3973	K15977	60.27	64.2	6.4e-18	putative oxidoreductase
CP005080.1_3976	K10112	546.20	566.8	2.2e-170	multiple sugar transport system ATP-binding protein [EC:7.5.2.-]
CP005080.1_3979	K06893	62.13	88.4	3.2e-25	uncharacterized protein
CP005080.1_3980	K19267	112.87	183.5	3.4e-54	NAD(P)H dehydrogenase (quinone) [EC:1.6.5.2]
CP005080.1_3983	K07284	60.53	83.5	6.1e-24	sortase A [EC:3.4.22.70]
CP005080.1_3986	K04343	77.20	312.8	1.8e-93	streptomycin 6-kinase [EC:2.7.1.72]
CP005080.1_3987	K00344	279.00	292.3	3e-87	NADPH:quinone reductase [EC:1.6.5.5]
CP005080.1_3999	K20273	451.77	604.0	1.1e-181	zinc metalloprotease ZmpA
CP005080.1_4000	K05795	235.30	342.4	1.2e-102	tellurium resistance protein TerD
CP005080.1_4001	K15034	126.97	185.3	3.5e-55	ribosome-associated protein
CP005080.1_4006	K01443	131.87	466.3	6.6e-140	N-acetylglucosamine-6-phosphate deacetylase [EC:3.5.1.25]
CP005080.1_4007	K25026	274.23	337.3	5.7e-101	glucokinase [EC:2.7.1.2]
CP005080.1_4008	K02027	193.17	221.9	8.2e-66	multiple sugar transport system substrate-binding protein
CP005080.1_4010	K01087	166.07	185.7	5.9e-55	trehalose 6-phosphate phosphatase [EC:3.1.3.12]
CP005080.1_4011	K00697	512.27	531.6	8.6e-160	trehalose 6-phosphate synthase [EC:2.4.1.15 2.4.1.347]
CP005080.1_4012	K13693	127.77	446.4	4.1e-134	glucosyl-3-phosphoglycerate synthase [EC:2.4.1.266]
CP005080.1_4013	K01733	243.03	328.7	2.7e-98	threonine synthase [EC:4.2.3.1]
CP005080.1_4014	K03636	36.60	75.5	2e-21	sulfur-carrier protein
CP005080.1_4015	K03704	80.73	128.0	1.4e-37	cold shock protein
CP005080.1_4016	K04077	136.23	838.9	1e-252	chaperonin GroEL [EC:5.6.1.7]
CP005080.1_4019	K01953	106.30	160.8	2.4e-47	asparagine synthase (glutamine-hydrolysing) [EC:6.3.5.4]
CP005080.1_4026	K03088	96.50	125.6	9.3e-37	RNA polymerase sigma-70 factor, ECF subfamily
CP005080.1_4032	K05350	554.93	652.2	5.4e-196	beta-glucosidase [EC:3.2.1.21]
CP005080.1_4033	K10242	321.97	407.4	9.1e-123	cellobiose transport system permease protein
CP005080.1_4033	K02026	280.30	308.2	2.8e-92	multiple sugar transport system permease protein
CP005080.1_4034	K10241	361.87	473.1	2.5e-142	cellobiose transport system permease protein
CP005080.1_4034	K02025	276.90	320.0	7e-96	multiple sugar transport system permease protein
CP005080.1_4035	K10240	310.77	558.4	4.7e-168	cellobiose transport system substrate-binding protein
CP005080.1_4036	K01180	175.60	456.8	6.4e-137	endo-1,3(4)-beta-glucanase [EC:3.2.1.6]
CP005080.1_4037	K02529	268.37	302.1	3.4e-90	LacI family transcriptional regulator, galactose operon repressor
CP005080.1_4042	K11354	453.27	737.4	1.2e-221	two-component system, chemotaxis family, sensor kinase Cph1 [EC:2.7.13.3]
CP005080.1_4044	K07315	145.67	204.6	1e-60	phosphoserine phosphatase RsbU/P [EC:3.1.3.3]
CP005080.1_4047	K02810	596.80	618.7	6.4e-186	sucrose PTS system EIIBCA or EIIBC component [EC:2.7.1.211]
CP005080.1_4048	K07106	127.00	495.7	6.4e-149	N-acetylmuramic acid 6-phosphate etherase [EC:4.2.1.126]
CP005080.1_4053	K18553	425.33	630.1	6.7e-190	MFS transporter, DHA1 family, chloramphenicol resistance protein
CP005080.1_4057	K07160	94.40	421.8	1.9e-126	5-oxoprolinase (ATP-hydrolysing) subunit A [EC:3.5.2.9]
CP005080.1_4060	K17686	799.73	1042.0	8.5e-314	P-type Cu+ transporter [EC:7.2.2.8]
CP005080.1_4060	K08364	90.13	95.8	9.6e-28	periplasmic mercuric ion binding protein
CP005080.1_4061	K06201	42.97	182.2	5.5e-54	copper homeostasis protein
CP005080.1_4064	K10843	221.37	717.0	1.1e-215	DNA excision repair protein ERCC-3 [EC:5.6.2.4]
CP005080.1_4065	K09166	75.10	340.2	3.7e-102	uncharacterized protein
CP005080.1_4069	K25286	277.13	311.5	5.1e-93	iron-siderophore transport system substrate-binding protein
CP005080.1_4069	K02016	154.03	157.7	1.8e-46	iron complex transport system substrate-binding protein
CP005080.1_4070	K23188	469.87	510.0	4.3e-153	iron-siderophore transport system ATP-binding protein [EC:7.2.2.17 7.2.2.-]
CP005080.1_4070	K02013	238.30	353.9	5.6e-106	iron complex transport system ATP-binding protein [EC:7.2.2.-]
CP005080.1_4071	K23187	358.37	425.7	1.3e-127	iron-siderophore transport system permease protein
CP005080.1_4072	K23186	409.23	480.0	4.3e-144	iron-siderophore transport system permease protein
CP005080.1_4073	K01091	112.83	142.3	9.7e-42	phosphoglycolate phosphatase [EC:3.1.3.18]
CP005080.1_4075	K03704	80.73	114.5	2e-33	cold shock protein
CP005080.1_4076	K11785	251.53	445.3	3.2e-134	5,8-dihydroxy-2-naphthoate synthase [EC:4.1.99.29]
CP005080.1_4077	K11783	110.30	333.2	7.4e-100	futalosine hydrolase [EC:3.2.2.26]
CP005080.1_4085	K12952	594.57	1365.0	0	cation-transporting P-type ATPase E [EC:7.2.2.-]
CP005080.1_4087	K06901	93.90	474.8	1.3e-142	adenine/guanine/hypoxanthine permease
CP005080.1_4090	K08217	225.00	241.4	5.7e-72	MFS transporter, DHA3 family, macrolide efflux protein

CP005080.1_4091	K13315	453.90	476.1	1.6e-143	NDP-hexose C3-ketoreductase / dTDP-4-oxo-2-deoxy-alpha-D-pentos-2-ene 2,3-reductase [EC:1.1.1.-]
CP005080.1_4091	K23107	334.20	336.2	1.4e-100	1-deoxyxylulose-5-phosphate synthase [EC:1.1.-.-]
CP005080.1_4092	K01259	136.77	425.5	2.2e-127	proline iminopeptidase [EC:3.4.11.5]
CP005080.1_4094	K12132	224.03	279.1	1.7e-83	eukaryotic-like serine/threonine-protein kinase [EC:2.7.11.1]
CP005080.1_4094	K11912	105.07	107.6	2.5e-31	serine/threonine-protein kinase PpkA [EC:2.7.11.1]
CP005080.1_4094	K02030	76.77	96.4	7.9e-28	polar amino acid transport system substrate-binding protein
CP005080.1_4099	K03088	96.50	113.9	3.3e-33	RNA polymerase sigma-70 factor, ECF subfamily
CP005080.1_4101	K00663	97.50	219.3	2.9e-65	aminoglycoside 6'-N-acetyltransferase [EC:2.3.1.82]
CP005080.1_4102	K03574	70.57	79.5	9.3e-23	8-oxo-dGTP diphosphatase [EC:3.6.1.55]
CP005080.1_4116	K03321	345.37	491.0	2.3e-147	sulfate permease, SulP family
CP005080.1_4117	K21902	113.00	113.2	5.5e-33	MerR family transcriptional regulator, repressor of the yfmOP operon
CP005080.1_4119	K07052	31.30	84.2	3.1e-24	CAAX protease family protein
CP005080.1_4121	K05349	305.00	708.0	8.6e-213	beta-glucosidase [EC:3.2.1.21]
CP005080.1_4122	K15269	141.60	397.3	2.1e-119	probable blue pigment (indigoidine) exporter
CP005080.1_4127	K13075	114.07	196.1	2.6e-58	N-acyl homoserine lactone hydrolase [EC:3.1.1.81]
CP005080.1_4132	K01990	262.37	333.9	6.2e-100	ABC-2 type transport system ATP-binding protein
CP005080.1_4135	K00573	87.43	147.7	1.9e-43	protein-L-isoaspartate(D-aspartate) O-methyltransferase [EC:2.1.1.77]
CP005080.1_4142	K06975	44.10	102.0	1.8e-29	uncharacterized protein
CP005080.1_4149	K00831	137.77	254.7	5.3e-76	phosphoserine aminotransferase [EC:2.6.1.52]
CP005080.1_4150	K20420	428.67	586.6	3.3e-176	2-hydroxy-5-methyl-1-naphthoate 7-hydroxylase [EC:1.14.15.31]
CP005080.1_4152	K06945	72.87	320.2	8.5e-96	uncharacterized protein
CP005080.1_4154	K07131	45.27	98.6	1.4e-28	uncharacterized protein
CP005080.1_4157	K18554	56.63	260.9	5.4e-78	chloramphenicol 3-O phosphotransferase [EC:2.7.1.-]
CP005080.1_4159	K11939	124.37	402.7	9.9e-121	inner membrane transporter RhtA
CP005080.1_4161	K03574	70.57	72.6	1.2e-20	8-oxo-dGTP diphosphatase [EC:3.6.1.55]
CP005080.1_4164	K01970	765.13	1017.3	4.8e-307	acyl-CoA carboxylase subunit beta [EC:6.4.1.-]
CP005080.1_4165	K27596	856.83	1063.7	0	acyl-CoA carboxylase subunit alpha [EC:6.4.1.-]
CP005080.1_4167	K01904	549.20	638.0	1e-191	4-coumarate--CoA ligase [EC:6.2.1.12]
CP005080.1_4173	K21495	31.03	44.9	5.8e-12	antitoxin FitA
CP005080.1_4175	K15520	99.47	133.5	5e-39	mycothiol synthase [EC:2.3.1.189]
CP005080.1_4183	K00275	31.30	319.1	1.5e-95	pyridoxamine 5'-phosphate oxidase [EC:1.4.3.5]
CP005080.1_4185	K02004	52.33	95.9	7.7e-28	putative ABC transport system permease protein
CP005080.1_4186	K02003	292.97	339.9	7.9e-102	putative ABC transport system ATP-binding protein
CP005080.1_4190	K18351	281.33	330.3	4.8e-99	two-component system, OmpR family, sensor histidine kinase VanS [EC:2.7.13.3]
CP005080.1_4192	K26252	197.67	386.6	2.5e-116	DtxR family transcriptional regulator, iron-dependent repressor
CP005080.1_4196	K01990	262.37	303.6	1e-90	ABC-2 type transport system ATP-binding protein
CP005080.1_4202	K01433	204.20	466.9	1.9e-140	formyltetrahydrofolate deformylase [EC:3.5.1.10]
CP005080.1_4205	K04749	63.93	96.3	1e-27	anti-sigma B factor antagonist
CP005080.1_4219	K13633	271.97	413.1	4.2e-124	AraC family transcriptional regulator, transcriptional activator FtrA
CP005080.1_4224	K16048	216.20	258.8	1.1e-77	3-hydroxy-9,10-secoandrosta-1,3,5(10)-triene-9,17-dione monooxygenase reductase component [EC:1.5.1.-]
CP005080.1_4227	K07006	34.40	144.7	1.6e-42	uncharacterized protein
CP005080.1_4228	K05337	42.27	65.6	1.9e-18	ferredoxin
CP005080.1_4229	K00529	395.20	397.0	5.7e-119	3-phenylpropionate/trans-cinnamate dioxygenase ferredoxin reductase component [EC:1.18.1.3]
CP005080.1_4230	K15866	288.97	393.0	8.1e-118	2-(1,2-epoxy-1,2-dihydrophenyl)acetyl-CoA isomerase [EC:5.3.3.18]
CP005080.1_4231	K24012	659.47	840.0	1.1e-252	acetate---CoA ligase (ADP-forming) [EC:6.2.1.13]
CP005080.1_4232	K19267	112.87	196.1	5e-58	NAD(P)H dehydrogenase (quinone) [EC:1.6.5.2]
CP005080.1_4235	K19267	112.87	208.3	9.6e-62	NAD(P)H dehydrogenase (quinone) [EC:1.6.5.2]
CP005080.1_4236	K19702	277.27	285.7	3.2e-85	aminopeptidase S [EC:3.4.11.24]
CP005080.1_4236	K19701	238.97	246.5	3e-73	aminopeptidase YwaD [EC:3.4.11.6 3.4.11.10]
CP005080.1_4238	K07222	277.37	389.7	6.6e-117	putative flavoprotein involved in K+ transport
CP005080.1_4239	K23185	221.67	379.5	5.2e-114	ferric enterobactin transport system substrate-binding protein
CP005080.1_4240	K23186	409.23	433.2	6.3e-130	iron-siderophore transport system permease protein
CP005080.1_4241	K23187	358.37	374.7	3.5e-112	iron-siderophore transport system permease protein
CP005080.1_4245	K03799	127.53	276.2	1.6e-82	heat shock protein HtpX [EC:3.4.24.-]
CP005080.1_4246	K00275	31.30	192.0	6.6e-57	pyridoxamine 5'-phosphate oxidase [EC:1.4.3.5]
CP005080.1_4249	K00540	74.27	101.1	3.3e-29	F420H(2)-dependent quinone reductase [EC:1.1.98.-]

CP005080.1_4251	K06996	53.13	119.6	9.3e-35	uncharacterized protein
CP005080.1_4252	K12132	224.03	255.4	2.7e-76	eukaryotic-like serine/threonine-protein kinase [EC:2.7.11.1]
CP005080.1_4258	K18687	599.20	866.7	2.7e-261	HIP---CoA ligase [EC:6.2.1.41]
CP005080.1_4262	K22894	131.70	303.5	1.1e-90	SARP family transcriptional regulator, regulator of embCAB operon
CP005080.1_4262	K03466	191.80	192.0	7.4e-57	DNA segregation ATPase FtsK/SpoIIIE, S-DNA-T family
CP005080.1_4265	K03704	80.73	131.2	1.4e-38	cold shock protein
CP005080.1_4266	K11782	90.73	381.6	1.2e-114	chorismate dehydratase [EC:4.2.1.151]
CP005080.1_4267	K12132	224.03	287.6	4.5e-86	eukaryotic-like serine/threonine-protein kinase [EC:2.7.11.1]
CP005080.1_4267	K11912	105.07	113.6	3.8e-33	serine/threonine-protein kinase PpkA [EC:2.7.11.1]
CP005080.1_4268	K11784	435.33	669.2	1.2e-201	cyclic dehypoxanthinyl futalosine synthase [EC:1.21.98.1]
CP005080.1_4272	K03088	96.50	99.8	6.5e-29	RNA polymerase sigma-70 factor, ECF subfamily
CP005080.1_4273	K15552	276.10	297.5	3.1e-89	taurine transport system permease protein
CP005080.1_4273	K02050	145.27	195.1	9.2e-58	NitT/TauT family transport system permease protein
CP005080.1_4274	K15551	233.63	429.0	5.8e-129	taurine transport system substrate-binding protein
CP005080.1_4274	K15553	187.27	197.1	2.8e-58	sulfonate transport system substrate-binding protein
CP005080.1_4276	K03119	329.20	389.6	2.9e-117	taurine dioxygenase [EC:1.14.11.17]
CP005080.1_4277	K03183	266.63	361.1	2.6e-108	demethylmenaquinone methyltransferase / 2-methoxy-6-polyprenyl-1,4-benzoquinol methylase [EC:2.1.1.163 2.1.1.201]
CP005080.1_4279	K21401	242.60	641.9	2.3e-193	menaquinone-9 beta-reductase [EC:1.3.99.38]
CP005080.1_4281	K03651	119.60	201.3	1e-59	3',5'-cyclic-AMP phosphodiesterase [EC:3.1.4.53]
CP005080.1_4283	K18553	425.33	547.7	6.6e-165	MFS transporter, DHA1 family, chloramphenicol resistance protein
CP005080.1_4285	K21471	158.57	178.0	2.1e-52	peptidoglycan DL-endopeptidase CwlO [EC:3.4.-.-]
CP005080.1_4286	K00330	127.67	186.3	3e-55	NADH-quinone oxidoreductase subunit A [EC:7.1.1.2]
CP005080.1_4287	K00331	263.67	303.0	7.4e-91	NADH-quinone oxidoreductase subunit B [EC:7.1.1.2]
CP005080.1_4288	K00332	197.50	219.4	3.4e-65	NADH-quinone oxidoreductase subunit C [EC:7.1.1.2]
CP005080.1_4289	K00333	518.83	653.8	6.2e-197	NADH-quinone oxidoreductase subunit D [EC:7.1.1.2]
CP005080.1_4290	K00334	152.77	291.0	5.3e-87	NADH-quinone oxidoreductase subunit E [EC:7.1.1.2]
CP005080.1_4291	K00335	601.70	736.6	6.7e-222	NADH-quinone oxidoreductase subunit F [EC:7.1.1.2]
CP005080.1_4292	K00336	705.90	923.2	7.4e-278	NADH-quinone oxidoreductase subunit G [EC:7.1.1.2]
CP005080.1_4293	K00337	403.23	569.2	3.3e-171	NADH-quinone oxidoreductase subunit H [EC:7.1.1.2]
CP005080.1_4294	K00338	139.87	269.1	1.5e-80	NADH-quinone oxidoreductase subunit I [EC:7.1.1.2]
CP005080.1_4295	K00339	129.03	233.0	1.7e-69	NADH-quinone oxidoreductase subunit J [EC:7.1.1.2]
CP005080.1_4296	K00340	94.00	148.2	7.9e-44	NADH-quinone oxidoreductase subunit K [EC:7.1.1.2]
CP005080.1_4297	K00341	553.83	807.5	7.2e-243	NADH-quinone oxidoreductase subunit L [EC:7.1.1.2]
CP005080.1_4298	K00342	541.47	645.6	2.3e-194	NADH-quinone oxidoreductase subunit M [EC:7.1.1.2]
CP005080.1_4299	K00343	338.40	569.5	3.9e-171	NADH-quinone oxidoreductase subunit N [EC:7.1.1.2]
CP005080.1_4300	K03654	618.33	792.9	1.6e-238	ATP-dependent DNA helicase RecQ [EC:5.6.2.4]
CP005080.1_4302	K01555	242.43	644.9	1.1e-193	fumarylacetoacetase [EC:3.7.1.2]
CP005080.1_4303	K06978	225.60	461.8	1.8e-138	uncharacterized protein
CP005080.1_4304	K03305	359.10	444.2	2.4e-133	proton-dependent oligopeptide transporter, POT family
CP005080.1_4307	K01990	262.37	327.3	6.2e-98	ABC-2 type transport system ATP-binding protein
CP005080.1_4321	K03823	193.23	273.9	1.6e-82	phosphinothricin acetyltransferase [EC:2.3.1.183]
CP005080.1_4327	K05786	96.17	478.4	5.6e-144	chloramphenicol-sensitive protein RarD
CP005080.1_4328	K19267	112.87	364.9	2.8e-109	NAD(P)H dehydrogenase (quinone) [EC:1.6.5.2]
CP005080.1_4330	K00175	192.63	472.0	1.5e-141	2-oxoglutarate/2-oxoacid ferredoxin oxidoreductase subunit beta [EC:1.2.7.3 1.2.7.11]
CP005080.1_4331	K00174	266.30	853.1	7.4e-257	2-oxoglutarate/2-oxoacid ferredoxin oxidoreductase subunit alpha [EC:1.2.7.3 1.2.7.11]
CP005080.1_4335	K00330	127.67	168.6	7.3e-50	NADH-quinone oxidoreductase subunit A [EC:7.1.1.2]
CP005080.1_4336	K00331	263.67	323.0	6.2e-97	NADH-quinone oxidoreductase subunit B [EC:7.1.1.2]
CP005080.1_4337	K00332	197.50	241.2	7.7e-72	NADH-quinone oxidoreductase subunit C [EC:7.1.1.2]
CP005080.1_4338	K00337	403.23	464.7	1.5e-139	NADH-quinone oxidoreductase subunit H [EC:7.1.1.2]
CP005080.1_4339	K00338	139.87	211.8	4.7e-63	NADH-quinone oxidoreductase subunit I [EC:7.1.1.2]
CP005080.1_4340	K00339	129.03	191.2	9.5e-57	NADH-quinone oxidoreductase subunit J [EC:7.1.1.2]
CP005080.1_4341	K00340	94.00	187.2	8.5e-56	NADH-quinone oxidoreductase subunit K [EC:7.1.1.2]
CP005080.1_4342	K00341	553.83	642.9	4e-193	NADH-quinone oxidoreductase subunit L [EC:7.1.1.2]
CP005080.1_4343	K00342	541.47	600.1	1.4e-180	NADH-quinone oxidoreductase subunit M [EC:7.1.1.2]
CP005080.1_4344	K00343	338.40	534.7	1.4e-160	NADH-quinone oxidoreductase subunit N [EC:7.1.1.2]
CP005080.1_4345	K03282	37.57	123.2	5.5e-36	large conductance mechanosensitive channel

CP005080.1_4346	K03799	127.53	337.4	3.8e-101	heat shock protein HtpX [EC:3.4.24.-]
CP005080.1_4350	K03919	179.97	341.8	9e-103	DNA oxidative demethylase [EC:1.14.11.33]
CP005080.1_4351	K00567	174.97	192.4	5.2e-57	methylated-DNA-[protein]-cysteine S-methyltransferase [EC:2.1.1.63]
CP005080.1_4352	K09767	72.13	237.0	7.6e-71	cyclic-di-GMP-binding protein
CP005080.1_4354	K01485	186.83	451.8	1.4e-135	cytosine/creatinine deaminase [EC:3.5.4.1 3.5.4.21]
CP005080.1_4355	K02913	23.23	60.9	4.5e-17	large subunit ribosomal protein L33
CP005080.1_4358	K00075	66.30	437.1	2.5e-131	UDP-N-acetylmuramate dehydrogenase [EC:1.3.1.98]
CP005080.1_4362	K00812	468.17	569.9	1.5e-171	aspartate aminotransferase [EC:2.6.1.1]
CP005080.1_4363	K03073	20.17	81.5	2.8e-23	preprotein translocase subunit SecE
CP005080.1_4364	K02601	101.20	290.2	6.6e-87	transcription termination/antitermination protein NusG
CP005080.1_4365	K02867	62.17	248.5	4.9e-74	large subunit ribosomal protein L11
CP005080.1_4366	K02863	85.67	300.1	9.9e-90	large subunit ribosomal protein L1
CP005080.1_4368	K02864	51.60	206.1	3.3e-61	large subunit ribosomal protein L10
CP005080.1_4369	K02935	22.77	134.7	2.1e-39	large subunit ribosomal protein L7/L12
CP005080.1_4370	K03043	656.80	1766.4	0	DNA-directed RNA polymerase subunit beta [EC:2.7.7.6]
CP005080.1_4371	K03046	932.17	2037.6	0	DNA-directed RNA polymerase subunit beta' [EC:2.7.7.6]
CP005080.1_4393	K02950	75.93	247.5	4.4e-74	small subunit ribosomal protein S12
CP005080.1_4394	K02992	105.50	207.6	9.7e-62	small subunit ribosomal protein S7
CP005080.1_4395	K02355	521.90	1091.2	0	elongation factor G
CP005080.1_4396	K02358	336.50	802.1	7.8e-242	elongation factor Tu
CP005080.1_4400	K02946	61.53	162.5	7.2e-48	small subunit ribosomal protein S10
CP005080.1_4401	K02906	104.83	330.6	7.8e-99	large subunit ribosomal protein L3
CP005080.1_4402	K02926	92.37	288.1	3.9e-86	large subunit ribosomal protein L4
CP005080.1_4403	K02892	57.40	107.7	3.4e-31	large subunit ribosomal protein L23
CP005080.1_4404	K02886	164.10	395.5	1e-118	large subunit ribosomal protein L2
CP005080.1_4405	K02965	102.33	151.0	1.2e-44	small subunit ribosomal protein S19
CP005080.1_4406	K02890	79.73	184.1	1.2e-54	large subunit ribosomal protein L22
CP005080.1_4407	K02982	103.63	363.8	2.6e-109	small subunit ribosomal protein S3
CP005080.1_4408	K02878	90.63	202.7	3e-60	large subunit ribosomal protein L16
CP005080.1_4409	K02904	59.10	95.9	9.9e-28	large subunit ribosomal protein L29
CP005080.1_4410	K02961	80.63	133.3	3.2e-39	small subunit ribosomal protein S17
CP005080.1_4411	K02874	94.83	191.6	4.3e-57	large subunit ribosomal protein L14
CP005080.1_4412	K02895	56.73	127.9	2e-37	large subunit ribosomal protein L24
CP005080.1_4413	K02931	104.27	329.8	4.8e-99	large subunit ribosomal protein L5
CP005080.1_4414	K02954	26.53	68.8	2.2e-19	small subunit ribosomal protein S14
CP005080.1_4415	K02994	80.50	187.3	1.5e-55	small subunit ribosomal protein S8
CP005080.1_4416	K02933	107.80	257.4	6.9e-77	large subunit ribosomal protein L6
CP005080.1_4417	K02881	45.33	133.7	3.2e-39	large subunit ribosomal protein L18
CP005080.1_4418	K02988	134.97	242.9	2.1e-72	small subunit ribosomal protein S5
CP005080.1_4419	K02907	40.27	69.4	1.1e-19	large subunit ribosomal protein L30
CP005080.1_4420	K02876	42.80	197.4	1.7e-58	large subunit ribosomal protein L15
CP005080.1_4421	K03076	210.97	594.7	5.8e-179	preprotein translocase subunit SecY
CP005080.1_4422	K00939	185.70	251.1	8.1e-75	adenylate kinase [EC:2.7.4.3]
CP005080.1_4423	K01265	141.83	279.5	1.6e-83	methionyl aminopeptidase [EC:3.4.11.18]
CP005080.1_4424	K02518	37.43	150.8	8e-45	translation initiation factor IF-1
CP005080.1_4425	K02919	24.63	59.4	2e-16	large subunit ribosomal protein L36
CP005080.1_4426	K02952	113.37	184.2	1.1e-54	small subunit ribosomal protein S13
CP005080.1_4427	K02948	100.47	198.7	5.4e-59	small subunit ribosomal protein S11
CP005080.1_4428	K03040	50.70	414.6	1.4e-124	DNA-directed RNA polymerase subunit alpha [EC:2.7.7.6]
CP005080.1_4429	K02879	39.00	225.6	4.3e-67	large subunit ribosomal protein L17
CP005080.1_4430	K06173	231.17	275.0	5.4e-82	tRNA pseudouridine38-40 synthase [EC:5.4.99.12]
CP005080.1_4433	K06893	62.13	81.2	4.9e-23	uncharacterized protein
CP005080.1_4436	K02871	66.60	237.7	7.6e-71	large subunit ribosomal protein L13
CP005080.1_4437	K02996	103.27	198.6	6.9e-59	small subunit ribosomal protein S9
CP005080.1_4438	K03431	454.20	655.1	4.3e-197	phosphoglucosamine mutase [EC:5.4.2.10]
CP005080.1_4440	K00867	171.90	535.8	2.4e-161	type I pantothenate kinase [EC:2.7.1.33]

CP005080.1_4442	K03088	96.50	125.4	1.1e-36	RNA polymerase sigma-70 factor, ECF subfamily
CP005080.1_4446	K04757	53.50	65.5	2.1e-18	serine/threonine-protein kinase RsbW [EC:2.7.11.1]
CP005080.1_4453	K05996	352.97	639.3	4.6e-192	carboxypeptidase T [EC:3.4.17.18]
CP005080.1_4456	K00820	183.57	839.0	1.6e-252	glutamine---fructose-6-phosphate transaminase (isomerizing) [EC:2.6.1.16]
CP005080.1_4457	K00997	102.37	132.7	7.9e-39	holo-[acyl-carrier protein] synthase [EC:2.7.8.7]
CP005080.1_4458	K23997	307.27	492.7	7.2e-148	ADP-dependent NAD(P)H-hydrate dehydratase / NAD(P)H-hydrate epimerase [EC:4.2.1.136 5.1.99.6]
CP005080.1_4460	K01775	106.13	534.3	8.4e-161	alanine racemase [EC:5.1.1.1]
CP005080.1_4462	K06925	60.83	199.5	3.6e-59	tRNA threonylcarbamoyladenosine biosynthesis protein TsaE
CP005080.1_4465	K14742	83.37	210.2	2e-62	tRNA threonylcarbamoyladenosine biosynthesis protein TsaB
CP005080.1_4466	K03789	102.70	181.2	9e-54	[ribosomal protein S18]-alanine N-acetyltransferase [EC:2.3.1.266]
CP005080.1_4467	K25706	413.33	572.5	3.5e-172	tRNA N6-adenosine threonylcarbamoyltransferase [EC:2.3.1.234]
CP005080.1_4471	K04750	56.87	63.1	1.2e-17	PhnB protein
CP005080.1_4472	K12132	224.03	277.8	4.1e-83	eukaryotic-like serine/threonine-protein kinase [EC:2.7.11.1]
CP005080.1_4475	K22278	73.27	104.6	2.4e-30	peptidoglycan-N-acetylglucosamine deacetylase [EC:3.5.1.104]
CP005080.1_4477	K04078	31.27	146.9	2.3e-43	chaperonin GroES
CP005080.1_4478	K04077	136.23	819.2	9.4e-247	chaperonin GroEL [EC:5.6.1.7]
CP005080.1_4481	K02025	276.90	277.9	4.5e-83	multiple sugar transport system permease protein
CP005080.1_4482	K02027	193.17	220.6	2e-65	multiple sugar transport system substrate-binding protein
CP005080.1_4484	K07124	220.10	334.0	4.3e-100	uncharacterized protein
CP005080.1_4487	K18955	70.43	117.9	1.8e-34	WhiB family transcriptional regulator, redox-sensing transcriptional regulator
CP005080.1_4489	K03088	96.50	116.9	4.2e-34	RNA polymerase sigma-70 factor, ECF subfamily
CP005080.1_4490	K00088	375.03	716.7	1.6e-215	IMP dehydrogenase [EC:1.1.1.205]
CP005080.1_4493	K13015	437.70	549.4	3.4e-165	UDP-N-acetyl-D-glucosamine dehydrogenase [EC:1.1.1.136]
CP005080.1_4494	K00111	151.87	600.5	1.4e-180	glycerol-3-phosphate dehydrogenase [EC:1.1.5.3]
CP005080.1_4495	K12132	224.03	295.6	1.7e-88	eukaryotic-like serine/threonine-protein kinase [EC:2.7.11.1]
CP005080.1_4495	K11912	105.07	130.9	2.1e-38	serine/threonine-protein kinase PpkA [EC:2.7.11.1]
CP005080.1_4497	K12132	224.03	306.2	1e-91	eukaryotic-like serine/threonine-protein kinase [EC:2.7.11.1]
CP005080.1_4497	K11912	105.07	127.2	2.8e-37	serine/threonine-protein kinase PpkA [EC:2.7.11.1]
CP005080.1_4499	K03333	328.20	681.8	3.7e-205	cholesterol oxidase [EC:1.1.3.6]
CP005080.1_4501	K04093	56.13	67.7	5.9e-19	chorismate mutase [EC:5.4.99.5]
CP005080.1_4502	K01951	100.47	752.2	2.7e-226	GMP synthase (glutamine-hydrolysing) [EC:6.3.5.2]
CP005080.1_4506	K16937	70.10	172.2	5.2e-51	thiosulfate dehydrogenase (quinone) large subunit [EC:1.8.5.2]
CP005080.1_4512	K21471	158.57	271.8	7.3e-81	peptidoglycan DL-endopeptidase CwlO [EC:3.4.-.-]
CP005080.1_4513	K21471	158.57	260.2	2.6e-77	peptidoglycan DL-endopeptidase CwlO [EC:3.4.-.-]
CP005080.1_4514	K03657	367.57	819.7	1.2e-246	ATP-dependent DNA helicase UvrD/PcrA [EC:5.6.2.4]
CP005080.1_4516	K01046	52.07	65.1	2.9e-18	triacylglycerol lipase [EC:3.1.1.3]
CP005080.1_4517	K25821	227.33	255.6	2.8e-77	isobutyryl-CoA mutase small subunit [EC:5.4.99.13]
CP005080.1_4517	K01849	173.00	222.5	5.6e-67	methylmalonyl-CoA mutase, C-terminal domain [EC:5.4.99.2]
CP005080.1_4523	K01903	498.17	570.8	7.9e-172	succinyl-CoA synthetase beta subunit [EC:6.2.1.5]
CP005080.1_4524	K01902	347.77	457.1	1.8e-137	succinyl-CoA synthetase alpha subunit [EC:6.2.1.5]
CP005080.1_4525	K07726	44.40	45.7	2.2e-12	putative transcriptional regulator
CP005080.1_4528	K11175	195.63	309.2	1.4e-92	phosphoribosylglycinamide formyltransferase 1 [EC:2.1.2.2]
CP005080.1_4529	K00602	115.70	778.0	2.5e-234	phosphoribosylaminoimidazolecarboxamide formyltransferase / IMP cyclohydrolase [EC:2.1.2.3 3.5.4.10]
CP005080.1_4532	K01491	414.73	442.2	7.8e-133	methylenetetrahydrofolate dehydrogenase (NADP+) / methenyltetrahydrofolate cyclohydrolase [EC:1.5.1.5 3.5.4.9]
CP005080.1_4535	K00024	377.73	445.4	9.4e-134	malate dehydrogenase [EC:1.1.1.37]
CP005080.1_4540	K00375	343.30	609.4	2.6e-183	GntR family transcriptional regulator / MocR family aminotransferase
CP005080.1_4544	K00819	481.33	667.9	4.5e-201	ornithine--oxo-acid transaminase [EC:2.6.1.13]
CP005080.1_4545	K01867	130.77	457.6	2.7e-137	tryptophanyl-tRNA synthetase [EC:6.1.1.2]
CP005080.1_4547	K16652	189.47	411.5	6.8e-124	decaprenylphospho-beta-D-erythro-pentofuranosid-2-ulose 2-reductase [EC:1.1.1.333]
CP005080.1_4548	K16653	252.03	607.4	7.1e-183	decaprenylphospho-beta-D-ribofuranose 2-oxidase [EC:1.1.98.3]
CP005080.1_4549	K07058	40.93	181.8	1.2e-53	membrane protein
CP005080.1_4550	K07258	223.10	302.7	2.1e-90	serine-type D-Ala-D-Ala carboxypeptidase (penicillin-binding protein 5/6) [EC:3.4.16.4]
CP005080.1_4552	K07098	96.03	368.5	1.9e-110	uncharacterized protein
CP005080.1_4553	K02035	249.67	290.8	8.9e-87	peptide/nickel transport system substrate-binding protein
CP005080.1_4559	K00240	157.40	344.1	2.4e-103	succinate dehydrogenase iron-sulfur subunit [EC:1.3.5.1]
CP005080.1_4560	K00239	665.27	840.4	3.3e-253	succinate dehydrogenase flavoprotein subunit [EC:1.3.5.1]

CP005080.1_4561	K00242	59.53	172.1	3.9e-51	succinate dehydrogenase membrane anchor subunit
CP005080.1_4562	K00241	50.80	81.4	3.2e-23	succinate dehydrogenase cytochrome b subunit
CP005080.1_4563	K16840	169.10	207.9	8.7e-62	2-oxo-4-hydroxy-4-carboxy-5-ureidoimidazoline decarboxylase [EC:4.1.1.97]
CP005080.1_4564	K12373	123.03	331.9	2.7e-99	hexosaminidase [EC:3.2.1.52]
CP005080.1_4568	K19809	322.23	521.4	2e-157	2-methyl-1-pyrroline reductase [EC:1.5.1.48]
CP005080.1_4573	K23747	1366.60	2164.7	0	spectinabilin polyketide synthase system NorA [EC:2.3.1.290]
CP005080.1_4573	K20786	1671.70	1676.5	0	niddamycin polyketide synthase 2
CP005080.1_4573	K00645	247.17	249.1	3.9e-74	[acyl-carrier-protein] S-malonyltransferase [EC:2.3.1.39]
CP005080.1_4578	K21185	185.03	241.6	1.5e-72	flavin reductase
CP005080.1_4580	K22894	131.70	282.0	3.7e-84	SARP family transcriptional regulator, regulator of embCAB operon
CP005080.1_4582	K22894	131.70	278.5	4.2e-83	SARP family transcriptional regulator, regulator of embCAB operon
CP005080.1_4583	K12242	248.83	270.5	3.7e-81	pyochelin biosynthesis protein PchC
CP005080.1_4585	K22894	131.70	252.1	4.4e-75	SARP family transcriptional regulator, regulator of embCAB operon
CP005080.1_4587	K02563	193.67	360.8	4.7e-108	UDP-N-acetylglucosamine--N-acetylmuramyl-(pentapeptide) pyrophosphoryl-undecaprenol N-acetylglucosamine transferase [EC:2.4.1.227]
CP005080.1_4588	K03088	96.50	166.9	2.6e-49	RNA polymerase sigma-70 factor, ECF subfamily
CP005080.1_4591	K11250	181.27	310.7	1.7e-93	leucine efflux protein
CP005080.1_4593	K01186	251.07	352.9	1.2e-105	sialidase-1 [EC:3.2.1.18]
CP005080.1_4594	K16568	159.77	182.1	6.3e-54	exopolysaccharide production protein ExoZ
CP005080.1_4599	K01654	406.17	535.1	1.1e-160	N-acetylneuraminate synthase [EC:2.5.1.56]
CP005080.1_4602	K07335	118.73	342.3	2.2e-102	basic membrane protein A and related proteins
CP005080.1_4603	K23537	596.97	808.4	3.1e-243	general nucleoside transport system ATP-binding protein
CP005080.1_4604	K23535	209.57	375.4	1.7e-112	general nucleoside transport system permease protein
CP005080.1_4605	K23536	183.10	419.8	3.5e-126	general nucleoside transport system permease protein
CP005080.1_4606	K01489	31.20	174.8	1.1e-51	cytidine deaminase [EC:3.5.4.5]
CP005080.1_4607	K00758	548.07	588.5	5.2e-177	thymidine phosphorylase [EC:2.4.2.4]
CP005080.1_4608	K17734	342.83	361.9	2.6e-108	serine protease AprX [EC:3.4.21.-]
CP005080.1_4610	K03088	96.50	167.3	1.9e-49	RNA polymerase sigma-70 factor, ECF subfamily
CP005080.1_4612	K08224	171.37	531.4	8.5e-160	MFS transporter, YNFM family, putative membrane transport protein
CP005080.1_4615	K01488	340.33	462.6	6.1e-139	adenosine deaminase [EC:3.5.4.4]
CP005080.1_4620	K07654	329.50	567.7	1.4e-170	two-component system, OmpR family, sensor histidine kinase MtrB [EC:2.7.13.3]
CP005080.1_4621	K02483	242.00	253.3	1.9e-75	two-component system, OmpR family, response regulator
CP005080.1_4622	K03088	96.50	132.4	7.8e-39	RNA polymerase sigma-70 factor, ECF subfamily
CP005080.1_4629	K00128	593.30	625.4	3.2e-188	aldehyde dehydrogenase (NAD+) [EC:1.2.1.3]
CP005080.1_4630	K01619	39.37	289.7	1.5e-86	deoxyribose-phosphate aldolase [EC:4.1.2.4]
CP005080.1_4632	K01840	449.03	643.5	2.1e-193	phosphomannomutase [EC:5.4.2.8]
CP005080.1_4633	K03783	165.87	326.7	1.2e-97	purine-nucleoside phosphorylase [EC:2.4.2.1]
CP005080.1_4635	K23842	484.43	776.8	2.8e-234	NAD(P)H dehydrogenase (quinone) [EC:1.6.5.2]
CP005080.1_4638	K11263	803.03	995.7	9.4e-300	acetyl-CoA/propionyl-CoA/long-chain acyl-CoA carboxylase, biotin carboxylase, biotin carboxyl carrier protein [EC:6.4.1.2 6.4.1.3 6.4.1.- 6.3.4.14]
CP005080.1_4640	K06287	207.20	280.5	7.8e-84	nucleoside triphosphate pyrophosphatase [EC:3.6.1.-]
CP005080.1_4642	K27095	37.00	59.4	2e-16	acyl-CoA carboxylase epsilon subunit
CP005080.1_4643	K27094	871.70	984.5	4.1e-297	propionyl-CoA/long-chain acyl-CoA carboxylase carboxyl transferase subunit [EC:6.4.1.3 6.4.1.- 2.1.3.15]
CP005080.1_4644	K03524	154.23	283.2	1.3e-84	BirA family transcriptional regulator, biotin operon repressor / biotin---[acetyl-CoA-carboxylase] ligase [EC:6.3.4.15]
CP005080.1_4648	K01745	410.10	703.2	1.1e-211	histidine ammonia-lyase [EC:4.3.1.3]
CP005080.1_4655	K00262	473.37	822.2	2.7e-247	glutamate dehydrogenase (NADP+) [EC:1.4.1.4]
CP005080.1_4656	K27043	96.73	115.6	1.3e-33	XRE family transcriptional regulator, stress-response regulator
CP005080.1_4659	K00433	197.80	439.1	5.3e-132	non-heme chloroperoxidase [EC:1.11.1.10]
CP005080.1_4663	K07304	43.27	295.5	2.1e-88	peptide-methionine (S)-S-oxide reductase [EC:1.8.4.11]
CP005080.1_4665	K01760	493.17	516.9	1.7e-155	cysteine-S-conjugate beta-lyase [EC:4.4.1.13]
CP005080.1_4665	K01739	420.37	480.9	2.2e-144	cystathionine gamma-synthase [EC:2.5.1.48]
CP005080.1_4669	K07315	145.67	173.2	3.4e-51	phosphoserine phosphatase RsbU/P [EC:3.1.3.3]
CP005080.1_4671	K05518	113.90	128.9	6e-38	phosphoserine phosphatase RsbX [EC:3.1.3.3]
CP005080.1_4671	K17752	96.77	112.0	9.9e-33	serine/threonine-protein kinase RsbT [EC:2.7.11.1]
CP005080.1_4672	K17752	96.77	203.0	1.5e-60	serine/threonine-protein kinase RsbT [EC:2.7.11.1]
CP005080.1_4673	K17762	98.20	195.2	1.6e-58	rsbT antagonist protein RsbS
CP005080.1_4674	K17763	101.07	256.7	1.9e-76	rsbT co-antagonist protein RsbR
CP005080.1_4676	K01754	266.27	515.5	7.3e-155	threonine dehydratase [EC:4.3.1.19]

CP005080.1_4677	K01990	262.37	350.0	8.1e-105	ABC-2 type transport system ATP-binding protein
CP005080.1_4679	K03624	159.57	177.5	1.2e-52	transcription elongation factor GreA
CP005080.1_4681	K18455	288.60	529.3	3.7e-159	mycothiol S-conjugate amidase [EC:3.5.1.115]
CP005080.1_4687	K06888	62.67	1037.3	4.3e-312	uncharacterized protein
CP005080.1_4688	K11068	170.70	258.5	3.4e-77	hemolysin III
CP005080.1_4689	K01596	159.43	1085.2	0	phosphoenolpyruvate carboxykinase (GTP) [EC:4.1.1.32]
CP005080.1_4695	K14260	327.23	741.1	6e-223	alanine-synthesizing transaminase [EC:2.6.1.66 2.6.1.2]
CP005080.1_4696	K00675	229.53	314.2	4.7e-94	N-hydroxyarylamine O-acetyltransferase [EC:2.3.1.118]
CP005080.1_4702	K06015	126.97	687.6	7.5e-207	N-acyl-D-amino-acid deacylase [EC:3.5.1.81]
CP005080.1_4704	K00874	245.93	512.8	6.8e-154	2-dehydro-3-deoxygluconokinase [EC:2.7.1.45]
CP005080.1_4706	K09022	107.97	119.1	1.1e-34	2-iminobutanoate/2-iminopropanoate deaminase [EC:3.5.99.10]
CP005080.1_4707	K03299	88.80	571.1	7.4e-172	gluconate:H+ symporter, GntP family
CP005080.1_4711	K02279	51.03	127.1	3.8e-37	pilus assembly protein CpaB
CP005080.1_4712	K02282	178.33	363.9	5.5e-109	pilus assembly protein CpaE
CP005080.1_4715	K02283	326.03	550.3	1.9e-165	pilus assembly protein CpaF [EC:7.4.2.8]
CP005080.1_4716	K12510	83.50	116.1	7.2e-34	tight adherence protein B
CP005080.1_4717	K12511	139.07	209.9	2.1e-62	tight adherence protein C
CP005080.1_4720	K02651	27.23	29.5	3.4e-07	pilus assembly protein Flp/PilA
CP005080.1_4730	K09005	27.23	105.7	1.3e-30	uncharacterized protein
CP005080.1_4731	K02654	116.93	166.4	4.3e-49	leader peptidase (prepilin peptidase) / N-methyltransferase [EC:3.4.23.43 2.1.1.-]
CP005080.1_4735	K19265	304.10	640.7	7.4e-193	L-glyceraldehyde 3-phosphate reductase [EC:1.1.1.-]
CP005080.1_4736	K12503	333.63	450.7	1.1e-135	short-chain Z-isoprenyl diphosphate synthase [EC:2.5.1.68]
CP005080.1_4737	K07175	228.10	596.0	3.9e-179	PhoH-like ATPase
CP005080.1_4739	K20469	203.00	563.2	1.7e-169	putative heme transporter
CP005080.1_4740	K04756	94.07	276.2	6.3e-83	lipoyl-dependent peroxiredoxin subunit D [EC:1.11.1.28]
CP005080.1_4741	K24126	220.03	304.0	1.2e-91	lipoyl-dependent peroxiredoxin subunit C [EC:1.11.1.28]
CP005080.1_4742	K04761	248.97	465.3	8.8e-140	LysR family transcriptional regulator, hydrogen peroxide-inducible genes activator
CP005080.1_4744	K02003	292.97	314.2	5e-94	putative ABC transport system ATP-binding protein
CP005080.1_4749	K07315	145.67	218.5	6.6e-65	phosphoserine phosphatase RsbU/P [EC:3.1.3.3]
CP005080.1_4749	K04757	53.50	55.1	3.1e-15	serine/threonine-protein kinase RsbW [EC:2.7.11.1]
CP005080.1_4751	K01679	639.40	754.2	8e-227	fumarate hydratase, class II [EC:4.2.1.2]
CP005080.1_4754	K01676	363.23	1004.7	1.3e-302	fumarate hydratase, class I [EC:4.2.1.2]
CP005080.1_4756	K18955	70.43	89.3	1.2e-25	WhiB family transcriptional regulator, redox-sensing transcriptional regulator
CP005080.1_4757	K02446	462.67	558.2	4.6e-168	fructose-1,6-bisphosphatase II [EC:3.1.3.11]
CP005080.1_4759	K09019	116.73	285.2	1e-85	3-hydroxypropanoate dehydrogenase [EC:1.1.1.-]
CP005080.1_4760	K03602	21.00	86.2	7.9e-25	exodeoxyribonuclease VII small subunit [EC:3.1.11.6]
CP005080.1_4761	K03601	56.10	449.0	1.2e-134	exodeoxyribonuclease VII large subunit [EC:3.1.11.6]
CP005080.1_4762	K14052	318.30	345.2	1.6e-103	putrescine importer
CP005080.1_4763	K03527	70.27	501.9	8.8e-151	4-hydroxy-3-methylbut-2-en-1-yl diphosphate reductase [EC:1.17.7.4]
CP005080.1_4764	K00886	141.63	429.8	3.2e-129	polyphosphate glucokinase [EC:2.7.1.63]
CP005080.1_4766	K06942	430.83	505.3	8.5e-152	ribosome-binding ATPase
CP005080.1_4773	K06880	109.97	186.5	3.7e-55	erythromycin esterase [EC:3.1.1.-]
CP005080.1_4775	K01733	243.03	340.8	6e-102	threonine synthase [EC:4.2.3.1]
CP005080.1_4777	K01183	115.40	313.3	1.4e-93	chitinase [EC:3.2.1.14]
CP005080.1_4782	K07315	145.67	208.9	5.4e-62	phosphoserine phosphatase RsbU/P [EC:3.1.3.3]
CP005080.1_4782	K04757	53.50	62.6	1.6e-17	serine/threonine-protein kinase RsbW [EC:2.7.11.1]
CP005080.1_4783	K03969	84.50	198.6	8e-59	phage shock protein A
CP005080.1_4784	K06872	48.37	57.7	4e-16	uncharacterized protein
CP005080.1_4785	K20534	338.93	489.6	3.5e-147	polyisoprenyl-phosphate glycosyltransferase [EC:2.4.-.-]
CP005080.1_4792	K08640	66.53	206.8	1.5e-61	zinc D-Ala-D-Ala carboxypeptidase [EC:3.4.17.14]
CP005080.1_4797	K01487	305.77	465.4	1.5e-139	guanine deaminase [EC:3.5.4.3]
CP005080.1_4807	K01689	269.20	701.6	5.9e-211	enolase 1/2/3 [EC:4.2.1.11]
CP005080.1_4817	K02026	280.30	366.8	4.6e-110	multiple sugar transport system permease protein
CP005080.1_4818	K02025	276.90	368.6	1.3e-110	multiple sugar transport system permease protein
CP005080.1_4819	K02027	193.17	298.7	4.1e-89	multiple sugar transport system substrate-binding protein
CP005080.1_4820	K01060	117.80	301.0	5.5e-90	cephalosporin-C deacetylase [EC:3.1.1.41]

CP005080.1_4822	K02529	268.37	314.8	4.7e-94	LacI family transcriptional regulator, galactose operon repressor
CP005080.1_4823	K02035	249.67	297.4	9.1e-89	peptide/nickel transport system substrate-binding protein
CP005080.1_4824	K06207	327.10	962.6	4.8e-290	GTP-binding protein
CP005080.1_4826	K15580	368.50	411.6	3.1e-123	oligopeptide transport system substrate-binding protein
CP005080.1_4826	K02035	249.67	297.3	9.5e-89	peptide/nickel transport system substrate-binding protein
CP005080.1_4827	K15581	345.63	365.1	1.7e-109	oligopeptide transport system permease protein
CP005080.1_4827	K02033	263.63	342.6	1.6e-102	peptide/nickel transport system permease protein
CP005080.1_4828	K02034	256.80	299.5	1.6e-89	peptide/nickel transport system permease protein
CP005080.1_4829	K02031	412.13	511.0	1.9e-153	peptide/nickel transport system ATP-binding protein
CP005080.1_4830	K02032	420.67	543.3	4.2e-163	peptide/nickel transport system ATP-binding protein
CP005080.1_4830	K10823	502.03	516.1	6.4e-155	oligopeptide transport system ATP-binding protein
CP005080.1_4830	K12372	463.00	476.4	5.5e-143	dipeptide transport system ATP-binding protein
CP005080.1_4831	K15580	368.50	395.1	2.9e-118	oligopeptide transport system substrate-binding protein
CP005080.1_4831	K02035	249.67	259.0	3.7e-77	peptide/nickel transport system substrate-binding protein
CP005080.1_4832	K15581	345.63	369.9	6e-111	oligopeptide transport system permease protein
CP005080.1_4832	K02033	263.63	354.7	3.4e-106	peptide/nickel transport system permease protein
CP005080.1_4833	K15582	364.50	403.6	1.9e-121	oligopeptide transport system permease protein
CP005080.1_4833	K02034	256.80	307.6	5.9e-92	peptide/nickel transport system permease protein
CP005080.1_4834	K15583	519.70	531.4	8e-160	oligopeptide transport system ATP-binding protein
CP005080.1_4834	K02031	412.13	506.7	3.8e-152	peptide/nickel transport system ATP-binding protein
CP005080.1_4834	K02032	420.67	423.0	1e-126	peptide/nickel transport system ATP-binding protein
CP005080.1_4835	K02032	420.67	545.5	9.1e-164	peptide/nickel transport system ATP-binding protein
CP005080.1_4835	K10823	502.03	518.1	1.6e-155	oligopeptide transport system ATP-binding protein
CP005080.1_4835	K12372	463.00	472.7	7e-142	dipeptide transport system ATP-binding protein
CP005080.1_4838	K15525	261.80	425.6	8.2e-128	N-acetyl-1-D-myo-inositol-2-amino-2-deoxy-alpha-D-glucopyranoside deacetylase [EC:3.5.1.103]
CP005080.1_4844	K00661	129.97	289.6	8.9e-87	maltose O-acetyltransferase [EC:2.3.1.79]
CP005080.1_4847	K24914	586.77	1058.5	8.9e-319	class III lanthionine synthetase [EC:3.13.2.4]
CP005080.1_4848	K24913	37.23	62.8	8.2e-18	lanthionine-containing peptide SapB
CP005080.1_4849	K24910	454.77	789.2	1e-237	ATP-binding cassette, subfamily B, bacterial RamA/AmfB
CP005080.1_4850	K24911	467.63	826.8	4.4e-249	ATP-binding cassette, subfamily B, bacterial RamB/AmfA
CP005080.1_4851	K24912	242.67	251.6	2.2e-75	LuxR family transcriptional regulator, transcriptional activator for aerial hyphae formation
CP005080.1_4853	K22476	94.77	409.4	5.3e-123	N-acetylglutamate synthase [EC:2.3.1.1]
CP005080.1_4855	K14254	527.30	564.9	2.6e-170	aminotransferase
CP005080.1_4858	K01439	285.40	308.6	4.1e-92	succinyl-diaminopimelate desuccinylase [EC:3.5.1.18]
CP005080.1_4860	K00796	237.57	322.7	1.3e-96	dihydropteroate synthase [EC:2.5.1.15]
CP005080.1_4862	K01246	36.57	255.9	1.5e-76	DNA-3-methyladenine glycosylase I [EC:3.2.2.20]
CP005080.1_4863	K15866	288.97	340.1	8.7e-102	2-(1,2-epoxy-1,2-dihydrophenyl)acetyl-CoA isomerase [EC:5.3.3.18]
CP005080.1_4866	K03088	96.50	195.1	6.7e-58	RNA polymerase sigma-70 factor, ECF subfamily
CP005080.1_4868	K08372	423.63	518.5	7.7e-156	putative serine protease PepD [EC:3.4.21.-]
CP005080.1_4869	K03117	74.50	81.9	3.1e-23	sec-independent protein translocase protein TatB
CP005080.1_4871	K03593	111.47	430.6	3.6e-129	ATP-binding protein involved in chromosome partitioning
CP005080.1_4873	K06213	106.03	229.5	2.6e-68	magnesium transporter
CP005080.1_4876	K03284	165.70	418.1	2.3e-125	magnesium transporter
CP005080.1_4878	K08221	232.17	531.2	6.3e-160	MFS transporter, ACDE family, multidrug resistance protein
CP005080.1_4880	K07053	178.93	343.9	7.9e-103	3',5'-nucleoside bisphosphate phosphatase [EC:3.1.3.97]
CP005080.1_4881	K05595	81.43	164.1	1.6e-48	multiple antibiotic resistance protein
CP005080.1_4893	K02035	249.67	335.1	3.5e-100	peptide/nickel transport system substrate-binding protein
CP005080.1_4894	K02034	256.80	275.6	2.8e-82	peptide/nickel transport system permease protein
CP005080.1_4895	K02033	263.63	331.5	3.8e-99	peptide/nickel transport system permease protein
CP005080.1_4896	K02031	412.13	772.0	1.8e-232	peptide/nickel transport system ATP-binding protein
CP005080.1_4896	K02032	420.67	429.6	1.1e-128	peptide/nickel transport system ATP-binding protein
CP005080.1_4897	K21011	221.50	318.3	3.7e-95	polysaccharide biosynthesis protein PelF
CP005080.1_4899	K21214	236.10	502.5	2.1e-151	NDP-hexose 4-ketoreductase
CP005080.1_4901	K21147	410.60	539.8	2.3e-162	sulfur-carrier protein adenylyltransferase/sulfurtransferase [EC:2.7.7.80 2.7.7.- 2.8.1.11 2.8.1.-]
CP005080.1_4901	K21029	316.43	344.7	2.6e-103	molybdopterin-synthase adenylyltransferase [EC:2.7.7.80]
CP005080.1_4904	K07497	13.93	84.0	5.1e-24	putative transposase

CP005080.1_4905	K20468	162.10	163.4	3.4e-48	putative heme transporter
CP005080.1_4905	K07027	85.43	131.0	2.8e-38	glycosyltransferase 2 family protein
CP005080.1_4907	K03657	367.57	377.7	5.4e-113	ATP-dependent DNA helicase UvrD/PcrA [EC:5.6.2.4]
CP005080.1_4907	K16899	216.03	241.9	8e-72	ATP-dependent helicase/nuclease subunit B [EC:5.6.2.4 3.1.-.-]
CP005080.1_4908	K03657	367.57	581.0	1.8e-174	ATP-dependent DNA helicase UvrD/PcrA [EC:5.6.2.4]
CP005080.1_4910	K03426	141.57	411.1	3.9e-123	NAD+ diphosphatase [EC:3.6.1.22]
CP005080.1_4911	K18917	71.77	129.8	2.7e-38	mycoredoxin [EC:1.20.4.3]
CP005080.1_4912	K03657	367.57	489.0	1.2e-146	ATP-dependent DNA helicase UvrD/PcrA [EC:5.6.2.4]
CP005080.1_4914	K18958	79.27	155.1	1e-45	WhiB family transcriptional regulator, redox-sensing transcriptional regulator
CP005080.1_4926	K03635	25.83	181.6	1.1e-53	molybdopterin synthase catalytic subunit [EC:2.8.1.12]
CP005080.1_4928	K07177	99.10	434.2	1.8e-130	Lon-like protease
CP005080.1_4930	K09118	184.20	1317.9	0	uncharacterized protein
CP005080.1_4931	K07126	64.03	95.4	1.6e-27	uncharacterized protein
CP005080.1_4932	K22297	134.93	149.6	3.7e-44	Fur family transcriptional regulator, stress-responsive regulator
CP005080.1_4933	K03781	68.70	780.7	5.9e-235	catalase [EC:1.11.1.6]
CP005080.1_4935	K05602	293.03	425.9	3.7e-128	histidinol-phosphatase [EC:3.1.3.15]
CP005080.1_4937	K11741	110.40	131.0	1.4e-38	quaternary ammonium compound-resistance protein SugE
CP005080.1_4938	K06949	90.53	346.7	7.4e-104	ribosome biogenesis GTPase / thiamine phosphate phosphatase [EC:3.6.1.- 3.1.3.100]
CP005080.1_4939	K00800	150.47	521.1	1.5e-156	3-phosphoshikimate 1-carboxyvinyltransferase [EC:2.5.1.19]
CP005080.1_4942	K07020	79.87	234.3	7.2e-70	uncharacterized protein
CP005080.1_4943	K03088	96.50	174.2	1.6e-51	RNA polymerase sigma-70 factor, ECF subfamily
CP005080.1_4948	K01462	69.73	221.5	6.8e-66	peptide deformylase [EC:3.5.1.88]
CP005080.1_4949	K13633	271.97	457.7	1.2e-137	AraC family transcriptional regulator, transcriptional activator FtrA
CP005080.1_4954	K00526	310.40	362.1	1.5e-108	ribonucleoside-diphosphate reductase beta chain [EC:1.17.4.1]
CP005080.1_4955	K00525	367.50	704.2	9.1e-212	ribonucleoside-diphosphate reductase alpha chain [EC:1.17.4.1]
CP005080.1_4957	K06200	289.50	776.8	6.3e-234	carbon starvation protein
CP005080.1_4958	K24969	292.47	446.5	7.2e-135	GntR family transcriptional regulator, nutrient-sensing system regulator
CP005080.1_4958	K24967	246.20	262.5	2.9e-78	GntR family transcriptional regulator, N-acetylglucosamine utilization regulator
CP005080.1_4958	K03710	178.10	247.7	9.6e-74	GntR family transcriptional regulator
CP005080.1_4959	K17329	313.30	447.7	1.8e-134	N,N'-diacetylchitobiose transport system substrate-binding protein
CP005080.1_4960	K17330	299.37	418.3	6.4e-126	N,N'-diacetylchitobiose transport system permease protein
CP005080.1_4961	K17331	321.87	378.4	8.6e-114	N,N'-diacetylchitobiose transport system permease protein
CP005080.1_4961	K02026	280.30	282.2	2.3e-84	multiple sugar transport system permease protein
CP005080.1_4962	K01207	251.63	462.1	1.3e-138	beta-N-acetylhexosaminidase [EC:3.2.1.52]
CP005080.1_4963	K02564	97.07	332.6	1.2e-99	glucosamine-6-phosphate deaminase [EC:3.5.99.6]
CP005080.1_4964	K00820	183.57	276.8	1.5e-82	glutamine---fructose-6-phosphate transaminase (isomerizing) [EC:2.6.1.16]
CP005080.1_4965	K00936	107.67	444.5	2.4e-133	two-component system, sensor histidine kinase PdtaS [EC:2.7.13.3]
CP005080.1_4966	K18955	70.43	115.6	9.5e-34	WhiB family transcriptional regulator, redox-sensing transcriptional regulator
CP005080.1_4967	K07029	147.20	170.1	2.6e-50	diacylglycerol kinase (ATP) [EC:2.7.1.107]
CP005080.1_4969	K03090	268.20	513.4	2.3e-154	RNA polymerase sigma-B factor
CP005080.1_4970	K04757	53.50	75.5	1.8e-21	serine/threonine-protein kinase RsbW [EC:2.7.11.1]
CP005080.1_4972	K24163	379.43	590.8	2e-177	monovalent cation/hydrogen antiporter
CP005080.1_4974	K05916	302.67	565.5	4.8e-170	nitric oxide dioxygenase [EC:1.14.12.17]
CP005080.1_4975	K13771	131.13	198.2	4.5e-59	Rrf2 family transcriptional regulator, nitric oxide-sensitive transcriptional repressor
CP005080.1_4977	K13787	299.27	393.3	7.3e-118	geranylgeranyl diphosphate synthase, type I [EC:2.5.1.1 2.5.1.10 2.5.1.29]
CP005080.1_4982	K01416	89.07	271.2	3.1e-81	snapalysin [EC:3.4.24.77]
CP005080.1_4987	K19267	112.87	347.6	5.1e-104	NAD(P)H dehydrogenase (quinone) [EC:1.6.5.2]
CP005080.1_4995	K00518	97.23	187.6	1.1e-55	nickel superoxide dismutase [EC:1.15.1.1]
CP005080.1_4999	K03088	96.50	103.8	4e-30	RNA polymerase sigma-70 factor, ECF subfamily
CP005080.1_5001	K02028	386.70	474.0	2.2e-142	polar amino acid transport system ATP-binding protein [EC:7.4.2.1]
CP005080.1_5002	K02029	214.53	319.6	1.4e-95	polar amino acid transport system permease protein
CP005080.1_5003	K02030	76.77	165.0	1.1e-48	polar amino acid transport system substrate-binding protein
CP005080.1_5004	K00027	507.30	601.2	1.4e-180	malate dehydrogenase (oxaloacetate-decarboxylating) [EC:1.1.1.38]
CP005080.1_5006	K05991	184.87	486.3	4.3e-146	endoglycosylceramidase [EC:3.2.1.123]
CP005080.1_5011	K11621	70.10	95.9	8.7e-28	lia operon protein LiaG
CP005080.1_5014	K01616	1319.00	2153.6	0	multifunctional 2-oxoglutarate metabolism enzyme [EC:2.2.1.5 4.1.1.71 1.2.4.2 2.3.1.61]

CP005080.1_5016	K02483	242.00	268.8	4e-80	two-component system, OmpR family, response regulator
CP005080.1_5019	K01338	863.10	933.2	6.4e-281	ATP-dependent Lon protease [EC:3.4.21.53]
CP005080.1_5020	K22278	73.27	249.5	2.6e-74	peptidoglycan-N-acetylglucosamine deacetylase [EC:3.5.1.104]
CP005080.1_5024	K07131	45.27	97.3	3.8e-28	uncharacterized protein
CP005080.1_5026	K06945	72.87	330.8	5.1e-99	uncharacterized protein
CP005080.1_5028	K21471	158.57	227.4	2.2e-67	peptidoglycan DL-endopeptidase CwlO [EC:3.4.-.-]
CP005080.1_5038	K05364	378.67	589.1	5.8e-177	penicillin-binding protein A
CP005080.1_5045	K01971	209.27	285.9	3.5e-85	bifunctional non-homologous end joining protein LigD [EC:6.5.1.1]
CP005080.1_5046	K10979	98.23	430.8	1.6e-129	DNA end-binding protein Ku
CP005080.1_5047	K12132	224.03	293.7	6.5e-88	eukaryotic-like serine/threonine-protein kinase [EC:2.7.11.1]
CP005080.1_5047	K11912	105.07	130.8	2.3e-38	serine/threonine-protein kinase PpkA [EC:2.7.11.1]
CP005080.1_5049	K22894	131.70	237.9	8.7e-71	SARP family transcriptional regulator, regulator of embCAB operon
CP005080.1_5059	K01887	107.30	194.8	1e-57	arginyl-tRNA synthetase [EC:6.1.1.19]
CP005080.1_5060	K01586	276.10	557.0	3e-167	diaminopimelate decarboxylase [EC:4.1.1.20]
CP005080.1_5061	K00003	285.10	590.2	2.5e-177	homoserine dehydrogenase [EC:1.1.1.3]
CP005080.1_5062	K01733	243.03	431.0	2.9e-129	threonine synthase [EC:4.2.3.1]
CP005080.1_5063	K00872	123.40	373.7	3.9e-112	homoserine kinase [EC:2.7.1.39]
CP005080.1_5064	K03628	186.43	760.0	4.7e-229	transcription termination factor Rho
CP005080.1_5065	K01005	193.97	217.9	1.3e-64	polyisoprenyl-teichoic acid--peptidoglycan teichoic acid transferase [EC:2.7.8.-]
CP005080.1_5066	K02909	21.83	121.6	1.1e-35	large subunit ribosomal protein L31
CP005080.1_5067	K02835	355.53	496.2	5.2e-149	peptide chain release factor 1
CP005080.1_5068	K02493	233.73	292.6	2.5e-87	release factor glutamine methyltransferase [EC:2.1.1.297]
CP005080.1_5069	K07566	93.50	243.1	2.3e-72	L-threonylcarbamoyladenylate synthase [EC:2.7.7.87]
CP005080.1_5073	K00600	75.20	552.1	7.3e-166	glycine hydroxymethyltransferase [EC:2.1.2.1]
CP005080.1_5074	K02851	237.03	404.8	1.8e-121	UDP-GlcNAc:undecaprenyl-phosphate/decaprenyl-phosphate GlcNAc-1-phosphate transferase [EC:2.7.8.33 2.7.8.35]
CP005080.1_5076	K02108	158.57	224.8	7.8e-67	F-type H+-transporting ATPase subunit a
CP005080.1_5077	K02110	68.60	74.8	2.9e-21	F-type H+-transporting ATPase subunit c
CP005080.1_5078	K02109	43.13	136.0	8.3e-40	F-type H+-transporting ATPase subunit b
CP005080.1_5079	K02113	45.90	141.4	1.7e-41	F-type H+-transporting ATPase subunit delta
CP005080.1_5080	K02111	432.10	862.1	1.6e-259	F-type H+/Na+-transporting ATPase subunit alpha [EC:7.1.2.2 7.2.2.1]
CP005080.1_5081	K02115	246.97	367.0	3.9e-110	F-type H+-transporting ATPase subunit gamma
CP005080.1_5082	K02112	465.83	841.0	2.5e-253	F-type H+/Na+-transporting ATPase subunit beta [EC:7.1.2.2 7.2.2.1]
CP005080.1_5083	K02114	99.10	130.4	2.8e-38	F-type H+-transporting ATPase subunit epsilon
CP005080.1_5085	K01183	115.40	249.8	2.4e-74	chitinase [EC:3.2.1.14]
CP005080.1_5089	K00798	35.70	223.8	9.4e-67	cob(I)alamin adenosyltransferase [EC:2.5.1.17]
CP005080.1_5090	K00074	404.50	418.2	2e-125	3-hydroxybutyryl-CoA dehydrogenase [EC:1.1.1.157]
CP005080.1_5091	K04749	63.93	77.2	6.8e-22	anti-sigma B factor antagonist
CP005080.1_5093	K07503	144.67	374.7	9.4e-113	endonuclease [EC:3.1.-.-]
CP005080.1_5098	K01990	262.37	350.6	5.4e-105	ABC-2 type transport system ATP-binding protein
CP005080.1_5100	K01990	262.37	325.9	1.7e-97	ABC-2 type transport system ATP-binding protein
CP005080.1_5103	K05606	80.33	172.4	3.4e-51	methylmalonyl-CoA/ethylmalonyl-CoA epimerase [EC:5.1.99.1]
CP005080.1_5104	K00626	488.13	513.6	2.9e-154	acetyl-CoA C-acetyltransferase [EC:2.3.1.9]
CP005080.1_5105	K07588	261.13	422.1	2.3e-126	GTPase [EC:3.6.5.-]
CP005080.1_5109	K02483	242.00	263.2	2e-78	two-component system, OmpR family, response regulator
CP005080.1_5124	K25820	974.80	1202.1	0	isobutyryl-CoA mutase large subunit [EC:5.4.99.13]
CP005080.1_5125	K07653	371.23	532.7	4.1e-160	two-component system, OmpR family, sensor histidine kinase MprB [EC:2.7.13.3]
CP005080.1_5125	K02484	312.80	322.1	3.4e-96	two-component system, OmpR family, sensor kinase [EC:2.7.13.3]
CP005080.1_5126	K07669	325.20	343.7	2.8e-103	two-component system, OmpR family, response regulator MprA
CP005080.1_5126	K02483	242.00	265.9	2.9e-79	two-component system, OmpR family, response regulator
CP005080.1_5127	K02563	193.67	393.8	5e-118	UDP-N-acetylglucosamine--N-acetylmuramyl-(pentapeptide) pyrophosphoryl-undecaprenol N-acetylglucosamine transferase [EC:2.4.1.227]
CP005080.1_5130	K05838	153.87	369.3	1e-110	putative thioredoxin
CP005080.1_5130	K03671	112.43	112.9	8.9e-33	thioredoxin
CP005080.1_5136	K00873	49.90	610.1	1.9e-183	pyruvate kinase [EC:2.7.1.40]
CP005080.1_5137	K00925	190.80	630.8	1.5e-189	acetate kinase [EC:2.7.2.1]
CP005080.1_5138	K13788	562.80	1041.1	1.3e-313	phosphate acetyltransferase [EC:2.3.1.8]
CP005080.1_5139	K21071	345.93	459.4	6.4e-138	ATP-dependent phosphofructokinase / diphosphate-dependent phosphofructokinase [EC:2.7.1.11 2.7.1.90]

CP005080.1_5142	K02476	523.20	738.6	2e-222	two-component system, CitB family, sensor kinase [EC:2.7.13.3]
CP005080.1_5143	K11103	521.30	652.6	2.3e-196	aerobic C4-dicarboxylate transport protein
CP005080.1_5145	K00700	301.20	1083.1	0	1,4-alpha-glucan branching enzyme [EC:2.4.1.18]
CP005080.1_5146	K16146	390.23	665.7	3.4e-200	maltokinase [EC:2.7.1.175]
CP005080.1_5147	K05343	524.60	1152.1	0	maltose alpha-D-glucosyltransferase / alpha-amylase [EC:5.4.99.16 3.2.1.1]
CP005080.1_5148	K16147	244.70	1107.2	0	starch synthase (maltosyl-transferring) [EC:2.4.99.16]
CP005080.1_5149	K00688	78.53	422.0	1.9e-126	glycogen phosphorylase [EC:2.4.1.1]
CP005080.1_5151	K20273	451.77	599.1	3.2e-180	zinc metalloprotease ZmpA
CP005080.1_5156	K01990	262.37	294.8	4.6e-88	ABC-2 type transport system ATP-binding protein
CP005080.1_5160	K01214	723.90	1111.3	0	isoamylase [EC:3.2.1.68]
CP005080.1_5160	K02438	1008.80	1026.1	7.1e-309	glycogen debranching enzyme [EC:3.2.1.196]
CP005080.1_5163	K01692	342.50	381.2	2.8e-114	enoyl-CoA hydratase [EC:4.2.1.17]
CP005080.1_5164	K04757	53.50	58.3	3.4e-16	serine/threonine-protein kinase RsbW [EC:2.7.11.1]
CP005080.1_5165	K22278	73.27	128.3	1.6e-37	peptidoglycan-N-acetylglucosamine deacetylase [EC:3.5.1.104]
CP005080.1_5168	K03929	467.47	513.2	4.6e-154	para-nitrobenzyl esterase [EC:3.1.1.-]
CP005080.1_5169	K01446	92.93	114.2	2.7e-33	peptidoglycan recognition protein
CP005080.1_5170	K18111	347.10	555.9	9.9e-168	(+)-beta-caryophyllene/(+)-caryolan-1-ol synthase [EC:4.2.3.89 4.2.1.138]
CP005080.1_5171	K13063	492.93	953.8	1.6e-287	2-amino-4-deoxychorismate synthase [EC:2.6.1.86]
CP005080.1_5172	K20261	314.27	341.3	6.2e-103	trans-2,3-dihydro-3-hydroxyanthranilic acid synthase [EC:3.3.2.15]
CP005080.1_5173	K00216	256.33	390.4	3.9e-117	2,3-dihydro-2,3-dihydroxybenzoate dehydrogenase [EC:1.3.1.28]
CP005080.1_5174	K01626	124.93	365.0	3.4e-109	3-deoxy-7-phosphoheptulonate synthase [EC:2.5.1.54]
CP005080.1_5176	K03527	70.27	448.2	1.7e-134	4-hydroxy-3-methylbut-2-en-1-yl diphosphate reductase [EC:1.17.7.4]
CP005080.1_5178	K01662	325.00	833.3	6.2e-251	1-deoxy-D-xylulose-5-phosphate synthase [EC:2.2.1.7]
CP005080.1_5179	K03526	92.70	575.3	5e-173	(E)-4-hydroxy-3-methylbut-2-enyl-diphosphate synthase [EC:1.17.7.1 1.17.7.3]
CP005080.1_5182	K16435	212.43	718.3	3.4e-216	dTDP-4-dehydro-6-deoxy-alpha-D-glucopyranose 2,3-dehydratase [EC:4.2.1.159]
CP005080.1_5184	K00973	234.80	250.5	1.4e-74	glucose-1-phosphate thymidylyltransferase [EC:2.7.7.24]
CP005080.1_5185	K22894	131.70	268.5	4.7e-80	SARP family transcriptional regulator, regulator of embCAB operon
CP005080.1_5186	K19784	108.27	118.5	1.7e-34	chromate reductase, NAD(P)H dehydrogenase (quinone)
CP005080.1_5187	K08167	383.90	664.2	7.8e-200	MFS transporter, DHA2 family, multidrug resistance protein
CP005080.1_5188	K16437	596.37	666.3	6.9e-201	methylation protein EvaC
CP005080.1_5191	K12711	381.13	394.7	1.7e-118	L-demethylnoviosyl transferase [EC:2.4.1.302]
CP005080.1_5192	K13316	293.63	306.3	3.1e-92	NDP-hexose 5-epimerase [EC:5.1.3.-]
CP005080.1_5193	K13327	343.67	429.2	2.6e-129	dTDP-3,4-didehydro-2,6-dideoxy-alpha-D-glucose 3-reductase [EC:1.1.1.384]
CP005080.1_5195	K25562	450.07	1008.2	9.2e-304	L-aspartate N-monooxygenase (nitrosuccinate-forming) [EC:1.14.13.248]
CP005080.1_5203	K06903	75.10	165.8	5.2e-49	Bacteriophage baseplate protein W
CP005080.1_5205	K06905	76.97	116.5	5.1e-34	Bacteriophage probable baseplate hub protein
CP005080.1_5205	K11904	72.10	108.9	8.3e-32	type VI secretion system secreted protein VgrG
CP005080.1_5212	K06907	144.57	566.2	1.8e-170	Bacteriophage tail sheath protein
CP005080.1_5219	K01029	368.27	395.6	3.3e-119	3-oxoacid CoA-transferase subunit B [EC:2.8.3.5]
CP005080.1_5220	K05972	74.77	207.2	1.8e-61	acetylxylan esterase [EC:3.1.1.72]
CP005080.1_5222	K00019	183.03	186.4	3.4e-55	3-hydroxybutyrate dehydrogenase [EC:1.1.1.30]
CP005080.1_5223	K08172	481.17	560.8	8.4e-169	MFS transporter, MHS family, shikimate and dehydroshikimate transport protein
CP005080.1_5223	K03762	459.97	479.5	6.6e-144	MFS transporter, MHS family, proline/betaine transporter
CP005080.1_5224	K09684	188.00	227.5	1.7e-67	PucR family transcriptional regulator, purine catabolism regulatory protein
CP005080.1_5225	K00865	57.57	491.7	9.8e-148	glycerate 2-kinase [EC:2.7.1.165]
CP005080.1_5227	K02483	242.00	262.2	3.9e-78	two-component system, OmpR family, response regulator
CP005080.1_5228	K02484	312.80	325.8	2.6e-97	two-component system, OmpR family, sensor kinase [EC:2.7.13.3]
CP005080.1_5230	K07284	60.53	61.1	3.9e-17	sortase A [EC:3.4.22.70]
CP005080.1_5234	K08970	48.13	225.9	2.5e-67	nickel/cobalt transporter (NicO) family protein
CP005080.1_5235	K01752	73.17	774.4	4.8e-233	L-serine dehydratase [EC:4.3.1.17]
CP005080.1_5236	K00600	75.20	669.6	1.9e-201	glycine hydroxymethyltransferase [EC:2.1.2.1]
CP005080.1_5237	K02437	35.70	170.9	1.1e-50	glycine cleavage system H protein
CP005080.1_5238	K00605	295.73	532.8	3.5e-160	glycine cleavage system T protein (aminomethyltransferase) [EC:2.1.2.10]
CP005080.1_5242	K02035	249.67	342.0	2.9e-102	peptide/nickel transport system substrate-binding protein
CP005080.1_5243	K02033	263.63	383.3	7e-115	peptide/nickel transport system permease protein
CP005080.1_5244	K02034	256.80	309.8	1.3e-92	peptide/nickel transport system permease protein

CP005080.1_5245	K02031	412.13	484.1	2.7e-145	peptide/nickel transport system ATP-binding protein
CP005080.1_5246	K02032	420.67	538.9	9.1e-162	peptide/nickel transport system ATP-binding protein
CP005080.1_5249	K22105	73.37	155.9	5.9e-46	TetR/AcrR family transcriptional regulator, fatty acid biosynthesis regulator
CP005080.1_5252	K04487	287.40	511.6	8.8e-154	cysteine desulfurase [EC:2.8.1.7]
CP005080.1_5254	K00566	424.50	476.5	3.2e-143	tRNA-uridine 2-sulfurtransferase [EC:2.8.1.13]
CP005080.1_5257	K22522	96.03	97.3	4.2e-28	cytokinin riboside 5'-monophosphate phosphoribohydrolase [EC:3.2.2.-]
CP005080.1_5260	K01972	119.00	957.8	1.8e-288	DNA ligase (NAD+) [EC:6.5.1.2]
CP005080.1_5262	K02435	35.93	87.8	2.5e-25	aspartyl-tRNA(Asn)/glutamyl-tRNA(Gln) amidotransferase subunit C [EC:6.3.5.6 6.3.5.7]
CP005080.1_5263	K02433	450.63	690.4	1.2e-207	aspartyl-tRNA(Asn)/glutamyl-tRNA(Gln) amidotransferase subunit A [EC:6.3.5.6 6.3.5.7]
CP005080.1_5263	K01426	303.90	409.9	1.1e-122	amidase [EC:3.5.1.4]
CP005080.1_5265	K02434	415.47	610.1	1.8e-183	aspartyl-tRNA(Asn)/glutamyl-tRNA(Gln) amidotransferase subunit B [EC:6.3.5.6 6.3.5.7]
CP005080.1_5266	K03893	199.80	471.7	1.2e-141	arsenical pump membrane protein
CP005080.1_5267	K01114	129.20	607.7	1.5e-182	phospholipase C [EC:3.1.4.3]
CP005080.1_5268	K06994	260.33	778.1	3.8e-234	putative drug exporter of the RND superfamily
CP005080.1_5269	K03817	131.60	256.6	1.4e-76	ribosomal-protein-serine acetyltransferase [EC:2.3.1.-]
CP005080.1_5272	K21430	239.17	305.9	2.2e-91	aldose sugar dehydrogenase [EC:1.1.5.-]
CP005080.1_5276	K01652	517.70	792.9	9.8e-239	acetolactate synthase I/II/III large subunit [EC:2.2.1.6]
CP005080.1_5277	K01653	49.70	242.4	1.4e-72	acetolactate synthase I/III small subunit [EC:2.2.1.6]
CP005080.1_5278	K00053	107.87	479.2	7.9e-144	ketol-acid reductoisomerase [EC:1.1.1.86]
CP005080.1_5279	K00058	329.93	491.6	1e-147	D-3-phosphoglycerate dehydrogenase / 2-oxoglutarate reductase [EC:1.1.1.95 1.1.1.399]
CP005080.1_5283	K12503	333.63	374.6	1.5e-112	short-chain Z-isoprenyl diphosphate synthase [EC:2.5.1.68]
CP005080.1_5285	K00318	179.80	188.9	6.2e-56	proline dehydrogenase [EC:1.5.5.2]
CP005080.1_5286	K00294	478.63	824.0	3.1e-248	1-pyrroline-5-carboxylate dehydrogenase [EC:1.2.1.88]
CP005080.1_5287	K17231	110.87	348.4	4.1e-104	iodotyrosine deiodinase [EC:1.21.1.1]
CP005080.1_5288	K18548	490.83	619.2	8.1e-187	glutamyl endopeptidase II [EC:3.4.21.82]
CP005080.1_5289	K17840	93.80	254.6	1.9e-76	aminoglycoside 2'-N-acetyltransferase I [EC:2.3.1.59]
CP005080.1_5293	K00052	432.33	464.4	2.6e-139	3-isopropylmalate dehydrogenase [EC:1.1.1.85]
CP005080.1_5294	K00826	167.23	425.6	8.8e-128	branched-chain amino acid aminotransferase [EC:2.6.1.42]
CP005080.1_5295	K10536	118.97	470.7	2.5e-141	agmatine deiminase [EC:3.5.3.12]
CP005080.1_5297	K01649	498.33	586.8	3.9e-176	2-isopropylmalate synthase [EC:2.3.3.13]
CP005080.1_5298	K02529	268.37	331.0	5.8e-99	LacI family transcriptional regulator, galactose operon repressor
CP005080.1_5299	K02035	249.67	294.3	7.8e-88	peptide/nickel transport system substrate-binding protein
CP005080.1_5300	K02032	420.67	450.8	4e-135	peptide/nickel transport system ATP-binding protein
CP005080.1_5301	K02033	263.63	348.4	2.7e-104	peptide/nickel transport system permease protein
CP005080.1_5302	K02034	256.80	289.8	1.4e-86	peptide/nickel transport system permease protein
CP005080.1_5303	K02031	412.13	425.4	1.5e-127	peptide/nickel transport system ATP-binding protein
CP005080.1_5304	K05350	554.93	632.3	5.4e-190	beta-glucosidase [EC:3.2.1.21]
CP005080.1_5305	K05349	305.00	630.9	1.7e-189	beta-glucosidase [EC:3.2.1.21]
CP005080.1_5310	K18939	163.23	164.4	1.1e-48	TetR/AcrR family transcriptional regulator, lmrAB and yxaGH operons repressor
CP005080.1_5310	K16137	104.80	113.8	5e-33	TetR/AcrR family transcriptional regulator, transcriptional repressor for nem operon
CP005080.1_5312	K13653	208.93	286.2	1.9e-85	AraC family transcriptional regulator
CP005080.1_5315	K04757	53.50	70.8	4.9e-20	serine/threonine-protein kinase RsbW [EC:2.7.11.1]
CP005080.1_5318	K01297	137.13	147.7	1.7e-43	muramoyltetrapeptide carboxypeptidase [EC:3.4.17.13]
CP005080.1_5325	K03781	68.70	764.4	5.2e-230	catalase [EC:1.11.1.6]
CP005080.1_5326	K07006	34.40	160.1	3.3e-47	uncharacterized protein
CP005080.1_5327	K23186	409.23	495.2	1.1e-148	iron-siderophore transport system permease protein
CP005080.1_5328	K23187	358.37	432.1	1.4e-129	iron-siderophore transport system permease protein
CP005080.1_5329	K23188	469.87	525.8	7.2e-158	iron-siderophore transport system ATP-binding protein [EC:7.2.2.17 7.2.2.-]
CP005080.1_5329	K02013	238.30	354.0	5.1e-106	iron complex transport system ATP-binding protein [EC:7.2.2.-]
CP005080.1_5330	K25286	277.13	321.3	5.2e-96	iron-siderophore transport system substrate-binding protein
CP005080.1_5336	K17624	790.20	1554.5	0	endo-alpha-N-acetylgalactosaminidase [EC:3.2.1.97]
CP005080.1_5338	K27095	37.00	44.7	6.4e-12	acyl-CoA carboxylase epsilon subunit
CP005080.1_5339	K06945	72.87	334.6	3.5e-100	uncharacterized protein
CP005080.1_5341	K07131	45.27	141.7	9.7e-42	uncharacterized protein
CP005080.1_5343	K06945	72.87	331.9	2.3e-99	uncharacterized protein
CP005080.1_5345	K07131	45.27	138.8	7.5e-41	uncharacterized protein

CP005080.1_5349	K01885	298.30	615.1	1.1e-184	glutamyl-tRNA synthetase [EC:6.1.1.17]
CP005080.1_5350	K07025	112.47	133.2	4.8e-39	putative hydrolase of the HAD superfamily
CP005080.1_5352	K01703	454.33	666.5	1.1e-200	3-isopropylmalate/(R)-2-methylmalate dehydratase large subunit [EC:4.2.1.33 4.2.1.35]
CP005080.1_5353	K01704	131.03	269.3	1.7e-80	3-isopropylmalate/(R)-2-methylmalate dehydratase small subunit [EC:4.2.1.33 4.2.1.35]
CP005080.1_5355	K03530	115.33	138.2	1.7e-40	DNA-binding protein HU-beta
CP005080.1_5357	K14941	82.90	244.9	3.6e-73	2-phospho-L-lactate/phosphoenolpyruvate guanylyltransferase [EC:2.7.7.68 2.7.7.105]
CP005080.1_5358	K00655	122.40	185.8	5.3e-55	1-acyl-sn-glycerol-3-phosphate acyltransferase [EC:2.3.1.51]
CP005080.1_5359	K00057	279.33	541.9	7.4e-163	glycerol-3-phosphate dehydrogenase (NAD(P)+) [EC:1.1.1.94]
CP005080.1_5360	K01921	226.63	438.3	2.1e-131	D-alanine-D-alanine ligase [EC:6.3.2.4]
CP005080.1_5362	K00946	141.30	406.9	6.6e-122	thiamine-monophosphate kinase [EC:2.7.4.16]
CP005080.1_5363	K00941	324.10	394.8	3.2e-118	hydroxymethylpyrimidine/phosphomethylpyrimidine kinase [EC:2.7.1.49 2.7.4.7]
CP005080.1_5364	K02902	22.37	66.0	1.8e-18	large subunit ribosomal protein L28
CP005080.1_5365	K07030	292.60	714.8	5.9e-215	fatty acid kinase [EC:2.7.2.18]
CP005080.1_5366	K03655	393.80	870.4	1.4e-261	ATP-dependent DNA helicase RecG [EC:5.6.2.4]
CP005080.1_5367	K08316	94.87	217.7	7.3e-65	16S rRNA (guanine966-N2)-methyltransferase [EC:2.1.1.171]
CP005080.1_5368	K00954	81.30	260.3	7e-78	pantetheine-phosphate adenylyltransferase [EC:2.7.7.3]
CP005080.1_5370	K07040	18.17	166.7	3.6e-49	DUF177 domain-containing protein
CP005080.1_5371	K02911	22.10	73.1	1.1e-20	large subunit ribosomal protein L32
CP005080.1_5372	K03685	155.00	286.2	2.2e-85	ribonuclease III [EC:3.1.26.3]
CP005080.1_5373	K10563	196.13	406.8	4.9e-122	formamidopyrimidine-DNA glycosylase [EC:3.2.2.23 4.2.99.18]
CP005080.1_5379	K03529	574.17	1422.6	0	chromosome segregation protein
CP005080.1_5380	K08139	431.47	452.7	1e-135	MFS transporter, SP family, sugar:H+ symporter
CP005080.1_5382	K03110	289.73	482.0	7.1e-145	fused signal recognition particle receptor
CP005080.1_5385	K03320	145.33	502.0	1.2e-150	ammonium transporter, Amt family
CP005080.1_5386	K04751	115.67	186.2	3.5e-55	nitrogen regulatory protein P-II 1
CP005080.1_5387	K00990	158.67	933.7	4.9e-281	[protein-PII] uridylyltransferase [EC:2.7.7.59]
CP005080.1_5388	K03106	330.30	669.4	2.2e-201	signal recognition particle subunit SRP54 [EC:3.6.5.4]
CP005080.1_5391	K02959	27.40	149.2	5.4e-44	small subunit ribosomal protein S16
CP005080.1_5392	K06960	46.00	96.8	5.2e-28	uncharacterized protein
CP005080.1_5393	K02860	39.17	231.4	6e-69	16S rRNA processing protein RimM
CP005080.1_5394	K00554	108.40	374.0	3e-112	tRNA (guanine37-N1)-methyltransferase [EC:2.1.1.228]
CP005080.1_5395	K02884	25.43	172.7	3.5e-51	large subunit ribosomal protein L19
CP005080.1_5396	K03100	114.77	226.8	1.5e-67	signal peptidase I [EC:3.4.21.89]
CP005080.1_5397	K03100	114.77	254.4	5.9e-76	signal peptidase I [EC:3.4.21.89]
CP005080.1_5398	K03100	114.77	234.5	6.9e-70	signal peptidase I [EC:3.4.21.89]
CP005080.1_5399	K03100	114.77	170.4	2.2e-50	signal peptidase I [EC:3.4.21.89]
CP005080.1_5402	K07460	34.73	165.3	4.6e-49	putative endonuclease
CP005080.1_5403	K07391	246.20	481.9	1.5e-144	magnesium chelatase family protein
CP005080.1_5405	K04757	53.50	64.2	5.4e-18	serine/threonine-protein kinase RsbW [EC:2.7.11.1]
CP005080.1_5410	K01446	92.93	102.7	8.3e-30	peptidoglycan recognition protein
CP005080.1_5427	K06909	42.80	121.4	2.2e-35	phage terminase large subunit
CP005080.1_5439	K03111	28.83	134.2	3.1e-39	single-strand DNA-binding protein
CP005080.1_5455	K06400	148.90	205.8	5.2e-61	site-specific DNA recombinase
CP005080.1_5456	K07391	246.20	261.1	8.6e-78	magnesium chelatase family protein
CP005080.1_5457	K04096	28.87	312.3	3.1e-93	DNA processing protein
CP005080.1_5466	K06919	27.00	60.5	2.9e-17	putative DNA primase/helicase
CP005080.1_5476	K06400	148.90	218.0	1.1e-64	site-specific DNA recombinase
CP005080.1_5476	K04096	28.87	59.5	1.5e-16	DNA processing protein
CP005080.1_5477	K02405	185.07	402.8	9.8e-121	RNA polymerase sigma factor FliA
CP005080.1_5480	K02967	165.67	443.1	6.5e-133	small subunit ribosomal protein S2
CP005080.1_5481	K02357	112.87	298.5	4.8e-89	elongation factor Ts
CP005080.1_5482	K09903	92.77	415.7	7.4e-125	uridylate kinase [EC:2.7.4.22]
CP005080.1_5483	K02838	25.83	221.1	7.7e-66	ribosome recycling factor
CP005080.1_5484	K00981	43.57	248.2	7e-74	phosphatidate cytidylyltransferase [EC:2.7.7.41]
CP005080.1_5485	K06941	65.87	501.8	1e-150	23S rRNA (adenine2503-C2)-methyltransferase [EC:2.1.1.192]
CP005080.1_5486	K02064	183.73	435.8	5.4e-131	thiamine transport system substrate-binding protein

CP005080.1_5487	K02063	333.90	667.1	7.1e-201	thiamine transport system permease protein
CP005080.1_5488	K02062	405.70	441.5	1.6e-132	thiamine transport system ATP-binding protein [EC:7.6.2.15]
CP005080.1_5493	K03718	149.50	249.1	1.4e-74	Lrp/AsnC family transcriptional regulator, regulator for asnA, asnC and gidA
CP005080.1_5494	K00130	687.90	688.2	3.8e-207	betaine-aldehyde dehydrogenase [EC:1.2.1.8]
CP005080.1_5498	K01126	152.53	154.0	1.8e-45	glycerophosphoryl diester phosphodiesterase [EC:3.1.4.46]
CP005080.1_5499	K18286	372.23	553.0	8.3e-167	aminodeoxyfutalosine deaminase [EC:3.5.4.40]
CP005080.1_5502	K00130	687.90	695.1	3e-209	betaine-aldehyde dehydrogenase [EC:1.2.1.8]
CP005080.1_5504	K11072	493.17	523.2	3e-157	spermidine/putrescine transport system ATP-binding protein [EC:7.6.2.11]
CP005080.1_5505	K11071	289.37	358.5	1.3e-107	spermidine/putrescine transport system permease protein
CP005080.1_5506	K11070	283.70	315.3	2.7e-94	spermidine/putrescine transport system permease protein
CP005080.1_5512	K07250	641.20	694.5	2.4e-209	4-aminobutyrate aminotransferase / (S)-3-amino-2-methylpropionate transaminase / 5-aminovalerate transaminase [EC:2.6.1.19 2.6.1.22 2.6.1.48]
CP005080.1_5514	K09684	188.00	329.0	2.9e-98	PucR family transcriptional regulator, purine catabolism regulatory protein
CP005080.1_5515	K16137	104.80	149.5	6.7e-44	TetR/AcrR family transcriptional regulator, transcriptional repressor for nem operon
CP005080.1_5519	K00099	227.13	608.2	7.3e-183	1-deoxy-D-xylulose-5-phosphate reductoisomerase [EC:1.1.1.267]
CP005080.1_5520	K11749	79.03	270.0	2.2e-80	regulator of sigma E protease [EC:3.4.24.-]
CP005080.1_5521	K03526	92.70	574.7	8e-173	(E)-4-hydroxy-3-methylbut-2-enyl-diphosphate synthase [EC:1.17.7.1 1.17.7.3]
CP005080.1_5522	K06976	72.13	357.9	3e-107	uncharacterized protein
CP005080.1_5524	K01881	163.60	480.8	2.7e-144	prolyl-tRNA synthetase [EC:6.1.1.15]
CP005080.1_5525	K04343	77.20	273.3	1.7e-81	streptomycin 6-kinase [EC:2.7.1.72]
CP005080.1_5528	K09748	22.77	168.9	5.7e-50	ribosome maturation factor RimP
CP005080.1_5529	K02600	35.63	475.6	7.8e-143	transcription termination/antitermination protein NusA
CP005080.1_5530	K07742	20.17	95.8	1.1e-27	uncharacterized protein
CP005080.1_5532	K02519	314.93	893.5	5.6e-269	translation initiation factor IF-2
CP005080.1_5533	K09764	37.30	135.6	8.2e-40	uncharacterized protein
CP005080.1_5534	K02834	26.40	167.8	1e-49	ribosome-binding factor A
CP005080.1_5535	K03177	194.47	347.1	6.9e-104	tRNA pseudouridine55 synthase [EC:5.4.99.25]
CP005080.1_5537	K11753	132.60	398.6	8.7e-120	riboflavin kinase / FMN adenylyltransferase [EC:2.7.1.26 2.7.7.2]
CP005080.1_5538	K07402	68.07	396.1	8.5e-119	xanthine dehydrogenase accessory factor
CP005080.1_5540	K02032	420.67	436.9	6.4e-131	peptide/nickel transport system ATP-binding protein
CP005080.1_5541	K02031	412.13	445.6	1.2e-133	peptide/nickel transport system ATP-binding protein
CP005080.1_5542	K02034	256.80	322.8	1.4e-96	peptide/nickel transport system permease protein
CP005080.1_5543	K02033	263.63	380.2	6.1e-114	peptide/nickel transport system permease protein
CP005080.1_5544	K02035	249.67	354.3	5.4e-106	peptide/nickel transport system substrate-binding protein
CP005080.1_5547	K27088	53.20	170.5	2.7e-50	ESX secretion system protein EccE
CP005080.1_5548	K27088	53.20	263.3	2.2e-78	ESX secretion system protein EccE
CP005080.1_5549	K27085	156.57	438.1	3.1e-131	ESX secretion system ATPase EccB
CP005080.1_5550	K14743	282.67	397.8	4.1e-119	membrane-anchored mycosin MYCP [EC:3.4.21.-]
CP005080.1_5554	K14956	27.87	27.9	1e-06	ESAT-6 family protein
CP005080.1_5555	K14956	27.87	61.5	5.4e-17	ESAT-6 family protein
CP005080.1_5556	K27086	939.33	1851.1	0	ESX secretion system protein EccC
CP005080.1_5557	K27087	55.33	330.5	8e-99	ESX secretion system protein EccD
CP005080.1_5558	K02956	27.43	123.8	4.6e-36	small subunit ribosomal protein S15
CP005080.1_5559	K00962	326.70	955.8	7.3e-288	polyribonucleotide nucleotidyltransferase [EC:2.7.7.8]
CP005080.1_5560	K07263	220.23	249.9	2.6e-74	zinc protease [EC:3.4.24.-]
CP005080.1_5561	K00215	135.50	292.0	2.7e-87	4-hydroxy-tetrahydrodipicolinate reductase [EC:1.17.1.8]
CP005080.1_5565	K03465	98.30	196.9	1.8e-58	thymidylate synthase (FAD) [EC:2.1.1.148]
CP005080.1_5566	K01714	259.73	358.7	2.1e-107	4-hydroxy-tetrahydrodipicolinate synthase [EC:4.3.3.7]
CP005080.1_5567	K12574	123.37	766.5	8.6e-231	ribonuclease J [EC:3.1.-.-]
CP005080.1_5568	K07315	145.67	230.8	1.3e-68	phosphoserine phosphatase RsbU/P [EC:3.1.3.3]
CP005080.1_5569	K13598	298.43	308.9	3e-92	two-component system, NtrC family, nitrogen regulation sensor histidine kinase NtrY [EC:2.7.13.3]
CP005080.1_5571	K03466	191.80	753.9	1.5e-226	DNA segregation ATPase FtsK/SpoIIIE, S-DNA-T family
CP005080.1_5572	K15539	70.70	227.4	1.6e-67	cytoskeleton protein RodZ
CP005080.1_5573	K14441	412.60	632.1	3.2e-190	ribosomal protein S12 methylthiotransferase [EC:2.8.4.4]
CP005080.1_5574	K00995	120.80	182.4	6.2e-54	CDP-diacylglycerol---glycerol-3-phosphate 3-phosphatidyltransferase [EC:2.7.8.5]
CP005080.1_5575	K03743	24.90	218.0	6.1e-65	nicotinamide-nucleotide amidase [EC:3.5.1.42]
CP005080.1_5576	K27043	96.73	164.9	1.2e-48	XRE family transcriptional regulator, stress-response regulator

CP005080.1_5577	K04047	77.30	162.9	5e-48	starvation-inducible DNA-binding protein
CP005080.1_5580	K05522	197.43	376.2	5.9e-113	endonuclease VIII [EC:3.2.2.- 4.2.99.18]
CP005080.1_5581	K03724	278.40	1909.5	0	ATP-dependent helicase Lhr and Lhr-like helicase [EC:5.6.2.6 5.6.2.4]
CP005080.1_5583	K26605	54.60	244.9	3.8e-73	branched chain amino acid efflux pump
CP005080.1_5584	K26606	27.30	62.9	1e-17	branched chain amino acid efflux pump
CP005080.1_5587	K20469	203.00	229.5	2.8e-68	putative heme transporter
CP005080.1_5591	K03553	190.37	729.2	2.4e-219	recombination protein RecA
CP005080.1_5592	K03565	36.33	222.3	3.6e-66	regulatory protein
CP005080.1_5596	K05712	356.13	583.0	2.9e-175	3-(3-hydroxy-phenyl)propionate hydroxylase [EC:1.14.13.127]
CP005080.1_5597	K10007	269.30	395.1	2.2e-118	glutamate transport system permease protein
CP005080.1_5598	K10006	244.67	299.8	8.3e-90	glutamate transport system permease protein
CP005080.1_5599	K10005	314.87	371.7	2.2e-111	glutamate transport system substrate-binding protein
CP005080.1_5599	K02030	76.77	133.0	6.1e-39	polar amino acid transport system substrate-binding protein
CP005080.1_5600	K10008	442.50	527.8	6.9e-159	glutamate transport system ATP-binding protein [EC:7.4.2.1]
CP005080.1_5600	K02028	386.70	452.7	6.4e-136	polar amino acid transport system ATP-binding protein [EC:7.4.2.1]
CP005080.1_5601	K02483	242.00	258.2	6.4e-77	two-component system, OmpR family, response regulator
CP005080.1_5603	K07080	93.20	269.0	3.1e-80	uncharacterized protein
CP005080.1_5604	K06168	451.40	770.8	3e-232	tRNA-2-methylthio-N6-dimethylallyladenosine synthase [EC:2.8.4.3]
CP005080.1_5607	K00791	183.90	362.1	2.2e-108	tRNA dimethylallyltransferase [EC:2.5.1.75]
CP005080.1_5609	K01778	61.00	316.8	7.3e-95	diaminopimelate epimerase [EC:5.1.1.7]
CP005080.1_5610	K01139	801.03	876.1	8.4e-264	GTP diphosphokinase / guanosine-3',5'-bis(diphosphate) 3'-diphosphatase [EC:2.7.6.5 3.1.7.2]
CP005080.1_5612	K03665	194.73	608.7	4.4e-183	GTPase
CP005080.1_5615	K00836	439.63	765.5	1.5e-230	diaminobutyrate-2-oxoglutarate transaminase [EC:2.6.1.76]
CP005080.1_5616	K24108	477.87	496.7	3.6e-149	spermidine-citrate ligase [EC:6.3.2.-]
CP005080.1_5619	K03722	230.90	724.6	6.2e-218	ATP-dependent DNA helicase DinG [EC:5.6.2.3]
CP005080.1_5620	K01356	128.77	325.1	1.9e-97	repressor LexA [EC:3.4.21.88]
CP005080.1_5621	K07738	41.80	281.2	2.2e-84	transcriptional repressor NrdR
CP005080.1_5622	K00525	367.50	713.9	1.1e-214	ribonucleoside-diphosphate reductase alpha chain [EC:1.17.4.1]
CP005080.1_5623	K05979	58.63	161.9	7.9e-48	2-phosphosulfolactate phosphatase [EC:3.1.3.71]
CP005080.1_5629	K03470	126.33	243.7	1e-72	ribonuclease HII [EC:3.1.26.4]
CP005080.1_5637	K03086	466.80	590.5	1.6e-177	RNA polymerase primary sigma factor
CP005080.1_5637	K03093	118.63	132.2	1.1e-38	RNA polymerase sigma-I factor
CP005080.1_5642	K14393	287.90	647.0	1.2e-194	cation/acetate symporter
CP005080.1_5644	K02476	523.20	730.8	4.6e-220	two-component system, CitB family, sensor kinase [EC:2.7.13.3]
CP005080.1_5645	K02529	268.37	304.4	6.7e-91	LacI family transcriptional regulator, galactose operon repressor
CP005080.1_5650	K07263	220.23	365.2	3.6e-109	zinc protease [EC:3.4.24.-]
CP005080.1_5654	K02784	81.30	84.1	4.7e-24	phosphocarrier protein HPr
CP005080.1_5658	K00857	82.33	147.9	1.8e-43	thymidine kinase [EC:2.7.1.21]
CP005080.1_5659	K06996	53.13	122.2	1.6e-35	uncharacterized protein
CP005080.1_5660	K01011	189.10	304.0	5.5e-91	thiosulfate/3-mercaptopyruvate sulfurtransferase [EC:2.8.1.1 2.8.1.2]
CP005080.1_5663	K05888	468.30	692.3	1.7e-208	L-gulono-1,4-lactone dehydrogenase [EC:1.1.2.-]
CP005080.1_5665	K01772	59.27	416.9	4.9e-125	protoporphyrin/coproporphyrin ferrochelatase [EC:4.98.1.1 4.99.1.9]
CP005080.1_5666	K01092	211.57	291.7	3.7e-87	myo-inositol-1(or 4)-monophosphatase [EC:3.1.3.25]
CP005080.1_5668	K18352	318.00	325.9	3.7e-98	two-component system, OmpR family, response regulator VanR
CP005080.1_5669	K18351	281.33	376.6	4.3e-113	two-component system, OmpR family, sensor histidine kinase VanS [EC:2.7.13.3]
CP005080.1_5674	K01520	47.27	164.9	9.7e-49	dUTP diphosphatase [EC:3.6.1.23]
CP005080.1_5676	K01545	22.23	35.3	3.8e-09	potassium-transporting ATPase KdpF subunit
CP005080.1_5677	K01546	126.87	867.1	3.1e-261	potassium-transporting ATPase potassium-binding subunit
CP005080.1_5678	K01547	363.10	1218.4	0	potassium-transporting ATPase ATP-binding subunit [EC:7.2.2.6]
CP005080.1_5679	K01548	33.50	240.2	8.2e-72	potassium-transporting ATPase KdpC subunit
CP005080.1_5680	K07646	228.07	1058.3	9.9e-319	two-component system, OmpR family, sensor histidine kinase KdpD [EC:2.7.13.3]
CP005080.1_5681	K07667	282.27	404.8	1.9e-121	two-component system, OmpR family, KDP operon response regulator KdpE
CP005080.1_5684	K03499	127.40	211.0	9.4e-63	trk/ktr system potassium uptake protein
CP005080.1_5685	K03499	127.40	193.1	2.7e-57	trk/ktr system potassium uptake protein
CP005080.1_5687	K03215	301.40	343.4	1.2e-102	23S rRNA (uracil1939-C5)-methyltransferase [EC:2.1.1.190]
CP005080.1_5695	K27097	58.70	181.6	5.2e-54	ESX-1-secreted protein regulator
CP005080.1_5700	K07116	433.37	810.4	8.9e-244	acyl-homoserine-lactone acylase [EC:3.5.1.97]
CP005080.1_5704	K22894	131.70	321.3	4.3e-96	SARP family transcriptional regulator, regulator of embCAB operon
CP005080.1_5713	K06413	333.77	736.2	4e-222	stage V sporulation protein K
CP005080.1_5713	K27084	413.47	572.0	5.7e-172	ESX secretion system protein EccA
CP005080.1_5715	K27086	939.33	1768.4	0	ESX secretion system protein EccC
CP005080.1_5716	K27087	55.33	283.0	1.9e-84	ESX secretion system protein EccD
CP005080.1_5717	K27085	156.57	420.6	6.3e-126	ESX secretion system ATPase EccB
CP005080.1_5719	K14956	27.87	47.8	8.3e-13	ESAT-6 family protein

CP005080.1_5730	K00612	190.27	862.0	2.4e-259	carbamoyltransferase [EC:2.1.3.-]
CP005080.1_5732	K18058	76.40	231.0	7.1e-69	L-asparagine oxygenase [EC:1.14.11.39]
CP005080.1_5736	K01956	530.93	686.2	3.4e-206	carbamoyl-phosphate synthase small subunit [EC:6.3.5.5]
CP005080.1_5737	K01955	1479.70	1724.7	0	carbamoyl-phosphate synthase large subunit [EC:6.3.5.5]
CP005080.1_5746	K01387	190.50	888.8	2.4e-267	microbial collagenase [EC:3.4.24.3]
CP005080.1_5747	K21061	319.30	584.0	1.2e-175	D-hydroxyproline dehydrogenase subunit beta [EC:1.5.99.-]
CP005080.1_5748	K22550	93.90	95.4	7.1e-28	D-hydroxyproline dehydrogenase subunit gamma
CP005080.1_5749	K22549	408.90	538.4	8.8e-162	D-hydroxyproline dehydrogenase subunit alpha [EC:1.5.99.-]
CP005080.1_5750	K21062	296.60	425.2	7.7e-128	1-pyrroline-4-hydroxy-2-carboxylate deaminase [EC:3.5.4.22]
CP005080.1_5751	K01777	440.43	512.3	2.5e-154	proline racemase [EC:5.1.1.4]
CP005080.1_5753	K09684	188.00	207.8	1.5e-61	PucR family transcriptional regulator, purine catabolism regulatory protein
CP005080.1_5755	K12528	927.53	1525.6	0	putative selenate reductase molybdopterin-binding subunit
CP005080.1_5760	K00573	87.43	248.9	3.1e-74	protein-L-isoaspartate(D-aspartate) O-methyltransferase [EC:2.1.1.77]
CP005080.1_5761	K20484	130.10	311.8	3.9e-93	class I lanthipeptide synthase [EC:3.13.2.4]
CP005080.1_5763	K20483	108.60	853.6	9e-257	class I lanthipeptide synthase [EC:3.13.2.4]
CP005080.1_5769	K25286	277.13	311.6	4.8e-93	iron-siderophore transport system substrate-binding protein
CP005080.1_5770	K23186	409.23	431.2	2.5e-129	iron-siderophore transport system permease protein
CP005080.1_5774	K14699	597.47	720.4	1.2e-216	ATP-binding cassette, subfamily B, bacterial IrtB/YbtQ [EC:7.-.-.-]
CP005080.1_5775	K14698	624.83	840.5	5.4e-253	ATP-binding cassette, subfamily B, bacterial IrtA/YbtP [EC:7.-.-.-]
CP005080.1_5777	K03975	59.97	106.9	4e-31	membrane-associated protein
CP005080.1_5779	K02081	291.97	338.3	1.2e-101	DeoR family transcriptional regulator, aga operon transcriptional repressor
CP005080.1_5780	K09772	38.13	90.7	4.2e-26	cell division inhibitor SepF
CP005080.1_5781	K16904	106.87	109.6	6.9e-32	dCTP diphosphatase [EC:3.6.1.12]
CP005080.1_5784	K00547	285.53	401.3	2.2e-120	homocysteine S-methyltransferase [EC:2.1.1.10]
CP005080.1_5785	K20798	86.27	217.1	2.1e-64	small RNA 2'-O-methyltransferase [EC:2.1.1.386]
CP005080.1_5787	K01478	115.00	576.7	1.2e-173	arginine deiminase [EC:3.5.3.6]
CP005080.1_5788	K00611	353.27	455.3	7.9e-137	ornithine carbamoyltransferase [EC:2.1.3.3]
CP005080.1_5789	K03294	476.00	518.2	1.1e-155	basic amino acid/polyamine antiporter, APA family
CP005080.1_5790	K04757	53.50	72.5	1.6e-20	serine/threonine-protein kinase RsbW [EC:2.7.11.1]
CP005080.1_5791	K15866	288.97	296.4	1.7e-88	2-(1,2-epoxy-1,2-dihydrophenyl)acetyl-CoA isomerase [EC:5.3.3.18]
CP005080.1_5792	K09461	658.73	1331.6	0	anthraniloyl-CoA monooxygenase [EC:1.14.13.40]
CP005080.1_5794	K02616	72.93	308.3	3.1e-92	phenylacetic acid degradation operon negative regulatory protein
CP005080.1_5795	K08295	533.40	962.8	4.3e-290	2-aminobenzoate-CoA ligase [EC:6.2.1.32]
CP005080.1_5796	K00249	382.07	409.3	1.2e-122	acyl-CoA dehydrogenase [EC:1.3.8.7]
CP005080.1_5799	K05565	689.50	1142.0	0	multicomponent Na+:H+ antiporter subunit A
CP005080.1_5800	K05567	122.63	173.6	3.3e-51	multicomponent Na+:H+ antiporter subunit C
CP005080.1_5801	K05568	402.87	578.8	5.3e-174	multicomponent Na+:H+ antiporter subunit D
CP005080.1_5802	K05569	112.73	130.0	4.3e-38	multicomponent Na+:H+ antiporter subunit E
CP005080.1_5803	K05570	77.63	93.8	3.6e-27	multicomponent Na+:H+ antiporter subunit F
CP005080.1_5804	K05571	107.83	145.5	4.9e-43	multicomponent Na+:H+ antiporter subunit G
CP005080.1_5806	K00980	122.07	185.7	2.5e-55	glycerol-3-phosphate cytidylyltransferase [EC:2.7.7.39]
CP005080.1_5807	K03208	189.13	473.6	4e-142	putative colanic acid biosynthesis glycosyltransferase WcaI
CP005080.1_5816	K01711	282.83	636.6	2.7e-191	GDPmannose 4,6-dehydratase [EC:4.2.1.47]
CP005080.1_5817	K02377	232.90	481.0	1.1e-144	GDP-L-fucose synthase [EC:1.1.1.271]
CP005080.1_5818	K16566	223.20	242.1	1.4e-72	exopolysaccharide production protein ExoY
CP005080.1_5821	K07315	145.67	196.9	2.3e-58	phosphoserine phosphatase RsbU/P [EC:3.1.3.3]
CP005080.1_5821	K04757	53.50	59.4	1.6e-16	serine/threonine-protein kinase RsbW [EC:2.7.11.1]
CP005080.1_5824	K17755	681.23	988.8	2.2e-298	choline oxidase [EC:1.1.3.17]
CP005080.1_5826	K00130	687.90	728.9	1.8e-219	betaine-aldehyde dehydrogenase [EC:1.2.1.8]
CP005080.1_5830	K22894	131.70	262.3	3.4e-78	SARP family transcriptional regulator, regulator of embCAB operon
CP005080.1_5835	K00761	217.77	235.6	3.4e-70	uracil phosphoribosyltransferase [EC:2.4.2.9]
CP005080.1_5836	K03809	60.03	183.7	1.9e-54	NAD(P)H dehydrogenase (quinone) [EC:1.6.5.2]
CP005080.1_5837	K27871	1128.67	1936.8	0	indigoidine synthase [EC:4.3.3.9]
CP005080.1_5838	K16329	279.93	412.3	6.6e-124	pseudouridylate synthase [EC:4.2.1.70]
CP005080.1_5840	K00789	19.03	612.9	2.3e-184	S-adenosylmethionine synthetase [EC:2.5.1.6]
CP005080.1_5842	K00297	201.37	345.1	2.9e-103	methylenetetrahydrofolate reductase (NADH) [EC:1.5.1.54]
CP005080.1_5845	K15269	141.60	416.6	3e-125	probable blue pigment (indigoidine) exporter
CP005080.1_5854	K07497	13.93	166.7	4e-49	putative transposase
CP005080.1_5858	K19147	46.97	227.9	9.4e-68	5-methylcytosine-specific restriction enzyme subunit McrC
CP005080.1_5859	K07452	78.20	335.2	2.4e-100	5-methylcytosine-specific restriction enzyme B [EC:3.1.21.-]
CP005080.1_5862	K04757	53.50	56.5	1.1e-15	serine/threonine-protein kinase RsbW [EC:2.7.11.1]
CP005080.1_5864	K00790	172.73	447.0	2.9e-134	UDP-N-acetylglucosamine 1-carboxyvinyltransferase [EC:2.5.1.7]
CP005080.1_5869	K27802	1411.80	1536.2	0	aconitate hydratase A / 2-methylisocitrate dehydratase [EC:4.2.1.3 4.2.1.99]
CP005080.1_5873	K10200	256.27	585.9	2.2e-176	N-acetylglucosamine transport system substrate-binding protein
CP005080.1_5874	K10201	325.23	419.3	2.8e-126	N-acetylglucosamine transport system permease protein
CP005080.1_5875	K10202	327.93	460.2	8.6e-139	N-acetylglucosamine transport system permease protein
CP005080.1_5875	K10119	296.93	330.1	5.7e-99	raffinose/stachyose/melibiose transport system permease protein

CP005080.1_5875	K02026	280.30	283.1	1.2e-84	multiple sugar transport system permease protein
CP005080.1_5876	K25026	274.23	284.2	7.3e-85	glucokinase [EC:2.7.1.2]
CP005080.1_5877	K10543	351.43	485.2	6.6e-146	D-xylose transport system substrate-binding protein
CP005080.1_5878	K10545	348.53	461.7	1.4e-138	D-xylose transport system ATP-binding protein [EC:7.5.2.10]
CP005080.1_5879	K10544	416.67	505.9	3.4e-152	D-xylose transport system permease protein
CP005080.1_5880	K01662	325.00	922.4	7.3e-278	1-deoxy-D-xylulose-5-phosphate synthase [EC:2.2.1.7]
CP005080.1_5881	K03294	476.00	617.7	8.5e-186	basic amino acid/polyamine antiporter, APA family
CP005080.1_5882	K07497	13.93	84.0	5.1e-24	putative transposase
CP005080.1_5884	K01005	193.97	223.2	3.2e-66	polyisoprenyl-teichoic acid--peptidoglycan teichoic acid transferase [EC:2.7.8.-]
CP005080.1_5888	K00712	180.83	227.8	1.1e-67	poly(glycerol-phosphate) alpha-glucosyltransferase [EC:2.4.1.52]
CP005080.1_5892	K00632	515.37	574.8	3.8e-173	acetyl-CoA acyltransferase [EC:2.3.1.16]
CP005080.1_5893	K03684	213.07	486.3	4.4e-146	ribonuclease D [EC:3.1.13.5]
CP005080.1_5896	K01599	181.83	524.2	1.8e-157	uroporphyrinogen decarboxylase [EC:4.1.1.37]
CP005080.1_5897	K18367	477.53	500.7	1.2e-150	CoA-dependent NAD(P)H sulfur oxidoreductase [EC:1.8.1.18]
CP005080.1_5899	K00231	144.83	473.6	4e-142	protoporphyrinogen/coproporphyrinogen III oxidase [EC:1.3.3.4 1.3.3.15]
CP005080.1_5900	K00435	268.83	330.1	3.1e-99	hydrogen peroxide-dependent heme synthase [EC:1.3.98.5]
CP005080.1_5903	K09930	59.60	396.4	4.8e-119	uncharacterized protein
CP005080.1_5904	K08995	39.93	119.5	9.1e-35	putative membrane protein
CP005080.1_5905	K01560	141.93	200.0	3e-59	2-haloacid dehalogenase [EC:3.8.1.2]
CP005080.1_5912	K03623	37.70	38.3	3.4e-10	ribonuclease inhibitor
CP005080.1_5914	K13633	271.97	289.7	1.2e-86	AraC family transcriptional regulator, transcriptional activator FtrA
CP005080.1_5915	K22278	73.27	152.1	9.6e-45	peptidoglycan-N-acetylglucosamine deacetylase [EC:3.5.1.104]
CP005080.1_5916	K07093	112.23	390.8	6.5e-117	uncharacterized protein
CP005080.1_5917	K04063	57.63	100.4	4.9e-29	lipoyl-dependent peroxiredoxin [EC:1.11.1.28]
CP005080.1_5921	K01924	374.10	605.4	5.3e-182	UDP-N-acetylmuramate--alanine ligase [EC:6.3.2.8]
CP005080.1_5922	K07305	92.37	220.2	1.1e-65	peptide-methionine (R)-S-oxide reductase [EC:1.8.4.12]
CP005080.1_5932	K12132	224.03	256.4	1.3e-76	eukaryotic-like serine/threonine-protein kinase [EC:2.7.11.1]
CP005080.1_5933	K12132	224.03	263.1	1.2e-78	eukaryotic-like serine/threonine-protein kinase [EC:2.7.11.1]
CP005080.1_5934	K00060	344.93	575.3	3.4e-173	threonine 3-dehydrogenase [EC:1.1.1.103]
CP005080.1_5935	K00639	513.17	679.4	1.5e-204	glycine C-acetyltransferase [EC:2.3.1.29]
CP005080.1_5941	K01236	428.03	876.3	5.6e-264	maltooligosyltrehalose trehalohydrolase [EC:3.2.1.141]
CP005080.1_5942	K05350	554.93	577.8	1.6e-173	beta-glucosidase [EC:3.2.1.21]
CP005080.1_5943	K01206	70.43	244.6	9.1e-73	alpha-L-fucosidase [EC:3.2.1.51]
CP005080.1_5947	K06044	492.80	1115.4	0	(1->4)-alpha-D-glucan 1-alpha-D-glucosylmutase [EC:5.4.99.15]
CP005080.1_5948	K01214	723.90	1182.1	0	isoamylase [EC:3.2.1.68]
CP005080.1_5950	K02342	101.50	134.2	2.2e-39	DNA polymerase III subunit epsilon [EC:2.7.7.7]
CP005080.1_5953	K02025	276.90	316.3	9.7e-95	multiple sugar transport system permease protein
CP005080.1_5956	K21251	433.83	704.1	2e-212	macrolide glycosyltransferase
CP005080.1_5960	K02021	479.07	556.8	3.5e-167	putative ABC transport system ATP-binding protein
CP005080.1_5962	K25286	277.13	347.0	8.5e-104	iron-siderophore transport system substrate-binding protein
CP005080.1_5963	K23186	409.23	466.2	6.7e-140	iron-siderophore transport system permease protein
CP005080.1_5964	K23187	358.37	431.2	2.7e-129	iron-siderophore transport system permease protein
CP005080.1_5965	K23188	469.87	528.1	1.4e-158	iron-siderophore transport system ATP-binding protein [EC:7.2.2.17 7.2.2.-]
CP005080.1_5965	K25130	419.90	426.6	1.1e-128	iron-siderophore transport system ATP-binding protein [EC:7.2.2.-]
CP005080.1_5965	K10829	365.87	368.6	8.3e-111	ferric hydroxamate transport system ATP-binding protein [EC:7.2.2.16]
CP005080.1_5965	K02013	238.30	360.4	5.9e-108	iron complex transport system ATP-binding protein [EC:7.2.2.-]
CP005080.1_5968	K03795	119.77	211.6	8.9e-63	sirohydrochlorin cobaltochelatase [EC:4.99.1.3]
CP005080.1_5969	K15554	248.37	277.5	4.1e-83	sulfonate transport system permease protein
CP005080.1_5969	K02050	145.27	184.9	1.1e-54	NitT/TauT family transport system permease protein
CP005080.1_5970	K15555	333.60	358.0	2.2e-107	sulfonate transport system ATP-binding protein [EC:7.6.2.14]
CP005080.1_5970	K02049	352.60	353.7	9.4e-106	NitT/TauT family transport system ATP-binding protein
CP005080.1_5971	K15553	187.27	248.3	8.6e-74	sulfonate transport system substrate-binding protein
CP005080.1_5971	K02051	157.20	162.1	1.1e-47	NitT/TauT family transport system substrate-binding protein
CP005080.1_5972	K00956	516.37	672.8	1.4e-202	sulfate adenylyltransferase subunit 1 [EC:2.7.7.4]
CP005080.1_5973	K00957	220.20	557.2	3.1e-167	sulfate adenylyltransferase subunit 2 [EC:2.7.7.4]
CP005080.1_5975	K00390	157.87	310.4	5.7e-93	phosphoadenosine phosphosulfate reductase [EC:1.8.4.8 1.8.4.10]
CP005080.1_5977	K00392	592.07	783.3	1.1e-235	sulfite reductase (ferredoxin) [EC:1.8.7.1]
CP005080.1_5980	K09456	282.10	900.8	1.6e-271	putative acyl-CoA dehydrogenase
CP005080.1_5981	K07058	40.93	243.9	1.8e-72	membrane protein
CP005080.1_5985	K01040	120.73	329.4	5.7e-99	glutaconate CoA-transferase, subunit B [EC:2.8.3.12]
CP005080.1_5986	K01039	171.33	399.3	1e-119	glutaconate CoA-transferase, subunit A [EC:2.8.3.12]
CP005080.1_5987	K21757	301.17	323.5	6.3e-97	LysR family transcriptional regulator, benzoate and cis,cis-muconate-responsive activator of ben and cat genes
CP005080.1_5988	K00626	488.13	489.7	5e-147	acetyl-CoA C-acetyltransferase [EC:2.3.1.9]
CP005080.1_5997	K06949	90.53	329.4	1.3e-98	ribosome biogenesis GTPase / thiamine phosphate phosphatase [EC:3.6.1.- 3.1.3.100]
CP005080.1_5998	K13529	347.03	645.9	3.2e-194	AraC family transcriptional regulator, regulatory protein of adaptative response / DNA-3-methyladenine glycosylase II [EC:3.2.2.21]
CP005080.1_5999	K09793	59.33	85.7	1.2e-24	uncharacterized protein
CP005080.1_6000	K23356	46.03	73.7	5.5e-21	HTH-type transcriptional regulator, sugar sensing transcriptional regulator

CP005080.1_6002	K00156	698.60	829.9	6.4e-250	pyruvate dehydrogenase (quinone) [EC:1.2.5.1]
CP005080.1_6003	K01191	442.83	978.6	1.6e-294	alpha-mannosidase [EC:3.2.1.24]
CP005080.1_6004	K04757	53.50	60.0	1e-16	serine/threonine-protein kinase RsbW [EC:2.7.11.1]
CP005080.1_6009	K09684	188.00	249.7	2.9e-74	PucR family transcriptional regulator, purine catabolism regulatory protein
CP005080.1_6011	K03518	235.73	265.6	1.2e-79	aerobic carbon-monoxide dehydrogenase small subunit [EC:1.2.5.3]
CP005080.1_6013	K06901	93.90	454.1	2.5e-136	adenine/guanine/hypoxanthine permease
CP005080.1_6014	K07402	68.07	409.3	8e-123	xanthine dehydrogenase accessory factor
CP005080.1_6021	K00540	74.27	202.4	4.1e-60	F420H(2)-dependent quinone reductase [EC:1.1.98.-]
CP005080.1_6022	K02078	39.80	40.8	9.2e-11	acyl carrier protein
CP005080.1_6025	K07004	132.70	519.2	9.6e-156	uncharacterized protein
CP005080.1_6029	K15554	248.37	276.7	7.5e-83	sulfonate transport system permease protein
CP005080.1_6029	K02050	145.27	187.9	1.4e-55	NitT/TauT family transport system permease protein
CP005080.1_6031	K24116	523.57	624.2	4.6e-188	N-acetyl-S-(2-succino)cysteine monooxygenase [EC:1.14.-.-]
CP005080.1_6032	K06147	612.93	680.2	1.6e-204	ATP-binding cassette, subfamily B, bacterial
CP005080.1_6033	K23107	334.20	358.3	2.7e-107	1-deoxyxylulose-5-phosphate synthase [EC:1.1.-.-]
CP005080.1_6037	K07393	68.87	481.8	8.6e-145	glutathionyl-hydroquinone reductase [EC:1.8.5.7]
CP005080.1_6040	K21685	120.43	374.6	3.3e-112	LuxR family transcriptional regulator, regulator of acetate metabolism
CP005080.1_6042	K00666	594.33	704.1	9.5e-212	fatty-acyl-CoA synthase [EC:6.2.1.-]
CP005080.1_6046	K01608	721.63	1090.3	0	tartronate-semialdehyde synthase [EC:4.1.1.47]
CP005080.1_6047	K07006	34.40	182.1	6.7e-54	uncharacterized protein
CP005080.1_6049	K22894	131.70	323.0	1.3e-96	SARP family transcriptional regulator, regulator of embCAB operon
CP005080.1_6053	K00600	75.20	527.2	2.4e-158	glycine hydroxymethyltransferase [EC:2.1.2.1]
CP005080.1_6056	K01778	61.00	249.1	2.8e-74	diaminopimelate epimerase [EC:5.1.1.7]
CP005080.1_6059	K03781	68.70	776.3	1.3e-233	catalase [EC:1.11.1.6]
CP005080.1_6060	K00042	404.30	440.6	2.4e-132	2-hydroxy-3-oxopropionate reductase [EC:1.1.1.60]
CP005080.1_6060	K00020	348.20	350.0	1.1e-104	3-hydroxyisobutyrate dehydrogenase [EC:1.1.1.31]
CP005080.1_6061	K01816	141.03	280.9	7.6e-84	hydroxypyruvate isomerase [EC:5.3.1.22]
CP005080.1_6063	K12373	123.03	414.5	2.7e-124	hexosaminidase [EC:3.2.1.52]
CP005080.1_6064	K17331	321.87	429.0	3.5e-129	N,N'-diacetylchitobiose transport system permease protein
CP005080.1_6064	K02026	280.30	281.9	2.9e-84	multiple sugar transport system permease protein
CP005080.1_6065	K17330	299.37	419.7	2.4e-126	N,N'-diacetylchitobiose transport system permease protein
CP005080.1_6065	K02025	276.90	281.3	4e-84	multiple sugar transport system permease protein
CP005080.1_6066	K17329	313.30	541.9	5.1e-163	N,N'-diacetylchitobiose transport system substrate-binding protein
CP005080.1_6066	K02027	193.17	229.4	4.2e-68	multiple sugar transport system substrate-binding protein
CP005080.1_6067	K03710	178.10	209.5	4e-62	GntR family transcriptional regulator
CP005080.1_6068	K07404	130.17	383.1	8.5e-115	6-phosphogluconolactonase [EC:3.1.1.31]
CP005080.1_6071	K16840	169.10	240.6	9.1e-72	2-oxo-4-hydroxy-4-carboxy-5-ureidoimidazoline decarboxylase [EC:4.1.1.97]
CP005080.1_6072	K07127	118.20	131.1	1.8e-38	5-hydroxyisourate hydrolase [EC:3.5.2.17]
CP005080.1_6073	K00365	211.80	415.9	8.7e-125	urate oxidase [EC:1.7.3.3]
CP005080.1_6074	K24206	593.73	652.8	2e-196	uric acid transporter
CP005080.1_6075	K18456	525.57	782.9	1.5e-235	8-oxoguanine deaminase [EC:3.5.4.32]
CP005080.1_6076	K24206	593.73	727.2	6.3e-219	uric acid transporter
CP005080.1_6077	K01233	36.97	394.3	3.7e-118	chitosanase [EC:3.2.1.132]
CP005080.1_6088	K01638	129.17	844.0	4.7e-254	malate synthase [EC:2.3.3.9]
CP005080.1_6089	K07141	98.60	183.5	2.4e-54	molybdenum cofactor cytidylyltransferase [EC:2.7.7.76]
CP005080.1_6093	K09813	324.13	505.7	3.3e-152	hemin transport system permease protein
CP005080.1_6094	K09814	301.07	369.6	3.3e-111	hemin transport system ATP-binding protein [EC:7.6.2.-]
CP005080.1_6095	K13641	274.47	338.7	1.8e-101	IclR family transcriptional regulator, acetate operon repressor
CP005080.1_6096	K01466	373.03	735.1	3.9e-221	allantoinase [EC:3.5.2.5]
CP005080.1_6097	K01477	148.47	498.1	1.5e-149	allantoicase [EC:3.5.3.4]
CP005080.1_6098	K06221	349.23	396.2	3.4e-119	2,5-diketo-D-gluconate reductase A [EC:1.1.1.346]
CP005080.1_6106	K20420	428.67	430.9	4.1e-129	2-hydroxy-5-methyl-1-naphthoate 7-hydroxylase [EC:1.14.15.31]
CP005080.1_6107	K00010	272.37	349.8	8.6e-105	myo-inositol 2-dehydrogenase / D-chiro-inositol 1-dehydrogenase [EC:1.1.1.18 1.1.1.369]
CP005080.1_6108	K03710	178.10	221.7	7.7e-66	GntR family transcriptional regulator
CP005080.1_6109	K02058	208.73	339.8	1.2e-101	simple sugar transport system substrate-binding protein
CP005080.1_6110	K02057	301.83	357.8	3.4e-107	simple sugar transport system permease protein
CP005080.1_6112	K25026	274.23	395.9	9.6e-119	glucokinase [EC:2.7.1.2]
CP005080.1_6114	K14727	349.27	759.5	1.1e-228	3-oxoadipate enol-lactonase / 4-carboxymuconolactone decarboxylase [EC:3.1.1.24 4.1.1.44]
CP005080.1_6114	K01055	250.30	274.4	9.5e-82	3-oxoadipate enol-lactonase [EC:3.1.1.24]
CP005080.1_6114	K01607	17.10	130.3	4.5e-38	4-carboxymuconolactone decarboxylase [EC:4.1.1.44]
CP005080.1_6116	K01142	228.27	293.4	1.3e-87	exodeoxyribonuclease III [EC:3.1.11.2]
CP005080.1_6123	K10008	442.50	503.1	2.2e-151	glutamate transport system ATP-binding protein [EC:7.4.2.1]
CP005080.1_6123	K02028	386.70	438.2	1.6e-131	polar amino acid transport system ATP-binding protein [EC:7.4.2.1]
CP005080.1_6124	K10005	314.87	400.3	4.5e-120	glutamate transport system substrate-binding protein
CP005080.1_6124	K02030	76.77	125.0	1.7e-36	polar amino acid transport system substrate-binding protein
CP005080.1_6125	K10006	244.67	301.9	1.9e-90	glutamate transport system permease protein
CP005080.1_6126	K10007	269.30	404.6	2.8e-121	glutamate transport system permease protein

CP005080.1_6129	K03980	202.87	543.5	2.9e-163	putative peptidoglycan lipid II flippase
CP005080.1_6130	K01953	106.30	521.8	1.3e-156	asparagine synthase (glutamine-hydrolysing) [EC:6.3.5.4]
CP005080.1_6132	K19222	83.07	117.2	3e-34	1,4-dihydroxy-2-naphthoyl-CoA hydrolase [EC:3.1.2.28]
CP005080.1_6133	K27292	433.83	639.1	2e-192	3-aminoavenalumate diazotase [EC:6.7.1.2]
CP005080.1_6134	K02078	39.80	49.2	2.4e-13	acyl carrier protein
CP005080.1_6137	K02078	39.80	57.3	8.3e-16	acyl carrier protein
CP005080.1_6138	K00059	269.80	320.8	5.1e-96	3-oxoacyl-[acyl-carrier protein] reductase [EC:1.1.1.100]
CP005080.1_6140	K00681	540.00	624.8	8.6e-188	gamma-glutamyltranspeptidase / glutathione hydrolase [EC:2.3.2.2 3.4.19.13]
CP005080.1_6142	K01265	141.83	240.4	1.3e-71	methionyl aminopeptidase [EC:3.4.11.18]
CP005080.1_6147	K01431	270.03	366.2	1.6e-109	beta-ureidopropionase [EC:3.5.1.6]
CP005080.1_6149	K01464	397.50	649.1	2.9e-195	dihydropyrimidinase [EC:3.5.2.2]
CP005080.1_6150	K00320	221.33	249.9	2.1e-74	5,10-methylenetetrahydromethanopterin reductase [EC:1.5.98.2]
CP005080.1_6152	K03457	180.17	556.0	4.5e-167	nucleobase:cation symporter-1, NCS1 family
CP005080.1_6153	K09992	76.67	161.1	1.7e-47	uncharacterized protein
CP005080.1_6157	K01426	303.90	395.6	2.4e-118	amidase [EC:3.5.1.4]
CP005080.1_6158	K00031	117.90	157.8	2e-46	isocitrate dehydrogenase [EC:1.1.1.42]
CP005080.1_6162	K03800	254.90	301.8	4.7e-90	lipoate---protein ligase [EC:6.3.1.20]
CP005080.1_6164	K01692	342.50	406.7	5.3e-122	enoyl-CoA hydratase [EC:4.2.1.17]
CP005080.1_6165	K00020	348.20	381.8	2.7e-114	3-hydroxyisobutyrate dehydrogenase [EC:1.1.1.31]
CP005080.1_6166	K05605	174.27	385.6	1.3e-115	3-hydroxyisobutyryl-CoA hydrolase [EC:3.1.2.4]
CP005080.1_6167	K11538	513.73	600.9	6.3e-181	isobutyryl-CoA dehydrogenase [EC:1.3.99.-]
CP005080.1_6168	K00140	503.87	860.2	6.7e-259	malonate-semialdehyde dehydrogenase (acetylating) / methylmalonate-semialdehyde dehydrogenase [EC:1.2.1.18 1.2.1.27]
CP005080.1_6169	K02228	136.20	410.3	3.5e-123	precorrin-6A synthase [EC:2.1.1.152]
CP005080.1_6171	K00549	27.93	1060.0	3.1e-319	5-methyltetrahydropteroyltriglutamate--homocysteine methyltransferase [EC:2.1.1.14]
CP005080.1_6172	K13745	417.70	636.8	2.1e-191	L-2,4-diaminobutyrate decarboxylase [EC:4.1.1.86]
CP005080.1_6173	K27501	461.57	650.8	4e-196	putrescine N-hydroxylase [EC:1.14.13.252]
CP005080.1_6174	K03522	225.07	351.0	6.2e-105	electron transfer flavoprotein alpha subunit
CP005080.1_6175	K03521	77.00	266.6	1.8e-79	electron transfer flavoprotein beta subunit
CP005080.1_6176	K24699	97.17	116.7	3.8e-34	TetR/AcrR family transcriptional regulator, regulator of mycofactocin system
CP005080.1_6183	K02182	500.83	648.1	5.1e-195	carnitine-CoA ligase [EC:6.2.1.48]
CP005080.1_6184	K06978	225.60	473.7	4.7e-142	uncharacterized protein
CP005080.1_6191	K00648	229.33	250.3	1.4e-74	3-oxoacyl-[acyl-carrier-protein] synthase III [EC:2.3.1.180]
CP005080.1_6192	K00648	229.33	318.2	3.5e-95	3-oxoacyl-[acyl-carrier-protein] synthase III [EC:2.3.1.180]
CP005080.1_6195	K25147	114.13	172.0	5.9e-51	beta-exotoxin I transport system permease protein
CP005080.1_6196	K25148	372.93	374.0	3e-112	beta-exotoxin I transport system ATP-binding protein
CP005080.1_6196	K01990	262.37	281.2	6.4e-84	ABC-2 type transport system ATP-binding protein
CP005080.1_6202	K23518	203.53	245.6	4.8e-73	O-acetyl-ADP-ribose deacetylase [EC:3.1.1.106]
CP005080.1_6203	K13529	347.03	764.3	4.8e-230	AraC family transcriptional regulator, regulatory protein of adaptative response / DNA-3-methyladenine glycosylase II [EC:3.2.2.21]
CP005080.1_6204	K00567	174.97	229.5	2.6e-68	methylated-DNA-[protein]-cysteine S-methyltransferase [EC:2.1.1.63]
CP005080.1_6205	K23541	31.17	131.7	1.3e-38	Ca2+/H+ antiporter, TMEM165/GDT1 family
CP005080.1_6207	K12410	226.47	279.5	1.4e-83	NAD-dependent protein deacetylase/lipoamidase [EC:2.3.1.286 2.3.1.313]
CP005080.1_6210	K00865	57.57	510.5	1.9e-153	glycerate 2-kinase [EC:2.7.1.165]
CP005080.1_6212	K01996	317.37	359.5	1.2e-107	branched-chain amino acid transport system ATP-binding protein
CP005080.1_6213	K01995	319.03	401.2	4.1e-120	branched-chain amino acid transport system ATP-binding protein
CP005080.1_6214	K01997	149.83	244.2	1.1e-72	branched-chain amino acid transport system permease protein
CP005080.1_6215	K01998	245.40	271.4	5.7e-81	branched-chain amino acid transport system permease protein
CP005080.1_6216	K01999	141.80	170.6	2.8e-50	branched-chain amino acid transport system substrate-binding protein
CP005080.1_6218	K12132	224.03	240.9	7.1e-72	eukaryotic-like serine/threonine-protein kinase [EC:2.7.11.1]
CP005080.1_6218	K01999	141.80	166.6	4.6e-49	branched-chain amino acid transport system substrate-binding protein
CP005080.1_6223	K17103	126.47	172.6	6.7e-51	CDP-diacylglycerol---serine O-phosphatidyltransferase [EC:2.7.8.8]
CP005080.1_6224	K01613	33.77	101.7	1.7e-29	phosphatidylserine decarboxylase [EC:4.1.1.65]
CP005080.1_6225	K00249	382.07	390.8	4.6e-117	acyl-CoA dehydrogenase [EC:1.3.8.7]
CP005080.1_6226	K18290	184.90	194.2	4.3e-58	itaconyl-CoA hydratase [EC:4.2.1.56]
CP005080.1_6227	K01644	132.63	380.0	6.1e-114	citrate lyase subunit beta / citryl-CoA lyase [EC:4.1.3.34]
CP005080.1_6228	K14447	943.53	1281.1	0	ethylmalonyl-CoA mutase [EC:5.4.99.63]
CP005080.1_6229	K17829	478.20	880.1	8.8e-266	crotonyl-CoA reductase [EC:1.3.1.86]
CP005080.1_6231	K07448	34.87	127.6	1.9e-37	restriction system protein
CP005080.1_6232	K24699	97.17	108.9	9.7e-32	TetR/AcrR family transcriptional regulator, regulator of mycofactocin system
CP005080.1_6233	K00074	404.50	449.3	7.7e-135	3-hydroxybutyryl-CoA dehydrogenase [EC:1.1.1.157]
CP005080.1_6240	K19265	304.10	592.9	2.3e-178	L-glyceraldehyde 3-phosphate reductase [EC:1.1.1.-]
CP005080.1_6242	K16203	84.30	320.6	4.2e-96	D-amino peptidase [EC:3.4.11.-]
CP005080.1_6245	K01297	137.13	376.6	5.1e-113	muramoyltetrapeptide carboxypeptidase [EC:3.4.17.13]
CP005080.1_6247	K08992	32.97	42.9	2.2e-11	lipopolysaccharide assembly protein A
CP005080.1_6248	K03790	144.10	275.9	2.4e-82	[ribosomal protein S5]-alanine N-acetyltransferase [EC:2.3.1.267]
CP005080.1_6249	K01476	187.27	201.8	9.3e-60	arginase [EC:3.5.3.1]
CP005080.1_6252	K19302	106.60	115.1	1.7e-33	undecaprenyl-diphosphatase [EC:3.6.1.27]
CP005080.1_6254	K03975	59.97	100.2	4.7e-29	membrane-associated protein

CP005080.1_6255	K13985	99.67	112.2	1.1e-32	N-acyl-phosphatidylethanolamine-hydrolysing phospholipase D [EC:3.1.4.54]
CP005080.1_6256	K08972	34.93	43.9	1.1e-11	putative membrane protein
CP005080.1_6257	K02529	268.37	315.9	2.2e-94	LacI family transcriptional regulator, galactose operon repressor
CP005080.1_6258	K08995	39.93	49.2	2.6e-13	putative membrane protein
CP005080.1_6260	K01972	119.00	735.9	2.2e-221	DNA ligase (NAD+) [EC:6.5.1.2]
CP005080.1_6261	K03569	108.73	119.6	5.1e-35	rod shape-determining protein MreB and related proteins
CP005080.1_6263	K12257	548.43	791.0	4.8e-238	SecD/SecF fusion protein
CP005080.1_6265	K01724	20.90	89.5	1e-25	4a-hydroxytetrahydrobiopterin dehydratase [EC:4.2.1.96]
CP005080.1_6267	K19267	112.87	162.4	8.4e-48	NAD(P)H dehydrogenase (quinone) [EC:1.6.5.2]
CP005080.1_6271	K06221	349.23	476.6	1.2e-143	2,5-diketo-D-gluconate reductase A [EC:1.1.1.346]
CP005080.1_6272	K01897	430.97	495.7	8.9e-149	long-chain acyl-CoA synthetase [EC:6.2.1.3]
CP005080.1_6274	K14347	118.47	404.8	2.5e-121	solute carrier family 10 (sodium/bile acid cotransporter), member 7
CP005080.1_6276	K02379	83.90	387.3	3.4e-116	FdhD protein
CP005080.1_6277	K00648	229.33	363.2	7.8e-109	3-oxoacyl-[acyl-carrier-protein] synthase III [EC:2.3.1.180]
CP005080.1_6278	K25026	274.23	307.7	5.6e-92	glucokinase [EC:2.7.1.2]
CP005080.1_6279	K10441	693.10	772.5	1.4e-232	ribose transport system ATP-binding protein [EC:7.5.2.7]
CP005080.1_6280	K10440	333.83	360.1	6.7e-108	ribose transport system permease protein
CP005080.1_6281	K10439	190.90	234.2	1.5e-69	ribose transport system substrate-binding protein
CP005080.1_6284	K09992	76.67	285.4	2.8e-85	uncharacterized protein
CP005080.1_6285	K01858	73.77	345.0	3.6e-103	myo-inositol-1-phosphate synthase [EC:5.5.1.4]
CP005080.1_6287	K03079	151.37	182.0	6.3e-54	L-ribulose-5-phosphate 3-epimerase [EC:5.1.3.22]
CP005080.1_6289	K07051	132.30	500.8	4.5e-151	uncharacterized protein
CP005080.1_6294	K02529	268.37	371.0	4.3e-111	LacI family transcriptional regulator, galactose operon repressor
CP005080.1_6295	K10441	693.10	787.0	6e-237	ribose transport system ATP-binding protein [EC:7.5.2.7]
CP005080.1_6295	K23537	596.97	597.0	2.4e-179	general nucleoside transport system ATP-binding protein
CP005080.1_6296	K10440	333.83	448.7	1e-134	ribose transport system permease protein
CP005080.1_6296	K10439	190.90	325.7	3e-97	ribose transport system substrate-binding protein
CP005080.1_6297	K00852	237.13	391.3	3.5e-117	ribokinase [EC:2.7.1.15]
CP005080.1_6298	K06726	111.10	182.4	2.3e-54	D-ribose pyranase [EC:5.4.99.62]
CP005080.1_6304	K07284	60.53	63.5	7.2e-18	sortase A [EC:3.4.22.70]
CP005080.1_6306	K07642	293.97	294.5	5.3e-88	two-component system, OmpR family, sensor histidine kinase BaeS [EC:2.7.13.3]
CP005080.1_6307	K02483	242.00	250.6	1.4e-74	two-component system, OmpR family, response regulator
CP005080.1_6311	K20420	428.67	548.2	1.4e-164	2-hydroxy-5-methyl-1-naphthoate 7-hydroxylase [EC:1.14.15.31]
CP005080.1_6313	K00130	687.90	700.1	9.2e-211	betaine-aldehyde dehydrogenase [EC:1.2.1.8]
CP005080.1_6315	K02000	415.90	571.0	1.8e-171	glycine betaine/proline transport system ATP-binding protein [EC:7.6.2.9]
CP005080.1_6316	K02001	326.57	629.0	6e-189	glycine betaine/proline transport system permease protein
CP005080.1_6317	K02002	122.57	198.4	8.7e-59	glycine betaine/proline transport system substrate-binding protein
CP005080.1_6320	K07085	209.40	426.9	4e-128	putative transport protein
CP005080.1_6322	K07114	102.73	118.5	1.2e-34	Ca-activated chloride channel homolog
CP005080.1_6324	K01805	102.00	305.6	2.8e-91	xylose isomerase [EC:5.3.1.5]
CP005080.1_6329	K27888	37.73	106.4	5e-31	phospholipid scramblase
CP005080.1_6334	K07131	45.27	85.5	1.5e-24	uncharacterized protein
CP005080.1_6336	K06945	72.87	308.7	2.7e-92	uncharacterized protein
CP005080.1_6338	K01222	394.63	570.7	8.9e-172	6-phospho-beta-glucosidase [EC:3.2.1.86]
CP005080.1_6340	K04757	53.50	62.3	2e-17	serine/threonine-protein kinase RsbW [EC:2.7.11.1]
CP005080.1_6345	K13633	271.97	466.8	2e-140	AraC family transcriptional regulator, transcriptional activator FtrA
CP005080.1_6346	K09858	46.27	194.4	8e-58	SEC-C motif domain protein
CP005080.1_6355	K02065	294.10	470.1	3.9e-141	phospholipid/cholesterol/gamma-HCH transport system ATP-binding protein
CP005080.1_6356	K02066	41.80	286.8	1.4e-85	phospholipid/cholesterol/gamma-HCH transport system permease protein
CP005080.1_6357	K02066	41.80	223.2	2.4e-66	phospholipid/cholesterol/gamma-HCH transport system permease protein
CP005080.1_6358	K02067	83.03	163.3	3.9e-48	phospholipid/cholesterol/gamma-HCH transport system substrate-binding protein
CP005080.1_6359	K02067	83.03	188.2	1.1e-55	phospholipid/cholesterol/gamma-HCH transport system substrate-binding protein
CP005080.1_6360	K02067	83.03	183.8	2.5e-54	phospholipid/cholesterol/gamma-HCH transport system substrate-binding protein
CP005080.1_6361	K02067	83.03	185.3	8.6e-55	phospholipid/cholesterol/gamma-HCH transport system substrate-binding protein
CP005080.1_6362	K02067	83.03	213.8	2e-63	phospholipid/cholesterol/gamma-HCH transport system substrate-binding protein
CP005080.1_6363	K02067	83.03	169.4	5.5e-50	phospholipid/cholesterol/gamma-HCH transport system substrate-binding protein
CP005080.1_6364	K18481	51.20	74.0	7.4e-21	Mce-associated membrane protein
CP005080.1_6365	K18481	51.20	108.9	1.7e-31	Mce-associated membrane protein
CP005080.1_6366	K18481	51.20	110.8	4.6e-32	Mce-associated membrane protein
CP005080.1_6367	K18481	51.20	126.3	8.5e-37	Mce-associated membrane protein
CP005080.1_6369	K07315	145.67	194.8	9.8e-58	phosphoserine phosphatase RsbU/P [EC:3.1.3.3]
CP005080.1_6373	K22468	274.30	492.1	6.1e-148	polyphosphate kinase [EC:2.7.4.34]
CP005080.1_6374	K01971	209.27	233.3	2.7e-69	bifunctional non-homologous end joining protein LigD [EC:6.5.1.1]
CP005080.1_6375	K01971	209.27	351.7	4.7e-105	bifunctional non-homologous end joining protein LigD [EC:6.5.1.1]
CP005080.1_6378	K09992	76.67	184.9	9.4e-55	uncharacterized protein
CP005080.1_6385	K15554	248.37	286.8	6.1e-86	sulfonate transport system permease protein
CP005080.1_6385	K02050	145.27	218.6	6.9e-65	NitT/TauT family transport system permease protein

CP005080.1_6387	K15553	187.27	208.0	1.5e-61	sulfonate transport system substrate-binding protein
CP005080.1_6388	K04091	325.40	426.4	9e-128	alkanesulfonate monooxygenase [EC:1.14.14.5 1.14.14.34]
CP005080.1_6389	K06996	53.13	115.7	1.4e-33	uncharacterized protein
CP005080.1_6390	K18955	70.43	117.6	2.4e-34	WhiB family transcriptional regulator, redox-sensing transcriptional regulator
CP005080.1_6392	K03921	161.33	311.0	5.6e-93	acyl-[acyl-carrier protein] desaturase [EC:1.14.19.2 1.14.19.11 1.14.19.26]
CP005080.1_6393	K03701	341.57	843.8	6.8e-254	excinuclease ABC subunit A
CP005080.1_6396	K09780	27.50	45.3	3.5e-12	uncharacterized protein
CP005080.1_6397	K05982	231.57	355.8	1.4e-106	deoxyribonuclease V [EC:3.1.21.7]
CP005080.1_6400	K01796	337.03	587.6	8.6e-177	alpha-methylacyl-CoA racemase [EC:5.1.99.4]
CP005080.1_6401	K00255	595.00	636.2	2.1e-191	long-chain-acyl-CoA dehydrogenase [EC:1.3.8.8]
CP005080.1_6401	K00249	382.07	393.9	5.3e-118	acyl-CoA dehydrogenase [EC:1.3.8.7]
CP005080.1_6403	K01782	813.67	989.0	1.2e-297	3-hydroxyacyl-CoA dehydrogenase / enoyl-CoA hydratase / 3-hydroxybutyryl-CoA epimerase [EC:1.1.1.35 4.2.1.17 5.1.2.3]
CP005080.1_6410	K00273	130.37	311.6	2.9e-93	D-amino-acid oxidase [EC:1.4.3.3]
CP005080.1_6412	K06959	437.10	1245.6	0	protein Tex
CP005080.1_6414	K13633	271.97	322.1	1.7e-96	AraC family transcriptional regulator, transcriptional activator FtrA
CP005080.1_6415	K18199	184.27	270.3	1.2e-80	cyclohexyl-isocyanide hydratase [EC:4.2.1.103]
CP005080.1_6417	K21908	32.87	67.1	6.5e-19	membrane protein HdeD
CP005080.1_6418	K04757	53.50	65.9	1.6e-18	serine/threonine-protein kinase RsbW [EC:2.7.11.1]
CP005080.1_6419	K01823	34.03	161.6	1.3e-47	isopentenyl-diphosphate Delta-isomerase [EC:5.3.3.2]
CP005080.1_6420	K03824	68.20	199.8	2.7e-59	putative acetyltransferase [EC:2.3.1.-]
CP005080.1_6420	K07154	30.30	70.1	5.6e-20	serine/threonine-protein kinase HipA [EC:2.7.11.1]
CP005080.1_6421	K16264	312.40	423.2	5.8e-127	cobalt-zinc-cadmium efflux system protein
CP005080.1_6422	K01784	253.97	362.5	1.7e-108	UDP-glucose 4-epimerase [EC:5.1.3.2]
CP005080.1_6425	K00096	164.17	346.1	1.4e-103	glycerol-1-phosphate dehydrogenase [NAD(P)+] [EC:1.1.1.261]
CP005080.1_6428	K09692	218.47	379.8	4.2e-114	teichoic acid transport system permease protein
CP005080.1_6429	K09693	367.70	421.5	1.3e-126	teichoic acid transport system ATP-binding protein [EC:7.5.2.4]
CP005080.1_6430	K02291	166.43	206.7	3.1e-61	15-cis-phytoene synthase [EC:2.5.1.32]
CP005080.1_6431	K02291	166.43	324.9	5e-97	15-cis-phytoene synthase [EC:2.5.1.32]
CP005080.1_6432	K21677	275.20	383.0	1.1e-114	hydroxysqualene dehydroxylase [EC:1.17.8.1]
CP005080.1_6433	K13787	299.27	361.3	3.6e-108	geranylgeranyl diphosphate synthase, type I [EC:2.5.1.1 2.5.1.10 2.5.1.29]
CP005080.1_6434	K06045	591.80	963.6	4.3e-290	squalene-hopene/tetraprenyl-beta-curcumene cyclase [EC:5.4.99.17 4.2.1.129]
CP005080.1_6440	K01104	65.07	111.2	2.3e-32	protein-tyrosine phosphatase [EC:3.1.3.48]
CP005080.1_6444	K02495	261.80	419.6	6.6e-126	oxygen-independent coproporphyrinogen III oxidase [EC:1.3.98.3]
CP005080.1_6453	K13985	99.67	313.4	1.2e-93	N-acyl-phosphatidylethanolamine-hydrolysing phospholipase D [EC:3.1.4.54]
CP005080.1_6457	K07131	45.27	96.6	5.8e-28	uncharacterized protein
CP005080.1_6459	K06945	72.87	321.6	3.3e-96	uncharacterized protein
CP005080.1_6461	K02529	268.37	316.9	1.1e-94	LacI family transcriptional regulator, galactose operon repressor
CP005080.1_6462	K02050	145.27	266.7	1.8e-79	NitT/TauT family transport system permease protein
CP005080.1_6463	K02051	157.20	159.4	7.5e-47	NitT/TauT family transport system substrate-binding protein
CP005080.1_6464	K02049	352.60	384.5	4.5e-115	NitT/TauT family transport system ATP-binding protein
CP005080.1_6464	K15555	333.60	359.6	7.1e-108	sulfonate transport system ATP-binding protein [EC:7.6.2.14]
CP005080.1_6468	K09992	76.67	245.3	4.4e-73	uncharacterized protein
CP005080.1_6477	K04068	118.80	149.2	5.5e-44	anaerobic ribonucleoside-triphosphate reductase activating protein [EC:1.97.1.4]
CP005080.1_6482	K01114	129.20	666.2	3.1e-200	phospholipase C [EC:3.1.4.3]
CP005080.1_6485	K07675	253.20	305.4	3.4e-91	two-component system, NarL family, sensor histidine kinase UhpB [EC:2.7.13.3]
CP005080.1_6488	K18928	254.60	371.3	1.2e-111	L-lactate dehydrogenase complex protein LldE
CP005080.1_6489	K18929	437.67	757.4	4.4e-228	L-lactate dehydrogenase complex protein LldF
CP005080.1_6490	K00782	116.30	220.0	1.8e-65	L-lactate dehydrogenase complex protein LldG
CP005080.1_6493	K09763	44.57	231.1	6.2e-69	uncharacterized protein
CP005080.1_6495	K06876	141.60	694.3	8.5e-209	(6-4)DNA photolyase [EC:4.1.99.13]
CP005080.1_6499	K03574	70.57	81.6	2.1e-23	8-oxo-dGTP diphosphatase [EC:3.6.1.55]
CP005080.1_6507	K00219	602.77	1245.8	0	2,4-dienoyl-CoA reductase (NADPH2) [EC:1.3.1.34]
CP005080.1_6508	K15977	60.27	124.1	3.8e-36	putative oxidoreductase
CP005080.1_6510	K06994	260.33	547.4	2e-164	putative drug exporter of the RND superfamily
CP005080.1_6515	K03088	96.50	126.0	6.8e-37	RNA polymerase sigma-70 factor, ECF subfamily
CP005080.1_6516	K22491	123.17	297.1	1.1e-88	MerR family transcriptional regulator, light-induced transcriptional regulator
CP005080.1_6517	K13633	271.97	529.3	2.1e-159	AraC family transcriptional regulator, transcriptional activator FtrA
CP005080.1_6521	K15395	3208.50	5629.6	0	hybrid polyketide synthase / nonribosomal peptide synthetase FtdB
CP005080.1_6526	K19302	106.60	119.2	9.3e-35	undecaprenyl-diphosphatase [EC:3.6.1.27]
CP005080.1_6528	K13483	259.73	320.1	6.1e-96	xanthine dehydrogenase YagT iron-sulfur-binding subunit
CP005080.1_6529	K11178	253.73	529.8	2.2e-159	xanthine dehydrogenase YagS FAD-binding subunit [EC:1.17.1.4]
CP005080.1_6530	K11177	664.90	996.1	7.2e-300	xanthine dehydrogenase YagR molybdenum-binding subunit [EC:1.17.1.4]
CP005080.1_6531	K07402	68.07	394.6	2.4e-118	xanthine dehydrogenase accessory factor
CP005080.1_6540	K01585	258.33	294.7	6.5e-88	arginine decarboxylase [EC:4.1.1.19]
CP005080.1_6551	K03427	116.20	231.9	7.2e-69	type I restriction enzyme M protein [EC:2.1.1.72]
CP005080.1_6551	K01154	44.73	79.3	1e-22	type I restriction enzyme, S subunit [EC:3.1.21.3]
CP005080.1_6552	K01153	169.67	225.7	5.4e-67	type I restriction enzyme, R subunit [EC:3.1.21.3]

CP005080.1_6564	K01579	52.57	240.7	4.9e-72	aspartate 1-decarboxylase [EC:4.1.1.11]
CP005080.1_6564	K03525	120.67	132.1	1.1e-38	type III pantothenate kinase [EC:2.7.1.33]
CP005080.1_6565	K06975	44.10	79.1	1.8e-22	uncharacterized protein
CP005080.1_6568	K03574	70.57	93.7	4.3e-27	8-oxo-dGTP diphosphatase [EC:3.6.1.55]
CP005080.1_6570	K01586	276.10	362.9	1.6e-108	diaminopimelate decarboxylase [EC:4.1.1.20]
CP005080.1_6572	K07223	143.73	549.1	4.2e-165	porphyrinogen peroxidase [EC:1.11.1.-]
CP005080.1_6573	K00033	157.37	756.5	1.1e-227	6-phosphogluconate dehydrogenase [EC:1.1.1.44 1.1.1.343]
CP005080.1_6575	K01928	408.67	565.2	6.9e-170	UDP-N-acetylmuramoyl-L-alanyl-D-glutamate--2,6-diaminopimelate ligase [EC:6.3.2.13]
CP005080.1_6576	K21744	131.27	163.2	4.6e-48	MerR family transcriptional regulator, thiopeptide resistance regulator
CP005080.1_6578	K07006	34.40	150.3	3.3e-44	uncharacterized protein
CP005080.1_6580	K18546	339.70	472.1	9.8e-142	streptogrisin C [EC:3.4.21.-]
CP005080.1_6583	K05350	554.93	648.4	7.4e-195	beta-glucosidase [EC:3.2.1.21]
CP005080.1_6584	K02026	280.30	292.6	1.6e-87	multiple sugar transport system permease protein
CP005080.1_6585	K02025	276.90	286.7	9.5e-86	multiple sugar transport system permease protein
CP005080.1_6586	K02027	193.17	250.8	1.4e-74	multiple sugar transport system substrate-binding protein
CP005080.1_6588	K02529	268.37	296.6	1.6e-88	LacI family transcriptional regulator, galactose operon repressor
CP005080.1_6589	K21687	174.20	244.7	1e-72	resuscitation-promoting factor RpfA
CP005080.1_6592	K04749	63.93	93.9	5.5e-27	anti-sigma B factor antagonist
CP005080.1_6596	K11355	167.37	183.1	8e-55	two-component system, chemotaxis family, response regulator Rcp1
CP005080.1_6599	K03090	268.20	413.4	5e-124	RNA polymerase sigma-B factor
CP005080.1_6602	K23555	261.63	491.4	1.8e-147	lipase maturation factor 1
CP005080.1_6606	K07315	145.67	213.8	1.7e-63	phosphoserine phosphatase RsbU/P [EC:3.1.3.3]
CP005080.1_6606	K04757	53.50	55.8	1.9e-15	serine/threonine-protein kinase RsbW [EC:2.7.11.1]
CP005080.1_6607	K00635	122.90	402.2	2.3e-120	diacylglycerol O-acyltransferase / wax synthase [EC:2.3.1.20 2.3.1.75]
CP005080.1_6608	K10563	196.13	241.0	1e-71	formamidopyrimidine-DNA glycosylase [EC:3.2.2.23 4.2.99.18]
CP005080.1_6610	K02477	121.47	251.9	3.8e-75	two-component system, LytTR family, response regulator
CP005080.1_6612	K14393	287.90	423.4	5.4e-127	cation/acetate symporter
CP005080.1_6613	K02478	269.77	285.3	3.1e-85	two-component system, LytTR family, sensor kinase [EC:2.7.13.3]
CP005080.1_6617	K03088	96.50	144.4	1.8e-42	RNA polymerase sigma-70 factor, ECF subfamily
CP005080.1_6620	K22278	73.27	228.1	7.9e-68	peptidoglycan-N-acetylglucosamine deacetylase [EC:3.5.1.104]
CP005080.1_6630	K11921	265.87	286.6	1.4e-85	LysR family transcriptional regulator, cyn operon transcriptional activator
CP005080.1_6630	K04761	248.97	260.0	1.7e-77	LysR family transcriptional regulator, hydrogen peroxide-inducible genes activator
CP005080.1_6631	K00241	50.80	160.3	2.9e-47	succinate dehydrogenase cytochrome b subunit
CP005080.1_6632	K00239	665.27	719.6	1.2e-216	succinate dehydrogenase flavoprotein subunit [EC:1.3.5.1]
CP005080.1_6633	K00240	157.40	216.2	2.1e-64	succinate dehydrogenase iron-sulfur subunit [EC:1.3.5.1]
CP005080.1_6634	K03701	341.57	1053.8	2.5e-317	excinuclease ABC subunit A
CP005080.1_6637	K09165	32.00	125.9	7.5e-37	dodecin
CP005080.1_6638	K17329	313.30	520.5	1.5e-156	N,N'-diacetylchitobiose transport system substrate-binding protein
CP005080.1_6638	K02027	193.17	200.1	3.2e-59	multiple sugar transport system substrate-binding protein
CP005080.1_6639	K22310	97.17	212.2	3.6e-63	L-ornithine Nalpha-acyltransferase [EC:2.3.2.30]
CP005080.1_6641	K18911	275.33	508.0	6.4e-153	L-histidine Nalpha-methyltransferase [EC:2.1.1.44]
CP005080.1_6642	K07008	199.33	385.6	4.6e-116	gamma-glutamyl hercynylcysteine S-oxide hydrolase [EC:3.5.1.118]
CP005080.1_6643	K18912	366.33	784.0	2.2e-236	gamma-glutamyl hercynylcysteine S-oxide synthase [EC:1.14.99.50]
CP005080.1_6644	K01919	80.70	375.2	2.8e-112	glutamate--cysteine ligase [EC:6.3.2.2]
CP005080.1_6648	K03671	112.43	124.7	2.3e-36	thioredoxin
CP005080.1_6657	K07315	145.67	179.9	3.3e-53	phosphoserine phosphatase RsbU/P [EC:3.1.3.3]
CP005080.1_6666	K27545	366.50	461.0	8e-139	arginine dihydrolase [EC:3.5.3.27]
CP005080.1_6673	K02362	100.93	237.0	1.8e-70	enterobactin synthetase component D / holo-[acyl-carrier protein] synthase [EC:6.3.2.14 2.7.8.7]
CP005080.1_6677	K00059	269.80	277.1	9.9e-83	3-oxoacyl-[acyl-carrier protein] reductase [EC:1.1.1.100]
CP005080.1_6681	K06911	36.97	249.8	2e-74	quercetin 2,3-dioxygenase [EC:1.13.11.24]
CP005080.1_6682	K00380	519.97	991.2	2.5e-298	sulfite reductase (NADPH) flavoprotein alpha-component [EC:1.8.1.2]
CP005080.1_6687	K08223	154.93	459.0	6.3e-138	MFS transporter, FSR family, fosmidomycin resistance protein
CP005080.1_6691	K07396	64.20	90.8	3.6e-26	putative protein-disulfide isomerase
CP005080.1_6692	K02035	249.67	306.9	1.2e-91	peptide/nickel transport system substrate-binding protein
CP005080.1_6693	K02033	263.63	289.2	2.4e-86	peptide/nickel transport system permease protein
CP005080.1_6694	K02034	256.80	265.7	2.9e-79	peptide/nickel transport system permease protein
CP005080.1_6700	K00299	91.57	167.0	2.7e-49	FMN reductase [EC:1.5.1.38]
CP005080.1_6704	K02529	268.37	352.8	1.4e-105	LacI family transcriptional regulator, galactose operon repressor
CP005080.1_6705	K10559	338.50	490.8	7.2e-148	rhamnose transport system substrate-binding protein
CP005080.1_6706	K10561	326.53	384.1	3.9e-115	rhamnose transport system permease protein
CP005080.1_6707	K10560	332.10	381.3	1.1e-114	rhamnose transport system permease protein
CP005080.1_6708	K10562	752.40	850.5	4.3e-256	rhamnose transport system ATP-binding protein [EC:7.5.2.-]
CP005080.1_6708	K10441	693.10	742.5	1.8e-223	ribose transport system ATP-binding protein [EC:7.5.2.7]
CP005080.1_6709	K01820	394.80	650.8	2.4e-196	L-rhamnose isomerase / sugar isomerase [EC:5.3.1.14 5.3.1.-]
CP005080.1_6711	K00848	300.13	756.2	1.1e-227	rhamnulokinase [EC:2.7.1.5]
CP005080.1_6712	K05989	232.07	1025.9	1.4e-308	alpha-L-rhamnosidase [EC:3.2.1.40]
CP005080.1_6717	K23228	462.50	645.5	3.3e-194	ferric hydroxamate transport system permease protein

CP005080.1_6718	K23227	189.93	271.4	4e-81	ferric hydroxamate transport system substrate-binding protein
CP005080.1_6719	K10829	365.87	394.8	8.5e-119	ferric hydroxamate transport system ATP-binding protein [EC:7.2.2.16]
CP005080.1_6719	K02013	238.30	303.7	9.3e-91	iron complex transport system ATP-binding protein [EC:7.2.2.-]
CP005080.1_6722	K02963	75.13	84.2	4.3e-24	small subunit ribosomal protein S18
CP005080.1_6724	K02909	21.83	100.7	3.1e-29	large subunit ribosomal protein L31
CP005080.1_6725	K02913	23.23	78.6	1.5e-22	large subunit ribosomal protein L33
CP005080.1_6726	K02902	22.37	89.7	1.1e-25	large subunit ribosomal protein L28
CP005080.1_6727	K02954	26.53	106.9	4.6e-31	small subunit ribosomal protein S14
CP005080.1_6728	K02911	22.10	36.4	2.1e-09	large subunit ribosomal protein L32
CP005080.1_6733	K02013	238.30	327.3	6.5e-98	iron complex transport system ATP-binding protein [EC:7.2.2.-]
CP005080.1_6738	K09136	53.07	222.2	4.8e-66	ribosomal protein S12 methylthiotransferase accessory factor
CP005080.1_6740	K16013	477.03	524.7	1.7e-157	ATP-binding cassette, subfamily C, bacterial CydD
CP005080.1_6741	K16012	468.53	510.8	2.2e-153	ATP-binding cassette, subfamily C, bacterial CydC
CP005080.1_6742	K07315	145.67	203.2	2.7e-60	phosphoserine phosphatase RsbU/P [EC:3.1.3.3]
CP005080.1_6746	K00505	172.43	223.1	2.8e-66	tyrosinase [EC:1.14.18.1]
CP005080.1_6751	K22278	73.27	77.0	5.9e-22	peptidoglycan-N-acetylglucosamine deacetylase [EC:3.5.1.104]
CP005080.1_6752	K03980	202.87	381.2	3.7e-114	putative peptidoglycan lipid II flippase
CP005080.1_6756	K20816	186.13	324.8	4.8e-98	streptothricin hydrolase [EC:3.5.2.19]
CP005080.1_6757	K13787	299.27	342.1	2.5e-102	geranylgeranyl diphosphate synthase, type I [EC:2.5.1.1 2.5.1.10 2.5.1.29]
CP005080.1_6759	K06996	53.13	78.1	4.1e-22	uncharacterized protein
CP005080.1_6760	K05846	171.73	228.1	8.9e-68	osmoprotectant transport system permease protein
CP005080.1_6761	K05846	171.73	253.0	2.6e-75	osmoprotectant transport system permease protein
CP005080.1_6762	K05845	152.73	301.5	3.8e-90	osmoprotectant transport system substrate-binding protein
CP005080.1_6763	K00540	74.27	147.1	3e-43	F420H(2)-dependent quinone reductase [EC:1.1.98.-]
CP005080.1_6766	K05847	352.27	475.7	5.6e-143	osmoprotectant transport system ATP-binding protein [EC:7.6.2.9]
CP005080.1_6771	K00344	279.00	363.6	7.7e-109	NADPH:quinone reductase [EC:1.6.5.5]
CP005080.1_6775	K02035	249.67	341.6	4e-102	peptide/nickel transport system substrate-binding protein
CP005080.1_6776	K02033	263.63	336.4	1.2e-100	peptide/nickel transport system permease protein
CP005080.1_6777	K02034	256.80	294.3	6.2e-88	peptide/nickel transport system permease protein
CP005080.1_6791	K04477	268.97	391.4	1.2e-117	putative hydrolase
CP005080.1_6794	K07315	145.67	213.1	2.7e-63	phosphoserine phosphatase RsbU/P [EC:3.1.3.3]
CP005080.1_6795	K00528	215.37	576.5	2.8e-173	ferredoxin/flavodoxin---NADP+ reductase [EC:1.18.1.2 1.19.1.1]
CP005080.1_6797	K03333	328.20	404.2	4.1e-121	cholesterol oxidase [EC:1.1.3.6]
CP005080.1_6802	K16167	337.77	536.6	1.1e-161	alkylresorcinol/alkylpyrone synthase
CP005080.1_6803	K16168	117.50	272.3	2.2e-81	methyltransferase
CP005080.1_6805	K08166	431.20	431.9	9.8e-130	MFS transporter, DHA2 family, methylenomycin A resistance protein
CP005080.1_6809	K03704	80.73	122.2	8.3e-36	cold shock protein
CP005080.1_6813	K06048	104.33	445.4	1.1e-133	glutamate---cysteine ligase / carboxylate-amine ligase [EC:6.3.2.2 6.3.-.-]
CP005080.1_6814	K05520	175.93	220.3	1.5e-65	deglycase [EC:3.5.1.124]
CP005080.1_6815	K06282	357.13	380.7	3.6e-114	hydrogenase small subunit [EC:1.12.99.6]
CP005080.1_6817	K04655	153.83	503.1	5e-151	hydrogenase expression/formation protein HypE
CP005080.1_6819	K05375	34.70	133.3	1.5e-39	MbtH protein
CP005080.1_6823	K20469	203.00	401.1	2.4e-120	putative heme transporter
CP005080.1_6826	K00528	215.37	499.1	8e-150	ferredoxin/flavodoxin---NADP+ reductase [EC:1.18.1.2 1.19.1.1]
CP005080.1_6830	K19668	224.60	904.5	3.6e-272	cellulose 1,4-beta-cellobiosidase [EC:3.2.1.91]
CP005080.1_6846	K03553	190.37	720.5	1.1e-216	recombination protein RecA
CP005080.1_6847	K18544	481.53	501.3	3e-151	streptogrisin A [EC:3.4.21.80]
CP005080.1_6847	K18545	376.10	383.6	4.3e-115	streptogrisin B [EC:3.4.21.81]
CP005080.1_6870	K07315	145.67	165.4	7.8e-49	phosphoserine phosphatase RsbU/P [EC:3.1.3.3]
CP005080.1_6871	K07483	40.77	86.6	8.9e-25	transposase
CP005080.1_6872	K07497	13.93	47.4	6.7e-13	putative transposase
CP005080.1_6873	K00986	84.27	270.4	1.3e-80	RNA-directed DNA polymerase [EC:2.7.7.49]
CP005080.1_6874	K07497	13.93	51.5	3.9e-14	putative transposase
CP005080.1_6881	K07497	13.93	93.0	9.8e-27	putative transposase
CP005080.1_6892	K07497	13.93	84.0	5.1e-24	putative transposase
CP005080.1_6895	K02075	260.30	275.5	2.5e-82	zinc/manganese transport system permease protein
CP005080.1_6896	K02013	238.30	238.5	5.5e-71	iron complex transport system ATP-binding protein [EC:7.2.2.-]
CP005080.1_6911	K03826	152.20	152.3	9e-45	putative acetyltransferase [EC:2.3.1.-]
CP005080.1_6916	K07315	145.67	183.9	2e-54	phosphoserine phosphatase RsbU/P [EC:3.1.3.3]
CP005080.1_6916	K04757	53.50	93.7	5.1e-27	serine/threonine-protein kinase RsbW [EC:2.7.11.1]
CP005080.1_6917	K07315	145.67	233.9	1.5e-69	phosphoserine phosphatase RsbU/P [EC:3.1.3.3]
CP005080.1_6918	K04749	63.93	111.3	2.7e-32	anti-sigma B factor antagonist
CP005080.1_6918	K07122	62.20	65.9	1.6e-18	phospholipid transport system transporter-binding protein
CP005080.1_6921	K07315	145.67	151.1	1.7e-44	phosphoserine phosphatase RsbU/P [EC:3.1.3.3]
CP005080.1_6928	K00806	330.87	331.4	2e-99	undecaprenyl diphosphate synthase [EC:2.5.1.31]
CP005080.1_6931	K00433	197.80	454.7	9.6e-137	non-heme chloroperoxidase [EC:1.11.1.10]
